# Transcriptional profiling analysis of *Penicillium digitatum*, the causal agent of citrus green mold, unravels an inhibited ergosterol biosynthesis pathway in response to citral

**DOI:** 10.1186/s12864-016-2943-4

**Published:** 2016-08-11

**Authors:** Qiuli OuYang, Nengguo Tao, Guoxing Jing

**Affiliations:** School of Chemical Engineering, Xiangtan University, Xiangtan, 411105 People’s Republic of China

**Keywords:** Citral, *Penicillium digitatum*, RNA-Seq, Ergosterol biosynthesis

## Abstract

**Background:**

Green mold caused by *Penicillium digitatum* is the most damaging postharvest diseases of citrus fruit. Previously, we have observed that citral dose-dependently inhibited the mycelial growth of *P. digitatum*, with the minimum inhibitory concentration (MIC) of 1.78 mg/mL, but the underlying molecular mechanism is barely understood.

**Results:**

In this study, the transcriptional profiling of the control and 1/2MIC-citral treated *P. digitatum* mycelia after 30 min of exposure were analyzed by RNA-Seq. A total of 6355 genes, including 2322 up-regulated and 4033 down-regulated genes, were found to be responsive to citral. These genes were mapped to 155 KEGG pathways, mainly concerning mRNA surveillance, RNA polymerase, RNA transport, aminoacyl-tRNA biosynthesis, ABC transporter, glycolysis/gluconeogenesis, citrate cycle, oxidative phosphorylation, sulfur metabolism, nitrogen metabolism, inositol phosphate metabolism, fatty acid biosynthesis, unsaturated fatty acids biosynthesis, fatty acid metabolism, and steroid biosynthesis. Particularly, citral exposure affected the expression levels of five ergosterol biosynthetic genes (e.g. *ERG7*, *ERG11*, *ERG6*, *ERG3* and *ERG5*), which corresponds well with the GC-MS results, the reduction in ergosterol content, and accumulation of massive lanosterol. In addition, *ERG11*, the gene responsible for lanosterol 14*α*-demethylase, was observed to be the key down-regulated gene in response to citral.

**Conclusion:**

Our present finding suggests that citral could exhibit its antifungal activity against *P. digitatum* by the down-regulation of ergosterol biosynthesis.

**Electronic supplementary material:**

The online version of this article (doi:10.1186/s12864-016-2943-4) contains supplementary material, which is available to authorized users.

## Background

Significant losses can occur after harvest during storage and marketing of citrus fruit primarily due to green mold, caused by *Penicillium digitatum*, and secondarily by blue mold and sour rot caused by *P. italicum* and *Geotrichum citri-aurantii*, respectively [1]. Among them, *P. digitatum* is the most economically devastating pathogen causing about 90 % of the total loss of postharvest citrus fruit [2]. Currently, the control of postharvest pathogens depends mainly on synthetic chemical application, but extensive use of synthetic chemical has caused the emergence of fungicide-resistant populations, therefore seeking natural and effective microbiocidal agents as alternatives has been immediate areas of research focus [3, 4].

Citral, one of the volatile constituents in plant essential oils, has been demonstrated to have strong antifungal activity against *P. digitatum*, *P. italicum*, and *G. citri-aurantii* [4–6]. Fumigation of oranges with citral (20, 60 or 150 mL/L in absorbent pads) in a closed system, following application of conidia (20 *μ*L of 10^6^ conidia/mL) to puncture wounds, delayed the onset of sour rot at room temperature by 7–10 days and at 5 °C, by 13–30 days [4]. Previously, we have observed that citral inhibited the myclial growths of *P. italicum* and *G. citri-aurantii* in a dose dependent manner [7, 8], and the application of wax enriched with citral significantly decreased the incidence of green mold after 6 days of storage at 25 ± 2 °C [9]. Therefore, it might be an alternative as fungicide in controlling postharvest diseases in citrus fruit.

The antifungal mechanism of volatile compounds has been attributed to its capacity to disturb the cellular membrane, interfere with the cellular metabolism, react with active sites of enzymes, or act as H^+^ carriers [10, 11]. In our previous studies, citral was found to destroy the membrane permeability and integrity of *P. italicum* and *G. citri-aurantii* by causing significant losses in total lipids or ergosterol contents [7, 8]. In addition, citral at a minimum inhibitory concentration (MIC, 1.78 mg/mL) evidently altered the mitochondrial morphology and inhibited the citrate cycle (TCA cycle) of *P. digitatum* [12]. However, information about the inhibitory mechanism of citral on *P. digitatum* at molecular level is rather limited, and thus, requires further studies.

Recently, numerous reports regarding the global gene expression in response to essential oils or their volatile components in fungal have been conducted. Parveen et al. [13] found that the cell wall- and membrane-related genes were major targets of *α*-terpinene against *Saccharomyces cerevisiae*, showing that genes associated with protein analysis, carbohydrate metabolism, and transcription were repressed, whereas genes involved in ergosterol biosynthesis, phospholipid biosynthesis, cell wall organization and detoxification were activated. Rao et al. [14] reported that carvacrol can stimulate the expression levels of genes involved in alternate metabolic and energy pathways, stress response, autophagy, drug efflux, but repress genes mediating ribosome biogenesis and RNA metabolism. A microarray analysis revealed that 1 % (v/v) *p*-anisaldehyde can induce the expression levels of genes related to sulphur assimilation, aromatic aldehydes metabolism, and secondary metabolism in *S. cerevisiae*, suggesting that the normal metabolism of aromatic aldehydes was influenced [15]. In another report, 90 min of exposure to 128 *μ*g/mL thymol resulted in a reduction in intracellular thiamine concentration in yeast by impairing thiamin metabolism. In contrast, many genes involving in maintaining cell membrane and cell wall integrity, sulfur assimilation, methionine biosynthesis, and production of AdoMET were drastically induced [16]. These findings provide some novel clues for the understanding of the antifungal mechanism of plant essential oils.

RNA-Seq is an increasingly attractive method for whole-genome expression studies in many biological systems. As compared with traditional microarrays, despite their instrumental role in profiling the global expression of genes, this technique extends the possibilities of transcriptome studies to the analysis of previously unidentified genes and splice variants due to the advantages such as no need to provide a referenced genomic sequence, a fully coverage of three types of RNA, an unlimited dynamic range of quantification at reduced technical variability, the declining cost of sequencing, and so on [17, 18]. It has been widely used to unravel the pathogenetic mechanisms or diseases control in citrus fruit [19–21]. Therefore, our present study is to analyze the global gene expression profiles in *P. digitatum* mycelia with or without citral treatment by RNA-Seq, in an effort to explore the underlying molecular mechanism and to find some key metabolic pathways or genes involved in.

## Methods

### Fungal cultivation

*P. digitatum* was isolated from infected citrus fruit and preserved on potato dextrose agar (PDA) at 25 ± 2 °C. Two hundred micro liter fungal suspensions (5 × 10^5^ cfu/mL) were added to 40 mL potato dextrose broth (PDB) and incubated in a moist chamber at 28 ± 2 °C for 72 h. The mycelia were vacuum-filtered and weighted at a 6 h interval to make a growth curve.

Based on the result of growth curve, 1 g wet mycelia in logarithmic metaphase (48 h of culture) were added to 20 mL PBS (pH 6.8) and incubated with 1/2MIC (half of minimum inhibitory concentration; 0.89 mg/mL) or MIC of citral for 0, 30, 60, and 120 min. Samples without citral were severed as a control. The resulting mycelia were vacuum-filtered, weighted, and recorded to select optimal concentration and time for the next analysis. All collected *P. digitatum* mycelia were grinded to powders in liquid nitrogen and stored at −80 °C until further use.

### RNA isolation, integrity examination and RNA-Seq library preparation

Total RNAs from control, 1/2 MIC or MIC citral-treated samples after 30 min of exposure were extracted with TRIzol regent (Invitrogen, USA) according to the manufacturer’s instruction and treated with RNase-free DNase I (Takara Biotechnology, China). RNA integrity was determined by a 2100 bioanalyzer (Agilent, USA). Poly (A) mRNA from control and 1/2MIC citral-treated samples, designated as CK30 and T30, respectively, was isolated with oligo-dT beads and then treated with the fragmentation buffer. The cleaved RNA fragments were then transcribed into first-strand cDNA using reverse transcriptase and random hexamer primers, followed by second strand cDNA synthesis using DNA polymerase I and RNaseH. The double stranded cDNA was further subjected to end-repair using T4 DNA polymerase, Klenow fragment, and T4 Polynucleotide kinase, followed by a single A base addition using Klenow 3′ to 5′ exo-polymerase. It was then ligated with adapter or index adapter using T4 quick DNA ligase. Adaptor ligated fragments were selected according to the size and the desired range of cDNA fragments were excised from the gel. PCR was performed to selectively enrich and amplify the fragments. Finally, after validating on an Agilent 2100 Bioanalyzer and ABI StepOnePlus Real-Time PCR System, the cDNA library was sequenced on a flow cell using Illumina HiSeq2000 ™ [22].

### Assembly and functional annotation

Transcriptome de novo assembly of the short reads was carried out using Trinty software [23]. After filtration of the low quality reads, clean reads were randomly clipped into 25-bp k-mers and assembled using de Bruijin graph and Trinty software. Furthermore, the reads producing large fragment without N is named contig and the result sequences of trinity is called unigenes. Then unigenes were clustered using TGI Clustering tools to obtain the finally unigenes [24]. Finally, blastx alignment (*e* value < 0.00001) between unigenes and protein databases (non-redundant protein (NR), Swiss-Prot, Kyoto Encyclopedia of Genes and Genomes (KEGG) and the Clusters of Orthologous Groups of proteins (COG)) were performed, and the best aligning results were selected as a priority order of NR, Swiss-Prot, KEGG and COG to determine the sequence orientation. When a unigene was not aligned to the above databases, ESTScan software [25] was used to predict its coding regions and to determine its sequence orientation. Gene ontology (GO) terms annotations of unigenes were performed with the Blast2GO program according to the NR annotation. The COG and KEGG pathway annotations were analyzed using Blastall software against the cluster of orthologous groups’ database and the kyoto encyclopedia of genes and genomes database.

### Differential expression of unigenes

The expression level of unigene is calculated by Fragments Per kb per Million reads (FPKM) method described by Audic and Claverie [26]. An expressed equally between two samples was calculated with the following formula:

$$ p\left(i\Big|x\right)={\left(\frac{N_2}{N_1}\right)}^i\frac{\left(x+i\right)!}{x!i!{\left(1+\frac{N_2}{N_1}\right)}^{\left(x+i+1\right)}} $$

Where, N_1_ is the total fragments number of the CK30, and N_2_ is total fragments number of T30; gene A holds x fragments in CK30 and i fragments in T30. The larger difference of the expression level between the CK30 and T30 is calculated with FDR ≤ 0.001 and log_2_ Ratio ≥ 1.

### GO annotation and pathway analysis of differentially expressed genes (DEGs)

First, mapping all DEGs to each term of gene ontology database and calculating the gene numbers for each GO term has to get a gene list and gene numbers for every certain GO term, then using hypergeometric test to find significantly enriched GO terms in DEGs comparing to the genome background. The *P* value calculating formula in this hypothesis test is.

$$ P=1-{\displaystyle \sum_{i=0}^{m-1}\frac{\left(\begin{array}{c}\hfill M\hfill \\ {}\hfill i\hfill \end{array}\right)\left(\begin{array}{c}\hfill N-M\hfill \\ {}\hfill n-i\hfill \end{array}\right)}{\left(\begin{array}{c}\hfill N\hfill \\ {}\hfill n\hfill \end{array}\right)}} $$

Where, in GO analysis, N is the number of all genes with GO annotation; n is the number of DEGs in N; M is the number of all genes that are annotated to the certain GO terms; m is the number of DEGs in M. The calculated *p* value goes through Bonferroni Correction, taking corrected-*P* value ≤ 0.05 as a threshold. In KEGG analysis, N is the number of all genes with KEGG annotation, n is the number of DEGs in N, M is the number of all genes annotated to specific pathways, and m is number of DEGs in M.

### Real-time Fluorescence Quantitative PCR (RTFQ-PCR) analysis

RNA was extracted from *P. digitatum* cells exposure to citral at various concentrations (0 and 1/2MIC) for 0, 30, 60, and 120 min using the Trizol reagent (Invitrogen, USA) following the manufacturer’s instructions. Two micrograms of DNA-free RNA were used for reverse transcription by M-MLV (Promega, USA) with oligo dT18. RTFQ-PCR was performed on a BIO-RAD CFX Connect Thermal Cycler using FastStart Universal SYBR Green Master (Roche, Switzerland). All primer pairs for expression assays are listed in Table 1. RTFQ-PCR was programmed as follows: 95 °C for 10 min followed by 40 cycles of 95 °C for 15 s, 60 °C for 1 min. The 2^-△△CT^ method was used to quantify the value of every sample using actin gene as an internal reference [27].

**Table 1:** Primer pair sequences designed for validation of differentially expressed genes in CK30 and T30 treatment *P. digitatum* using Real-time Fluorescence Quantitative PCR (RTFQ-PCR)

Gene ID	Gene name	Primer sequence (5′-3′, forward/reverse)
Unigene6144	*Actin*	TGCGCTGAACCGAACTGCCG TCGGGAGCCTCGAAGCGCTC
Unigene8313	*ERG7*	GCGCTGGCGATTGGTCGATG
		CAGGCCCAGTTTCCGGGCTCC
Unigene8539	*ERG11*	CCATCGACCTCGTCCCCGCC
		TCGCGCTTGCGGTTTTGGGG
Unigene6797	*ERG6*	CGCGTGATGCCGCCTTCAAC
		TGAGCCTTGCGGGCCTCACG
Unigene5828	*ERG3*	CAGGCCATGGCCGCAATGCC
		GGTGCAGGCCACGGTGGATCC
Unigene8125	*ERG5*	TCTCGCCATTGGCGGATGCG
		TCTCGCCATTGGCGGATGCG

### Sterols compositions by gas chromatography-mass spectrometry (GC-MS)

The sterols compositions of *P. digitatum* cells exposure to citral at a concentration of 1/2MIC for 0, 30, 60, and 120 min were determined by GC-MS method [28, 29] with some modifications. The 2-day-old mycelia from 50 mL PDB were collected and centrifuged at 4000 g for 15 min. Then the samples were dried with a vacuum freeze drier for 4 h. About 0.5 g of dry mycelia were homogenized with liquid nitrogen and were suspended in 4 mL of freshly prepared 25 % (w/v) NaOH and 8 mL of absolute ethanol, vortexed for 30 sec and sonicated for 10 min, and then saponified at 80 °C for 1 h. The mixtures were extracted with 2 mL petroleum ether for three times and washed by saturated NaCl solution twice. The organic upper layer was transferred into a new 10.0 mL plastic microcentrifuge safe-lock tube containing 40 ± 2 mg anhydrous sodium sulphate. The samples were then vacuum concentrated. Each residue was dissolved in 950 μL of methyl tert-butyl ether and 50 μL of silylation reagent mixture N-methyl-N-trimethylsilyltrifluoroacetamide (MSTFA) and N-trimethylsilylimidazole (TSIM) (9:1) was added. The samples were gently shaken and stored for completion of the silylation reaction at room temperature for at least 30 min, before being subjected to GC–MS analysis. The samples without citral treatment were used as a control.

The analytical GC was carried out on a Shimadzu QP2010 plus gas chromatograph (Shimadzu, Kyoto, Japan) equipped with flame ionization detector (FID). A non-polar cross-linked fused-silica capillary column, HP-5MS (30 m × 0.25 mm × 0.25 μm; Agilent, Santa Clara, CA), was used. The oven temperature was held at 50 °C for 1 min, programmed at a rate of 50 °C/min to 260 °C, then increased to 300 °C at a rate of 4 °C/min where it remained for 10 min. The carrier gas was helium (1.3 mL/min). The injector temperature was 250 °C, detector temperature 310 °C and the volume injected was 2 μL. MS analysis was carried out on the same chromatograph equipped with a Shimadzu QP 2010 GC/MS system, ionisation voltage 70 eV, ion source temperature 200 °C, mass range m/z 50–700, scanning interval 0.5 s and scanning speed 1000 amu/s. The sterol TMS ethers were identified by comparison with commercial references, the NIST™ database or data from literature.

### Determination of total ergosterol and lanosterol contents

Total ergosterol and lanosterol contents of *P. digitatum* cells exposure to citral at a concentration of 1/2MIC for 0, 30, 60, and 120 min were determined by high-performance liquid chromatography (HPLC) as reported by our previous study [7], whereas the detected wavelength for lanosterol was set at 210 nm. The samples without citral treatment were used as a control.

### Statistical analysis

All data were expressed as the mean ± SD by measuring three independent replicates. Analysis of variance using one-way ANOVA followed by Duncan’s test was performed to test the significance of differences between means obtained among the treatments at the 5 % level of significance using SPSS statistical software package release 16.0 (SPSS Inc., Chicago, IL, USA).

## Results and discussions

### The growth of *P. digitatum*

**Fig. 1:**
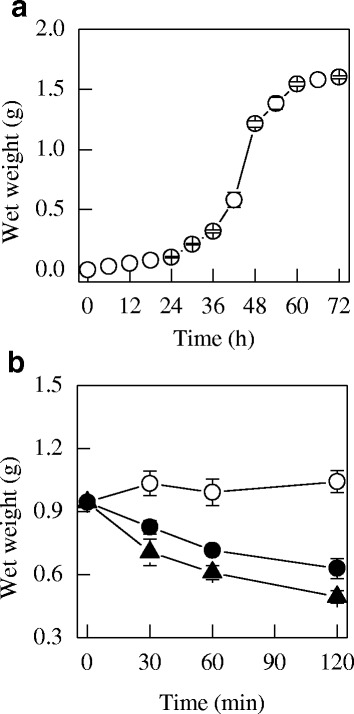
**a** The growth curve of *P. digitatum* mycelia; **b** The wet weight of *P. digitatum* mycelia exposed to citral, (○) CK; (●) 1/2MIC; (▲) MIC. Values are the mean ± SD of three measurements

The growth curve of *P. digitatum* is shown in Fig. 1a. During the first 24 h of initial incubation, the growth of *P. digitatum* cells was slow and remained in the lag phase. Then *P. digitatum* cells visibly grew and reached the logarithmic phase at 60 h of incubation. Correspondingly, the mycelia of *P. digitatum* that grew for 48 h, which was suggested to be in the logarithmic metaphase, were chosen for the next analysis.

To determine the effects of citral on the growth of *P. digitatum*, the wet weight of *P. digitatum* mycelia exposed to citral was determined. As shown in Fig. 1b, citral markedly inhibited the mycelial growth of *P. digitatum* during the whole period, whereas that of the control group remained stable. After 30 min of exposure, the wet weight of 1/2MIC and MIC mycelia were 0.83 ± 0.03 g and 0.71 ± 0.06 g, respectively, which were significantly lower than that of the control (0.99 ± 0.06 g, *P* < 0.05). This inhibition was more obvious with the prolonging of the culture time. Considering that a RNA integrated number (RIN) value higher than 6.5 was the prerequisite for the RNA-Seq analysis, we further determined the RIN values in citral-treated or control samples at 30 min incubation. Results showed that the RIN values in 1/2 MIC or MIC citral -treated and control groups were 6.7, 2.6, and 7.9, respectively (data not show), suggesting that MIC citral -treated samples were not suitable for further analysis. Therefore, only samples treated with 1/2MIC citral (T30) or without citral treatment (CK30) were chosen for RNA-Seq analysis.

### Overall transcriptome profiles *P. digitatum*

**Fig. 2:**
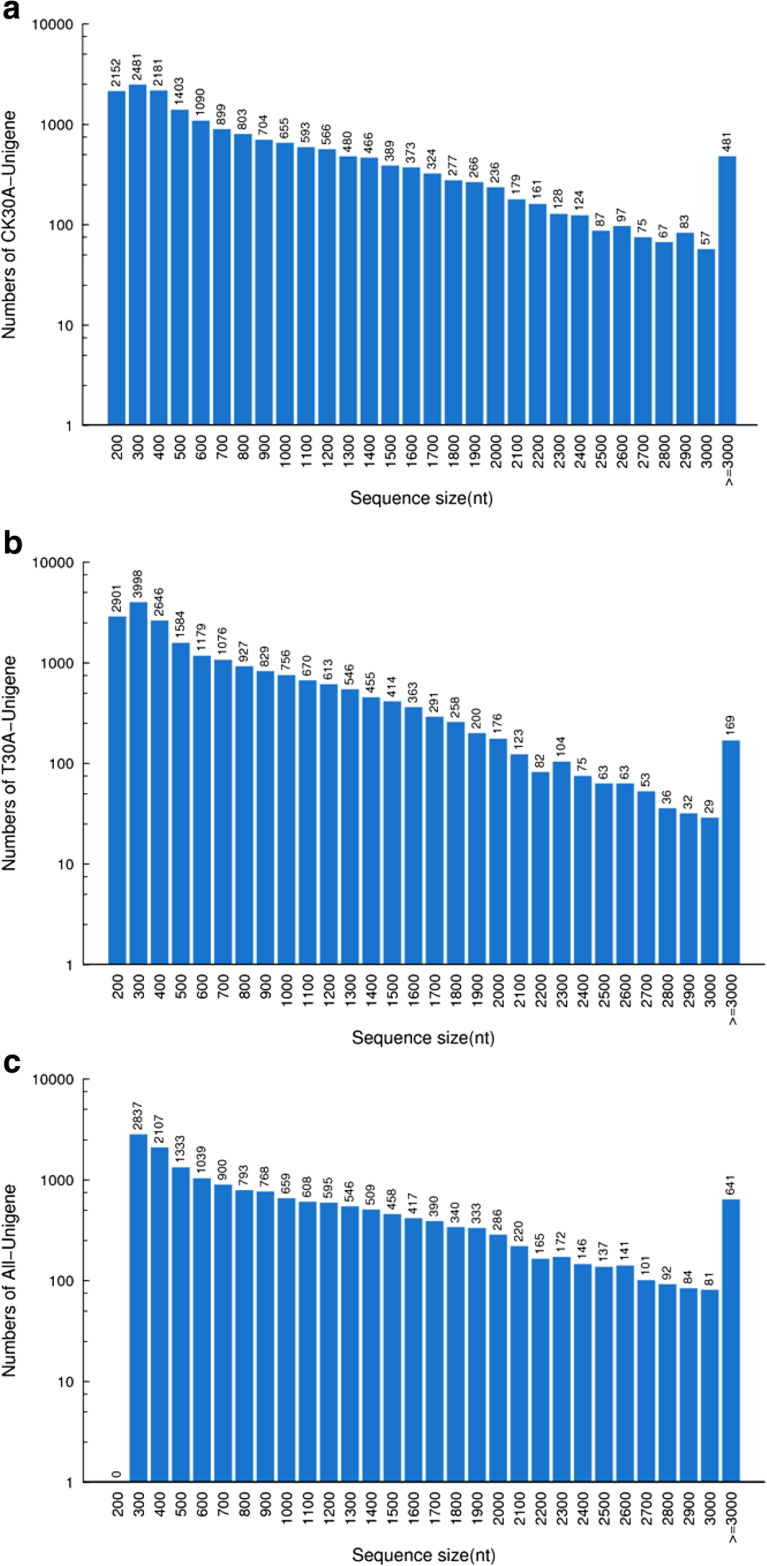
The length distribution of unigenes after de novo assembly using the reads from CK30, T30, and CK30&T30 samples, the horizontal coordinates are unigene lengths and the vertical coordinates are numbers of unigenes

**Fig. 3:**
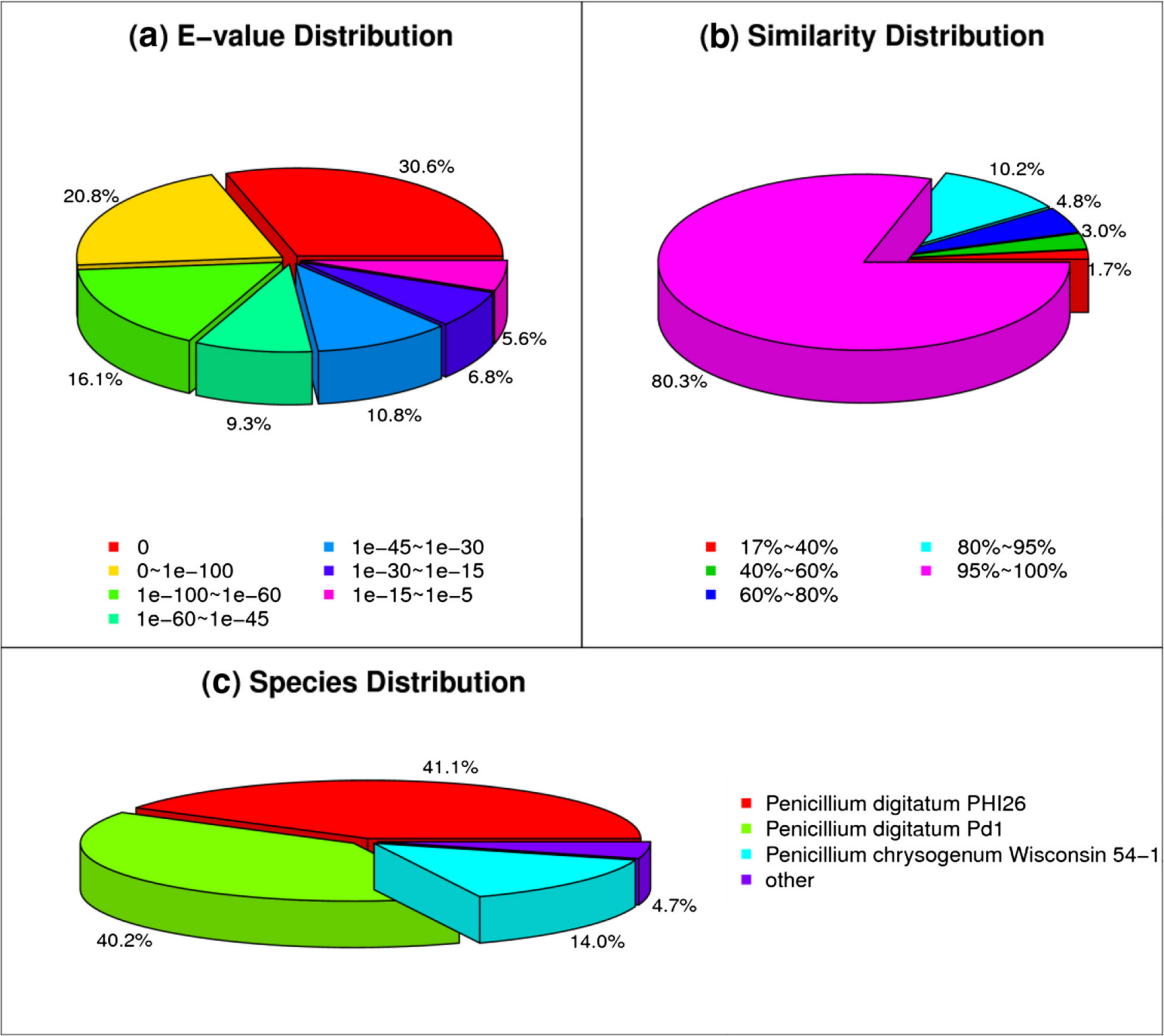
Species distribution of the BLASTx matches (with an *E*-value < 10^−5^) to the unique transcripts against the non-redundant protein database. **a** The E-value distribution of NR annotation result; **b** The similarity distribution of NR annotation result; **c** The species distribution of NR annotation result

To analyze the transcriptome profiles in CK30 and T30, a pooled cDNA sample from each strain was separately sequenced with the Illumina sequencing platform. The details in assembly and annotation information are shown in Table 2. After filtering the dirty raw reads, 25 and 27 million clean reads were obtained from CK30 and T30, respectively. Totally, 16898 unigenes were used for functional annotation and 14189 coding sequence (CDS) were mapped to the protein database or predicted (Additional file 1: Table S1 and Additional file 2: Table S2). Among them, 14235, 14555, 8584, 8714, 5832, and 9158 unigenes were annotated to NR, non-redundant nucleotide sequence (NT), Swiss-Prot, KEGG, COG, and GO database, respectively. The size distribution showed that the lengths of the CK30, T30, and all- unigenes were no more than 3000 nt (Fig. 2), and the distribution of unigenes was decreased with the increase of sequence size. The 300 nt sequences were mostly distributed unigenes in CK30 and T30, followed by 200 or 400 nt, whereas only a few 3000 nt sequences and no 200 nt sequences were found. After searching in the National Center for Biotechnology Information (NCBI) nucleotide database by blastx using unigenes obtained from CK30 and T30, the major transcriptome profiles of *P. digitatum* were significantly matched to *P. digitatum* PHI26 (41.1 %), *P. digitatum* Pd1 (40.2 %), *P. chrysogenum* Wisconsin 54–1 (14.0 %), and other fungi (4.7 %) (Fig. 3).

**Table 2:** Summary data of reads in untreated strains (CK30) and citral-treated strains (T30) of *P. digitatum* mycelia transcriptomes

Parameters	CK30	T30	CK30 & T30
Total clean reads	25,722,906	27,714,798	-
Total clean nucleotides (nt)	2,315,067,540	2,494,331,820	
Q20 percentage	97.61 %	97.45 %	
N percentage	0.01 %	0.01 %	
GC percentage	52.76 %	45.2 %	-
Total number of contigs	23,279	29,572	-
Mean length of contigs (nt)	612	471	-
The number of unigenes	17,877	20,711	-
Mean length of unigenes (nt)	859	688	-
The number of all-unigenes	-	-	16898
Mean length of unigenes	-	-	1017
Unigenes annotation against NR		14,235	
Unigenes annotation against NT		14,555	
Unigenes annotation against Swiss-Prot		8,584	
Unigenes annotation against KEGG		8,714	
Unigenes annotation against COG		5,832	
Unigenes annotation against GO		9,158	
Up-regulated genes		2,322	
Down-regulated genes		4,033

**Fig. 4:**
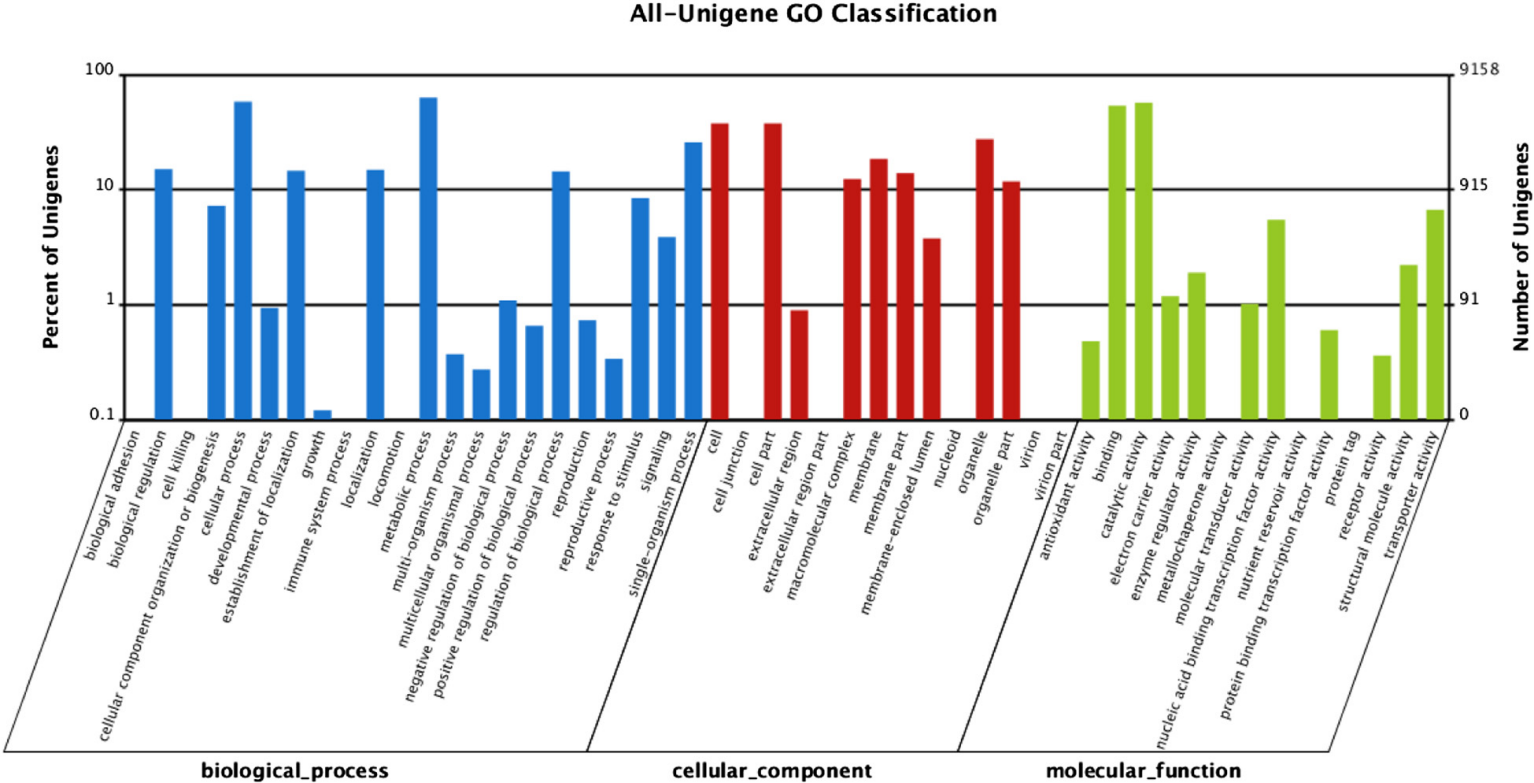
GO classification analysis of unigenes in all-unigene. GO functions is showed in X-axis. The right Y-axis shows the number of genes which has the GO function, and the left Y-axis shows the percentage

**Fig. 5:**
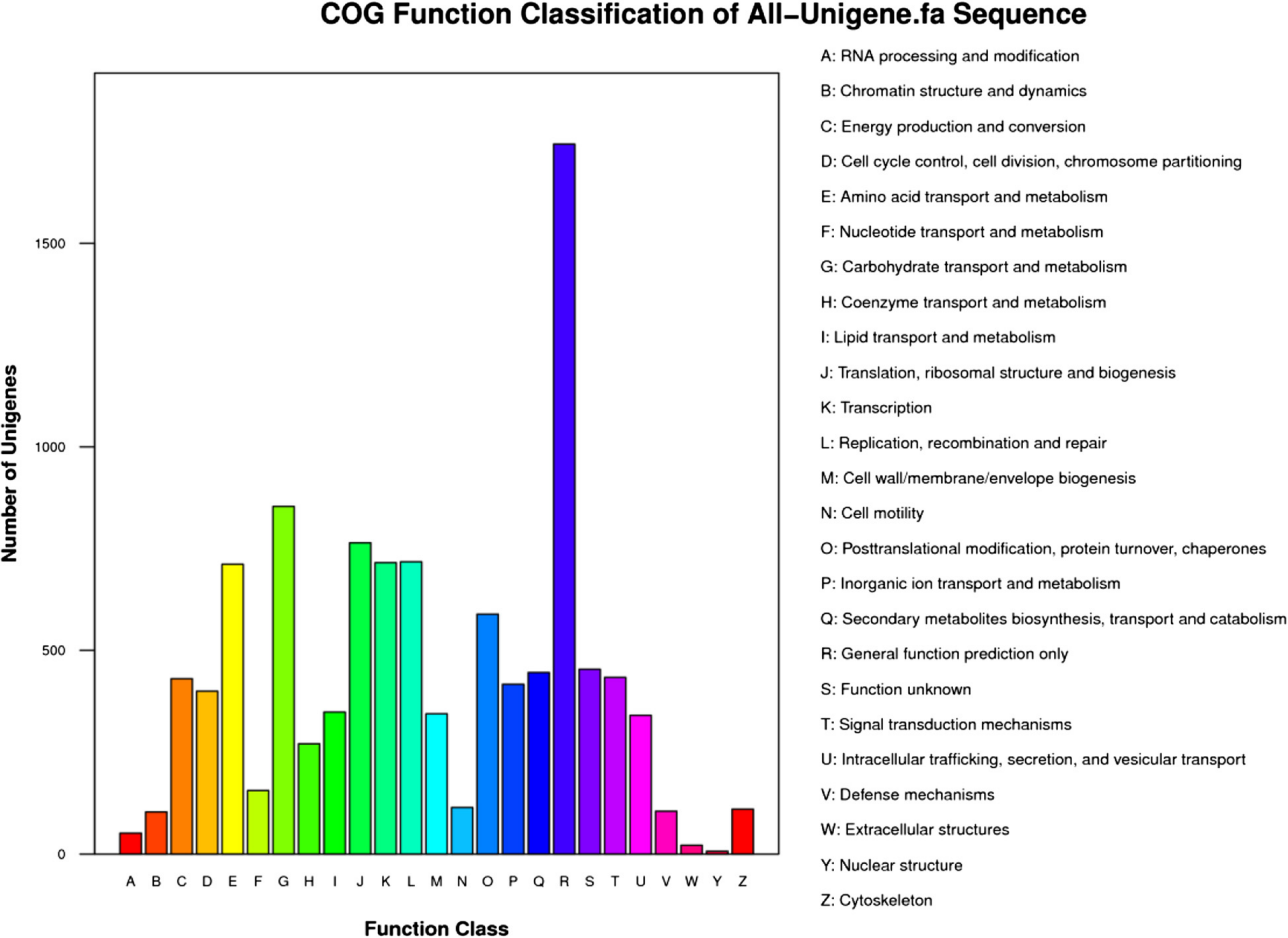
Clusters of orthologous groups (COG) function classification of unigenes in all-unigene. The horizontal coordinates are COG function classes, and the vertical coordinates are numbers of unigenes in one class. The notation on the right is the full name of the functions in X-axis

Based on functional annotation of unigenes, the predicted genes were further classified by Blast2GO analysis (Table 2) and 9158 unigenes from all-unigenes were categorized into 47 GO terms distributed into biological process, cellular component, and molecular function (Fig. 4). Clearly, the cellular process, metabolic process, and catalytic activity were the dominant categories. Genes involved in binding, cell, cell part, organelle, growth, multicellular organismal process, reproductive process, and receptor activity were found in those categories, while those related to biological adhesion, virion, virion part, cell killing, or locomotion were barely detected (Fig. 4). For the COG analysis, 5832 unigenes (Table 2) from all unigenes were categorized to 26 categories (Fig. 5). The most distributed one was the cluster of general function prediction (1744), followed by carbohydrate transport and metabolism (854), translation, ribosomal structure and biogenesis (765), and amino acid transport and metabolism (712).

A total of 16,898 all-unigenes were mapped to the referenced canonical pathways in KEGG, 8714 of them were annotated to 168 KEGG pathways. Among of which, 2550, 1087, and 898 unigenes were distributed to metabolic pathway, biosynthesis of secondary metabolites, and microbial metabolism in diverse environments, respectively. Few unigenes correlated with carotenoid biosynthesis (1 unigene), phosphotransferase system (PTS, 1 unigene), and sesquiterpenoid and triterpenoid biosynthesis (2 unigenes) were also found.

### Differences in overall gene expressions and pathway between CK30 and T30

The differences in gene expressions between CK30 and T30 were further analyzed (Additional file 3: Table S3). The variations of gene length and total reads distributions in gene expression levels were eliminated by FPKM calculation. The different gene expressions were calculated by false discovery rate (FDR ≤ 0.001) and the absolute value of log_2_Ration ≥ 1 as a threshold. A total of 6355 differential expression genes were detected between CK30 and T30, including 2322 up-regulated and 4033 down-regulated genes (Table 2). The distribution of down-regulated genes was nearly twice as that of up-regulated genes, which might be caused by the inhibition of *P. digitatum* mycelial growth.

A total of 3232 differentially expressed genes (DEGs) were mapped to the KEGG database and summed up to 155 pathways. The specific pathways associated with environmental information processing, such as ABC transport (64, 1.98 %), MAPK signaling pathway (88, 2.72 %), two-component system (48, 1.49 %), phosphatidylinositol signaling system (22, 0.68 %); the pathways belonged to cell membrane, such as fatty acid biosynthesis (28, 0.87 %), biosynthesis of unsaturated fatty acids (14, 0.43 %), steroid biosynthesis (20, 0.62 %); the pathways involved in energy, such as nitrogen metabolism (38, 1.18 %), oxidative phosphorylation (47, 1.45 %), glycolysis/gluconeogenesis (64, 1.98 %), and TCA cycle (21, 0.65 %); the pathways related to genetic information, such as ribosome biogenesis in eukaryotes (78, 2.41 %), RNA degradation (91, 2.82 %), RNA polymerase (24, 0.74 %), aminoacyl-tRNA biosynthesis (29, 0.9 %) and ribosome (33, 1.02 %), were all affected by citral treatment (Table 3).

**Table 3:** Enrichment pathway analysis of DEGs in *P. digitatum*

Pathway	DEGs genes with pathway annotation (3232)	All genes with pathway annotation (8714)	Pathway ID
ABC transporters	64 (1.98 %)	134 (1.54 %)	ko02010
Ribosome biogenesis in eukaryotes	78 (2.41 %)	171 (1.96 %)	ko03008
Alanine, aspartate and glutamate metabolism	40 (1.24 %)	83 (0.95 %)	ko00250
Starch and sucrose metabolism	175 (5.41 %)	426 (4.89 %)	ko00500
RNA degradation	91 (2.82 %)	213 (2.44 %)	ko03018
Fatty acid biosynthesis	28 (0.87 %)	62 (0.71 %)	ko00061
Non-homologous end-joining	21 (0.65 %)	45 (0.52 %)	ko03450
MAPK signaling pathway	88 (2.72 %)	221 (2.54 %)	ko04011
Amino sugar and nucleotide sugar metabolism	98 (3.03 %)	248 (2.85 %)	ko00520
Fructose and mannose metabolism	90 (2.78 %)	230 (2.64 %)	ko00051
Phosphatidylinositol signaling system	22 (0.68 %)	53 (0.61 %)	ko04070
Sulfur metabolism	16 (0.5 %)	38 (0.44 %)	ko00920
Nitrogen metabolism	38 (1.18 %)	98 (1.12 %)	ko00910
Meiosis	80 (2.48 %)	210 (2.41 %)	ko04113
Inositol phosphate metabolism	30 (0.93 %)	79 (0.91 %)	ko00562
Purine metabolism	121 (3.74 %)	326 (3.74 %)	ko00230
Citrate cycle (TCA cycle)	21 (0.65 %)	56 (0.64 %)	ko00020
Linoleic acid metabolism	36 (1.11 %)	97 (1.11 %)	ko00591
Glycolysis/Gluconeogenesis	64 (1.98 %)	173 (1.99 %)	ko00010
mRNA surveillance pathway	55 (1.7 %)	150 (1.72 %)	ko03015
Glutathione metabolism	29 (0.9 %)	80 (0.92 %)	ko00480
Pentose phosphate pathway	17 (0.53 %)	48 (0.55 %)	ko00030
RNA transport	94 (2.91 %)	267 (3.06 %)	ko03013
Pyrimidine metabolism	86 (2.66 %)	247 (2.83 %)	ko00240
RNA polymerase	24 (0.74 %)	73 (0.84 %)	ko03020
DNA replication	32 (0.99 %)	96 (1.1 %)	ko03030
Peroxisome	42 (1.3 %)	127 (1.46 %)	ko04146
Steroid biosynthesis	20 (0.62 %)	64 (0.73 %)	ko00100
Oxidative phosphorylation	47 (1.45 %)	144 (1.65 %)	ko00190
Biosynthesis of unsaturated fatty acids	14 (0.43 %)	53 (0.61 %)	ko01040
Fatty acid metabolism	43 (1.33 %)	147 (1.69 %)	ko00071
Aminoacyl-tRNA biosynthesis	29 (0.9 %)	109 (1.25 %)	ko00970
Plant-pathogen interaction	1 (0.03 %)	11 (0.13 %)	ko04626
Ribosome	33 (1.02 %)	224 (2.57 %)	ko03010

### Genes related to stress response

To survive from drastic environment changes, eukaryotes must rapidly adjust their gene expression programs to develop a series of protective mechanisms [30, 31]. In this study, considerable alterations in gene expression levels related to cell rescue, defense, and detoxification were influenced by citral. Genes associated with the over-represented pathway, such as ribosome biogenesis in eukaryotes, mRNA surveillance pathway, RNA polymerase, RNA transport and aminoacyl-tRNA biosynthesis, were partially repressed, indicating a lesion in the translational activity of cells. This result, to a large extent, accounts for the low RIN value (2.6) in *P. digitatum* cells treated with MIC of citral for 30 min. In addition, one gene responsible for mitochondrial ribosomal protein (*MRPS5*) was found to be down-regulated, indicating a blocking in mitochondrial translation and the loss of mitochondrial functions [32]. Correspondingly, a repression in energy-related pathways including TCA cycle, glycolysis/gluconeogenesis, oxidative phosphorylation, sulfur metabolism, and nitrogen metabolism, was also observed. Most genes in these pathways, such as *SDH1*, *frdA*, *CS*, *IDH1*, *HK*, *PFK*, *PK*, *fbcH*, *ATPeF1G*, *ATPeV0C*, *cysQ*, *cysJ*, *aspA*, *nasB*, and *glnA*, were down-regulated. Previously, we have demonstrated that citral treatment could evidently alter the mitochondrial morphology, reduce the ATP content and inhibit the TCA cycle of *P. digitatum* [12]. These results supported the hypothesis that the down-regulation of energy-demanding processes is a very important approach to help the cells escaping from energy crisis under extreme environmental conditions [33]. Interestingly, significant changes in genes involved in the environmental stress response were also observed, which was consistent with a previous report describing the global gene expressions in *S. cerevisiae* cells treated with thymol [16].

### Genes involved in multidrug resistance

The typical gene subfamilies involved in multidrug resistance in fungal, such as multi-drug resistance (MRD, ABCB subfamily), multi-drug resistance-associated protein (MRP, ABCC subfamily), and pleiotropic drug resistance (PDR, ABCG subfamily), were affected by citral. A slight increase in the MRD gene *PMR1* expression was observed in samples exposed to citral. This gene was an important determinant of resistance to demethylation inhibitor-resistant strains, which is normally suggested to protect the cells against triflumizole [34]. Genes belonged to plasma membrane ATP-binding cassette (ABC) transporters, such as *PDR5* and *SNQ2*, were also up-regulated. These two genes were involved in a robust induction of drug efflux mechanisms, and their expression levels in *S. cerevisiae* cells have been demonstrated to be strongly induced by thymol [16]. Interestingly, the expression levels of five MRP genes including *MRP1*, *MRP4*, *MRP5*, *MRP7*, and *MRP13*, were down-regulated. This type of genes generally transports xenobiotic compounds or toxic metabolites that conjugated to glutathione, glucuronide, or sulfur, and the over-expression of *MRP1* gene can enhance the multidrug resistance in eukaryotic cells [35]. Therefore, the decrease in expression levels of MRP genes suggested that the multidrug resistance in *P. digitatum* cell might be impaired by citral.

### Genes associated with cell integrity

Several targets including cell wall, cell membrane, mitochondrion, and genetic material, have been proposed to account for the antifungal activity of essential oils or their volatile compositions [3, 12–16, 36]. The cell wall is an extracellular layer outside the cell membrane which protects the cell against mechanical damage, osmotic strength and determines the cell shape. We found that several pathways associated with cell wall biogenesis, such as amino sugar and nucleotide sugar metabolism, starch and sucrose metabolism, were drastically influenced by citral. *CHI1*, *Fks1*, two important cell wall-related genes encoding for chitin synthase and 1,3-β-glucan synthase, respectively, were repressed. This finding underlined that citral can block the formation of cell wall architecture and thereby improve the sensitivity of cells to citral. In contrast, Parveen et al. [13] reported that theses two genes were dramatically induced in *S. cerevisiae* cell exposed to *α*-terpinene, which in turn activated cell wall compensatory mechanism to overcome the terpinene toxicity. This discrepancy might be, to a large extent, caused by different strains, or by different antifungal mechanisms, as suggested by other reports [36, 37].

The lipophilic nature of terpenoid enables it preferentially enter into the lipid membrane, which results in the increased membrane fluidity and eventually leads to the increase of membrane permeability [38, 39]. The expression levels of some genes involved in cell membrane-related pathways, such as inositol phosphate metabolism, fatty acid biosynthesis, biosynthesis of unsaturated fatty acids, fatty acid metabolism, and steroid biosynthesis, were changed upon citral treatment. Among them, *INO1*, *PI4K*, *CDIPT*, *accC*, *FAS1*, *FAS2*, *FAB1*, *ERG7*, *ERG11*, *ERG6*, *ERG3*, *ERG5* were down-regulated, suggesting that the integrity of cell membrane might be destroyed. Oppositely, expression of *FAD6* was increased by 12.4 folds. These results were consistent with our previous study that citral could induce the disruption of membrane integrity by causing a decrease in total lipid contents of pathogen cells [8].

Ergosterol is one of the principal sterol components in fungal membrane. Generally, a decrease in ergosterol contents results in osmotic disturbances, disruption of cell growth and proliferation [7, 39–41]. Some chemical fungicides widely used in controlling the green mold of citrus fruit, such as imazalil, prochloraz, and triflumizole, exhibited their antifungal mode by blocking the ergosterol biosynthesis, which can give rise to the disruption of cell structure and function, even to the death of cell [42–44]. Notably, the expression levels of five genes in ergosterol biosynthetic pathway encoding for lanosterol synthase (*ERG7*), lanosterol 14*α*-demethylase (*ERG11*), C-24 sterol methyl-transferase (*ERG6*), C-5 sterol desaturase (*ERG3*), and C-22 sterol desaturase (*ERG5*), were down-regulated by 2.7, 2.1, 2.0, 2.1 and 2.0 folds, respectively.

### Effect of citral on gene expression levels in ergosterol biosynthesis

**Fig. 6:**
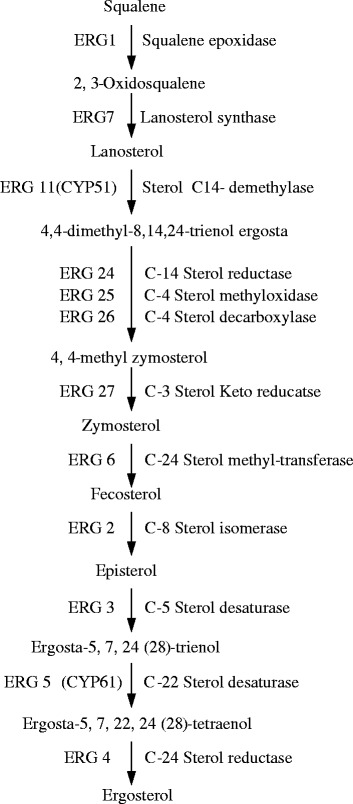
Factors and intermediates of fungal ergosterol biosynthetic pathway

To verify the RNA-Seq results, we investigated the gene expression levels of ergosterol biosynthetic genes mentioned above. These genes serve indispensable roles in ergosterol biosynthetic pathway (Fig. 6). *ERG7* is responsible for the conversion of the last acyclic sterol precursor into lanosterol, the initial cyclic intermediate in the ergosterol biosynthesis [45, 46]. *ERG11* is well-known to be the target of antifungal drugs [41, 43, 47]. *ERG6* catalyzes the accumulation of fecosterol, the intermediates of ergosterol biosynthesis, and a mutated *ERG6* gene makes the *C. albicans* cell loss the ability to synthesize ergosterol [48]. *ERG3* introduces the C5/6 unsaturation in ergosterol biosynthesis [49]. *ERG5* catalyzes the biosynthesis of ergosta-5,7,22,24(28)-trienol, a direct precursor for ergosterol biosynthesis [50]. Generally, commercial chemical fungicides, such as imazalil, ketoconazole, fluconazole, clotrimazole and dodemorph, could affect the gene expression levels of *ERG11*, *ERG6, ERG3*, and *ERG5*, thereby block the ergosterol biosynthesis, lead to the impairment in cell membrane functional and ultimately induce the occurrence of cell disruption [40, 41, 43, 44]. Particularly, *ERG11* is the target site of some commercial chemical fungicides against *P. digitatum* [41, 43].

**Fig. 7:**
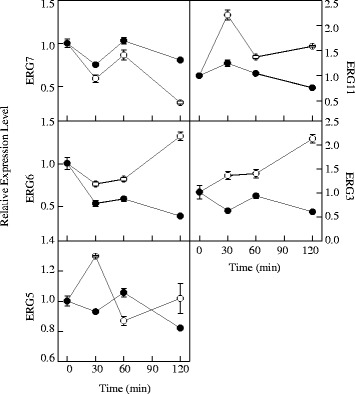
Changes in the expression of ergosterol biosynthesis genes of *P. digitatum* mycelia treated by CK and 1/2MIC citral for 0, 30, 60 and 120 min, (○) CK; (●) 1/2MIC. Values are the mean ± SD of three measurements

As revealed by Fig. 7, the test genes followed a similar changing tendency but differed in expression levels in control and 1/2MIC citral-treated samples. The expression of *ERG7* in control and 1/2MIC citral-treated samples decreased before 30 min of treatment, with the expression level higher in 1/2MIC citral-treated samples than that in control samples. In contrast, the gene expressions of *ERG11*, *ERG6*, *ERG3*, and *ERG5* at 30 min of treatment were dramatically repressed and the expression levels in 1/2MIC citral-treated groups were significantly lower than those in control groups by 0.96, 0.23, 0.74, and 0.36 folds, respectively (Fig. 7). These results were consistent with those of RNA-Seq analysis except the up-regulation of *ERG7*. After 30 min of exposure, the gene expressions of *ERG6* and *ERG3* increased first and then decreased in 1/2MIC citral-treated samples, whereas the expression levels of *ERG6* and *ERG3* were still lower than those of control groups. Moreover, the gene expression of *ERG11* in citral-treated samples was continuously down-regulated and was markedly lower (*P* < 0.05) than that of control samples during the entire period. In contrast, the gene expression levels of *ERG5* in citral-treated samples were remarkable higher (*P* < 0.05) than that in control samples at 60 min incubation. Taken these together, it can be concluded that citral can affect the expression levels of genes in ergosterol biosynthetic pathway of *P. digitatum*, and *ERG11* might be an important target site of citral against *P. digitatum*.

### Analysis of sterols composition by GC-MS

Essential oils have been illustrated to inhibit the growth of fungi by decreasing ergosterol content, affecting membrane structure, and disrupting sterol biosynthesis [13, 39, 50, 51]. Previously, we have demonstrated that total ergosterol contents in *P. italicum* cells were considerably decreased by citral [7]. To illustrate the effect of citral on sterols biosynthesis, we firstly determined the sterols composition of *P. digitatum* by GC-MS. The results are shown in Table 4. Nine sterols including lanosterol, ergosta-5,7,24(28)-trienol, ergosta-7,22-dienol, ergosta-5,7-dienol, ergosterol, cholesterol, lanosta-7,9(11)-dienol, ergost-4,7,22-trienol, and 4α-methylergosta-7,24(28)-dienol were tentatively identified by comparison with commercial references, the NIST™ database or data from literature [28, 52]. Among them, lanosterol, ergosta-5,7,24(28)-trienol, and ergosterol are the key intermediates in the ergosterol biosynthetic pathway, whereas ergosta-7,22-dienol, ergosta-5,7-dienol, and cholesterol are involved in the branched metabolic pathway related with ergosterol biosynthesis [28, 45, 46, 52]. At 30 min of treatment, ergosta-5,7,24(28)-trienol, the substrate for *ERG5* [28, 29], was exclusively identified in 1/2MIC citral-treated group. In contrast, ergosta-5,7-dienol, a sterol in the branched metabolic pathway [28], was exclusively identified in the CK group. With prolongation of treatment, ergosta-7,22-dienol, one of the substrates for *ERG3*, was absent in 1/2MIC citral-treated groups. Meanwhile, lanosterol and ergosterol were present in all test samples. These results indicate that the target site of citral against *P. digitatum* might be the upstream genes before *ERG 3,* which are consistent with the gene expression results.

**Table 4:** The sterols composition of *P. digitatum* by GC-MS

Substance	Rt (min)	Major ions [m/z]	CK group				1/2MIC citral-treated group		
			0 min	30 min	60 min	120 min	30 min	60 min	120 min
Lanosta-7,9(11)-dienol	11.975	498^*^,442,426	+	-	-	-	-	-	-
Cholesterol	12.008	368,329,129^*^	-	-	+	-	+	-	-
Ergosta-5,7,24(28)-trienol	12.550	468,363^*^,337	-	-	-	-	+	-	-
Ergosterol	12.875	378,363^*^,337	+	+	+	+	+	+	+
Ergosta-7,22-dienol	13.042	470,343^*^,255	-	+	+	+	+	+	-
Ergosta-5,7-dienol	13.492	380,365^*^,339	-	+	-	-	-	-	-
ergost-4,7,22-trienol	13.717	482,392,377^*^	-	+	-	+	-	-	-
Lanosterol	13.908	498,393^*^,241	+	+	+	+	+	+	+
4α-methylergosta-7,24(28)-dienol	14.517	497,407^*^,207	+	+	+	+	+	+	+

*Rt* retention time; *: Base peak; ‘ + ’: detected; ‘ - ’: not detected

### Ergosterol and lanosterol contents

**Fig. 8:**
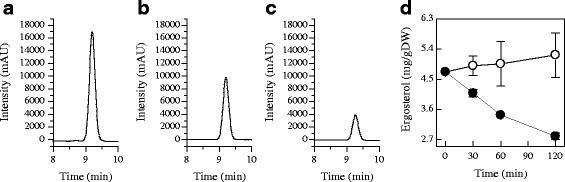
HPLC analysis of ergosterol in control and 1/2MIC citral treated samples. **a**: The ergosterol standard (with a retention time of 9.376 min); **b**: The ergosterol in control samples (with a retention time of 9.205 min); **c**: The ergosterol in 1/2MIC citral treated samples (with a retention time of 9.269 min); **d**: Total ergosterol contents in control (○) and 1/2MIC citral treated (●) *P. digitatum* cells, and values are the mean ± SD of three measurements

To confirm the results of GC-MS analysis and to illustrate the effect of citral on ergosterol biosynthesis further, we determined the lanosterol and ergosterol contents of *P. digitatum* by HPLC-UV. The chromatograms for the ergosterol standard, the ergosterol in control and 1/2MIC citral treated samples were presented in Fig. 8a, b and c, respectively. The ergosterol contents of *P. digitatum* cells treated with citral continuously decreased throughout the entire period, whereas those in the untreated cells remained stable (Fig. 8d). The ergosterol content of *P. digitatum* cells incubated with 1/2MIC of citral for 30 min was 4.08 ± 0.11 mg/g DW, which was significantly lower than that of the control (4.90 ± 0.29 mg/g DW). This difference became more evident with the increasing of exposure time. The ergosterol contents in treatment cells was 2.80 ± 0.09 mg/g DW at 120 min of exposure, as compared to 5.22 ± 0.66 mg/g DW in control cells.

**Fig. 9:**
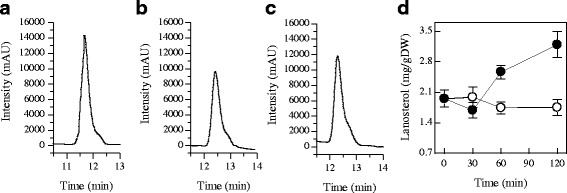
HPLC analysis of lanosterol in control and 1/2MIC citral treated samples. **a**: The lanosterol standard (with a retention time of 11.669 min); **b**: The lanosterol in control samples (with a retention time of 12.426 min); **c**: The lanosterol in 1/2MIC citral treated samples (with a retention time of 12.288 min); **d**: Total lanosterol contents in control (○) and 1/2MIC citral treated (●) *P. digitatum* cells, and values are the mean ± SD of three measurements

The chromatograms for the lanosterol standard, the lanosterol in control and 1/2MIC citral treated samples were presented in Fig. 9a, b and c, respectively. The lanosterol content in control samples did not alter significantly during the entire period (Fig. 9d). Likely, the lanosterol content in citral-treated samples did not alter significantly at the initial 30 min (Fig. 9d). After 60 min of exposure, the lanosterol exclusively accumulated in citral-treated samples, whose content reached 2.56 ± 0.15 mg/g DW and was significantly higher than that of the control (1.74 ± 0.14 mg/g DW). This result, together with the decreased ergosterol content in citral-treated samples, indicates that the ergosterol biosynthesis pathway in *P. digitatum* was partially blocked by citral exposure. This blocking was commonly caused by the antifungal substances used in controlling postharvest diseases [53, 54].

The ergosterol and lanosterol contents in 1/2MIC citral-treated *P. digitatum* cells correspond well with the transcriptional regulation of *ERG7, ERG11*, *ERG6*, *ERG3*, and *ERG5* genes. After the initial addition of citral, the gene expressions of *ERG11*, *ERG6*, *ERG3*, and *ERG5* were partially inhibited and these led to the decrease in total ergosterol content before 30 min of exposure. Correspondingly, the absence of ergosterol might induce the gene expression of *ERG7* at this time, as convinced by the up-regulation of this gene and the accumulation of lanosterol content. The gene expressions of *ERG7* in both groups have the same changing tendency after 60 min of exposure, but the expression levels in 1/2MIC citral-treated samples were significantly higher (*P* < 0.05) than those in control group. This up regulation, together with the reduction of ergosterol content, might stimulate the gene expressions of *ERG6*, *ERG3* and *ERG5* to provide more ergosterol to meet the demand of cells, which is thought to be a general response to decreased ergosterol levels [54]. This regulatory manner is similar to a previous finding that described in *S. cerevisiae* cells exposed to *α*-terpinene [13]. Nevertheless, the gene expressions of *ERG6* and *ERG3* in 1/2MIC group were still lower than the control groups, which were supposed to contribute a lot to the inhibition of ergosterol biosynthesis, as illustrated by other documents [41, 55–57]. The gene expression of *ERG5* in 1/2MIC group was lower than that in the CK group at 120 min of treatment, which might be caused by the reduction in its precursor (ergosta-5,7,24(28)-trienol). Interestingly, the expression levels of *ERG11* continuously declined in 1/2MIC citral-treated groups and were always lower than those of CK group. Considering that the down-regulation of *ERG11* can block the ergosterol biosynthesis and lead to the decrease of ergosterol content to inhibit the growth of fungi [43, 47], our present finding implies that *ERG11* is a critical target of citral against *P. digitatum*.

## Conclusions

In conclusion, our present research revealed that the global gene expressions involved in many important pathways in *P. digitatum* hyphae, including ABC transport, steroid biosynthesis, amino sugar and nucleotide sugar metabolism, starch and sucrose metabolism, TCA cycle, oxidative phosphorylation, RNA degradation, and ribosome, can be greatly affected by citral exposure. RTFQ-PCR verified that citral can affect the expression level of five genes (*ERG7*, *ERG11*, *ERG6*, *ERG3* and *ERG5*) in ergosterol biosynthetic pathway of *P. digitatum*, showing that *ERG11* is a critical targeted gene. In addition, the ergosterol and lanosterol contents in citral-treated samples correspond well with the coordinated regulation of these five genes and GC-MS results. These results suggest that citral could exhibit its antifungal activity against the mycelial growth of *P. digitatum* by disrupting ergosterol biosynthesis.

## Abbreviations

MIC, minimum inhibitory concentration; PDA, potato dextrose agar; PDB, potato dextrose broth; DEGs, differentially expressed genes; FPKM, fragments per Kb million fragments; FDR, false discovery rate; RTFQ-PCR, real-time fluorescence quantitative PCR; RIN, RNA integrated number; CDS, coding sequence; NR, non-redundant protein; NT, non-redundant nucleotide sequence; KEGG, Kyoto Encyclopedia of Genes and Genomes; COG, clusters of orthologous groups of proteins; GO, gene ontology; NCBI, National Center for Biotechnology Information; GC-MS, gas chromatography-mass spectrometry; HPLC, high-performance liquid chromatography; MSTFA, N-methyl-N-trimethylsilyltrifluoroacetamide; TSIM, N-trimethylsilylimidazole; MRD, multi-drug resistance; MRP, Multi-drug resistance-associated protein; PDR, Pleiotropic drug resistance; 1/2MIC, Half of minimum inhibitory concentration

## Additional files

Additional file 1: Table S1. Annotation of all *Penicillium digitatum* unigenes. (XLS 11070 kb) Additional file 2: Table S2. The sequencing data of all *Penicillium digitatum* CDS mapped to the protein database or predicted. (DOC 17505 kb) Additional file 3: Table S3. The differentially expressed genes in untreated *Penicillium digitatum* (CK30) and citral-treated *P. digitatum* (T30) (*P* < 0.05). (XLS 4848 kb)
