# Supplementary material for: Transcriptional profiling analysis of Penicillium digitatum, the causal agent of citrus green mold, unravels an inhibited ergosterol biosynthesis pathway in response to citral
Source: BMC Genomics. 2016 Aug 11;17:599. doi: 10.1186/s12864-016-2943-4 (PMC4982135; doi:10.1186/s12864-016-2943-4)
Supplement: Additional file 2: Table S2. — The sequencing data of all Penicillium digitatum CDS mapped to the protein database or predicted. (DOC 17505 kb) [file 12864_2016_2943_MOESM2_ESM.doc]

**Additional file 2: Table S2.** The genetic/sequencing data of CDS which mapped to the protein database.

>CL1.Contig1_All 3 1637 minus strand FKS1 [Penicillium digitatum] >gi|425768938|gb|EKV07449.1| 1,3-beta-glucan synthase catalytic subunit FksP [Penicillium digitatum PHI26] >gi|425776228|gb|EKV14454.1| 1,3-beta-glucan synthase catalytic subunit FksP [Penicillium digitatum Pd1]

CTCTGCGTCAACCTGATCCCTATTATCAACTGGGTTAACCGCTGTGTCATCTCCATCTTC

ATTGTTTTTTGGATCTCATTCGTTCCACTAGCGGTCCAAGAATTGACAGAGAGAGGAGTG

TGGCGTATGGCCACTCGTTTGGCCAAGCACTTTGGTTCGTTCTCGTTCATGTTTGAGGTC

TTCGTTTGTCAGATATACTCCAATGCCGTCCACCAGAACTTGTCTTTCGGAGGCGCGCGA

TACATTGGTACCGGCCGTGGTTTCGCAACAGCTCGTATTCCATTCGGTGTCCTCTACTCT

CGATTTGCTAGCCCTTCAATCTACTTGGGTGCTCGCCTCCTGCTGATGCTATTGTTCAGT

ACCACTACTGTCTGGACTCCTGCTCTGATCTGGTTCTGGGTTTCTCTGCTCGCACTATCC

ATCTCGCCTTTCCTATTCAACCCCCACCAATTTTCCTGGAATGACTTCTTCATTGATTAC

CGTGACTACATCCGCTGGCTTTCTCGTGGCAACTCCCGATCCCATGCTTCCTCTTGGATT

GGTTTCTGTCGCCTTTCTCGTACCCGGACTACTGGTTACAAGCGAAAACTTATTGGTGTT

CCATCGGAGAAAGGCTGCGGTGATATTCCAAAAGCGCGTCTAAGTAACATCTTTTTCAGC

GAAATCGTTGGGCCTCTGATCGGTGTTGCCGTTACCCTGATTCCGTATCTGTACATCAAC

TCCCGCACAATGTACTCCGATGATAACAAATGGGCTGCGAATCCCATCCTTCGCGTTGCG

ATTGTGGGTCTTGCCCCTGTGGGTGTCAATGCCGGTGTTGCCGGCATGTTCTTCGGCCTG

GCTTGTTGTATGGGCCCGGTATTTGGTATGTGCTGCAAGAAGTTCGGCGCTGTTCTCGCT

GCCATCGCCCATGCCATTGCTGTGATTGTTTTCCTCCTTCTATTCCTGGCCATGTTCTTC

CTTGAATCTCTCAGCTGGGCTAGAACTATCTCCGGAATGATCGCTGCGATGACTCTTCAG

CGCTTCATCTACAAGCTTATCATTTCCTTAGCCCTGACGCGTGAATTCAAGAACGACCAG

TCTAACATTGCCTGGTGGACCGGAAAATGGTACAATATGGGTTGGCACTCCTTCTCTCAG

CCTGGACGTGAATTCCTGTGCAAGATCACTGAACTTGGTTATTTCGCCACTGATTTCTTC

CTGGGCCACATTCTCCTGTTTGCTATGCTTCCGCTCCTTGCTATTCCCTACATTGACACC

TTCCATTCCGTCCTCCTGTTCTGGTTGCGACCAAGCTCTCAAATCCGCCCTCCCATCTAT

TCCCTCAAGCAGTCCAAGCTTCGTAAGAGACGTGTTGTCCGCTTTGCCATTCTCTACTTT

ACCTTGCTACTTATCTTTGTCGTCATTATTGCTGCTCCCGTTGTCCTGCGGAACACAAAT

CAACTGGACAGCATCCTGGAGGGTGGCATCTGGAAGAGCCTCGGTGTCGCCAGCACAACA

GGAATCGGACTCCTGCAGCCACTTGATCGTGGACTTAATGACACTGTCTCCTGGTATACC

GGCTCTAACATCCCCAAAGGCTACACCTCAGGCACAATCCCGGCAGCCTTTGAGGGCACT

GGAGTTTGGTCCATC

>CL1.Contig2_All 2 247 Pc22g04100 [Penicillium chrysogenum Wisconsin 54-1255] >gi|211591466|emb|CAP97698.1| Pc22g04100 [Penicillium chrysogenum Wisconsin 54-1255]

CAGTCCAAACTTCGCAAGAGACGCGTTGTCCGCTTCGCCATTCTCTACTTTGTCATGCTT

GTCATCTTCGTCGTTATTATCGCTGCGCCTGTCGTCATGCGGAACACGGGGCAATTGGAT

AGCGTCCTGAAGGGCGACCTGTGGAAGAGCCTCGGTGTATCCAAGACCAACGCAGTTGGC

CTCCTGCAGCCACTGGATCGCGGACTCAATGATACCGTCTCCTGGTACACCGGATCTAAC

ATCCCG

>CL2.Contig1_All 294 2315 minus strand hypothetical protein PDIP_21860 [Penicillium digitatum Pd1]

ATGTCTCATGCTCACCATGCCGGCTTGGTGATGCGAAACCACGTTCGTCGGGATGTCATC

CCTGCGAATCGAGTGCCGATCTTTGTGCCCAGTCTCTCTGTAGCCACACAGCTTCCAACC

CTTGTGGCAAGATCCGAATCTGAGCCGACCTCGGGACCCAAGGCCACCAGCAATCTGGCA

ACGACCGTCTTTCCCATCGTGTTCGGTGCAGGAATTCCCATTTTTTGTGCATTAATCATC

CTCGTTGTGCTTCACCGGAGACAAGTCAAGAAACTTGTGCGGGAAGATGCTATGGATAAA

CACAAGTCTCTTGATTTTGGACTGGATACAGTGGGGCCTGCAACTAGGAGAAAAGGCGCC

AAGGGAATGCCCCCGATGTCAGAACACAACCATACCAAAGGATTGTCTCTTGATGTCGGA

CCATACCTACTGCCTCCGGGCCTGCAACATTCCACCGACTCTCTGCGCTCCATGTCAATA

GATGATGGCAAGTACCGTCCTGCCACTGCCTCCATTCGGTCGAACTCCAGGAATTCCAAA

TATGGAGGTACGGATGATGGCAATTCAGGTCTTCTACAGAATGCACAGAGGATTCCTCGT

TCATCCCCGCCGCTATGCTCTCCTATCGAACCTCGTGCACGCAGCCCTCTGAACCAACAC

GATGACTACATTGGGCAAGTTCCCGAAGTGACACATCCACCTGCCGTACACCAACCCGGC

ATGGCTATTGGAAGTCCCAATACCAACCGGATTCCCTCGCCAGAGCCTCTGCCCCACGTG

GACTCCTCTTCGGGCCTAAACTTCAGATTGGACGATACAGACGACAACACACATAACAAC

CAGCATGAAGAGCGCCCCAACTTCCCGCTGCCTGAGCCTTCGCAACCACGAGACTCGAAC

CTTGACCAGGCTCCTGCATCACAACTTCCTCGTATCTCCCTGCCGGTCAGCATTGTTACT

ACTAGTGACTATGGCGATGACCGCAAGTCGCAGCAAATTCTGCCTGCGGTCAACATTTAT

GGCACAGAAGGTGCACAGCAACGTGATGCTCCAGCCCACGATAACAAGCACCCAGCACTC

CGAGAGGAATCTAAGAATCTGGATGCGGCATATGACAACCGCCGCGATACCCGCCGGATG

ACTCTCGGGTTGCGCCCTTTGCCGCCAGAGGACCCCAGCGACAACCCAGAACAGCGTGCC

AATCGGATTCGCTCCTTCTACAAGGAGTACTTCGATGAGAACAAAACGGGCAGCCAGGAG

CCCACATACCACGAGGACTTCGGCCCTGAGTCCCGTGAGGGTGGTCGTAATTCGGCTGGT

TTCATCTACGACCCAACCACCGGCGACTATTTTGATGCACGTCATGGTGTTGCTCCATTC

GCTGAGCCGATAACTCGTCGCGCAATGACACCGCCGCCTCGCGCACCTCCACGCTTCCAG

GGAGCCGCTCGCCACATGGCCACCAACTCTGCTGGCAACAATGGTCTTCCCGGCGCACGC

GCCTTCTCACCTGCCTCCGGCCATATGCCTGGTCCACGCGGTCCCAAGAAGCCCATCCCC

CGCCCCCCGCCGCTGCAAATCCTTCCTACTCCTCACATGTTGACGGATGACTCTCTCATG

AGCGCCATGGAATTTGCCCCGGGAAATGGAATCAAGGACCGCCGCGCCGGTCGCCCTGGG

ACACCCACCGGTGGTGTGCGCCCGTACATCCCTGGCGCGCGTGTCCACACGCCATTGGCC

TCTGCCTTTGATGAGCTCGCCGTGATGCCCAGTGCCCACGCCCTGCGCAAGTCTGGCACG

TTCAACAACCTGGACTTTGTGCCCCCGCCACGGTTCAAGAACGAAGGTGGTGTCGGCAGC

GATGCGGGTAGTATCCGCAGTGGCCGGACTGCCGTCTCTGCCAACCACCAAAATAATATC

CGCATGGGCAATTACCGTGTCAGTCGTCTCCCAGCTGAAGCGGTCGGTACCAAGGACGAC

ATGATGGCCAGTCTGCGTCCGAAGTGGGGTATGAACAACGGA

>CL3.Contig1_All 56 310 minus strand Serine/threonine protein kinase, putative [Penicillium digitatum PHI26] >gi|425776369|gb|EKV14588.1| Serine/threonine protein kinase, putative [Penicillium digitatum Pd1]

ATGGATCCCAACAACAACACTCGAAACAACTTGAGTTACGGCTATGACCGTAACTACAAT

TCCAGCAGTCGAGCCTATCCCACTACCCCCTCTGCCTTCCCCCAGCCCATCTATCAGACT

CAGGGGGCCCAGGAACACGTCGATCCCTCCAATCCAGCTTACGCTCCGGGCTACTTTATG

CCCAACAACTACCCTCCCAACCAGGTGCAGCCGCAGTTTGCCCAGCAACATCAGCAGCAG

CAGCAGCAGCAGTAT

>CL3.Contig2_All 56 1981 minus strand Serine/threonine protein kinase, putative [Penicillium digitatum PHI26] >gi|425776369|gb|EKV14588.1| Serine/threonine protein kinase, putative [Penicillium digitatum Pd1]

ATGGATCCCAACAACAACACTCGAAACAACTTGAGTTACGGCTATGACCGTAACTACAAT

TCCAGCAGTCGAGCCTATCCCACTACCCCCTCTGCCTTCCCCCAGCCCATCTATCAGACT

CAGGGGGCCCAGGAACACGTCGATCCCTCCAATCCAGCTTACGCTCCGGGCTACTTTATG

CCCAACAACTACCCTCCCAACCAGGTGCAGCCGCAGTTTGCCCAGCAACATCAGCAGCAG

CAGCAGCAGCAGTATGCGCAGCAGCAAGCATTGCAATCGCCCCAGCCCGCCTACCAAGCA

CGCCAGGGCTACCCCGATGGCACCAACAGTCTGATCCAACAATTCTCCAACCAAGATCTC

AACGCAAACCGTGCAGGGTTTTTCAATAATCGAACTGGCTCCCCGGCCCAGCGGCCTCGC

ACCGCCGGCGGCTCCCCCGCTCAGTCACAAGCCCAAGCATCACACCTTATCCCCCCCGTG

CCCCGCAGCCCTCGTCCGCCCCCCGAGAATGAAGAGTTGCAGCGCTTCCCCGACCGATAC

TCCGAAAATGCGCACAAGCGAGGCAAGGCTGCAAAGGAGCTGGTGACTGTGTTCTTCCAC

GAGAATATCGAACGGGCCCGTGACCGTAACATGCGATCAGCGGAACTTGACAAAACTATC

CGTGAACCCAACATCTCCACCGAAAAGAAGCTCCAGGATGCCAACTTGATTTCTAAAAGA

GAGTCTGATTTCCTTCGGTTCTTGCGCACAAAGGAGACACCGGCAAATTTCCAGACCATC

AAAATTGTTGGAAAGGGTGCCTTCGGTGAGGTCAAGCTCGTGCAGCGCAAGACTGATGGC

AAGGTTTACGCCCTCAAGTCGTTGATCAAGACCGAAATGTTCAAGAAGGACCAATTAGCC

CACGTCCGTGCCGAGCGTGATATTCTGGCGGATTCGAAGGACAACCCTTGGCTTGTGAAG

CTTCACGCGTCGTTCCAGGACGCTGCGTACCTTTACTTGTTGATGGAGTTTTTGCCCGGT

GGTGATTTGATGACCATGTTGATCAAGTACGAGATCTTCTCGGAGGATATTACACGGTTC

TACATGGCTGAGATCGTGATGGCAATTGAAGCCGTACACAAGCTTGGTTTCCTCCATCGT

GATATCAAACCTGACAACATCTTGCTTGACCGCGGTGGACACGTCAAGCTTACTGACTTC

GGTCTTTCAACCGGAGGCAAGAAGACCCATGACAATGCTTACTACCAAAACCTTCTCAAG

AACTCCACTTCCAAAGATAGGAACCGCAACTCTGGATACTTCAATGATGCCATTAATCTG

ACAGTGTCCAATCGTGGTCAAATCAACACCTGGCGAAAGTCTCGTCGTGCCATGGCCTAT

TCGACTGTCGGTACTCCTGATTATATTGCTCCTGAGATTTTCAACGGTCAAGGCTACACT

TATCTTTGCGATTGGTGGTCGGTGGGTGCTATCATGTTTGAATGTCTTGTGGGCTGGCCT

CCATTCTGTGCTGAAGACACAAATGACACCTACCGGAAAATCGTTAACTGGAGAGAGTGC

TTGTACTTCCCCGAGGAACTGGTTCTTGGGCGTGACTCTGAGGCACTGATCCGAAGTTTC

TTGTGTGACCCAGATCACCGAATTGGCAGCGAAGGTGGTCAGCACGGAGGCGCGACACAA

ATCAAAAACCACCCCTTCTTCAGGGGTGTTGTTTGGGACCAACTGCGCAGCATCCGTGCT

CCCTTCGAGCCGCGCCTCAGCTCGAACATCGATGTGTCGTACTTCCCGATTGATGAAATT

CCTCAGGAGGATACCAGCGCGATCCACCGTGCCCAGGCTCGTGCCATGCCCGACTCACAG

GAAGCCGAGATGAGTTTGCCGTTCATCGGCTACACCTATAAGGCCTTCAATGCCTTCCAA

ACCAAC

>CL3.Contig3_All 512 2206 minus strand Serine/threonine protein kinase, putative [Penicillium digitatum PHI26] >gi|425776369|gb|EKV14588.1| Serine/threonine protein kinase, putative [Penicillium digitatum Pd1]

CAGCAGCAGCAGCAGCAGCAGTATGCGCAGCAGCAAGCATTGCAATCGCCCCAGCCCGCC

TACCAAGCACGCCAGGGCTACCCCGATGGCACCAACAGTCTGATCCAACAATTCTCCAAC

CAAGATCTCAACGCAAACCGTGCAGGGTTTTTCAATAATCGAACTGGCTCCCCGGCCCAG

CGGCCTCGCACCGCCGGCGGCTCCCCCGCTCAGTCACAAGCCCAAGCATCACACCTTATC

CCCCCCGTGCCCCGCAGCCCTCGTCCGCCCCCCGAGAATGAAGAGTTGCAGCGCTTCCCC

GACCGATACTCCGAAAATGCGCACAAGCGAGGCAAGGCTGCAAAGGAGCTGGTGACTGTG

TTCTTCCACGAGAATATCGAACGGGCCCGTGACCGTAACATGCGATCAGCGGAACTTGAC

AAAACTATCCGTGAACCCAACATCTCCACCGAAAAGAAGCTCCAGGATGCCAACTTGATT

TCTAAAAGAGAGTCTGATTTCCTTCGGTTCTTGCGCACAAAGGAGACACCGGCAAATTTC

CAGACCATCAAAATTGTTGGAAAGGGTGCCTTCGGTGAGGTCAAGCTCGTGCAGCGCAAG

ACTGATGGCAAGGTTTACGCCCTCAAGTCGTTGATCAAGACCGAAATGTTCAAGAAGGAC

CAATTAGCCCACGTCCGTGCCGAGCGTGATATTCTGGCGGATTCGAAGGACAACCCTTGG

CTTGTGAAGCTTCACGCGTCGTTCCAGGACGCTGCGTACCTTTACTTGTTGATGGAGTTT

TTGCCCGGTGGTGATTTGATGACCATGTTGATCAAGTACGAGATCTTCTCGGAGGATATT

ACACGGTTCTACATGGCTGAGATCGTGATGGCAATTGAAGCCGTACACAAGCTTGGTTTC

CTCCATCGTGATATCAAACCTGACAACATCTTGCTTGACCGCGGTGGACACGTCAAGCTT

ACTGACTTCGGTCTTTCAACCGGAGGCAAGAAGACCCATGACAATGCTTACTACCAAAAC

CTTCTCAAGAACTCCACTTCCAAAGATAGGAACCGCAACTCTGGATACTTCAATGATGCC

ATTAATCTGACAGTGTCCAATCGTGGTCAAATCAACACCTGGCGAAAGTCTCGTCGTGCC

ATGGCCTATTCGACTGTCGGTACTCCTGATTATATTGCTCCTGAGATTTTCAACGGTCAA

GGCTACACTTATCTTTGCGATTGGTGGTCGGTGGGTGCTATCATGTTTGAATGTCTTGTG

GGCTGGCCTCCATTCTGTGCTGAAGACACAAATGACACCTACCGGAAAATCGTTAACTGG

AGAGAGTGCTTGTACTTCCCCGAGGAACTGGTTCTTGGGCGTGACTCTGAGGCACTGATC

CGAAGTTTCTTGTGTGACCCAGATCACCGAATTGGCAGCGAAGGTGGTCAGCACGGAGGC

GCGACACAAATCAAAAACCACCCCTTCTTCAGGGGTGTTGTTTGGGACCAACTGCGCAGC

ATCCGTGCTCCCTTCGAGCCGCGCCTCAGCTCGAACATCGATGTGTCGTACTTCCCGATT

GATGAAATTCCTCAGGAGGATACCAGCGCGATCCACCGTGCCCAGGCTCGTGCCATGCCC

GACTCACAGGAAGCCGAGATGAGTTTGCCGTTCATCGGCTACACCTATAAGGCCTTCAAT

GCCTTCCAAACCAAC

>CL4.Contig1_All 267 548 minus strand Pre-rRNA-processing protein ipi1 [Penicillium digitatum PHI26]

ATGTCTAACACGGACGAAGCCTCGGAGCCATTCACTACCAAAACGAATACCACAGATAGA

GTCTCCGGCCGCGTGCATGAGTCTGCCAGTCATGCACGCGAGAAGATATCCGAACAGCTC

CAACCCGGAGATATGAAGGCGACATCTCGTCGCTCGTCGGATACACCCGATAATATCTGG

CAATTTGGACAAGGTGGTGCAAGAGAAGAGCGAGATTATGGCCTGGAGAGGGATAAGTCT

ATTCTACAGTCGGTGCAGGAGAAGGTGGCTAAGGCCCTCGGG

>CL4.Contig2_All 321 608 minus strand Pre-rRNA-processing protein ipi1 [Penicillium digitatum PHI26]

ATGTCTAACACGGACGAAGCCTCGGAGCCATTCACTACCAAAACGAATACCACAGATAGA

GTCTCCGGCCGCGTGCATGAGTCTGCCAGTCATGCACGCGAGAAGATATCCGAACAGCTC

CAACCCGGAGATATGAAGGCGACATCTCGTCGCTCGTCGGATACACCCGATAATATCTGG

CAATTTGGACAAGGTGGTGCAAGAGAAGAGCGAGATTATGGCCTGGAGAGGGATAAGTCT

ATTCTACAGTCGGTGCAGGAGAAGGTGGCTAAGGCCCTCGGGGGAGGG

>CL4.Contig3_All 321 449 minus strand Pre-rRNA-processing protein ipi1 [Penicillium digitatum PHI26]

ATGTCTAACACGGACGAAGCCTCGGAGCCATTCACTACCAAAACGAATACCACAGATAGA

GTCTCCGGCCGCGTGCATGAGTCTGCCAGTCATGCACGCGAGAAGATATCCGAACAGCTC

CAACCCGGT

>CL4.Contig4_All 267 395 minus strand Pre-rRNA-processing protein ipi1 [Penicillium digitatum PHI26]

ATGTCTAACACGGACGAAGCCTCGGAGCCATTCACTACCAAAACGAATACCACAGATAGA

GTCTCCGGCCGCGTGCATGAGTCTGCCAGTCATGCACGCGAGAAGATATCCGAACAGCTC

CAACCCGGT

>CL5.Contig1_All 2 238 Aminopeptidase, putative [Penicillium digitatum PHI26] >gi|425780730|gb|EKV18731.1| Aminopeptidase, putative [Penicillium digitatum Pd1]

TTCAAGGAAGAACAAAATTATCTTGTTTGGTCCCAAGTCTCTGCGTCCCTGGCGAACCTC

CGGTCGGTTTTCTCGCAAAACGAGAAAGTGGCCGAGGGATTGAAGCAGTTTACCCTTAAA

CTGGTTTCCCCGGCCGCAGAACGTATTGGGTGGGAGTTCAAGTCCGATGAGGACTACCTC

ATTGTGCAGCTTCGAAAGCTGCTCATTGCAATGGCCTGTAATGCGGGTCATGAAGGG

>CL5.Contig2_All 258 2195 Aminopeptidase, putative [Penicillium digitatum PHI26] >gi|425780730|gb|EKV18731.1| Aminopeptidase, putative [Penicillium digitatum Pd1]

ATGGCATCTCAAGAGCGAGAGACACTTCCTGACGTGGCTAAGCCTATCAACTATCACGTC

TCTCTTTTCGACTTGCAGTTCGGTGGTTCGTGGGAATACAAGGGCGCGTTGCAGATCGAC

CTCAAAGTCACACGTGCTACCAGGGAGATTGTTCTGAACTCGAAGGAGATCGAAGTACAA

AATGCCGAGATTCTGGGCAAAGATGGGAGTCAATTAGCCAAAGCATCCGGAATCACCTAC

GACAAGCAATCGGAGCGAGTCTCCCTCGCTTTCTCGCAAGAGATTGCGCCGGCAAACGTT

GTGCTTTCTATCAATTTTACCGGCATTATGAACAATGCGATGGCCGGTTTCTACCGCTCG

AAGTATAAGCCCATTGGAGAACCATCTCCTGACACACCCAAAGAGGGTGACTTTCACTAC

ATGCTAAGCACTCAATTTGAGTCCTGTGACGCACGCAGGGCTTTCCCGTGTTTCGATGAG

CCAAACTTGAAATCCACCTTCGATTTCGAGATTGAAGTTCCCAAGGGACAGACTGCATTG

AGTAACATGCCCGTTCAATCTGAAAGAGATGGAAACAAGCCCGGTCTGAAGTTTGTCACG

TTCGAGAAGACACCGGTGATGAGCACCTATCTCCTGGCCTGGGCAGTTGGTGACTTCGAA

TACGTCGAGGCTATGACTGAGCGCAAATATCAGGGCAAGAGTATTCCCGTTCGAGTCTAC

ACCACTCGGGGCCTTCAAGACCAGGCTCGTTTTGCACTGGAATGTGCTCACCGCACGGTT

GACTACTTCTCGGAAGTCTTCGAAATTGAATACCCTCTCCCCAAGGCTGATCTCCTAGCT

GTTCATGAATTCGCCATGGGTGCCATGGAGAACTGGGGTCTTGTGACCTATCGAACGACC

GCCGTTCTCTTCGATGAAGGAAAATCGGACAACCGCTATAAGAACCGCATTGCTTATGTT

GTAGCTCACGAGCTGGCTCACCAGTGGTTTGGCAATCTCGTCACCATGGACTGGTGGAAT

GAGTTGTGGCTGAATGAGGGATTCGCAACCTGGGTCGGCTGGCTTGCCGTTGATCATTTC

TATCCTGAGTGGAACGTCTGGTCTCAGTTTGTTGCTGAAGGTGTGCAACAAGCATTTCAC

CTCGACTCGCTCCGCGCTTCCCACCCCATCGAAGTTCCCGTTCGGAACGCCCTCGAAGTT

GACCAAATTTTCGACCATATCAGCTATCTGAAGGGAAGCTCCGTCATTCGCATGCTGAGT

GTTCACCTCGGTAGAGAGACTTTCTTGCGCGGAGTTGCAGACTATCTCAAATCTCATGCA

TATGGTAAGGTGTCAAAAAAGAGGATTCTGAGGAACGTGTGCTGAGTGGTTATAGGAAAT

GCGACCACAAATGACCTGTGGTCTGCCTTGAGCAAAGCCTCCGGCCAGGACGTGCATTCT

TTTATGGTTAGTAAATACCCAATCCTTGGCTCTCTGTTTGACTCTGTTTGGTCAACTCGA

GATCTAACTCGTCTGCCCTAGGATCCCTGGATTCGCAAGATTGGTTTCCCGGTTGTCACC

GTGACAGAAGAGCCCGGTCAGGTCACCGTTAGCCAGAATCGATTCTTGTCTACCGGTGAT

GCGAAGCCAGAGGAGAATGAGACGAAATGGTGGATTCCTCTCGGCATCAAATCCGGACCG

AAACTGGCTACTGTTGACACCCGTGCTCTGACTTCGAAATCCGACACCGTTGGAGGAATT

GGGGAGGACTCTTTTTACAAGATCAACAAGGACCTGTCTGGGTTCTATCGAACCAACTAT

CCTCCTATGCATCTGGCAAAGCTGGGCCAGTCGCTTAATCTCCTGAGCACTGAGGACAAG

ATCGGCTTGCTGGGTGATGCAGCTGCCCTTGCAGTCTCTGGCGAAGGCACTACGCCCGCC

TTGTTGAATCTTCTGGAA

>CL5.Contig3_All 258 2900 Aminopeptidase, putative [Penicillium digitatum PHI26] >gi|425780730|gb|EKV18731.1| Aminopeptidase, putative [Penicillium digitatum Pd1]

ATGGCATCTCAAGAGCGAGAGACACTTCCTGACGTGGCTAAGCCTATCAACTATCACGTC

TCTCTTTTCGACTTGCAGTTCGGTGGTTCGTGGGAATACAAGGGCGCGTTGCAGATCGAC

CTCAAAGTCACACGTGCTACCAGGGAGATTGTTCTGAACTCGAAGGAGATCGAAGTACAA

AATGCCGAGATTCTGGGCAAAGATGGGAGTCAATTAGCCAAAGCATCCGGAATCACCTAC

GACAAGCAATCGGAGCGAGTCTCCCTCGCTTTCTCGCAAGAGATTGCGCCGGCAAACGTT

GTGCTTTCTATCAATTTTACCGGCATTATGAACAATGCGATGGCCGGTTTCTACCGCTCG

AAGTATAAGCCCATTGGAGAACCATCTCCTGACACACCCAAAGAGGGTGACTTTCACTAC

ATGCTAAGCACTCAATTTGAGTCCTGTGACGCACGCAGGGCTTTCCCGTGTTTCGATGAG

CCAAACTTGAAATCCACCTTCGATTTCGAGATTGAAGTTCCCAAGGGACAGACTGCATTG

AGTAACATGCCCGTTCAATCTGAAAGAGATGGAAACAAGCCCGGTCTGAAGTTTGTCACG

TTCGAGAAGACACCGGTGATGAGCACCTATCTCCTGGCCTGGGCAGTTGGTGACTTCGAA

TACGTCGAGGCTATGACTGAGCGCAAATATCAGGGCAAGAGTATTCCCGTTCGAGTCTAC

ACCACTCGGGGCCTTCAAGACCAGGCTCGTTTTGCACTGGAATGTGCTCACCGCACGGTT

GACTACTTCTCGGAAGTCTTCGAAATTGAATACCCTCTCCCCAAGGCTGATCTCCTAGCT

GTTCATGAATTCGCCATGGGTGCCATGGAGAACTGGGGTCTTGTGACCTATCGAACGACC

GCCGTTCTCTTCGATGAAGGAAAATCGGACAACCGCTATAAGAACCGCATTGCTTATGTT

GTAGCTCACGAGCTGGCTCACCAGTGGTTTGGCAATCTCGTCACCATGGACTGGTGGAAT

GAGTTGTGGCTGAATGAGGGATTCGCAACCTGGGTCGGCTGGCTTGCCGTTGATCATTTC

TATCCTGAGTGGAACGTCTGGTCTCAGTTTGTTGCTGAAGGTGTGCAACAAGCATTTCAC

CTCGACTCGCTCCGCGCTTCCCACCCCATCGAAGTTCCCGTTCGGAACGCCCTCGAAGTT

GACCAAATTTTCGACCATATCAGCTATCTGAAGGGAAGCTCCGTCATTCGCATGCTGAGT

GTTCACCTCGGTAGAGAGACTTTCTTGCGCGGAGTTGCAGACTATCTCAAATCTCATGCA

TATGGAAATGCGACCACAAATGACCTGTGGTCTGCCTTGAGCAAAGCCTCCGGCCAGGAC

GTGCATTCTTTTATGGATCCCTGGATTCGCAAGATTGGTTTCCCGGTTGTCACCGTGACA

GAAGAGCCCGGTCAGGTCACCGTTAGCCAGAATCGATTCTTGTCTACCGGTGATGCGAAG

CCAGAGGAGAATGAGACGAAATGGTGGATTCCTCTCGGCATCAAATCCGGACCGAAACTG

GCTACTGTTGACACCCGTGCTCTGACTTCGAAATCCGACACCGTTGGAGGAATTGGGGAG

GACTCTTTTTACAAGATCAACAAGGACCTGTCTGGGTTCTATCGAACCAACTATCCTCCT

ATGCATCTGGCAAAGCTGGGCCAGTCGCTTAATCTCCTGAGCACTGAGGACAAGATCGGC

TTGCTGGGTGATGCAGCTGCCCTTGCAGTCTCTGGCGAAGGCACTACGCCCGCCTTGTTG

AATCTTCTGGAAGGATTCAAGGAAGAACAAAATTATCTTGTTTGGTCCCAAGTCTCTGCG

TCCCTGGCGAACCTCCGGTCGGTTTTCTCGCAAAACGAGAAAGTGGCCGAGGGATTGAAG

CAGTTTACCCTTAAACTGGTTTCCCCGGCCGCAGAACGTATTGGGTGGGAGTTCAAGTCC

GATGAGGACTACCTCATTGTGCAGCTTCGAAAGCTGCTCATTGCAATGGCCTGTAATGCG

GGTCATGAAGGATTTGTCACGGAAGCCAAGCGCCGCTTTGATCTCTGGGCGACCGAAAAA

GATGCCAGTGCCATTCATACCAACCTGCGGTCAGTGATTTTCAGCGTCAATGTGTCCGAG

GGCGGCCGTAAGGAGTACGATGCCGTTAAGAACGAGTATATCCGAACCGACTCTGTGGAT

GGCAAAGAAATTTGCCTGTCTGCACTTGGACGCACCAAGGATGCTGCTCTGGTGGAGGAC

TACTTGAACTTCGTGTTTTCGGACAAGGTGGCCATCCAGGACATCCACAGCGGTGCCGTA

TCTCTGGCCGGTAATTCGAAGGTCCGGCATTTGCTATGGCAGTACATCAAGGACAACTGG

ACTGCTGTCGAAACACGATTGTCATTTAACAATGTTGTGTTTGAGCGCTTTGTCCGTATG

GGTCTATCAAAGTTTGCCGATCACCAGATCAGTGACGACATTGCATCCTTCTTCAAGGAC

AAGGAAACCGGGGCCTATGACCGTGCACTCGTGATTGTCTCGGACAACATTCGAACCAAT

GCAACCTACAAGGAGCGTGAGGAGGCGTTGGTTCTCGAATGGCTGCAGGCACACGGTTAC

GCG

>CL5.Contig4_All 81 905 Aminopeptidase, putative [Penicillium digitatum PHI26] >gi|425780730|gb|EKV18731.1| Aminopeptidase, putative [Penicillium digitatum Pd1]

GCTGAAGGTGTGCAACAAGCATTTCACCTCGACTCGCTCCGCGCTTCCCACCCCATCGAA

GTTCCCGTTCGGAACGCCCTCGAAGTTGACCAAATTTTCGACCATATCAGCTATCTGAAG

GGAAGCTCCGTCATTCGCATGCTGAGTGTTCACCTCGGTAGAGAGACTTTCTTGCGCGGA

GTTGCAGACTATCTCAAATCTCATGCATATGGTAAGGTGTCAAAAAAGAGGATTCTGAGG

AACGTGTGCTGAGTGGTTATAGGAAATGCGACCACAAATGACCTGTGGTCTGCCTTGAGC

AAAGCCTCCGGCCAGGACGTGCATTCTTTTATGGTTAGTAAATACCCAATCCTTGGCTCT

CTGTTTGACTCTGTTTGGTCAACTCGAGATCTAACTCGTCTGCCCTAGGATCCCTGGATT

CGCAAGATTGGTTTCCCGGTTGTCACCGTGACAGAAGAGCCCGGTCAGGTCACCGTTAGC

CAGAATCGATTCTTGTCTACCGGTGATGCGAAGCCAGAGGAGAATGAGACGAAATGGTGG

ATTCCTCTCGGCATCAAATCCGGACCGAAACTGGCTACTGTTGACACCCGTGCTCTGACT

TCGAAATCCGACACCGTTGGAGGAATTGGGGAGGACTCTTTTTACAAGATCAACAAGGAC

CTGTCTGGGTTCTATCGAACCAACTATCCTCCTATGCATCTGGCAAAGCTGGGCCAGTCG

CTTAATCTCCTGAGCACTGAGGACAAGATCGGCTTGCTGGGTGATGCAGCTGCCCTTGCA

GTCTCTGGCGAAGGCACTACGCCCGCCTTGTTGAATCTTCTGGAA

>CL5.Contig5_All 81 779 Aminopeptidase, putative [Penicillium digitatum PHI26] >gi|425780730|gb|EKV18731.1| Aminopeptidase, putative [Penicillium digitatum Pd1]

GCTGAAGGTGTGCAACAAGCATTTCACCTCGACTCGCTCCGCGCTTCCCACCCCATCGAA

GTTCCCGTTCGGAACGCCCTCGAAGTTGACCAAATTTTCGACCATATCAGCTATCTGAAG

GGAAGCTCCGTCATTCGCATGCTGAGTGTTCACCTCGGTAGAGAGACTTTCTTGCGCGGA

GTTGCAGACTATCTCAAATCTCATGCATATGGAAATGCGACCACAAATGACCTGTGGTCT

GCCTTGAGCAAAGCCTCCGGCCAGGACGTGCATTCTTTTATGGATCCCTGGATTCGCAAG

ATTGGTTTCCCGGTTGTCACCGTGACAGAAGAGCCCGGTCAGGTCACCGTTAGCCAGAAT

CGATTCTTGTCTACCGGTGATGCGAAGCCAGAGGAGAATGAGACGAAATGGTGGATTCCT

CTCGGCATCAAATCCGGACCGAAACTGGCTACTGTTGACACCCGTGCTCTGACTTCGAAA

TCCGACACCGTTGGAGGAATTGGGGAGGACTCTTTTTACAAGATCAACAAGGACCTGTCT

GGGTTCTATCGAACCAACTATCCTCCTATGCATCTGGCAAAGCTGGGCCAGTCGCTTAAT

CTCCTGAGCACTGAGGACAAGATCGGCTTGCTGGGTGATGCAGCTGCCCTTGCAGTCTCT

GGCGAAGGCACTACGCCCGCCTTGTTGAATCTTCTGGAA

>CL7.Contig1_All 116 589 minus strand Cytochrome c oxidase subunit Va, putative [Penicillium digitatum Pd1] >gi|425776487|gb|EKV14704.1| Cytochrome c oxidase subunit Va, putative [Penicillium digitatum PHI26]

ATGTCGTCCCTATCTATCTTCCGTGTGGCGACCCGCGCCGTTCGCCCTACCGGCGTCTTT

AGAGCTCCCCAATTGACCCGGTCTCGGATGCAGTCCCCGGTGACTCTGGCTGCCGCCCGC

GCCCACAACTTCGGTACCACCTCTATGCTCCGCTCCGGCCACGAGGATGAGACATTTGAA

GAGTTCACTGCCAGATTCGAGAAGGAATTCGATGGTGTGCAGGACGTTTTCGAGCTCCAG

CGCAACCTGAACAACTGCTTCGCTTACGATCTCGTTCCCTCCGTCGAGGTCCTCACTGCC

GCTCTCAAGGCCGCCCGTCGTGTTAACGACTTCCCTACCGCTGTCCGCGTCTTCGAGGGC

ATCAAGGCCAAGGTCGAGAGCCCTGACCAGTACAAGCAATATCTGGCGTCCCTCGAGGGT

CTCCGCGTCGAGTTGGGTGTCGCTCTGCGGGAGGAGCTCTACCCCGAGGAGGCG

>CL7.Contig2_All 1 195 minus strand Pc21g18840 [Penicillium chrysogenum Wisconsin 54-1255] >gi|211590586|emb|CAP96781.1| Pc21g18840 [Penicillium chrysogenum Wisconsin 54-1255]

TCCGTCGAGGTCCTCACTGCCGCCCTCAAGGCCGCCCGCCGTGTTAACGACTTCCCCACC

GCTGTTCGCGTCTTCGAGGGTGTCAAGGCCAAGGTCGAGAACCCCGACCAGTACAAGCAA

TATCTCGAGTCTCTCGAGGGTCTGCGCGTCGAGCTGGGCGTCGCTCTCCGGGAGGAGCTC

TACCCCCAGGAGGCG

>CL8.Contig1_All 59 445 COPI-coated vesicle protein, putative [Penicillium digitatum Pd1] >gi|425777159|gb|EKV15343.1| COPI-coated vesicle protein, putative [Penicillium digitatum PHI26]

ACCCAATGCAGCCGTCTTGTCAACATCGCCGTGGGCGCGTTTATGGTGCTCGGTGGTATC

AGTAACTTCTTCACCGGAACCTGGAGTACATTCATTCTGGGCGCATATGTCGTTGTCTTC

GGTCTTGTCGTCGGCGGGCTTGAGTTCCTTCCTCACGTCCCGGACCACGCATACCGTTAT

GCGTCGTTCCTCTTCTCCTTTCTGGGCCGCGGTGTCTTCTACATCTTTATCGGTTCCATT

CTGCTGCACGACCAAGTGCTGCGCATCATTGATGGTTCGCTGATCGCCTTCATCGGTCTC

GGATACATCGCTCTCGAGTTCATTCCCTCTATCGAGCCTCCTTCGAACATGCGCGAATCT

GACCAGGGCTGGGGTGCCGAGCAAGTC

>CL8.Contig2_All 59 184 COPI-coated vesicle protein, putative [Penicillium digitatum Pd1] >gi|425777159|gb|EKV15343.1| COPI-coated vesicle protein, putative [Penicillium digitatum PHI26]

ACCCAATGCAGCCGTCTTGTCAACATCGCCGTGGGCGCGTTTATGGTGCTCGGTGGTATC

AGTAACTTCTTCACCGGAACCTGGAGTACATTCATTCTGGGCGCATATGTCGTTGTCTTC

GGTCTT

>CL8.Contig3_All 233 628 minus strand COPI-coated vesicle protein, putative [Penicillium digitatum Pd1] >gi|425777159|gb|EKV15343.1| COPI-coated vesicle protein, putative [Penicillium digitatum PHI26]

ATGGATTTTACAAACATCTTCCGTCTTGTCAACATCGCCGTGGGCGCGTTTATGGTGCTC

GGTGGTATCAGTAACTTCTTCACCGGAACCTGGAGTACATTCATTCTGGGCGCATATGTC

GTTGTCTTCGGTCTTGTCGTCGGCGGGCTTGAGTTCCTTCCTCACGTCCCGGACCACGCA

TACCGTTATGCGTCGTTCCTCTTCTCCTTTCTGGGCCGCGGTGTCTTCTACATCTTTATC

GGTTCCATTCTGCTGCACGACCAAGTGCTGCGCATCATTGATGGTTCGCTGATCGCCTTC

ATCGGTCTCGGATACATCGCTCTCGAGTTCATTCCCTCTATCGAGCCTCCTTCGAACATG

CGCGAATCTGACCAGGGCTGGGGTGCCGAGCAAGTC

>CL8.Contig4_All 254 649 minus strand COPI-coated vesicle protein, putative [Penicillium digitatum Pd1] >gi|425777159|gb|EKV15343.1| COPI-coated vesicle protein, putative [Penicillium digitatum PHI26]

ATGGATTTTACAAACATCTTCCGTCTTGTCAACATCGCCGTGGGCGCGTTTATGGTGCTC

GGTGGTATCAGTAACTTCTTCACCGGAACCTGGAGTACATTCATTCTGGGCGCATATGTC

GTTGTCTTCGGTCTTGTCGTCGGCGGGCTTGAGTTCCTTCCTCACGTCCCGGACCACGCA

TACCGTTATGCGTCGTTCCTCTTCTCCTTTCTGGGCCGCGGTGTCTTCTACATCTTTATC

GGTTCCATTCTGCTGCACGACCAAGTGCTGCGCATCATTGATGGTTCGCTGATCGCCTTC

ATCGGTCTCGGATACATCGCTCTCGAGTTCATTCCCTCTATCGAGCCTCCTTCGAACATG

CGCGAATCTGACCAGGGCTGGGGTGCCGAGCAAGTC

>CL9.Contig1_All 92 256 hypothetical protein PDIP_85720 [Penicillium digitatum Pd1] >gi|425774507|gb|EKV12810.1| hypothetical protein PDIG_40800 [Penicillium digitatum PHI26]

TTCGTCAACGTTCCCATCAACACAGTCAACAAGCGCCTTACGCGCGAGAGCGGCGAGGCC

ACCGCTGAGATCGCCAACCTCGAGAAAAAGCTGCACTATCACGAGACGACAAACCAAAAG

AGTCGGGAGAACCTGGAGCAGATTCTCAAGTCCGGTGGAAGGTCG

>CL9.Contig2_All 191 553 hypothetical protein PDIP_85720 [Penicillium digitatum Pd1] >gi|425774507|gb|EKV12810.1| hypothetical protein PDIG_40800 [Penicillium digitatum PHI26]

ATGTCAATCCCCAATGAAGCTCTCCAAAAGCTCCTTCAGGAGATTGAAGCCCGTGCGATC

GCCTCGCAGCAGCAGATCAGTATCACTAAGGCTCATATGACCTCCAAGCAGCGCGATATC

CGCATGCTACAGCTTACATCGAAGGAGTTGTCTGAGTTGCCTTCCGAGACAAAGGTTTAT

GAGGGCGTTGGAAAAATGTTCGTCAACGTTCCCATCAACACAGTCAACAAGCGCCTTACG

CGCGAGAGCGGCGAGGCCACCGCTGAGATCGCCAACCTCGAGAAAAAGCTGCACTATCAC

GAGACGACAAACCAAAAGAGTCGGGAGAACCTGGAGCAGATTCTCAAGTCCGGTGGAAGG

TCG

>CL9.Contig3_All 92 256 hypothetical protein PDIP_85720 [Penicillium digitatum Pd1] >gi|425774507|gb|EKV12810.1| hypothetical protein PDIG_40800 [Penicillium digitatum PHI26]

TTCGTCAACGTTCCCATCAACACAGTCAACAAGCGCCTTACGCGCGAGAGCGGCGAGGCC

ACCGCTGAGATCGCCAACCTCGAGAAAAAGCTGCACTATCACGAGACGACAAACCAAAAG

AGTCGGGAGAACCTGGAGCAGATTCTCAAGTCCGGTGGAAGGTCG

>CL9.Contig4_All 191 553 hypothetical protein PDIP_85720 [Penicillium digitatum Pd1] >gi|425774507|gb|EKV12810.1| hypothetical protein PDIG_40800 [Penicillium digitatum PHI26]

ATGTCAATCCCCAATGAAGCTCTCCAAAAGCTCCTTCAGGAGATTGAAGCCCGTGCGATC

GCCTCGCAGCAGCAGATCAGTATCACTAAGGCTCATATGACCTCCAAGCAGCGCGATATC

CGCATGCTACAGCTTACATCGAAGGAGTTGTCTGAGTTGCCTTCCGAGACAAAGGTTTAT

GAGGGCGTTGGAAAAATGTTCGTCAACGTTCCCATCAACACAGTCAACAAGCGCCTTACG

CGCGAGAGCGGCGAGGCCACCGCTGAGATCGCCAACCTCGAGAAAAAGCTGCACTATCAC

GAGACGACAAACCAAAAGAGTCGGGAGAACCTGGAGCAGATTCTCAAGTCCGGTGGAAGG

TCG

>CL9.Contig5_All 192 554 hypothetical protein PDIP_85720 [Penicillium digitatum Pd1] >gi|425774507|gb|EKV12810.1| hypothetical protein PDIG_40800 [Penicillium digitatum PHI26]

ATGTCAATCCCCAATGAAGCTCTCCAAAAGCTCCTTCAGGAGATTGAAGCCCGTGCGATC

GCCTCGCAGCAGCAGATCAGTATCACTAAGGCTCATATGACCTCCAAGCAGCGCGATATC

CGCATGCTACAGCTTACATCGAAGGAGTTGTCTGAGTTGCCTTCCGAGACAAAGGTTTAT

GAGGGCGTTGGAAAAATGTTCGTCAACGTTCCCATCAACACAGTCAACAAGCGCCTTACG

CGCGAGAGCGGCGAGGCCACCGCTGAGATCGCCAACCTCGAGAAAAAGCTGCACTATCAC

GAGACGACAAACCAAAAGAGTCGGGAGAACCTGGAGCAGATTCTCAAGTCCGGTGGAAGG

TCG

>CL9.Contig6_All 92 256 hypothetical protein PDIP_85720 [Penicillium digitatum Pd1] >gi|425774507|gb|EKV12810.1| hypothetical protein PDIG_40800 [Penicillium digitatum PHI26]

TTCGTCAACGTTCCCATCAACACAGTCAACAAGCGCCTTACGCGCGAGAGCGGCGAGGCC

ACCGCTGAGATCGCCAACCTCGAGAAAAAGCTGCACTATCACGAGACGACAAACCAAAAG

AGTCGGGAGAACCTGGAGCAGATTCTCAAGTCCGGTGGAAGGTCG

>CL9.Contig7_All 92 256 hypothetical protein PDIP_85720 [Penicillium digitatum Pd1] >gi|425774507|gb|EKV12810.1| hypothetical protein PDIG_40800 [Penicillium digitatum PHI26]

TTCGTCAACGTTCCCATCAACACAGTCAACAAGCGCCTTACGCGCGAGAGCGGCGAGGCC

ACCGCTGAGATCGCCAACCTCGAGAAAAAGCTGCACTATCACGAGACGACAAACCAAAAG

AGTCGGGAGAACCTGGAGCAGATTCTCAAGTCCGGTGGAAGGTCG

>CL9.Contig8_All 192 554 hypothetical protein PDIP_85720 [Penicillium digitatum Pd1] >gi|425774507|gb|EKV12810.1| hypothetical protein PDIG_40800 [Penicillium digitatum PHI26]

ATGTCAATCCCCAATGAAGCTCTCCAAAAGCTCCTTCAGGAGATTGAAGCCCGTGCGATC

GCCTCGCAGCAGCAGATCAGTATCACTAAGGCTCATATGACCTCCAAGCAGCGCGATATC

CGCATGCTACAGCTTACATCGAAGGAGTTGTCTGAGTTGCCTTCCGAGACAAAGGTTTAT

GAGGGCGTTGGAAAAATGTTCGTCAACGTTCCCATCAACACAGTCAACAAGCGCCTTACG

CGCGAGAGCGGCGAGGCCACCGCTGAGATCGCCAACCTCGAGAAAAAGCTGCACTATCAC

GAGACGACAAACCAAAAGAGTCGGGAGAACCTGGAGCAGATTCTCAAGTCCGGTGGAAGG

TCG

>CL10.Contig1_All 2 1438 TMEM1 family protein, putative [Penicillium digitatum PHI26] >gi|425780445|gb|EKV18452.1| TMEM1 family protein, putative [Penicillium digitatum Pd1]

CCAGGTACTGCCGGCCTGCGGCTTCGCCTTGCAGAAACAGAGGTCGTGGATGGTAATATT

GAGGTAGATGCCAACCACGATTCGGGCATCATCGAATTCACGCAAATGCCGGCGCGGTCA

TTCATCCGACTGCGTGTACCATATACTGTAGAGGAGGCGTTCTCGACCCTGTCGGCGCGA

GCGGAGATCGCGTATGAGACCGAGCAAGGCCGATTCGCTTCCTCGGCATCGTTCAATGTT

GTTTCAACACTACCTATATCGGTCAACGTGCAGGACATTTTCAAGGATGAATTACTATTC

TCTCGATTCACCATCAGCCCGGCCATGCTGATCCCGCTTCGCATCATAAACTGCAGCCTG

CCTAGCTCAGACATCTACGACGTGCAATCCAGCATTACTGGCCCCGTCGCCCTTGATGTC

TTCCCTAAGCAGCCCGCATCTCTTCTTTACAAAATTCGCCATACTAAAGAAAACCCAGTC

GCATCAACCCCGCATAGCCTACAATTGGGCGTTGACTTCACATGTGTCGATGATGAGTGT

CTGGATGCAGTTGAAAAGCAATTTGCTGCTTCCATTGCCTCCAGCCCTTTCCGCCAGTAC

GCCGCGCTGCTCACATCGCACATTGTCTGCAGTTTCCGCGCACAGCTGTCCACGAATGAC

ATGGAAGCCATAGGGCTCGTTCGAGAGGTAAACATGCTACCCTATCAGACTGTGCAATGG

GACGATCTGCTCGGAGCTTTGAAAGTACCAGGGGAGGATGTGCGACAATGGCTCCAGCAG

TGGCACAAGAACAATTCCACCATCCCCCTCCCCGCACAACCTACCATCCCAACCCGCCGC

ATCATCATCCCTGTCGACGTCCCCGAGATCCAAGTAGTTCACACAGCCGACCTCCAGCTC

CAGCCCAAACCCACTACCGGCCCAGGGCCCTCCACGCACGCCGCCGTCGGCCAGATGATC

ACCGCTGAGCTAAGCCTCCGCCATACGCGACGCTGGTGTTCCCCAGCGAACCGAGAAAAC

GCCGACCAACCACTCGAATGCTCCTACGAGATCCACGCCAGTCCAGACCAGTGGCAAATA

GGCGGTCGTCGCCGGGGCAACTTCCTCGCACGTGACGGCGAAACCACCCGCTTCACCGTC

CTACTCCTCCCGCAGAAACCAGGATATCTACTTGTCCCAACTCTTGAAATCCGTACCTTC

CTACCATCGGCACCTCAGCTCCGGGCGTCGCCGCCTGCGGCTGATGCCACTGGTTCCATG

GCGCCGGCTCGTCGCTCAATCCCTTGTGAGGTTGACTACCGTAGTCATGGTGAGACGGTT

CTGGTTCTCCCGAATTTGAAGAAGACGACTGTCAATCTGAGTGCCGCTGGCGGTAATCAT

GCTGCTGGTGGGGGATCGTGGCTCGTTGATTCGGAGCGGCGGACGGAGCCGGTTCGA

>CL10.Contig2_All 2 1492 TMEM1 family protein, putative [Penicillium digitatum PHI26] >gi|425780445|gb|EKV18452.1| TMEM1 family protein, putative [Penicillium digitatum Pd1]

CCAGGTACTGCCGGCCTGCGGCTTCGCCTTGCAGAAACAGAGGTCGTGGATGGTAATATT

GAGGTAGATGCCAACCACGATTCGGGCATCATCGAATTCACGCAAATGCCGGCGCGGTCA

TTCATCCGACTGCGTGTACCATATACTGTAGAGGAGGCGTTCTCGACCCTGTCGGCGCGA

GCGGAGATCGCGTATGAGACCGAGCAAGGCCGATTCGCTTCCTCGGCATCGTTCAATGTT

GTTTCAACACTACCTATATCGGTCAACGTGCAGGACATTTTCAAGGATGAATTACTATTC

TCTCGATTCACCATCAGCCCGGCCATGCTGATCCCGCTTCGCATCATAAACTGCAGCCTG

CCTAGCTCAGACATCTACGACGTGCAATCCAGCATTACTGGCCCCGTCGCCCTTGATGTC

TTCCCTAAGCAGCCCGCATCTCTTCTTTACAAAATTCGCCATACTAAAGAAAACCCAGTC

GCATCAACCCCGCATAGCCTACAATTGGGCGTTGACTTCACATGTGTCGATGATGAGTGT

CTGGATGCAGTTGAAAAGCAATTTGCTGCTTCCATTGCCTCCAGCCCTTTCCGCCAGTAC

GCCGCGCTGCTCACATCGCACATTGTCTGCAGTTTCCGCGCACAGCTGTCCACGAATGAC

ATGGAAGCCATAGGGCTCGTTCGAGAGGTAAACATGCTACCCTATCAGACTGTGCAATGG

GACGATCTGCTCGGAGCTTTGAAAGTACCAGGGGAGGATGTGCGACAATGGCTCCAGCAG

TGGCACAAGGTAAGAGCGAATGCGAGTGTTTTCAGCCAGCCATCTTACTCACATATGAAA

CAGAACAATTCCACCATCCCCCTCCCCGCACAACCTACCATCCCAACCCGCCGCATCATC

ATCCCTGTCGACGTCCCCGAGATCCAAGTAGTTCACACAGCCGACCTCCAGCTCCAGCCC

AAACCCACTACCGGCCCAGGGCCCTCCACGCACGCCGCCGTCGGCCAGATGATCACCGCT

GAGCTAAGCCTCCGCCATACGCGACGCTGGTGTTCCCCAGCGAACCGAGAAAACGCCGAC

CAACCACTCGAATGCTCCTACGAGATCCACGCCAGTCCAGACCAGTGGCAAATAGGCGGT

CGTCGCCGGGGCAACTTCCTCGCACGTGACGGCGAAACCACCCGCTTCACCGTCCTACTC

CTCCCGCAGAAACCAGGATATCTACTTGTCCCAACTCTTGAAATCCGTACCTTCCTACCA

TCGGCACCTCAGCTCCGGGCGTCGCCGCCTGCGGCTGATGCCACTGGTTCCATGGCGCCG

GCTCGTCGCTCAATCCCTTGTGAGGTTGACTACCGTAGTCATGGTGAGACGGTTCTGGTT

CTCCCGAATTTGAAGAAGACGACTGTCAATCTGAGTGCCGCTGGCGGTAATCATGCTGCT

GGTGGGGGATCGTGGCTCGTTGATTCGGAGCGGCGGACGGAGCCGGTTCGA

>CL11.Contig1_All 1 294 minus strand DNA ligase 4 [Penicillium digitatum Pd1] >gi|425777342|gb|EKV15520.1| DNA ligase 4 [Penicillium digitatum PHI26]

TTCCACTCGAACGGGGATCGGCAGGACGAATCAGCCTCCCAAGAACTGTGGAAGAAGAGC

CAACCACTCTACTTGGCTCGCAACACAGCCCGGTTTGCAGGTGCCGAGAGCGCCAGCTCC

TTGAAGAGCTCGGGCACCACACATGTGATAGTCGACCCGGAGACTTTGTCGTCAGCGGAC

ATATCATCTCTCCGAAAGTCTCTGGCCGAGAAGCCAGGTGCGAAAATGCCGCATCTTGTT

AGTGCGAGCTGGGTGGACGAATGCTGGAAAAATCGCACATTGCTGGATGAAGAG

>CL11.Contig2_All 2 325 minus strand DNA ligase 4 [Penicillium digitatum Pd1] >gi|425777342|gb|EKV15520.1| DNA ligase 4 [Penicillium digitatum PHI26]

TTGAAGTTTTACTTCCACTCGAACGGGGATCGGCAGGACGAATCAGCCTCCCAAGAACTG

TGGAAGAAGAGCCAACCACTCTACTTGGCTCGCAACACAGCCCGGTTTGCAGGTGCCGAG

AGCGCCAGCTCCTTGAAGAGCTCGGGCACCACACATGTGATAGTCGACCCGGAGACTTTG

TCGTCAGCGGACATATCATCTCTCCGAAAGTCTCTGGCCGAGAAGCCAGGTGCGAAAATG

CCGCATCTTGTTAGTGCGAGCTGGGTGGACGAATGCTGGAAAAATCGCACATTGCTGGAT

GAAGAGAGATTCCCGGTTCCGCGA

>CL13.Contig1_All 75 467 Pc12g13990 [Penicillium chrysogenum Wisconsin 54-1255] >gi|211582824|emb|CAP81026.1| Pc12g13990 [Penicillium chrysogenum Wisconsin 54-1255]

ATGGGCGAGCACTCAGCAATCTGGCAAAGCTATGTCGACTCCAGCCTAATGGGCTCGGGT

CAATTCGACAAGGCCGGTATCCTTGCCGCTGACTTCTCCGGTGTTGAGGCTGCCTCTCCC

GGATTCGCGCTCTCCCAGGAGGAGATTAACTCCCTGATCACCGCCTACACTTCTAGTGAC

CAGGCCTTTGCCAGTGGATTCTCTCTCTGCGGTGAGAAGTTCGTCACTATCCGGGCTGAC

GAGCGCAGTCTATACGGCAAGAAGGGCAAGGAGGGTGTTATCATTGCCCGTGCCTCCTCT

TGCACCATCATCGCCCACCACACTGAGGCCGTCCAGACCCCCAACGCGGCCACTGTTGTC

GAGAACCTTGTCGACTACCTCAACAACCCCCAG

>CL13.Contig2_All 93 485 Pc12g13990 [Penicillium chrysogenum Wisconsin 54-1255] >gi|211582824|emb|CAP81026.1| Pc12g13990 [Penicillium chrysogenum Wisconsin 54-1255]

ATGGGCGAGCACTCAGCAATCTGGCAAAGCTATGTCGACTCCAGCCTAATGGGCTCGGGT

CAATTCGACAAGGCCGGTATCCTTGCCGCTGACTTCTCCGGTGTTGAGGCTGCCTCTCCC

GGATTCGCGCTCTCCCAGGAGGAGATTAACTCCCTGATCACCGCCTACACTTCTAGTGAC

CAGGCCTTTGCCAGTGGATTCTCTCTCTGCGGTGAGAAGTTCGTCACTATCCGGGCTGAC

GAGCGCAGTCTATACGGCAAGAAGGGCAAGGAGGGTGTTATCATTGCCCGTGCCTCCTCT

TGCACCATCATCGCCCACCACACTGAGGCCGTCCAGACCCCCAACGCGGCCACTGTTGTC

GAGAACCTTGTCGACTACCTCAACAACCCCCAG

>CL14.Contig1_All 216 2051 FGGY-family carbohydrate kinase, putative [Penicillium digitatum PHI26] >gi|425782667|gb|EKV20564.1| FGGY-family carbohydrate kinase, putative [Penicillium digitatum Pd1]

ATGGTTTCACTGCCCTACCATCGTGCCTCCCACCGGAGCTCTCGGAAAGCGTCCGGCGAC

GCTCGGGAGGATCTGACAGTTCATCTCGACCACTATATTGGCATTGATATCGGAACTGGC

AGTGCCCGTGCCTGTATCATTAACGCTAACGGTGATATTGTGGGGTTGGCCTCAGAAAAT

ATTGGAATTTGGCAACCAGAGCACGGTTACTATGAACAATCTACCTCTGATATCTGGAAT

TGCATTTGTGTCGCCGTTCAGCGCGCTATTAGCCAGCAAAACATTGACCCGGACACGGTT

CGAGGTATCGGGTTTGATGCCACCTGCTCCTTGTCGGTTTTCTCCAATGATACCGACGAT

CCAATCTCTGTAACGGGCCCGAACTTCGATTCGGACCGCAACGTGATATTGTGGCTGGAT

CACCGTCCAGTAGAGGAAGCTGCGAAGATCAATGCTAGCAACCATAACCTGCTCCGCTAT

GTCGGTGGAAAGATGTCTGTTGAGATGGAGATCCCGAAGGTCCTTTGGCTCAAGAATCAC

ATGCCTAAGGAGCTTTTTGACAAATGCAAGTTCTATGATCTGGCGGATGCACTTACGCAC

ATTGCCACGGGCAATGAAAAGCGAAGTTTCTGCAGTGTGGTCTGCAAGCAAGGATATGTG

CCTGTTGGTGTCGATGGGAGCGTCAAGGGATGGCAAAAAGAATTTTTGCAGGGCATTGGT

CTGGGTGACCTGACCGAAGATAACTTCAAGCGCATGGGCGGAGTCGACGGGGTGAATGGT

GATTATTTAAGTGCCGGTGAATTGGTTGGCACTCTCTGCAAAAAGGCTGCAGCCGAACTA

GGCTTACCAGTTGGTATTGCCATTGGCAGTGGCGTAATTGACGCCTACGCGGGCTGGATC

GGTACCGTCGGATCCAAGGTCGATTTGGACGCAGGTCAATCCAGCGCAGATGTCCCCAAA

CTTAATAAATCAGAGGCCTTCTCTCGCCTCGCGGCTGTCGCGGGAACCTCGACATGTCAC

CTTGCCATGTCACCAGATCCGGTTTTTGTTGATGGTGTCTGGGGTCCATATCGGGATATC

ATCCTCCCCGGATACTGGATGGCCGAGGGCGGGCAATCTGCCACGGGGGAATTGCTCAAA

CATGTGATTGAGACTCACCCGGCATTTAACCAGGCTACTTCAATTGCCGAGTCATACAAT

GCCAACATATACGAATATCTGAACGGGCATCTCAAAGAGATGGCACATAGCCAAGGAGCC

CCATGTGTATCTTATCTCGGACGCCACTTTTTTTTCTATGGTGATCTATGGGGCAACCGC

TCTCCCATCGCCGATCCAAACATGACTGGATCCATCTTTGGCCTAACTAGTGACAAATCT

GTGGATGGTCTTGCCATTTACTACTACGCTACCATGGAGTTCATTGCGCTGCAAACCAAG

CAGATTGTAGAGACAATGAACAAGTCAGGCCATCGCATCACTTCGGTGTTCATGTCGGGC

TCACAATGCCAGAATGAAATCCTAGTCAATCTCATTGCATCTGCCTGTGACATGCCTGTG

ATGATTCCTCGGTATATTCATGCTGCTGTATGCCACGGTGCTGCTATGCTCGGCGCCAAA

GCTGCCAGCGCAGATTCCCAGGGCATAACTGAAGATCTATGGGATATCATGGAACGGATG

AGCAAGCCTGGCAAGAAGGCGTTGCCGACGGAGGATAAGAACGAGAAAGCCTTGCTCAGC

GTTAAATATGAGGTATTTCTGGAGCAGTGCTTCAAGCAACAGAAATACCGTGCGATGGTG

GATGACGTTGTGGGGGCCTGGATGTCCAAGTCCATC

>CL14.Contig2_All 278 2113 FGGY-family carbohydrate kinase, putative [Penicillium digitatum PHI26] >gi|425782667|gb|EKV20564.1| FGGY-family carbohydrate kinase, putative [Penicillium digitatum Pd1]

ATGGTTTCACTGCCCTACCATCGTGCCTCCCACCGGAGCTCTCGGAAAGCGTCCGGCGAC

GCTCGGGAGGATCTGACAGTTCATCTCGACCACTATATTGGCATTGATATCGGAACTGGC

AGTGCCCGTGCCTGTATCATTAACGCTAACGGTGATATTGTGGGGTTGGCCTCAGAAAAT

ATTGGAATTTGGCAACCAGAGCACGGTTACTATGAACAATCTACCTCTGATATCTGGAAT

TGCATTTGTGTCGCCGTTCAGCGCGCTATTAGCCAGCAAAACATTGACCCGGACACGGTT

CGAGGTATCGGGTTTGATGCCACCTGCTCCTTGTCGGTTTTCTCCAATGATACCGACGAT

CCAATCTCTGTAACGGGCCCGAACTTCGATTCGGACCGCAACGTGATATTGTGGCTGGAT

CACCGTCCAGTAGAGGAAGCTGCGAAGATCAATGCTAGCAACCATAACCTGCTCCGCTAT

GTCGGTGGAAAGATGTCTGTTGAGATGGAGATCCCGAAGGTCCTTTGGCTCAAGAATCAC

ATGCCTAAGGAGCTTTTTGACAAATGCAAGTTCTATGATCTGGCGGATGCACTTACGCAC

ATTGCCACGGGCAATGAAAAGCGAAGTTTCTGCAGTGTGGTCTGCAAGCAAGGATATGTG

CCTGTTGGTGTCGATGGGAGCGTCAAGGGATGGCAAAAAGAATTTTTGCAGGGCATTGGT

CTGGGTGACCTGACCGAAGATAACTTCAAGCGCATGGGCGGAGTCGACGGGGTGAATGGT

GATTATTTAAGTGCCGGTGAATTGGTTGGCACTCTCTGCAAAAAGGCTGCAGCCGAACTA

GGCTTACCAGTTGGTATTGCCATTGGCAGTGGCGTAATTGACGCCTACGCGGGCTGGATC

GGTACCGTCGGATCCAAGGTCGATTTGGACGCAGGTCAATCCAGCGCAGATGTCCCCAAA

CTTAATAAATCAGAGGCCTTCTCTCGCCTCGCGGCTGTCGCGGGAACCTCGACATGTCAC

CTTGCCATGTCACCAGATCCGGTTTTTGTTGATGGTGTCTGGGGTCCATATCGGGATATC

ATCCTCCCCGGATACTGGATGGCCGAGGGCGGGCAATCTGCCACGGGGGAATTGCTCAAA

CATGTGATTGAGACTCACCCGGCATTTAACCAGGCTACTTCAATTGCCGAGTCATACAAT

GCCAACATATACGAATATCTGAACGGGCATCTCAAAGAGATGGCACATAGCCAAGGAGCC

CCATGTGTATCTTATCTCGGACGCCACTTTTTTTTCTATGGTGATCTATGGGGCAACCGC

TCTCCCATCGCCGATCCAAACATGACTGGATCCATCTTTGGCCTAACTAGTGACAAATCT

GTGGATGGTCTTGCCATTTACTACTACGCTACCATGGAGTTCATTGCGCTGCAAACCAAG

CAGATTGTAGAGACAATGAACAAGTCAGGCCATCGCATCACTTCGGTGTTCATGTCGGGC

TCACAATGCCAGAATGAAATCCTAGTCAATCTCATTGCATCTGCCTGTGACATGCCTGTG

ATGATTCCTCGGTATATTCATGCTGCTGTATGCCACGGTGCTGCTATGCTCGGCGCCAAA

GCTGCCAGCGCAGATTCCCAGGGCATAACTGAAGATCTATGGGATATCATGGAACGGATG

AGCAAGCCTGGCAAGAAGGCGTTGCCGACGGAGGATAAGAACGAGAAAGCCTTGCTCAGC

GTTAAATATGAGGTATTTCTGGAGCAGTGCTTCAAGCAACAGAAATACCGTGCGATGGTG

GATGACGTTGTGGGGGCCTGGATGTCCAAGTCCATC

>CL15.Contig1_All 278 625 minus strand Pc16g11450 [Penicillium chrysogenum Wisconsin 54-1255] >gi|211586071|emb|CAP93815.1| Pc16g11450 [Penicillium chrysogenum Wisconsin 54-1255]

AAGTCCGCCACCGCAGCTGGGTAAGAACAAGTGCAAGAGACATAGCAATTAGCCTCGACC

GGTGTAACGGACTATCCCAATGGTCACCCCTCAAGTCGCAATTCAAACGTCGATGAAGAA

GCACCCCGGTCGGCATTTTCCGTTGTCGGGCCCCCAGCGGAGCTATCCATACTCACAACC

CATTTGATCAGTCAACAGTGCCAACGACACATTTCCAGATGAAGGACTGCAGGCATGGTG

TATTGTGGCGGGCTCATTCGGTCTCTCGATGGGTACAGTTGGCGTGATGCAACGCGACTG

GAAGTCTGCAAAAATTACTTCGTCACGCATCAGCTAGCCAGCTACACA

>CL15.Contig2_All 278 625 minus strand Pc16g11450 [Penicillium chrysogenum Wisconsin 54-1255] >gi|211586071|emb|CAP93815.1| Pc16g11450 [Penicillium chrysogenum Wisconsin 54-1255]

AAGTCCGCCACCGCAGCTGGGTAAGAACAAGTGCAAGAGACATAGCAATTAGCCTCGACC

GGTGTAACGGACTATCCCAATGGTCACCCCTCAAGTCGCAATTCAAACGTCGATGAAGAA

GCACCCCGGTCGGCATTTTCCGTTGTCGGGCCCCCAGCGGAGCTATCCATACTCACAACC

CATTTGATCAGTCAACAGTGCCAACGACACATTTCCAGATGAAGGACTGCAGGCATGGTG

TATTGTGGCGGGCTCATTCGGTCTCTCGATGGGTACAGTTGGCGTGATGCAACGCGACTG

GAAGTCTGCAAAAATTACTTCGTCACGCATCAGCTAGCCAGCTACACA

>CL15.Contig3_All 1592 1753 minus strand Pc16g11450 [Penicillium chrysogenum Wisconsin 54-1255] >gi|211586071|emb|CAP93815.1| Pc16g11450 [Penicillium chrysogenum Wisconsin 54-1255]

AGCACACTTATCTGTGTCCCCACTGGCGGCGGAATGCTCGACAAAGTCGGGAAGAGAGCC

ATGATGGTATACAGGGGAAGCATTTGAATCGAATCACTTGGTTTGATCGAGATGGCCAGA

TGGGCCTGTTTGAGGCACCGTTGGATATGGCAAGCAAAGATT

>CL15.Contig4_All 278 625 minus strand Pc16g11450 [Penicillium chrysogenum Wisconsin 54-1255] >gi|211586071|emb|CAP93815.1| Pc16g11450 [Penicillium chrysogenum Wisconsin 54-1255]

AAGTCCGCCACCGCAGCTGGGTAAGAACAAGTGCAAGAGACATAGCAATTAGCCTCGACC

GGTGTAACGGACTATCCCAATGGTCACCCCTCAAGTCGCAATTCAAACGTCGATGAAGAA

GCACCCCGGTCGGCATTTTCCGTTGTCGGGCCCCCAGCGGAGCTATCCATACTCACAACC

CATTTGATCAGTCAACAGTGCCAACGACACATTTCCAGATGAAGGACTGCAGGCATGGTG

TATTGTGGCGGGCTCATTCGGTCTCTCGATGGGTACAGTTGGCGTGATGCAACGCGACTG

GAAGTCTGCAAAAATTACTTCGTCACGCATCAGCTAGCCAGCTACACA

>CL16.Contig1_All 266 847 minus strand UPF0619 GPI-anchored membrane protein [Penicillium digitatum Pd1] >gi|425780322|gb|EKV18332.1| GPI-anchored membrane protein [Penicillium digitatum PHI26]

ATGCGTTTCTCCACCATTCTGTCTCTTCTGCCCCTGGCCCTCTCCGTTGCGGCCATCAAC

GTCACCGAGCCCGCCAAGGGTGCCGAGGTCGATGTCTCCGGCTCTTTCACCGTGAAGTGG

AATTCCGTTGATACCGATGCTTCCACCGTTGACATTGTGCTCGTCAACAATGCCGTGTTC

CCTACCGTCTCGGAGAAGATTGCCTCCGGCGTGGACACCTCCAAGGGCAGCTACGATGCC

TCCGGCCTGAAGGGTATCACCAGCGGCCCCGGTTTCCAGATCAACCTGCTTTCCACCGAT

GCCAAGAACACCGGCATCCTCGCCCAGTCCCAGCAGTTCGATGTGACCGAGTCCAAGAGC

TCCTCCGCTACTACTACTGCTACCGGTTCTTCTACCACCTCTACCGGAACTTCCACTGCC

ACCAAGACCTCCACCAAGACCGGTCTGACCACCAGCGCTTCCTCCACCGGCACCGCTACT

GGCTCTGCCGCCGCCTCCGCCACTGCCTCCACCGGCGCTGCTATGGGTCTCGTTGCTCCC

GGTGCCGCTGCTGGTCTTCTGGCTGGTGTCCTCGCTTTCCTG

>CL16.Contig2_All 266 970 minus strand UPF0619 GPI-anchored membrane protein [Penicillium digitatum Pd1] >gi|425780322|gb|EKV18332.1| GPI-anchored membrane protein [Penicillium digitatum PHI26]

ATGCGTTTCTCCACCATTCTGTCTCTTCTGCCCCTGGCCCTCTCCGTTGCGGCCATCAAC

GTCACCGAGCCCGCCAAGGGTGCCGAGGTCGATGTCTCCGGCTCTTTCACCGTGAAGTGG

AATTCCGTTGATACCGATGCTTCCACCGTTGACATTGTGCTCGTCAACAATGCCGTGTTC

CCTACCGTCTCGGAGAAGATTGCCTCCGGCGTGGACACCTCCAAGGGCAGCTACGATGCC

TCCGGCCTGAAGGGTATCACCAGCGGCCCCGGTTTCCAGATCAACCTGCTTTCCACCGAT

GCCAAGAACACCGGCATCCTCGCCCAGTCCCAGCAGTTCGATGTGACCGAGTCCAAGAGC

TCCTCCGCTACTACTACTGGTACATCTTCCACTGTCTCTTCCTCTTCTACTTCCTCTACC

TCTGTTGTCTCTTCCGAGACCTCTTCCACTGCTTCCACCTCCACTGGTGCCACCACCACT

GAGACCGATGCTTCCACTACTGCTACCGGTTCTTCTACCACCTCTACCGGAACTTCCACT

GCCACCAAGACCTCCACCAAGACCGGTCTGACCACCAGCGCTTCCTCCACCGGCACCGCT

ACTGGCTCTGCCGCCGCCTCCGCCACTGCCTCCACCGGCGCTGCTATGGGTCTCGTTGCT

CCCGGTGCCGCTGCTGGTCTTCTGGCTGGTGTCCTCGCTTTCCTG

>CL16.Contig3_All 54 278 UPF0619 GPI-anchored membrane protein [Penicillium digitatum Pd1] >gi|425780322|gb|EKV18332.1| GPI-anchored membrane protein [Penicillium digitatum PHI26]

AGCTCCTCCAGCGCTACTACTACCGCTACCGGTTCTACCTCTACCGGAACTACCACTGGC

ACCGAGACCTCCACCAACACTGGTATGACTACCAGCGCTTCTTCCGCCACTGGCTCTGCT

TCCGGCTCTGCCTCTGCCTCTGCCTCCCCAACTCCTTCCACCGGTGCTGCTATGGGTCTC

GTTGCCCCCGGTGCCGCTGCTGGCCTTCTGGCTGGTGTCCTGGCT

>CL16.Contig4_All 3 422 Pc23g00980 [Penicillium chrysogenum Wisconsin 54-1255] >gi|211904278|emb|CAP79592.1| Pc23g00980 [Penicillium chrysogenum Wisconsin 54-1255]

ACCGGCATCCTCGCCCAGTCCGAGCAGTTCGATGTCACCGAGTCCGACAAGAGCTCCTCC

AGCGCTACTACTACCGGTACATCTTCCACTGTCTCTTCTGCCTCTTCTACTGCTTCTGCT

GCCTCTACCTCTGTTGTCTCTACTGGTACCACTTCCACTGCTTCTACTTCCGCTGGTGTC

TCCTCCACTGAAACCGAGGCTTCCTCTACTGCTACCGGTTCTACCTCTACCGGAACTACC

ACTGGCACCGAGACCTCCACCAACACTGGTATGACTACCAGCGCTTCTTCCGCCACTGGC

TCTGCTTCCGGCTCTGCCTCTGCCTCTGCCTCCCCAACTCCTTCCACCGGTGCTGCTATG

GGTCTCGTTGCCCCCGGTGCCGCTGCTGGCCTTCTGGCTGGTGTCCTGGCTCTCAACCTG

>CL16.Contig5_All 79 402 minus strand Pc16g06160 [Penicillium chrysogenum Wisconsin 54-1255] >gi|211585575|emb|CAP93286.1| Pc16g06160 [Penicillium chrysogenum Wisconsin 54-1255]

ATGCGTTTCGCTACCGTTCTGGCTCTTCTGCCTCTGGCCCTCTCCGTTGGAGCTATCAAG

GTCACTGAGCCCGCTAAGAATGACGAGGTTGATGTGTCTGGCTCTTTCACCGTGAAGTGG

TCGTCCGTTAGCACCGATGCCTCCACTGTTGACATTGTCCTTGTCAACAATGCCGTCTAC

CCCCCTGTTGAGGAGAAGATTGCCTCCGGCATCGACACCTCCAAGGGCTCCTACACTGCC

TCTGGCCTGAAGGACGTCACCGATGGCTCCGGCTTCCAGATCAACCTGATCTCCACTGAG

GCCAAGAACACCGGCATCCTCGCC

>CL17.Contig2_All 170 1951 minus strand BZIP transcription factor (LziP), putative [Penicillium digitatum PHI26] >gi|425783036|gb|EKV20905.1| BZIP transcription factor (LziP), putative [Penicillium digitatum Pd1]

ATGGCGACTCTTGCGGAACATTCGATGCAGCCGGTTGCAAACTATGACCAGGATCTGGAA

TCATTCATCAACTTCGATCAACTCAACTACACAGCCGATCCCTCCCGCTCAAAGGTTATG

GTCAGCCAACCGTCGGTCGCAAGCACCGAGTTCAGTGCCAGTGACGCCCGCAGTGCCAGC

TTTGCCTCAAGTGGCCAGTCTCCCCTCGCATTTCAGGCCCCAAGTCACCAGTATGACGAA

CACAGACAACAGACTGGCCTCCCGCCCGGTGCTTTGTCCATGTCCTACAGTCAGGTCCCG

ATGGGCTTTAACAATGGTCCAGGATTTCCCGTGAACGCCGAGGTGTATGCTGGTCATCAC

ATGAAGCGAGAGAATGCCCAATTTGACTTCAACACTGCACCGCGCCGGAACCCGTCGGAC

ATGGAGATTGATCCGGAAAACATGAACCCCTCGTCATACTTCTACCCGGTAAACCCTGGA

AACAAAAACCAATACGTCGATCCCAACGCTCTCGGAGGCCATGAGCTGGCTCAGGCTGGT

CCATCCACACAGGTCGGTCGCATGTACCCCGGCATGCATCAGCAGCAGGCTGCGATGGCC

AAGGCCCAACAACAGAAGCAAAGCGAGATGGTCCGCCTTCAGATACAGCAGCGTCCGGAT

CAACTGCCCGATGCTGTGCCTCAAGTCCACGGTCCCCGAAATCCTGACCCTGTGGTTGAG

GAGCGCATTTCCCGTCTACTTCAACAGATGCGCCAGAATGCAATGGCCAATGGCGAGGGC

TCCCCCAGTCCCTCCAACGGTATGCCGCAGATGGCCAAGGCCCGGAAGGATGAGGCCGAT

ATGGATGAAGATGAGCGCCTTCTCGCTAGCGAGGAGGGCAAGAAACTCAGCAGCAAAGAG

CGCCGCCAGTTGCGAAACAAGGTCTCCGCTCGCGCTTTCCGATCTCGCCGCAAGGAATAC

ATTGGTCAGCTTGAAAGTGAGGTGGCCGCTAGAACCAATGAGGCCCACGAGGTTCGCCTC

CAGAACCGCGCTCTTTATGAAGAGAATGCCCGCCTGAACGACTTGGCCCGCATGCTTCTG

GGCTCTCCTCATTTCTCCAACTTCTTGAACGATATAGGTGACACAATGCCCAATCAACCC

CAGCAGCAATCTCAACAACAACAACAACAACCTCAGCAACAAGCCCAGCAGGCTGCACCG

CAGCGCAACATGCAGGGTATGCCCAAGGACACGAACGCCAACCGTGGCCAAGAGTTCCAG

ATGCAGCAAACCCCCCAGGCCAACATGGTCATGGTTCCCAACCAGGGTATGGACCCCTCC

AGTATGGGCATGAACAACGGCGGCTGGAACTCGGGAATTGACATGAACTACGGCAACACT

CCCGTCTTTGCTGTCATGGAGGTTCCCGAGGGCCCGGCCTTGGATGCTCAGGTTTTGTCT

GGAAAACCCTTCTCTCCTTTCAGCATCCCCGAGAGTTCCAAGAATGAGGCTCCTATCGTC

GACCGTCCTGCTAGCGACTCGCCCAGCCAGTCCGACATTGGTGCTGCCAACCCTGATGTC

GAGATTGATGAGTCTGATCCTGCCTTTGCTCTCTTTGTTGATTCTCCTGCCCCCGTTACT

AACATCGCATTCGAAGGTGTGTCGACCGAGAAGACTGCTCAGTTTCAACTCGTCGTTGAC

AACTCTGAAGTTAGCGATTCGGCCAAGAAAACCTTCCACGCTCTCTGCAACAGCATTGAC

GCTGCATTCGAGCGTGTCTCAGCTGTCACGTCCCATCTTTGG

>CL18.Contig1_All 1462 2922 Glutamine synthetase, putative [Penicillium digitatum PHI26] >gi|425782516|gb|EKV20422.1| Glutamine synthetase, putative [Penicillium digitatum Pd1]

ATGTCGAGCCCCGCGGACCTCGAGGTCACAGCCGAGAATGTTGCTGAGATCCTCCAGAAT

GACACAAAAGTCAAGGTGGCAGGAGTGGATGTGGATGGCATTCTGCGTGGCAAACTCATG

AAGAAAGACAAGTTCTTATCTATCATCACCGAGGGTTTCGGCTTCTGCTCTGTCATCTTC

GGCTGGGATCAACACGATGCGACGTACTATAAGGAGCTGGCCATCAGCAACAAGGAGAAT

GGATATCGCGATCTTGTCGCAGTCCCAGATTTACGCAGTTTCCGTCGCATTCCATGGGAA

AACAATGTGCCCTTTTTCCTCATCAGCTTCTTGGATCCAGAAACCAGGAAGCCGGTGTGC

GCCTGCCCGCGAGGATTGATAAAGACTGCGGCCGCTAAAGTCGAGGCGGCAGGGTATCGG

GCGATGGCAGGAGCCGAATACGAATTCTACCAATTTCGCACGCCAGGCAATCACTCCACC

CCCGAGCAAGGCGCATCTGCAACGGCGGCCTTTCTCCAGTCGAATCCAGTCGAATCGTTG

CCATCTGTCACAGACGGCATGTTTGGTTATTCAATCACTCGGCCTCTCCATAACCAAGAT

TACTACTATGGCATCTTCGACGCCTGCGAGCAGTTCCGATGCGATATTGAAGGGTGGCAT

ACTGAAAGTGGACCAGGAGTCTATGAAGCTGCATTGCAATTTGGCGAAGCGAAAGACATG

GCTGACAAGGCCGGGTTATTCAAATACGTCGTAAAAGCATTCGGAATTAAGCATGGCATC

ACCCCATGCTTCATGGCGAAACCCCGACAAGGCCTACCAGGAAACAGCGGGCACATGCAC

ATCTCGCTCGTCACAGCCGACGGAAAGAATGCCTTCATTCGCGATGCTCCGGATCCATCC

CCCCTCTACCCTGATATCACTCATCTGTCCGACTTGGGTCGACATTTTCTCGCTGGCCTT

CTCGTCGGCCTGCCCGATATCATGCCAATTTTGGCACCGACGATCAACTCCTACAAACGC

CTGGTGGAGAACTTTTGGGCACCGGTCACTGTTTCTTGGGGACTAGAACACCGGGCCGCG

TCAATCCGTCTGATCACCCCACCGACAGCTAGTCCGAAAGCGACTCGTTTCGAGGTGCGC

GTGCCAGGTGCAGATACCAATGCGCATCTTGTCTTAGCGGCGATACTGGCATTGGGATGG

CGCGGAGTCGAGAAGAAGCTCGAGATCCCGTTGCCGCCTCTTGCCCGTGGCGAGGACATG

GGCGGTGCTAGTGATAAGGGTGTGCGTCTGGCCAAATCCTTGAAGGAGGCTATTGTTACA

TTTACACGGCCGGAGAGTGTCGCACGGGAAGTCTTTGGCGACTCATTCGTCGACCACTTT

GCCGGAACTCGGGAACACGAGGTGCGCTTGTGGGAAGAGGCCGTCACTGATTGGGAGATG

AGGCGGTACATCGAGACCGTG

>CL18.Contig2_All 2397 3989 Glutamine synthetase, putative [Penicillium digitatum PHI26] >gi|425782516|gb|EKV20422.1| Glutamine synthetase, putative [Penicillium digitatum Pd1]

ATGTCGAGCCCCGCGGACCTCGAGGTCACAGCCGAGAATGTTGCTGAGATCCTCCAGAAT

GACACAAAAGTCAAGGTGGCAGGAGTGGATGTGGATGGCATTCTGCGTGGCAAACTCATG

AAGAAAGACAAGTTCTTATCTATCATCACCGAGGGTTTCGGCTTCTGCTCTGTCATCTTC

GGCTGGGATCAACACGATGCGACGTACTATAAGGAGCTGGCCATCAGCAACAAGGAGAAT

GGATATCGCGATCTTGTCGCAGTCCCAGATTTACGCAGTTTCCGTCGCATTCCATGGGAA

AACAATGTGCCCTTTTTCCTCATCAGCTTCTTGGATCCAGAAACCAGGAAGCCGGTGTGC

GCCTGCCCGCGAGGATTGATAAAGACTGCGGCCGCTAAAGTCGAGGCGGCAGGGTATCGG

GCGATGGCAGGAGCCGAATACGAATTCTACCAATTTCGCACGCCAGGCAATCACTCCACC

CCCGAGCAAGGCGCATCTGCAACGGCGGCCTTTCTCCAGTCGAATCCAGTCGAATCGTTG

CCATCTGTCACAGACGGCATGTTTGGTTATTCAATCACTCGGCCTCTCCATAACCAAGAT

TACTACTATGGCATCTTCGACGCCTGCGAGCAGTTCCGATGCGATATTGAAGGGTGGCAT

ACTGAAAGTGGACCAGGAGTCTATGAAGCTGCATTGCAATTTGGCGAAGCGAAAGACATG

GCTGACAAGGCCGGGTTATTCAAGTGAGAGCGTTCTTTTGCCAGTGGTTCGGGTTGTTCT

TTCATGATGGGTCATCGCTAACCAGGACGCTCGCATAGATACGTCGTAAAAGCATTCGGA

ATTAAGCATGGCATCACCCCATGCTTCATGGCGAAACCCCGACAAGGCCTACCAGGAAAC

AGCGGGCACATGCACATCTCGCTCGTCACAGCCGACGGAAAGAATGCCTTCATTCGCGAT

GCTCCGGATCCATCCCCCCTCTACCCTGATATCACTCATCTGTCCGACTTGGGTCGACAT

TTTCTCGCTGGCCTTCTCGTCGGCCTGCCCGATATCATGCCAATTTTGGCACCGACGATC

AACTCCTACAAACGCCTGGTGGAGAACTTTTGGGCACCGGTCACTGTTTCTTGGGGACTA

GAACACCGGGCCGCGTCAATCCGTCTGATCACCCCACCGACAGCTAGTCCGAAAGCGACT

CGTTTCGAGGTGCGCGTGCCAGGTGCAGATACCAATGCGCATCTTGTCTTAGCGGCGATA

CTGGCATTGGGATGGCGCGGAGTCGAGAAGAAGCTCGAGATCCCGTTGCCGCCTCTTGCC

CGTGGCGAGGACATGGGCGGTGCTAGTGATAAGGGTGTGCGTCTGGCCAAATCCTTGAAG

GAGGCTATTGTTACATTTACACGGCCGGAGAGTGTCGCACGGGAAGTCTTTGGCGACTCA

TTCGTCGACCACTTTGCCGGAACTCGGGAACACGAGGTGCGCTTGTGGGAAGAGGCCGTC

ACTGATTGGTATGTTGTATTATCAACTGCGATATTCTTTTGGATGCTGACTTGACCTGTT

CACAGGGAGATGAGGCGGTACATCGAGACCGTG

>CL18.Contig3_All 1462 2997 Glutamine synthetase, putative [Penicillium digitatum PHI26] >gi|425782516|gb|EKV20422.1| Glutamine synthetase, putative [Penicillium digitatum Pd1]

ATGTCGAGCCCCGCGGACCTCGAGGTCACAGCCGAGAATGTTGCTGAGATCCTCCAGAAT

GACACAAAAGTCAAGGTGGCAGGAGTGGATGTGGATGGCATTCTGCGTGGCAAACTCATG

AAGAAAGACAAGTTCTTATCTATCATCACCGAGGGTTTCGGCTTCTGCTCTGTCATCTTC

GGCTGGGATCAACACGATGCGACGTACTATAAGGAGCTGGCCATCAGCAACAAGGAGAAT

GGATATCGCGATCTTGTCGCAGTCCCAGATTTACGCAGTTTCCGTCGCATTCCATGGGAA

AACAATGTGCCCTTTTTCCTCATCAGCTTCTTGGATCCAGAAACCAGGAAGCCGGTGTGC

GCCTGCCCGCGAGGATTGATAAAGACTGCGGCCGCTAAAGTCGAGGCGGCAGGGTATCGG

GCGATGGCAGGAGCCGAATACGAATTCTACCAATTTCGCACGCCAGGCAATCACTCCACC

CCCGAGCAAGGCGCATCTGCAACGGCGGCCTTTCTCCAGTCGAATCCAGTCGAATCGTTG

CCATCTGTCACAGACGGCATGTTTGGTTATTCAATCACTCGGCCTCTCCATAACCAAGAT

TACTACTATGGCATCTTCGACGCCTGCGAGCAGTTCCGATGCGATATTGAAGGGTGGCAT

ACTGAAAGTGGACCAGGAGTCTATGAAGCTGCATTGCAATTTGGCGAAGCGAAAGACATG

GCTGACAAGGCCGGGTTATTCAAGTGAGAGCGTTCTTTTGCCAGTGGTTCGGGTTGTTCT

TTCATGATGGGTCATCGCTAACCAGGACGCTCGCATAGATACGTCGTAAAAGCATTCGGA

ATTAAGCATGGCATCACCCCATGCTTCATGGCGAAACCCCGACAAGGCCTACCAGGAAAC

AGCGGGCACATGCACATCTCGCTCGTCACAGCCGACGGAAAGAATGCCTTCATTCGCGAT

GCTCCGGATCCATCCCCCCTCTACCCTGATATCACTCATCTGTCCGACTTGGGTCGACAT

TTTCTCGCTGGCCTTCTCGTCGGCCTGCCCGATATCATGCCAATTTTGGCACCGACGATC

AACTCCTACAAACGCCTGGTGGAGAACTTTTGGGCACCGGTCACTGTTTCTTGGGGACTA

GAACACCGGGCCGCGTCAATCCGTCTGATCACCCCACCGACAGCTAGTCCGAAAGCGACT

CGTTTCGAGGTGCGCGTGCCAGGTGCAGATACCAATGCGCATCTTGTCTTAGCGGCGATA

CTGGCATTGGGATGGCGCGGAGTCGAGAAGAAGCTCGAGATCCCGTTGCCGCCTCTTGCC

CGTGGCGAGGACATGGGCGGTGCTAGTGATAAGGGTGTGCGTCTGGCCAAATCCTTGAAG

GAGGCTATTGTTACATTTACACGGCCGGAGAGTGTCGCACGGGAAGTCTTTGGCGACTCA

TTCGTCGACCACTTTGCCGGAACTCGGGAACACGAGGTGCGCTTGTGGGAAGAGGCCGTC

ACTGATTGGGAGATGAGGCGGTACATCGAGACCGTG

>CL18.Contig4_All 1535 2995 Glutamine synthetase, putative [Penicillium digitatum PHI26] >gi|425782516|gb|EKV20422.1| Glutamine synthetase, putative [Penicillium digitatum Pd1]

ATGTCGAGCCCCGCGGACCTCGAGGTCACAGCCGAGAATGTTGCTGAGATCCTCCAGAAT

GACACAAAAGTCAAGGTGGCAGGAGTGGATGTGGATGGCATTCTGCGTGGCAAACTCATG

AAGAAAGACAAGTTCTTATCTATCATCACCGAGGGTTTCGGCTTCTGCTCTGTCATCTTC

GGCTGGGATCAACACGATGCGACGTACTATAAGGAGCTGGCCATCAGCAACAAGGAGAAT

GGATATCGCGATCTTGTCGCAGTCCCAGATTTACGCAGTTTCCGTCGCATTCCATGGGAA

AACAATGTGCCCTTTTTCCTCATCAGCTTCTTGGATCCAGAAACCAGGAAGCCGGTGTGC

GCCTGCCCGCGAGGATTGATAAAGACTGCGGCCGCTAAAGTCGAGGCGGCAGGGTATCGG

GCGATGGCAGGAGCCGAATACGAATTCTACCAATTTCGCACGCCAGGCAATCACTCCACC

CCCGAGCAAGGCGCATCTGCAACGGCGGCCTTTCTCCAGTCGAATCCAGTCGAATCGTTG

CCATCTGTCACAGACGGCATGTTTGGTTATTCAATCACTCGGCCTCTCCATAACCAAGAT

TACTACTATGGCATCTTCGACGCCTGCGAGCAGTTCCGATGCGATATTGAAGGGTGGCAT

ACTGAAAGTGGACCAGGAGTCTATGAAGCTGCATTGCAATTTGGCGAAGCGAAAGACATG

GCTGACAAGGCCGGGTTATTCAAATACGTCGTAAAAGCATTCGGAATTAAGCATGGCATC

ACCCCATGCTTCATGGCGAAACCCCGACAAGGCCTACCAGGAAACAGCGGGCACATGCAC

ATCTCGCTCGTCACAGCCGACGGAAAGAATGCCTTCATTCGCGATGCTCCGGATCCATCC

CCCCTCTACCCTGATATCACTCATCTGTCCGACTTGGGTCGACATTTTCTCGCTGGCCTT

CTCGTCGGCCTGCCCGATATCATGCCAATTTTGGCACCGACGATCAACTCCTACAAACGC

CTGGTGGAGAACTTTTGGGCACCGGTCACTGTTTCTTGGGGACTAGAACACCGGGCCGCG

TCAATCCGTCTGATCACCCCACCGACAGCTAGTCCGAAAGCGACTCGTTTCGAGGTGCGC

GTGCCAGGTGCAGATACCAATGCGCATCTTGTCTTAGCGGCGATACTGGCATTGGGATGG

CGCGGAGTCGAGAAGAAGCTCGAGATCCCGTTGCCGCCTCTTGCCCGTGGCGAGGACATG

GGCGGTGCTAGTGATAAGGGTGTGCGTCTGGCCAAATCCTTGAAGGAGGCTATTGTTACA

TTTACACGGCCGGAGAGTGTCGCACGGGAAGTCTTTGGCGACTCATTCGTCGACCACTTT

GCCGGAACTCGGGAACACGAGGTGCGCTTGTGGGAAGAGGCCGTCACTGATTGGGAGATG

AGGCGGTACATCGAGACCGTG

>CL18.Contig5_All 1512 3047 Glutamine synthetase, putative [Penicillium digitatum PHI26] >gi|425782516|gb|EKV20422.1| Glutamine synthetase, putative [Penicillium digitatum Pd1]

ATGTCGAGCCCCGCGGACCTCGAGGTCACAGCCGAGAATGTTGCTGAGATCCTCCAGAAT

GACACAAAAGTCAAGGTGGCAGGAGTGGATGTGGATGGCATTCTGCGTGGCAAACTCATG

AAGAAAGACAAGTTCTTATCTATCATCACCGAGGGTTTCGGCTTCTGCTCTGTCATCTTC

GGCTGGGATCAACACGATGCGACGTACTATAAGGAGCTGGCCATCAGCAACAAGGAGAAT

GGATATCGCGATCTTGTCGCAGTCCCAGATTTACGCAGTTTCCGTCGCATTCCATGGGAA

AACAATGTGCCCTTTTTCCTCATCAGCTTCTTGGATCCAGAAACCAGGAAGCCGGTGTGC

GCCTGCCCGCGAGGATTGATAAAGACTGCGGCCGCTAAAGTCGAGGCGGCAGGGTATCGG

GCGATGGCAGGAGCCGAATACGAATTCTACCAATTTCGCACGCCAGGCAATCACTCCACC

CCCGAGCAAGGCGCATCTGCAACGGCGGCCTTTCTCCAGTCGAATCCAGTCGAATCGTTG

CCATCTGTCACAGACGGCATGTTTGGTTATTCAATCACTCGGCCTCTCCATAACCAAGAT

TACTACTATGGCATCTTCGACGCCTGCGAGCAGTTCCGATGCGATATTGAAGGGTGGCAT

ACTGAAAGTGGACCAGGAGTCTATGAAGCTGCATTGCAATTTGGCGAAGCGAAAGACATG

GCTGACAAGGCCGGGTTATTCAAGTGAGAGCGTTCTTTTGCCAGTGGTTCGGGTTGTTCT

TTCATGATGGGTCATCGCTAACCAGGACGCTCGCATAGATACGTCGTAAAAGCATTCGGA

ATTAAGCATGGCATCACCCCATGCTTCATGGCGAAACCCCGACAAGGCCTACCAGGAAAC

AGCGGGCACATGCACATCTCGCTCGTCACAGCCGACGGAAAGAATGCCTTCATTCGCGAT

GCTCCGGATCCATCCCCCCTCTACCCTGATATCACTCATCTGTCCGACTTGGGTCGACAT

TTTCTCGCTGGCCTTCTCGTCGGCCTGCCCGATATCATGCCAATTTTGGCACCGACGATC

AACTCCTACAAACGCCTGGTGGAGAACTTTTGGGCACCGGTCACTGTTTCTTGGGGACTA

GAACACCGGGCCGCGTCAATCCGTCTGATCACCCCACCGACAGCTAGTCCGAAAGCGACT

CGTTTCGAGGTGCGCGTGCCAGGTGCAGATACCAATGCGCATCTTGTCTTAGCGGCGATA

CTGGCATTGGGATGGCGCGGAGTCGAGAAGAAGCTCGAGATCCCGTTGCCGCCTCTTGCC

CGTGGCGAGGACATGGGCGGTGCTAGTGATAAGGGTGTGCGTCTGGCCAAATCCTTGAAG

GAGGCTATTGTTACATTTACACGGCCGGAGAGTGTCGCACGGGAAGTCTTTGGCGACTCA

TTCGTCGACCACTTTGCCGGAACTCGGGAACACGAGGTGCGCTTGTGGGAAGAGGCCGTC

ACTGATTGGGAGATGAGGCGGTACATCGAGACCGTG

>CL18.Contig6_All 121 963 minus strand Short-chain dehydrogenase/reductase family protein, putative [Penicillium digitatum PHI26] >gi|425782515|gb|EKV20421.1| Short-chain dehydrogenase/reductase family protein, putative [Penicillium digitatum Pd1]

ATGGCTGCCACTACTCCCCAAGGACGTCTCCAAGGCAAGAATGCCATCATCACAGGTGCA

GGCGGTGGCATCGGTCTCGAAACCAGTATTCTCTTCGCCCGCGAGGGTGCCAATGTCCTG

ATGGCCGACGTCTCTGAGCCCGCCCTTGAAAAGGCATTGGCTAAAGTGAAGGAAGTAGTT

CCCTCCGCTCCTCGAGTCGAGTCTATCCGATGTGACGTGTCCAAAGAGTCGGATGTCCAG

GCAATGGTTGAGTACCAGGACAGCTGGGGAGGCACTGACGTGATCTTCAACAATGCCGGA

ATCATGCACGCCAACGATGCAGATGCCATCGATACCCCCGAGAAGATCTGGGACCTCACA

CACAATATCAATGTCAAGGGTGTCTGGTTCGGCAGCAAGCACGCCGTCCTCAGTCTTCGT

CGTCACAAGAAGTCCCGGGGCAGTATTATCAACACAGCTAGTGTGGTCGCCTTGGTCGGC

GCTGCTACTCCTCAGCTCGCGTATACGGCGAGCAAGGGTGCGGTCTTGGCGATGACTCGG

GAATTGGCCATCGTTCACGCCCGTGAGGGATTCCGTTTCAACGCTCTGTGCCCCGCACCC

CTGAACACTCCTCTTTTACAAGACTGGCTGGGTGATGACAAGGCCAAGCGGCTCCGTCGG

GAGGTGCACTTCCCCACTGGCCGGTTTGGTGAACCCATCGAGCAGGCTCACGCAGTGGTT

TTCCTGGCCAGCGATGAGAGCAGCTTCGTGAATGGAACGGACTTTGTGGTCGATGGTGGA

ATGACTAAAGCTTATGTGACCCCGGAGGGTCCAGCAACCCCTGCGCCGCAGAACAACGGC

AAT

>CL18.Contig7_All 1462 2979 Glutamine synthetase, putative [Penicillium digitatum PHI26] >gi|425782516|gb|EKV20422.1| Glutamine synthetase, putative [Penicillium digitatum Pd1]

ATGTCGAGCCCCGCGGACCTCGAGGTCACAGCCGAGAATGTTGCTGAGATCCTCCAGAAT

GACACAAAAGTCAAGGTGGCAGGAGTGGATGTGGATGGCATTCTGCGTGGCAAACTCATG

AAGAAAGACAAGTTCTTATCTATCATCACCGAGGGTTTCGGCTTCTGCTCTGTCATCTTC

GGCTGGGATCAACACGATGCGACGTACTATAAGGAGCTGGCCATCAGCAACAAGGAGAAT

GGATATCGCGATCTTGTCGCAGTCCCAGATTTACGCAGTTTCCGTCGCATTCCATGGGAA

AACAATGTGCCCTTTTTCCTCATCAGCTTCTTGGATCCAGAAACCAGGAAGCCGGTGTGC

GCCTGCCCGCGAGGATTGATAAAGACTGCGGCCGCTAAAGTCGAGGCGGCAGGGTATCGG

GCGATGGCAGGAGCCGAATACGAATTCTACCAATTTCGCACGCCAGGCAATCACTCCACC

CCCGAGCAAGGCGCATCTGCAACGGCGGCCTTTCTCCAGTCGAATCCAGTCGAATCGTTG

CCATCTGTCACAGACGGCATGTTTGGTTATTCAATCACTCGGCCTCTCCATAACCAAGAT

TACTACTATGGCATCTTCGACGCCTGCGAGCAGTTCCGATGCGATATTGAAGGGTGGCAT

ACTGAAAGTGGACCAGGAGTCTATGAAGCTGCATTGCAATTTGGCGAAGCGAAAGACATG

GCTGACAAGGCCGGGTTATTCAAATACGTCGTAAAAGCATTCGGAATTAAGCATGGCATC

ACCCCATGCTTCATGGCGAAACCCCGACAAGGCCTACCAGGAAACAGCGGGCACATGCAC

ATCTCGCTCGTCACAGCCGACGGAAAGAATGCCTTCATTCGCGATGCTCCGGATCCATCC

CCCCTCTACCCTGATATCACTCATCTGTCCGACTTGGGTCGACATTTTCTCGCTGGCCTT

CTCGTCGGCCTGCCCGATATCATGCCAATTTTGGCACCGACGATCAACTCCTACAAACGC

CTGGTGGAGAACTTTTGGGCACCGGTCACTGTTTCTTGGGGACTAGAACACCGGGCCGCG

TCAATCCGTCTGATCACCCCACCGACAGCTAGTCCGAAAGCGACTCGTTTCGAGGTGCGC

GTGCCAGGTGCAGATACCAATGCGCATCTTGTCTTAGCGGCGATACTGGCATTGGGATGG

CGCGGAGTCGAGAAGAAGCTCGAGATCCCGTTGCCGCCTCTTGCCCGTGGCGAGGACATG

GGCGGTGCTAGTGATAAGGGTGTGCGTCTGGCCAAATCCTTGAAGGAGGCTATTGTTACA

TTTACACGGCCGGAGAGTGTCGCACGGGAAGTCTTTGGCGACTCATTCGTCGACCACTTT

GCCGGAACTCGGGAACACGAGGTGCGCTTGTGGGAAGAGGCCGTCACTGATTGGTATGTT

GTATTATCAACTGCGATATTCTTTTGGATGCTGACTTGACCTGTTCACAGGGAGATGAGG

CGGTACATCGAGACCGTG

>CL18.Contig8_All 1462 3054 Glutamine synthetase, putative [Penicillium digitatum PHI26] >gi|425782516|gb|EKV20422.1| Glutamine synthetase, putative [Penicillium digitatum Pd1]

ATGTCGAGCCCCGCGGACCTCGAGGTCACAGCCGAGAATGTTGCTGAGATCCTCCAGAAT

GACACAAAAGTCAAGGTGGCAGGAGTGGATGTGGATGGCATTCTGCGTGGCAAACTCATG

AAGAAAGACAAGTTCTTATCTATCATCACCGAGGGTTTCGGCTTCTGCTCTGTCATCTTC

GGCTGGGATCAACACGATGCGACGTACTATAAGGAGCTGGCCATCAGCAACAAGGAGAAT

GGATATCGCGATCTTGTCGCAGTCCCAGATTTACGCAGTTTCCGTCGCATTCCATGGGAA

AACAATGTGCCCTTTTTCCTCATCAGCTTCTTGGATCCAGAAACCAGGAAGCCGGTGTGC

GCCTGCCCGCGAGGATTGATAAAGACTGCGGCCGCTAAAGTCGAGGCGGCAGGGTATCGG

GCGATGGCAGGAGCCGAATACGAATTCTACCAATTTCGCACGCCAGGCAATCACTCCACC

CCCGAGCAAGGCGCATCTGCAACGGCGGCCTTTCTCCAGTCGAATCCAGTCGAATCGTTG

CCATCTGTCACAGACGGCATGTTTGGTTATTCAATCACTCGGCCTCTCCATAACCAAGAT

TACTACTATGGCATCTTCGACGCCTGCGAGCAGTTCCGATGCGATATTGAAGGGTGGCAT

ACTGAAAGTGGACCAGGAGTCTATGAAGCTGCATTGCAATTTGGCGAAGCGAAAGACATG

GCTGACAAGGCCGGGTTATTCAAGTGAGAGCGTTCTTTTGCCAGTGGTTCGGGTTGTTCT

TTCATGATGGGTCATCGCTAACCAGGACGCTCGCATAGATACGTCGTAAAAGCATTCGGA

ATTAAGCATGGCATCACCCCATGCTTCATGGCGAAACCCCGACAAGGCCTACCAGGAAAC

AGCGGGCACATGCACATCTCGCTCGTCACAGCCGACGGAAAGAATGCCTTCATTCGCGAT

GCTCCGGATCCATCCCCCCTCTACCCTGATATCACTCATCTGTCCGACTTGGGTCGACAT

TTTCTCGCTGGCCTTCTCGTCGGCCTGCCCGATATCATGCCAATTTTGGCACCGACGATC

AACTCCTACAAACGCCTGGTGGAGAACTTTTGGGCACCGGTCACTGTTTCTTGGGGACTA

GAACACCGGGCCGCGTCAATCCGTCTGATCACCCCACCGACAGCTAGTCCGAAAGCGACT

CGTTTCGAGGTGCGCGTGCCAGGTGCAGATACCAATGCGCATCTTGTCTTAGCGGCGATA

CTGGCATTGGGATGGCGCGGAGTCGAGAAGAAGCTCGAGATCCCGTTGCCGCCTCTTGCC

CGTGGCGAGGACATGGGCGGTGCTAGTGATAAGGGTGTGCGTCTGGCCAAATCCTTGAAG

GAGGCTATTGTTACATTTACACGGCCGGAGAGTGTCGCACGGGAAGTCTTTGGCGACTCA

TTCGTCGACCACTTTGCCGGAACTCGGGAACACGAGGTGCGCTTGTGGGAAGAGGCCGTC

ACTGATTGGTATGTTGTATTATCAACTGCGATATTCTTTTGGATGCTGACTTGACCTGTT

CACAGGGAGATGAGGCGGTACATCGAGACCGTG

>CL18.Contig9_All 1535 3070 Glutamine synthetase, putative [Penicillium digitatum PHI26] >gi|425782516|gb|EKV20422.1| Glutamine synthetase, putative [Penicillium digitatum Pd1]

ATGTCGAGCCCCGCGGACCTCGAGGTCACAGCCGAGAATGTTGCTGAGATCCTCCAGAAT

GACACAAAAGTCAAGGTGGCAGGAGTGGATGTGGATGGCATTCTGCGTGGCAAACTCATG

AAGAAAGACAAGTTCTTATCTATCATCACCGAGGGTTTCGGCTTCTGCTCTGTCATCTTC

GGCTGGGATCAACACGATGCGACGTACTATAAGGAGCTGGCCATCAGCAACAAGGAGAAT

GGATATCGCGATCTTGTCGCAGTCCCAGATTTACGCAGTTTCCGTCGCATTCCATGGGAA

AACAATGTGCCCTTTTTCCTCATCAGCTTCTTGGATCCAGAAACCAGGAAGCCGGTGTGC

GCCTGCCCGCGAGGATTGATAAAGACTGCGGCCGCTAAAGTCGAGGCGGCAGGGTATCGG

GCGATGGCAGGAGCCGAATACGAATTCTACCAATTTCGCACGCCAGGCAATCACTCCACC

CCCGAGCAAGGCGCATCTGCAACGGCGGCCTTTCTCCAGTCGAATCCAGTCGAATCGTTG

CCATCTGTCACAGACGGCATGTTTGGTTATTCAATCACTCGGCCTCTCCATAACCAAGAT

TACTACTATGGCATCTTCGACGCCTGCGAGCAGTTCCGATGCGATATTGAAGGGTGGCAT

ACTGAAAGTGGACCAGGAGTCTATGAAGCTGCATTGCAATTTGGCGAAGCGAAAGACATG

GCTGACAAGGCCGGGTTATTCAAGTGAGAGCGTTCTTTTGCCAGTGGTTCGGGTTGTTCT

TTCATGATGGGTCATCGCTAACCAGGACGCTCGCATAGATACGTCGTAAAAGCATTCGGA

ATTAAGCATGGCATCACCCCATGCTTCATGGCGAAACCCCGACAAGGCCTACCAGGAAAC

AGCGGGCACATGCACATCTCGCTCGTCACAGCCGACGGAAAGAATGCCTTCATTCGCGAT

GCTCCGGATCCATCCCCCCTCTACCCTGATATCACTCATCTGTCCGACTTGGGTCGACAT

TTTCTCGCTGGCCTTCTCGTCGGCCTGCCCGATATCATGCCAATTTTGGCACCGACGATC

AACTCCTACAAACGCCTGGTGGAGAACTTTTGGGCACCGGTCACTGTTTCTTGGGGACTA

GAACACCGGGCCGCGTCAATCCGTCTGATCACCCCACCGACAGCTAGTCCGAAAGCGACT

CGTTTCGAGGTGCGCGTGCCAGGTGCAGATACCAATGCGCATCTTGTCTTAGCGGCGATA

CTGGCATTGGGATGGCGCGGAGTCGAGAAGAAGCTCGAGATCCCGTTGCCGCCTCTTGCC

CGTGGCGAGGACATGGGCGGTGCTAGTGATAAGGGTGTGCGTCTGGCCAAATCCTTGAAG

GAGGCTATTGTTACATTTACACGGCCGGAGAGTGTCGCACGGGAAGTCTTTGGCGACTCA

TTCGTCGACCACTTTGCCGGAACTCGGGAACACGAGGTGCGCTTGTGGGAAGAGGCCGTC

ACTGATTGGGAGATGAGGCGGTACATCGAGACCGTG

>CL18.Contig10_All 1512 2972 Glutamine synthetase, putative [Penicillium digitatum PHI26] >gi|425782516|gb|EKV20422.1| Glutamine synthetase, putative [Penicillium digitatum Pd1]

ATGTCGAGCCCCGCGGACCTCGAGGTCACAGCCGAGAATGTTGCTGAGATCCTCCAGAAT

GACACAAAAGTCAAGGTGGCAGGAGTGGATGTGGATGGCATTCTGCGTGGCAAACTCATG

AAGAAAGACAAGTTCTTATCTATCATCACCGAGGGTTTCGGCTTCTGCTCTGTCATCTTC

GGCTGGGATCAACACGATGCGACGTACTATAAGGAGCTGGCCATCAGCAACAAGGAGAAT

GGATATCGCGATCTTGTCGCAGTCCCAGATTTACGCAGTTTCCGTCGCATTCCATGGGAA

AACAATGTGCCCTTTTTCCTCATCAGCTTCTTGGATCCAGAAACCAGGAAGCCGGTGTGC

GCCTGCCCGCGAGGATTGATAAAGACTGCGGCCGCTAAAGTCGAGGCGGCAGGGTATCGG

GCGATGGCAGGAGCCGAATACGAATTCTACCAATTTCGCACGCCAGGCAATCACTCCACC

CCCGAGCAAGGCGCATCTGCAACGGCGGCCTTTCTCCAGTCGAATCCAGTCGAATCGTTG

CCATCTGTCACAGACGGCATGTTTGGTTATTCAATCACTCGGCCTCTCCATAACCAAGAT

TACTACTATGGCATCTTCGACGCCTGCGAGCAGTTCCGATGCGATATTGAAGGGTGGCAT

ACTGAAAGTGGACCAGGAGTCTATGAAGCTGCATTGCAATTTGGCGAAGCGAAAGACATG

GCTGACAAGGCCGGGTTATTCAAATACGTCGTAAAAGCATTCGGAATTAAGCATGGCATC

ACCCCATGCTTCATGGCGAAACCCCGACAAGGCCTACCAGGAAACAGCGGGCACATGCAC

ATCTCGCTCGTCACAGCCGACGGAAAGAATGCCTTCATTCGCGATGCTCCGGATCCATCC

CCCCTCTACCCTGATATCACTCATCTGTCCGACTTGGGTCGACATTTTCTCGCTGGCCTT

CTCGTCGGCCTGCCCGATATCATGCCAATTTTGGCACCGACGATCAACTCCTACAAACGC

CTGGTGGAGAACTTTTGGGCACCGGTCACTGTTTCTTGGGGACTAGAACACCGGGCCGCG

TCAATCCGTCTGATCACCCCACCGACAGCTAGTCCGAAAGCGACTCGTTTCGAGGTGCGC

GTGCCAGGTGCAGATACCAATGCGCATCTTGTCTTAGCGGCGATACTGGCATTGGGATGG

CGCGGAGTCGAGAAGAAGCTCGAGATCCCGTTGCCGCCTCTTGCCCGTGGCGAGGACATG

GGCGGTGCTAGTGATAAGGGTGTGCGTCTGGCCAAATCCTTGAAGGAGGCTATTGTTACA

TTTACACGGCCGGAGAGTGTCGCACGGGAAGTCTTTGGCGACTCATTCGTCGACCACTTT

GCCGGAACTCGGGAACACGAGGTGCGCTTGTGGGAAGAGGCCGTCACTGATTGGGAGATG

AGGCGGTACATCGAGACCGTG

>CL18.Contig11_All 121 921 minus strand Short-chain dehydrogenase/reductase family protein, putative [Penicillium digitatum PHI26] >gi|425782515|gb|EKV20421.1| Short-chain dehydrogenase/reductase family protein, putative [Penicillium digitatum Pd1]

ATGGCTGCCACTACTCCCCAAGGACGTCTCCAAGGCAAGAATGCCATCATCACAGGTGCA

GGCGGTGGCATCGGTCTCGAAACCAGTATTCTCTTCGCCCGCGAGGGTGCCAATGTCCTG

ATGGCCGACGTCTCTGAGCCCGCCCTTGAAAAGGCATTGGCTAAAGTGAAGGAAGTAGTT

CCCTCCGCTCCTCGAGTCGAGTCTATCCGATGTGACGTGTCCAAAGAGTCGGATGTCCAG

GCAATGGTTGAGTACCAGGACAGCTGGGGAGGCACTGACGTGATCTTCAACAATGCCGGA

ATCATGCACGCCAACGATGCAGATGCCATCGATACCCCCGAGAAGATCTGGGACCTCACA

CACAATATCAATGTCAAGGGTGTCTGGTTCGGCAGCAAGCACGCCGTCCTCAGTCTTCGT

CGTCACAAGAAGTCCCGGGGCAGTATTATCAACACAGCTAGTGTGGTCGCCTTGGTCGGC

GCTGCTACTCCTCAGCTCGCGTATACGGCGAGCAAGGGTGCGGTCTTGGCGATGACTCGG

GAATTGGCCATCGTTCACGCCCGTGAGGGATTCCGTTTCAACGCTCTGTGCCCCGCACCC

CTGAACACTCCTCTTTTACAAGACTGGCTGGGTGATGACAAGGCCAAGCGGCTCCGTCGG

GAGGTGCACTTCCCCACTGGCCGGTTTGGTGAACCCATCGAGCAGGCTCACGCAGTGGTT

TTCCTGGCCAGCGATGAGAGCAGCTTCGTGAATGGAACGGACTTTGTGGTCGATGGTGGA

ATGACTAAAGTAGGTGTGACG

>CL18.Contig12_All 774 971 minus strand Short-chain dehydrogenase/reductase family protein, putative [Penicillium digitatum PHI26] >gi|425782515|gb|EKV20421.1| Short-chain dehydrogenase/reductase family protein, putative [Penicillium digitatum Pd1]

AGCACTCCTCTTTTACAAGACTGGCTGGGTGATGACAAGGCCAAGCGGCTCCGTCGGGAG

GTGCACTTCCCCACTGGCCGGTTTGGTGAACCCATCGAGCAGGCTCACGCAGTGGTTTTC

CTGGCCAGCGATGAGAGCAGCTTCGTGAATGGAACGGACTTTGTGGTCGATGGTGGAATG

ACTAAAGTAGGTGTGACG

>CL18.Contig13_All 242 799 minus strand 2-keto-3-deoxy-L-fuconate dehydrogenase OS=Xanthomonas campestris pv. campestris (strain ATCC 33913 / NCPPB 528 / LMG 568) GN=XCC4067 PE=1 SV=1

ATCTCGGAATTGGCTAGTGGCATCGGTCTCGAAACCAGTATTCTCTTCGCCCGCGAGGGT

GCCAATGTCCTGATGGCCGACGTCTCTGAGCCCGCCCTTGAAAAGGCATTGGCTAAAGTG

AAGGAAGTAGTTCCCTCCGCTCCTCGAGTCGAGTCTATCCGATGTGACGTGTCCAAAGAG

TCGGATGTCCAGGCAATGGTTGAGTACCAGGACAGCTGGGGAGGCACTGACGTGATCTTC

AACAATGCCGGAATCATGCACGCCAACGATGCAGATGCCATCGATACCCCCGAGAAGATC

TGGGACCTCACACACAATATCAATGTCAAGGGTGTCTGGTTCGGCAGCAAGCACGCCGTC

CTCAGTCTTCGTCGTCACAAGAAGTCCCGGGGCAGTATTATCAACACAGCTAGTGTGGTC

GCCTTGGTCGGCGCTGCTACTCCTCAGCTCGCGTATACGGCGAGCAAGGGTGCGGTCTTG

GCGATGACTCGGGAATTGGCCATCGTTCACGCCCGTGAGGGATTCCGTTTCAACGCTCTG

TGCCCCGCACCCCTGAAG

>CL18.Contig14_All 242 976 minus strand Uncharacterized oxidoreductase YxbG OS=Bacillus subtilis (strain 168) GN=yxbG PE=3 SV=2

ATCTCGGAATTGGCTAGTGGCATCGGTCTCGAAACCAGTATTCTCTTCGCCCGCGAGGGT

GCCAATGTCCTGATGGCCGACGTCTCTGAGCCCGCCCTTGAAAAGGCATTGGCTAAAGTG

AAGGAAGTAGTTCCCTCCGCTCCTCGAGTCGAGTCTATCCGATGTGACGTGTCCAAAGAG

TCGGATGTCCAGGCAATGGTTGAGTACCAGGACAGCTGGGGAGGCACTGACGTGATCTTC

AACAATGCCGGAATCATGCACGCCAACGATGCAGATGCCATCGATACCCCCGAGAAGATC

TGGGACCTCACACACAATATCAATGTCAAGGGTGTCTGGTTCGGCAGCAAGCACGCCGTC

CTCAGTCTTCGTCGTCACAAGAAGTCCCGGGGCAGTATTATCAACACAGCTAGTGTGGTC

GCCTTGGTCGGCGCTGCTACTCCTCAGCTCGCGTATACGGCGAGCAAGGGTGCGGTCTTG

GCGATGACTCGGGAATTGGCCATCGTTCACGCCCGTGAGGGATTCCGTTTCAACGCTCTG

TGCCCCGCACCCCTGAACACTCCTCTTTTACAAGACTGGCTGGGTGATGACAAGGCCAAG

CGGCTCCGTCGGGAGGTGCACTTCCCCACTGGCCGGTTTGGTGAACCCATCGAGCAGGCT

CACGCAGTGGTTTTCCTGGCCAGCGATGAGAGCAGCTTCGTGAATGGAACGGACTTTGTG

GTCGATGGTGGAATG

>CL19.Contig1_All 56 262 minus strand hypothetical protein PDIP_52610 [Penicillium digitatum Pd1] >gi|425776200|gb|EKV14429.1| hypothetical protein PDIG_33050 [Penicillium digitatum PHI26]

ATGAGTACTCCAGTTACAGCTCCATTCGGCCAATGGAAGAGTCCGATCAGCTCTACCTTG

CTTGGCGCTGACGGTGTTCAATTTGAATCCATCGCGACATCCAAGGGAAAGGTTTATGTA

ATTGAGGATCGACCCAAAGAGCAGGGACGTGGCTGCATTGTCGAATACGCTGGCCATGAA

GGACGAGATATCTTACCAGCCAAGTAC

>CL19.Contig2_All 84 2066 minus strand hypothetical protein PDIP_52610 [Penicillium digitatum Pd1] >gi|425776200|gb|EKV14429.1| hypothetical protein PDIG_33050 [Penicillium digitatum PHI26]

ATGAGTACTCCAGTTACAGCTCCATTCGGCCAATGGAAGAGTCCGATCAGCTCTACCTTG

CTTGGCGCTGACGGTGTTCAATTTGAATCCATCGCGACATCCAAGGGAAAGGTTTATGTA

ATTGAGGATCGACCCAAAGAGCAGGGACGTGGCTGCATTGTCGAATACGCTGGCCATGAA

GGACGAGATATCTTACCAGCCAAATATGACGCTCGCACCAAAGTTCATGAATATGGCGGC

GCATCGATGATCGCTTTCGATGGACATGTCGTCTTTTCTGATCGGGAGACACAGGATCTG

CATAAACTCGATCCCTCCACAGGTCAAGTTGAGCAGATCACAAAGACAAGCAATGCTCTC

CGCTATGCGTCCACCTCTGCTACGGGATCTCTGTCAGATGGCCAGGCGGAGTCAGGCTGG

ATCTTAGCGATCGAAGAAGATCACTCCAAACCCTTACCGTCGGAAGTTCGGAACCGACTC

GTCGCGGTAAACGTACAGTCCAAAGAGATTGTCAACGTCGCCTCTGGGGATGATTTCTAC

AACGCTGCTCAGTTTTCCCCTGATGGCAGCAGGATATGCTGGACCCAGTGGTCTCATCCA

GATATGCCATGGACAGGAGCCCGCCTGTATGTGGCCAAGTGGAATAATGGTCAGGTTACA

GACATTTGCCATGTGTCGGGTGTGCCGGAGAAAGAAAGCGTTAGCCAGCCACGATGGGGC

CTTGATAACAAGTTGTACTTCACGAGTGATTGTTCGGGGTATTGGCAGCTCTATCGGTCT

CATGCTGACACTTTGGAGTGTCAACGGGTGCCCCTACATGGTCTGGAAGAAGTGGAGTTT

TCGCAGCCAGACTGGCATCTAGGAAATTGCACCTACGTGTGTTTGACTCCAACTTCAATG

ATCGCGTCGTACACAAAGAATGGTAGATGGAGTTTCATATTGATTGATCTCTCGAACGAA

TCCTGGCAGGAAATGAATCTGCCAGTGACAGACACGATTGTTTTGGCAGATACTCCTCTC

TCAGATACAAAAATAGCGCTTCTTGGATCTAGCGAGACGAGCTTCAACACTGTTTTTACA

TTGGAAATGGCCGACACCGTCGAGCTCGATGCACTCAAGGTAGCTTCTGAGCTCCCCATG

CCAAGTTCTCTTGTTTCCAAGCCAGAGCATCTATCTTTTCCAAGAGTGCACGGTGAGAAC

TTGGAAGGAGTGGCACATGCTATCTTCTACGCACCTCAGAACCCAGATTATCAGCCACCT

TCTGGAGCGCTGCCGCCTCTGATCGTCTGTGTCCATGGTGGACCAACGTCCCAAACCGGA

ACTGGCCTGAATATTACGAACCAATACTGGACTTCTCGAGGGTATGCCGTTGTTTGGGTA

AATTATGGAGGTAGCTCCGGATATGGCAGAGCGTACCGGGATGATTTGAACGGACAATGG

GGGATCCTTGACACAGCAGATGCTGCTAGCTGTGTGTCATACTTAGCGTCCACTGGCCGA

ATCGACCAAAATGGAGTCGGCATTCGCGGCCAAAGTGCGGGCGGATACATCGTACTCCAA

GCTCTTTGTGACTACCCCAATCTCTTCGCCGGTGGCAATTCTCTTTACGGAATCGGCAAT

GTCAAGGCTCTCTGTGAGGATACCCATAAGTTTGAGAGCCATTACGCATTCGCCTTGCTG

TTTGATCCAGGAGTGGATGAAGACGAGAAAACCCGCATTTTCAACGAACGCAGCCCCTGT

CTCAAGGTAGACAAGATCACTGCGCCATTGTTGTTGCTGCAAGGAGACGAGGATTTGGTC

GTTCCTATGAACCAGGCAGAGGAAATGGTCGAAATGATGACAAAGGTTGGTCGTGAATCT

AAGTTGGTGGTTTTCCATGGAGAAGGGCATGGATTCCGGCAGGCGAAGAGTCGGATAGCG

GCCGTGGAAGAGGAGGAGAAGTGGTGGAAGAAAGAGTTGTTGAAGATGGATTCGGTGGAT

CTT

>CL20.Contig1_All 195 2075 Transporter, putative [Penicillium digitatum PHI26] >gi|425781801|gb|EKV19746.1| Transporter, putative [Penicillium digitatum Pd1]

ATGTCAAGGGTCGAAAGAACAGATGAAGATAACGACGGTGAACCGGTCCATGGCTCACCC

AGGATACCGGCTGCAACACCAATCTCCTGCGGTTATGCCCATCTAGAGCGTGAGAATCCG

ACCGCCGAGTCGGAGATCGGCGGTAGACAGAGATCGTCATCGTACTCGATTCGAGTCCGC

CAAGCGGGGGGTCCCAACAGTATAGACAATTTCGCGCGGTCTTGGCAGAGAGCTGCGGCC

TTCCCAGAAGTCCTCCCACGGCGGTCGTCGTTTGTCTCTGCGCATTCGGACGATGACTTT

GCCGTTGCAACTGGTCAAGAACATGGAACAAGAAGTTTTCCATCATGGGGTCGGAATTCG

GATGTGGACCGGCCTCTGCTTCGCAGGGACTCGGATTTGGACCAGGATGATGAAGACTCA

CGAGCGCTGGAATCTTCCAAGAAGGCACTTCCTAGTACTGGGCTGCTTGCTTCGTCATTT

GATAGAACATTTGCGACTTCGTATGGCACTATCTCATCACGAATGAGCGAATCGACGCGG

AGGAATGCGATTCAATTCCACCGAGAGCACCATCCCCATGTGCATGTGGATGGGTCTGGA

GATTTGGATCGGGGCCCATTGCTTGTCAAGTATGTCCAACATGAAGATGGGACCAAGGAG

GATTTCATCGTTGGGCAATCAACTCTTCCCCAGACCATTTTCAACTCTGTCAATGTGTTG

ATTGGCATCGGCCTGCTGAGTCTTCCATTGGCAATGAAGCATGCTGGGTGGGTGCTGGGA

TTGTTATTCCTTATCTTCTCGGCGGTCACCACTTCCTACACAGCCAAGATTCTGGCTAAA

TGTCTGGATGTCGATCAGAGCGTTGTCACTTATGCTGATCTGGCCTACATAAGTTTCGGG

CAAAATGCCCGTTTGATAACAAGTTTCTTGTTCTGCTTAGAACTTTTAGGAGCATGCGTG

GCGTTGGTTGTGCTCTTTGCAGACAGCCTATATGCCCTGATACCAGGATTTAGCATCCTC

CGATGGAAGATCGTCTGTGGTGTTGTGCTAGTTCCGCTCAACTTCCTTCCGCTACGATTC

CTGAGTATCACCAGTATATTGGGTATAATTTCATGCACATCAATTGTGGTACTCATTTGC

ATCGATGGCTTTGTCAAGCCAGATGCACCGGGGTCCTTGCGGCAGCCAGCAAATACGTTC

CTCTTTCCTGAAAATTGGGCTACCCTTCCTTTAAGTTTTGGTCTTATTATGTCACCATGG

GGTGGTCACGGTGTCTTCCCTAACATCTACAGAGATATGAGGCACCCTCAGAAATACGGG

AAGAGTCTCTGGGTGACCTATCTTTTTACATTTGCTTTGGACTGCTCAATGGCGATCATT

GGTTGGCTCATGTTTGGCGATATCGTTCGCGACGAAATCACGGCGAACATTCTCACCATC

ACCAGCTACCCACAGTCCTTATCTGTCTGTATTGTAGTCTTCATTTCGATCATCCCGCTC

ACGAAAGTGCCGTTGAATGCCCGGCCTCTTGTGGCCACATTTGAAGTTCTTTGTGGACTA

GGATCCGGCCCTGTGCCCGGAAATGGATCAGAGACAATGCAAAAGTTTTCACGGGCAATG

GTCCGCGTTTTTGTGGTGGCCACGATTGTTTTTCTGGCGGTTATCTTCCCGGCTTTCGAC

CGTATCATGGCTTTCTTGGGATCATTCTTGTGCTTCACGATCTGTATCATCTTCCCTCTT

GCATTTTATATCAAGATTTTTGGAAAGGAGATCAGTCGAGGCGAGTACATCCTTGATTGG

ATTCTGCTCATCATCTCCTCGATATTGGCAGCCGTGGGGACCGTCTGGGCCTTCTTGCCA

CAAGACATGCTCTCTGCAAAC

>CL20.Contig2_All 1760 2125 minus strand Transporter, putative [Penicillium digitatum PHI26] >gi|425781801|gb|EKV19746.1| Transporter, putative [Penicillium digitatum Pd1]

AGTGCCCGGCCTCTTGTGGCCACATTTGAAGTTCTTTGTGGACTAGGATCCGGCCCTGTG

CCCGGAAATGGATCAGAGACAATGCAAAAGTTTTCACGGGCAATGGTCCGCGTTTTTGTG

GTGGCCACGATTGTTTTTCTGGCGGTTATCTTCCCGGCTTTCGACCGTATCATGGCTTTC

TTGGGATCATTCTTGTGCTTCACGATCTGTATCATCTTCCCTCTTGCATTTTATATCAAG

ATTTTTGGAAAGGAGATCAGTCGAGGCGAGTACATCCTTGATTGGATTCTGCTCATCATC

TCCTCGATATTGGCAGCCGTGGGGACCGTCTGGGCCTTCTTGCCACAAGACATGCTCTCT

GCAAAC

>CL22.Contig1_All 155 1750 minus strand hypothetical protein PDIG_18970 [Penicillium digitatum PHI26] >gi|425784146|gb|EKV21940.1| hypothetical protein PDIP_01650 [Penicillium digitatum Pd1]

ATGGGCCAATTTCATTCCACTAGTCAGTCTAGCTCGAGCGCTTTGGAAAAATTAGACCAG

AAGACCGACTATTACGAGCTGCTTGGTGTAACCCGCGATGCAACAAATGATGAGATCAAA

AAAGCATATCGAAGAAAAGCTCTCGTATTGCACCCAGATCGAAATTATGGGAATGTTGAT

GAGGCTACCAAGCTGTTTGCCGAAATCCAATCTGCCTACGAAGTACTGGCAGATCCTCAG

GAACGAGCATGGTACGACTCCCATAGCGATGCATTTTTAGGGACCAACGGAAATACCGAT

GATCAACACTCGTACAACGTTCGGATTACAACAGCCGAAGATGTCCTCAGACTCTTTTCG

AAGTTCAGTCCTCGAATGGAATTCTCTGATTCCCCAACCGGATTCTTTGGTGGTTTGCGA

GAGCAATTCGAACAGCTCGTGCTGGAAGAAAGATTGGCCTGTCGGTGGGAGAATCAAGAT

CCTGTTGAATACCCATCATTCGGATCTGGTAACGATGATTTCGAGACCGTTGTACGGCCC

TTCTATGCTACCTGGACCGGATTCTCCACCCAAAAGTCGTTCGCATGGAAAGATGCTCAT

CGTTATTCTGAAGCGCCTGATCGTCGGGTGCGGCGAATGATGGAAAGAGAGAACAGGCGT

CTTCGGGAGGAAGGCATCCGAGAATTCAACGATGCTGTCAGGTCCCTCGTAGCTTTTGTC

AAGAAACGTGATCCGCGGTATAAAGTCAATGCCCAAAGTGAAGCTCAACGCCAAGAAACC

CTCCGCCAGTCTGTGGCTGCACAGGCTGCAAGATCACGAGCTGTGAATCAAGCTAAAATG

CGGGATCATATCCTCCCCGAGTGGGCCCAATCAGAGCAACCAGTGGCAGATGACGACCAG

GAGAGCAGCGAAGAAAGTGAAGTCGAGAGTTTTGAATGTGTGGCATGTCACAAGTATTTC

AAAAGCCAGAAGCAGTTCGAGGCACACGAACGTAGCAAGAAGCACTTGAAGGCAGTCAAA

CAGCTGTGCCGGGAGATGCGAATGCAGAACCACGAGTTGGATTTGGAATCCGCTGATGAA

TCGAGTATAAATGCAGTTACAACTAACCCTCTCGGGGTCGATGACGGGAAAGTTGAGGTT

TCTGCTGCCTCAGATGTCGAACTGGAAGTAAATGCCTCTGTGCAGAGTCACGACGTTCCC

GCTGATGACCGAAAAAGTTCGCATGGTCTAGAGGAAACAACTCCCCTTTCCGAGCCAGAT

ACCCCAACGTCTTCGCACGACGACGATTATGCCTCCAGGGAATATGTTGAGACGAGGCTC

CGCACTGATATGGATCATTTGTCCACAGGTGCTGGAGATTCTTCTGGTCATCCGCTCTTT

TCGGGCCTCACTGGGGAGTCACCAACTCCCAAATTGGGCAAGGCCAAACAGAAACGGGCG

AAAAGGGCCGCCAAGCAAGCGGATCAACCCATGGCCTTCATTTGTGCGAATTGCCAGGCT

CACTTCGCGTCCAAGACTAAACTATTCGATCATCTTAGAGACCTTGACCACGCAGAACCC

TTGTTGAGGTCTATTGCTAAAAAGGGGAGGAAACAC

>CL22.Contig2_All 152 265 hypothetical protein PDIG_18970 [Penicillium digitatum PHI26] >gi|425784146|gb|EKV21940.1| hypothetical protein PDIP_01650 [Penicillium digitatum Pd1]

ATGGGCCAATTTCATTCCACTAGTCAGTCTAGCTCGAGCGCTTTGGAAAAATTAGACCAG

AAGACCGACTATTACGAGCTGCTTGGTGTAACCCGCGATGCAACAAATGATGAG

>CL23.Contig1_All 604 771 minus strand Rho-like small GTPase, putative [Penicillium digitatum PHI26] >gi|425781471|gb|EKV19435.1| Rho-like small GTPase, putative [Penicillium digitatum Pd1]

GAGAAGGGCTATCGTATTGCACAGGAACTTCGTTGCGATAGATACGCCGAGTGTTCGGCT

GTCACAGGCGAGCTGCTTGTAGAGACGTTTGAAGATCTTGCTAGATTGGCGGCAATGACG

ACGACTGAAAAAGGTGGTCAGTCTGAGGGCACCTCTTGCGTTGTTATG

>CL23.Contig2_All 183 797 minus strand Rho-like small GTPase, putative [Penicillium digitatum PHI26] >gi|425781471|gb|EKV19435.1| Rho-like small GTPase, putative [Penicillium digitatum Pd1]

ATGGCCGAGGCAGATCCATACCCGGAGAGTAACCACTCAGTCTCTCTACTTCTACTTGGA

GATCCAGGATGTGGGAAATCAACATTCTTATCTCGCCTGAAGAGTGGAAGACCACTCCCA

TCAGGGCCATCTAATACTGACGCAAATACCCTTGAACTATTACGAGATGGTGATCAGCCT

TTCATCTACGACATCAGGTTCTCAAAAAAGACATTCACTTTAGAGCTTTACGACACATCC

AACCCAAACCAGCACTGGACCAATCTTCGACCCGATGTCGCCGTTCTAGCCTATGATATT

TCCAATCGAAACACACTTGCTGGACTAAAAGAGTGGCGAAATGACATTACGCGCTACTTC

CAATACGGTCATAGCGAGCGGCTTCCAGTGATGATGATAGGCCTGAAAAGGGATCTGCGG

AGAGAAGGTGAGGGCATCATCTATCCACAAGAAGGCTATCGTATTGCACAGGAACTTCGT

TGCGATAGATACGCCGAGTGTTCGGCTGTCACAGGCGAGCTGCTTGTAGAGACGTTTGAA

GATCTTGCTAGATTGGCGGCAATGACGACGACTGAAAAAGGTGGTCAGTCTGAGGGCACC

TCTTGCGTTGTTATG

>CL24.Contig1_All 166 1344 minus strand Protein fyv10 OS=Aspergillus terreus (strain NIH 2624 / FGSC A1156) GN=fyv10 PE=3 SV=2

AATGTACTTTTACAGGACCAACCACTCCTTCGACTACCACATGAACTAGCCCGCAGAAAT

TTGAAAACTGTACAACGAGCTGTCGAGCGAGAGAAAGAATATGTCATCCCTGCAATCAAA

GAGGCCGCCGCCGCCTCACTCTCCAACACCCAAACACCCGACCAGACCCTCGCTGCACTC

GACGCAATTATATCCCGAATGCAAGGCCTCAAAAGGAAAATGGAAAGTCTTCAAGAGGAA

GAGAAGAAAATTCACAATCAATCTCGCAAGCGCATTCAGCACTTGGAGACCCTGTATAAT

ATCCCAAGCCTAGCCGATGTCAAGTACGATCAGTGGTCAAGGATACGGCTCGACCGCTTA

CTCGTTGATCATATGCTGCGCTCAGGTTACTCCGAAAGTGCGAAGCAGCTGGCCCAGGTT

AGGGGAGTGGAGGATCTTGTGGATGTCGGCGTGTTCACGCAGTGTCAACGGGTTGTGGAT

AGCTTGCGGCGCGGGGAAACCAAGGAGGCATTGCAATGGTGTGGAGAGAATAAGGCTGCA

CTGAAGAAGAGCCAGCATAATCTTGAATTCGAACTCCGGTTGCAGCAATATATTGAGATG

GTCAGGACACAAGACAAGTCCAAGAAGATTGAGGCAATAATTCATGCAAAGAAATACCTC

ATACCGAACCATCAATCCCAGAATTCTGAGATTATGCGAGCTGCAGGGCTACTAGTTTTC

ACACAAGACACAAGAGCCGAGCCTTACAAATCTCTCTTTTCAGTGGACCGCTGGAGGTAT

CTCGTGCAGCTTTTCATTCAAACTCACCATGAGCTGCTTTCATTGCCATCACAACCATTG

TTACATATTGCACTTTCTGCAGGACTTTCAGCCCTTAAGACACCTCTGTGTCATTCAGCA

TATACATCGTCTAGCTCAAACTCACAGTCAACGTCGACATCTGTGTGCCCTATCTGCTCA

ACCGAACTGAACGAGCTTGCACGCAAAATGCCGTACGCCCACCACTCAAAGAGTTATGTG

GAAAGCGACCCAATCGTTCTACCAAATGGCCGAGTCTACGGAAAGCAAAGACTGATAGAG

ATTAGCCAGAAGATGGGATCAGTGGAATCAGGCAACGTCAAAGATCCAACAACAGGCCAG

GTTTTCCACGAGAGCGAAATGAAGAAAGTGTACATATTG

>CL24.Contig2_All 1 1128 hypothetical protein PDIG_84740 [Penicillium digitatum PHI26] >gi|425781698|gb|EKV19645.1| hypothetical protein PDIP_22400 [Penicillium digitatum Pd1]

GGCCCATCTTCCAATCCTACCAAGGGCCAGCAGCATGGCAATGGGTATAAGCAGGGTAGT

GGCAATGCCAATGCACCTAAGAAAGCTTCGATGCAAGTAAACCAGAGGCCTATCGTGCCA

TTTTTAAGGAAGGATCGAGTTCTCTTGATTGGTGAAGGTGATTTCTCATTCGCCCGTTCT

CTGGCAAAGCAATACAAGTGTCGCAACCTATGCGCCACATGCTACGATTCCAAGGAAGCA

CTGTACAACAAGTACCCGCAAGCTCCTCAGAACGTCTTAGATATCCAGAATGCATCAGCA

AACCCAACAAGCGATGACACCGAAAACCAACCAGAAGAGAGTAAATCCGAAGAGCAAGAC

TCAACCAAGCCCAACCCCAATCCCAATCCCAACCAGCAAACCCCCAAAGTCATCTTCTCC

GTGGACGCTCGCAAACTCGGCACTCCAGCAGGCGGCGGCAAGGAGATCCGCACAGGGTTC

GCACGCCGCGAGCGCAAGAGACCAGCTTGGTACCAGCAGAATGAGCCAGCAGGACCACCC

TACCAGCTGGGCGGGCCGTGGGATGTGATCTGCTTCAATTTCCCGCATGTTGGCGGGCTC

TCCACGGACGTGAACCGACAGGTTCGGGCGAATCAAGAGCTGCTGGTTGCGTTCTTCAAG

GCGTGTGTGCCTCTTATGTCGAAGCCGCCGCCGCTTATGGATGCTGATGATGATGAGTGG

GTGTATGCGGATGGGGAGGAGAGTGAAGAAGATGAGGACGAGGATGGAGGAGACGGCGAG

GAGCTCGGGAAAGATGATGATACTGCCGGGAAGGGGTTCCGGGTTGGCCCTGGCCAAATT

CTTGTGACTCTGTTTGAAGGGGAGCCGTATACGCTTTGGAATATTAGGGATTTGGCGAGG

CATGCGGGACTGGTTGTTGTGACTAGTTTTCGGTTTCCTTGGACTTCTTATGAGGGGTAT

TCGCATGCAAGAACGGCGGGCCATATTGAAGGGAAAGATGGGGAGCGTGCAGGATGGAGG

GGAGAGGATCGGGAGGCTCGGATGTATGTCTTTGAAGTGAAGCAGAAGGAGCCGGCCAAG

AAGGGAGGAAAGAAAAGGAGCAGAGATGAAGATTCTAGTGATAGTGAG

>CL25.Contig1_All 3 233 Pc16g12230 [Penicillium chrysogenum Wisconsin 54-1255] >gi|211586145|emb|CAP93893.1| Pc16g12230 [Penicillium chrysogenum Wisconsin 54-1255]

CCCATGCTGGTACAACAACCTCTCGCCAGTAACCAGGGTTGGGATAGCAACCTGCAGTTA

TTGAGCCCTGCGCGCATCCCATACCCCGGTGTGCAAAAGAGATCTTCACCCTCAGCGTCC

TCGCGCTCAGCCCCCTCAGGGTTGCCAGCCAACAACTACCACCGCCGCTCCAACAGCGCC

AACAAGCCGTTGCCCACCCCGGTGCAAACACCTCTTCAGAACTCATTCCTT

>CL25.Contig2_All 293 2224 minus strand C2H2 transcription factor (Rpn4), putative [Penicillium digitatum Pd1] >gi|425771654|gb|EKV10091.1| C2H2 transcription factor (Rpn4), putative [Penicillium digitatum PHI26]

ATGCTTATGTACCCACGTAAGGGTCTCATCACCGATCCATACAATTCAGAGGACATCATC

ATACAAGACGACCAGTACTCACCTGATTTCTATTCCGAGAACGCCTTGCACTACGATCAA

TCTCGTTTGCAGTCGCCGTTCCAGTGCCACCCCAGTGTACAGCTCTCCCCGCCATCTTCC

GCACCTTCTTCGCCTACTTTCTCCTCTCCGACCTTTTCGACTTCTTATCCGCATACGTCT

GACTGTCTATCGTTTGGGGCTCCTTCGATGACCCCAGCGCCTTCGTACAACCCGTCGTAC

GACATCCTGGGTTCCAATTCACCTTCGCACTATTTGCCTCAATTTCCTCAGCAATATTAT

ACGCCGATGCTGGTACAACAGCCTCTCACCGGTAACCAGGGTTGGGACAGCAACCTGCAG

CTGTTGAGCCCTGCACGTATCCCATACGCCGGTGTGCAAAAGCGATCTTCACCCTCGGCA

TCCGCGCGCTCGGCGCCCTCAAGGTCGGCAGCCAACAACTTCCACCGCCGTTCCAATAGC

GCCAACAAGCCGCTACCTACCCCGGTGCAAACCCCTATCCAAAACTCATTTCTTGCTACT

CCCTTCCAAAATTTCGACCCCTCCTCCCAAGATGGTCAAAATGCCGAGGCCGAGTCTGCG

ATGAGAAAAGCGATCATGGACCAGCAGAAGCAGTCAACCCCGCAGCAAATGCAGAACGAC

TACTCTCTGGCTCCATCGGTATCGACAATGAGCCATAACTCGCCGGTGACTCCGCAGACA

ACCCTCGACGAACTCGACGAAGCATCCAAGTCGATGACTAATGAATACAACAACACCAGT

GGTGCCATGTCAATTGGAGTCCCAAAACTGAACAGAACCATTTCCGACATATACCAAGAT

GAATTATATAATCCCGCCATCATGGCAGCACCCCAGGTGTCCAAACCGATGGGACAAACC

CATCTCAACCCGCGCAATTTCATTGCCGATCGCCTACAGGCTGCCAGTCAGGGACACATG

TCTGCTCGTTCGCAATCACCTGCCAACAGACGGGATCGGTCACCTTTCCGTACCTCTTCC

CCATTCGCTGGTGAGATGGGCAGCAATGCTCTCCAGCATCCGCAGATGGCTACCAGCATC

CCGTTGGGCCAGAACGTTATGCACGGAATGAACACCGGAGAAATCAAGACTATGTCCCCC

AAGGATGCAGTCTTGGATTTCCAAGACAACGATGATGCCCTGCCTCCTCTGTTCCCCCCC

GGCCAAGCTGATTTCAACCTGGGTGATGCGCTTGGCTTGCGACGTGATAATGGCTCAATG

CGACCGATAGAAGCATTCCCTCAATATTCGGTACCCAGCATGGCCCAACCACAATTCGCA

TTTTCCGAACCACAAATGCATCGTGCGCAGAGTAACCTCTTGCAACAAAGTAGCAAATTC

CCTTCGTTACCAGCAGTGGAATCGACTGAGAGCTCGCCTCCAAGCCAAATGAATATGCCA

ATTACAGAAGGTGTTCCTCGTCCTGCCAACACATCTTCGGATGCGGGCACCTACACTTGC

ACCTACCACAACTGCAGCGACCGATTTGACTCCCCCTCCAGACTCCAGCGACACAAGCGT

GAGGCACATCGGCAAACAACACCGGGCGGCCACCTTGTCAGCCGGGATACCTCGCTCCGC

AACTCACAGGCTGGTCCACACAAGTGCGAACGCATCAATCCGTCTACAGGCAAAGCATGC

AATTCCATCTTCTCCAGACCCTACGATCTTACACGGCACGAGCACACGATCCACACAGCC

GGAAAGCAGAAGGTGCGCTGTCACATCTGTAACGAAGACAAAACCTTCAGCCGAAACGAT

GCTTTGACAAGACATATGCGAGTGGTTCATCCCGAAATCGACTGGCCAGGCAAGCAGCGC

AGGAGGAGAGAC

>CL25.Contig4_All 1 393 minus strand Pc16g12230 [Penicillium chrysogenum Wisconsin 54-1255] >gi|211586145|emb|CAP93893.1| Pc16g12230 [Penicillium chrysogenum Wisconsin 54-1255]

TCAAACACTGGAGATATTAAGACTATGTCCCCCAAGGATGCGGTCTTGGATTTCCATGAA

AACGATGATGCCATGCCTCCTCTGTTCCCCCCGGGTCAGGCTGATTTCAACCTGGGCGAT

GCGCTTGGCTTGCGACGCGATAGTGGCTCGATGCGACCGATGGAAGCATTCCCTCAATAT

TCGGCACCCAGCATGGCCCAGCCACAATTCGCATTTACTGAACCTCAGATGCATCACCCT

CAGAATAACCTCTTACCGCAGAGTACCGAGTTCCCGTCGCTCCCAACAGTGGAATCTACC

GAGAGCTCGCCTCCAAGTCAAATGAATATGTCAATTACAGAGGGTATCCCTCGTCCTGCC

AACACATCGTCGGATGCGGGCACCTACACTTGC

>CL26.Contig1_All 8 787 Flap endonuclease 1 [Penicillium digitatum PHI26] >gi|425784232|gb|EKV22023.1| Flap endonuclease 1 [Penicillium digitatum Pd1]

ATGGGTATTAAGCACCTGTACCAGGTTATCTCCGAGAATGCCCCCGATGCGATCAAGACG

GGCGAAATCAAGAACCACTTCGGCCGCAAGGTCGCGATTGATGCGTCGATGAGCATCTAC

AGTTTCCTGATCGCCGTGCGCTCGGAGGGTCAACAGCTCATGAGTGAGTCAGGTGAGACC

ACATCCCACCTGATGGGCATGTTCTACCGTACACTCCGGATGGTGGATAACGGCATCAAA

CCTCTCTATGTTTTCGACGGAGCCCCGCCGAAACTCAAGTCCGGTGAATTGGCGAAGCGT

GTTGCACGCAAGGCTGAAGCAGCCGAAGCCCATGAAGAGGCGAAAGAAACCGGAACTGCG

GAAGACATCGAAAAATTCTCACGACGGACTGTCCGCGTCACCCGTGAACATAATGCAGAG

TGCAAGAAGCTCCTTGGACTGATGGGAATTCCTTTCATTAACGCACCGACGGAAGCCGAA

GCCCAGTGTGCCGTCCTGGCTCGTGCGGGTAAGGTGTATGCAGCTGCGTCCGAAGACATG

GATACATTGTGTTTTGAGTCGCCGATTCTCCTGCGCCACCTGACATTCAGTGAGCAGCGC

AAGGAACCTATCCAAGAAATCCACTTGGATCGCGCACTTGAGGGTCTTGATATGGACCGC

CCACAGTTCATCGATCTCTGCATTCTTCTCGGCTGTGACTACCTGGAGCCTATTCCCAAA

GTGGGAGCCACCACTGCTCTCAGCCTCATCAAGGAGCACAAGTCTCTGGAGAAAGTCCTG

>CL26.Contig2_All 8 685 Flap endonuclease 1 [Penicillium digitatum PHI26] >gi|425784232|gb|EKV22023.1| Flap endonuclease 1 [Penicillium digitatum Pd1]

ATGGGTATTAAGCACCTGTACCAGGTTATCTCCGAGAATGCCCCCGATGCGATCAAGACG

GGCGAAATCAAGAACCACTTCGGCCGCAAGGTCGCGATTGATGCGTCGATGAGCATCTAC

AGTTTCCTGATCGCCGTGCGCTCGGAGGGTCAACAGCTCATGAGTGAGTCAGGTGAGACC

ACATCCCACCTGATGGGCATGTTCTACCGTACACTCCGGATGGTGGATAACGGCATCAAA

CCTCTCTATGTTTTCGACGGAGCCCCGCCGAAACTCAAGTCCGGTGAATTGGCGAAGCGT

GTTGCACGCAAGGCTGAAGCAGCCGAAGCCCATGAAGAGGCGAAAGAAACCGGAACTGCG

GAAGACATCGAAAAATTCTCACGACGGACTGTCCGCGTCACCCGTGAACATAATGCAGAG

TGCAAGAAGCTCCTTGGACTGATGGGAATTCCTTTCATTAACGCACCGACGGAAGCCGAA

GCCCAGTGTGCCGTCCTGGCTCGTGCGGGTAAGGTGTATGCAGCTGCGTCCGAAGACATG

GATACATTGTGTTTTGAGTCGCCGATTCTCCTGCGCCACCTGACATTCAGTGAGCAGCGC

AAGGAACCTATCCAAGAAATCCACTTGGATCGCGCACTTGAGGGTCTTGATATGGACCGC

CCACAGGTACTATATCTT

>CL27.Contig1_All 76 708 minus strand DnaJ chaperone (Caj1), putative [Penicillium digitatum Pd1] >gi|425771765|gb|EKV10202.1| DnaJ chaperone (Caj1), putative [Penicillium digitatum PHI26]

AAGACTCTTAGTGTCGCCCAAGATGCCACCCAACAACAGATCCGCAGTGCATATAAGAGG

GAATCACTAAAATCCCATCCCGATCGAGTCCCGATCGACTCCCCGGAACGGCCCTCACGC

ACACGAAAGTTCCAGGAAATCAACGACGCCTACTTCACCTTATCCGACCCCGCGCGTCGT

CGGGAGTACGACGCTACTCGCGCACACCAGGCGGCCGAGGAAGAAGCGGACGCGGAAGTC

CCTCCAACCAGCACTGGCGGTTTCCCCTGGTCTAGCTTTGGGTTTGGCGGGACCCGCGAT

GACCGTAACAATGACCAGTTTGGCTCTGTCTTTGAAGAAATGCTGCGCGAAGAAGGCCTC

GCAGAGGAGAACGAGGGAGATGGCGGGAGGCGGACTCGCCCAACCAGGCGATTCTGGGCA

GTGGTCGGCGGTGTAAGTGGTGGCACTATGGGCTTCATTGTTGCCAACTTACCGGGTGCG

CTGGCGGGTGCTGTTGCGGGCAATCGTCTCGGCGCGATCCGCGATGCCAAGGGCAAAAGT

GTTTATGAGGTTTTCTTGGATCTTCCAGCGCAGGATCGGACGCGTCTCCTGGGTGAGCTT

GCGGCTAAGGTTTTCCAGTCTACTATGGGACAC

>CL27.Contig2_All 68 700 minus strand DnaJ chaperone (Caj1), putative [Penicillium digitatum Pd1] >gi|425771765|gb|EKV10202.1| DnaJ chaperone (Caj1), putative [Penicillium digitatum PHI26]

AAGACTCTTAGTGTCGCCCAAGATGCCACCCAACAACAGATCCGCAGTGCATATAAGAGG

GAATCACTAAAATCCCATCCCGATCGAGTCCCGATCGACTCCCCGGAACGGCCCTCACGC

ACACGAAAGTTCCAGGAAATCAACGACGCCTACTTCACCTTATCCGACCCCGCGCGTCGT

CGGGAGTACGACGCTACTCGCGCACACCAGGCGGCCGAGGAAGAAGCGGACGCGGAAGTC

CCTCCAACCAGCACTGGCGGTTTCCCCTGGTCTAGCTTTGGGTTTGGCGGGACCCGCGAT

GACCGTAACAATGACCAGTTTGGCTCTGTCTTTGAAGAAATGCTGCGCGAAGAAGGCCTC

GCAGAGGAGAACGAGGGAGATGGCGGGAGGCGGACTCGCCCAACCAGGCGATTCTGGGCA

GTGGTCGGCGGTGTAAGTGGTGGCACTATGGGCTTCATTGTTGCCAACTTACCGGGTGCG

CTGGCGGGTGCTGTTGCGGGCAATCGTCTCGGCGCGATCCGCGATGCCAAGGGCAAAAGT

GTTTATGAGGTTTTCTTGGATCTTCCAGCGCAGGATCGGACGCGTCTCCTGGGTGAGCTT

GCGGCTAAGGTTTTCCAGTCTACTATGGGACAC

>CL28.Contig1_All 1 594 minus strand Pc09g00310 [Penicillium chrysogenum Wisconsin 54-1255] >gi|211581512|emb|CAP79278.1| Pc09g00310 [Penicillium chrysogenum Wisconsin 54-1255]

TGCCACCTTCATAAGCTTGGCATTGCTCATGGAGATGTACGAATTGATAATGTACTCTTG

GATAATCGAGGCTCTGCTATTCTTTGTGATTTCAGTGCCGCTAGTCCCTTTGGTTATTCA

AATCTGGTCATCTCGGATCTTCCACTCCCAGTCAATGGTCCGTCGCCAAATCTGTCTGAG

GCAACTGATATGTTTGCAATGGCCTCGCTCCTTTTCCAGGTTGAGCATGGAATCAAACCT

GAGCTTTCTGTTGACAGTGATGGTGAATTGATTTTGCCTAGCATACAGACCAATCATCTG

GGTATTGATGCGATTATTCGAAATGCCTGGCTTAAACAATATAGCAGCACCTCGGAGATG

CTACAGAACCTTTATGCACTTGACGCTCAGACTAGTCAAGCTGTTTACGGTACCCAAATA

CATTCAGAGCCAATTGCTTCACTGAGAGAGCGGATCAAAGTATGGAGAAATGGACGCGAG

AACAGTATTGGCCGTGTACTTGATGGTATACTTTCAGGAGACCAGTTGCAAGTACTAGCA

GATTGCTATGACTTGGATAGAGATGCCGAATTACGGTTCACAAGCTATAGTGTA

>CL28.Contig2_All 1 609 minus strand Pc09g00310 [Penicillium chrysogenum Wisconsin 54-1255] >gi|211581512|emb|CAP79278.1| Pc09g00310 [Penicillium chrysogenum Wisconsin 54-1255]

TGCCACCTTCATAAGCTTGGCATTGCTCATGGAGATGTACGAATTGATAATGTACTCTTG

GATAATCGAGGCTCTGCTATTCTTTGTGATTTCAGTGCCGCTAGTCCATTTGGTCAATCA

AATCTAGTCATTTCGGATCTTCCGCTTCCAGTCAACGGTCCTTCGCCAAATCTGTCTGAG

GCAACTGATATGTTTGCAATGGGCTCGCTCCTATTCCAGGTTGAGCATGGAATTAAACCT

GAGCTTTCTGTTGACAGGGATGGTAAATTGATTTTGCCTACAATACAGACGAATCACCAA

GGCATTGATACTATTATCCGAAACGCTTGGCTTGGACACTATAGCAGCACCTCGCAGATG

CTAGAGAACCTTCATTCACTTGACGTTTAAATTGGCCAAGGTGTTCATGATACCCAAATA

CACTCAGAGCCAGTTGCTTCATTGAGGGAGCGGGTCAGAGCATGGAGAAAAAGACGTGAG

AACAACATTGGCTGCGTACTCGATGGCATACTCTCAGGAAATCAGTTGCAAGTACTGGCA

GATTGCTATGGGTTGGACAAAGATGCCGAATTACGTTTTGCTAGCTATGATGTATGCATG

CATGGGAAC

>CL29.Contig1_All 198 1823 minus strand Choline oxidase (CodA), putative [Penicillium digitatum Pd1] >gi|425767105|gb|EKV05687.1| Choline oxidase (CodA), putative [Penicillium digitatum PHI26]

ATGGCCACCACTAACGAGCTCCCGGCCCTCGATGTCAACAGCTACGACTACATTGTCGTT

GGTGGCGGTACCGCCGGCTGTGTAATCGCGGCACGCCTGGCTGAGTACCTCCCTAACAAG

CGGACTCTGGTTATTGAGGGAGGTCCCAGTGATTTCATGGATGACCGTGTTCTGAACTTG

AAGGAATGGCTCAGCTTGCTGGGAGGAGAGCTTGACTACGACTACGGCACCACCGAGCAG

CCCATGGGTAACAGCAATATCCGTCACTCCCGAGCCAAGGTCCTGGGTGGTTGCTCTAGC

CACAACACCCTCATCTCTTTCCGTCCCTTCGAGTATGATTGCCGTCAATGGGTTTCCAAG

GGTTGCAAGGGCTGGGACTTTGAGACCTTCACCCGCATTATTGATAATTTGCGCAACACT

ATCCAGCCGGTGCATGCCCGCCACCGTAACCAGCTGTGTAAGGACTGGATCCAGGCCTGC

TCTAGCGCCATGAAAATCCCTGTCATCGAGAACTTCAACGATGACATCCGCAAGACTGGT

GAGCTGACTGAGGGTGTCGGCTTCTTCAACATCTCCTACAACCCCGATGATGGCCGCCGC

AGCAGTGCCAGTGTCGCCTACATCCACCCCATTCTCCGTGGTGATGAGAAGCGTCCTAAC

TTGACTATTCTCACCAACGCTTGGGTTAGCAAAGTTAACATTGAGGGTGATACCGTGACC

GGCGTTGATGTTACTCTGCAGTCTGGTATCAAGCACACACTACGTGCCAAGAAGGAAACC

GTTCTCTGTGCCGGAGCTGTCGACACCCCTCGCCTCATGATGCTCTCCGGTCTTGGTCCG

CGTGAGCAGCTGTCTGCACTCGGAATTCCCGTTGTGAAGGATCTCCCCGGTGTCGGTGAG

AACCTACTGGACCACCCCGAGAGCATCATCATGTGGGAGCTCAACAGCCCCGTGGATCAC

AACATGACCACAATGGACTCTGACGCTGGTATCTTCTTGCGCCGCGAGCTGCCCGACGCC

GCTGGATTCGACGGCAAGATCGCCGACGTGATGATGCACTGCTACCAGATTCCGTTCACC

CTCAACACCACTCGTCTCGGCTACGACGAGCCCTTGAACGCATTCTGCATGACTCCCAAC

ATCCCCCGCCCCCGCTCCCGCGGTCGTCTCTTCTTGGCCTCCGCCGACCCCTCCGTAAAG

CCCTCGCTGGACTTCCGTTACTTCACCGACCCCGAGGGCTACGACGCTGCCACGATCGTG

GCGGGTCTCAAGGCTGCTCGTGAGATTGCCAAGCAGTCTCCCTTCAAGGAGTGGATCAAG

CGCGAGGTTGCTCCGGGCCCCAAGTTGCAGACTGACGAGGAGTTGTCTGAATACGGCCGT

CGCGTTGCACACACTGTGTACCACCCCGCTGGTACTACCAAGATGGGCAACGTTGCCACC

GACCCCATGGCGGTTGTTGACCCCACCCTGAAGGTCCGTGGCCTAAAGGGCATGCGTGTC

GCTGATGCTGGTGTCTTCCCTGACATGCCTAGCATCAACCCTATGCTGACAGTCCTGGCC

ATTGGTGAGCGTGCCGCCGAGCTGATTGCTGGCGAGGCCGGTTGGTCTCGCAACCAACCC

CGTCTG

>CL29.Contig2_All 72 1697 minus strand Choline oxidase (CodA), putative [Penicillium digitatum Pd1] >gi|425767105|gb|EKV05687.1| Choline oxidase (CodA), putative [Penicillium digitatum PHI26]

ATGGCCACCACTAACGAGCTCCCGGCCCTCGATGTCAACAGCTACGACTACATTGTCGTT

GGTGGCGGTACCGCCGGCTGTGTAATCGCGGCACGCCTGGCTGAGTACCTCCCTAACAAG

CGGACTCTGGTTATTGAGGGAGGTCCCAGTGATTTCATGGATGACCGTGTTCTGAACTTG

AAGGAATGGCTCAGCTTGCTGGGAGGAGAGCTTGACTACGACTACGGCACCACCGAGCAG

CCCATGGGTAACAGCAATATCCGTCACTCCCGAGCCAAGGTCCTGGGTGGTTGCTCTAGC

CACAACACCCTCATCTCTTTCCGTCCCTTCGAGTATGATTGCCGTCAATGGGTTTCCAAG

GGTTGCAAGGGCTGGGACTTTGAGACCTTCACCCGCATTATTGATAATTTGCGCAACACT

ATCCAGCCGGTGCATGCCCGCCACCGTAACCAGCTGTGTAAGGACTGGATCCAGGCCTGC

TCTAGCGCCATGAAAATCCCTGTCATCGAGAACTTCAACGATGACATCCGCAAGACTGGT

GAGCTGACTGAGGGTGTCGGCTTCTTCAACATCTCCTACAACCCCGATGATGGCCGCCGC

AGCAGTGCCAGTGTCGCCTACATCCACCCCATTCTCCGTGGTGATGAGAAGCGTCCTAAC

TTGACTATTCTCACCAACGCTTGGGTTAGCAAAGTTAACATTGAGGGTGATACCGTGACC

GGCGTTGATGTTACTCTGCAGTCTGGTATCAAGCACACACTACGTGCCAAGAAGGAAACC

GTTCTCTGTGCCGGAGCTGTCGACACCCCTCGCCTCATGATGCTCTCCGGTCTTGGTCCG

CGTGAGCAGCTGTCTGCACTCGGAATTCCCGTTGTGAAGGATCTCCCCGGTGTCGGTGAG

AACCTACTGGACCACCCCGAGAGCATCATCATGTGGGAGCTCAACAGCCCCGTGGATCAC

AACATGACCACAATGGACTCTGACGCTGGTATCTTCTTGCGCCGCGAGCTGCCCGACGCC

GCTGGATTCGACGGCAAGATCGCCGACGTGATGATGCACTGCTACCAGATTCCGTTCACC

CTCAACACCACTCGTCTCGGCTACGACGAGCCCTTGAACGCATTCTGCATGACTCCCAAC

ATCCCCCGCCCCCGCTCCCGCGGTCGTCTCTTCTTGGCCTCCGCCGACCCCTCCGTAAAG

CCCTCGCTGGACTTCCGTTACTTCACCGACCCCGAGGGCTACGACGCTGCCACGATCGTG

GCGGGTCTCAAGGCTGCTCGTGAGATTGCCAAGCAGTCTCCCTTCAAGGAGTGGATCAAG

CGCGAGGTTGCTCCGGGCCCCAAGTTGCAGACTGACGAGGAGTTGTCTGAATACGGCCGT

CGCGTTGCACACACTGTGTACCACCCCGCTGGTACTACCAAGATGGGCAACGTTGCCACC

GACCCCATGGCGGTTGTTGACCCCACCCTGAAGGTCCGTGGCCTAAAGGGCATGCGTGTC

GCTGATGCTGGTGTCTTCCCTGACATGCCTAGCATCAACCCTATGCTGACAGTCCTGGCC

ATTGGTGAGCGTGCCGCCGAGCTGATTGCTGGCGAGGCCGGTTGGTCTCGCAACCAACCC

CGTCTG

>CL30.Contig2_All 2 286 hypothetical protein PDIP_30550 [Penicillium digitatum Pd1]

CAAAGTAAACGTGATGTCCTCTGTGCCATCATCGAGTTTCGCAATCTCAAAGGTCGACCG

GAAGGTGCTGATGGCGATTATCAACTGGCATATTGCCCGATAGCTCAAATCATTGCCTTC

GCTTTCCGCGATGGGTCTTTTGCTAATACCGACCTCACACCAGAGCTCATTTGGCGGCTA

CGAGTTCCAAGAGGCAGTCCATTTCTTCCCCTCCGGTGGAAGCCCGAAGTTCTTAATGTA

CCCTTCCTTCGACGTCTTGAACGCACTCCCTACGGCTACGGACTC

>CL30.Contig3_All 1 1032 hypothetical protein PDIP_67010 [Penicillium digitatum Pd1]

CTGATGGGTAAGCTGAAATTTTGATTCGAATTTCTCATGAACCAACTTCGACACTGATCC

TTGCCTACAGAACCAAATTCTTCATGCACGGCGATTTTCAACTGGCCTATTGCCCGATAG

CTCAGATCATTGCGTTCGCTTTCCGCGATGGAGCTTTTGCCAATGACCTATGATTCAAGT

CGGCAGGCGCTCAAGGAGTTAGGTCGAGATGCTAGATTCGAATACGATATTGGGCATTAT

AATTTCCGCCGCTGGACGGCAAATGAAGTCAACAAAAATTTTACCAGCCAGGAGCGACAA

AGGGTGCTCGGTCAGTCTGGCGACGCAGTTTTTGAAAATCATTACCAATCGCAATTTATT

GCGCGCGACCTTCAGCATGTCGTTCTTCTTCGACCCTCCCAAGAAGGTCTGGTACGCTTC

GCTGGGAGTATGCTTAGGAAGAGAGATTTATCGGCACCATCTGATCTTACCGAAGCTCAT

AAACGTGCCATTTGCCAGAACCCTGAAATTCTGCAACTGAGACGCGAGAAAAGAGAGCTT

ATGGCGGAGATGCGGTCCGTGGCTGGCACAATCAAGAACGCCCGTGACACCTTTCCACAC

CTCTTCCAAAGGCATGAGACCGTCAAAAAAGACATGGCCAAACTTCGGAAAACGTTGGCG

ATCGAAACTCGCGAGACAGCCAGAAAGGACTATTTTAATAATGCCCCCGTGCTTGAAGTT

GACAGGCAGATCAAGCAAATGCTTCTCGGCGAATCTGATGTTCAGGAACGCGATGCTGAT

AGTTCCGCTGAAGAAGACTGGGAGCTCCCTATCCCGGGCTACGTATTTCCTGAACGAGCT

CGGCTTGTGGAGAACTTTTACGGCCCTGACGCAGAAGACTTTCATGAGGACAAATTACTT

ACTCGACACATCCAGGTTACCAAGGACATGGTTGCGCTTCTACTGCTTTGCGAGCCGAGC

AGGCGAGGCAACCGTGTCAATTGGGATTTCGATGAAGAGGTAAACATCCCTATTGAACAG

CCCGAGCAACTG

>CL30.Contig4_All 261 1529 conserved hypothetical protein [Aspergillus fumigatus Af293] >gi|66850619|gb|EAL90946.1| conserved hypothetical protein [Aspergillus fumigatus Af293]

ATGTCACCTGCGCCTGTATATCCGGCTCAAACTGATGAGCACCAATACTGGACCGATCCC

ATTCTTTGCGAAGAAACTCGAGCCAGACTGGAACATTTTCGCAGTCTTGGATGGCTCCCT

CCAAACTTCAAGCCAAAGACCTTGGAAGGCATTGCTGTCGTCGAACGTTATTGGCGAAAA

TTTTGCATTCAATCGAACGAGGATTATGTGGACTTTCTTCTTTTGGAGGATCAGACGATC

TACATGAACTTTTTTGACTGGATGTTCAAAACATCACGAAAGAAACTGCTTCAGTCGTAT

GATGAATACTGGCGACGCCTTTGTCAATACTTCGAGCTATTCGCCCGTCGCCGCGTCGGT

GAGGATGCCCGTCAACAGATGCGACGATTTCTCGTAGGTCTGTTTCCAGCTGAGTGCAGA

ATCCCCCGATGAACGAAGGACAAAAACACTCTAGATATAGATGTATTTGGTGTGATTTAC

CGGCATCACTGGGTCCATTCGAGATTCTTTCGTCATAGGAGCATGATTGTTCAGTTTGCT

ACAGTCCAGCTCTGGTCCGCCATCACAGGCACGCGACCTGGCGTCTTGCTTCCTCAAGAC

ACCTCATCACCTGGCACTCCAGCTTTGGGCAAACGCAAACGGGATGACACCTTCCAGAGT

GATCTTCCAAAGCACATTACCTTGAAAGATCTTCCGGACTCGGTGTGCTATCGCGATATT

GAACTTCTCTACCTGAAAGACCCTCAAAGTAAACGTGATGTCCTCTGTGCCATCATCGAG

TTTCGCAATCTCAAAGGTCGACCGGAAGGTGCTGATGGAACCAAATTCTTCATGCACAGC

GATTATCAACTGGCATATTGCCCGATAGCTCAAATCATTGCCTTCGCTTTCCGCGATGGG

TCTTTTGCTAATACCGACCTCACACCAGAGCTCATTTGGCGGCTACGAGTTCCAAGAGGC

AGTCCATTTCTTCCCCTCCGGTGGAAGCCCGAAGTTCTTAATGTACCCTTCCTTCGACGT

CTTGAACGCACTCCCTACGGCTACGGACTCGATGGGTCTTTGCCAATGACCTATGATTTA

AGTCGGCAGGCGCTCAAGGAGTTGGGTCGAGATGCTAGATTTGAAGACGATACAGGGCAT

TATAATTTCCGCCGCTGGACGGCAAATGAAGTCAACAAAAATTTTATCAGCCAGGAGCGA

CAAAGGGTGCTCGGTCAGTCTGGCGACGCAGTTTTTGAAAATCATTACCAATCGCAATTT

ATTGCGCGC

>CL31.Contig1_All 2 1222 minus strand hypothetical protein PDIP_56750 [Penicillium digitatum Pd1] >gi|425773583|gb|EKV11928.1| hypothetical protein PDIG_47370 [Penicillium digitatum PHI26]

AGTTTAATGTTCACTATGCTCTACCAGTGCCTTGTCCCAGAGGAAGCATCAAATACCGCC

TTTTTCTACCACAGTGTCATCTTGATCGGGGCTTCATCTCTCATGGAACTCAGCATCGAG

CCTTTTTTCTCGGTAGTGCAACAACACATGCTTTACGAGAAACGCGCTGCTGTTGAAATG

CCAGCGGCCTTTCTGAGAAGTGCGGTGACATCCTCTGCCTTTATATGTGCCTCCCAAGTC

AACCATGACCTCGGTGTGCTACCATTTGCTCTTGGTCACCTCAGCTACTCACTGGCACTT

ATCTGTGGCTACTCGCTGGTCCTACTCAGAGGAACCAACACAACCCGGTTCTCTTTCTCA

TTGACACGTATACAATCCAGGAACCCCTCAAACTACTTTCTGGGACGGTTCTCACGCGAG

TTAACGTCGCTTGCTGCGAATGTCTTTTTCCAATCTTTGGTGAAGCATCTGCTCACACAG

GGCGACACTATGATGCTTGTAGCCCTTTCAGGGCTGGAAGATCAGGGAATATATTCTCTT

GCGTCGAACTATGGCGGGCTCATTGCACGGATCATCTTCCAGCCACTGGAGGAGAGCAGC

CGGAACCTATTCTCGGCCCTACTGAGCCGCGGTGTGGATGGGAAGCTGAAGAATGTCCAC

ATCCATACTGCCAAAAATCACCTTGTAGATATTCTACGCGCATACCAAGTGATGTCAATC

TTGATATTTCCGTTGGGCCCAATGATGGTTCCCCAATTGTTGCATATCCTGGGAGGTCGT

CTATGGGCCTCCCCCAAAACCAGGGACCTGCTCTCTGTCTATTGCTACTATATCCCATTT

TTGGCATTTAATGGGATTACCGAAGCGTTCGTGTCGTCTGCAGCAAATTCGCAACAGATA

CGGAAGCAGACGGCGTGGATGGGTTTCTTTTCCGCCTGTTATGCCTTGGCGGCGTATATG

TTCCTAGAAGTAGGGAACTTGGGTGCGTATGGATTGGTCTTGGCCAACATTGTGAATATG

GTGGTTCGAACCTTTTGGAGTTACAGTTTTATCAAATCGTACCTCCATCGAAATGGATGC

AGCCTGTACACGAAAGAAGTGGCCATTCGACCTGCTAGCTTTATTCTGAGTGCTCTGGCG

AGTTTGTTCTTGGGCGGATTTGGCCTACAATTGGGAATTATCAAAGCATGCGTCTTGAGT

GGCAGCTATGCTCTCCTCATG

>CL31.Contig2_All 78 1583 hypothetical protein PDIP_56740 [Penicillium digitatum Pd1] >gi|425773582|gb|EKV11927.1| hypothetical protein PDIG_47360 [Penicillium digitatum PHI26]

ACTTTTAGAATACTCGTCATGGGTAAGCGAAGCCATCTCGAGCCAACGGAGCAGCGACCG

AAGAAAAAGTCCAAGTCCGAGAAGCCCACAAAAGCTTCCAAAGACAAGCAGGAAAACGGA

GATGAGAAGACTACATCCTATTCTGACGCGGCCGCTCTTTCTGAGATCCCCCAGTCAGAC

ATTGACAGTTTCTTGACTAAGAACGTGATAAAAATATCTGATTCCTCCTCACCCGACGCC

TCCCAATTCCGCCCAATCCTTTCGTTTGATCACCTGCCTGAATGTGACGCGGGTCTGTAT

ACTCAATTGAGGTCTTTCTCCGCACCAACGCCGATTCAGTCCTCTACCTGGCCGCTTCTT

TTTGCTGGACGTGATGTGATTGGTATTGCAGAGACTGGAAGTGGCAAGACCCTGGGTTTT

GGCTTGCCTTGTCTGAAGAAATTGATTGACTCCAAGTCAAGCAAACCTTGTCAACCCAAG

GCAGTGATTATTTCACCAACGCGAGAGCTTGCAATGCAGATTTATGACCAGCTTGTTAAA

TTCGGCGACACTGAAAAGACTCGGGTGACATGCATTTACGGTGGTGTGGGTAAAGATGAA

CAGCGTCGGGCTCTTCAAAAGGCTGCCATTGTGGTTGCCACTCCTGGCCGTCTCAAGGAT

CTCCTGAATGATGGTTCGATTGACCTTGCAAAGGTCACCTATCTCGTCTTGGATGAGGCA

GATCGCATGCTTGATAAAGGATTTGAACAGGATATCAAGGACATTGTCAAGCCAATGCCC

GTCTCCAGGAGACAGACTGTCATGTTTACGGCAACATGGCCCCGGTCTGTTCGAGATCTC

GCAGCATCCTTTATGAAGACCCCGGTAACTGTCACCATCGGCGGAGACCCGTCTGCGGAT

CCTCGAGCCAACACAAGGATCAAGCAAGTCGTTGAGGTTATTGATGGTCGGGAGAAGGAG

GGCCGACTCGTACAACTGCTTACTAAATCCCAGCGTGGTAATCAATCGCCCGAGAAAGTC

TTGGTTTTCTGCCTCTATAAGAAAGAGGCTATGCGCATTGAGAATCTCATCAGAAACAAG

GGATTTGCAGTCGCCGGCATTCACGGCGATTTGAACCAATCAGATAGATTCCGAAATCTC

GATGCTTTCAAGAAGGGCAATGCTACCGTTCTTGTCGCAACCGATGTGGCAGCGCGTGGC

CTTGATATCCCGAATGTGAAACTGGTGATTAATGTCACTTTCCCTCTTACCGTTGAGGAC

TATGTGCACCGGATTGGAAGAACTGGACGTGCTGGGGCCGATGGCCTTGCCATCACCATG

TTTACTGAAACTGACAAGGGACTATCCGGCGGATTGATCAATGTTCTCAAGGCAGCTAAG

CAAGACGTGCCAGAGGCGCTGTTGAAGTTTGGCACAACAGTGAAGAAGAAGCAGCACGAT

GTCTACGGTGCCTTTTACAAGGACGTTGATATGGAAAAAACAGCGACAAAGATCACATTT

GACGAT

>CL33.Contig1_All 3 881 minus strand hypothetical protein PDIG_65880 [Penicillium digitatum PHI26]

CGTGATGATCCCGACGATGTAGCCGGGGAGCTAAAAGCTGACTACAAAATTGGCGGTGTC

TACGAGTCATACGCACAGAAGGCGGGCAATGGCATCATTTTCTATCTTTTCGTTTTACCA

TCCAAATTATATGAAAAGTGTCTGAACAAAACGAGTGATGTGGATAAAGTCCTTGGGCAC

TACAGTTCACTTGGTTTCCAACAAGAGGATTCTACAACATCTGCAGCAAACAGCATAGTG

AGCTTTATCACAAAACAGTTCAATTCGCAGGTATCCATCTTCAAATCGAGTGAGGCCGAT

CAAAAGGTCAAACCGACACGGAAAAATGGCCAAAAGAAGCGGACCCAGAAGACACGGACC

CCAAAAACCTCGAAACGGACTGGAAAGAACCGTCAGTTTCATTCACCAAAGCCTACATTT

TGCCAGCCGTCTGGCCAAAGTGCCGAGACGACTGTTGATGGAAGTCTCCACGCTACAAAC

CCCAATAGCACAGAATTTCCGAGTCCAATGAACTCGCCTCGTCTGCACGATGGTACGAAT

CTTGGATTGTCGCATTCTAGTACGGCAAATGTGGAAGCCTCTGCTCACTCTTTTCATGAA

TACGAATCCCCTATTACGGCAGTTGAACCCATGAACCACGCGATATCTGGGGGATCAATA

GTGTCTCTTTCAAATGTCTCCTTAGACGCCAGCCAAACTCAACCACTGGCGCTGAGCGGG

ATGTTCAACAATGCCGCTCACTTGATGAATGAATTCCAATTGGAGCCGATGACATATGGT

GCCAATGCTGCTCAGCTAATGCACGAATTCCATCTAGAAACCTTCAGCCCGGCAGACTAC

ACCAATGCTGCCCAGCTGATGCAGCAATTTGACATGTTT

>CL33.Contig2_All 3 908 minus strand hypothetical protein PDIP_75200 [Penicillium digitatum Pd1]

CGTGATGATCCCGACGATGTAGCCGGGGAGCTAAAAGCTGACTACAAAATTGGCGGTGTC

TACGAGTCATACGCACAGAAGGCGGGCAATGGCATCATTTTCTATCTTTTCGTTTTACCA

TCCAAATTATATGAAAAGTGTCTGAACAAAACGAGTGATGTGGATAAAGTCCTTGGGCAC

TACAGTTCACTTGGTTTCCAACAAGAGGATTCTACAACATCTGCAGCAAACAGCATAGTG

AGCTTTATCACAAAACAGTTCAATTCGCAGGTATCCATCTTCAAATCGAGTGAGGCCGAT

CAAAAGGTCAAACCGACACGGAAAAATGGCCAAAAGAAGCGGACCCAGAAGACACGGACC

CCAAAAACCTCGAAACGGACTGGAAAGAACCGTCAGTTTCATTCACCAAAGCCTACATTT

TGCCAGCCGTCTGGCCAAAGTGCCGAGACGACTGTTGATGGAAGTCTCCACGCTACAAAC

CCCAATAGCACAGAATTTCCGAGTCCAATGAACTCGCCTCGTCTGCACGATGGTACGAAT

CTTGGATTGTCGCATTCTAGTACGGCAAATGTGGAAGCCTCTGCTCACTCTTTTCATGAA

TACGAATCCCCTATTACGGCAGTTGAACCCATGAACCACGCGATATCTGGGGGATCAATA

GTGTCTCTTTCAAATGTCTCCTTAGACGCCAGCCAAACTCAACCACTGGCGCTGAGCGGG

ATGTTCAACAATGCCGCTCACTTGATGAATGAATTCCAATTGGAGCCGATGACATATGGT

GCCAATGCTGCTCAGCTAATGCACGAATTCCATCTAGAAACCTTCAGCCCGGCAGACTAC

ACCAATGCTGCCCAGCTGATGCAGCAATTTGACATGTCTAATGGATACTATGGTCAGCAG

CACTTG

>CL33.Contig3_All 123 1094 minus strand hypothetical protein PDIG_65880 [Penicillium digitatum PHI26]

ATGGAGCTTCATGCATCGAAGAAGTTCACAACCAAAATTGCAAGATTTTGTGCAAATGTT

ATACTCGAGGACGACGGTGATACGCGTGATGATCCCGACGATGTAGCCGGGGAGCTAAAA

GCTGACTACAAAATTGGCGGTGTCTACGAGTCATACGCACAGAAGGCGGGCAATGGCATC

ATTTTCTATCTTTTCGTTTTACCATCCAAATTATATGAAAAGTGTCTGAACAAAACGAGT

GATGTGGATAAAGTCCTTGGGCACTACAGTTCACTTGGTTTCCAACAAGAGGATTCTACA

ACATCTGCAGCAAACAGCATAGTGAGCTTTATCACAAAACAGTTCAATTCGCAGGTATCC

ATCTTCAAATCGAGTGAGGCCGATCAAAAGGTCAAACCGACACGGAAAAATGGCCAAAAG

AAGCGGACCCAGAAGACACGGACCCCAAAAACCTCGAAACGGACTGGAAAGAACCGTCAG

TTTCATTCACCAAAGCCTACATTTTGCCAGCCGTCTGGCCAAAGTGCCGAGACGACTGTT

GATGGAAGTCTCCACGCTACAAACCCCAATAGCACAGAATTTCCGAGTCCAATGAACTCG

CCTCGTCTGCACGATGGTACGAATCTTGGATTGTCGCATTCTAGTACGGCAAATGTGGAA

GCCTCTGCTCACTCTTTTCATGAATACGAATCCCCTATTACGGCAGTTGAACCCATGAAC

CACGCGATATCTGGGGGATCAATAGTGTCTCTTTCAAATGTCTCCTTAGACGCCAGCCAA

ACTCAACCACTGGCGCTGAGCGGGATGTTCAACAATGCCGCTCACTTGATGAATGAATTC

CAATTGGAGCCGATGACATATGGTGCCAATGCTGCTCAGCTAATGCACGAATTCCATCTA

GAAACCTTCAGCCCGGCAGACTACACCAATGCTGCCCAGCTGATGCAGCAATTTGACATG

TTTAGCGGATAT

>CL34.Contig1_All 1 885 hypothetical protein PDIG_42960 [Penicillium digitatum PHI26] >gi|425776897|gb|EKV15095.1| hypothetical protein PDIP_41540 [Penicillium digitatum Pd1]

GAAAGAGTCAGACCCTGGGAAAATCTCCACCACGAGGGTTATTGGCTCCACCGTTCAGCG

AAACATACAATGATCCGACGTGCGCTTGCCCATAAAATTCCCATGGAAGATCGCATGTCC

CCTGGTCAATCGCCTGCCTCACAATTGGCAAACAAATCCTACTTGTACGACACATATCTT

GTTCCGGAAACTCATGCCGAAGCACCCCAAGAAGGACGAACTGGCTTCGACCACTCTGGT

CTAATACTGAACACGCTTAAATCTGCTATCGAGGAATTCGCAAAGCGTCACCAAACAAGG

AAAGTCGAGAGCTTGAGTCTCGAGGCGGCCGAGGAGTATATGCGCATTGGCTCATGGTCT

GAAGCGCATAGCCTTCTTCAACCGCTGTGGTCTACTCTTAGCTGGCGCCGCTCAGGGTGG

TGGCATCTGATGGTCAATTTCGGATGGGCACTCAGAGAATGCGCCCTCAGGGTGCAAGAC

AGCGAGACAGTGTTGCGAGTGGACTGGGAGCTTCTAAACAAGGCTTTCAAACCAAGACCT

GCGTGGCATTATGACATCCACAGGAGTCTTGAGAATCTGCCTTCAGAAAAGCCCAAACCC

TCTCTTGTCCTTCGCGCAGAGGATGTCATAACTAGTCTGGCCGCGTCATTAGTGTTCGAG

AGATCCGATGGCAACGTTGGGGAACCTCTACAGGCCCAACTTTCCATCACCTCCTGTGCT

CACAAATCCTCGGCTTCTATCCGACTTTCAGAGGTTAAATTGGTTTTCGAAGGATGTCTT

CGCCCAGTAAAGATTCAATCTGACCAGAATCAGGATGCTGACACCACAACCCCATGCTGC

GTCGCAACTCTTCCACTCCGCGAGCCGAGCAACTCTGATACCGCG

>CL35.Contig1_All 2 976 minus strand Pc12g14200 [Penicillium chrysogenum Wisconsin 54-1255] >gi|211582844|emb|CAP81047.1| Pc12g14200 [Penicillium chrysogenum Wisconsin 54-1255]

ACTCCCAAACCCGGCCAGGTCCTCATCCGGGTAGTGGTGTCGGGTACCAACCCGAAGGAT

TGGAAAGTCCCCAAATGGCAACCAGATACTGTCCTGAACCAGGGAGATGACATCGCGGGG

TATGTGGAGGCAGTAGGGGAAGGAGTCCGGAACTTCCGCAAGGGAGACAAAGTGGCGGCG

TTCCACGAGACGCTCAGCCCGCATGGAAGTTATGCCGAGTACGCCATTGCATGGGAACAT

ACAACATTCCACCTCACCGAGAAGACCAGCTTTGAAGAGGCTGCTACTATCCCCGTCGCT

GCGATGACTGCTGCTTTGGGCCTATATCAAGAACTGAGACTGCCTCTACCTTGGGCTCCT

GCTGATAAGCCCACACCGCTGGTTGTGTACGGCGGTGCCACAGCGGTTGGCGCATTTGCG

ATCAAGTTCGCTCAGCTATCGAACATTCACCCTATCATCGCTGTTGCAGGCAAAGGTGCA

GCCTTCGTTGAGACGCTGATCAGCAGAGAAAAGGGAGACACGATCGTCGACTACCGTGAG

GGTGATGACGCGGTCCGCTCGGCGATCAAGGCTGCCAGTAAAGGGACACCCATTCACCAT

GCCTACGATGCCGTGTCCGAGAAGGGAAGCTATGTGAATCTCGGCGCTGCTCTCGATGCA

CCTGGGAGGATTACTGTCGTTCTACCTGCAGAGCCTGATAAGGTGAAGGAGCAAATCAGC

ATCCATCGGACTATGGTTGGGTCTGTTCATATGGCCCCTGCTGAGGGCCAGGCTCTTGGT

GATAAGGAATTCGGAGCTGCTTTCTTCCCATTTATTGGCCAAGGTCTGGCACAAGGTTGG

TTCTCGGGCCACCCATATGAAGTGAGGAAAGGTGGATTGGGGGGTCTCGAGGGCGCATTG

AAAGATCTGGAGGCCGGCAAGGCTTCTGCGGTGAAATATTTGATTAAGATTGCCGAGACT

GAGGGAGTCCAGCAA

>CL36.Contig1_All 286 600 hypothetical protein PDIP_64740 [Penicillium digitatum Pd1] >gi|425776749|gb|EKV14957.1| hypothetical protein PDIG_30360 [Penicillium digitatum PHI26]

GTTCTTTGGAAAATTACACCTCTCTTTGCAGAATGGATAACGAATCCCTCGAATCCGCTC

TGGACAACCTCCCTTCTTAGCCAGACTTCAACAGTGGCTGAACTCGGCACCGGGATCTCG

GCGTTGGTCGCACTCGTACTGGCGCCCTCTGTGCGCCACTACATCGCCACTGACCAGGAA

TATGTGCGCAAATTATTCCGAACGAATCTTGACGCCAATGCGTCCGTCACTACGTCTCAT

AGCAACCGTAACAGCAAAGGATCAGGCAAGAGCAGGGGATCCAAATCAAAGCAAACCTTG

ACCGCCAAGAATGTA

>CL36.Contig2_All 118 1080 hypothetical protein PDIP_64740 [Penicillium digitatum Pd1] >gi|425776749|gb|EKV14957.1| hypothetical protein PDIG_30360 [Penicillium digitatum PHI26]

ATGGACCAAATCATCCGCTTGCTCGCCAATGGCGAAGAGATTGTCGATGTGGATGAGGAA

TCATTTCTCCTCTTCGCACAAGACATCCCAAGCAACAACCTAGGGATGCTCAATCCCCGT

GCACCATCAGTAGAGATCTCTATCAATGGTAATGAATATACAATCCATCAATCACCATCA

CTACTCTCCTCTCATCGAGCAGGAGGTACTACAGGCGCTGTTCTTTGGAAAATTACACCT

CTCTTTGCAGAATGGATAACGAATCCCTCGAATCCGCTCTGGACAACCTCCCTTCTTAGC

CAGACTTCAACAGTGGCTGAACTCGGCACCGGGATCTCGGCGTTGGTCGCACTCGTACTG

GCGCCCTCTGTGCGCCACTACATCGCCACTGACCAGGAATATGTGCGCAAATTATTCCGA

ACGAATCTTGACGCCAATGCGTCCGTCACTACGTCTCATAGCAACCGTAACAGCAAAGGA

TCAGGCAAGAGCAGGGGATCCAAATCAAAGCAAACCTTGACCGCCAAGTCTGTCAATAAT

ATCTCCTTCACAACGCTAGACTGGGAGACTGACCAGGCCGCCTCACTAAAGGAATGTATG

GATTCTGACGATGCCCATGGCCAAGACGCAGAGGAAGAAGGAGAGGACGATAAAGGCTTC

GACCTTCTCCTTTCCTGTGACTGCATCTACAACGAAGCACTCGTGGCCCCGTTCGTGCGC

ACTTGCGCTGAGATCTGTCGACTGCGGCCGGCGTATGTGGGTAGTTCTGAAGAACCTCGA

TCGCGCCGCAAGCCTACGGTTTGCATTATCGCGCAGCAGCAAAGGTCGCCGGATGTGTTT

GAGACTTGGCTCAGAGAGACGATGCGGGAGTTTGGGGTTTGGAGGTTGAGTGATGATGTG

CTTGGGGAGGGACTGAGAAGTGGGTCTGGATACTTGGTACATTTGTTATTGGCGAGGGAG

AAA

>CL36.Contig3_All 286 1029 hypothetical protein PDIP_64740 [Penicillium digitatum Pd1] >gi|425776749|gb|EKV14957.1| hypothetical protein PDIG_30360 [Penicillium digitatum PHI26]

GTTCTTTGGAAAATTACACCTCTCTTTGCAGAATGGATAACGAATCCCTCGAATCCGCTC

TGGACAACCTCCCTTCTTAGCCAGACTTCAACAGTGGCTGAACTCGGCACCGGGATCTCG

GCGTTGGTCGCACTCGTACTGGCGCCCTCTGTGCGCCACTACATCGCCACTGACCAGGAA

TATGTGCGCAAATTATTCCGAACGAATCTTGACGCCAATGCGTCCGTCACTACGTCTCAT

AGCAACCGTAACAGCAAAGGATCAGGCAAGAGCAGGGGATCCAAATCAAAGCAAACCTTG

ACCGCCAAGTCTGTCAATAATATCTCCTTCACAACGCTAGACTGGGAGACTGACCAGGCC

GCCTCACTAAAGGAATGTATGGATTCTGACGATGCCCATGGCCAAGACGCAGAGGAAGAA

GGAGAGGACGATAAAGGCTTCGACCTTCTCCTTTCCTGTGACTGCATCTACAACGAAGCA

CTCGTGGCCCCGTTCGTGCGCACTTGCGCTGAGATCTGTCGACTGCGGCCGGCGTATGTG

GGTAGTTCTGAAGAACCTCGATCGCGCCGCAAGCCTACGGTTTGCATTATCGCGCAGCAG

CAAAGGTCGCCGGATGTGTTTGAGACTTGGCTCAGAGAGACGATGCGGGAGTTTGGGGTT

TGGAGGTTGAGTGATGATGTGCTTGGGGAGGGACTGAGAAGTGGGTCTGGATACTTGGTA

CATTTGTTATTGGCGAGGGAGAAA

>CL36.Contig4_All 642 1016 hypothetical protein PDIP_64740 [Penicillium digitatum Pd1] >gi|425776749|gb|EKV14957.1| hypothetical protein PDIG_30360 [Penicillium digitatum PHI26]

CAAGAATGTATGGATTCTGACGATGCCCATGGCCAAGACGCAGAGGAAGAAGGAGAGGAC

GATAAAGGCTTCGACCTTCTCCTTTCCTGTGACTGCATCTACAACGAAGCACTCGTGGCC

CCGTTCGTGCGCACTTGCGCTGAGATCTGTCGACTGCGGCCGGCGTATGTGGGTAGTTCT

GAAGAACCTCGATCGCGCCGCAAGCCTACGGTTTGCATTATCGCGCAGCAGCAAAGGTCG

CCGGATGTGTTTGAGACTTGGCTCAGAGAGACGATGCGGGAGTTTGGGGTTTGGAGGTTG

AGTGATGATGTGCTTGGGGAGGGACTGAGAAGTGGGTCTGGATACTTGGTACATTTGTTA

TTGGCGAGGGAGAAA

>CL37.Contig1_All 211 2013 Fungal specific transcription factor, putative [Penicillium digitatum PHI26] >gi|425781872|gb|EKV19808.1| Fungal specific transcription factor, putative [Penicillium digitatum Pd1]

CCAAGGTTTGAGCGCCTCTCTCGCAACGGATCTCAGTCGGCCTACACTCCAATGCAAGCC

GGGCTAGCAAAGCCACGAGCCCGTCACCCACGCTTCCATGGTCCAACAAGCTCGGCCTTC

AACTTTGACGTGGCCAAATCTAGCTTACAGAATATGGGTATCACCCCGACAGAAGAGGGA

ACGCCAGATGACTTGAACACCGCACATGTTTCTCCAGCGGGCTCACCTTCTGCCAATCTG

GGTCAGCTTCTTCCAACCACACACCCTACCAAGGATCCAATATGGACGATCCCGCGTGAA

GAGGCGATGCGTCTCTGTAAGGTCTACGAGGAAGAGGTTGGGATCATGTATCCGGTGGTG

GATATCACCAAGGTTATCAGTCAGGCTAATCTGCTCTATACATTTATGGAGGCTGCTACC

AGGACAGGCTTTGCACAACGAGGGTTTCCGGGCTCTGATGGTCTTTATGACGAAAGCTCG

ATCATTCTTAAATTGATTCTCGCCACCACGCTCGTCGTGGAGGGAAGCGGTCAAAGTGAA

CTTGGGCAGCGGCTCTACTTGGATGTTAAGCCATCTATAGAATCGAAGTTATGGGAGTCT

CATACCATCAAAACCATTCAGCTCTTTGCTATTGTGGCCACATACCACTACCATACAGAT

GATGATGCCATGGCTTATCGGCTTATTGGATTGGCGGCACGGATGTGTTTAGCGATGGGA

TTGCATCGTCGGGATGCTTTGGTGAAGTCATTTCCAGTTGAGCAGCAGTGGAACGAGGTC

ACTCGGCTGTTCTGGTCAGTCTACAGCTTGGATCGACGATGGAGTTTAGGAACGGGTTTA

CCTTTCGTGATTCAGGACGAGGATATTGATCCTAACTTACCAGAACCTGATGCATCACTG

CCTTACTTGCGCTGCATGGTCCTATACAACCGGATCAGCTCCAAGATCTGGTACTCCGGT

TTGGGCTCCGAAGGCACCACTGACATCCGTCGCGATGAAATCGGCTATCTGGATTACCAA

ATTCTACAGTGGTACAAGCAAGTTCCAGACGAGCTCAAGTTCCATCCCGTTGAGACCCTC

AAAAACGGTGAAACTTTAAGCAGAGGCATGAGACGACTCAGGGTTCTTTTATACCTACGA

ATGAACCAGCTGCGCATCCTTATCTATCGCCCCGTGCTCCATTCTCCTGCCAGTATTGCC

GAAGATAGAGGTCATGCTCAGACTGTTGTGGAAGTAGCTAAAGACTCGGTCCATGTCCTC

ACTCGGTTGAACCAGATGTCCGATATCTACCGGACACAGCAAATAACGTTCAACTATTTC

CTGGTTGCTGCATTGGCGGTGCTGTTCCTAGCAGTATCCCATGCCCCGGTCGAGTTCAAC

CGACAGGTCCGCGATGAATTCTACATGGCGCTCGACTTAGTCAACGGGTTCAGCACCAAG

TCCTTCGTTTCCAAACGGTTGTGGAAGACAATTAAAGGTCTACGGAAGATCGGCGAGAAA

CTTGGCGTTATCGCCCGTCCTTTCGGACCGGACTCAAGTGATCCTCATTCAAGCGCGGCA

GTGGCTATGGCCGGACTCGCGGGGCATCCAATTCAGGACCTGTCCATGTACGGGCCCATG

AATGGTGGGAACGAACTTGGAAATAGTCCTCTCAATGGACTCCAGATGAGCCAAGAGTTG

ACAAACCTGTTTGAGGCTGTTGGTGCTTTTGGCAATTTCATGCCTAACAGTTCGAGCGAT

GGTATTAGTGGCTACGTTGGGCCGGATGGGGAAATCCAGAACACTGGCGAAGGGCTATCG

GGG

>CL37.Contig2_All 80 2035 Fungal specific transcription factor, putative [Penicillium digitatum PHI26] >gi|425781872|gb|EKV19808.1| Fungal specific transcription factor, putative [Penicillium digitatum Pd1]

GAGTTCCGGTCAATGACAGATCAGATCACTACTCTACAAGACCAAGTCAATAGCTTGTTT

ACCAATCTCAATGATCTTCGTGCTCAGAGACCAACATTTGATTCACCAGGGTTTGAGCGC

CTCTCTCGCAACGGATCTCAGTCGGCCTACACTCCAATGCAAGCCGGGCTAGCAAAGCCA

CGAGCCCGTCACCCACGCTTCCATGGTCCAACAAGCTCGGCCTTCAACTTTGACGTGGCC

AAATCTAGCTTACAGAATATGGGTATCACCCCGACAGAAGAGGGAACGCCAGATGACTTG

AACACCGCACATGTTTCTCCAGCGGGCTCACCTTCTGCCAATCTGGGTCAGCTTCTTCCA

ACCACACACCCTACCAAGGATCCAATATGGACGATCCCGCGTGAAGAGGCGATGCGTCTC

TGTAAGGTCTACGAGGAAGAGGTTGGGATCATGTATCCGGTGGTGGATATCACCAAGGTT

ATCAGTCAGGCTAATCTGCTCTATACATTTATGGAGGCTGCTACCAGGACAGGCTTTGCA

CAACGAGGGTTTCCGGGCTCTGATGGTCTTTATGACGAAAGCTCGATCATTCTTAAATTG

ATTCTCGCCACCACGCTCGTCGTGGAGGGAAGCGGTCAAAGTGAACTTGGGCAGCGGCTC

TACTTGGATGTTAAGCCATCTATAGAATCGAAGTTATGGGAGTCTCATACCATCAAAACC

ATTCAGCTCTTTGCTATTGTGGCCACATACCACTACCATACAGATGATGATGCCATGGCT

TATCGGCTTATTGGATTGGCGGCACGGATGTGTTTAGCGATGGGATTGCATCGTCGGGAT

GCTTTGGTGAAGTCATTTCCAGTTGAGCAGCAGTGGAACGAGGTCACTCGGCTGTTCTGG

TCAGTCTACAGCTTGGATCGACGATGGAGTTTAGGAACGGGTTTACCTTTCGTGATTCAG

GACGAGGATATTGATCCTAACTTACCAGAACCTGATGCATCACTGCCTTACTTGCGCTGC

ATGGTCCTATACAACCGGATCAGCTCCAAGATCTGGTACTCCGGTTTGGGCTCCGAAGGC

ACCACTGACATCCGTCGCGATGAAATCGGCTATCTGGATTACCAAATTCTACAGTGGTAC

AAGCAAGTTCCAGACGAGCTCAAGTTCCATCCCGTTGAGACCCTCAAAAACGGTGAAACT

TTAAGCAGAGGCATGAGACGACTCAGGGTTCTTTTATACCTACGAATGAACCAGCTGCGC

ATCCTTATCTATCGCCCCGTGCTCCATTCTCCTGCCAGTATTGCCGAAGATAGAGGTCAT

GCTCAGACTGTTGTGGAAGTAGCTAAAGACTCGGTCCATGTCCTCACTCGGTTGAACCAG

ATGTCCGATATCTACCGGACACAGCAAATAACGTTCAACTATTTCCTGGTTGCTGCATTG

GCGGTGCTGTTCCTAGCAGTATCCCATGCCCCGGTCGAGTTCAACCGACAGGTCCGCGAT

GAATTCTACATGGCGCTCGACTTAGTCAACGGGTTCAGCACCAAGTCCTTCGTTTCCAAA

CGGTTGTGGAAGACAATTAAAGGTCTACGGAAGATCGGCGAGAAACTTGGCGTTATCGCC

CGTCCTTTCGGACCGGACTCAAGTGATCCTCATTCAAGCGCGGCAGTGGCTATGGCCGGA

CTCGCGGGGCATCCAATTCAGGACCTGTCCATGTACGGGCCCATGAATGGTGGGAACGAA

CTTGGAAATAGTCCTCTCAATGGACTCCAGATGAGCCAAGAGTTGACAAACCTGTTTGAG

GCTGTTGGTGCTTTTGGCAATTTCATGCCTAACAGTTCGAGCGATGGTATTAGTGGCTAC

GTTGGGCCGGATGGGGAAATCCAGAACACTGGCGAAGGGCTATCGGGGGTGCTGGGTGAT

GAGGGAGAGTTTGCTCGAGTCATCCGGGATTTGTTT

>CL38.Contig1_All 1 210 minus strand Endoplasmic reticulum calcium ATPase, putative [Penicillium digitatum Pd1] >gi|425771519|gb|EKV09960.1| Endoplasmic reticulum calcium ATPase, putative [Penicillium digitatum PHI26]

CTTTCCCATGATGCCAAGAGCGGAGCCTTCTCTAGCATCGGTGAGCCCACCGAGGGAGCC

CTGCGTACTTTGGTTGAAAAGATTGGATCCACCGACCTCGCCTTGAACCAGAAGCTTTAT

CGCCTCCCTGCGTCCGAAAGATTGCATGCCGCCAGCGCCCATTACGAGTCCCGTCTTCCC

CTCAAGGCCACCTATGAATTCTCCCGTGAC

>CL39.Contig1_All 2 277 minus strand hypothetical protein PDIG_21570 [Penicillium digitatum PHI26] >gi|425781406|gb|EKV19375.1| hypothetical protein PDIP_23850 [Penicillium digitatum Pd1]

GAGATCCCAGTTATAGGATATGGGGTGTACATGATTCCATCATCCGCAACCGAGAAGTCT

ACCCTCGAAGCCTTGCACGTTGGATTCCGCCATATCGACTCGGCAATCATGTATCGCAAC

GAGAAGCCCTGTGGAAACGCAATTCTGAATTCCAATCTGGATCGATCACAGATCTTCTTT

ACGACCAAGATCCCGCCGGGATCCATGGGATACGAGTCAACCAAGCAAGCTATCAGTTCC

AGTCTGCGAGAAGCGGCACAGGAATATTTTGACCTG

>CL39.Contig2_All 204 407 minus strand Uncharacterized oxidoreductase C28F2.05 OS=Schizosaccharomyces pombe (strain 972 / ATCC 24843) GN=SPBC28F2.05c PE=3 SV=1

CTCACACTAAAGATATCGCAGATCGACTCGGCAATCATGTATCGCAACGAGAAGCCCTGT

GGAAACGCAATTCTGAATTCCAATCTGGATCGATCACAGATCTTCTTTACGACCAAGATC

CCGCCGGGATCCATGGGATACGAGTCAACCAAGCAAGCTATCAGTTCCAGTCTGCGAGAA

GCGGCACAGGAATATTTTGACCTG

>CL39.Contig3_All 34 876 hypothetical protein PDIG_21570 [Penicillium digitatum PHI26] >gi|425781406|gb|EKV19375.1| hypothetical protein PDIP_23850 [Penicillium digitatum Pd1]

ATGGCAAACCTTACTCTTCAGTCAAAGCATCGACTACAGTCAGGATATGAGATCCCAGTT

ATAGGATATGGGGTGTACATGATTCCATCATCCGCAACCGAGAAGTCTACCCTCGAAGCC

TTGCACGTTGGATTCCGCCATATCGACTCGGCAATCATGTATCGCAACGAGAAGCCCTGT

GGAAACGCAATTCTGAATTCCAATCTGGATCGATCACAGATCTTCTTTACGACCAAGATC

CCGCCGGGATCCATGGGATACGAGTCAACCAAGCAAGCTATCAGTTCCAGTCTGCGAGAA

GCGGCACAGGAATATTTTGACCTTGTTCTAATCCATGCACCCTACGGTGGCAAAGAAGCC

CGTCTGGGTTCCTGGGATGCACTAGTGGAAGCCCAAAAGGCAGGCACAGTGAGATCCATC

GGTGTCTCCAACTACGGCATCCACCACCTCGAAGAACTGGAGGCTTACATCCTGTGTGGT

GGGGGTGGTCAGATCGATGTCGGTCAGTATGAGCTGCATCCGTGGCTGGATCATTCGGAT

ATCGTGGAGTGGTTGCAGAAAAGGAAGGTTGTTGTTGAGGCGTATTCGCCTCTTGCACAT

GGAAGTCGGTTGGCGGAGCCTGTGTTGTTGTCGATCGGGCAGAAGTATGGGAAGACGCCG

GCGCAAGTTTTGATTCGGTGGAGTTTGCAAATGGGATTCGTGCCTCTGCCCAAGTCTACT

ACGCCGAAGCGCATTCGGGAGAATGCCGATGTCTTTGACTTTGAGTTGACGGCTGAGGAT

ATGAAGTTGCTTGATACGGGAGAGTACTCTCCCACAGATTGGGATCCGACTGTTGATGAG

GAT

>CL39.Contig4_All 798 947 hypothetical protein PDIG_21570 [Penicillium digitatum PHI26] >gi|425781406|gb|EKV19375.1| hypothetical protein PDIP_23850 [Penicillium digitatum Pd1]

GGATTCGTGCCTCTGCCCAAGTCTACTACGCCGAAGCGCATTCGGGAGAATGCCGATGTC

TTTGACTTTGAGTTGACGGCTGAGGATATGAAGTTGCTTGATACGGGAGAGTACTCTCCC

ACAGATTGGGATCCGACTGTTGATGAGGAT

>CL40.Contig2_All 1 375 hypothetical protein PDIG_77480 [Penicillium digitatum PHI26] >gi|425783803|gb|EKV21623.1| hypothetical protein PDIP_04590 [Penicillium digitatum Pd1]

CACGGGTATGAGTGTGTCTATTCCATCTCGCAACCGCTGGGCCGGCCGGCCAAGAAAAAG

ACGACCCGGTCAGTGGCGGCGGGGGAAATCGGAAAGGCGCGCGGGAGGGAGGGGGAGGTG

GTTGACCGTCCGACTCGGCGGGGTGCCGGGAGGGCACCGAGGCCAACCACAGTAGCGAGG

GTGCAGAGGGCGCGCAGGGTCCCGAGTCGGGTCCCGAGTCCAACAACAGCATCCGGCTCG

GGGCCTGGTTCAAGGGAAGAGTCGCACAAGAGCGACTCCAGGGCCGGCACTGAAGTCACA

CCACCGGAGGAAGGCTTTCAATGGCCGAGTTTCACAGCGTGGTTCTTAGAGGTTCCAGGT

GAGCACAGACCTAGT

>CL41.Contig1_All 3 359 minus strand Pc16g12910 [Penicillium chrysogenum Wisconsin 54-1255] >gi|211586213|emb|CAP93961.1| Pc16g12910 [Penicillium chrysogenum Wisconsin 54-1255]

GTCCGCGAACGCCCCTCCAAATCATACTCCTGGCAAGCCTTCATGTCCGCAAACATCATT

GTCGAACTCCCCTGGAACGCCCTCATGTCCGTCCTCATCTTCGTCTGCTGGTACTACCCC

ATCGGGCTCCAACGCAACACCAGCGCCGACGACCTGCACGAGCGCGGCGCCTTGATGTGG

CTCCTCATCCTGAGCTTCATGATCTTCACCTGCACCTTCGCACACATGATGATTGCCGGC

ATCGAGCTCGCCGAAACAGGAGGAAACCTCGCCAACCTGCTCTTCTCCCTGTGTCTGGTC

TTCTGTGGTGTCCTCGCCACGCCCGACAAGATGCCCGGCTTCTGGATCTTCATGTAC

>CL41.Contig2_All 195 4715 minus strand ABC transporter, putative [Penicillium digitatum Pd1] >gi|425771857|gb|EKV10289.1| ABC transporter, putative [Penicillium digitatum PHI26]

ATGTCGGTCCTTGGGACATTTAACACCAACTTCGCCGGCATTCAAGACGCACAGGAATCA

CATGTCGCCAACATGGAAGCTCGCCGCCATGACCCTACTGCCAGCGTGAACACCGAAGAC

ACTGCCAATGAGAAGAGCGAGGTCGGTGACGAGAAGTACACGGATGCCGAAGTCACCCGT

CTGGCCCAGCAACTCACCCGCCAGTCAACGCGCTTCTCTGTGTCACCCCAGAATGCAGAG

AACCCTTTCATCGAAACCCATGAGGACTCAACTCTCAACCCTCACAGTGGAAACTTCAAG

GCCAAGAATTGGATGAAGAACCTCCTTGCTATCCAATCACGCGATCCAGAACGATACCCT

AAGCGTCAGGCTGGTCTGGCATTCAAAAACCTCAGCGTACACGGATTCGGCAGTCCTACA

GACTACCAGAAGGATGTGGCTAACTCTGTTCTGGAAATTGGTGCCTTTTTCCGTACGATG

GCCGGCACTGGCAAGCAGAAGATTCAAATTCTGCGCGACTTCGATGGTCTTGTTAAGAAC

GGTGAGATGCTGATCGTTCTCGGCCGTCCTGGATCTGGCTGTTCGACCTTCTTGAAGACC

ATTGCGGGGGAGATGAACGGTATCTTCAAAGATGCCAATTCTCACATGAACTACCAAGGA

ATCTCCGATAAGGAGATGCGCAATCAGTTCCGCGGTGAGGCTATCTATACTGCCGAAACC

GATGTCCATTTCCCCCAGCTTTCGGTAGGAAATACGTTGAAATTTGCTGCTCTGGCTCGT

GCCCCGCGCAATCGTTTGCCGGGCGTCAGTCGTGACCAGTATGCAGAGCACATGCGTGAT

GTTGTCATGGCTATGCTTGGTCTTTCGCACACTATCAACACCAGAGTCGGTAACGATTTC

ATCCGTGGAGTTTCCGGTGGTGAACGAAAGCGTGTCAGTATCGCCGAGGCTACTCTCTGT

GGCAGCCCTCTTCAATGCTGGGATAACAGTACCCGTGGTCTGGACAGTGCCAATGCTCTG

GAGTTCTGTAAGACATTGAATCTGATGTCTAAGTACTCGGGCACCACCTGTGCGGTCGCT

ATCTACCAGGCATCTCAGAGTGCCTACGATGTCTTCGATAAGGTGACTGTACTGTACGAG

GGTCGACAGATCTACTTTGGCCGCACCACCGAGGCTAGGGAATTCTTCACGAACATGGGC

TTCCATTGTCCCGACCGCCAGACTACCGCCGATTTCCTGACCTCTCTTACTAGCCCCGCT

GAGCGAGTTGTTAAGCCTGGCTTTGAGAAAATGGTTCCCCGTACCCCCGACGAGTTCGCT

AAGGCCTGGAAAAATAGTGCTGCGTACAAGGAGCTTCAGAAAGAGATTGACGACTACAAT

ACACAGTACCCTATTGGTGGCGAGTCGTTCCAACAGTTTGTTGAATCTCGCAAGGCTATG

CAGTCGAAGGGCCAGCGCGCTAAGTCTCCATACACCCTATCTGTCGCCGAGCAGGTCCAA

ATCTGTGTTACACGTGGTTTCCAGCGCTTGAAAAGTGACTACAGTCTAACTATATCCGCC

TTGATTGGAAACACGATTATGGCTTTGATTGTCGGTTCGGTTTTCTATCAGCTGCCCGAC

GACGTGACCAGTTTTTACTCTCGTGGTGCCCTCCTCTTCTTCGCTGTCCTGCTCAACTCC

TTCTCCAGTGCCCTGGAAATTCTAACTCTTTACGCGCAACGTCCTATTGTCGAGAAACAA

GCTCGCTACGCCATGTATCATCCGTTTGCCGAAGCAATTTCGTCAATGCTATGTGACATG

CCGTACAAAATCCTTAATGCCATCACTTTTAACGTTACGCTCTACTTCATGACTGGTCTT

CGCCAAAACGCTGGCGCTTTCTTCACCTTCATGCTCTTCTCATTCGTGACCACATTGACC

ATGTCGATGATTTTCCGAACCATCGCTTCTTACTCGCGTACCCTATCTCAGGCTCTGGTG

CCCGCCGCAATCCTGATCCTCGGTCTGGTCATCTACACTGGATTCACCATCCCAACCCGC

AACATGCTTGGTTGGTCTCGCTGGATGAACTACATTGACCCAATCGCATATGGGTTTGAG

ACCTTGATTGTGAACGAGTTCCACGGTCGCAATTTCCCCTGCAACCCAGAGTCTTTCATC

CCGGCGGGAGACTCCTACGCTGATGTCGGCCGGTTCAACAAGATTTGCTCGGCGAAAGGA

GCTGTGGCCGGCCAGAACTTTGTCAGTGGTGAGGCCTACTACACTGCAAGCTTCCAGTAC

TCCAACAGCCACAGGTGGAGGAACATGGGAATTATGATTGGGTTTATGGTCTTCTTCATG

GTCACCTACCTTGTCGGCACCGAGTACATCTCCGAGGCTAAGTCCAAGGGCGAGGTTCTG

CTTTTCCGTCGTGGCTACGCCCCCAAGAACTCCGGCAATTCCGATGGTGATGTCGAGCAG

ACACACGGTGTGTCCTCTGCGGAAAAGAAGGATGGTGCTGGCTCCGGCGGCGAACAAGAG

TCTGCTGCAATCCAAAGACAAACTTCTATTTTCCAATGGCAAGACGTGTGTTACGATGTT

CACATCAAAAATGAGGAACGTCGCATTCTCGACCACGTTGACGGCTGGGTTAAGCCCGGT

ACCTGCACTGCATTGATGGGTGTTTCTGGGGCCGGTAAAACCACTCTTCTGGATGTCCTT

GCCACGCGTGTCACAATGGGTGTTGTGTCTGGTGAGATGTTGGTTGATGGTCGTCCTCGC

GACCAGTCTTTCCAGCGTAAGACTGGTTACGTTCAACAGCAGGATCTCCATCTCCACACT

ACCACAGTCCGCGAGGCCCTCCGCTTCAGCGCCATTCTCCGTCAGCCGCGTCACGTTTCT

CACCAGGAGAAACTTGACTACGTCGAGGAAGTAATTAAGCTTCTTGGAATGGAACACTAT

GCGGATGCCGTCGTCGGTGTCCCCGGTGAAGGTCTCAATGTCGAACAGCGCAAGCGTCTT

ACTATTGGTGTCGAGCTAGCTGCCAAGCCGCAGCTGCTTCTCTTCCTGGATGAGCCCACT

TCGGGTCTTGACAGTCAAACATCTTGGTCTATCTTGGATCTTATTGACACCTTGACTAAG

CACGGTCAGGCTATTCTCTGCACAATTCACCAGCCCTCTGCTATGCTCTTCCAGAGATTC

GATCGTCTCCTATTCCTCGCTAAGGGTGGTAGAACCGTCTATTTCGGAGAAATCGGCGAG

CATTCTTCCACGCTCTCTAACTACTTTGAACGAAATGGTGCTCCCAAGCTTTCTCCTGAG

GCCAACCCTGCTGAGTGGATGCTTGAGGTGATTGGAGCTGCTCCCGGAACCCATAGTGAT

ATCGACTGGCCTGCTGTATGGCGCGAAAGCCCTGAGCGCAAGGCAGTACAAAACCACCTC

GCCGAGCTCAGGAACAACCTCTCTCTAAAGCCCGTTGCCACAACCGATAACGACCCAGCC

GGCTTCAATGAATTCGCTGCACCGTTCGCCGTCCAACTCTGGCAGTGTCTCATCCGTGTG

TTCAGCCAATACTGGCGGACTCCTATCTACATTTACTCCAAGACTGCGCTCTGCAGTTTG

ACTGCCCTCTACGTCGGTTTCTCCTTCTTCCACGCCCAGAACAGTATGCAAGGACTCCAG

AACCAGATGTTCAGTATCTTCATGTTGATGACAATCTTCGGTAACTTGGTGCAACAAATC

ATGCCACACTTCGTTACCCAGCGCTCCCTCTACGAAGTCCGCGAACGCCCCTCCAAAACA

TACTCATGGCAAGCCTTTATGTCGGCAAACATCCTTGTCGAACTCCCATGGAACGCCCTG

ATGTCTGTGCTCATCTTCCTCTGCTGGTACTATCCCGTCGGGCTGCAACGCAACGCCAGT

GCCGATGACCTCCATGAGCGAGGCGCTCTGATGTGGCTCCTCATCTTAACCTTTATGTTG

TTCACCAGCACATTCTCTCATATGATGATTGCCGGTATCGAGCTTGCCGAAACAGGAGGC

AATCTCGCCAACTTGCTCTTCTCCCTCTGTCTGATCTTCTGTGGTGTCCTCGCTACCCCG

GACAAAATGCCTCACTTCTGGATCTTCATGTACCGCGTCTCGCCGTTCACTTACCTAGTC

TCTGCGATGTTGTCGACCGGCACATCCGGTGCAAAGGTCGAGTGTGAGTCCGTCGAGTTG

CTCCACTTCGAGCCCACTGCCGGCAAGACCTGTTTCGAGTACATGAATACCTATATGAAC

GGACTCGTTGTCAACGGAACTCAGGTCGCTGCTCCTGCTGGCGGTTACCTCGTCGACAAC

AACGCCACTAGCAACTGCGCTTTCTGCACTATCGCAGATACCGATACTTATCTCGCCAGC

GTGCTGAGCTACTACAAAGATGCATGGCGCAACTTCGGTATCATGTGGGCGTTTATCATC

TTCAACATCTTTGGTGCTGTGTGCATCTACTGGCTCGCTCGTGTGCCCAAGGGAACACGT

AGCAAGAAGACAAAGACTGCT

>CL42.Contig1_All 2 271 minus strand Pc22g01540 [Penicillium chrysogenum Wisconsin 54-1255] >gi|211591215|emb|CAP97442.1| Pc22g01540 [Penicillium chrysogenum Wisconsin 54-1255]

ATGATGATGGGGTCGCACCACCAGACTAGCATGACATCGACCATGGTGCACAAGCAATGG

GGTAACATGCTGGTTGGTTTTGCACTGGCGCGCGGCATGACATACGTTCTGCTGTACCTC

AAGCCTCCGACCTCGTACCTCCCGGCTCGTCCCCCGACAGAGATCATTGCTGCGTTCTGT

CTGATCTCGGGAGGTCTGATTTTCATGATGAGCACCCGCAATGTGATTGAAGCGATGGAG

TACTATGAACTGGACGCAATGTTCACCTTC

>CL42.Contig2_All 260 2026 Integral membrane protein [Penicillium digitatum PHI26] >gi|425775811|gb|EKV14062.1| Integral membrane protein [Penicillium digitatum Pd1]

ATGCGGCCTAAAATAACTCGGGTCGCATTGGCGGCAACTTTTACTCTGTTTCTCGCATCC

CTTGCGACCGCGCTTCCACATGGTGACGATGAATCAATGGACATGGAAATGGATATGAAT

GCGGGCACCGCTACCCCGCAACCTACAACCACTGTGATGCAGAATCACACCAATGGACCG

ATGAGCTATTTTGCGTATAGCAAGCACTCTAGCACCATCATAGCTCACATTATCCTCATG

GTTCTAGGATGGTGCTTTGTTCTTCCGGTCGCCGTTATGCTGAGCATTGCACGCTCATGG

CTTGCGCTTCCGTCTCAGTTCCTCTTTCTGGCCTTCAACGTTCTCGGTGTCCTGCTTGGG

GCTGTCTACAACAGCCAGACCCCAGACCTCTACGAGAACAATGTACACCACAAATTGGGT

TGGGTCGCGACGTGCATTGTCACCGCTCAGGTCATCCTGGCTTTGCTCTTTGCCTACGCT

GGCCGCGGCGAGTCGAATTCTACTGCGCCCTCATATGAGCACGCTGCGTTCTTCGCTGTC

CCGACTGACGACCATGACCATGAGCGGGTTTGTCTCACCGACGCCATGCGCGAGCATCGC

TGGTCTCGCGATAGTGGCCAGGGCACAGAGAGTAACTCGTCTATCCATAGCCCTGGATCG

TCTTGTGGGTCGCCGACTGAATACGATGGATTCGAAAAGCCCGACGAACTGCCCGCAAGG

ACTCCCCCTCAACGCGGCTGGATTCATCGCACGGGCGTAGGTCGCTTCCTGTCCAAGACC

GTCCCTGGTCTAATTCCCGGCCGGGTTCTCCACGTTCTGAATGTGGTGTATAACATCGTT

GACCGTGTCATTCTGCCATTCGGATTTGTGGCCATTGCAACTGGGGCTGTGACGTACGGG

GGTATCATGCGGAGTCGTGAGATCTTCAATGGGCTTGCACACTTCATCAAGGGTGGCATC

TTTTTCTGGTACGGTATCCTGACACTTGGACGCTATGTTGGCTGCTGGGCAGATTTGGGT

TGGGCCTGGAATAAGAAGCCCTCTGCGTCGATTGTTGGTTGGAAAGCCAAAGTTCCCTCG

GGTGAAGCTACAGAGTCCTTCGTCATCTTCCTATATGGCGCCTCAAATGTTTTTCTCGAG

CACCTCTCCGGTACGGGCAAGGCATGGTCTGCTACAGATCTCGAACACGTCTCGATTTCC

GTGCTGTTCTTCGGCGGCGGTCTGGCCGGTATACTGTTCGAATCTACTTGTATCCGGGAC

TGGGTCAACACCACCATCCTCCAACCCCCTGCTCATGCTACCTCGGATGAAGCCTGGACG

CCCCCGAGGTCGCAGGGTGTTTCATTGAACCCCATGCCAGCCCTAGTCATCATGTTGCTT

GGCATGATGATGGGGTCGCACCACCAAGATAGTATGACATCTTCTATGGTCCACAAGCAG

TGGGGTAACATGTTGGTTGGATTTGCGCTGGCGCGCGGGATGACCTATGTTATGTTGTAC

CTCAAGCCACCGACTTCGTACCTCCCGGCTCGTCCTCCGACCGAGATCATTGCTGCATTC

TGCTTGATTTCAGGAGGGCTGATTTTCATGATGAGTACCCGCAATGTGGTTCAAGCGATG

GAGTACTACAAACTGGACGCAATGTTCACCTTCACTGTCGGACTGGGCTTCAGTGCTTTT

ATCATGGCTTATGAAATCTTAATGATTGCCATCAAGGCCTGGGCTGTGAAGCGCGCCCAG

CGCTCACGGCCAGACTTCCGCTTCAAG

>CL43.Contig1_All 74 988 Pyrroline-5-carboxylate reductase [Penicillium digitatum Pd1] >gi|425769792|gb|EKV08274.1| Pyrroline-5-carboxylate reductase [Penicillium digitatum PHI26]

ATCTATGGTGTGCGCACTCTCAGCTCCACAATTTTCGACTACCGAGTTTCCCGCATCCAA

CGCCGCCTTGATGACTTCCAGAAACAGCGCGAGGAGACCATCGAAAAGCTCAAAGTCGCA

ACCAAGTATACCTCCACCCAGCAACTACTAGAGAAGTACGGCAACGAGTCCCCCAAGCCC

TCTCCGGGCTCAAAGGAACAAGCCGAGAAAGAAAAGCCAGCCCAACAGCCACAGTATGTG

GCTCGCACTGGTCTCCCGCCGCCGCCCACTGCCAATATTCGTCCTCCTCCCTCCGCACCG

CAAACCCCGAATGACCTGCCATCCCTTAGTTATCCCTCTCGCCCTCTCGAGTTAACCCAA

AGTGCCCCGCAGACCCCACAGCAGCCCCTTTTTCGACCTTCTTTCCCTCCCCAAACACCC

ACCGATCAAACCAGCTTTGCACCGAACGCATTTCCTCAGAACAGCGAATACATCGAACAA

CCACATTGGTACGACCGCCTCTTGGATGTTCTGCTTGGAGAAGATGAAACCCAGCCCCGC

AACCGGATGGTGATGATGTGCACCGCCTGTCGGCTCGTGAACGGCCAGGCTCCCCCTGGG

ATCAAAACACCGGAAGAGCTCGGCCGCTGGCGGTGTTGCAGCTGTGGGGCTTGGAATGGC

ATGGAAAGCGAGACAACAAAGATTCTCAACAATTTGCGCCAAGATGCTGTGCCAGCCGAG

GGAACTTGGGAGCCTGTCTCGAAGGCTGACGCGGATACCCAGTCCTCCGAGGGAACGGAA

GAAGGTGTGATGGTGGCTTCCAGTGAAGAGGACCAAGTGGACTCTATTGGCTCCGATGCG

GAGGACCAGAAGAAAGAGGAACCTAAGGCTGCCCCTGTGCGGCGGTCCAAGCGCGGTGCA

AAGGGAAGCAAGGCA

>CL43.Contig2_All 59 1189 Pyrroline-5-carboxylate reductase [Penicillium digitatum Pd1] >gi|425769792|gb|EKV08274.1| Pyrroline-5-carboxylate reductase [Penicillium digitatum PHI26]

GCTTTCGAGAAAACCCTATCCACGCTCTCGACGAAGATCGCCCAGGCCACCACGCGCCTC

GAACAGCAACGTCAGTCTTCCCGGCGCATCAAGGCTCTCTGGACGCTCTACTCTACATTT

GCCTATCTCTTTTACTCAATCATTCTCGCCCTGGTGCTGGGGTGGGAGAGCTGGGGAATC

AAGGAATATGCAGCCATCGCAGGCGGGCCGGTCCTGATCTATGGTGTGCGCACTCTCAGC

TCCACAATTTTCGACTACCGAGTTTCCCGCATCCAACGCCGCCTTGATGACTTCCAGAAA

CAGCGCGAGGAGACCATCGAAAAGCTCAAAGTCGCAACCAAGTATACCTCCACCCAGCAA

CTACTAGAGAAGTACGGCAACGAGTCCCCCAAGCCCTCTCCGGGCTCAAAGGAACAAGCC

GAGAAAGAAAAGCCAGCCCAACAGCCACAGTATGTGGCTCGCACTGGTCTCCCGCCGCCG

CCCACTGCCAATATTCGTCCTCCTCCCTCCGCACCGCAAACCCCGAATGACCTGCCATCC

CTTAGTTATCCCTCTCGCCCTCTCGAGTTAACCCAAAGTGCCCCGCAGACCCCACAGCAG

CCCCTTTTTCGACCTTCTTTCCCTCCCCAAACACCCACCGATCAAACCAGCTTTGCACCG

AACGCATTTCCTCAGAACAGCGAATACATCGAACAACCACATTGGTACGACCGCCTCTTG

GATGTTCTGCTTGGAGAAGATGAAACCCAGCCCCGCAACCGGATGGTGATGATGTGCACC

GCCTGTCGGCTCGTGAACGGCCAGGCTCCCCCTGGGATCAAAACACCGGAAGAGCTCGGC

CGCTGGCGGTGTTGCAGCTGTGGGGCTTGGAATGGCATGGAAAGCGAGACAACAAAGATT

CTCAACAATTTGCGCCAAGATGCTGTGCCAGCCGAGGGAACTTGGGAGCCTGTCTCGAAG

GCTGACGCGGATACCCAGTCCTCCGAGGGAACGGAAGAAGGTGTGATGGTGGCTTCCAGT

GAAGAGGACCAAGTGGACTCTATTGGCTCCGATGCGGAGGACCAGAAGAAAGAGGAACCT

AAGGCTGCCCCTGTGCGGCGGTCCAAGCGCGGTGCAAAGGGAAGCAAGGCA

>CL44.Contig1_All 139 840 minus strand Glutathione S-transferase, putative [Penicillium digitatum Pd1] >gi|425776917|gb|EKV15114.1| Glutathione S-transferase, putative [Penicillium digitatum PHI26]

ATGCCGTTGAAAATCCACCACCTTGGCCGCTCGCAATCAGAGCGTATCGTCTGGCTCGCT

GAAGAACTTGGCATCAAGTATGACTATGTCTTCCACAAGCGCGACCCTTTACTCGCCCCG

CAATCAATCAAAGACCTGCATCCCTCTGGCACCGCACCGGTGATCGAAGACGACAACTCT

CCCTTTACGAAAAACAAGGTCGTGCTTGCTGAATCAGGCGCTATCATTGATTACATCATT

GCCGCCTACGGAGAGGGCCGCTTCGCGCGCACACCGAAGGATGGCGAGGAATACATCCAA

TTCCTGCAGTGGTACCACTGGGCAAATGCCTCCCTGCAGCCAGCCTTGCTCCGTGTGTTC

ATGAACAAGCGCTCGTCCGATGACCCAGAAGCACCGTTCGTCAAGCTGGCCGACACGAAA

CTAGAGTGTGCGTTCTCAATGATCGAGGCGAGACTCGGTGAAACCGGATCTTACTTGGTT

GGGAATGACGTCACCGCAGCGGATATCATGGCGGTCTTTACTCTCACTACCATGAGGGGA

TTTTCCCCTATTGATTTTGATGAGCAGAAACACAAGAACATTCTGGCTTATCTCAAGCGT

GTTGGTGAGCGGCCTGCTTATCAAAAGGCTATGAAGATTTGTGAGGGTGAAGATTTCGTG

CCTCTTCTTGGTGCGAAGGCTGAGCAGTTTGTGTTCCCCTAT

>CL44.Contig2_All 1 225 minus strand Pc22g23120 [Penicillium chrysogenum Wisconsin 54-1255] >gi|211593222|emb|CAP99600.1| Pc22g23120 [Penicillium chrysogenum Wisconsin 54-1255]

GTTGGGAATGACGTCACCGCAGCCGATATCATGGCAATCTGTACTCTCACCACCATGAGA

GGGTTCTGCCCCATTGAGTTTGATGAGCACAAGCATAAGAACATCCTGGCTTATCTTAAG

CGTGTTGGGGAGCGGCCTGCTTACCAAAAGGCTATGAAGATCTGCGAGGGTGAGGATTTC

GTGCCTATTCTTGGTGCAAAGGCTGAGCAGTTTGTATTCCCTTAC

>CL45.Contig1_All 620 1678 minus strand Nuclear protein export protein Yrb2, putative [Penicillium digitatum PHI26]

ATGACCGCCGGCGGAAACGAGGATGAGTCTTCACAAACCCTCAATAAGAAACGGAGCCGA

GAAGAGCTTGAGGATGGTGCCTCGAAGAACTCCTACATTGCGACTGAGCCCGCACCCGGC

TTGACTGAGACAACTTCGGACGAAAAATCTACTGCCGAAGGACAACCCGAGAAGAAGAGA

CCTCGAGACAATTCGGAGGAGCGCAAGGCTAAGGTGGACCAGACTTTCACTGCGAGTGCA

TTCGGAATGGCTGCTGCTGCCAACGCACCTTCACCTTTCGCAATGCCTGCAACTAAGTTG

ACATCTGAAACTTCGCCTTTTGCAACCAGCGGTGCCTCGACTTCTGGTTTCGGTACCCTC

GGATCCGGTTTCTCTGCATTTGGCAGCGCTTTCCCGGCTTTGTCTGGTAAACTCACCAGT

TTCGCGTCTCCTAATGCTCCCTCCGCTTTCCCCGGAACCGCCGGTAAACTGGCCAGTTTC

GCATCTGCCAATACCCCCACCAGCTTCTCTGGCGCAAGTGGTAAACTGACCAGCTTCGTG

TCTCCGAATGCCCCCGTTTCCTTTGGCGAGTCTAGTGATAAGACCCTTGGCGCCAAACAA

TCCGACAACGAAGATAGTGACAATGAACCAGTTGGTGAAACGGATGACACCTTTGTTGCC

GAGAAGACCGACAAGCGTTTCCACGCACAAACTGTTGAAACCGGCGAAGAAAATGAGAGC

ACCGAGTTCACAGCTAAGGCCAAGCTGTATTATTTTGACGACAAGAAATGGAAGGAGCGT

GGTACCGGCACCTTCAAGGTTAACCTCAAGACGGAATCCAACGGAAAGAAATCTGGCCGC

ATTATCATGCGTGCCGATGGTGCCCTGCGTGTTATGTTGAACAGTGCGGTTTGGCATAGC

ATGCCCTTTGGTGATGCCAAGAGTTCTCGCCCGACCACTCGGGATATTTATCTCGCCAGC

AACGAAGACGGGAAGGTCGTGAGCCTTCTTCTCCGGCTCGGTAATGAGAAGCAGTCCGGC

GAATTGTTTGATGTACTAAAGGATATCATGGAGCAAATT

>CL45.Contig2_All 560 1618 minus strand Nuclear protein export protein Yrb2, putative [Penicillium digitatum PHI26]

ATGACCGCCGGCGGAAACGAGGATGAGTCTTCACAAACCCTCAATAAGAAACGGAGCCGA

GAAGAGCTTGAGGATGGTGCCTCGAAGAACTCCTACATTGCGACTGAGCCCGCACCCGGC

TTGACTGAGACAACTTCGGACGAAAAATCTACTGCCGAAGGACAACCCGAGAAGAAGAGA

CCTCGAGACAATTCGGAGGAGCGCAAGGCTAAGGTGGACCAGACTTTCACTGCGAGTGCA

TTCGGAATGGCTGCTGCTGCCAACGCACCTTCACCTTTCGCAATGCCTGCAACTAAGTTG

ACATCTGAAACTTCGCCTTTTGCAACCAGCGGTGCCTCGACTTCTGGTTTCGGTACCCTC

GGATCCGGTTTCTCTGCATTTGGCAGCGCTTTCCCGGCTTTGTCTGGTAAACTCACCAGT

TTCGCGTCTCCTAATGCTCCCTCCGCTTTCCCCGGAACCGCCGGTAAACTGGCCAGTTTC

GCATCTGCCAATACCCCCACCAGCTTCTCTGGCGCAAGTGGTAAACTGACCAGCTTCGTG

TCTCCGAATGCCCCCGTTTCCTTTGGCGAGTCTAGTGATAAGACCCTTGGCGCCAAACAA

TCCGACAACGAAGATAGTGACAATGAACCAGTTGGTGAAACGGATGACACCTTTGTTGCC

GAGAAGACCGACAAGCGTTTCCACGCACAAACTGTTGAAACCGGCGAAGAAAATGAGAGC

ACCGAGTTCACAGCTAAGGCCAAGCTGTATTATTTTGACGACAAGAAATGGAAGGAGCGT

GGTACCGGCACCTTCAAGGTTAACCTCAAGACGGAATCCAACGGAAAGAAATCTGGCCGC

ATTATCATGCGTGCCGATGGTGCCCTGCGTGTTATGTTGAACAGTGCGGTTTGGCATAGC

ATGCCCTTTGGTGATGCCAAGAGTTCTCGCCCGACCACTCGGGATATTTATCTCGCCAGC

AACGAAGACGGGAAGGTCGTGAGCCTTCTTCTCCGGCTCGGTAATGAGAAGCAGTCCGGC

GAATTGTTTGATGTACTAAAGGATATCATGGAGCAAATT

>CL45.Contig3_All 251 1309 minus strand Nuclear protein export protein Yrb2, putative [Penicillium digitatum PHI26]

ATGACCGCCGGCGGAAACGAGGATGAGTCTTCACAAACCCTCAATAAGAAACGGAGCCGA

GAAGAGCTTGAGGATGGTGCCTCGAAGAACTCCTACATTGCGACTGAGCCCGCACCCGGC

TTGACTGAGACAACTTCGGACGAAAAATCTACTGCCGAAGGACAACCCGAGAAGAAGAGA

CCTCGAGACAATTCGGAGGAGCGCAAGGCTAAGGTGGACCAGACTTTCACTGCGAGTGCA

TTCGGAATGGCTGCTGCTGCCAACGCACCTTCACCTTTCGCAATGCCTGCAACTAAGTTG

ACATCTGAAACTTCGCCTTTTGCAACCAGCGGTGCCTCGACTTCTGGTTTCGGTACCCTC

GGATCCGGTTTCTCTGCATTTGGCAGCGCTTTCCCGGCTTTGTCTGGTAAACTCACCAGT

TTCGCGTCTCCTAATGCTCCCTCCGCTTTCCCCGGAACCGCCGGTAAACTGGCCAGTTTC

GCATCTGCCAATACCCCCACCAGCTTCTCTGGCGCAAGTGGTAAACTGACCAGCTTCGTG

TCTCCGAATGCCCCCGTTTCCTTTGGCGAGTCTAGTGATAAGACCCTTGGCGCCAAACAA

TCCGACAACGAAGATAGTGACAATGAACCAGTTGGTGAAACGGATGACACCTTTGTTGCC

GAGAAGACCGACAAGCGTTTCCACGCACAAACTGTTGAAACCGGCGAAGAAAATGAGAGC

ACCGAGTTCACAGCTAAGGCCAAGCTGTATTATTTTGACGACAAGAAATGGAAGGAGCGT

GGTACCGGCACCTTCAAGGTTAACCTCAAGACGGAATCCAACGGAAAGAAATCTGGCCGC

ATTATCATGCGTGCCGATGGTGCCCTGCGTGTTATGTTGAACAGTGCGGTTTGGCATAGC

ATGCCCTTTGGTGATGCCAAGAGTTCTCGCCCGACCACTCGGGATATTTATCTCGCCAGC

AACGAAGACGGGAAGGTCGTGAGCCTTCTTCTCCGGCTCGGTAATGAGAAGCAGTCCGGC

GAATTGTTTGATGTACTAAAGGATATCATGGAGCAAATT

>CL46.Contig1_All 122 634 Pc21g01130 [Penicillium chrysogenum Wisconsin 54-1255] >gi|211588895|emb|CAP95010.1| Pc21g01130 [Penicillium chrysogenum Wisconsin 54-1255]

ATGGCCTCTTTCCAGGAGCGTGCCCAGCACTCTATCGCTCAGCTTGACAAGGAGCTCTCC

AAGTACCCTGTCCTTGCCAACCTTGAGCGTCAGACCAATGTCCCCAAGGTCTATGTCGTC

CTGGGCCTTGTTGGAATCTACTTCTTCCTGGTCTTCTTTAACATCGCGGGCGAGTTCCTC

GTCAACTTTGCTGGTTTCCTGGTCCCCGGTTACTACTCCCTGCAGGCGCTCTTCACCTCG

AGCACCAAGGACGACACCCAGTGGCTCACGTACTGGGTTGTCTTCGCCTTCCTGACGGTC

ATTGAGAGCGCTATCAGCGCCGCTTACTGGTTCCCCTTCTACTACATCTTCAAGTTCGTC

CTGATCATGTGGATGGCTCTTCCCCAGACCAGCGGTGCCCAAATTGTCTTCCACTCCTTC

CTCCAGCCCGTGGTTGGCCGCTTCTTCTCCAACAACGGTACCTCCTCCAACCTCCGTGCT

CAGGCCGAGGCTGCGAGCAAGGCCCACTCCACC

>CL47.Contig1_All 2762 2908 minus strand hypothetical protein PDIG_07800 [Penicillium digitatum PHI26]

CGCACAAGCCCGATACGAAGAACAAACGAACCGTCACCGCGCCCCTGCCCGCCGATACCG

TCCTGGACAACTGGTCTGGCTGAACGCCAGGAATATCCGAACCCTCCGCCCGCAGAAGAA

ACTGGACTGGAAGAACCTAGGCCCCTT

>CL47.Contig2_All 2831 2977 minus strand hypothetical protein PDIG_07800 [Penicillium digitatum PHI26]

CGCACAAGCCCGATACGAAGAACAAACGAACCGTCACCGCGCCCCTGCCCGCCGATACCG

TCCTGGACAACTGGTCTGGCTGAACGCCAGGAATATCCGAACCCTCCGCCCGCAGAAGAA

ACTGGACTGGAAGAACCTAGGCCCCTT

>CL47.Contig4_All 2711 2857 minus strand hypothetical protein PDIG_07800 [Penicillium digitatum PHI26]

CGCACAAGCCCGATACGAAGAACAAACGAACCGTCACCGCGCCCCTGCCCGCCGATACCG

TCCTGGACAACTGGTCTGGCTGAACGCCAGGAATATCCGAACCCTCCGCCCGCAGAAGAA

ACTGGACTGGAAGAACCTAGGCCCCTT

>CL47.Contig5_All 2642 2788 minus strand hypothetical protein PDIG_07800 [Penicillium digitatum PHI26]

CGCACAAGCCCGATACGAAGAACAAACGAACCGTCACCGCGCCCCTGCCCGCCGATACCG

TCCTGGACAACTGGTCTGGCTGAACGCCAGGAATATCCGAACCCTCCGCCCGCAGAAGAA

ACTGGACTGGAAGAACCTAGGCCCCTT

>CL47.Contig6_All 149 1588 hypothetical protein PDIG_59720 [Penicillium digitatum PHI26]

ATGGTTTCGAGAATCGTTGAAGAGCTCTACAAGGATCCACAACTTCGACGCACTTTCCGT

CTAAGAGGGTCGATCCGTTTCGAAAACCATTCTAATACACTGAGCCCAGCACGCGAACTA

GAAGAAGGGTTGAAGAGTATGAATATTCACGGTCGCTCGGCACCGCGGCGCTCACCGCGT

ATAGCTGCCCGAGAGAAGACGCCATCCAGTGCGGCTGCACGTGAGCCTAAAACTCCGCTG

CCAGCAATCAAACGACCTCAAGCTGACCAATTTTGCGTCTATAATATTCCAATCGAATTC

TCAGAATCAACACATCGCGTCGCTGCTTATATCAAAGAATACAAATCTCCACACAATGTG

ACTTTGGGCCATATATACGAGGGATTGGGAGATATGGATGTTGATGAAGTGATACAGCAG

AAAGATGATGAGTCTCTGAAATCTCGCTTTCAGCGAGCTCTTATTGCACTCCTCTCGCAG

CCCTTTGACTATATGGTACGCGCTGGTACGCAGGTAGGAGTCCTCAGCACTGGCGAAGCC

GATATCTATCTGCGAATTGACGATGACCCTTCAACACTTTTCTACCACCTTTCCGTGCCA

AAAGGAGACGTAGGAAATGAGACAGGGTGGGACTCTCACTCGGATTGCCCTAATCGCTTA

CACTTGACTGCCGTAGGTCAGTCTCTCGCCTTTACTCTCCAGGCGCTACGCTTGCAGCCC

CGGAATCAGGCTTGGAGGTGGCAAGCAATCGATAACCTGCCAAAGTGGGAAATTGTACTT

GCGGGAATTCTGGACGGTATTTCAGAAGATGACGTGCCATCTTCCGAATATCGTCCCCCA

AAAGCGAATAACTCCTTCGTGCGATCTCCGGTCCAACTGCGTCGCAGGAGGCTTAAATCC

AACACGGATGGTTTTCGACCCTCGAGCACGCATAGTAGTTCTGATGACGACGGGGATGAC

CCCAATACACCAAGCCGACGTTTGCACGTTCCTCAGGGCCTCGAAAGAAGGCAACGAGTA

GCAATTGGCCAACAGCGTGACCCAGCCAAGGCAGGGTCATTTTGCGAGGATAATCATCGG

CGCTACTGCACGTTAAAATGCCTCCGTGGTCTAGTAAATCGAGGGGCTTTGAACAGGGAA

TTAGACCGGGCTTGCCCCAATGTCATTAATCATGGAAAAACGAAGCACATTCTGACCTTG

CAATCCTTCATCAAGCTGTTGCAGCGTCAACTATCCGAGACCGTCAATGCAGACTGTGAG

ACGCTTGGTATCCATGGTTCCCGAGGTGCTCTTTTGAAAGTGACGCTCTCATCACACGGG

TACACCGCGCCCGCGAAATGCACGGTTCCAGAGTTCCGCGACTTCCTTCGCCATGAAGCA

GCTGTATACAACATCCTTCGTCCGATCCAGGGAGTATGTCACGGTGCGAACCGTGATGCT

>CL48.Contig1_All 2143 2262 DNA replication licensing factor mcm6 [Ajellomyces dermatitidis SLH14081] >gi|239590311|gb|EEQ72892.1| DNA replication licensing factor mcm6 [Ajellomyces dermatitidis SLH14081]

GACAAAATTCTGCTGCGTATCTCCGGGTCTGGTCTCATGGATGGCACTGGCGATGTCGCT

CAGCAAACTGACGGAAAGGTCCTTTTCGTCATGCATCCCGATTGCGCTTTCGAGGAGATG

>CL48.Contig2_All 53 172 DNA replication licensing factor Mcm6, putative [Penicillium digitatum Pd1] >gi|425773423|gb|EKV11776.1| DNA replication licensing factor Mcm6, putative [Penicillium digitatum PHI26]

GACAAAATTCTGCTGCGTATCTCCGGGTCTGGTCTCATGGATGGCACTGGCGATGTCGCT

CAGCAAACTGACGGAAAGGTCCTTTTCGTCATGCATCCCGATTGCGCTTTCGAGGAGATG

>CL49.Contig1_All 2 232 minus strand Pc20g08540 [Penicillium chrysogenum Wisconsin 54-1255] >gi|211588112|emb|CAP86183.1| Pc20g08540 [Penicillium chrysogenum Wisconsin 54-1255]

GATGCGGATGATCGGACGATCAAACGTGCGTATCGCCAGTTAGTGAAGCAACATCACCCT

GATAAGGCCAACGCCCAAGGTGTAAGCAAGGAAGAAGCAGAGAAAAGGATGGCAGCCATC

AACGAAGCCTACGAGGTTCTATCAGACTCCGAACTCCGAACACGGTTTGACAACGGCGAC

GACCCCAATGATCCTGAATCGCAGCAGCGGGGCAATCCGTTCCAGGGTAAC

>CL49.Contig2_All 147 1688 minus strand hypothetical protein PDIG_19060 [Penicillium digitatum PHI26] >gi|425784136|gb|EKV21930.1| hypothetical protein PDIP_01550 [Penicillium digitatum Pd1]

ATGCTCGTTTCCTTGGAATCACTCGCGGCCTTTCTAGCTTGTCTCTCGTCTGGATACGCC

CTTCAAATTCCTTCAGATACACCTCTCTCTGAGTTGATCTCTTCCGCCAAGGCCCACCTC

GCGCAGGGCTCACCCCGAGATGCCGTGTTATACTTCGACGCCGCCGTTTCGCGCGACCCA

ACCAACTATATCACCATCTTTCAACGGGGTGCGGCATATCTTTCTATCGGCAAAAACTCA

CAAGCCTCAAGTGACTTTGATCGAGTTCTAGAACTCAAGCCGGATTTCGAGGGCGCTCTT

CTACAGCGATCTCGCCTAAACGCACGCTCCGCGCATTGGCAAGAGGCCTTGCAGGATCTC

GAGCGGGCCGGAAAGAAGTCGACGGATGATTACAGGGAATTAGAAGCCGCACGTGATGCG

GCCACCTTGGCTCTCAATGCCGAAAAGCAAGGCGCTTGGGAGACATGTGTCTCCGAGGCA

AATGTGGCCATCCTGAAGGCAAATACGGCGCTTCCTCTACGGCAGGCCCGAGCCCATTGT

CACTTTGAAAAGGGCGAGACGGAAGAAGGGCTTAGCGATTTAGCACATGTTTTGCAAATG

TCCCCGAGCTTGGTCGAACCTCACCTGCAAATGTCATCCATGCTGTTCTACTCTCTCGGA

GACAGTGACCGTGGCCTTACTCAAATTCGAAAATGCCTCCATGCTGATCCAGACTCGAAA

CCGTGCAACCGTCTCTATCGACGTGAACGGAAGCTTGCCAAGCAACTAGAAACATTGCAA

ACTGCCCTGGGCGCACACAAATTCAGCAATGCTGCAAAACTCATGGTCGGCGATGGTGAA

TCCGGTGGACTGATTATAGACGTCAAGGCCGATGTTGAAGAAGCAAGACAGGCTAACCAT

ATTCATCGCCTGGCACCAAATAATCTTTACACATTCCTAGTCGAGAAGACCTGCGAGGCT

TACCGTGAAATGCGTATGATCAAAAAGGCTGGTCCTTTTTGCGCCGAAGCTCTCCAGCTT

GTTCCTCACTCGCTTGCCGGGCTCCTGTATAAAGCACAGACTGCTTTGGACGAGGATCGA

TTCGAGGACGCCATACGTACGCTCGAATTAGCCAAGGAACATCATCCAAGTTCGGAAGAG

GCGCAATCCCTCCAACAAAAGGCACAGACCCTACTCAAGCGCTCCAAGCAGAAAGATTAT

TACAAGGTCCTTGGTATTAGCCGTGATGCGGATGATCGGACGATCAAGCGCGCGTACCGC

CAGCTAGTGAAGCAACACCACCCAGATAAGGCCAATTCCCAGGGTGTAAGCAAGGAAGAG

GCAGAGAAAAAGATGGCGGCTATTAATGAAGCCTATGAAGTTCTATCTGACTCCGAACTC

CGGACACGGTTTGACAACGGTGACGATCCCAATGATCCTGAATCGCAACAGCGAGGCAGT

CAGTTCCAGGGCAACCCATTTGGTGGCGGTGGTCAACAGTTCTTCTTCCAACAGGGTGGT

CCACAATTCCAGGGAGGTTTCAAGTTCCCTGGTGGCTTCCGC

>CL50.Contig1_All 19 192 hypothetical protein PDIG_75510 [Penicillium digitatum PHI26] >gi|425775706|gb|EKV13960.1| hypothetical protein PDIP_45980 [Penicillium digitatum Pd1]

ATGAAGACTATTTCAGTTGACCCGAGCAGGAACACGCCTCCAAACCGAGACCTTCCACTG

CTGACTACGACCCCGGCTGAACTTCCTTCAGCTAAGGTGACGAAGCTTCATCCTTTCAAC

CCCGGCGAGGAGAACGCAGCCCTGTTCTTTGTGGGAACAGCCACAACAATCATA

>CL50.Contig2_All 50 1099 hypothetical protein PDIG_75510 [Penicillium digitatum PHI26] >gi|425775706|gb|EKV13960.1| hypothetical protein PDIP_45980 [Penicillium digitatum Pd1]

ATGAAGACTATTTCAGTTGACCCGAGCAGGAACACGCCTCCAAACCGAGACCTTCCACTG

CTGACTACGACCCCGGCTGAACTTCCTTCAGCTAAGGTGACGAAGCTTCATCCTTTCAAC

CCCGGCGAGGAGAACGCAGCCCTGTTCTTTGTGGGAACAGCCACAACAATCATAGAATGG

AGTGGAGTACGGATTTTGACTGATGTCAGCTTTTGATGGGGACTCTAATTCTCCCAGACT

GACTGTGTTAGCCCAACTTCCTCCATACAGGCGACCCTGTCCACCTCGGTCCAGGCGGGC

CAAGTGAGCGCCTCACCGACCCAGCTATCGACCTGCATGACCTCCCTCGGATTGATCTGG

TCCTTCTCTCCCATTATCACGAGTAGGTCTCAGAGGCTCAAGGTTATCGAGCGGATCACT

AAGATTGATCCTGCCAGGGACCACTTTGATGACAGGGTCGAGGCTTCCTTGAGACGTGAT

CTACCTATTATCACTACACCACATGCAGAGAAGCATCTCACTTCTAAGCAACAGGACCCG

TTTACATCCGTATCCGTGCTCAATCCATTTGAGCAGATCAATGTCAGCATTCAAGGTACG

GAAGGGCCAGGACAGCCCCGACTGCGTGTAGCGGGAATGCCCGGGAAGCATGTGCCTTCT

AACCGAGTGGTAGAGAAATTCAACACAGTAGCCAATGTGTTTCCGCCAACCAACGGCTGG

ATGCTTGAGCTAGGCCACGGCAGCACCAACCCTGCAGACTTTTCCTGTGGTTATCGCATC

TACATCTCCGGAGACACCCTCATGTTCGACGAGCTCCGGAGAATCCCCGAACGATACGCA

GGCCAACCAATCGACCTGATGCTCGTCCACCTCGGCGGCATGACCGTTCCTTCTACCTTG

GTAGGAAGACTGATGGAACCGCTAGCGTTGACGGTGACTATGGATGCGGAGCAGAGTCTG

TCATTGATTCAGTTGATCCAACCGAACATCATCATCCCGATTCACTATGATGACTACGAC

GGATTTGTGAGCCCACTAGAAGATTTCAGG

>CL51.Contig1_All 2173 2478 Monoacylglycerol lipase ABHD12 [Penicillium digitatum PHI26] >gi|425781479|gb|EKV19441.1| Monoacylglycerol lipase ABHD12 [Penicillium digitatum Pd1]

GAGGAAACTCTCTGCCTTATTGCAGAAGGTCTGGATCACCAGCGTGATCGCTATCCCCTA

GTTCTGTCATGTCGTCGCCTGTACACAGTTCTTCTCCCAACTCTCTACTCCCGGGTTATT

CTTGGTGACGTTAGCAAGCATACCATCGGCCAATTCAGTGGATTTTTCAACGCAATCGCC

CGACGGCCGCAGTTGGCGAGCGCTGTGCGAGCTTTACGCTTGGAGAATTGGGATACCGAG

GATAGCACTGAGGACATCGACTGCGAATTCGAGTATGATGGAGAGCTTATTGACGGGCTT

GTCGCT

>CL51.Contig2_All 2173 2478 Monoacylglycerol lipase ABHD12 [Penicillium digitatum PHI26] >gi|425781479|gb|EKV19441.1| Monoacylglycerol lipase ABHD12 [Penicillium digitatum Pd1]

GAGGAAACTCTCTGCCTTATTGCAGAAGGTCTGGATCACCAGCGTGATCGCTATCCCCTA

GTTCTGTCATGTCGTCGCCTGTACACAGTTCTTCTCCCAACTCTCTACTCCCGGGTTATT

CTTGGTGACGTTAGCAAGCATACCATCGGCCAATTCAGTGGATTTTTCAACGCAATCGCC

CGACGGCCGCAGTTGGCGAGCGCTGTGCGAGCTTTACGCTTGGAGAATTGGGATACCGAG

GATAGCACTGAGGACATCGACTGCGAATTCGAGTATGATGGAGAGCTTATTGACGGGCTT

GTCGCT

>CL52.Contig1_All 185 703 minus strand Peptidyl-prolyl cis-trans isomerase [Penicillium digitatum Pd1] >gi|425771115|gb|EKV09569.1| Peptidyl-prolyl cis-trans isomerase [Penicillium digitatum PHI26]

ATGTCCAACACCACTGCTTTCTTCGAGGTCCAGTACGCCCCTGCTGGCTCCTCTACCCCT

AAGATTGGTCGCATCAACTTCAACCTCTTCGCCAACGAGGTTCCCAAGACCGCCAAGAAC

TTCGCTGAGCTTTGCAACGCCAAGCAGGGTGAGGGCTACAAGGGCTCTTCCTTCCACCGT

GTCATCCCCCAGTTCATGCTCCAGGGTGGTGACTTCACCCGTGGCAACGGTACCGGTGGC

CGCTCTATCTACGGCGAGAAGTTCCCCGATGAGAACTTCATCCACAAGCACACCCGCCCT

GGTCTCCTGTCCATGGCCAACGCTGGCCCTAACACCAACGGCTCCCAGTTCTTCATCACC

ACCGTTGTAACCTCGTGGCTCGATGGTAAGCACGTCGTCTTCGGCGAGGTCGCTGATGCC

GAGTCCATGAACGTTGTTAAGGAGATCGAGGCTCTCGGTTCCTCTTCCGGCTCCATCCGC

TCCGCTGTCAAGCCCACCATCGTCAACTGCGGTGGCAAC

>CL53.Contig1_All 3 242 minus strand Pc21g18310 [Penicillium chrysogenum Wisconsin 54-1255] >gi|211590536|emb|CAP96728.1| Pc21g18310 [Penicillium chrysogenum Wisconsin 54-1255]

CGTGGCTTTGGCTTCGTCAACCTTGCTTCGGAGGAGCTCCAGGCCAAGGCCGTCAGCGAG

ATGAACGGCAAGGACATTGATGGTCGCGTCATCGCTGTGAAGGTCGCCATTGACAGCCCC

GGTAAGGAAGATGAAGATATCAACGCTGTGGCTGAGACCGAGGAGACTGCTCCTGCTTCT

GCCCCTGCCCCTGCTGCTCCTGCTCAGGAGAACGCTGCTCCTGCCGTCACTACTCAGGCT

>CL53.Contig2_All 256 1341 hypothetical protein PDIP_50840 [Penicillium digitatum Pd1] >gi|425776456|gb|EKV14673.1| hypothetical protein PDIG_31260 [Penicillium digitatum PHI26]

ATGTCCGACGTCGACGCACTTGCTGACTCCGTGGCTGCGACCACTCTGACCGATAAGCCC

GAAACCAACGGTGCTACCCCTTCCAACCAGGCTGCCGATGCCGCGGCCGCCAGTGCAGAT

GAGGGTCGTCGTCTTTACATCGGGAACCTCGCATACGCGACCACCGAGGAGGAGCTCAAG

GAGTTCTTCAAGACCTACACCATCGAGACCACCTCGATCCCAGTGAACCCCCGCACCAAC

CGCCCCGTTGGCTATGCATTTGTGGACATTGCTACTGCCGCGGAAGCTTCTGCCGCTATC

GAGGCCCTCTCCGGCAAGGAGATTCTTCAACGTAAGGTGTCCGTGCAGCTTGCTCGCAAG

CCCGAGCCCGCCGAGGCCAAGGAAGGTGCTGCTAGTGGCGGCGAGGGTGCCAGCGGTACT

GAGGGTCGCAAGCGTGCTGGTGGTCGCGGCCGTGGCCGTGGCCGTGCCCGCGGTCGTGGT

GGCCGTACCGGCCGTAGCCGTGCTGCCCAGGATGGCCAGGAAGCCACTGAGGCCCCTGTC

AGCGAAACCCCTCTTGCTGACACTACAAACGAGAAGGACATTGCCCCCAAGGCCGGCGAG

GCCCGCCCCGCACGCCCCCAGAAGCAGCGCGGTCCTCCCGAGGATGGCATCCCCTCTAAG

ACTAAGGTGATGGTTGCCAACCTGCCGTACGACTTGACTGAGGATAAGCTCAAGGAGATC

TTCGCCGCCTACGAGCCCGTCTCTGCCAAGGTTGCTCTGCGTCCTATCCCCCGTTTCATG

ATCAAGAAGTTGCAGGCTCGCAACGAGCGCCGCAAGACCCGTGGATTCGGCTTCGTCAAC

CTGGCCTCAGAGGAGCTCCAGGCCAAGGCTGTCAGCGAGATGAACGGCAAGGATATTGAC

GGCCGTGTTATCGCCGTGAAGGTTGCCATTGATAGCCCTGGTAAGGAGGACGAGGATGTC

AACGCTGTGGCCGAAACCGAGGAGACTGCTGCTTCTGCTGCTGCTCCTGCTACTGCCCAG

GAAAACGCTGCTCCTGCTCCTGCTCCTGCTGAAGAGAACGCTGCTCCTGCCGTCACCACT

AAGGCT

>CL55.Contig1_All 936 1202 Pc13g00800 [Penicillium chrysogenum Wisconsin 54-1255] >gi|211583147|emb|CAP91149.1| Pc13g00800 [Penicillium chrysogenum Wisconsin 54-1255]

GATGACGCATATTTCAGTTTACGCGGTGCACACGTCGAGGCTTTACAGTCGATCTTCAGC

ACGGAAGTATGCAGAGGCATAGATGAAAGCCAATTGAGAACATGGGAGAGAGAGCAGCTG

CTTGTGGATACAACGGACTGTATTACAATGCAGTTATGGCGGGGGCAGCCGCATCTTGGA

ATTATCCGGCTGCGAATCGGATTCTATGCGGGATCTCATTTAGCCAATGTTTTGTATCAA

GAAGCTCCTCGTATTAGTTTACCTTCA

>CL55.Contig2_All 98 1147 Pc13g00800 [Penicillium chrysogenum Wisconsin 54-1255] >gi|211583147|emb|CAP91149.1| Pc13g00800 [Penicillium chrysogenum Wisconsin 54-1255]

TTCACTCCAGTATACACGAAATACGACGAAATCAAATTGAAAAGGGCAGTTTCCGAGAAT

GACCCCGAAATACTCGGCGAGCGCTTAAGCATCGCCTGCTCCACAGCACCAAAGAAATGT

AATTACAGGTTACAGCACCAACGAAAAGCGGCCCGAAGAATCTACCTACTGGTTTTTGAG

GAAGATCCGAACATATTTATCCCATTCATCCTCTCCGTTTCACCGAGAGCTTGCGAGCAC

TTCAATTTCTCGATTTTCAAGTCTCAGCACGAGAAACGAACCAAAATCATGTACCATGAA

TGTGCTTTGTTCCTCATTTGGAGGGTGGCCCGTGGGCATGGCATTGAGAAGACGAACATT

TTCATCAAATTGGTACAGTGGCACATGCAAACATCCCCGCCAGTGACGGGAGCTGAAGGC

AAGGAGGACCAATACTGGTCGTTACGACTGTCTAGTTTTGCCGCAATTCAGAGTATGTTC

GGTGATGTCATTGCAGACGCAGTTCAACGTTTCCCACCAGAAGCAGAAATGAGTGTAGGG

GATTACTTACCAGAGAGTACAACCAACATTTGGACAAGGATCCCCCACCGAGAGCCTCAA

GATTCACTAATTTGCCTCTATGTTGGACGGGCTCATGAGATCGCCAAGGTGCTATTTCCA

ATTGGAAGCCAGAAAGTTGCCTCCGTTCTCGCGGCAGATGTTCCGAATGCAACCATGACA

AGTACTCCGCAGGCTGCTCTAGGTCCAGAGAGCGCTAATAAAACATCAACATGTTCCGAG

TTTGATGACGCATATTTCAGTTTACGCGGTGCACACGTCGAGGCTTTACAGTCGATCTTC

AGCACGGAAGTATGCAGAGGCATAGATGAAAGCCAATTGAGAACATGGGAGAGAGAGCAG

CTGCTTGTGGATACAACGGACTGTATTACAATGCAGTTATGGCGGGGGCAGCCGCATCTT

GGAATTATCCGGCTGCGAATCGGATTCTATGCGGGATCTCATTTAGCCAATGTTTTGTAT

CAAGAAGCTCCTCGTATTAGTTTACCTTCA

>CL55.Contig3_All 92 790 hypothetical protein AFUA_5G14900 [Aspergillus fumigatus Af293] >gi|66850803|gb|EAL91129.1| hypothetical protein AFUA_5G14900 [Aspergillus fumigatus Af293] >gi|159124750|gb|EDP49868.1| hypothetical protein AFUB_079010 [Aspergillus fumigatus A1163]

CCTGGATTCACTCCAGTATACACGAAATACGACGAAATCAAATTGAAAAGGGCAGTTTCC

GAGAATGACCCCGAAATACTCGGCGAGCGCTTAAGCATCGCCTGCTCCACAGCACCAAAG

AAATGTAATTACAGGTTACAGCACCAACGAAAAGCGGCCCGAAGAATCTACCTACTGGTT

TTTGAGGAAGATCCGAACATATTTATCCCATTCATCCTCTCCGTTTCACCGAGAGCTTGC

GAGCACTTCAATTTCTCGATTTTCAAGTCTCAGCACGAGAAACGAACCAAAATCATGTAC

CATGAATGTGCTTTGTTCCTCATTTGGAGGGTGGCCCGTGGGCATGGCATTGAGAAGACG

AACATTTTCATCAAATTGGTACAGTGGCACATGCAAACATCCCCGCCAGTGACGGGAGCT

GAAGGCAAGGAGGACCAATACTGGTCGTTACGACTGTCAGAGAGTACAACCAACATTTGG

ACAAGGATCCCCCACCGAGAGCCTCAAGATTCACTAATTTGCCTCTATGTTGGACGGGCT

CATGAGATCGCCAAGGTGCTATTTCCAATTGGAAGCCAGAAAGTTGCCTCCGTTCTCGCG

GCAGATGTTCCGAATGCAACCATGACAAGTACTCCGCAGGCTGCTCTAGGTCCAGAGAGC

GCTAATAAAACATCAACATGTTCCGAGTTTGGTATGAAG

>CL55.Contig4_All 98 988 Pc13g00800 [Penicillium chrysogenum Wisconsin 54-1255] >gi|211583147|emb|CAP91149.1| Pc13g00800 [Penicillium chrysogenum Wisconsin 54-1255]

TTCACTCCAGTATACACGAAATACGACGAAATCAAATTGAAAAGGGCAGTTTCCGAGAAT

GACCCCGAAATACTCGGCGAGCGCTTAAGCATCGCCTGCTCCACAGCACCAAAGAAATGT

AATTACAGGTTACAGCACCAACGAAAAGCGGCCCGAAGAATCTACCTACTGGTTTTTGAG

GAAGATCCGAACATATTTATCCCATTCATCCTCTCCGTTTCACCGAGAGCTTGCGAGCAC

TTCAATTTCTCGATTTTCAAGTCTCAGCACGAGAAACGAACCAAAATCATGTACCATGAA

TGTGCTTTGTTCCTCATTTGGAGGGTGGCCCGTGGGCATGGCATTGAGAAGACGAACATT

TTCATCAAATTGGTACAGTGGCACATGCAAACATCCCCGCCAGTGACGGGAGCTGAAGGC

AAGGAGGACCAATACTGGTCGTTACGACTGTCAGAGAGTACAACCAACATTTGGACAAGG

ATCCCCCACCGAGAGCCTCAAGATTCACTAATTTGCCTCTATGTTGGACGGGCTCATGAG

ATCGCCAAGGTGCTATTTCCAATTGGAAGCCAGAAAGTTGCCTCCGTTCTCGCGGCAGAT

GTTCCGAATGCAACCATGACAAGTACTCCGCAGGCTGCTCTAGGTCCAGAGAGCGCTAAT

AAAACATCAACATGTTCCGAGTTTGATGACGCATATTTCAGTTTACGCGGTGCACACGTC

GAGGCTTTACAGTCGATCTTCAGCACGGAAGTATGCAGAGGCATAGATGAAAGCCAATTG

AGAACATGGGAGAGAGAGCAGCTGCTTGTGGATACAACGGACTGTATTACAATGCAGTTA

TGGCGGGGGCAGCCGCATCTTGGAATTATCCGGCTGCGAATCGGATTCTTC

>CL55.Contig5_All 98 1087 Pc13g00800 [Penicillium chrysogenum Wisconsin 54-1255] >gi|211583147|emb|CAP91149.1| Pc13g00800 [Penicillium chrysogenum Wisconsin 54-1255]

TTCACTCCAGTATACACGAAATACGACGAAATCAAATTGAAAAGGGCAGTTTCCGAGAAT

GACCCCGAAATACTCGGCGAGCGCTTAAGCATCGCCTGCTCCACAGCACCAAAGAAATGT

AATTACAGGTTACAGCACCAACGAAAAGCGGCCCGAAGAATCTACCTACTGGTTTTTGAG

GAAGATCCGAACATATTTATCCCATTCATCCTCTCCGTTTCACCGAGAGCTTGCGAGCAC

TTCAATTTCTCGATTTTCAAGTCTCAGCACGAGAAACGAACCAAAATCATGTACCATGAA

TGTGCTTTGTTCCTCATTTGGAGGGTGGCCCGTGGGCATGGCATTGAGAAGACGAACATT

TTCATCAAATTGGTACAGTGGCACATGCAAACATCCCCGCCAGTGACGGGAGCTGAAGGC

AAGGAGGACCAATACTGGTCGTTACGACTGTCTAGTTTTGCCGCAATTCAGAGTATGTTC

GGTGATGTCATTGCAGACGCAGTTCAACGTTTCCCACCAGAAGCAGAAATGAGTGTAGGG

GATTACTTACCAGAGAGTACAACCAACATTTGGACAAGGATCCCCCACCGAGAGCCTCAA

GATTCACTAATTTGCCTCTATGTTGGACGGGCTCATGAGATCGCCAAGGTGCTATTTCCA

ATTGGAAGCCAGAAAGTTGCCTCCGTTCTCGCGGCAGATGTTCCGAATGCAACCATGACA

AGTACTCCGCAGGCTGCTCTAGGTCCAGAGAGCGCTAATAAAACATCAACATGTTCCGAG

TTTGATGACGCATATTTCAGTTTACGCGGTGCACACGTCGAGGCTTTACAGTCGATCTTC

AGCACGGAAGTATGCAGAGGCATAGATGAAAGCCAATTGAGAACATGGGAGAGAGAGCAG

CTGCTTGTGGATACAACGGACTGTATTACAATGCAGTTATGGCGGGGGCAGCCGCATCTT

GGAATTATCCGGCTGCGAATCGGATTCTTC

>CL55.Contig6_All 92 889 hypothetical protein AFUA_5G14900 [Aspergillus fumigatus Af293] >gi|66850803|gb|EAL91129.1| hypothetical protein AFUA_5G14900 [Aspergillus fumigatus Af293] >gi|159124750|gb|EDP49868.1| hypothetical protein AFUB_079010 [Aspergillus fumigatus A1163]

CCTGGATTCACTCCAGTATACACGAAATACGACGAAATCAAATTGAAAAGGGCAGTTTCC

GAGAATGACCCCGAAATACTCGGCGAGCGCTTAAGCATCGCCTGCTCCACAGCACCAAAG

AAATGTAATTACAGGTTACAGCACCAACGAAAAGCGGCCCGAAGAATCTACCTACTGGTT

TTTGAGGAAGATCCGAACATATTTATCCCATTCATCCTCTCCGTTTCACCGAGAGCTTGC

GAGCACTTCAATTTCTCGATTTTCAAGTCTCAGCACGAGAAACGAACCAAAATCATGTAC

CATGAATGTGCTTTGTTCCTCATTTGGAGGGTGGCCCGTGGGCATGGCATTGAGAAGACG

AACATTTTCATCAAATTGGTACAGTGGCACATGCAAACATCCCCGCCAGTGACGGGAGCT

GAAGGCAAGGAGGACCAATACTGGTCGTTACGACTGTCTAGTTTTGCCGCAATTCAGAGT

ATGTTCGGTGATGTCATTGCAGACGCAGTTCAACGTTTCCCACCAGAAGCAGAAATGAGT

GTAGGGGATTACTTACCAGAGAGTACAACCAACATTTGGACAAGGATCCCCCACCGAGAG

CCTCAAGATTCACTAATTTGCCTCTATGTTGGACGGGCTCATGAGATCGCCAAGGTGCTA

TTTCCAATTGGAAGCCAGAAAGTTGCCTCCGTTCTCGCGGCAGATGTTCCGAATGCAACC

ATGACAAGTACTCCGCAGGCTGCTCTAGGTCCAGAGAGCGCTAATAAAACATCAACATGT

TCCGAGTTTGGTATGAAG

>CL56.Contig1_All 183 797 hypothetical protein PDIP_84870 [Penicillium digitatum Pd1] >gi|425775160|gb|EKV13442.1| hypothetical protein PDIG_38240 [Penicillium digitatum PHI26]

ATGACTGAGCCTGGCGAACCCCGTGTCCCGGAGGAATCACCAACCGCAATCCTGACCGAC

TCCCGCCCGACACCCGACACCCGCGATGACGACAGGGAAGCATTAGAACTCCATTCGATC

CAAACGCACGAAGACAACGAGCTAGACTGCTCAACCCCATCTGGCTCGTCTGGCGATGAG

CATCCCGTGTCCACACATCAGACTGCGTCGCAAGCAGGTTCCCGGCGCAGACGTGAAGCC

CACAATGGCCTTTGGGGCCAGATCTGTCGGTTCTGGACTCGACATGTTATTATAACTGTG

CCGCAGAAAAGCAACCGAGATCATTTTGCCCTGGAAAGAACGTTCCTGGCGTACGTCCGA

ACGTCCACGGTTATCGCCATGCAAGGGGTACTTGTTGCTCAACTTTTGCGTCTTCAACGG

CCATCGGAAAAAGTCGATCGCTTGTCGTTTCACGAAGTGGGTATCCCGCTTTCTGTTGCC

TGTCACTTTGTTGCAGTTTTTGTCGCCTTGACAGGTGCGTTTCGATTCTGGAGACAACAA

AACGCGATCGCTCGTGGGAAAGTATATGCTGGGGGATGGGAGTTAAACTCGGTGGGGATT

TTGTTATTCATGGTC

>CL56.Contig2_All 204 818 hypothetical protein PDIP_84870 [Penicillium digitatum Pd1] >gi|425775160|gb|EKV13442.1| hypothetical protein PDIG_38240 [Penicillium digitatum PHI26]

ATGACTGAGCCTGGCGAACCCCGTGTCCCGGAGGAATCACCAACCGCAATCCTGACCGAC

TCCCGCCCGACACCCGACACCCGCGATGACGACAGGGAAGCATTAGAACTCCATTCGATC

CAAACGCACGAAGACAACGAGCTAGACTGCTCAACCCCATCTGGCTCGTCTGGCGATGAG

CATCCCGTGTCCACACATCAGACTGCGTCGCAAGCAGGTTCCCGGCGCAGACGTGAAGCC

CACAATGGCCTTTGGGGCCAGATCTGTCGGTTCTGGACTCGACATGTTATTATAACTGTG

CCGCAGAAAAGCAACCGAGATCATTTTGCCCTGGAAAGAACGTTCCTGGCGTACGTCCGA

ACGTCCACGGTTATCGCCATGCAAGGGGTACTTGTTGCTCAACTTTTGCGTCTTCAACGG

CCATCGGAAAAAGTCGATCGCTTGTCGTTTCACGAAGTGGGTATCCCGCTTTCTGTTGCC

TGTCACTTTGTTGCAGTTTTTGTCGCCTTGACAGGTGCGTTTCGATTCTGGAGACAACAA

AACGCGATCGCTCGTGGGAAAGTATATGCTGGGGGATGGGAGTTAAACTCGGTGGGGATT

TTGTTATTCATGGTC

>CL57.Contig1_All 373 564 minus strand hypothetical protein AOR_1_2638174 [Aspergillus oryzae RIB40]

ATGCCACACCCAGTGAACGACTCCAATTCCACATTAGCCAGCAGGGGCAGCAGCAACGAT

CAGGTTGTCGCCACCGAAGTCCGAAGTCTAACAACCAATCCCGTCCGCAAGCCCCCGGTG

GTCGTTCACAACAAGGGTGGCCGACTATATGACGAGACACGACCGTCTGACTGGGACAAA

CAACGCTGGAAG

>CL57.Contig2_All 368 559 minus strand hypothetical protein AOR_1_2638174 [Aspergillus oryzae RIB40]

ATGCCACACCCAGTGAACGACTCCAATTCCACATTAGCCAGCAGGGGCAGCAGCAACGAT

CAGGTTGTCGCCACCGAAGTCCGAAGTCTAACAACCAATCCCGTCCGCAAGCCCCCGGTG

GTCGTTCACAACAAGGGTGGCCGACTATATGACGAGACACGACCGTCTGACTGGGACAAA

CAACGCTGGAAG

>CL58.Contig1_All 106 675 minus strand Calcium sensor (NCS-1), putative [Penicillium digitatum PHI26] >gi|425775609|gb|EKV13867.1| Calcium sensor (NCS-1), putative [Penicillium digitatum Pd1]

GTGTCCCGCAGTCAATCAAAGCTTTCGCCAACGCAGCTTGAGGAGCTGCAAAGGGCAACA

CATTTTGATAAGAAGGAGCTACAGCAATGGTATAAGGGATTCCTCAAGGACTGCCCGTCC

GGTACCCTGACCAAGGAAGAGTTCCAGAAAATCTACCGGCAGTTCTTCCCCTTTGGCGAC

CCATCCTCCTTTGCCAATTATGTCTTTCGCGTTTTTGATTCGGATAACAGTGGTATGATC

GATTTCAAAGAGTTTATCTGCGCCCTTTCCGTGACTTCGCGGGGCAAGATGGAGGATAAG

CTGGATTGGGCATTCCAATTATATGATATTGATGGCGATGGAAAAATCACTTATGATGAA

ATGCTGGCCATTGTCGAGGCGATCTACAAGATGGTTGGATCCATGGTGAAACTCCCCGAA

GATGAAGATACCCCGGAGAAACGTGTACGGAAGATCTTCCGGATGATGGACAAGGATGAG

AATGGCAGTCTCGACATTGAGGAATTTAAAGAAGGCAGCAAACGGGATGAAACCATCGTT

AGTGCCTTGTCGCTTTATGACGGATTGGTT

>CL58.Contig2_All 234 803 minus strand Calcium sensor (NCS-1), putative [Penicillium digitatum PHI26] >gi|425775609|gb|EKV13867.1| Calcium sensor (NCS-1), putative [Penicillium digitatum Pd1]

ATGGGAAAGTCTCAATCAAAGCTTTCGCCAACGCAGCTTGAGGAGCTGCAAAGGGCAACA

CATTTTGATAAGAAGGAGCTACAGCAATGGTATAAGGGATTCCTCAAGGACTGCCCGTCC

GGTACCCTGACCAAGGAAGAGTTCCAGAAAATCTACCGGCAGTTCTTCCCCTTTGGCGAC

CCATCCTCCTTTGCCAATTATGTCTTTCGCGTTTTTGATTCGGATAACAGTGGTATGATC

GATTTCAAAGAGTTTATCTGCGCCCTTTCCGTGACTTCGCGGGGCAAGATGGAGGATAAG

CTGGATTGGGCATTCCAATTATATGATATTGATGGCGATGGAAAAATCACTTATGATGAA

ATGCTGGCCATTGTCGAGGCGATCTACAAGATGGTTGGATCCATGGTGAAACTCCCCGAA

GATGAAGATACCCCGGAGAAACGTGTACGGAAGATCTTCCGGATGATGGACAAGGATGAG

AATGGCAGTCTCGACATTGAGGAATTTAAAGAAGGCAGCAAACGGGATGAAACCATCGTT

AGTGCCTTGTCGCTTTATGACGGATTGGTT

>CL59.Contig1_All 111 1709 minus strand Epd1 [Penicillium digitatum PHI26] >gi|425776544|gb|EKV14760.1| Epd1 [Penicillium digitatum Pd1]

ATGAAGTTCTCCTTGGTGGCACTGTTAACCCTGGCTGGGGTTACTGTTGCCGATTTGGAT

CCCATCGTCATCAAGGGCTCCAAGTTCTTCTATTCCAGCAACAACACTCAGTTCTTTATG

CGTGGAGTTGCCTATCAAAGGGAGAGTACGAGTAGCAGTGGCTACGCTGACCCTCTCGCC

GATGTCACCGCTTGTGAGCGTGACGTGCCTATCATGAAGGAGCTGCGCACCAACGTTATC

CGGACCTATGCCATCAACGCCACTGCTGACCACTCGGCTTGTATGAAGCTCTTGTCGGAC

GCTGGGATTTACGTCATCTCCGATCTATCCGATCCCAATCTATCGATCGATCGAAACGAT

CCTTCGTGGAAAACGGATCTCTTTGCCCGATACACCAGTGTTGTCGATGAACTTGCCAAG

TATAACAATACTATTGGCTTCTTCGCGGGAAATGAGGTTTCTAACACCATCGCTACTTCC

GACGCCAGCGCTTTCGTCAAGGCTGCTGTTCGCGATACCAAGAAGTACATCAAGAAGAAG

GGCTATCGCCCTATGGGGGTGGGATATGCTACCGCCGATGTGTCCGACATCCGGGCGGAC

ATGGCCGACTACTTCAACTGCGGAGACATCGACGACACGATTGACTTCTGGGGATACAAT

ATTTACTCGTGGTGCGGGGATTCCAACTACGTGGAATCTAAATACCAGGATCGCACAGAG

GAGTTTGCCAACTACTCCGTCCCTGTCTTCTTCGCCGAGTACGGTTGCAACCAGGTCCAG

CCCCGCGAATTCACCGAGGTGAAGGCCCTGTATGGCGAGACTATGGCTAGTGTCTGGTCC

GGTGGCATTGTGTACATGTACTTCCAGGAGGCCAACGACTTCGGCCTCGTTTCCGTTGTC

GATAGCACCAGTGTCCGCACAATGAGCGACTTCAGTTACTATTCCAACGAGATCGCCAGC

GCCAATCCCACTGGTGTCAACAAGGCCTCCTACACTCCCACCAACACCGCTCTGCGAAGT

TGCCCTACCGTCGGGCCCAACTGGGAAGCCGAATCCTCCCCTCTGCCCCCTATTGCGGAT

ATCGACCTGTGCGACTGCATGTACGATGCCTCCGCCTGCGCGGTCGCTGATTCTCTGGAC

TCGACGAAGTACGCCAAGCTGTTCGGCACCGTCTGCGGTAACACCGACTGCAGCGGACTG

GCAGCCAATGCTACGACCGGCGAATACGGTGCTTACAGCATGTGTTCGACCAAGCAGCAG

CTTACCTTTGCTTTGAACAAGTACTACATTGAGCAGAACCGTGCTGCTGGTGCTTGCGAC

TTTGACGGATCTGCATCCATCAAGGCCGTTACCAGTGCTACGGGCACTTGCTCTACACAG

ATGATGGAGGCCGGTATCGCTGGGACCGCAACTATCACGACGCAGAACACTGGCACTGGT

CGCTCTCGCTCTGCTTCCTCGACGGGCTCGTCGACGGGCTCGTCGACCAAGACTAGTAGC

GGCGCTATCAGCGTGCATTCCAGCTCTTCCTTCGGCTCTTTCCAGGTGGTTGCCTCCATT

GCGACCGCACTCCTGGCAGGTGTTGGAATGATCGCTCTG

>CL59.Contig2_All 3 311 Pc16g00270 [Penicillium chrysogenum Wisconsin 54-1255] >gi|211585074|emb|CAP92697.1| Pc16g00270 [Penicillium chrysogenum Wisconsin 54-1255]

ATGTACGATGCCTCCGCCTGCGTTGTGGCCGATTCCCTCTCCTCGACGAAGTACTCCAAG

CTGTTCAGCACTGTCTGCGGTTACACCGACTGCAGCGGACTGACCGCCAATGCCACCACC

GGCGAGTATGGTGCTTACAGCATGTGCTCGACCAAGCAGCAACTTGCCTTTGCTCTGAAC

AAGTACTACGTTGAGCAGAACCGTGCTGCCGATGCTTGTAGCTTCGCCGGGTCTGCAACC

GTCAAGTCGGTGACCAAGGCCACTGGCACTTGCGCTACACAGATGAAGGAGGCTGGCACT

GCTGGAACC

>CL60.Contig1_All 371 964 Rho GTPase Rho1 [Penicillium digitatum Pd1] >gi|425771412|gb|EKV09855.1| Rho GTPase Rho1 [Penicillium digitatum PHI26]

ATGGCCGAGATCCGTCGCAAGCTCGTCATCGTCGGCGATGGTGCCTGCGGTAAGACCTGT

CTGCTCATTGTTTTCTCCAAGGGCACCTTCCCCGAGGTCTACGTCCCTACAGTCTTCGAG

AACTACGTCGCCGATGTCGAGGTTGATAACAAGCACGTCGAGCTCGCTCTTTGGGATACT

GCTGGCCAGGAAGATTACGACCGTCTTCGTCCTCTTTCATACCCGGACTCACATGTGATT

TTGATCTGTTTCGCCGTGGACTCTCCCGATTCGCTTGACAACGTCCAGGAGAAGTGGATC

TCCGAGGTTCTGCACTTCTGCCAGGGTCTTCCTATTATCCTCGTCGGCTGTAAGAAGGAT

CTTCGCGATGACCGCAAGACTATTGACGAGCTTGCCAAGACCTCCCAGCGCCCCGTCTCG

CAAGACCAGGGTGAGGAAGTCCGCAAGAAGATCGGTGCCTACAAGTACCTCGAATGCTCG

GCCCGCACCAACGAGGGTGTCCGTGAAGTCTTTGAGGCTGCCACCCGTGCTGCTCTCCTG

AAGGCCGTCAAGGGCAAGGGAGGTCGCAAGCAGGGCAAGGGCTGCCTGATTCTC

>CL62.Contig1_All 1 1548 Cell-cycle checkpoint protein kinase, putative [Penicillium digitatum PHI26] >gi|425784069|gb|EKV21868.1| Cell-cycle checkpoint protein kinase, putative [Penicillium digitatum Pd1]

GTGAAGGATGAGGACGCCGAAGGGGTCTGGGGCTACCTACTACCACTGGACGAGAACGGA

AATGGTCCACTTGTACTGAGGAAGCGCGACGGTTGCGATGATGGTGGTACTGCCAAAGCC

AAGGGGAAAAAGGCTCCAAAGTCCAAGAGCGCTAAGTCGCCAGGTGGCTTCTTGGTCGGC

CGTCATCCTGAGTGTGATCGTGTCCTTGAAATTCCCACAATTTCAAACCGACACTTCCTC

ATTTTTGCCGAAAAGAAAAAAGGCGACATGGTTGCAGTACTGGAGGATCTCTCCAGCAAT

GGGACATTTGTAAATGATGCCATTGTTGGGCGTAACAAGCACCGGGAACTGGAGGACGGC

GATGAAGTCTCAATTCTGAACGAAGCTCGATTTGTCTTCCGCTACCCCCGGACTCGGGAA

ACGAATGGTTTCCGTCAGCAATACCGGATTCTCAACCAACTCGGCAAAGGCCACTTTGCA

ACCGTTTATCTGTGTGCTGAACGATCTAGTGGAGACAAATATGCAGTCAAAGTCTTTGAA

AGACGCCTCGGTGACTCGCAGAAGTCACAGAACGATACTGCTCTTCAACAAGAGATTGCC

TTGTTGATGGGCGTCCATCACCCGAATCTTCTATGTCTCAAGGAACCTTTTGATGAAAGT

GATGGCGCTTATTTGGTACTTGAATTGGCACCCGAGGGAGAACTGTTCAACTGGATTGTT

AACAATCAGAAAATGACCGAGGATGAAACTCGTGGTGTGTTCCGCCAACTGTTTGATGGA

TTGAAGTATCTGCATGAACGAGGAATTGTGCATCGAGATATCAAGCCAGAAAATATTTTG

ATTGCAGACAGAAATTTGCACGTGAAATTGGGTGACTTTGGACTGGCTAAAATTATTGGG

GAAGAGTCTTTCACAACAACACTCTGCGGTACACCAAGCTACGTTGCCCCGGAGATTTTG

CAAGATAGCCACCACCGAAAATACACCAAGGCTGTGGATGTATGGTCCCTTGGTGTTGTT

CTCTATATCTGTCTCTGTGGCTTCCCGCCATTCTCCGATGAACTGAACACTCCAGAGAAT

CCATTGACTCTCGCCCAACAGATTAAGACGGGCAGCTTTGACTACCCGTCACCGTATTGG

GACTCTGTTGGCGATCCCGCTCTGGACCTTATCGACAGGATGCTTACCGTGGACGTCAAC

AAGAGAATCACGGTGGATGAGTGTCTCGAGCACCCTTGGATGACAGGAAAGTACCCGAAT

GTCACTGAAAGCACAGATAGCCTGACGGGGGCACTTGGTAAACTGGACTTTTCTAAGCGC

AAGTTTCAACGGGAGCGGACCTTGTTGAGCAGCATAAACGATGCTCACTTCAGCGAGCAC

GCCGAAGGTAGCGACCAACCGGTCAAAGTCTTTCGAAATGAGGCCGGGAAGCGTGTCCAC

AATCGACCAGCCAAGGCGTCCCAACGTGAAGTCTCACCAAGTGGCAACCGGGCTTCAAAA

GATTTTATCAACATTGGGGAGCGTGGCGATCCCACCTTGTATGACAAT

>CL62.Contig2_All 1052 1657 minus strand Cell-cycle checkpoint protein kinase, putative [Penicillium digitatum PHI26] >gi|425784069|gb|EKV21868.1| Cell-cycle checkpoint protein kinase, putative [Penicillium digitatum Pd1]

GTTGCCCCGGAGATTTTGCAAGATAGCCACCACCGAAAATACACCAAGGCTGTGGATGTA

TGGTCCCTTGGTGTTGTTCTCTATATCTGTCTCTGTGGCTTCCCGCCATTCTCCGATGAA

CTGAACACTCCAGAGAATCCATTGACTCTCGCCCAACAGATTAAGACGGGCAGCTTTGAC

TACCCGTCACCGTATTGGGACTCTGTTGGCGATCCCGCTCTGGACCTTATCGACAGGATG

CTTACCGTGGACGTCAACAAGAGAATCACGGTGGATGAGTGTCTCGAGCACCCTTGGATG

ACAGGAAAGTACCCGAATGTCACTGAAAGCACAGATAGCCTGACGGGGGCACTTGGTAAA

CTGGACTTTTCTAAGCGCAAGTTTCAACGGGAGCGGACCTTGTTGAGCAGCATAAACGAT

GCTCACTTCAGCGAGCACGCCGAAGGTAGCGACCAACCGGTCAAAGTCTTTCGAAATGAG

GCCGGGAAGCGTGTCCACAATCGACCAGCCAAGGCGTCCCAACGTGAAGTCTCACCAAGT

GGCAACCGGGCTTCAAAAGATTTTATCAACATTGGGGAGCGTGGCGATCCCACCTTGTAT

GACAAT

>CL63.Contig1_All 52 366 minus strand Mitochondrial import inner membrane translocase subunit tim14 [Penicillium digitatum Pd1] >gi|425771099|gb|EKV09553.1| Mitochondrial import inner membrane translocase subunit tim14 [Penicillium digitatum PHI26]

ATGGCTTCTGCTCTTGCGATTGGATTTGGCATTGCCACAACTGCATTTTTGGGCCGCGCC

GGTCTGGTCGCCTACCGCCGCTCCAAGGGAGGTTTGAACGCTGCCGGAAAGGCATTCTAC

AAGGGAGGATTCGAACAGCGCATGAACCGCCGGGAAGCCTCTCTAATTCTACAACTTGCT

GAACGAACTTTGACCAAGGATAAGATCCGCAAGAACCACCGCCAGCTCATGTTGCTCAAC

CACCCCGATCGCGGCGGCAGTCCATACTTGGCCACTAAGATTAACGAGGCGAAGGAATTC

CTTGACAAACATGTT

>CL63.Contig2_All 52 366 minus strand Mitochondrial import inner membrane translocase subunit tim14 [Penicillium digitatum Pd1] >gi|425771099|gb|EKV09553.1| Mitochondrial import inner membrane translocase subunit tim14 [Penicillium digitatum PHI26]

ATGGCTTCTGCTCTTGCGATTGGATTTGGCATTGCCACAACTGCATTTTTGGGCCGCGCC

GGTCTGGTCGCCTACCGCCGCTCCAAGGGAGGTTTGAACGCTGCCGGAAAGGCATTCTAC

AAGGGAGGATTCGAACAGCGCATGAACCGCCGGGAAGCCTCTCTAATTCTACAACTTGCT

GAACGAACTTTGACCAAGGATAAGATCCGCAAGAACCACCGCCAGCTCATGTTGCTCAAC

CACCCCGATCGCGGCGGCAGTCCATACTTGGCCACTAAGATTAACGAGGCGAAGGAATTC

CTTGACAAACATGTT

>CL64.Contig1_All 208 1074 minus strand Pc20g01080 [Penicillium chrysogenum Wisconsin 54-1255] >gi|211587404|emb|CAP85437.1| Pc20g01080 [Penicillium chrysogenum Wisconsin 54-1255]

ATTTCGGATGTATCCGCGGAGCCATCCATCACCTCGCTCGATGGTATGACCGATGTGCGA

CCCCAGGCGAGTAGAGTTGCGTCTACTGCATCCCACGACAGTACTGGGAGGCACAGACGG

CGAAACCCAAGAGGAAGGCGTCCGGTGAAGGAAACGCTGGACGCTCGCTCAGAATACTAT

ACAAGCCAAGATGATGGCACTGCGGAACATCGTATCAACCAATATGTGATCAAACAGGAG

ATCGGACGTGGATCGTTTGGGGCGGTCCATCTGGCTGCAGATCAGTTTGGCAATGAATAT

GCAGTCAAGGAATTCTCCAAGTCCAGACTAAGAAAACGTGCACAGTCGCATCTGCTGCGA

AGACCGAGAGGTCCGATTCGGCCAGGAACAGATTTCAACTCGCCATTACATCGTCATCCT

TCTGGTGACGACGCTGAGACAGCAAAGAATCCTCTATATCTGATCAAGGAGGAAATCGCA

ATTATGAAGAAACTGAACCATAACAATCTAGTGTCATTGATCGAGGTCTTGGACGATCCG

ACAGAGGACTCGTTGTACATGGTGATGGAAATGTGCAAGAAGGGTGTTGTCATGAAGGTC

GGACTGGAAGAGAAAGCAGATCCGTATGACGACGAGCAATGTCGATGTTGGTTCCGTGAT

CTGATCTTGGGAATCGAGTATCTGCATTCACAGGGGATTGTCCACCGAGATATAAAACCA

GACAACTGTCTGGTGACCAATGACGACATTTTGAAAGTGGTCGATTTCGGCGTGTCGGAG

ATGTTCGAAAAAGATTCAAATATGTTCACTGGCAAGTCAGCAGGGTCACCAGCATTTCTC

CCGCCAGAGCTGTGCGTGGTCAAACAT

>CL64.Contig2_All 208 507 minus strand Pc20g01080 [Penicillium chrysogenum Wisconsin 54-1255] >gi|211587404|emb|CAP85437.1| Pc20g01080 [Penicillium chrysogenum Wisconsin 54-1255]

ATTTCGGATGTATCCGCGGAGCCATCCATCACCTCGCTCGATGGTATGACCGATGTGCGA

CCCCAGGCGAGTAGAGTTGCGTCTACTGCATCCCACGACAGTACTGGGAGGCACAGACGG

CGAAACCCAAGAGGAAGGCGTCCGGTGAAGGAAACGCTGGACGCTCGCTCAGAATACTAT

ACAAGCCAAGATGATGGCACTGCGGAACATCGTATCAACCAATATGTGATCAAACAGGAG

ATCGGACGTGGATCGTTTGGGGCGGTCCATCTGGCTGCAGATCAGTTTGGCAATGAATAT

>CL65.Contig1_All 892 1935 minus strand hypothetical protein PDIP_72720 [Penicillium digitatum Pd1] >gi|425770737|gb|EKV09201.1| hypothetical protein PDIG_63340 [Penicillium digitatum PHI26]

ATGATTATACGGAAGCTTTGTATGCGTCAGAGCCGCCAGGTCCTTGCTCTGAGCCGACGA

GCCCCGGTTTCGAGTCGCTTCCAATCGACATTGCCCCAACCCCCAGTGGAGACATTGCAG

GCATCTATCAGAGATGAGATCTCACGGGCAGAAAACCCCCTTTTTGAGATGGTGCAGGCG

CAATCTGAGGCCACCACACCCGCACAACAAGAGTTCACATCAGACGAGCCACAAAGAAGC

AATTTCAAAAAACTCGGCACAACCGTAAAATCCTCCTACGACCCCGAAAATGTCATCCGC

AACCCTCCCAAGCCCTCCCAGATCTCCCTGGAAATGTTGCTCGCAGCAGGAACCCACCTG

GGCCACTCGACTTCCCGCTGGAACCCACAGAACTCGCGGTACATCTTCGGTATCCGCGAG

GGTGTTCACATCATCTCGCTGGACATAACAGCCGCTCACCTCCGCCGCGCCGCAAAAGTA

GTTGAGGAAGTCGCCGCACGCGGCGGACTCATCCTCTTCGCCGGTACACGTAAAGGTCAG

AAGCGGGCCGTTGTCAAGGCGGCGTCGCTGGCAAAGGGATACCACATCTTCGAGCGGTGG

ATTCCAGGCTCTCTGACGAACGGCCAGCAGATTCTCGGCCACTGTGAGACGAAGGTTGTC

AACGCGCTAGACGAGGAACTGCCTCACTTCAAGTCTGATCTCGCTGATCGCCCTTCGCTC

AAGCCCGATCTGGTGATTTGTCTTAACCCGCTTGAGAACGTTGTCTTGCTGCATGAATGT

GGGCTTAACAATGTTCCTACTATCGGGATTATTGATACCGATGCGGATCCTACGCGTGTC

ACCTACCCGATTCCCTCGAACGATGATAGTCTGCGGGCTACATGTCTTATTGCGGGAATT

CTTGGCCGTGCTGGTGAGGCGGGTCAGTTGCGTCGGTTGAAGATGGCACGGGAGGGTCGG

ACCTCCTACACTCCTATCACGGCTCAAGAGCTGCGTCTGGATCCCTTTACGGGTCTGGCG

CCTGGCGGTGAGGGGCAGAAGAAT

>CL66.Contig1_All 2 640 Pc22g15230 [Penicillium chrysogenum Wisconsin 54-1255] >gi|211592458|emb|CAP98811.1| Pc22g15230 [Penicillium chrysogenum Wisconsin 54-1255]

ACGGTTTGTGATGAGAATACTTGGAAAAAACTGGTGGCAGAGGAAGTGTGTATACGTATC

GCCTGTTGGGTCTTTCTAGCGGATGGTTTCCTTACAGTCTGCTTCAAAAACCACCCATCA

ATATCGGTCTTTGAGATGGACTGTCATTTTCCTTGGAGTGCCGGGTTATGGGAAGCCGAA

AGCGCGTCTTCCTTTAGCAGAATTGCCATGTCGCACTCGACAGAGCTCCCGCTTCCTCCC

TTCAAGGATGTCATCACGCAGTTGCTCGAAACCACTGCAAATAATGGCCCAATAACATGG

GGTCTTTCGGTATCCGTGGAACATCTCTTGATCCTGATTTATGCTATTAACTCTCTCGCA

TTCCAAGCGAGAGCGGGCTTATTAAGATACCTATCGCTCGATAAAATACGCTGTGCGTCT

GATAATTGGAAGCGCATTTGGGATTCTGTCATCGGTCTCTTGAATAAGGATCAATTCCTT

CACCTCGGGTATCCAAAACACGCTCAAGAACTTTGGTGGTTGTTGAATGCCACGCTAGAT

GCTTCCAGCAGAGATGATGTCAGCCTTAGTTATATGGACAACACTGCTACTGATGATTTG

GGGAATTTGAATGAATTTATTCAATGGTGTCACCAAAGC

>CL66.Contig2_All 32 553 Pc22g15230 [Penicillium chrysogenum Wisconsin 54-1255] >gi|211592458|emb|CAP98811.1| Pc22g15230 [Penicillium chrysogenum Wisconsin 54-1255]

ATCGCCTGTTGGGTCTTTCTAGCGGATGGTTTCCTTACAGTCTGCTTCAAAAACCACCCA

TCAATATCGGTCTTTGAGATGGACTGTCATTTTCCTTGGAGTGCCGGGTTATGGGAAGCC

GAAAGCGCGTCTTCCTTTAGCAGAATTGCCATGTCGCACTCGACAGAGCTCCCGCTTCCT

CCCTTCAAGGATGTCATCACGCAGTTGCTCGAAACCACTGCAAATAATGGCCCAATAACA

TGGGGTCTTTCGGTATCCGTGGAACATCTCTTGATCCTGATTTATGCTATTAACTCTCTC

GCATTCCAAGCGAGAGCGGGCTTATTAAGATACCTATCGCTCGATAAAATACGCTGTGCG

TCTGATAATTGGAAGCGCATTTGGGATTCTGTCATCGGTCTCTTGAATAAGGATCAATTC

CTTCACCTCGGGTATCCAAAACACGCTCAAGAACTTTGGTGGTTGTTGAATGCCACGCTA

GATGCTTCCAGCAGAGATGATGTCAGCCTTAGTTATATGGAC

>CL67.Contig1_All 395 751 RecName: Full=Protein pxr1; AltName: Full=PinX1-related protein 1

AAGACCACCGAGGACGACGAAACCTCTTCAAGTGAATCGGATGCCCCAATCCGCAAATCA

AAGTCCAAGTCCAAGTCCAAATCTAAATCCAAGAAATCGCGCTCTCGAGATGAAAACGAT

GGCAACGAATCAAGCTCGGAACCGAAGAAGAAAAAGAAGTCCAAGAAGAGAAAGGCTGAT

ACGGAGGAGAGTGATTCTTCAGACAAGTCATCTGAACCGGAAGTCAAGGTAGCAGCAACG

ATATCCAGGGAGCGGCGGCCCATGGGACGGAATGTCACTCGGTCACGCCATATCGCCCAG

AAGAAGAGAGCTATCATGGATGACAAGTCTCTCAACGAGATCTTCATGATCAAAGCA

>CL67.Contig2_All 28 879 G-patch RNA maturation protein (Gno1), putative [Penicillium digitatum PHI26] >gi|425784063|gb|EKV21862.1| G-patch RNA maturation protein (Gno1), putative [Penicillium digitatum Pd1]

ATGGGTTTAGCAGGGCCGCGGAAAAGTACCAAGATTGGGAATGATCCTAATAATACCAAA

TGGACCCGGTCCACGACCGGGTTCGGACATCGAATCATGAGCTCCCAGGGTTGGATTCCT

GGAAGTCTTCTGGGAGCGAAGGATGCCGCGCACGCCAACTTGTTGACTGCCGCCAGCGCT

TCCCACATCAAAGTCACCCTGAAGGACGATAATCTCGGATTGGGTGCTCGTATTGGACGA

GAGAATGAGCCTACTGGACTTGATGCCTTCAAAGGATTGCTTGGTCGACTCAATGGCAAG

AGCGAAGTAGAATTGAAGAAGGATGAACAAAAGCGCGATGATGTTCGATTGGCTCGATAT

GCTGCCCTGAAATTCCCGGAAGTCAGGTTCGTCAGCGGTGGACTTTTGGCACAGGAAAAA

GAAGCCGAGATCCCTCCGCCGACCCCCAAGGATACAAAAACCAAGAAATCAAAATTGGAC

AAGAAAGAACACACCAAGACCACCGAGGACGACGAAACCTCTTCAAGTGAATCGGATGCC

CCAATCCGCAAATCAAAGTCCAAGTCCAAGTCCAAATCTAAATCCAAGAAATCGCGCTCT

CGAGATGAAAACGATGGCAACGAATCAAGCTCGGAACCGAAGAAGAAAAAGAAGTCCAAG

AAGAGAAAGGCTGATACGGAGGAGAGTGATTCTTCAGACAAGTCATCTGAACCGGAAGTC

AAGGTAGCAGCAACGATATCCAGGGAGCGGCGGCCCATGGGACGGAATGTCACTCGGTCA

CGCCATATCGCCCAGAAGAAGAGAGCTATCATGGATGACAAGTCTCTCAACGAGATCTTC

ATGATCAAAGCA

>CL68.Contig4_All 99 563 minus strand hypothetical protein PDIG_07390 [Penicillium digitatum PHI26] >gi|425782995|gb|EKV20870.1| hypothetical protein PDIP_12090 [Penicillium digitatum Pd1]

ATGGCCTCTACTGCATACGACGCGTTCATTCACAAGATATTCGCTAAAGTCACTCAGCTC

CAATCGAGGGGTCTGTATACGGAGGATCGGAATACCATCAAAGGAAATCGCCTCAATCTC

ATTTGGCTTGAACCTACTGGTGACTCCGTTCCAAAACAGACAAGATGGCGACAAAACCGT

GCGCGTGACAAGTATAGGGAGATCCAGGAAGCCAGCAGTCATTTATTTCTTGCGGTGTTT

TTAACTATTCCACCCAGTATCTGCTTCAGTTCTGAGTTCCAGTCAGTGATCAATTACCTG

GTTGGCCTTGATAATTACGAAGATTTCAGATTCTCGCTGAGCCTCAAAGAAAAGGAGCTT

TTTGAATCGGCAGCAGCGGAACAAGGCTACGCTGGAAGCACACTGTATCTCAGGTTTATG

CAAGTCATGTTTCCTGAAGTAGAACGCAGACAAATTCAATATGCA

>CL68.Contig5_All 99 563 minus strand hypothetical protein PDIG_07390 [Penicillium digitatum PHI26] >gi|425782995|gb|EKV20870.1| hypothetical protein PDIP_12090 [Penicillium digitatum Pd1]

ATGGCCTCTACTGCATACGACGCGTTCATTCACAAGATATTCGCTAAAGTCACTCAGCTC

CAATCGAGGGGTCTGTATACGGAGGATCGGAATACCATCAAAGGAAATCGCCTCAATCTC

ATTTGGCTTGAACCTACTGGTGACTCCGTTCCAAAACAGACAAGATGGCGACAAAACCGT

GCGCGTGACAAGTATAGGGAGATCCAGGAAGCCAGCAGTCATTTATTTCTTGCGGTGTTT

TTAACTATTCCACCCAGTATCTGCTTCAGTTCTGAGTTCCAGTCAGTGATCAATTACCTG

GTTGGCCTTGATAATTACGAAGATTTCAGATTCTCGCTGAGCCTCAAAGAAAAGGAGCTT

TTTGAATCGGCAGCAGCGGAACAAGGCTACGCTGGAAGCACACTGTATCTCAGGTTTATG

CAAGTCATGTTTCCTGAAGTAGAACGCAGACAAATTCAATATGCA

>CL68.Contig9_All 99 563 minus strand hypothetical protein PDIG_07390 [Penicillium digitatum PHI26] >gi|425782995|gb|EKV20870.1| hypothetical protein PDIP_12090 [Penicillium digitatum Pd1]

ATGGCCTCTACTGCATACGACGCGTTCATTCACAAGATATTCGCTAAAGTCACTCAGCTC

CAATCGAGGGGTCTGTATACGGAGGATCGGAATACCATCAAAGGAAATCGCCTCAATCTC

ATTTGGCTTGAACCTACTGGTGACTCCGTTCCAAAACAGACAAGATGGCGACAAAACCGT

GCGCGTGACAAGTATAGGGAGATCCAGGAAGCCAGCAGTCATTTATTTCTTGCGGTGTTT

TTAACTATTCCACCCAGTATCTGCTTCAGTTCTGAGTTCCAGTCAGTGATCAATTACCTG

GTTGGCCTTGATAATTACGAAGATTTCAGATTCTCGCTGAGCCTCAAAGAAAAGGAGCTT

TTTGAATCGGCAGCAGCGGAACAAGGCTACGCTGGAAGCACACTGTATCTCAGGTTTATG

CAAGTCATGTTTCCTGAAGTAGAACGCAGACAAATTCAATATGCA

>CL68.Contig10_All 99 563 minus strand hypothetical protein PDIG_07390 [Penicillium digitatum PHI26] >gi|425782995|gb|EKV20870.1| hypothetical protein PDIP_12090 [Penicillium digitatum Pd1]

ATGGCCTCTACTGCATACGACGCGTTCATTCACAAGATATTCGCTAAAGTCACTCAGCTC

CAATCGAGGGGTCTGTATACGGAGGATCGGAATACCATCAAAGGAAATCGCCTCAATCTC

ATTTGGCTTGAACCTACTGGTGACTCCGTTCCAAAACAGACAAGATGGCGACAAAACCGT

GCGCGTGACAAGTATAGGGAGATCCAGGAAGCCAGCAGTCATTTATTTCTTGCGGTGTTT

TTAACTATTCCACCCAGTATCTGCTTCAGTTCTGAGTTCCAGTCAGTGATCAATTACCTG

GTTGGCCTTGATAATTACGAAGATTTCAGATTCTCGCTGAGCCTCAAAGAAAAGGAGCTT

TTTGAATCGGCAGCAGCGGAACAAGGCTACGCTGGAAGCACACTGTATCTCAGGTTTATG

CAAGTCATGTTTCCTGAAGTAGAACGCAGACAAATTCAATATGCA

>CL68.Contig11_All 99 563 minus strand hypothetical protein PDIG_07390 [Penicillium digitatum PHI26] >gi|425782995|gb|EKV20870.1| hypothetical protein PDIP_12090 [Penicillium digitatum Pd1]

ATGGCCTCTACTGCATACGACGCGTTCATTCACAAGATATTCGCTAAAGTCACTCAGCTC

CAATCGAGGGGTCTGTATACGGAGGATCGGAATACCATCAAAGGAAATCGCCTCAATCTC

ATTTGGCTTGAACCTACTGGTGACTCCGTTCCAAAACAGACAAGATGGCGACAAAACCGT

GCGCGTGACAAGTATAGGGAGATCCAGGAAGCCAGCAGTCATTTATTTCTTGCGGTGTTT

TTAACTATTCCACCCAGTATCTGCTTCAGTTCTGAGTTCCAGTCAGTGATCAATTACCTG

GTTGGCCTTGATAATTACGAAGATTTCAGATTCTCGCTGAGCCTCAAAGAAAAGGAGCTT

TTTGAATCGGCAGCAGCGGAACAAGGCTACGCTGGAAGCACACTGTATCTCAGGTTTATG

CAAGTCATGTTTCCTGAAGTAGAACGCAGACAAATTCAATATGCA

>CL68.Contig12_All 99 563 minus strand hypothetical protein PDIG_07390 [Penicillium digitatum PHI26] >gi|425782995|gb|EKV20870.1| hypothetical protein PDIP_12090 [Penicillium digitatum Pd1]

ATGGCCTCTACTGCATACGACGCGTTCATTCACAAGATATTCGCTAAAGTCACTCAGCTC

CAATCGAGGGGTCTGTATACGGAGGATCGGAATACCATCAAAGGAAATCGCCTCAATCTC

ATTTGGCTTGAACCTACTGGTGACTCCGTTCCAAAACAGACAAGATGGCGACAAAACCGT

GCGCGTGACAAGTATAGGGAGATCCAGGAAGCCAGCAGTCATTTATTTCTTGCGGTGTTT

TTAACTATTCCACCCAGTATCTGCTTCAGTTCTGAGTTCCAGTCAGTGATCAATTACCTG

GTTGGCCTTGATAATTACGAAGATTTCAGATTCTCGCTGAGCCTCAAAGAAAAGGAGCTT

TTTGAATCGGCAGCAGCGGAACAAGGCTACGCTGGAAGCACACTGTATCTCAGGTTTATG

CAAGTCATGTTTCCTGAAGTAGAACGCAGACAAATTCAATATGCA

>CL69.Contig1_All 2 952 minus strand hypothetical protein PDIG_76310 [Penicillium digitatum PHI26] >gi|425775562|gb|EKV13822.1| hypothetical protein PDIP_46770 [Penicillium digitatum Pd1]

TTGTTTGTTGACGAGCTTCAGTGCTCCTACCCAGGATCTAAAGTCAGAGTAATCGGATGT

GACATCTCCAATAAGGATAGCTTTGCTCTTGGATTGGAAAAAATTGCCGCGGAACTTCCG

CCAATCCGGGGTGTTGTCCAGGCTGCAATGGTGCTCGACGATTCCATTCTCGAGAACATG

ACAATCAACAACTATAATGCAGCTATCCAGCCCAAGGTACAGGGAACATGGAACCTCCAT

CAGCAGCTCGGTTCTGATCTGGACTTTTTCATTATGCTCTCCTCTCTCGCTGGTGTAATC

GGAAACGCCAGTCAGAGCAACTACACAGCTGGCGGAGCCTTCCAGGACGCGCTCGCCAGG

CATCGAGTGGCCAAAGGTCTTCCAGGAGTGGCTCTCGATATTGGCGCTGTCAAAGATATC

GGATATGTGGCATCAAACAAGGGCGTGTATGAGCGTCTGAAGAAAATGGGCTACCGATTG

CTTGCAGAGGAGGAAATCATGTCCGCCATAGAATCAGCCATCCTTGATCCTTGCCCACAA

GTCATGGTCGGAATTAACACTGGCGGTGGTTCTTCTGACTCGATTCTGGCTCGAGACTCA

CGGTTCAGTGCCCTGCGCTTTACTAAACCAGCCAACGGCAGCAATAGTGCATCCAAGGCT

AGCAGCGTAGCCGGTAGTCTCGCTGGTAAGCTGTCTTCTGCCGAATCCCTACATGAGGCA

GCTGGCTTGGTTATGGAAGCTTTGGTCCAGAAGTTGGTGGACATTTTCATGATTCCTGCG

GAGGAGGTCATACCTGCCAAGTCGATGGCAGCTTTTGGCGTCGATTCCCTTGTTGCTGTC

GAGCTGCGGAACATGCTAGCTTTGAAGGCGGGATCAGAGGTTTCGATTTTTGACATAATG

CAGAGTCCGTCTCTCGCAGTCCTCTGCGACAAGGTCGCGTCAACCAGTGGT

>CL69.Contig2_All 2 964 minus strand hypothetical protein PDIG_76310 [Penicillium digitatum PHI26] >gi|425775562|gb|EKV13822.1| hypothetical protein PDIP_46770 [Penicillium digitatum Pd1]

TTGTTTGTTGACGAGCTTCAGTGCTCCTACCCAGGATCTAAAGTCAGAGTAATCGGATGT

GACATCTCCAATAAGGATAGCTTTGCTCTTGGATTGGAAAAAATTGCCGCGGAACTTCCG

CCAATCCGGGGTGTTGTCCAGGCTGCAATGGTGCTCGACGATTCCATTCTCGAGAACATG

ACAATCAACAACTATAATGCAGCTATCCAGCCCAAGGTACAGGGAACATGGAACCTCCAT

CAGCAGCTCGGTTCTGATCTGGACTTTTTCATTATGCTCTCCTCTCTCGCTGGTGTAATC

GGAAACGCCAGTCAGAGCAACTACACAGCTGGCGGAGCCTTCCAGGACGCGCTCGCCAGG

CATCGAGTGGCCAAAGGTCTTCCAGGAGTGGCTCTCGATATTGGCGCTGTCAAAGATATC

GGATATGTGGCATCAAACAAGGGCGTGTATGAGCGTCTGAAGAAAATGGGCTACCGATTG

CTTGCAGAGGAGGAAATCATGTCCGCCATAGAATCAGCCATCCTTGATCCTTGCCCACAA

GTCATGGTCGGAATTAACACTGGCGGTGGTTCTTCTGACTCGATTCTGGCTCGAGACTCA

CGGTTCAGTGCCCTGCGCTTTACTAAACCAGCCAACGGCAGCAATAGTGCATCCAAGGCT

AGCAGCGTAGCCGGTAGTCTCGCTGGTAAGCTGTCTTCTGCCGAATCCCTACATGAGGCA

GCTGGCTTGGTTATGGAAGCTTTGGTCCAGAAGTTGGTGGACATTTTCATGATTCCTGCG

GAGGAGGTCATACCTGCCAAGTCGATGGCAGCTTTTGGCGTCGATTCCCTTGTTGCTGTC

GAGCTGCGGAACATGCTAGCTTTGAAGGCGGGATCAGAGGTTTCGATTTTTGACATAATG

CAGAGTCCGTCTCTCGCAGTCCTCTGCGACAAGGTCGCGTCAACCAGTGGCTTTGTTGTT

GTT

>CL71.Contig1_All 77 1351 minus strand Pc22g00210 [Penicillium chrysogenum Wisconsin 54-1255] >gi|211591084|emb|CAP97309.1| Pc22g00210 [Penicillium chrysogenum Wisconsin 54-1255]

ATGGCCTCTGAAACCATCACCCGGAACGTTGCCACTCAGTCCTGGTTTGATCAAGCTTGT

TTCATCCTACCCGATGCAATGTTTGCATTAACTGCTCAGTACATCACAGATTCATCTCCC

ACGAAAGTCAACTTGGGCCAAGGTACGTACCGCGACGAGCAGGGGAACCCGTGGGTTCTT

CCCTCGGTCAGGAAGAGTCGGAAGTTGCCATCACAGGAGTTGAACCACGAATATCTCCTT

ATTGTTGGTCTGGCAGAATTCTGCAAAGAGGCTGCAAAGTTAGCCCTTGGCCCAGAGCTC

TTTCAAAAGAAACATGAAAAAGCATGAAAAAGCATGAAAAAGTACTACAACCCAGAGACC

AAAAACTTGGACATTGATTGTTACTGTTCGGCATTGAAGTTGGCCGAGCCCCAGTCTGTG

TTTATCATCCATGCATGTGCTCACAACCCAACAGGGGGTGATCCGAGTAAGGAGCAATGG

CAAGAACTTGCGCGTCTCTTTAAAGCGAGGCAGTTGTTCCCACTTTTTGACGCCGCTTTT

CTAGGGTTCAACTCCGGCAATGTTGATAGTGATGCCTTTGCCGTTCGACTATTAATCGAG

AAAATGAGGTTGGAAGCTGGAGTGTGTCTGTCTTTTGCGAAAAACATGGGTCTTTGTGGT

ATCTACCCGTGCCAGGGTCAGTCCGTTGTCGAAACGATGGCCAATAACATTCGACAGGGG

GAACGAGTCGGCTGCTTTTTTCTGGCAACGAGCACCGAAAAAGCGGCGATGAAGACCCAA

TAAATGCTTGAGATGCTTCAGCGTTCCGAAGTCTCGAACCCTCCAGCCTATGGTGCGAAA

ATCGCGAGCACCACATTAGCCGATGCTACTCTTTGGAAGGCTTGGCATGATGATCTGATC

GCAATGAGCAGCCGCATTCGCTCTATGAGATCTGAATTATCTGATAGTCTGGTGTCCTCT

GGTAGGCGTCGCCTGTCCGGAACAGACGACCTAAGATCTTATTAACATGATCAGGCGTGC

CTGGCTCTTGGGAGCACCTAATCCGCCAGTCCGGAATGTTTGGATTTCTGGGACTATCCC

CGACCGTCGTGCGAATGCGTCGTGGTTTGTTCTGTCATTGCAACAACTGTTTTTGTGATA

TACTGACATGTGAGGACCTCAGAAAATTTCCACATCTACATGGCCAAATTTTCCCGAATC

TCAATTGCTGGCTCAACAAACAAGAATGTGGGCTATGTTGGAAAATCAGTCACTGACTGC

TTGAAGCAAGAGTGC

>CL71.Contig2_All 99 1373 minus strand Pc22g00210 [Penicillium chrysogenum Wisconsin 54-1255] >gi|211591084|emb|CAP97309.1| Pc22g00210 [Penicillium chrysogenum Wisconsin 54-1255]

ATGGCCTCTGAAACCATCACCCGGAACGTTGCCACTCAGTCCTGGTTTGATCAAGCTTGT

TTCATCCTACCCGATGCAATGTTTGCATTAACTGCTCAGTACATCACAGATTCATCTCCC

ACGAAAGTCAACTTGGGCCAAGGTACGTACCGCGACGAGCAGGGGAACCCGTGGGTTCTT

CCCTCGGTCAGGAAGAGTCGGAAGTTGCCATCACAGGAGTTGAACCACGAATATCTCCTT

ATTGTTGGTCTGGCAGAATTCTGCAAAGAGGCTGCAAAGTTAGCCCTTGGCCCAGAGCTC

TTTCAAAAGAAACATGAAAAAGCATGAAAAAGCATGAAAAAGTACTACAACCCAGAGACC

AAAAACTTGGACATTGATTGTTACTGTTCGGCATTGAAGTTGGCCGAGCCCCAGTCTGTG

TTTATCATCCATGCATGTGCTCACAACCCAACAGGGGGTGATCCGAGTAAGGAGCAATGG

CAAGAACTTGCGCGTCTCTTTAAAGCGAGGCAGTTGTTCCCACTTTTTGACGCCGCTTTT

CTAGGGTTCAACTCCGGCAATGTTGATAGTGATGCCTTTGCCGTTCGACTATTAATCGAG

AAAATGAGGTTGGAAGCTGGAGTGTGTCTGTCTTTTGCGAAAAACATGGGTCTTTGTGGT

ATCTACCCGTGCCAGGGTCAGTCCGTTGTCGAAACGATGGCCAATAACATTCGACAGGGG

GAACGAGTCGGCTGCTTTTTTCTGGCAACGAGCACCGAAAAAGCGGCGATGAAGACCCAA

TAAATGCTTGAGATGCTTCAGCGTTCCGAAGTCTCGAACCCTCCAGCCTATGGTGCGAAA

ATCGCGAGCACCACATTAGCCGATGCTACTCTTTGGAAGGCTTGGCATGATGATCTGATC

GCAATGAGCAGCCGCATTCGCTCTATGAGATCTGAATTATCTGATAGTCTGGTGTCCTCT

GGTAGGCGTCGCCTGTCCGGAACAGACGACCTAAGATCTTATTAACATGATCAGGCGTGC

CTGGCTCTTGGGAGCACCTAATCCGCCAGTCCGGAATGTTTGGATTTCTGGGACTATCCC

CGACCGTCGTGCGAATGCGTCGTGGTTTGTTCTGTCATTGCAACAACTGTTTTTGTGATA

TACTGACATGTGAGGACCTCAGAAAATTTCCACATCTACATGGCCAAATTTTCCCGAATC

TCAATTGCTGGCTCAACAAACAAGAATGTGGGCTATGTTGGAAAATCAGTCACTGACTGC

TTGAAGCAAGAGTGC

>CL72.Contig1_All 367 615 Negative regulator of DNA transposition, putative [Penicillium digitatum Pd1] >gi|425771410|gb|EKV09853.1| Negative regulator of DNA transposition, putative [Penicillium digitatum PHI26]

GCTGAGGCAGCCGGTCAAGATGAAAGCGAGCTCCAGAAAGCCCAGAGGGAGCTTGAAGAC

CAAGAGGATGAAGAGGAGGAAGATTACAACCCTGACAGTGACGATAGTGAAGGCAGCGGG

TCCAGCAGTGAAGAAGATGATGAAGATGATGAAGACGATGAAGACGATGAAGATGCTGGC

CAGGACAGTGATGAAGACATAGTCGAGAATGAGCTTCGGAGTGAAGCTGAGGACATTGCT

GAAGATCAT

>CL72.Contig2_All 95 667 Negative regulator of DNA transposition, putative [Penicillium digitatum Pd1] >gi|425771410|gb|EKV09853.1| Negative regulator of DNA transposition, putative [Penicillium digitatum PHI26]

GGCTACCTCTTCCTTCTTTCTACAGGTATCTTGTTTGCATTTAAGAAACCGCTTCTGTTC

TTTTCCTTTGCTACCGTTGACTCTGTCTCTTACACATCCGTCCTCCAACGGACATTCAAT

CTCAATGTCATGGCTCGCCCGTCGAACGGCTCCGAGAAAGACATCCAAGAATTCGAATTT

TCGATGATTGATCAGGATAATTTCTCCGGGATCGACACCTACATCAAACGACATGGCCTC

CAGGATGCTAGTCTCGCAGAGGCGCGGCGCGCTAAAGTTTACAATGTCAATAAGGCCAGC

GGAGAGGACGCAACGGAACCCACCGCTGAGGCAGCCGGTCAAGATGAAAGCGAGCTCCAG

AAAGCCCAGAGGGAGCTTGAAGACCAAGAGGATGAAGAGGAGGAAGATTACAACCCTGAC

AGTGACGATAGTGAAGGCAGCGGGTCCAGCAGTGAAGAAGATGATGAAGATGATGAAGAC

GATGAAGACGATGAAGATGCTGGCCAGGACAGTGATGAAGACATAGTCGAGAATGAGCTT

CGGAGTGAAGCTGAGGACATTGCTGAAGATCAT

>CL73.Contig1_All 52 615 minus strand Beta-N-acetylglucosaminidase, putative [Penicillium digitatum Pd1] >gi|425770160|gb|EKV08633.1| Beta-N-acetylglucosaminidase, putative [Penicillium digitatum PHI26]

ATGGCTTCTTCGGATCTTCAAAAGCAGGTTGGCCAGCTCTTCACGGTGGGGTTCTATGGC

TGCACCCTAAGTCCCGAAATCAAGACTTTAATCCATGACTACCATGTTGGAGGAATTGTC

CTCTTCTCACGCAATTTGAAGAATGCAGAACAGCTTCAGGCCTTGACACTGGCTTTACAG

AATGAAGCTAAATCGGCCGGGCATGAGCGACCACTCTTGATCGGAATTGACCAGGAGAAT

GGATTGGTCACTAGAATTTCTCCCCCGATCGCTACTCGAGTACCGGGCCCTATGGCACTC

GGCGCCACGCATGATCCAGAGTGTGCCTATAGTGCGGGGAAAGCCACGGGGGAAACTCTG

GGCTTCTTCGGGATCAACATGAACTATGCACCCGTGTGCGATGTCAACTCAGAGCCGTTA

AACCCGGTTATTGGTGTACGTAGCCCTGGTGATGACCCAGAATTTGTGGGCCGGTTTGCG

AGTGCAGCCGCGCGGGGCCTGCGCGAACAAAATGTTGTCCCCAGTGTGAAGCATTTTCCA

GGTCATGGAGACACAGCTGTCGAC

>CL73.Contig2_All 108 272 Pc18g01310; K01207 beta-N-acetylhexosaminidase [EC:3.2.1.52]

CAGCTCTTCACGGTGGGGTTCTATGGCTGCACCCTAAGTCCCGAAATCAAGACTTTAATC

CATGACTACCATGTTGGAGGAATTGTCCTCTTCTCACGCAATTTGAAGAATGCAGAACAG

CTTCAGGCCTTGACACTGGCTTTACAGAATGAAGCTAAATCGGCC

>CL74.Contig1_All 67 1314 Aspartyl proteinase [Penicillium digitatum PHI26] >gi|425782900|gb|EKV20780.1| Aspartyl proteinase [Penicillium digitatum Pd1]

ATGAAAGCACCTGCTCATCTCTCACTGGGGATCCTGTTCCTGCTTCCTGCAAGCACTGTG

CTTGGTAGCAAAACGAAGAAGCATGCGGAAGCGACAACTACAAAAGTGTCAGTCCCCATG

CAATCAAGTCCCCTAGGCCCTCTATTCCATATTACACTTGGCACACCGCCACAGTCCTTG

ACTGTCCTAAGTGACTGGACGTGGATGTCACTTTTTGCACGTTCCGCAAACTGCGTGGGA

TCATACGATCTTGCAAAGTGCATCCCATCAGGCCAAGAATACTTCAATGAGAAAGATTCC

AGAACATTCCTTTCGACCAATCTCTCCGCAACCGGTTGGGACGGGACTAGCTTCCTACCC

GGATACGAATTTGCCGTGACCTACAATCGGGACAAAGTCTGTATCGGGGACGTCTGTGAT

GACGGAGTCAGTTTCCAACTTTCTGATTTCGATCTTGTCATTGATGAGGTGGTTCCATTT

GGTGGCATCTTTGGACTCTCTCCTGTGTTTCCTTCCGAAAATGCTACATTCTATCCAGCT

TCATACCAGGCTTACCTTGGAGGTCAGCTAGGCCCGCAAGTGGGATTCCATTCTTGCGCG

GCATTGACTTCCAAATCCACATGCGGAGGAGGTGATATGCAGACTGTGTTTGGTGGCACG

GCGGATAGGGAAGTTTTTGATCCCGCCGAAGTTGTTCGTTTCGAAGTGAACGTAGGAAAC

TGCCTCTTTGAAAGTTCTCCACTGAATGTCACTCCTTCTCGACACAATTTCTGGGCGATT

GAGTGGACAGGAATGTGGATTGGATCGGATAAAGTCGAACTCAACCAGGAGACGTTACTC

ACCGGATGCGCTGATGGGAAACCCAGAGCTGTGTTTGATGAAGGCTCGGAAGGAAATGGT

GCTCCGATTCCACTGAATGCACTTCCTGGCCTCCTGCATACGACAAATGCGACGGAGTTT

GGTACAGGCCCAGAGGCATCATATCGACTGTCGACAGTTGAAAATCTTGACAGGTTTCCG

ACTCTCACTTATGAGTTCCAAGGGAAGCAGAATTACACTGTCTCACCCAGGTTTTATGTG

AGTTGCACCAATGAAAGCTGCACACTCAACATCAAGACGTGGGACTTTTACTCGAATGGA

CCCATGGCATTTTTTGGACAAACTTTCTTCGCGGCTCTTTACGTGATTCTTGACTTTGAA

AGCCTTCAAGTGGGACTGGCACCTTTGAAACAGAGCTTGATAGCTTCG

>CL74.Contig2_All 180 677 hypothetical protein PDIG_01680 [Penicillium digitatum PHI26] >gi|425782898|gb|EKV20778.1| hypothetical protein PDIP_13010 [Penicillium digitatum Pd1]

ATGGCTTCGGGGCTTTTCTTCGACCCGCTCAAGCTCCTCCGCGTTGCCCCGCTTCTTACC

ACCACATCGGCACTGCTCTATGCTTGGGATGAGCATTGGTATCTGTCAGGCTTCCTGCGC

TCAGAGCATAAGGCCGAAACGGAGACGGTCCTCCCGAGTTACTTCCGTCGCTTCTTTGAA

CAGGGAATCTTTATTGTCGCCGGTCTCAACACCCTCACGGTCTCCACTTCGGTCGCCAAT

CTGCTGACTGATCGCCCGGTACTGGACCGTCTCCGATCTTCGCAATGGTACTGGGCTGGT

CTGGGCTTCACTGCTTGTCACTTTTTGTTCGTGCCTCTTATCGCGTATCCAATTCGCGAC

ATTATAGAGAATCGGTCTCAGGGGAAAAGCACTAAGGATTTGCAGCGGTGGATCGATATT

CATCGTATTCGGGTTTTTGTTGCTGATTTGCCGGCGTGGATGAGTTTCCTGGTCGCCGTG

CTTTCTACAATTCACCTA

>CL75.Contig1_All 543 1676 NOT2 family protein [Penicillium digitatum PHI26] >gi|425783144|gb|EKV21004.1| NOT2 family protein [Penicillium digitatum Pd1]

ATGTGGGCCAACGCAAGTCAACGCGCAGCTCAGCAAACACCTGTCCAAAGGCAACAGCTC

CCTCAGCCATCGCAAACCCCCTCCCGTTCCTCCCAGACCCAAGGTCTTCCTACACCTCAA

CAATCTCAACCATCGCATGATGACATGTTTCCTTCTGGTTCGCAGTTTGCAAATCGACTC

GATGACTTTAGAAATGGTGGCCAGGGTATCAGTAGCCAACTCAGTGCGGGTGGCCAGCCC

CAGCCAAGCAATATTGAAGAGTTTCCCCCTTTGGGTCGTAATGCTGCTGCTGAGCTGCCT

CTCGGTCGAACTGGTTCATTGATGCAAGGTGCGGGTTTTGGCGGCTATGGAACTTCTATG

GGGGCACCGCGCTCGCCAGTCAGCCAAGTGCCCAATGGCATTTTGGGACAAGAGAAAGAA

GAATTGCATAACGGTGTGCTTCCAGGCCAGCGTAATTTTAGTGACCCACAACAGCTACAA

CAAAGGCAACCAGAAAATAACGACGGACAAGATGTTTCTATCGCGCCTCAGAGCACTGAA

CAACCGCCCCTCGCACAGATGAGTGAGCTTGACAGGTTTGGGTTGGCGGGTCTGCTGCGT

ATGATTCACAGCGAGAGTCCTGATGTTGCAAGTCTTGCTGTTGGCCAAGATTTGATGACA

CTAGGATTGGATTTAAATCAGGCGGAGCCTTTACACACATCGTTTGCTTCACCATTCGTT

TCATCGATGTCAGCAGTCCCTTTGGAACAAGACTTCTCTTTGCCGGGATGTTATAATGTC

GCCAATATCCAACCCCTCCAGTCTCGCATTCCCGGTTTCAGCGACGAGACCCTGTTTTTC

ATCTTTTACAGTATGCCTCGGGATATCATGCAGGAACTGGTGGCCGAGGAATTGATGGGC

CGCAAATGGCGGTACCACAAGCTTGAACGATGCTGGCTAACTCGCGATGAGACATATCCT

GGACCCGTTGACGTGGAACGTGGTGTGACGGAACGCGGCGTGTATCTTCTATGGGATTCA

TCTGCCTGGAAGAAAGTCCGACGTGAATTCATCCTTCGATATGAAGACTTGGATAACCGG

TTGGATCCTAATCGAGGCGCCGCTCGCCCACCTGGCGTGGTGCATCATGCCTCA

>CL75.Contig2_All 651 1784 NOT2 family protein [Penicillium digitatum PHI26] >gi|425783144|gb|EKV21004.1| NOT2 family protein [Penicillium digitatum Pd1]

ATGTGGGCCAACGCAAGTCAACGCGCAGCTCAGCAAACACCTGTCCAAAGGCAACAGCTC

CCTCAGCCATCGCAAACCCCCTCCCGTTCCTCCCAGACCCAAGGTCTTCCTACACCTCAA

CAATCTCAACCATCGCATGATGACATGTTTCCTTCTGGTTCGCAGTTTGCAAATCGACTC

GATGACTTTAGAAATGGTGGCCAGGGTATCAGTAGCCAACTCAGTGCGGGTGGCCAGCCC

CAGCCAAGCAATATTGAAGAGTTTCCCCCTTTGGGTCGTAATGCTGCTGCTGAGCTGCCT

CTCGGTCGAACTGGTTCATTGATGCAAGGTGCGGGTTTTGGCGGCTATGGAACTTCTATG

GGGGCACCGCGCTCGCCAGTCAGCCAAGTGCCCAATGGCATTTTGGGACAAGAGAAAGAA

GAATTGCATAACGGTGTGCTTCCAGGCCAGCGTAATTTTAGTGACCCACAACAGCTACAA

CAAAGGCAACCAGAAAATAACGACGGACAAGATGTTTCTATCGCGCCTCAGAGCACTGAA

CAACCGCCCCTCGCACAGATGAGTGAGCTTGACAGGTTTGGGTTGGCGGGTCTGCTGCGT

ATGATTCACAGCGAGAGTCCTGATGTTGCAAGTCTTGCTGTTGGCCAAGATTTGATGACA

CTAGGATTGGATTTAAATCAGGCGGAGCCTTTACACACATCGTTTGCTTCACCATTCGTT

TCATCGATGTCAGCAGTCCCTTTGGAACAAGACTTCTCTTTGCCGGGATGTTATAATGTC

GCCAATATCCAACCCCTCCAGTCTCGCATTCCCGGTTTCAGCGACGAGACCCTGTTTTTC

ATCTTTTACAGTATGCCTCGGGATATCATGCAGGAACTGGTGGCCGAGGAATTGATGGGC

CGCAAATGGCGGTACCACAAGCTTGAACGATGCTGGCTAACTCGCGATGAGACATATCCT

GGACCCGTTGACGTGGAACGTGGTGTGACGGAACGCGGCGTGTATCTTCTATGGGATTCA

TCTGCCTGGAAGAAAGTCCGACGTGAATTCATCCTTCGATATGAAGACTTGGATAACCGG

TTGGATCCTAATCGAGGCGCCGCTCGCCCACCTGGCGTGGTGCATCATGCCTCA

>CL76.Contig1_All 162 536 hypothetical protein PDIG_15760 [Penicillium digitatum PHI26] >gi|425779461|gb|EKV17513.1| hypothetical protein PDIP_31330 [Penicillium digitatum Pd1]

ATGATCACCATTGAAAACAACATGAATACCGGCACCACACAAAGAGCTCCCAAGACTCCG

AAACCAACTGCCTCTGTGCCAATGGTTCAAAATAAACGCTGGCCCCCTATGGCAAACCGA

AACGCCCTTCCTTACAAACTCACGCACCTGTCGAAAGAGAAGCTTGCCCGTGAAGCCACC

GCACCAGACCCTGATCTTCGCCGCTGCGTGGCACACTTTCGCCTGCACTGTGGTTCCGTC

TCCTGGACCGAGAATGACATGAAGTCTCGGATCAGCTCATTCGATTTCGAGGATACCGAC

GAGGAAGACGATGTGGAAGAGTTCAACATAGAACTGCCTGTCATCAGGAACAGAGTTGCA

GCCAACGAGACTCCA

>CL76.Contig2_All 274 648 hypothetical protein PDIG_15760 [Penicillium digitatum PHI26] >gi|425779461|gb|EKV17513.1| hypothetical protein PDIP_31330 [Penicillium digitatum Pd1]

ATGATCACCATTGAAAACAACATGAATACCGGCACCACACAAAGAGCTCCCAAGACTCCG

AAACCAACTGCCTCTGTGCCAATGGTTCAAAATAAACGCTGGCCCCCTATGGCAAACCGA

AACGCCCTTCCTTACAAACTCACGCACCTGTCGAAAGAGAAGCTTGCCCGTGAAGCCACC

GCACCAGACCCTGATCTTCGCCGCTGCGTGGCACACTTTCGCCTGCACTGTGGTTCCGTC

TCCTGGACCGAGAATGACATGAAGTCTCGGATCAGCTCATTCGATTTCGAGGATACCGAC

GAGGAAGACGATGTGGAAGAGTTCAACATAGAACTGCCTGTCATCAGGAACAGAGTTGCA

GCCAACGAGACTCCA

>CL77.Contig1_All 2 640 minus strand hypothetical protein PDIG_52160 [Penicillium digitatum PHI26] >gi|425782048|gb|EKV19979.1| hypothetical protein PDIP_21360 [Penicillium digitatum Pd1]

CAGTCTGGGACGTTTCCCAAGGTTTTCTCGGATTATAGAGAAGAGGATGTGAAGGAATTG

TCGAGACGGACAAACGAGTTCCTTGAGCAGGCTTTGACGGCGGAATTGGGGATTCCGGTT

ACGGCTGATACTGCTGCTCAGTGGAGGAACTTGGGAAGTCTTGGTTCGCATGATCTTAGT

GCTATGAGAGAGGCTCTTGGCATGCCGACGGCCGTGCTTGGTGCTTCTTTGTGCTCTGCG

AGGGGTCCGCCGTTCTGGAGTGCCCTTTTCCAATATCCCTCCTTTACTGTGTCTTATGAG

TCTGGGATTGATTATGTCCCCCGTTTCGACGCGTCAATTGAGGTCTTTGGTGGGAACAAA

ACTGTCAAGATCTGCTTTGATTCGCCTTACGTTAAGGGATTGCCTACCACTATGCACATT

CGTGAGAAATTGGAAGATGGCTCATTCCGGGAGTCTATGGTTCGGAAGACGTATGAAGAT

GCTTATACTTTGGAAATGAAGGAGCTTTATACATTTGTTGTTGACGGTAAACCGGCTAAA

ACCACTTCGGAGGATGCTAAGAAGGATCTTGAGATCTTCGGTATGATCATGAAGGCAGGC

ATGGAAGGACAAGCTAGACTTAACAAGGCGGTTCAAAAC

>CL77.Contig2_All 2 259 minus strand hypothetical protein PDIG_52160 [Penicillium digitatum PHI26] >gi|425782048|gb|EKV19979.1| hypothetical protein PDIP_21360 [Penicillium digitatum Pd1]

CAGTCTGGGACGTTTCCCAAGGTTTTCTCGGATTATAGAGAAGAGGATGTGAAGGAATTG

TCGAGACGGACAAACGAGTTCCTTGAGCAGGCTTTGACGGCGGAATTGGGGATTCCGGTT

ACGGCTGATACTGCTGCTCAGTGGAGGAACTTGGGAAGTCTTGGTTCGCATGATCTTAGT

GCTATGAGAGAGGCTCTTGGCATGCCGACGGCCGTGCTTGGTGCTTCTTTGTGCTCTGCG

AGGGGTCCGCCGTTCTGG

>CL78.Contig1_All 234 1073 minus strand Translational initiation factor 2 beta [Penicillium digitatum PHI26] >gi|425783361|gb|EKV21215.1| Translational initiation factor 2 beta [Penicillium digitatum Pd1]

ATGGATACCAACGGCCAAGTTACAGACGCCCCCCCGGCTGAGAAGCCCACAGGCGAGAAG

GATGTCGACGAGGTCACTGAGATGTTCAAGGGTCTCTCAAAAAAGAAGAAGTCATCCAAG

AAGTCTAAGACCACCGAGGCTGGCGATGACGAGGAAGCCCCCGCCGCCGCCGATGGCGAA

TTCGATCCCTCCGCGCTGAAGAAGAAAAAGAAGAGCAGCAAGTCTAAGAAGGTCGACCCC

AATGACTTCGATGCCAAGCTTGCTGAGGCTGGTATTGCTGAGGAGGCTGGCGAGGATCAG

CCCGAGGAGCTGCCCGAGGGAGACCTCGAGGCCGGCACCGGTATCTGGGCACACGACGCC

ACACAAGCCATCCCCTACTCGCTCCTCGTCTCCCGCTTCTTCTCCCTCATCGAGAGCCAC

CACCCCGACCTTCTGTCCAGCGGCGCCAAGTCCTACAAGATTCCCCCGCCCCAGTGCCTG

CGTGAAGGTAACCGTCGCACCATTTTCGCCAACATTGCCGATATCTGCAAGCGCATGAAG

CGAAGCGAGGAACACTGCACCCAATTCCTGTTCGCTGAACTCGGTACATCAGGCAGTGTC

GACGGTAGCCGTCGCCTGGTCATCAAGGGACGATTCCAACAGAAGGGTATCGAATCGGTT

CTGCGTCGCTATATTGTCGAGTACGTCACATGCAAGACTTGCCGCAGCCCCGACACCGAG

CTCAACAAGGGAGAGAACCGTCTGTATTTCGTTACTTGCAACTCCTGTGGATCCCGTCGT

TCCGTCGCCGCCATCAAGACCGGTTTCCGTTCCCAGATCGGTCGCAGAAAGCGTGTGGGC

>CL78.Contig2_All 178 1017 Translational initiation factor 2 beta [Penicillium digitatum PHI26] >gi|425783361|gb|EKV21215.1| Translational initiation factor 2 beta [Penicillium digitatum Pd1]

ATGGATACCAACGGCCAAGTTACAGACGCCCCCCCGGCTGAGAAGCCCACAGGCGAGAAG

GATGTCGACGAGGTCACTGAGATGTTCAAGGGTCTCTCAAAAAAGAAGAAGTCATCCAAG

AAGTCTAAGACCACCGAGGCTGGCGATGACGAGGAAGCCCCCGCCGCCGCCGATGGCGAA

TTCGATCCCTCCGCGCTGAAGAAGAAAAAGAAGAGCAGCAAGTCTAAGAAGGTCGACCCC

AATGACTTCGATGCCAAGCTTGCTGAGGCTGGTATTGCTGAGGAGGCTGGCGAGGATCAG

CCCGAGGAGCTGCCCGAGGGAGACCTCGAGGCCGGCACCGGTATCTGGGCACACGACGCC

ACACAAGCCATCCCCTACTCGCTCCTCGTCTCCCGCTTCTTCTCCCTCATCGAGAGCCAC

CACCCCGACCTTCTGTCCAGCGGCGCCAAGTCCTACAAGATTCCCCCGCCCCAGTGCCTG

CGTGAAGGTAACCGTCGCACCATTTTCGCCAACATTGCCGATATCTGCAAGCGCATGAAG

CGAAGCGAGGAACACTGCACCCAATTCCTGTTCGCTGAACTCGGTACATCAGGCAGTGTC

GACGGTAGCCGTCGCCTGGTCATCAAGGGACGATTCCAACAGAAGGGTATCGAATCGGTT

CTGCGTCGCTATATTGTCGAGTACGTCACATGCAAGACTTGCCGCAGCCCCGACACCGAG

CTCAACAAGGGAGAGAACCGTCTGTATTTCGTTACTTGCAACTCCTGTGGATCCCGTCGT

TCCGTCGCCGCCATCAAGACCGGTTTCCGTTCCCAGATCGGTCGCAGAAAGCGTGTGGGC

>CL79.Contig1_All 159 1091 minus strand hypothetical protein PDIP_58160 [Penicillium digitatum Pd1] >gi|425773390|gb|EKV11746.1| hypothetical protein PDIG_48780 [Penicillium digitatum PHI26]

ATGTCCGAATCCCCTCCACCTCCGACTTGGAGCATCAGCAGCATGGCCAACAGTGCTGCG

CAGTTCATGCGACTGCCAGTTCTTGCGTCGTCTGGCCTGGCTGTTGTTGCAAGTGGGCTC

CTTTACTTCAAGCAAAATGAACTAATCTACCCCCGCAATGTCCCTGTGGATGCGCGCACA

AATGTCCCCAGTCCTCGCCAATTTGGAATCAGCGACTTTGAGGACCTCCAGATCCCCACG

CCCGACGGGGAGTCCTTGCATGCCCTTTTCCTCCGGCAACGACCAGGCCGTTTCTCTCGC

AATCTGACAGTCCTAATGTTCCACGGAAATGCAGGAAACATCGGCCACCGCGTCCCTATC

GCCAAAGCCGTACAGGATACGCTTCAGTGCAATGTTTTCCTGCTGGAATACCGGGGATAC

GGGATGTCTACAGGCACGCCGGATGAAGCCGGTCTTAAGATCGATGCTCAAACTGGCCTT

GATTACCTACGACAGCGCTCAGAGACAAGGGATACCGAGATCGTTATCTACGGACAAAGT

TTAGGTGGTGCTGTGGCTATCAATCTAGTTGCCACCAACGAGGAGAAAGGTGACATCGTA

GGACTGATCTTAGAGAACACTTTCCTGAGCATTCGCAAGTTGATCCCAAATGTCTTTCCC

CCAGCACGGTATCTGGCACGTTTCTGCCACCAATATTGGATCAGTGAAGATGTGCTGCCT

AAGATCACCAAAACTCCAGTTCTTTTCCTGAGTGGACTCAAGGATGAGCTTGTTCCCCCG

TCGAATATGACCCAATTATTTGCCGTTTGCCAGTCTGAATGCAAGATCTGGCGTACGCTC

CCGAACGGTGGTCACAATGACTCTGTGGCTGAGCCAGGCTACTTCGAACACATTCTTTCC

TTCATTACGGAAGAAGTGCTACCTCCTAAACAG

>CL79.Contig2_All 159 1091 minus strand hypothetical protein PDIP_58160 [Penicillium digitatum Pd1] >gi|425773390|gb|EKV11746.1| hypothetical protein PDIG_48780 [Penicillium digitatum PHI26]

ATGTCCGAATCCCCTCCACCTCCGACTTGGAGCATCAGCAGCATGGCCAACAGTGCTGCG

CAGTTCATGCGACTGCCAGTTCTTGCGTCGTCTGGCCTGGCTGTTGTTGCAAGTGGGCTC

CTTTACTTCAAGCAAAATGAACTAATCTACCCCCGCAATGTCCCTGTGGATGCGCGCACA

AATGTCCCCAGTCCTCGCCAATTTGGAATCAGCGACTTTGAGGACCTCCAGATCCCCACG

CCCGACGGGGAGTCCTTGCATGCCCTTTTCCTCCGGCAACGACCAGGCCGTTTCTCTCGC

AATCTGACAGTCCTAATGTTCCACGGAAATGCAGGAAACATCGGCCACCGCGTCCCTATC

GCCAAAGCCGTACAGGATACGCTTCAGTGCAATGTTTTCCTGCTGGAATACCGGGGATAC

GGGATGTCTACAGGCACGCCGGATGAAGCCGGTCTTAAGATCGATGCTCAAACTGGCCTT

GATTACCTACGACAGCGCTCAGAGACAAGGGATACCGAGATCGTTATCTACGGACAAAGT

TTAGGTGGTGCTGTGGCTATCAATCTAGTTGCCACCAACGAGGAGAAAGGTGACATCGTA

GGACTGATCTTAGAGAACACTTTCCTGAGCATTCGCAAGTTGATCCCAAATGTCTTTCCC

CCAGCACGGTATCTGGCACGTTTCTGCCACCAATATTGGATCAGTGAAGATGTGCTGCCT

AAGATCACCAAAACTCCAGTTCTTTTCCTGAGTGGACTCAAGGATGAGCTTGTTCCCCCG

TCGAATATGACCCAATTATTTGCCGTTTGCCAGTCTGAATGCAAGATCTGGCGTACGCTC

CCGAACGGTGGTCACAATGACTCTGTGGCTGAGCCAGGCTACTTCGAACACATTCTTTCC

TTCATTACGGAAGAAGTGCTACCTCCTAAACAG

>CL80.Contig1_All 194 1450 minus strand Bacterial hemoglobin [Penicillium digitatum Pd1] >gi|425770447|gb|EKV08920.1| Bacterial hemoglobin [Penicillium digitatum PHI26]

ATGAGTTCCAATCCAGGAATGACCCCAAACCAAATTGCGCTCATCAAGGCGACGGTTCCG

GTTCTCGCAGAACATGGCAACACCATCACGACCGTCTTCTATCGCAATATGCTCGAAGCC

CACCCGGAGCTGAACACCGTATTTAACACTGCCAATCAGGTCAACGGCCATCAGCCTCGT

GCGTTGGCTGGTGCACTCTACGCATATGCGTCGCACATCGATGATCTCGGCGCACTTAGC

TCAGCGGTTGAGTTGATCTGCAACAAGCACGCCTCTCTGTATATCCAGCCCGACGATTAC

AAGATCGTTGGAAAGTACCTCCTCGAAGCTATGGGCGAGGTGCTTGGCGCAGCATTGACC

CCGGAAATTCTCGACGCATGGGGCACAGCCTACTGGCAGCTCGCCGATATCTTGATCGGC

CGCGAAAAGCAGCTTTATGAACAAGCCGAGGGCTGGACTGACTGGAGGGATTTCAAGATC

GTGAACAAAGTCAAGGAATCCGAGGAAATCACATCCTTCTATCTCGCTCCTGTCGATGAA

AAGCCATTGCCGGTCTTCCAGCCCGGCCAGTACATCTCCGTTCAAACGCACGTCCCCGCT

CTCAAGTGCCTGCAGGCTAGACAGTACTCTCTCAGCGACCAGCCAAAGCCGGACTACTAT

CGCATCAGCGTGAAAAGAGAATTGGGCCTCAACCCAGCTGCTCCAGGTGCTGCCGCCCAT

CCGGGTTACATCTCGAATGTGCTACATGACACATTCAACATCGGCGACAAGCTTAAGGTG

TCACACCCCTGCGGCGACTTTTTCCTTACCCCTGTCGACTCGGAAGCCGGCAATCCAATT

GTGTTAATCTCAGCCGGCGTGGGCTTAACGCCACTGACGTCGATGCTGAACACCTTGATC

TCCAAATCGCCAACAACGCGCAAACTGCACTTCATCCATGGCGCACGTTCGTCGGGCGCG

CGTGCTTTCAAGGAGCACATCTCTGAGCTTACAACACCATTCCCCTCTATTCGAACAACA

TTCTTTACTAGTCACCCCTCCGAGGGAGAGAAGGAAGGCGTGGACTATGACCATGCCGGG

CGGGTGGACTTGACCAAGGTGGATGATCAAGGCTTATTCCTCGATGATGCCAAGACAGAT

TATTATATTTGTGGACCCGGAAAGTTCATGATCGATGTCGAGTCGGGTCTCAAAGCCAGA

GGCGTGGGAGCTGATCGCATCAAGATGGAATTGTTTGGAACCGGAGGTATTCCTCAT

>CL80.Contig2_All 2 208 Pc12g14620 [Penicillium chrysogenum Wisconsin 54-1255] >gi|211582885|emb|CAP81089.1| Pc12g14620 [Penicillium chrysogenum Wisconsin 54-1255]

CCGGTTCTCGTGGAACATGGCAACACCATCACCACTGTCTTTTATCGCAACATGCTTGCA

GCCCATCCGGAGTTGAACACCGTGTTCAACACTGCTAACCAGGTCAATGGCCATCAACCT

CGCGCGTTGGCCGGTGCACTCTACGCATATGCATCGCATATCGATGATCTCGGCGCACTT

GGCTCCGCGGTTGAGTTGATCTGCAAC

>CL80.Contig3_All 3 332 minus strand Pc12g14620 [Penicillium chrysogenum Wisconsin 54-1255] >gi|211582885|emb|CAP81089.1| Pc12g14620 [Penicillium chrysogenum Wisconsin 54-1255]

TACTGGCAGCTCGCCGATATCATGATCGGCCGCGAAAAGCAGATTTACGAACAGGCCGAG

GGTTGGACTGACTGGAGAGACTTCAAGATTGTGAACAAAGTCAAGGAATCTGAGGAAATC

ACCTCCTTCTATCTCTCCCCCGTTGATGAAAAGCCACTGCCGGCCTTCCAGCCCGGCCAA

TACATTTCCATCCAAACCTACGTCCCAGCTCTCAAGTACCCGCAGGCCAGACAGTACTCA

CTCAGCGACCAGCCGAAGCCGGACTATTATCGCATCAGTGTTAAGAGAGAACTAGGCCTC

AGCCCTGCCGCTCCAGGTGCTGCCGCCCAT

>CL81.Contig1_All 2 199 NAP family protein [Penicillium digitatum PHI26] >gi|425774965|gb|EKV13256.1| NAP family protein [Penicillium digitatum Pd1]

CCCGATGCGCTCAGCTACTACGGTATGTAGATTTCCGGATAGCGTGTGAGATGGTTTTTT

CCCCCACTAACTGTGACTGCAGTTCAATCCTTCCAGATCGGCGAGGCGCTCGAAGATATG

GACTTCGACATGGAGGAGATTGATGGAGAAGACTCTGATGAAGAAAGTGAGTTGCGTCCG

CGCAAGAAGGCTCGCAAC

>CL81.Contig2_All 175 1125 NAP family protein [Penicillium digitatum PHI26] >gi|425774965|gb|EKV13256.1| NAP family protein [Penicillium digitatum Pd1]

GTGCGCCACTCTGCTAAGCTCATGACCCCCCTGATCCAGAAGCGCAGCGAGATCGTCAAC

ATTCCCGAAGTGCAGGCTGAGTTCTGGATGCGCGTGTTCTCCAGCGCTCCCCCCAACATC

GACGAGTACATTCTGTCCAGCGACGCTCAAGTCCTCGGCGAGAGCCTCAAGAACATGAAC

GTGGAGCGATTTGAGCTTGATGCCCAAGGCAACGGCGAGCCCCGCAGCTTGCGCTTCACC

TTTGAGTTCAAGACCGGCGAGGAGAACCCATTCTTCACCAACGAGAAGCTCGTCAAGGAG

TTCTACTGGCGCCAGGAGGTCTCCAAGAACGCCGCTGGCAAGAGCCGCACCTGGGAGGGT

CTCGTTTCCGAGCCCGTTCGCATCAACTGGAAGAAGGATATGGATTTGACCAAGGGCATG

CTCGATGCTGCGTGTGACCTTGCTGAGGCTGAGAAGAAGAAGGGTGGTGACCGTAAGAAG

TTGCCCGAGTTCGCTGCTCTCGTTAAGAAGATCGAGGAGGCTGAGGCTGATGCCAGCGCC

GAGGATGACGAGGATGATGAGAACCCTAGCCCCGTTGGCATGAGTTTCTTCGGATTTTTC

GGATACCGCGGTCGTGACGTCTCTGCGGCTCAGTCCAAGGAAGCTGCCCAGGAGATCGAG

GCTCGCTGGGCCAAGATCCAGAAGGGCGAGGATGTTGAAGATGCTGGTGACTCCGAGGAT

GAGGATGAGGAGGATGACTCCAACAGTCTTGAGGAAATCGAGATCTTCCCTGACGGAGAT

GACCTCGCTGTTAACATTGCCGAAGACCTCTGGCCCGATGCGCTCAGCTACTACGTTCAA

TCCTTCCAGATCGGCGAGGCGCTCGAAGATATGGACTTCGACATGGAGGAGATTGATGGA

GAAGACTCTGATGAAGAAAGTGAGTTGCGTCCGCGCAAGAAGGCTCGCAAC

>CL81.Contig3_All 250 1314 NAP family protein [Penicillium digitatum PHI26] >gi|425774965|gb|EKV13256.1| NAP family protein [Penicillium digitatum Pd1]

ATGTCTAACGATCAGTCCCTCCTTGACCGCGTCGAGATGCCTAGCATCCCTGAGGCGGCT

ATGAAGAAGATTGCGATTTTGGAAGAGCAGTTCTCGCGTGCTGAGGTCGAACGGTTGCGC

CACTCTGCTAAGCTCATGACCCCCCTGATCCAGAAGCGCAGCGAGATCGTCAACATTCCC

GAAGTGCAGGCTGAGTTCTGGATGCGCGTGTTCTCCAGCGCTCCCCCCAACATCGACGAG

TACATTCTGTCCAGCGACGCTCAAGTCCTCGGCGAGAGCCTCAAGAACATGAACGTGGAG

CGATTTGAGCTTGATGCCCAAGGCAACGGCGAGCCCCGCAGCTTGCGCTTCACCTTTGAG

TTCAAGACCGGCGAGGAGAACCCATTCTTCACCAACGAGAAGCTCGTCAAGGAGTTCTAC

TGGCGCCAGGAGGTCTCCAAGAACGCCGCTGGCAAGAGCCGCACCTGGGAGGGTCTCGTT

TCCGAGCCCGTTCGCATCAACTGGAAGAAGGATATGGATTTGACCAAGGGCATGCTCGAT

GCTGCGTGTGACCTTGCTGAGGCTGAGAAGAAGAAGGGTGGTGACCGTAAGAAGTTGCCC

GAGTTCGCTGCTCTCGTTAAGAAGATCGAGGAGGCTGAGGCTGATGCCAGCGCCGAGGAT

GACGAGGATGATGAGAACCCTAGCCCCGTTGGCATGAGTTTCTTCGGATTTTTCGGATAC

CGCGGTCGTGACGTCTCTGCGGCTCAGTCCAAGGAAGCTGCCCAGGAGATCGAGGCTCGC

TGGGCCAAGATCCAGAAGGGCGAGGATGTTGAAGATGCTGGTGACTCCGAGGATGAGGAT

GAGGAGGATGACTCCAACAGTCTTGAGGAAATCGAGATCTTCCCTGACGGAGATGACCTC

GCTGTTAACATTGCCGAAGACCTCTGGCCCGATGCGCTCAGCTACTACGTTCAATCCTTC

CAGATCGGCGAGGCGCTCGAAGATATGGACTTCGACATGGAGGAGATTGATGGAGAAGAC

TCTGATGAAGAAAGTGAGTTGCGTCCGCGCAAGAAGGCTCGCAAC

>CL82.Contig1_All 72 218 Peroxiredoxin-6 [Penicillium digitatum Pd1] >gi|425769487|gb|EKV07979.1| Peroxiredoxin-6 [Penicillium digitatum PHI26]

ATGGCTTCCAGAATTGGCCCTCAGATGTTCCGTGCATTCAGAGCTGCTCCATGGCCCTCG

TGGTCTGCACAGGTGCCTCGTGCGCCCGCTTTCCGCCGCTGCATCTCGGCCAGCATGGAA

CAACCGCGTCTGCGCCTGGGCTCAATC

>CL83.Contig1_All 2 1300 Pyruvate decarboxylase [Penicillium digitatum PHI26] >gi|425779698|gb|EKV17735.1| Pyruvate decarboxylase [Penicillium digitatum Pd1]

GCGGGCATCTCCTGCTACGTCGAACGTCTGAATGACCCCAAGGACGCAGCCACTCTGATC

GACAGTGCGATTCGGGAATGTTGGATCCGCAGCCGTCCGGTGTATATCACTCTGCCAACG

GATTTGGTCGCTGCCAAAGTCAACGGCGATCGCCTGAAGACCCCGATTGATCTCTCACTG

CCTAAGAATGACCCGGAGAAGGAGGACTACGTGGTCGATGTCGTCCTGAAGTATCTCCAT

GCCGCAAAGAACCCTGTTATCTTGGTTGACGCCTGTGCCATTCGTCACCGCGCACTAGAA

GAAGTGCGCGAGTTGGTGGAGAAGTCTGGCTTGCCAACCTTTGTGACTCCGATGGGCAAG

GGAGCCGTGAACGAGGATCACAAGAACTTCGGTGGTGTCTATGCTGGTAATGGATCTAAC

CCTGGAGTTAGTGAGGCTGTCGAGTCCTCCGACCTCATTCTGAGCGTTGGAGCGATCAAG

TCTGACTTCAACACTACCGGTTTCACTTATCGTGTTGGACAGCTGAACACCATTGACTTC

CACAGTACCTTTGTGAGAGTGCGGTACTCGGAGTACCCTGATATCAACATGAAGGGCGTT

CTGAGGAAGGTCGTTGAACGGATGAATCCTCTGACCCCGGCCCCTATCCCTCTTATTACG

AACCGACTGCCCGAAAGCGAGCAAAGCTCTACTGATCAAACTATTACCCACAAGTGGCTC

TGGCCCATCGTCGGACAGTGGCTAAGGGCAAAGGATATCGTCCTTACCGAGACTGGCACT

GCCAACTTCGGTATCTGGGATACTCGCTTCCCCGCCAACGTGACTGCCATCAGCCAAGTT

CTTTGGGGCAGTATTGGATTTGCCATGGGAGCCTGTCAGGGTGCTGCCCTCGCTGCTAAG

GAACAGAAAGACCGCCGAACTATCCTCTTCATCGGCGACGGAAGCATTCAACTCACGGTG

CAGGAACTCAGCACTATCCTGAAGAATAAGTTGAACCCTATCGTCTTCGTTATCTGTAAC

GACGGCTACACTATCGAGCGATACATCCACGGATGGGATGCGGTTTACAACGACATCCAG

CCTTGGGAATTTGTTAACATTCCCAAGGTCTTCGGAGCCAAGGATAACTACCAAGGCTAC

CGTATCAAGACCCGTGACGAACTCAACCAGCTGTTCGCTGACGAGGACTTCAACGTGTCC

GACAAGCTTCGACTGGTCGAGTTGTACATGCCTCGCGATGACGCCCCCGCGGCCTTGAAG

TTGACCGCCGAGGCTGCTGCGCAACGCAACAGCGGCAAA

>CL84.Contig1_All 215 3406 putative beta-glucosidase E [Penicillium digitatum PHI26] >gi|425782519|gb|EKV20425.1| putative beta-glucosidase E [Penicillium digitatum Pd1]

ATGGTTTCCCCACGTGCCAAGGTCGGAGCCTCGCGCTCGGCCAAATCCTCCAACCCCACG

CATCGCGGCTATACGTCACTGCAGAATCCAACGATCGAACACTCAACCTCAGAGAGCAAT

CGGGAACAAAAGGATGAATTGGATACTCCTTCAAGTGGAGATGAATGCTCTGAGCAGGTT

TATACAAAACTTCTACCTTTAAAACACGATAAGGCGCGAGATCCACGACCCTCGGATTCC

AAAGCAAATGACCGGATTCTGACTTCCATCAATGTCCCCGATGACGAGCGTGATATTTCA

AAGCACATTAACAAGCTTCTCGAGGTGGATCACACTGCGCTGTCGGACTCACTCGCCACA

GATTTGGAGGAGGGATACTCGGGCAGAGACTATCTGGTTCTTAGTGATCGAGATAGTGAC

GACCATGGCATCCCACCGGACAAGGGCTGGTTGCTAAAAGACAAAGAGAACGAATATGAG

AAGAAAGGCATGGACTGGAGGTCACTGTTCCGTCTGCATTCGTGGTGGAATATTGTCGGC

GCGTTGACAATCGCCCTTCTGGTAGTCTGGCTTTCGATCAAGGGACTACCCTGGTCCTCT

GCCGGAGCTGCCTTTGATGAATATCAAGCGGTTCCCTGGTACCCTACTCCCCTTGGCGGA

ACGAGTGAACAATGGAAGGAAAGTTATATCAAGGCCCAACACATGGTCAAAGAAATGACT

CTTGCCGAAAAAGTGAACGTCACAACCGGAACAGGATGGCAAATGGGACTCTGTGTTGGA

AATACTGGACCTGCTGAACACGTCAAATTCCCTTCTCTTTGCCTACAAGACGGCCCACTT

GGACTCCGATTCGTGCAAAACATTACAGCCTTCCCCGCAGGAATCACCACGGGTGCGACA

TGGAACCGTGAGCTTATGCAGAAGAGGGGCTATGCTCTTGGAAAGGAAGCGCGGCTGAAA

GGCGTGAACATCATTCTCGGTCCATCCATGGGGCCGCTCGGCATGATGCCTGCTGGTGGT

CGTAATTGGGAGGCATTTGGTTCAGATCCGGTTTTGCAAGGCGTGGCTGCGGCAGAGACC

ATTCGCGGTATCCAGCGGAACGGGGTCATGGCGACGGCGAAACATTTTATTGGGAATGAA

CAAGAACACTTCCGCCAGTCGTTTGAATGGGGTACTCCAACTGCACTCTCGTCGAATATT

GATGATCGTGCCCTGCACGAGGTCTTTGCCTGGCCCTTCGCGGAGAGTGTACGTGCCGAC

GTGGCTAGTGTCATGTGCTCCTACCAAATGGTCAACAACAGCTATGCTTGCGAGAATAGT

AAGCTCATTAATGGTATTCTGAAAGATGAGCTCGGCTTTCAGGGATTCGTGCAGTCGGAC

TGGCTGGCTCAGCGCTCAGGCGTTCACAGCGCCATTGGAGGCCTTGATATGAGCATGCCC

GGTGATGGCCTCCATTGGGTCGACGGTGTCCCGCTCTGGGGAAGTGAACTGACCCGTGCA

GCGTTGAACACGTCTGTTCCCATGGAGCGTTTGAACGATATGGTCACTCGTATCGTGGCC

GCCTGGTACCATTTCCAACAGGACTCTTGGGAACCCCCATCACCCGAGGGGGATGGCGGT

CCGAACTTCTCCTCCTGGACGAAAGAGCGAGTCGGACACTTACACGAAGGCTCACCGGAT

AATGACGCAACAGGCGTGGTCAACAAATTTGTCGACGCACAAGGAACCGGCGAGCACGCT

CATTCCATCGTTGCTCGCCGTATCGCCGCAGAAGGCACTGTGCTTTTGAAAAATGTCAAC

AATACACTTCCACTCTTGCGTACACAGTCCGCATCCGATGGCAAATACCGGGTCGGTGTA

TACGGTGAAGACGCTGGTCCCGGCCAAGGACCGAACGCTTGCGCTGACCGAGGATGTAAT

CAAGGCACCTTGGCCTCTGGTTGGGGTAGCGGGGCTGTAGACTTCCCTTACTTGATCAGT

CCGTGGGAAGCACTGCAAAATGCGTGGCCGAAAGACTCTGTGGATGTGAAGGCTTACTTG

ACGAACAACGTTGCACCTCAAGACCTCGGAGCGCAGGACCTCTGTTTGGTTTTTGTCAAC

GCAGATGGAGGCGAAGGATTTATCCGCAGTGACGGCATTGACGCAGACAGAAATGACCTC

TTTCTGCAAAAGAACGGCACTCAGTTGGTAGAAGCGGCGGCCAAACACTGCGGCGGTGGC

CAAGGGAGAACCGTGGTGATTGTACACTCCATTGGTCCTGTTGTAATGGAGTCTTGGATT

AACCTTCCTGGTGTGCACGCTGTACTGTATGCAAATCTACCGGGGCAAGAAAGCGGGAAT

GCATTAAAGGACGTTCTGTTTGGAGATGTCGATGCCAGTGGACGACTACCATATACCATC

GGACGAAGCCTACAAGACTACGGCCCAGGGGCGCAAGTCCTGTATAAGCACACAGACCCA

TCCGTTCCACAGGTCAATCTTGACCAGGGCCTGTACGTTGATTACCGCTATTTCGACAAG

TTCAACATCACTCCCCGATTCGAGTTCGGCTTCGGTCTGTCCTACACAACCTTCGACTAT

ACTGCACTGAGCATAGAAAAACTGCAGAAGAAGTCCCAATGGCCTTCCAAACGGCCCAAA

AACGAAGCCGACCCTCCCACATACGATGACAAACAGAGTGACCCTACTAGCAGTTTATTC

CCCCCCGGTTTCCGTGCACTCCGCAAATATATCTATCCATACCTCGGGACCTTGGACGGA

ACCGAACCCGGCCCATACCCATACCCGGAAGGATACAAGAAACAGCAGAAACCATCCGCC

GCAGGTGGCGGGCTGGGCGGGAATCCCTCCCTATACGAGGCTATGCTCGAGCTCACCATT

CAAGTGACAAACACCGGCACACGCACAGGACAAGATGTGATCCAGGTCTATGTCTCCTTC

CCAGAAGATGTCGCCGAGCACCGTGGACTGGGCTGGTCGCGCCAGTCGATTGAGTTCCCT

GAGCGGGTCCTGCGGAATTTTTCCAAAGTCCTCCTGGAACCGGGTGAGACCAGAAATGTC

AAGATGACACTGACTCGGAAGGACCTCAGCTTTTGGAGTGTTCGCGCGCAGAACTGGGTG

TTGCCTACTGAAGGTAAATTCCGTATCTGGGCTGGGCGTAGTTCTAGGGATCTACCACTC

GTGGTCGAGTTT

>CL84.Contig2_All 886 1068 minus strand putative beta-glucosidase E [Penicillium digitatum PHI26] >gi|425782519|gb|EKV20425.1| putative beta-glucosidase E [Penicillium digitatum Pd1]

GCCACTCATACATCACAGCAAGCGGTTCCCTGGTACCCTACTCCCCTTGGCGGAACGAGT

GAACAATGGAAGGAAAGTTATATCAAGGCCCAACACATGGTCAAAGAAATGACTCTTGCC

GAAAAAGTGAACGTCACAACCGGAACAGGATGGCAAATGGGACTCTGTGTTGGAAATACT

GGT

>CL84.Contig3_All 215 1003 minus strand putative beta-glucosidase E [Penicillium digitatum PHI26] >gi|425782519|gb|EKV20425.1| putative beta-glucosidase E [Penicillium digitatum Pd1]

ATGGTTTCCCCACGTGCCAAGGTCGGAGCCTCGCGCTCGGCCAAATCCTCCAACCCCACG

CATCGCGGCTATACGTCACTGCAGAATCCAACGATCGAACACTCAACCTCAGAGAGCAAT

CGGGAACAAAAGGATGAATTGGATACTCCTTCAAGTGGAGATGAATGCTCTGAGCAGGTT

TATACAAAACTTCTACCTTTAAAACACGATAAGGCGCGAGATCCACGACCCTCGGATTCC

AAAGCAAATGACCGGATTCTGACTTCCATCAATGTCCCCGATGACGAGCGTGATATTTCA

AAGCACATTAACAAGCTTCTCGAGGTGGATCACACTGCGCTGTCGGACTCACTCGCCACA

GATTTGGAGGAGGGATACTCGGGCAGAGACTATCTGGTTCTTAGTGATCGAGATAGTGAC

GACCATGGCATCCCACCGGACAAGGGCTGGTTGCTAAAAGACAAAGAGAACGAATATGAG

AAGAAAGGCATGGACTGGAGGTCACTGTTCCGTCTGCATTCGTGGTGGAATATTGTCGGC

GCGTTGACAATCGCCCTTCTGGTAGTCTGGCTTTCGATCAAGGGACTACCCTGGTCCTCT

GCCGGAGCTGCCTTTGATGAATATCAAGCGGTTCCCTGGTACCCTACTCCCCTTGGCGGA

ACGAGTGAACAATGGAAGGAAAGTTATATCAAGGCCCAACACATGGTCAAAGAAATGACT

CTTGCCGAAAAAGTGAACGTCACAACCGGAACAGGATGGCAAATGGGACTCTGTGTTGGA

AATACTGGT

>CL84.Contig4_All 215 838 minus strand putative beta-glucosidase E [Penicillium digitatum PHI26] >gi|425782519|gb|EKV20425.1| putative beta-glucosidase E [Penicillium digitatum Pd1]

ATGGTTTCCCCACGTGCCAAGGTCGGAGCCTCGCGCTCGGCCAAATCCTCCAACCCCACG

CATCGCGGCTATACGTCACTGCAGAATCCAACGATCGAACACTCAACCTCAGAGAGCAAT

CGGGAACAAAAGGATGAATTGGATACTCCTTCAAGTGGAGATGAATGCTCTGAGCAGGTT

TATACAAAACTTCTACCTTTAAAACACGATAAGGCGCGAGATCCACGACCCTCGGATTCC

AAAGCAAATGACCGGATTCTGACTTCCATCAATGTCCCCGATGACGAGCGTGATATTTCA

AAGCACATTAACAAGCTTCTCGAGGTGGATCACACTGCGCTGTCGGACTCACTCGCCACA

GATTTGGAGGAGGGATACTCGGGCAGAGACTATCTGGTTCTTAGTGATCGAGATAGTGAC

GACCATGGCATCCCACCGGACAAGGGCTGGTTGCTAAAAGACAAAGAGAACGAATATGAG

AAGAAAGGCATGGACTGGAGGTCACTGTTCCGTCTGCATTCGTGGTGGAATATTGTCGGC

GCGTTGACAATCGCCCTTCTGGTAGTCTGGCTTTCGATCAAGGGACTACCCTGGTCCTCT

GCCGGAGCTGCCTTTGATGAATAT

>CL85.Contig1_All 23 1027 minus strand hypothetical protein PDIG_90580 [Penicillium digitatum PHI26] >gi|425783473|gb|EKV21321.1| hypothetical protein PDIP_07500 [Penicillium digitatum Pd1]

ATGGTCTATCGCTCAGGGCAATTGGTTCAGACAGCTTTAGCTGTCCGCACCATTTCCTTC

GTCCCTCGCCGAACATTGACCTCCACGGCCATCCGTTACGCTTTGAACGATGGCGAAACC

AAAAAGCCGACCGGTTTGTTTTCTTCCTTGAAGAACATATTTTTGGGGGGATCTAGCCAG

AAACCCAAAGTCGCCCACCGACCCTCGGCCCCTAAGAAGGAAGAAGGTGGTCTAGGAACC

TCCATCTTCGGATCAGACTTCACTTCACCAACAACCGGTCCCATGCCATCCCGGCCAACG

GGGGAACAACCAAAAGAAGGCATCTCCGGAAAGCTGGAGGACCGCGACTTGAGTCGCCTG

CAAGTGGGACTGGGACCCGACCCTAAAGCCCAGCTACGATGGGAGAGGAAGATGGCCGTC

CGCGAGGTCCGGAAACGCGGCCGTGTGCCGACGAAGGTGCAAATTAAGCGCACAGAGCGC

GAATCGCTTTCTAAATCTCACTGGATCAAGACGTCACTGAAGAAGCTGGGTCCTCTGGCC

CGCCAAATCGCCGGCAAGAATATCGACGAGGCCATCCTCCAGATGCGCTTCAGCAACAAA

AAGGCCGCTAAGGACGTACTGGAACACCTGGAACACGCAAAGAACGTCGCCATTGTCCGT

GCCGGCATGGGCCTTGGGGCCTCTAACTCCGAGCCATCGAAACCTATCACTGTCACACTG

AAGACTGGCGAGCGACAGAAGATTGCCAACCCCACTGACATCTACATCCAAGAAGCCTGG

GTCAACCGTGGTCCCTATGGATATGAGATGGATCATCGCGCCCGTGGTCAGATCAACCGT

ATGCGTCCGCCTACCACAAGCATTTCAGTCCTGCTGAAGGAAGAGAAGACTCGTATCCGG

GAGTGGGAGGACCGGGAGGCTAAGGCCCAGCGGGAGCGCAGGTCTCAGCTCTGGACACAA

CTGCCGGATCGCAAGATCTCTGCGCAGAACCAGTATTACAGCTGG

>CL85.Contig2_All 83 184 minus strand hypothetical protein PDIG_90590 [Penicillium digitatum PHI26] >gi|425783474|gb|EKV21322.1| hypothetical protein PDIP_07510 [Penicillium digitatum Pd1]

ATGAACACCCCACCCCTGTTGCGAATCAAAACAGTTACGGGGTATTTCCTCCAGGATGAC

CCCATTACTGATCCCGATACTTTCGACTACGTAAGAGAGTCC

>CL86.Contig1_All 2 265 minus strand Pc15g01030 [Penicillium chrysogenum Wisconsin 54-1255] >gi|211584950|emb|CAP82989.1| Pc15g01030 [Penicillium chrysogenum Wisconsin 54-1255]

CTCTCCGAGTACGGCTGCAACACCAACAAGCGCGAGTGGCAGGAGGTCAAGGCTCTTTAC

TCCACCAAGATGACCCCCGTCTACTCCGGTGGTCTGGTCTACGAGTACTCTGAGGCCGGT

AACAAGTACGGTCTGGCCAAGATCGACGGTGACAAGGTCACCACCAACAAGGATTACGAT

GCTCTCAAGAAGGCCTTCGAAGCTACCGAGAACCCCTCCGGCAACGGCGGCTACAACTCC

ACCGGCGGTGCCTCCGGCTGCCCC

>CL86.Contig2_All 206 1585 1,3-beta-glucanosyltransferase Gel1 [Penicillium digitatum PHI26] >gi|425783216|gb|EKV21075.1| 1,3-beta-glucanosyltransferase Gel1 [Penicillium digitatum Pd1]

ATGAAGTCCTCTGTAGCCTTGGCCACGGCCATCACCCTGGGCGCCTCAACCGTCCTTGGT

GCCGCAACCAACATGAAGGCCCGCGACAGCAGTATCACCCCCATCACTGTCAAGGGTAAC

GCCTTCTTCAAGGGCACGGACCGTTTCTACATCCGCGGTGTCGACTACCAGCCCGGTGGT

TCTTCCAACCTCACCGATCCGATCGCTGATGCCGATGGTTGCAAGCGTGACATCAAGAAC

TTCCAGAACTTGGGCCTGAACACTATCCGTGTCTACTCCGTCGATAACTCCCAGGACCAC

GACGAGTGTATGTCTGCCCTTGCTGATGCCGGCATCTACCTCGTCCTCGATGTCAACACT

CCCAAATACTCCATCAACCGTGCCGATCCCGAGATTTCCTACAACGATGTCTACCTCCAG

AACATCTTCGCCACCCTAGAGATGTTCGCCAAGTACGACAACACCCTGGCTTTCTTCTCC

GGTAATGAGGTCATCAACGACGGTCCCTCTTCCAAGGCCGCTCCGTACGTCAAGGCTGTC

ACCCGTGATATGCGGACCTACCTCCGCTCTCGCAAGCTCCGCCAGGTCCCCGTCGGATAC

TCCGCCGCTGATGTTGACACCAACCGCCTCGAGATGGCCGAGTATATGAACTGCGGTACC

GATGACGAGCGCAGTGATTTCTTCGCCTTCAACGACTACTCCTGGTGCGATCCCTCTTCC

TTCACCATCTCCGGTTGGGACCAGAAGGTGAAGAACTTCACCGGCTACGGACTCCCCCTT

TTCCTCTCCGAGTACGGCTGCAACATCAACACCCGCAAGTTCGAGGAGGTCAAGGCCCTC

TACTCCACTGAGATGACTTCCGTCTACTCTGGTGGTCTTGTCTACGAGTACACCGAGACA

GGCAACAACTATGGTCTGGTGGAGATCAGCGGTAACACGGTCACCACCAATAAGGATTAC

GATGCTCTCAAGACCGCCTTCGAGGGCACCAAGAACCCCACTGGTAACGGCGGCTACAAC

TCCACTGGCGGTGCCTCCGGCTGCCCCAAGATGCAGAAGCCCAACTGGGATGTCGACTCC

GACTCTCTCCCCGCTATGCCCGCCCCCGCCAAGAAGTACCTGACTGAGGGTGCCGGCAAG

GGCGTTGGCTTTGCCGGCAAGGGCAGCCAGAACTCCGGCACTGCGTCCTCCGGCACTGCC

ACCCAGGGCTCTGGTGAGGTCGCTGCCGCCACCGGGACCAGCACTACTACTTCCAGCTCC

AGCTCCAGCTCCACCGGTGCTGCCGCTGGTCTCAAGCCTTTCCAGTTCAGCTTCGCTCCT

GCTACCGTCGGCCTGGTGACTGTCCTGTCGACTGTCCTCGGCGCTAGCTTCATGCTTCTG

>CL87.Contig1_All 90 257 Pc22g01560 [Penicillium chrysogenum Wisconsin 54-1255] >gi|211591217|emb|CAP97444.1| Pc22g01560 [Penicillium chrysogenum Wisconsin 54-1255]

ATGACTCACGAGAGCGTTTGGTACAGCCGGCCCCGCAAGTACGGCAAGGGCTCCCGCCAA

TGCCGTGTCTGCTCCCACCGCGCCGGTCTGATCCGCAAGTACGGCATGGATATCTGCCGT

CAGTGCTTCCGTGAGAAGTCCGCCGACATCGGTTTCACCAAGTACCGC

>CL87.Contig2_All 90 257 Pc22g01560 [Penicillium chrysogenum Wisconsin 54-1255] >gi|211591217|emb|CAP97444.1| Pc22g01560 [Penicillium chrysogenum Wisconsin 54-1255]

ATGACTCACGAGAGCGTTTGGTACAGCCGGCCCCGCAAGTACGGCAAGGGCTCCCGCCAA

TGCCGTGTCTGCTCCCACCGCGCCGGTCTGATCCGCAAGTACGGCATGGATATCTGCCGT

CAGTGCTTCCGTGAGAAGTCCGCCGACATCGGTTTCACCAAGTACCGC

>CL87.Contig3_All 92 259 Pc22g01560 [Penicillium chrysogenum Wisconsin 54-1255] >gi|211591217|emb|CAP97444.1| Pc22g01560 [Penicillium chrysogenum Wisconsin 54-1255]

ATGACTCACGAGAGCGTTTGGTACAGCCGGCCCCGCAAGTACGGCAAGGGCTCCCGCCAA

TGCCGTGTCTGCTCCCACCGCGCCGGTCTGATCCGCAAGTACGGCATGGATATCTGCCGT

CAGTGCTTCCGTGAGAAGTCCGCCGACATCGGTTTCACCAAGTACCGC

>CL87.Contig4_All 90 257 Pc22g01560 [Penicillium chrysogenum Wisconsin 54-1255] >gi|211591217|emb|CAP97444.1| Pc22g01560 [Penicillium chrysogenum Wisconsin 54-1255]

ATGACTCACGAGAGCGTTTGGTACAGCCGGCCCCGCAAGTACGGCAAGGGCTCCCGCCAA

TGCCGTGTCTGCTCCCACCGCGCCGGTCTGATCCGCAAGTACGGCATGGATATCTGCCGT

CAGTGCTTCCGTGAGAAGTCCGCCGACATCGGTTTCACCAAGTACCGC

>CL88.Contig1_All 686 889 minus strand hypothetical protein ANI_1_2028104 [Aspergillus niger CBS 513.88]

AGTTGTTTTGAAAGGGCTAAGATAGAAAATATTGAGATGGAGGCGCTTGTTGCGTTCGGG

GAAGCGACCTATGATGAAAAGCATCAAAATGATATGCGGGAGGCATTTTTATGCATTCTA

AATACCTGTCCGTTTAGACGATCTGGAAATTCGAAGCCGCGGCTTCCCTCAAATTCGACA

CGAAGGCGCCTCCGGAAAAAGGCC

>CL88.Contig2_All 193 921 minus strand hypothetical protein ANI_1_2028104 [Aspergillus niger CBS 513.88]

ATGGACGCGGACATTTGGTTCCAGGAATGGGCGTCAAAAAAGGGCCAGAAGCTATCGAAT

ACACAAATAGAACGGCTTGTCACTCTCACAAAACATTGGAAAAACACTGTTACATCTGAT

ACTTTGGGCGTTTGGACCTCAGACCCTTCGGCTGAATTCTGGGGGTTTGAACCTTACCGT

ACAGAGGGCGCGTGGGTACGGGAATGTCTGGAGATCAAAGTCCCAACATTCGTGGATTGT

GATGAAAACACTGACGACTATGAGAAAGTCTCTGCACGCCGCAGGGTCCTTCTTATTATT

CTTCATGTCATCATTCAAAGGGAAATACTGCGATTACGAGCATCTTTCCAGGCCCAGTCA

GTCAAGTTTCTTACCGCAGCTGTCAGAAATATTGTGGCACAAGCTTATCCTGGAAAGGAT

ATTAAGAAGTTATGTAATAAATGTATCCATCTTCAAAGGTATGGGCAAAGGTATTTGTCA

CTGGCGAGAAAAGAGTTGGTATTAACCCCCCTGCAAGCTACTTCATCTAATTTTGAAAGG

GCTAAGATAGAAAATATTGAGATGGAGGCGCTTGTTGCGTTCGGGGAAGCGACCTATGAT

GAAAAGCATCAAAATGATATGCGGGAGGCATTTTTATGCATTCTAAATACCTGTCCGTTT

AGACGATCTGGAAATTCGAAGCCGCGGCTTCCCTCAAATTCGACACGAAGGCGCCTCCGG

AAAAAGGCC

>CL88.Contig3_All 525 860 minus strand similar to An12g06820 [Aspergillus kawachii IFO 4308]

ATTACGAGCATCTTTCCAGGCCCAGATATTAAGAAGTTATGTAATAAATGTATCCATCTT

CAAAGGTATGGGCAAAGGTATTTGTCACTGGCGAGAAAAGAGTTGGTATTAACCCCCCTG

CAAGCTACTTCATCTAATTTTGAAAGGGCTAAGATAGAAAATATTGAGATGGAGGCGCTT

GTTGCGTTCGGGGAAGCGACCTATGATGAAAAGCATCAAAATGATATGCGGGAGGCATTT

TTATGCATTCTAAATACCTGTCCGTTTAGACGATCTGGAAATTCGAAGCCGCGGCTTCCC

TCAAATTCGACACGAAGGCGCCTCCGGAAAAAGGCC

>CL88.Contig4_All 97 828 minus strand hypothetical protein An12g06820 [Aspergillus niger]

TTACTTGCGCCCTGGATGGATGGCTTATTATCTTTGTTTGATAGAATGCTCAGTTATAGT

AACATTTCAGTCCGATTCAGCGCCGTTGTTTTCGAGATGGACGCGGACATTTGGTTCCAG

GAATGGGCGTCAAAAAAGGGCCAGAAGCTATCGAATACACAAATAGAACGGCTTGTCACT

CTCACAAAACATTGGAAAAACACTGTTACATCTGATACTTTGGGCGTTTGGACCTCAGAC

CCTTCGGCTGAATTCTGGGGGTTTGAACCTTACCGTACAGAGGGCGCGTGGGTACGGGAA

TGTCTGGAGATCAAAGTCCCAACATTCGTGGATTGTGATGAAAACACTGACGACTATGAG

AAAGTCTCTGCACGCCGCAGGGTCCTTCTTATTATTCTTCATGTCATCATTCAAAGGGAA

ATACTGCGATTACGAGCATCTTTCCAGGCCCAGATATTAAGAAGTTATGTAATAAATGTA

TCCATCTTCAAAGGTATGGGCAAAGGTATTTGTCACTGGCGAGAAAAGAGTTGTTTTGAA

AGGGCTAAGATAGAAAATATTGAGATGGAGGCGCTTGTTGCGTTCGGGGAAGCGACCTAT

GATGAAAAGCATCAAAATGATATGCGGGAGGCATTTTTATGCATTCTAAATACCTGTCCG

TTTAGACGATCTGGAAATTCGAAGCCGCGGCTTCCCTCAAATTCGACACGAAGGCGCCTC

CGGAAAAAGGCC

>CL88.Contig5_All 110 772 minus strand hypothetical protein An12g06820 [Aspergillus niger]

GTCCGATTCAGCGCCGTTGTTTTCGAGATGGACGCGGACATTTGGTTCCAGGAATGGGCG

TCAAAAAAGGGCCAGAAGCTATCGAATACACAAATAGAACGGCTTGTCACTCTCACAAAA

CATTGGAAAAACACTGTTACATCTGATACTTTGGGCGTTTGGACCTCAGACCCTTCGGCT

GAATTCTGGGGGTTTGAACCTTACCGTACAGAGGGCGCGTGGGTACGGGAATGTCTGGAG

ATCAAAGTCCCAACATTCGTGGATTGTGATGAAAACACTGACGACTATGAGAAAGTCTCT

GCACGCCGCAGGGTCCTTCTTATTATTCTTCATGTCATCATTCAAAGGGAAATACTGCGA

TTACGAGCATCTTTCCAGGCCCAGATATTAAGAAGTTATGTAATAAATGTATCCATCTTC

AAAGGTATGGGCAAAGGTATTTGTCACTGGCGAGAAAAGAGTTGTTTTGAAAGGGCTAAG

ATAGAAAATATTGAGATGGAGGCGCTTGTTGCGTTCGGGGAAGCGACCTATGATGAAAAG

CATCAAAATGATATGCGGGAGGCATTTTTATGCATTCTAAATACCTGTCCGTTTAGACGA

TCTGGAAATTCGAAGCCGCGGCTTCCCTCAAATTCGACACGAAGGCGCCTCCGGAAAAAG

GCC

>CL88.Contig6_All 469 804 minus strand similar to An12g06820 [Aspergillus kawachii IFO 4308]

ATTACGAGCATCTTTCCAGGCCCAGATATTAAGAAGTTATGTAATAAATGTATCCATCTT

CAAAGGTATGGGCAAAGGTATTTGTCACTGGCGAGAAAAGAGTTGGTATTAACCCCCCTG

CAAGCTACTTCATCTAATTTTGAAAGGGCTAAGATAGAAAATATTGAGATGGAGGCGCTT

GTTGCGTTCGGGGAAGCGACCTATGATGAAAAGCATCAAAATGATATGCGGGAGGCATTT

TTATGCATTCTAAATACCTGTCCGTTTAGACGATCTGGAAATTCGAAGCCGCGGCTTCCC

TCAAATTCGACACGAAGGCGCCTCCGGAAAAAGGCC

>CL88.Contig7_All 630 833 minus strand hypothetical protein ANI_1_2028104 [Aspergillus niger CBS 513.88]

AGTTGTTTTGAAAGGGCTAAGATAGAAAATATTGAGATGGAGGCGCTTGTTGCGTTCGGG

GAAGCGACCTATGATGAAAAGCATCAAAATGATATGCGGGAGGCATTTTTATGCATTCTA

AATACCTGTCCGTTTAGACGATCTGGAAATTCGAAGCCGCGGCTTCCCTCAAATTCGACA

CGAAGGCGCCTCCGGAAAAAGGCC

>CL88.Contig8_All 137 865 minus strand hypothetical protein ANI_1_2028104 [Aspergillus niger CBS 513.88]

ATGGACGCGGACATTTGGTTCCAGGAATGGGCGTCAAAAAAGGGCCAGAAGCTATCGAAT

ACACAAATAGAACGGCTTGTCACTCTCACAAAACATTGGAAAAACACTGTTACATCTGAT

ACTTTGGGCGTTTGGACCTCAGACCCTTCGGCTGAATTCTGGGGGTTTGAACCTTACCGT

ACAGAGGGCGCGTGGGTACGGGAATGTCTGGAGATCAAAGTCCCAACATTCGTGGATTGT

GATGAAAACACTGACGACTATGAGAAAGTCTCTGCACGCCGCAGGGTCCTTCTTATTATT

CTTCATGTCATCATTCAAAGGGAAATACTGCGATTACGAGCATCTTTCCAGGCCCAGTCA

GTCAAGTTTCTTACCGCAGCTGTCAGAAATATTGTGGCACAAGCTTATCCTGGAAAGGAT

ATTAAGAAGTTATGTAATAAATGTATCCATCTTCAAAGGTATGGGCAAAGGTATTTGTCA

CTGGCGAGAAAAGAGTTGGTATTAACCCCCCTGCAAGCTACTTCATCTAATTTTGAAAGG

GCTAAGATAGAAAATATTGAGATGGAGGCGCTTGTTGCGTTCGGGGAAGCGACCTATGAT

GAAAAGCATCAAAATGATATGCGGGAGGCATTTTTATGCATTCTAAATACCTGTCCGTTT

AGACGATCTGGAAATTCGAAGCCGCGGCTTCCCTCAAATTCGACACGAAGGCGCCTCCGG

AAAAAGGCC

>CL88.Contig9_All 48 251 minus strand hypothetical protein ANI_1_2028104 [Aspergillus niger CBS 513.88]

TCTAGTTTTGAAAGGGCTAAGATAGAAAATATTGAGATGGAGGCGCTTGTTGCGTTCGGG

GAAGCGACCTATGATGAAAAGCATCAAAATGATATGCGGGAGGCATTTTTATGCATTCTA

AATACCTGTCCGTTTAGACGATCTGGAAATTCGAAGCCGCGGCTTCCCTCAAATTCGACA

CGAAGGCGCCTCCGGAAAAAGGCC

>CL89.Contig1_All 5 433 Pc13g01220 [Penicillium chrysogenum Wisconsin 54-1255] >gi|211583188|emb|CAP91191.1| Pc13g01220 [Penicillium chrysogenum Wisconsin 54-1255]

CTATTTCCTGAAGCGCAGCACGATTTGTTTTGTACAAAAATCAAATCAATAGTTCAGAAC

GCGGCCGAATTTTGGCTTCCAACTCAGAGATCGCAACAGAAGTTCGAGACAGATTTCGAA

GAACCTTTTGATCCCGACGACAATGAATGGGATCGCTTTCCTTTCGCTGGTGAAAACACA

ACACCGGTGGCACAAGACCATGGCCCTTATCTTCTCAATGTCTTCCCGTGTATCTCCTGG

GTGGAAGACGGCGACCATAATCCATTGACCAAGATCATTCAACTCCGCAGTTCCCAGGAA

TTATACTTGGCAGCGCAGCATGAAGCCACTCAAATAACAACCTCTGCCACCACCAGACGG

CATTCTGCCGGCCCCAGGAGACAATCAACTGCACGCTCAAATGGGAAGCCTTTTTTAGGT

GGAAATTCA

>CL89.Contig2_All 174 1502 minus strand hypothetical protein PDIG_89630 [Penicillium digitatum PHI26] >gi|425779273|gb|EKV17345.1| hypothetical protein PDIP_31820 [Penicillium digitatum Pd1]

ATGAGCACTTCGTCTCCTCCCACTCCGCCATCCAGGGCCTTAATAGATGATGAACGGCGA

TTCAAAACCATTACATTACCTTGTGAGTGGGTGGAGGATTATCGGCCAGGTGGTTATCAT

CCAGTCGTTCTCGGCGACGTCTTCAACCACCAGTACAAGGTCATCCGGAAGCTTGGCGAG

GGCTCCTACTCAACGGTTTGGCTCGCCCATGACCTAAATAACAGTCGATATGTCGCCCTG

AAGATACTTGTGTCGGAAATCTCGGAATCGACAACTGAGCTACGAATATTACGCCATATC

ATCGAATACGCACCAGAAGAAGGGTCCCGATATATCACGCTACTGCTGGACGAATTCGAA

CACCGCGGACCCAATGGCCTCCACATGTGCCTGGTACTTGAGCCTATGGGCCCGAGTGTC

AATACGATGGTCGAGGAGCTGCCCCAGTTCAAACCTCGTAGGCGAGGAATGAAGATCCGC

TATCCGCTTCAGATGGCAAAGAGTATCCTTAAGCAATCCTTACAGGCTCTTGCATTTCTG

CATGAAAATGGTATTGCCCATGGAGACTTCCAGCCGGGAAACATACTCTTCCCTCTTATC

GATGTTGACTCGACCCCTGAGGAATTGCTACTACAAGAGGAAGATGTGCAAGCCCGGTCG

ATCTCGCCTCCGGTACAGAGGCTGGATGGTAAACAAGATAAATGGGCGCCTCAATATCTT

TGCGTTGCGCAGCCACTGGTGCCCTTTACTTGCTACGCCGAAGGGTTCAAGGTCAAATTA

TCCGATATGGGCGGTGCTTATTTCTTTACCGACCCACCAACAAAGCCCGTTACTCCACTC

GGTCTTCGAGCTCCCGAGTTGATTCTTACCGGAGCCGTCAACAATACCGTTGATATCTGG

AGCTTCGGTTGCCTTATTTTTGAGCTAATTACCGGACAGCCACTCTTCTGTATACCATGT

TCCGAGATGGAAGATGACGATCATCTTCTCTCCCTCACCGCCCAGCTCGGTGCCCTCCCC

GACGAGTTATTCAGGCATTGGACAACCTCGTCGCTCTACTTTACGCCCGAGAGGAAGCTC

TTTAATTGTCAGCTTGGAGGAGTCGCTCCCGGGGAGGAACCGCTTATAGTGGAGCAGACG

TCCATGGAAGAGTGCTTCGATCGGGCAGGCCCGGATCTCGATGAGGAGGAAGCTAAGAAA

GTGAAAGCGCTTATTCGATGGATTTTGCAATATGACCCCGGGAAGAGACCTTCGCCTACG

AGAATCTTATCAGATCCATGGTTTTGCGAGATTGACGTTGAGGACGACCCATCTAAGATA

TCTATAGTC

>CL91.Contig1_All 435 3038 hypothetical protein PDIG_26990 [Penicillium digitatum PHI26]

ATGCCCTCTCAGTCCCATCCACTGTCTCAACCTCCATCTCAACCTCGTTCCCGCTCTCGC

TCCCCCTCTCCCTCTCAACCCTCAAGCCCCTCCGATACAACCACGGCGCTTCCGGTTCCT

ATTTCGCCTAAGGAATCAAAACCAGAATTAAAACCAGATCCAAAGCAGGATCTGAAACCA

TTTCATCGTCGCCTATTGCAGTTTGCGCAACGTCAAAAGAAGGAATCATCGCCCCGGTCA

AAGCAAGAAGACCAACAGCGCCAAATCGAAGCACTAACACGGGGAGGTTATCTTATTCCA

GCCGCTTTGCCTTTGTCCAAGGAGGCAAAGGGACATAAGGAGGTAAAGCAATCGAAAGAA

AGCCCGTCACTTTCTCTCTCTCGATCTCTTTCTAAATCGAAGAAAGATGTGGAAAGCATT

GGACAGCCGTGGCTGCAAGCCAAAGTCGATGGAAGCACGCGACCAACCGGTACAGGGCGT

CACTTGGGCTCTCTGGATCTTGGTGACTTTGACTCAATGGTCGAAGTCGCAGTCTCACTC

ACTTCCGATTTTGACGATTCTGTGCCGCCGCCATACCAGCCAAACGATCACACCCCATCC

CAATCCTCATCAGCTAAACCGACATCAAGTTCTATGGTAGACTTCAGACCTCGAAGTAGT

ACCCGATTAACGTCCTCGTTGGCTTCTACCAGTGATTCCTATAGGAACGCGTCAATTGAT

GAGCAAAGCCAGTGGCTATCTAACTCTTTAGAGTCGAGCAGTCGCGCCGATCCTGTCTCA

CGAATCACTATCAATGCCTCCGTGGGCATGAATGGGTTAGAGTCACAGCCTAAACCGCCC

ATTGATAGCCAAAAGCAAACTCAGTCAGAACAGTCTTCCCTTCGTACTGTCACCCCCTTG

TCGCCTACCTTGAAACTTTTCCCTGATGTTGCAACTCCTCGCAAATTTAGCCAAGGCTCA

TGGAGAAATTCATCAATTCCTCGGTACCAAACGATCGAAAGTTCCGCAGCATCGCCGGCA

TCTCTGGCATCTAGCTCAGGACCTGGACCTTCTGATGTTCCAGTCAGCAAGGCAGAGCCG

AAGTCCTTCGATACGCTTGCATCTTGTGTGAGCTCTTCTGCAGCGGCACAATCTAAGGAA

GAAGCCACAAAGGATTCCGCCTCGAAAGATGAGACCAAAATTCGACCGCTCTTCTTGTCT

CTGAGCACATCCAAGGCATTTCCTCTCCCAGCGCCTACAAGGCCCCTGCCATCACTGCCC

GAAACGAAATGCTCGCCGAGTACTCCAGACACCAACGCTCGCTCTATTCCAGCGATTGCT

TCGGAGCCATTATCTTCCAAACTGCATTTTAATTCAGCAACGCTGACTGAAGACCAGGAG

ACTGAATCCGCCGTTAACAGTCCTTGTGCCCTTAACTCGCGCCCGACAGTCGCTTTTGAC

GGTATCGACGCTGGAGCGTCATTGGCCGATGCAATAGTCGATGACGAGGAAAGCTTGTTT

AACACTTCGCTTTCGGAACACTACCAGCCAACCCCGGAGCATTACACTCCTAGAGAACGT

GTTTCAAGCGTGCGCATCCCGCGACTGCAAGAATTTCCCGAAAGCCTATCAGGTCAACGT

GATGCGAGGGTCTCAGAGGGGCAGCCTTTGGCCGACTCTCCTGTGCTAGGACACTCTATC

CCTACACAATCTAATGGCAGACGTGCTGTTATGAAGGGGCTTCAGATCAATTCTCAAATC

ATCCGAAACAACCTTCCATTTGGTCTGCCGTCGCCTCCACCCACTGCATCACTCCCATCA

GCCCCACCTCCTCAGCACCCACCTCCTCCGCCTCCTGGACTAAGGGTTGGCCAACGCAAC

TACACTGCGCCCAATGTGGCTATTCTGCCATCGATGAGGAACATGGAAACGGAGCCCTAC

CGAAGTTTGACCTTATCCCAGAAGGATAGCTCGGGGAGCAGCCTTCGACATGAGAGTGTC

CCAGATCCAAACCAAAGTCGCTCCGAATCTCACCCAGAGTCTCTGCTTCCATCCTCGGAT

GATGAAATCTTTGGTCCAGAGAAAGACACCAAAGTTTCGCGTCAGGAGGCCGACAAAAAC

CACCGCCTTCAGCCAACACATCGAGGACATGAGACAAGAGATCCACGGCAGATGCCCTCT

CGCAATCGACTTCGTTACCCTAATCCAGCGCGCCCTATGACACCCCAAAGCCGTCACCTC

CACAGTTTTGAGAAGACTTCGTCTCCTCAGTCACAATATTCCCAATCAACCCACAGATCG

AGGGGGTCTCAAAGCAGTCAACACACCCGCGCTGCCCCTCAACGTCATCACTACCTCGAA

GACCGAGTCGCGAATCTAGAGCGCCAGAACCAGATTCTACAGGCCGCTCTTATGGCGGCG

CTCAACGTTGAAGTCAAGAATCCTCTCATGGATCTCAATCTCGACCCGAACCTGTCTTTG

GGCCCATCACACGCTCCCTTTGTGCACCAATACCCCAGCCGCCACACTTCACGATCTGAT

AGCTGGGTTAGTTCATCTCGCAGCAGTGTCCACAGCGGGGTTGAGACTTCTAGCTCCTAT

CAAGATGGCCGCCCAAATGTCAAG

>CL91.Contig2_All 435 3095 minus strand hypothetical protein PDIG_26990 [Penicillium digitatum PHI26]

ATGCCCTCTCAGTCCCATCCACTGTCTCAACCTCCATCTCAACCTCGTTCCCGCTCTCGC

TCCCCCTCTCCCTCTCAACCCTCAAGCCCCTCCGATACAACCACGGCGCTTCCGGTTCCT

ATTTCGCCTAAGGAATCAAAACCAGAATTAAAACCAGATCCAAAGCAGGATCTGAAACCA

TTTCATCGTCGCCTATTGCAGTTTGCGCAACGTCAAAAGAAGGAATCATCGCCCCGGTCA

AAGCAAGAAGACCAACAGCGCCAAATCGAAGCACTAACACGGGGAGGTTATCTTATTCCA

GCCGCTTTGCCTTTGTCCAAGGAGGCAAAGGGACATAAGGAGGTAAAGCAATCGAAAGAA

AGCCCGTCACTTTCTCTCTCTCGATCTCTTTCTAAATCGAAGAAAGATGTGGAAAGCATT

GGACAGCCGTGGCTGCAAGCCAAAGTCGATGGAAGCACGCGACCAACCGGTACAGGGCGT

CACTTGGGCTCTCTGGATCTTGGTGACTTTGACTCAATGGTCGAAGTCGCAGTCTCACTC

ACTTCCGATTTTGACGATTCTGTGCCGCCGCCATACCAGCCAAACGATCACACCCCATCC

CAATCCTCATCAGCTAAACCGACATCAAGTTCTATGGTAGACTTCAGACCTCGAAGTAGT

ACCCGATTAACGTCCTCGTTGGCTTCTACCAGTGATTCCTATAGGAACGCGTCAATTGAT

GAGCAAAGCCAGTGGCTATCTAACTCTTTAGAGTCGAGCAGTCGCGCCGATCCTGTCTCA

CGAATCACTATCAATGCCTCCGTGGGCATGAATGGGTTAGAGTCACAGCCTAAACCGCCC

ATTGATAGCCAAAAGCAAACTCAGTCAGAACAGTCTTCCCTTCGTACTGTCACCCCCTTG

TCGCCTACCTTGAAACTTTTCCCTGATGTTGCAACTCCTCGCAAATTTAGCCAAGGCTCA

TGGAGAAATTCATCAATTCCTCGGTACCAAACGATCGAAAGTTCCGCAGCATCGCCGGCA

TCTCTGGCATCTAGCTCAGGACCTGGACCTTCTGGTAACGGACGCAAAGTTCCAGATGAG

CCAAAAAGATTGTCATCTGATGGTCTCATAGATGTTCCAGTCAGCAAGGCAGAGCCGAAG

TCCTTCGATACGCTTGCATCTTGTGTGAGCTCTTCTGCAGCGGCACAATCTAAGGAAGAA

GCCACAAAGGATTCCGCCTCGAAAGATGAGACCAAAATTCGACCGCTCTTCTTGTCTCTG

AGCACATCCAAGGCATTTCCTCTCCCAGCGCCTACAAGGCCCCTGCCATCACTGCCCGAA

ACGAAATGCTCGCCGAGTACTCCAGACACCAACGCTCGCTCTATTCCAGCGATTGCTTCG

GAGCCATTATCTTCCAAACTGCATTTTAATTCAGCAACGCTGACTGAAGACCAGGAGACT

GAATCCGCCGTTAACAGTCCTTGTGCCCTTAACTCGCGCCCGACAGTCGCTTTTGACGGT

ATCGACGCTGGAGCGTCATTGGCCGATGCAATAGTCGATGACGAGGAAAGCTTGTTTAAC

ACTTCGCTTTCGGAACACTACCAGCCAACCCCGGAGCATTACACTCCTAGAGAACGTGTT

TCAAGCGTGCGCATCCCGCGACTGCAAGAATTTCCCGAAAGCCTATCAGGTCAACGTGAT

GCGAGGGTCTCAGAGGGGCAGCCTTTGGCCGACTCTCCTGTGCTAGGACACTCTATCCCT

ACACAATCTAATGGCAGACGTGCTGTTATGAAGGGGCTTCAGATCAATTCTCAAATCATC

CGAAACAACCTTCCATTTGGTCTGCCGTCGCCTCCACCCACTGCATCACTCCCATCAGCC

CCACCTCCTCAGCACCCACCTCCTCCGCCTCCTGGACTAAGGGTTGGCCAACGCAACTAC

ACTGCGCCCAATGTGGCTATTCTGCCATCGATGAGGAACATGGAAACGGAGCCCTACCGA

AGTTTGACCTTATCCCAGAAGGATAGCTCGGGGAGCAGCCTTCGACATGAGAGTGTCCCA

GATCCAAACCAAAGTCGCTCCGAATCTCACCCAGAGTCTCTGCTTCCATCCTCGGATGAT

GAAATCTTTGGTCCAGAGAAAGACACCAAAGTTTCGCGTCAGGAGGCCGACAAAAACCAC

CGCCTTCAGCCAACACATCGAGGACATGAGACAAGAGATCCACGGCAGATGCCCTCTCGC

AATCGACTTCGTTACCCTAATCCAGCGCGCCCTATGACACCCCAAAGCCGTCACCTCCAC

AGTTTTGAGAAGACTTCGTCTCCTCAGTCACAATATTCCCAATCAACCCACAGATCGAGG

GGGTCTCAAAGCAGTCAACACACCCGCGCTGCCCCTCAACGTCATCACTACCTCGAAGAC

CGAGTCGCGAATCTAGAGCGCCAGAACCAGATTCTACAGGCCGCTCTTATGGCGGCGCTC

AACGTTGAAGTCAAGAATCCTCTCATGGATCTCAATCTCGACCCGAACCTGTCTTTGGGC

CCATCACACGCTCCCTTTGTGCACCAATACCCCAGCCGCCACACTTCACGATCTGATAGC

TGGGTTAGTTCATCTCGCAGCAGTGTCCACAGCGGGGTTGAGACTTCTAGCTCCTATCAA

GATGGCCGCCCAAATGTCAAG

>CL92.Contig1_All 579 1667 minus strand hypothetical protein PDIP_52020 [Penicillium digitatum Pd1] >gi|425776307|gb|EKV14529.1| hypothetical protein PDIG_32430 [Penicillium digitatum PHI26]

ATGTCTTTCTCCACACTGGTGGCAACCTCCGCTGCGGAGTTCAACTTCTGGGATTACATC

CACGAGAGGCCGGCTCTACAAGGTGGTGTGTTATATTTTGGAATCCCACTAATGCTTGTC

TTCATCAATGCCATAAGAATTGAGATCTATGGTTGGTTGGAAGTGGCAACAGGCAGCCTC

AAAATACTGTTCCTTGGCTTTATCGTCGTGACACTGATTGCAATTAACCGTGGGGCTGGC

CCAGGTACTCGGCCCTTGGGTGATAAGTATTGGGCTTCGGCGAAAGAATTTGATAAACGG

GCGGCAAATAACTGGAGCACTGCTCTTCTAATGAGCATTTCGATAGCAACCTTTGCCTAC

ACTGGAGTCGAAGTTTTTGCTGTGTCTGCTCTGGAAGCAAAATGGACTTATCGTGCAGAC

GAAACTTCAACCATCTCCGACGTACTTGAACGGCCCAACGACGCTCAAATCGGGAAATCA

TTCCAGTTTTCCGCTAGATTCATTCCTTTGCTTGCAATGGTTGGCTATACCATGAGTGGA

TTGGTTGCAACATTTGATATAGAGCGGGATAATTGCGCTCTCCCTCGGCTCAGTTGGCTG

TCATTGGATGCGGAAGCCGGGTGCAAAAAACCTTCAAACAGTGCGGCATTCGTTTCAATT

GCTGCAATGTCCAACATTCCGCACATGGCAGACGTGTTCAATGCTTTGCTCGTGTTTACG

TGTCTCTCTTGTGCTGCAACGAATCTGTATGTCGCCTCTCGCACCTTGTTTGGTCTGACA

AGTCGTCTGGATGGAGGCGAAGGCCAACGATGGTATCTGCGTATCTTTGCGTGGTTTGGC

AAAACGGATGGACGACAGGTTCCCCTGCGAGCTATGGTATTCTCCGCAGTTGCATTCTGG

TGGGTTCCATTCCTGCAGCTGATCCGCGGCAATAAAAGCACCCAATCCTCGGAGAATTCC

ACTAAACATAATGTGAAGGAAACGCGGGCTTCCGTGGATATGTTTGTGGAGGTATTGAGC

CAAATGGCCTACTCTGCGGTTTTAATCGTCTGGGCATGCGAGTGTCTGGCGTTCATTCGC

TTCTATCAT

>CL92.Contig2_All 57 329 minus strand Pc22g16190 [Penicillium chrysogenum Wisconsin 54-1255] >gi|211592552|emb|CAP98907.1| Pc22g16190 [Penicillium chrysogenum Wisconsin 54-1255]

ATGTCGCAGCCGGTCGAGTCACAGTGGATGGAGATGCCAGAATGGGCACGGTCCTGGCTC

TCTGGCATTTTGCCCACAGGGGAGATCGAATCTCAGAATTCTTCTCTTCATCCCCAAAAT

GACTCTCCGAGCAGAACAAGATCACCCTCATGTGTTTTAATGAATCCAGAAGACATCGAA

ATCCTTGCAAAGTGCCCTGAGCATCGGATTATTGTCAGGCCTAGCCGGCAACGAACTGTC

AGTAGAGCTCTGAGAGGCGTTCATTTATTTGTA

>CL92.Contig3_All 11 271 minus strand proline-specific permease, putative [Talaromyces stipitatus ATCC 10500] >gi|218721301|gb|EED20720.1| proline-specific permease, putative [Talaromyces stipitatus ATCC 10500]

AGTGCGGCATTCGTTTCAATTGCTGCAATGTCCAACATTCCGCACATGGCAGACGTGTTC

AATGCTTTGCTCGTGTTTACGTGTCTCTCTTGTGCTGCAACGAATCTGTATGTCGCCTCT

CGCACCTTGTTTGGTCTGACAAGTCGTCTGGATGGAGGCGAAGGCCAACGATGGTATCTG

CGTATCTTTGCGTGGTTTGGCAAAACGGATGGACGACAGGTTCCCCTGCGAGCTATGGTA

TTCTCCGCAGTTGCATTCTGG

>CL93.Contig1_All 225 1073 Calcineurin binding protein, putative [Penicillium digitatum PHI26] >gi|425778396|gb|EKV16524.1| Calcineurin binding protein, putative [Penicillium digitatum Pd1]

ATGGCCACTTCAGCTTCACCTCCACGCTCGCTGGCTTCGTCACCCAAACTCACTGGCTCA

CTTCACACACCGCGACCATCTCTCTCTCTCGATATCGCCAACATGCCGGCACTCTCCCAA

CCATCACTACCATCCAACACCCTCCTAATCACCGATCTCCACGATCTCCTCGTGTTCCAA

CCCCCAGCACTGGAAGAAATCCGCAATAAAATCACTGCAGTGGCACCTCTCAACTCTTTC

TCCCCACTCCCCTCAATGCGCCGCATCGTGTGCTCCTTCCACAACGAAGCCGATGCCACA

GCAGTGCGCCAACTCCTGGACGGCCAGCGCCTCTTAAACCGAGACGTGCACCCCCGTATC

TACTTCGGCGAGCCAACCGCAATCCTAGACGGCGGCCGGCCAAAACTCCTGGAAGCCCCG

CAAGTCTCCAAGATGTTCTTTATCTCGCCGCCGCCCAGCCCCCCGCACGGATGGGTCGTG

CGCAACGAGGGCCCGCCCAACAAGGAAGTCCACGCTATGGATTTGGCACTTGCCCTATCC

ATGCTGAAGACGGATCAGATGCAGTCGGAGACGTCTGCAGCCGACCCCGCAACCCCGGTC

TCTACCTCTTCTCACAAGCGTATGTCCAGCTGGCCGCTTGCTGGCTCCCAGCAGCGTAGC

AGGAGCAGTACCATCATCTACCACCCCGAAGACCATGGCAGTAGCCCCAATCTTCCGGCG

GTGACGGTTGAGGATATGACGATGGATGGCGAGGATGAGGATGTCGACATGAATGCGATG

AGCCCTATTGAGCTGTCTGTTAACCAAATGCCGCCCAAGACTTCGCGTCCGCCGATTGAG

TTGATGGAG

>CL93.Contig2_All 153 665 minus strand Pc12g07360 [Penicillium chrysogenum Wisconsin 54-1255] >gi|211582190|emb|CAP80363.1| Pc12g07360 [Penicillium chrysogenum Wisconsin 54-1255]

ATGGCCATCTCAGCTTCATCTCCACACTCACTGGCTTCATCACCAACATTAACTCGCTCA

TCCCACACCTCACGACCATCTCTAACTCTCGATATCGCCAACATGCCCGCAGTCTCTCAG

CCAACACCCCCCTCCAACACCCTCCTAATCACCAATCTCCACGATCTCCTCGTCTTCCAA

CCCGCAGCACTGGAAGAGATCCGCCACCAAATCACCGCCGTGGCCCCCCTAAACTCATTC

TCCCCACTTCCCTCCATGCGCCGCATCGTGTGCTCGTTCCACAACGACACAGACGCCACA

GCAGTGCGCCAACTCCTAGACGGCAAGCGTCTCCTAAACCGAGACGTCCACCCCCGGATC

TACTTCGGCGAGCCGACCGCACTCCTCGACGGCGGCCGTCCCAAACTCCTCGAAGCACCA

CAAGTCTCGAAGATGTTCTTTATCTCACCGCCGCCCAGCCCGCCACACGGATGGGTCATG

CGCAACGAGGACCCGCCCAACAAGGAAGTCCAC

>CL94.Contig1_All 119 1171 hypothetical protein PDIP_38620 [Penicillium digitatum Pd1] >gi|425779824|gb|EKV17852.1| hypothetical protein PDIG_12400 [Penicillium digitatum PHI26]

ATGCCAAAATCACCACAAATTGCAGTGGATACGGATATCTACCTGTCAACCGCCTATGGC

GATGATTGGCAGTCAGAGACGACGTCTATCGGTTCCTCAATTTATAAGGGCTTGATGGAA

AATGGAAGACGGTATCAAACTCTGAGCGACAAAGAATATCTGGTTCCGTCCGATGACCAG

GCATTCGAGTCCTACGAAGTCGGTCATCTGCTCGCCCTCGTTCTAGATTCGGAACGAGAG

AACCCGCTCTTCAGGGCACCCATCGGGAGGAGCCCCAAACATATCCTCGACATTGGAACG

GGTAAGGGTAATTGGGCTATTGATGTTGCCGACATGTTCCCATCCGCAACGGTCCGAGGA

GTCGACCTCTTCCCACCTCCCGTTTCATGGATGCCGCCCAATTGTATCCTCGAAGTCGAC

AACGTCCTAGAAGAATGGACCTGGAAAAACCCCCTCGACCTGATCCACATGCGGATTCTG

GATGGCTCATTCGACTCCGCGGGATGGGATCATGTCTACACCAGTTCCTTGAAACATCTA

CGGCCAGGAGGCTGGATAGAACAACTTGAAGGTAGTACATCCATCGAATGTGACGACGAT

AGCCTGCCAGCAGACAGCATCCTGCGAACCTGGGGCCCAACTATGAACGCATGCGGTGCG

CGCGGTGGACTCAAGCTCAATACCCTTGATGGCATGCGCGGCATGATGGAGAAGGCTGGG

TTTGTGGATATCCACGAGAAGGCATACCAGTGGCCTATTGGCCCGTGGGCTCGAGATCAG

AAATATAAAGAGGCGGGCGTTGTCAATTTCCAACATTGGCTTTCGGGGATGGAGGGATGG

TGCATGTGGTTGCTGACCCACTTCGGTGCGCCGCATCCTTGGTCAAAGGATGAGGTTACT

GTTTATCTGGCAAAAATCCGATCGGAACTTAAGAATCCAAGATATCATATTTATCATAGA

GCACGACGTGTGTGGGCGCGCAAGCCCTTTCCCGACGAGCGTACGCCTGAGATGACCCCG

ATCAAGGATGAGGAAAAGTTCAAAGAGGAGCGG

>CL94.Contig2_All 280 402 hypothetical protein PDIP_38620 [Penicillium digitatum Pd1] >gi|425779824|gb|EKV17852.1| hypothetical protein PDIG_12400 [Penicillium digitatum PHI26]

ATGCCAAAATCACCACAAATTGCAGTGGATACGGATATCTACCTGTCAACCGCCTATGGC

GATGATTGGCAGTCGTAAATACTCTCATTTCTCCCGTGATTTTTTACATCCTTGATCTCT

AAC

>CL95.Contig1_All 1 648 Pc21g08580 [Penicillium chrysogenum Wisconsin 54-1255] >gi|211589608|emb|CAP95755.1| Pc21g08580 [Penicillium chrysogenum Wisconsin 54-1255]

GGTGGATCGGCGGATCCTGTACCCAAGAAGCGGAAGCGGGATCAGGCTATCTCGGTCGCT

GTGCGGCTTGTCTCAGTTTGTAATGTTTCGGGGGCTGGGGTCAGGCTTTTGTCTTCTGTC

TTGCTTGAGAGGGGATTCTTGGTGCCTAATGGGCGGGAACTTGGGGATTCGCTGAATGAT

ACATTCAAGGAATGGAACCCGGTTCTGCAAAGGGTTGCAGAGAGTCAACCTGCGTTTTTG

AGGCATTTGACGGAAGATCTGGTGAATGATCTAGCCTTTAAGAACACAACCGGCATTTCG

ACAGATGTGTCTTCCGAGGCACTGTATTTGTGGATCGCGCATATTTTGACATCTACTGCC

TGGGAGTTCCACAGACAATCATGCCCGCAAAGCTACGTTCTTCGAGCCTGCGATGAAAGT

CCCCACCACTGGACAGAGATGTTGGGAGATCAATTAAGGAAGCACGCTAGCAAGCCCAAA

TCAGTTCTTGGCGCTCGTCCCGCTGCGAAGAATCGAGTCTCGAAACCTAAACACGTCAGG

AATGACTCGCTATACGCTCCATCTCAGCTCTCAGATAAGCTGCTGCAGCATGGATGGGGA

TTCCTGGAGAAATGGGACAGTCGACCTTTGGGCGTGGTATCAAGTAAT

>CL95.Contig2_All 57 545 Pc21g08580 [Penicillium chrysogenum Wisconsin 54-1255] >gi|211589608|emb|CAP95755.1| Pc21g08580 [Penicillium chrysogenum Wisconsin 54-1255]

CTTGGGGATTCGCTGAATGATACATTCAAGGAATGGAACCCGGTTCTGCAAAGGGTTGCA

GAGAGTCAACCTGCGTTTTTGAGGCATTTGACGGAAGATCTGGTGAATGATCTAGCCTTT

AAGAACACAACCGGCATTTCGACAGATGTGTCTTCCGAGGCACTGTATTTGTGGATCGCG

CATATTTTGACATCTACTGCCTGGGAGTTCCACAGACAATCATGCCCGCAAAGCTACGTT

CTTCGAGCCTGCGATGAAAGTCCCCACCACTGGACAGAGATGTTGGGAGATCAATTAAGG

AAGCACGCTAGCAAGCCCAAATCAGTTCTTGGCGCTCGTCCCGCTGCGAAGAATCGAGTC

TCGAAACCTAAACACGTCAGGAATGACTCGCTATACGCTCCATCTCAGCTCTCAGATAAG

CTGCTGCAGCATGGATGGGGATTCCTGGAGAAATGGGACAGTCGACCTTTGGGCGTGGTA

TCAAGTAAT

>CL96.Contig1_All 2 217 general amino acid permease Gap1-Penicillium chrysogenum [Penicillium chrysogenum Wisconsin 54-1255] >gi|211589202|emb|CAP95342.1| general amino acid permease Gap1-Penicillium chrysogenum [Penicillium chrysogenum Wisconsin 54-1255]

GCTTTGCCTTCGATCATGAATGTCGTGATTATGATTGCGGTCCTGTCTGTCGGCAATTCC

TCCGTCTATGGATCCTCACGTACTCTTGCTGCCCTTGCCGAGCAGGGCCAAGCCCCCAAG

TTCCTAGCATACATTGACCGCAAGGGACGTCCTCTACCCGCCATTATCATTGCATCTATA

TTGGGTCTGCTTTCCTACCTCGCCGCCTCTGACGTG

>CL96.Contig2_All 194 1942 minus strand Amino acid permease (Gap1), putative [Penicillium digitatum Pd1] >gi|425780123|gb|EKV18141.1| Amino acid permease (Gap1), putative [Penicillium digitatum PHI26]

ATGGAGGAGAAGAAGGTCGAAGAGAGCACCGTCCAGACTGCCTTGGACGGTCCTCCAGCC

TATGGCGACTCATCTCCAACGCGGGGAGGCATGGGCCAACGGATAATCGACAGTTTCAAG

AGAGACCCCAACCAAACTGTCTCCGGCCATTCTGGAGCTGATGGCGCTGGCTTCGATCTC

GAAAATGCCGCCCACAACACTGCCAACTCCCCCCTCAAGCGTCACTTGAAGGGGCGTCAT

CTCCAGATGATTGCGATTGGTGGTTCTATTGGTACTGGTCTCTTCGTTGGTTCCGGATCT

GTTTTGGCCGCCGGCGGTCCAGCCTCAGTTTTGATTGCCTATGTTCTGATTGGCTGCATG

CTTTACTGCACAGTTCACGCCTTGGGTGAAATGGCAGTTCTCTTCCCAGTTGCCGGTTCT

TTCGCTCATTACTCGACTCGTTTCGTGGATCCTGCGTGGGGGTTTGCTATGGGCTGGAAC

TACGCTCTTCAGTGGCTCATTGTCCTACCCTTGGAGGTCGTCGCTGCTTCGATCACAGTT

GATTATTGGAGCCCAGGTGTTTCCAATGCTGCCTGGGTAACCATCTTCTGGGTTCTGATT

GTCTCAATCAACATGTTTGGTGTGCGAGGATATGGTGAGGCTGAGTTTGTGTTCTCCATC

ATCAAGGTTATTGCCGTTATTGGTTTCATCATCCTTGGGATTATCCTCAACTGCGGCGGT

GGTCCCGAAGGTGGATATATCGGCGGCAAGTACTGGCATGACCCTGGTGCATTCAGAAAC

GGTTTCAAGGGTCTCTGCAGTGTTTTCGTCAACGCTGCGTTTGCTTTTTCTGGAACCGAA

CTTGTTGGTCTCGCCGCAGCTGAAACCACAAATCCCCGCAAATCCCTTCCGACTGCCGTT

AAGCAGGTCTTCTGGCGTATTGCTCTCTTCTACGTCGTTTCCCTTGCTATCGTCGGCCTC

CTCGTAAGATACGACGACCCCAAACTCATCAGTGGTACCTCCTCCGCCGATGCCAAGGCC

TCTCCTTTCGTCATTGCCATTGAGAATGCCGGAATCAAAGTCCTGCCTTCGATCATGAAC

GTTGTCATCTTGATTGCTGTCCTGTCTGTTGGCAACTCCTCCGTCTACGGATCTTCGCGT

ACTCTTGCTGCCCTTGCCGACCAGGGTCAAGCCCCCAAGTTCCTCGCATACATCGATCGC

CAGGGTCGTCCTCTACCCGCCATTCTGGTTGCATCTGCCTTGGGTCTGCTTTGCTACCTC

GCTGCCTCAGATGTGCAGACTACTGCCTTCGCCTGGATGATGGCCATCTCGGGTCTATCG

TCTGTCTTCACCTGGGGTTCTGTGTGCGGTGCCCACATCCTCTTTCGTCGCGCGTGGAAG

GTACAGGGCCACAGCCTGGACGAGTTGGCCTTCCGTTCTCAGCCCGGCATCATTGGCTCG

TGGGTCGGTCTTATTTTCAACTGTCTCGTGCTGATTGCGCAGTTCTGGGTTGGTTTCGCC

CCCATTGGTTACGCAGACATGACCTCCCAGCAGATTGCCTACAACTTCTTCTCTGTCTAC

TTGGCCGCCCCCGTGGTTCTGGTATCCTACGTCGGTTATAAGATTGTGTATAGGACCAAG

TTTATTCTCCCCTCCGAGGCCGATTTGGCTACCGGTCGTCGCGACCTTGATGTCCAGCAT

TTGATTGAGCAGGAGCGGGCCGAGCAAAAACAATGGCCGAAGTGGAAGAAGGTTTACAAT

TTCTTCTGT

>CL97.Contig1_All 238 1530 Mitochondrial import receptor subunit (Tom37), putative [Penicillium digitatum Pd1] >gi|425771125|gb|EKV09579.1| Mitochondrial import receptor subunit (Tom37), putative [Penicillium digitatum PHI26]

ATGGTACTACAATTGCATGTATGGGGCCCAGCCTTTTCCCTGCCCTCAATTGATGCGCAA

TGCCTTGCTGCAATCGCATACTGTTCCGAGGTCCTCCCTAAAGACTCGTGGGAGCTAATC

GCGAGCAGCGATCCTTCCGTGTCCCCAACAGGTGAGCTCCCCGCTCTCCAGAATGGCTCT

ATCTGGGTAAGCCGGTTCCGCAATATCGTCGACTACCTCCGCCAATACTCCGAGGGTGCA

TGGAACCTGGATCAAAACCTAGATGATGTGCAAAAAGCCGATAGCATTGCCTTCTCCTCC

TTCATCGAATCCCGCGGTCAATCCATCCTCGATCTCTACCTCTACGTAACCAGTCAGAAC

TACTACGCCAACACATCCCCAGCCTACGGCTCTCTCCTTCAATGGCCAAATCAATGGATC

CTCCCGCCAAAGCTCCATGGCGCCGCCAAAATCCGCACCGAACACCTCGGCCTATCCTCG

CTAGACCTACAAGCAATGGAAGATCAGCGCAAACGCGAGCACTCCGCCGCCGTAGCATCC

GGCCAAATCCCTAGCAACCTGATCCAGCAGCCCCGCGAAACAGTCTCCAAACTCCTCGGT

CGCACAGTGCAAACCAACCAATTCCGCCTAGAAGCCCTAACAGCTGACTTCTTCGATCCA

CTCGAAGCAATGCTCGCCCGCAGCAAGACCTGTCTGCTCCCCGCAGACGACAACAACAAC

CCCTCCTCGCTGGACTGCGTCGCACTGGGATACCTATCACTGGCACTCGTACCCGAGCTA

GCCTTCCCCTGGCTGCGCGACGCGATGCGCGCCAAGGCCTCGCTGCTGGCGGCGTACACG

GAGCGCATGCGCAGTCGCTGTTTCGGCGAGGCGCCCGTCGACGTTAAGCATGCTTTCCAG

CCCACGGAGCCGGCTGTCCTGCCGTGGCGCGCACCGGAGCGCATCTCCGTTGCCGCGGTC

GGGAGTACACTCCTCGGCACGCTGGCTGACAACACACCGTTCTTGCGCGAGGTGCGCCAG

AATAGGCGATTGAAGCAGGCCGTTGAGGCTGATTCAGCATTTAGTTCGGTGGAGAAACAG

GTCCTGTCTTCGTATGCGGATTCTAGCAACAAGGACTTGTTGCTTTCCATTGCGACTGCT

GTGGCGGGGACAGCTGCTCTCGTTGGGTATATGGTGCATGTGGGCTTGCTTTCTTTTTCG

ACTGGGGGTGTCGAGGAAGAAGAGCAATTTGAAGAAGACGCGGGTGTTCTGCAGATTGAT

CCTGGCTCTGCTGCTGACTTTCTCGGCGCGTTC

>CL97.Contig2_All 372 1664 Mitochondrial import receptor subunit (Tom37), putative [Penicillium digitatum Pd1] >gi|425771125|gb|EKV09579.1| Mitochondrial import receptor subunit (Tom37), putative [Penicillium digitatum PHI26]

ATGGTACTACAATTGCATGTATGGGGCCCAGCCTTTTCCCTGCCCTCAATTGATGCGCAA

TGCCTTGCTGCAATCGCATACTGTTCCGAGGTCCTCCCTAAAGACTCGTGGGAGCTAATC

GCGAGCAGCGATCCTTCCGTGTCCCCAACAGGTGAGCTCCCCGCTCTCCAGAATGGCTCT

ATCTGGGTAAGCCGGTTCCGCAATATCGTCGACTACCTCCGCCAATACTCCGAGGGTGCA

TGGAACCTGGATCAAAACCTAGATGATGTGCAAAAAGCCGATAGCATTGCCTTCTCCTCC

TTCATCGAATCCCGCGGTCAATCCATCCTCGATCTCTACCTCTACGTAACCAGTCAGAAC

TACTACGCCAACACATCCCCAGCCTACGGCTCTCTCCTTCAATGGCCAAATCAATGGATC

CTCCCGCCAAAGCTCCATGGCGCCGCCAAAATCCGCACCGAACACCTCGGCCTATCCTCG

CTAGACCTACAAGCAATGGAAGATCAGCGCAAACGCGAGCACTCCGCCGCCGTAGCATCC

GGCCAAATCCCTAGCAACCTGATCCAGCAGCCCCGCGAAACAGTCTCCAAACTCCTCGGT

CGCACAGTGCAAACCAACCAATTCCGCCTAGAAGCCCTAACAGCTGACTTCTTCGATCCA

CTCGAAGCAATGCTCGCCCGCAGCAAGACCTGTCTGCTCCCCGCAGACGACAACAACAAC

CCCTCCTCGCTGGACTGCGTCGCACTGGGATACCTATCACTGGCACTCGTACCCGAGCTA

GCCTTCCCCTGGCTGCGCGACGCGATGCGCGCCAAGGCCTCGCTGCTGGCGGCGTACACG

GAGCGCATGCGCAGTCGCTGTTTCGGCGAGGCGCCCGTCGACGTTAAGCATGCTTTCCAG

CCCACGGAGCCGGCTGTCCTGCCGTGGCGCGCACCGGAGCGCATCTCCGTTGCCGCGGTC

GGGAGTACACTCCTCGGCACGCTGGCTGACAACACACCGTTCTTGCGCGAGGTGCGCCAG

AATAGGCGATTGAAGCAGGCCGTTGAGGCTGATTCAGCATTTAGTTCGGTGGAGAAACAG

GTCCTGTCTTCGTATGCGGATTCTAGCAACAAGGACTTGTTGCTTTCCATTGCGACTGCT

GTGGCGGGGACAGCTGCTCTCGTTGGGTATATGGTGCATGTGGGCTTGCTTTCTTTTTCG

ACTGGGGGTGTCGAGGAAGAAGAGCAATTTGAAGAAGACGCGGGTGTTCTGCAGATTGAT

CCTGGCTCTGCTGCTGACTTTCTCGGCGCGTTC

>CL97.Contig3_All 454 1746 Mitochondrial import receptor subunit (Tom37), putative [Penicillium digitatum Pd1] >gi|425771125|gb|EKV09579.1| Mitochondrial import receptor subunit (Tom37), putative [Penicillium digitatum PHI26]

ATGGTACTACAATTGCATGTATGGGGCCCAGCCTTTTCCCTGCCCTCAATTGATGCGCAA

TGCCTTGCTGCAATCGCATACTGTTCCGAGGTCCTCCCTAAAGACTCGTGGGAGCTAATC

GCGAGCAGCGATCCTTCCGTGTCCCCAACAGGTGAGCTCCCCGCTCTCCAGAATGGCTCT

ATCTGGGTAAGCCGGTTCCGCAATATCGTCGACTACCTCCGCCAATACTCCGAGGGTGCA

TGGAACCTGGATCAAAACCTAGATGATGTGCAAAAAGCCGATAGCATTGCCTTCTCCTCC

TTCATCGAATCCCGCGGTCAATCCATCCTCGATCTCTACCTCTACGTAACCAGTCAGAAC

TACTACGCCAACACATCCCCAGCCTACGGCTCTCTCCTTCAATGGCCAAATCAATGGATC

CTCCCGCCAAAGCTCCATGGCGCCGCCAAAATCCGCACCGAACACCTCGGCCTATCCTCG

CTAGACCTACAAGCAATGGAAGATCAGCGCAAACGCGAGCACTCCGCCGCCGTAGCATCC

GGCCAAATCCCTAGCAACCTGATCCAGCAGCCCCGCGAAACAGTCTCCAAACTCCTCGGT

CGCACAGTGCAAACCAACCAATTCCGCCTAGAAGCCCTAACAGCTGACTTCTTCGATCCA

CTCGAAGCAATGCTCGCCCGCAGCAAGACCTGTCTGCTCCCCGCAGACGACAACAACAAC

CCCTCCTCGCTGGACTGCGTCGCACTGGGATACCTATCACTGGCACTCGTACCCGAGCTA

GCCTTCCCCTGGCTGCGCGACGCGATGCGCGCCAAGGCCTCGCTGCTGGCGGCGTACACG

GAGCGCATGCGCAGTCGCTGTTTCGGCGAGGCGCCCGTCGACGTTAAGCATGCTTTCCAG

CCCACGGAGCCGGCTGTCCTGCCGTGGCGCGCACCGGAGCGCATCTCCGTTGCCGCGGTC

GGGAGTACACTCCTCGGCACGCTGGCTGACAACACACCGTTCTTGCGCGAGGTGCGCCAG

AATAGGCGATTGAAGCAGGCCGTTGAGGCTGATTCAGCATTTAGTTCGGTGGAGAAACAG

GTCCTGTCTTCGTATGCGGATTCTAGCAACAAGGACTTGTTGCTTTCCATTGCGACTGCT

GTGGCGGGGACAGCTGCTCTCGTTGGGTATATGGTGCATGTGGGCTTGCTTTCTTTTTCG

ACTGGGGGTGTCGAGGAAGAAGAGCAATTTGAAGAAGACGCGGGTGTTCTGCAGATTGAT

CCTGGCTCTGCTGCTGACTTTCTCGGCGCGTTC

>CL98.Contig1_All 216 374 hypothetical protein PDIG_36300 [Penicillium digitatum PHI26] >gi|425783748|gb|EKV21574.1| hypothetical protein PDIP_05190 [Penicillium digitatum Pd1]

ATGCCACCAACTAAAATTAACGACGAAGCCCTTGCGAAGTTGAAGGATTTGGCAACATGG

CTTTACGTTAGTGCCGAAATGAAGCAAGAAAAGAGCCTTATCAAATGTGTGGACGTGGAT

CGTTATAAGAAGACGCTCCCTGTACTAAAAGCATTGGGG

>CL98.Contig2_All 216 623 hypothetical protein PDIG_36300 [Penicillium digitatum PHI26] >gi|425783748|gb|EKV21574.1| hypothetical protein PDIP_05190 [Penicillium digitatum Pd1]

ATGCCACCAACTAAAATTAACGACGAAGCCCTTGCGAAGTTGAAGGATTTGGCAACATGG

CTTTACGTTAGTGCCGAAATGAAGCAAGAAAAGAGCCTTATCAAATGTGTGGACGTGGAT

CGTTATAAGAAGACGCTCCCTGTACTAAAAGCATTGGGGTATGACCAAATACCGGGCCAT

GGTTATCATATTTACCGCACGTCTGGGGAGACAGAAATCATTAAACGACCTGATCTGAAG

ACACTTTTCTTTCCTTTGTCTGTCGGTTCCGGGAATGACAGTGTAGCGTCAGGGGAGGTA

CAGGTGGCAGGCGAACTACTCACACCTGGCAATTTTATTGAATTGAATAGCACCTTGACT

TTCACCGCGCAGTTGGATTGTCTTATTGTCTATTTGCCGGAGGGAAAA

>CL99.Contig1_All 208 1077 Phosphoribosyl-AMP cyclohydrolase, putative [Penicillium digitatum Pd1] >gi|425771967|gb|EKV10395.1| Phosphoribosyl-AMP cyclohydrolase, putative [Penicillium digitatum PHI26]

ATGGCGACCCCCTTCCTTATCTCTCACGACCCCTCCTCAACCTCCGGCTCCGGACTTTCG

CTCAAGCAGATCGCCTACTTCGGCCGTGTACTGATCAAGGTCTCGAGTCTCACCCAGGCA

GAGCAATTCCTGCGCCAGAATTTCCGCGCCCTCGACATCTTCATCGATGCAACTGAGGTC

TCCTCTTCTGGTGATCTTGTCGATATTCTCAACGCCGGTGCCGCCAAGATCTTGATCAAC

TTGGATCAATTGACCACCCTCTCCGAAGAACAATCCGTGCCCTCATCCCGTCTGCTCGTC

CACGCCCCGTCAGATTCTCAGTTGGATCTTCTCCAGCAATGGGTTGCCGTCAATGCCGAG

CGCAGCGAAGCTAGCGTCTGCACCGCACCTTCCACCGTCCCCGCCGCCGCCAAGAAATTG

AAGATTAGCTCGGACTCCCCACGTCTCTTCACAACCTTCGGTACTCAGGCGTTGTCGGAG

GATGCCATTACACAAGTCACAAAGCAGGGTGCTATTGCTGTCGTTCCCTCACAGGCACTG

ACCGTTGAGCGAGATGTGGCTGGCCAGATCTCCGCCGCTAAGCTTATTGCATCGACCGCC

GTCACTGACCAGGCCAATGGCTTGTATGCCACTTCTGTCACTGATGAGCGGGGAGCTTGT

CTAGGCTTTGTTTGGAGCAGTGACGAGAGCATTGTCGAGGCCTTGCGCACTGGCACCGGT

GTTTACCAGAGCCGCAAGCGTGGTCTGTGGTACAAAGGCCAGTCCAGCGGGGATGTGCAG

GAATTGATCCGCATTGGCTTCGATTGTGATGCCGACTGTTTGGTCTTTGTTGTTAAGCAG

ATTGGACGTGGTAAGAACCAGCTTTCGACA

>CL99.Contig2_All 81 2690 Phosphoribosyl-AMP cyclohydrolase, putative [Penicillium digitatum Pd1] >gi|425771967|gb|EKV10395.1| Phosphoribosyl-AMP cyclohydrolase, putative [Penicillium digitatum PHI26]

ATGGCGACCCCCTTCCTTATCTCTCACGACCCCTCCTCAACCTCCGGCTCCGGACTTTCG

CTCAAGCAGATCGCCTACTTCGGCCGTGTACTGATCAAGGTCTCGAGTCTCACCCAGGCA

GAGCAATTCCTGCGCCAGAATTTCCGCGCCCTCGACATCTTCATCGATGCAACTGAGGTC

TCCTCTTCTGGTGATCTTGTCGATATTCTCAACGCCGGTGCCGCCAAGATCTTGATCAAC

TTGGATCAATTGACCACCCTCTCCGAAGAACAATCCGTGCCCTCATCCCGTCTGCTCGTC

CACGCCCCGTCAGATTCTCAGTTGGATCTTCTCCAGCAATGGGTTGCCGTCAATGCCGAG

CGCAGCGAAGCTAGCGTCTGCACCGCACCTTCCACCGTCCCCGCCGCCGCCAAGAAATTG

AAGATTAGCTCGGACTCCCCACGTCTCTTCACAACCTTCGGTACTCAGGCGTTGTCGGAG

GATGCCATTACACAAGTCACAAAGCAGGGTGCTATTGCTGTCGTTCCCTCACAGGCACTG

ACCGTTGAGCGAGATGTGGCTGGCCAGATCTCCGCCGCTAAGCTTATTGCATCGACCGCC

GTCACTGACCAGGCCAATGGCTTGTATGCCACTTCTGTCACTGATGAGCGGGGAGCTTGT

CTAGGCTTTGTTTGGAGCAGTGACGAGAGCATTGTCGAGGCCTTGCGCACTGGCACCGGT

GTTTACCAGAGCCGCAAGCGTGGTCTGTGGTACAAAGGCCAGTCCAGCGGGGATGTGCAG

GAATTGATCCGCATTGGCTTCGATTGTGATGCCGACTGTTTGGTCTTTGTTGTTAAGCAG

ATTGGACGTGGATTCTGCCACCTTGGTACGGAGACCTGCTTCGGCGCATCTTCCGGTCTG

TCTCGTCTCCAAAAGACCTTAGATGCCCGTAAGGCGGATGCTCCGGCTGGATCATACACC

GCGCGTTTGTTCAATGAGCCCAAGCTCATCGATGCTAAGATCATGGAGGAGGCTGATGAG

TTGTGTCGTGCGAACACGAAGGAGGAGATTGCTTTCGAGGCCGCCGACCTGCTTTACTTT

GCCCTGACCAAGTGCACTGCTGCCGGTGTCAGTCTGGAGGATATCGAGCGGAACCTTGAC

CTCAAGAGCTTGAAGGTGAAGCGGAGAAAGGGCGACGCTAAGGGACCTTGGGCTGAAAAG

GCTGGCCTGGCTAAGCCCGAGGCAAAGCCTGCCCCTACCCCGGCTCCCGTACCCGCCCCG

ATTGAGGATCGCACATCCCGAATTGAGATGAGGCGCGTGATCACTGCCTCTACACCCCCC

CAGGTGGTCGCTGATTACCTCAAGCGTCCTTCCCAAAAATCTAACGAAGCCATTGTCAAC

CTGGTGAAGCCAATCATCCAGGAGGTCCGTGATGGCGGCGACGCTGCTGTACTCAAATAC

ACCCACAAGTTCGAGAATGCGACATCACTTACCTCCCCTGTTATTCACGCACCATTCCCC

GCTGAACTCATGAAGCTGTCCCCCGATGTCCAAGAGGCAATTGATATCAGCATTGGCAAC

ATCGACCGCTTCCACTCCGCACAGATGGGAAGCAACGAAACCCTCCAGATGGAGACTATG

CCTGGTGTGGTCTGCTCTCGCTTCTCGCGTCCCATCGAGCGTGTCGGTTTGTACATTCCC

GGCGGTACCGCTGTGCTGCCCTCCACTGCCATGATGCTGGGTGTTCCCGCCATGGTGGCA

GGCTGCAACAAGATTGTCCTGGCCTCGCCTCCCCGCTCCGACGGCAGCATCTCCCCCGAA

ATTGTCTACGTGGCCCACAAGGTCGGCGCTGAGAGCATCGTCTTGGCCGGTGGTGCCCAG

GCCGTGGCTGCCATGGCCTACGGCACAGAAAGCATCACCAAGGTCGACAAGATCCTGGGT

CCGGGCAACCAGTTCGTGACGGCCGCAAAGATGTTCGTGTCCAACGATACCTCCGCAGGC

GTCAGCATCGACATGCCCGCCGGTCCCAGTGAAGTCCTCGTGATTGCCGACAAGACCGCC

ATCCCGGCCTTTGTTGCATCCGACCTTCTCAGCCAGGCCGAGCACGGCGTCGACTCACAG

GTTATCCTCATCGCTGTCGACCTGAACGAGGCCGAGCTGCGTGCCATCGAAGATGAGGTC

GATGCCCAGGCCAAGGCTTTACCCCGCATGGATATCGTCCGCGGCTCCCTCGCGCACTCC

GTCACCTTTGTTGTCCGCGACATCAACGAGGCCATGGCCCTCAGCAACGACTACGCGCCC

GAGCACCTAATTCTGCAGGTCGAGAACGCCGAGTCTCTGGTTAAGGATGTCCAGAATGCC

GGCAGTGTCTTCATCGGCGCCTGGACCCCCGAGAGTGTTGGTGATTACTCCGCCGGAGTC

AACCACTCGCTGCCTACCTACGGCTACGCCAAGCAGTACTCGGGTGTCAACCTCGGCTCC

TTCCTGAAGCACATCACCAGCTCGAACTTGACCGCCGATGGCTTGCTCGGTCTTGCCAAG

ACGGTTGAGACCCTCGCCGCTGTGGAGGGCCTGGAGGCGCACAAGCGTGCCATCAGCATC

CGTGTTGCCCACATGAAGAAGGACCAGTCA

>CL99.Contig3_All 78 287 Phosphoribosyl-AMP cyclohydrolase, putative [Penicillium digitatum Pd1] >gi|425771967|gb|EKV10395.1| Phosphoribosyl-AMP cyclohydrolase, putative [Penicillium digitatum PHI26]

AACTCCTTTCTAGCTACCTACGGCTACGCCAAGCAGTACTCGGGTGTCAACCTCGGCTCC

TTCCTGAAGCACATCACCAGCTCGAACTTGACCGCCGATGGCTTGCTCGGTCTTGCCAAG

ACGGTTGAGACCCTCGCCGCTGTGGAGGGCCTGGAGGCGCACAAGCGTGCCATCAGCATC

CGTGTTGCCCACATGAAGAAGGACCAGTCA

>CL100.Contig1_All 577 2844 minus strand hypothetical protein PDIP_58440 [Penicillium digitatum Pd1] >gi|425773321|gb|EKV11680.1| hypothetical protein PDIG_49060 [Penicillium digitatum PHI26]

ATGACCACCGTTTTGCCGACCCCCAACTCTCCCCCCGAGCTCTCCGGGTCCAAATCCTCT

AAATCCTCCTCGTTTCATTCCTCTTCTCACCTGGACGGTCCCGACAGCATCTTCACGGAT

ATTTCGAATTTCGAAGACATTGGCTTGGAAGATGATGCCGCCCTATCGTTCGCCGATCCA

CCAGGCTCCTACGGCCGCTCTGGGGCAAAGGCACGGTCTCCCGCCGCCGGAATGTCAAGC

AAAGTCCCTACGATCTCAACTCGCGACTTGACCGCAACCCCGAAATCTCGCCAGCGAAGC

CCCCTACCCCCGATTCACGGTGGGTTGGCCAATGCGCCAGCAGGCTCCCTCGCAGCGAGA

ACTGCAAATCGGACACACTCGGGCAACACGCTGCAGTCACCTGGTTTGACACCAGCGCAC

GCCCGCCGTGCACGATCTGTTTCACCACTACGGCCGACATCCAGTCGCTCCACCTCGAGC

ACCAGCCCAGCTTTGTCTCCGCTATCTGCGCGAGTGCCAACTCAGAAACAAAGCTGGCAG

CCAAATCGCAAGTCGGTTCAAGACCTGGAGGAAGAATACCATGATTCGGACGATGAACTG

CCGGACGATGCAAGTCTGTGGAACATTCCTATCTCTCCACGCCCAGTCCAAGAGCGGACT

TTTTCACGATCTGCCAGTCCAAATGGTCGCAGCCCTGGGCGGCGGCCTCTGCCTATTCAG

CACACCGTGGCGGAAACCGACAAGCTAGCGTCACCCGAAAATACGGCCAAGGCATCCCGC

ATGAAGCGTATACAACGATCGAGCTCCGCGGGGCCGGAGCGTGGACAAATCTCGCCACGC

AACCCTCGCACCTACTCGTATAACAGTTATCTTTCTGACTTGTCCGAGGAAGCAAAGATT

ATTACTGAAGCTCTGGAGCTGCATGCTGATGATAAAGATCGCGAGCGGGAGGAGCATGTT

CAAACCCGTGCTTCGCGGAAGTCTAGCGAGGACTCGCATCGTGCATCCCGTGATGCTGTC

GCCTTACCGCCTCTGCAGAAATCAAACATCATGATTGATCCGCTGCCAATTAGCAAAGAG

AAAGAAAAATTCCTCTCGCGGACACGCCCAAGCTGGCTTCCGCCTAAGGACCAGAAGGAG

GAGAAAAGACACCTGAGGCAGTATCAGCAAATGATGGCCCAGTCTCGAGAAGCTGAAAAA

CGCAAAGCTGCCAAAATTGCGAATGCGCAGTGTGCAAAGGACAACACCCGAGCAACACTG

CAGAACATCTGGGATGAATATGTGTATCCGAATTGGGACCGCGCTCTTCGCGAGCCCCGA

ATTCGCGAATTGTGGTGGCGTGGAATCCCACCCCGCAATCGGGGCCACATATGGGAACGA

GCCATTGGCAACGAACTAGCATTAACTGAGGAAACTTTTGCCAAAGCGCTTCTTCGAGCA

AAGGATCTCCAAAGCAAGAAGGATGGAGAAAGCGAGAGCAACAAAAGACTACAAGACTGC

TTTGAAGCGATCGAAACAGACGTGCCCAAAGCCTTCCCAGATCTGAACATCTTCCAAGAA

GGTGGTCCACTACGGGAAATCCTCATTGACGTCCTGAAAGCATACTGCATGTACCGCAGC

GACGTAGGCTACATCCACGGCCTGCACACCATCGCCGCCCTCTTCGTTTTGCAATTCCCC

ACACCCGCTTCCGCCTTCCTCGCCATGGCCAACGCCCTCAACCGTCCTTTACCCGTCGCG

TTCCTCACTTGGGACCGTGGCGCCATGGCCCGCTCCTACGCACTAGCCTCGGACACCTTG

CGCTATAAGTTCCCTCGTTTGCATACTCACCTGACCGAAATCCTCCGCCTCTCCGAGGCA

GAGATCTGGGAGCCGATTTTCCGCTCGCTGCTGACAAACGGCCTGGACCTCGAACGCATC

TCCCGTGTCTGGGACTGCTGGGTCTTCGAGGGCGACCGCATCATGATCCGCTCTGCCGTT

GCCATAATGGGCTGTCTCCAGGCCCAGCTGTTCTCCTTCCAACAGACTAATGACCAATCC

CGTCTTGCTGTGCGGGACATGCTAGGCTGGGGTCCCCATAATCTTGATCTTGGAGCAAAC

ATGCAGAAAGCTAAAGAGCGGAACAGCGCCCCTGCTGCTGGCTTCGGCGGGGGGCGGATC

GAAAATTTCGGCGTTGGTGATTATTGGGTTCTTACTGCTGCGGGCAACGAAGATGGGTTT

ATGAGTGCCGTCCGAGAGGCTGGGAAAGTGCGCCAACAACCCCAAACT

>CL100.Contig2_All 1824 2273 hypothetical protein PDIP_58440 [Penicillium digitatum Pd1] >gi|425773321|gb|EKV11680.1| hypothetical protein PDIG_49060 [Penicillium digitatum PHI26]

TCAGAAAAACGCAAAGCTGCCAAAATTGCGAATGCGCAGTGTGCAAAGGACAACACCCGA

GCAACACTGCAGAACATCTGGGATGAATATGTGTATCCGAATTGGGACCGCGCTCTTCGC

GAGCCCCGAATTCGCGAATTGTGGTGGCGTGGAATCCCACCCCGCAATCGGGGCCACATA

TGGGAACGAGCCATTGGCAACGAACTAGCATTAACTGAGGAAACTTTTGCCAAAGCGCTT

CTTCGAGCAAAGGATCTCCAAAGCAAGAAGGATGGAGAAAGCGAGAGCAACAAAAGACTA

CAAGACTGCTTTGAAGCGATCGAAACAGACGTGCCCAAAGCCTTCCCAGATCTGAACATC

TTCCAAGAAGGTGGTCCACTACGGGAAATCCTCATTGACGTCCTGAAAGCATACTGCATG

TACCGCAGCGACGTAGGCTACATCCACGGC

>CL102.Contig1_All 2 745 hypothetical protein PDIG_43150 [Penicillium digitatum PHI26] >gi|425778528|gb|EKV16652.1| hypothetical protein PDIP_34380 [Penicillium digitatum Pd1]

GAGCACATGAAATCACACATGGCAATTTTTCGCAATAAGTTGAATAAACCCACACGAGAT

GAATATCTCCTAGCGCTCTCTTGTGTTCCCATTGTTTCAACCCAGTACAATCGAAATCCG

GGTGCTTGGGCCCGAGAGGAACGGGAGACGCTGGAGCGCCAAATGGCCATGATGAACCGC

TATCGCTCAGGAAACTACGAACCAAAGTTGAAGAAGATTGCTCCTGCTCCAGTTAAAAGA

TCAGGAACTCAACCGCGCGTGCAGCGAAGCCGGGTCAAACGTACCCCTAAATCCACACCT

AAGCAGCAGGTGTTTGATAACTTCGATCCCCCCCAAACTCCGCTTACCAAACCGCGTGCC

TTGGGTTCGAATCGCGATGACACCGATTATCACTCACTCAGGGACTATTCACCCCCGGTA

GACACGCTAGGCAGTAATGCCAAAGCATTGAAGGCCGATTGGAAGGGGCAGATGTTAGAT

TTGAGCAATGACCCGGACAGAAACAATCTCTGTCCCGCCGAGCTCAGTCTAGCCTCCACC

CTGAGACTTTCATGTGCGACTTACCTATGCAGCAAGCGTCGAATTTTTGAAGCTCGAGTT

CGAGCTCTTGGCGTTGGCAAAGAGTTTCGCAAAACAGATGCGCAGCAGGCTTGCAAGATC

GATGTCAATAAAGCCAGCAAACTTTGGACCGCTTACGAACGTGTGGGTTGGTTTAAAGCT

GAACATTTCCACCAGTACCTAGCA

>CL102.Contig2_All 2 745 hypothetical protein PDIG_43150 [Penicillium digitatum PHI26] >gi|425778528|gb|EKV16652.1| hypothetical protein PDIP_34380 [Penicillium digitatum Pd1]

GAGCACATGAAATCACACATGGCAATTTTTCGCAATAAGTTGAATAAACCCACACGAGAT

GAATATCTCCTAGCGCTCTCTTGTGTTCCCATTGTTTCAACCCAGTACAATCGAAATCCG

GGTGCTTGGGCCCGAGAGGAACGGGAGACGCTGGAGCGCCAAATGGCCATGATGAACCGC

TATCGCTCAGGAAACTACGAACCAAAGTTGAAGAAGATTGCTCCTGCTCCAGTTAAAAGA

TCAGGAACTCAACCGCGCGTGCAGCGAAGCCGGGTCAAACGTACCCCTAAATCCACACCT

AAGCAGCAGGTGTTTGATAACTTCGATCCCCCCCAAACTCCGCTTACCAAACCGCGTGCC

TTGGGTTCGAATCGCGATGACACCGATTATCACTCACTCAGGGACTATTCACCCCCGGTA

GACACGCTAGGCAGTAATGCCAAAGCATTGAAGGCCGATTGGAAGGGGCAGATGTTAGAT

TTGAGCAATGACCCGGACAGAAACAATCTCTGTCCCGCCGAGCTCAGTCTAGCCTCCACC

CTGAGACTTTCATGTGCGACTTACCTATGCAGCAAGCGTCGAATTTTTGAAGCTCGAGTT

CGAGCTCTTGGCGTTGGCAAAGAGTTTCGCAAAACAGATGCGCAGCAGGCTTGCAAGATC

GATGTCAATAAAGCCAGCAAACTTTGGACCGCTTACGAACGTGTGGGTTGGTTTAAAGCT

GAACATTTCCACCAGTACCTAGCA

>CL102.Contig3_All 2 217 Pc12g05070 [Penicillium chrysogenum Wisconsin 54-1255] >gi|211581973|emb|CAP80134.1| Pc12g05070 [Penicillium chrysogenum Wisconsin 54-1255]

GCCGATTGGAAGGGGCAGATGTTGGATTTGAGCAATGACCCAGACAGAAATATTCTCAGT

CCCGCTGAGCTCAATCTCGCCTCTACGCTGAGACTTTCATGTGCGACCTACCTATGCAGC

AAGCGTCGGATCTTCGAAGCTCGGGTTCGAGCCCTTGGTGTAGGCAAAGAGTTTCGCAAG

ACGGATGCCCAACAGGCTTGCAAGATCGATGTCAAT

>CL103.Contig1_All 265 1578 minus strand Pc13g13260 [Penicillium chrysogenum Wisconsin 54-1255] >gi|211584360|emb|CAP92395.1| Pc13g13260 [Penicillium chrysogenum Wisconsin 54-1255]

ATGGCCGCCTCAATCAACCACTCACACCCGCGTCATGAAGTACCTGACACAAGCCCCAAA

ATGGTCAATCGCTTGCAGCAAAGCAAATCACCTTATGTCCGGGCACATATGAACAACCCG

GTAGCATGGCAAGTTTGGGATGCAGAAGCTATGGAGCTTGCTAAGAAGCACAATCGTTTA

ATCTTTCTCAGTATCGGTTACTCCGCATGTCATTGGTGCCATGTCATGGAAAAAGAATCG

TTCATGTCATCAGAGGTGGCCTCGATTCTAAATGAATCCTTCGTCCCCATCAAGGTTGAT

CGCGAAGAGCGACCAGACATCGACGACATCTACATGAATTATGTCCAGGCCACGACGGGT

TCTGGTGGCTGGCCCTTGAACGTGTTCTTGACTCCTGACCTCGAGCCTGTGTTTGGTGGC

ACGTACTGGCAAGGCCCAAATTCAACCACATTCACCGGTCCCGAAGCAATCGGATTTGTG

GAGATTCTAGAAAAACTTAGAGATGTGTGGCAGACACAACAGCAACGCTGTCTCGACAGC

GCCAAAGAAATCACCAAACAATTGAGAGAGTTTGCCGAGGAAGGGACGCACTCGCAGCAA

GGCGATCGCGATGATGATAATGATGAGGATATGGATATTGAGCTTCTGGAAGAAGCATAT

CAGCATTTTGCATCACGATATGACTCGGTCAATGGCGGGTTTGGCCGTGCCCCCAAATTT

CCTACACCGTCCAATCTTAGCTTCTTACTCCGGCTCGGTGCCTACCCGACCCAGGTCATG

GATGTTGTAGGACATGATGAATGTGAACAAGCAACCGCGATGGCCGTCACAACACTAGTG

AATATGGCTCGTGGTGGTATTCGGGACCACATCGGGCACGGATTCGCACGGTATAGCGTG

ACGACAGATTGGGGTCTCCCCCATTTCGAGAAGATGCTTTATGACCAGGCACAGCTATTG

GATGTCTACGTGGATGCTTTCCGGCTCACCCATGACCCCGAACTTCTGGGCGCTGTCTAT

GATCTGGCTGCTTACTTGACGAGCGCCCCGATACAGTCGCCCACCGGAGGGTTCTTTTCT

TCAGAAGATGCCGACAGTTACCCTCATCCAAATGACACCGAAAAGCGGGAGGGCGCCTTC

TATGTCTGGTCGCTGAAGGAACTCACTTCAGTCCTCGGCCCTCGGGATGCTCCAGTTTGC

GCCAAACACTGGGGTGTTCTCCCCGACGGCAACGTTCCACCCGAGTACGACCCGCATGAT

GAGTTCATGAACCAAAACGTGTTGTCCATCCGTGCCACGCCCAGCAAACTCGCC

>CL103.Contig2_All 210 1523 minus strand Pc13g13260 [Penicillium chrysogenum Wisconsin 54-1255] >gi|211584360|emb|CAP92395.1| Pc13g13260 [Penicillium chrysogenum Wisconsin 54-1255]

ATGGCCGCCTCAATCAACCACTCACACCCGCGTCATGAAGTACCTGACACAAGCCCCAAA

ATGGTCAATCGCTTGCAGCAAAGCAAATCACCTTATGTCCGGGCACATATGAACAACCCG

GTAGCATGGCAAGTTTGGGATGCAGAAGCTATGGAGCTTGCTAAGAAGCACAATCGTTTA

ATCTTTCTCAGTATCGGTTACTCCGCATGTCATTGGTGCCATGTCATGGAAAAAGAATCG

TTCATGTCATCAGAGGTGGCCTCGATTCTAAATGAATCCTTCGTCCCCATCAAGGTTGAT

CGCGAAGAGCGACCAGACATCGACGACATCTACATGAATTATGTCCAGGCCACGACGGGT

TCTGGTGGCTGGCCCTTGAACGTGTTCTTGACTCCTGACCTCGAGCCTGTGTTTGGTGGC

ACGTACTGGCAAGGCCCAAATTCAACCACATTCACCGGTCCCGAAGCAATCGGATTTGTG

GAGATTCTAGAAAAACTTAGAGATGTGTGGCAGACACAACAGCAACGCTGTCTCGACAGC

GCCAAAGAAATCACCAAACAATTGAGAGAGTTTGCCGAGGAAGGGACGCACTCGCAGCAA

GGCGATCGCGATGATGATAATGATGAGGATATGGATATTGAGCTTCTGGAAGAAGCATAT

CAGCATTTTGCATCACGATATGACTCGGTCAATGGCGGGTTTGGCCGTGCCCCCAAATTT

CCTACACCGTCCAATCTTAGCTTCTTACTCCGGCTCGGTGCCTACCCGACCCAGGTCATG

GATGTTGTAGGACATGATGAATGTGAACAAGCAACCGCGATGGCCGTCACAACACTAGTG

AATATGGCTCGTGGTGGTATTCGGGACCACATCGGGCACGGATTCGCACGGTATAGCGTG

ACGACAGATTGGGGTCTCCCCCATTTCGAGAAGATGCTTTATGACCAGGCACAGCTATTG

GATGTCTACGTGGATGCTTTCCGGCTCACCCATGACCCCGAACTTCTGGGCGCTGTCTAT

GATCTGGCTGCTTACTTGACGAGCGCCCCGATACAGTCGCCCACCGGAGGGTTCTTTTCT

TCAGAAGATGCCGACAGTTACCCTCATCCAAATGACACCGAAAAGCGGGAGGGCGCCTTC

TATGTCTGGTCGCTGAAGGAACTCACTTCAGTCCTCGGCCCTCGGGATGCTCCAGTTTGC

GCCAAACACTGGGGTGTTCTCCCCGACGGCAACGTTCCACCCGAGTACGACCCGCATGAT

GAGTTCATGAACCAAAACGTGTTGTCCATCCGTGCCACGCCCAGCAAACTCGCC

>CL104.Contig1_All 1107 1319 minus strand Superoxide dismutase [Penicillium digitatum Pd1] >gi|425771975|gb|EKV10403.1| Superoxide dismutase [Penicillium digitatum PHI26]

TCAATGCGCATTTCGACATTTCGACACGCTCTGAGTGCGTGGTATGTCTCGATATCATCC

GTTCGGAAGATTGCTCTCGTTTTATGGGTGGTTGCATCCGAGTCACGGAAGAAGAGAGAG

TATGATGACATTGAGGCAATCGACGATGATGATGCAAAGGATGATACTGATGCTGGCGAC

GAGGATAATGACGTATCTGGAATGTTTGAGCTC

>CL104.Contig2_All 2 223 conserved hypothetical protein [Aspergillus flavus NRRL3357] >gi|220699730|gb|EED56069.1| conserved hypothetical protein [Aspergillus flavus NRRL3357]

ACGAGAGCAATCTTCCGAACGGAAGACGCAGAAACATATCATGCACTCAGAGGATGTGGA

AATGTCGATATGCGCATTGAGAAATTTGGTGATCTCTCCTCTACAGCTCCATCCACAACT

CCTCTTCATCAGTTCCGCTTGAATATGGGACAGGACAAGAACCACAGTACAGTGAATCCG

ACAGAGATCGAGTTCGAGCTGCCCGAAAGGCTGGACCTGGGC

>CL104.Contig3_All 23 652 manganese superoxide dismutase AAC36583-Penicillium chrysogenum [Penicillium chrysogenum Wisconsin 54-1255] >gi|7388256|sp|O75007.1|SODM_PENCH RecName: Full=Superoxide dismutase [Mn], mitochondrial; Flags: Precursor >gi|3642873|gb|AAC36583.1| manganese superoxide dismutase [Penicillium chrysogenum] >gi|3642877|gb|AAC36585.1| manganese superoxide dismutase [Penicillium chrysogenum] >gi|118429506|gb|ABK91801.1| Asp f 6 allergen-like [Penicillium citrinum] >gi|211586343|emb|CAP94092.1| manganese superoxide dismutase AAC36583-Penicillium chrysogenum [Penicillium chrysogenum Wisconsin 54-1255]

ATGGCTTCTCAAACACACACTCTGCCTCCTCTCCCATATGCCTACGATGCGTTGGAGCCC

GTCATCTCCAAGCAGATAATGGAGCTGCATCATCAGAAGCACCACCAAACATACATCAAC

AATTTGAACGCAGCGCTCTCCGCCCAAGCTTCTGCAACAGCATCAAATGATGTCCCCACG

TTGATTTCATTGCAACAAAAGCTCCGCTTCAACGGTGGAGGCCATATCAACCACTCCCTA

TTCTGGAAGAACCTAACTCCCCCCGGCACACCTGCGAATGACATCGCTGGGGCTCCCACG

CTGCGCGAAGCTATCGTCTCCCGCTGGGGCTCGCATGAAGCGTTTGTCAAGGCTTTTGGC

GCCGAGCTGCTCAGTCTTCAGGGCAGTGGCTGGGGATGGCTGGTGAGCAAGGGTGGTGCC

AAGGGACGACTTGAGATCGTTACGACGAAGGACCAGGACCCTGTTAATGCACCTGATGTG

CCTGTCTTCGGTGTGGATATGTGGGAGCATGCCTACTACCTCCAGTACCTGAACAACAAG

GCTGGCTATGTTGAGGGGATTTGGAAGATCATTCACTGGGCTGAAGCTGAGAAGCGTTAC

ACTGCTGGTGTTGAGAACCCGCTGAAGCTG

>CL105.Contig1_All 12 608 minus strand Pc20g05520 [Penicillium chrysogenum Wisconsin 54-1255] >gi|211587821|emb|CAP85881.1| Pc20g05520 [Penicillium chrysogenum Wisconsin 54-1255]

ATGCGAAAACATTATCCCTTAGATTCCCCTTTTTCACATTCCTTTTCTTCCTTGTCTTCA

TCTTCCTCTTCCTCTTCTTTCTATCCTTCATCATATTGTAGTTCATTTTCCCTAGGCCTA

TCATCAGCCTCGCACCAACACTTAGCATTCCAGCCAAAAGATTATTGGGAAAGTCTGGTC

ACCAAACACGGTTACCCCACACCTGTGTCACTCTCCTCGTTGCGAGATTTGACAGCCACC

TCGTCTACTGATACACCCTCTTCACCACCTCGTCAACCCGGTTGTCCGAATACCATCCCG

ACCGACGCTTTTGACTCATTTACCGTATCCTCATCCACGAGACCCATTCCTATCCCAAGC

CGCCCTGGTCCAATCTACGAGGACCTTCCTGTGACACCCCTCACCGGTCGATTTGATCAC

GAATCCTATTTTGACGATTGGGAACGTGCGTCTCAATCCGCCAAATTTCGACACATTCCT

TTAATCCCGCCTGCCCGTCGCGACCAGCATCCATCGCGGCCTCCGCCAGCGTCGACAAGG

ATGCGTTCTGATAACTCACCTTACTACTCCCCGGCTGCCTCGCCTATCATGTCTCCA

>CL105.Contig2_All 93 578 minus strand Pc20g05520 [Penicillium chrysogenum Wisconsin 54-1255] >gi|211587821|emb|CAP85881.1| Pc20g05520 [Penicillium chrysogenum Wisconsin 54-1255]

CTATCATCAGCCTCGCACCAACACTTAGCATTCCAGCCAAAAGATTATTGGGAAAGTCTG

GTCACCAAACACGGTTACCCCACACCTGTGTCACTCTCCTCGTTGCGAGATTTGACAGCC

ACCTCGTCTACTGATACACCCTCTTCACCACCTCGTCAACCCGGTTGTCCGAATACCATC

CCGACCGACGCTTTTGACTCATTTACCGTATCCTCATCCACGAGACCCATTCCTATCCCA

AGCCGCCCTGGTCCAATCTACGAGGACCTTCCTGTGACACCCCTCACCGGTCGATTTGAT

CACGAATCCTATTTTGACGATTGGGAACGTGCGTCTCAATCCGCCAAATTTCGACACATT

CCTTTAATCCCGCCTGCCCGTCGCGACCAGCATCCATCGCGGCCTCCGCCAGCGTCGACA

AGGATGCGTTCTGATAACTCACCTTACTACTCCCCGGCTGCCTCGCCTATCATGTCTCCA

CGACGC

>CL105.Contig3_All 12 122 minus strand Pc20g05520 [Penicillium chrysogenum Wisconsin 54-1255] >gi|211587821|emb|CAP85881.1| Pc20g05520 [Penicillium chrysogenum Wisconsin 54-1255]

ATGCGAAAACATTATCCCTTAGATTCCCCTTTTTCACATTCCTTTTCTTCCTTGTCTTCA

TCTTCCTCTTCCTCTTCTTTCTATCCTTCATCATATTGTAGTTCATTTTCC

>CL106.Contig1_All 2 277 minus strand Pc22g00560 [Penicillium chrysogenum Wisconsin 54-1255] >gi|211591118|emb|CAP97344.1| Pc22g00560 [Penicillium chrysogenum Wisconsin 54-1255]

CGTCGTCCTCACCACAACAGCCATCACAAGAACTCCTTTATGCGAATCATCCGCCCCATC

ATTCTTCCTGCTTTGCTGGGTACTGCCGCTGGGCTGGTGGCCTGCTTGGTCGGGTTCTTC

ATCGGAGAGCTGCTGATGTCCTTGGGAGTACGTCTTGGATGGCAAAAGGTACTAGGCGAT

AGGTCACGGATTATCTCAGTGGAAGAAGGCACGGTTTCTGAAAAGACCCCCATGGTGCCG

CATGTTTATGTGACAGACACTACCCAGTCAAATGTA

>CL106.Contig2_All 311 1105 minus strand hypothetical protein PDIG_75870 [Penicillium digitatum PHI26] >gi|425775592|gb|EKV13850.1| hypothetical protein PDIP_46340 [Penicillium digitatum Pd1]

ATGCAGCTCAATGCGCCCCTGTACGAAGACACAGACCAAATCCATCCCAGTGACAAAACC

GCCGAATTATCCTACACCCTTGTGACTCAACCAATCCCCACAAACGCATTAGGATCCATG

GCGGATATGATCCGCGTGCGAGTCGAGCTGTTCGATCTACAGGGCAACCTGGTGTCACCG

GACGCAGTGCTCGTGGACCTTCTTGTTTACCAAAATGGAAAGTACGGCATGACCCGTATC

CGTGTCGAACCAGCTCGTGGCAGCGATCAGGAAGGTACTTTCTATCAAAAGAGTCAACTG

TGGGTAGTGAACTACTGGAGGACCCAATTCGGTTCCATTTTCGATCAGTCCAGGACTAGG

ACGATGGGGCTACCCCACGATTCAGCCCCGATTATGGAACCCTCCGCCCACAAATGCGCG

ATCAAGGCCATTGGCAGTGGCACCGCAACCGACTCTACTGCCAAGACTCGTTTTTTCTCG

TTCTGGGCTACTCCAGCTCATCTTCACCACCATACTGAACACCGTCCTCACTACAGCTAC

CATCACAAGGATTCCTCTGTCCGCACCGTCCGTCCCATCATGATTCCTGCTTTGATTGGC

ACTGCCGCTGGCCTGATGGCTTGTTTGATCGGGTTCTTCGTCGGAAGCCTGCTGATGTCC

TTGGCGGTGCGCCTCGGATGGCAAATGGTACTAGACCATAGATCACGGATTGTCTCAGTC

GAAGAAGGCATGGTCTCTGAAAAGGCCGCCACAATGCCGCAAGATTACGTGACAGTTATC

TTCGAGTCGAATATA

>CL107.Contig1_All 2 2719 minus strand hypothetical protein PDIP_61050 [Penicillium digitatum Pd1] >gi|425777173|gb|EKV15357.1| hypothetical protein PDIG_26600 [Penicillium digitatum PHI26]

GACCATACCAAGCAAGACAAATCCAATCAAGTCGATTGCTTTGAAGAGAAGACTATAGTC

TCATCACCGCCGGCTGACCCATTTTCCCCTGGGGCGGCCTGTGTACCCTTGCCTTCACCA

TCTTTGACTGAAGCACGGTATATGTCAAGTCCCTCTCAGGACAACCAGCTTTATTATCCT

GGACCATCAGCATACTCCCCATACGCTTCACCCGTCCCCCGCTCTGCGCCAGTACCTTAC

GCTTCATCGGTGCCACACGTTGCGTCCCCGGTGCCGTACAGTCCGCCTAACGCCTACTCC

TCTCCGGTACCCTACGCATCACCTGCGCTTTACGCCCCAGTGCCCTACACTGGTTCCCAG

GTGCCTTACACTTCATCTACACCGTGCGCTGGCTCTCCAGTGCCTTACAGCTTGCCGGGA

CCGTACGCTGGTTATCCGGCGCCCTACACCTCACCAGTAGCTCATACTGCCTCACCGGTG

CCCAAAGTCAGCAGCCCACTTGCCCATTCCCATTACCCTAAAATGTCTCCAAATATTTCA

CCAACTACAACTCCCCCACCTCAGAATCCACCGGCGGCTCCGATGGCTCCTCCAGAAGCG

CCTTCTATAGCCCCAAGTGGACCGCATGCCCTCCAATCTCCCGTCATGAGTTCCACCGGA

ATGGTACCTCCTTTTGGGCATTATTCACCTCATCACCAATCGGAGGCCCATTATGTGAAC

CGGAGCTACAGTATCCCAGATTCATCATATCAAGCATCATACCAGGCTCTTCAAAACCTG

TCCATGGGCAATAGCTATCCTCTTGAGAATGGGTCTCCCCCAGAGAACAATAGTGAGGAC

ATAGAGCTTCTTCAACGCATCCAGTCAGCAATTCCGGACATCAACCGTCTTCTTCATGGG

TTTCGTAGCACCCATAACAAGCTCTCAAACCGGGAGGCTGAGATGAAGCACCTTGGAAGT

CAGCATGACCAAGCTCTAATGCACAAGGACTACTACATTGAAGCTCTGCAAGCTCAAATG

AAGAAGACTGCCAATGAAAGTGCTGAAGAAGACGCCAAGCTGAAACATACCATTAGTGAA

CTACGTCTCGAACTCGGGGATCTGCAAGAGAAGCAAAGGGACCTTGAGGATGGCTTAGCT

GCGCAACAGAGGTCCAATGAGGAGCTTTCTGAGACTAAGGTCGGACTTGAGGGAAAAATT

GATCAGCTTGATGAAAGTATTAAGGAGTCAAAAGAAGCCCATGAGAAAGATTTGGAGACT

CTAAGGGAGGAACATGAGAAGGCTCTCGTGGCACAGAAGGAGGAGCTTACTGAATTATTT

GAGGAGATCAAGGCTGAGGATGAGAAAACGGCAGCCGAGACTCTGGAGACTCGTGAGCGC

GAACTTCGCAGTGAACACGAAGCTAGTCAAAACGAATGGGAGAAAGTGAAGGCCCAATTG

CAGGAATCTTTCGAAGCCCAGTGCACAGAGTTAGAGGCCATCAAGACAGAAGTGGCATCC

CAGATCACTGCTTTGGAATCCAAGGAGACCGAGTTGCAAGCCCGACTCACTGAACTCACA

TCCACTCGTGAGGAACTAGCAGCTAAGCTAACAGAGCTGGAAGAAACTCAACAGAAGAAC

ACCCGGGAAACGGAAAAACTTCGCCAAGGCCATGCTGGTGAATTGGACTCCTTGCGACAA

TCTTCCGACGAACAACTCGCTGCTGCTGTCAAAGAGTTGGCCGACAAGATCGCATCTCTG

GAGGCCCACTTCAATGAGAAAGAGCAGCATTGGACCACAGAGCGCGCTGCCCTGGAGGAG

CAACTCTCGGAGAAAAGTGGCGAGCTTTCCAGTGCTGAGCGAGAGAAGGAAAGGTTGGAA

GGAGACAATATCCTCAAGGAGAAGCACCTTCAACGCGCGGTGGATGGGATGCGAATGACC

ATCGATAACTTGGGATCGGACTGTGACAGGCTGAGAAAGACACTTTCCAGCCTGGGAGAG

GCCACTGACCTCAGAAGCACCAAGGGCGATCAATTCTTCTTTGACTGCTTTACAGAGCTC

TCGCAGCTCATTCAAAATATATCCAAAGAGCACTTCGGATATCTCCCCATCGATCCCCCA

AAAGACATCCTGTCCAAGATTCCATCCGAACTCCCACCTTTCCTTGACAACACCCCAACC

TCTCGTGAACTCCGATGTGCCTATATCCAGCATATCATCTCCAAAACCCTAACCTTCCGC

GTCTTCCAGCCTTTCCTCTTCACCCTGGGCCGGCGGTACGACAAAGCCGACACCTTCTTC

CAGATGCTATCCATGGACATCCGACGCAAGTCCGTCCGGCGCGAGGCATTCTGGCGTCAA

CAGACGCTAAAAGCGGCCTACACAACCTCAGACGCAAAGCAGTCAATTAATGTCGCAGCA

GCAGTCATCGTAGACGAAATCATTGACCACATAAAACACTTCGCCGATCCTAAGCACCTC

GACTCCCTCCTGGTCGGCGTCCGGAAGATCGTCAAGCTCGCCGCCGAGACATGGAGACAT

GCACGTGTCGAACGCGAGCTCGTACTCGCTAATTTCCCCGCCCCGGATGCAGAGAGCATC

TCGAATGAGGACTGGCTGGAGTACGCCGCCAACAAGGATCAGAAACACACCCCGTCAAAC

GAGCCCACTCGCCACGTCGTGCTGCGCACCTTCCCGCGTATTACACGCGAGGCTGCTCAC

GAGGATTTTGCTAGCGAT

>CL107.Contig2_All 2 2017 minus strand hypothetical protein PDIP_61050 [Penicillium digitatum Pd1] >gi|425777173|gb|EKV15357.1| hypothetical protein PDIG_26600 [Penicillium digitatum PHI26]

GACCATACCAAGCAAGACAAATCCAATCAAGTCGATTGCTTTGAAGAGAAGACTATAGTC

TCATCACCGCCGGCTGACCCATTTTCCCCTGGGGCGGCCTGTGTACCCTTGCCTTCACCA

TCTTTGACTGAAGCACGGTATATGTCAAGTCCCTCTCAGGACAACCAGCTTTATTATCCT

GGACCATCAGCATACTCCCCATACGCTTCACCCGTCCCCCGCTCTGCGCCAGTACCTTAC

GCTTCATCGGTGCCACACGTTGCGTCCCCGGTGCCGTACAGTCCGCCTAACGCCTACTCC

TCTCCGGTACCCTACGCATCACCTGCGCTTTACGCCCCAGTGCCCTACACTGGTTCCCAG

GTGCCTTACACTTCATCTACACCGTGCGCTGGCTCTCCAGTGCCTTACAGCTTGCCGGGA

CCGTACGCTGGTTATCCGGCGCCCTACACCTCACCAGTAGCTCATACTGCCTCACCGGTG

CCCAAAGTCAGCAGCCCACTTGCCCATTCCCATTACCCTAAAATGTCTCCAAATATTTCA

CCAACTACAACTCCCCCACCTCAGAATCCACCGGCGGCTCCGATGGCTCCTCCAGAAGCG

CCTTCTATAGCCCCAAGTGGACCGCATGCCCTCCAATCTCCCGTCATGAGTTCCACCGGA

ATGGTACCTCCTTTTGGGCATTATTCACCTCATCACCAATCGGAGGCCCATTATGTGAAC

CGGAGCTACAGTATCCCAGATTCATCATATCAAGCATCATACCAGGCTCTTCAAAACCTG

TCCATGGGCAATAGCTATCCTCTTGAGAATGGGTCTCCCCCAGAGAACAATAGTGAGGAC

ATAGAGCTTCTTCAACGCATCCAGTCAGCAATTCCGGACATCAACCGTCTTCTTCATGGG

TTTCGTAGCACCCATAACAAGCTCTCAAACCGGGAGGCTGAGATGAAGCACCTTGGAAGT

CAGCATGACCAAGCTCTAATGCACAAGGACTACTACATTGAAGCTCTGCAAGCTCAAATG

AAGAAGACTGCCAATGAAAGTGCTGAAGAAGACGCCAAGCTGAAACATACCATTAGTGAA

CTACGTCTCGAACTCGGGGATCTGCAAGAGAAGCAAAGGGACCTTGAGGATGGCTTAGCT

GCGCAACAGAGGTCCAATGAGGAGCTTTCTGAGACTAAGGTCGGACTTGAGGGAAAAATT

GATCAGCTTGATGAAAGTATTAAGGAGTCAAAAGAAGCCCATGAGAAAGATTTGGAGACT

CTAAGGGAGGAACATGAGAAGGCTCTCGTGGCACAGAAGGAGGAGCTTACTGAATTATTT

GAGGAGATCAAGGCTGAGGATGAGAAAACGGCAGCCGAGACTCTGGAGACTCGTGAGCGC

GAACTTCGCAGTGAACACGAAGCTAGTCAAAACGAATGGGAGAAAGTGAAGGCCCAATTG

CAGGAATCTTTCGAAGCCCAGTGCACAGAGTTAGAGGCCATCAAGACAGAAGTGGCATCC

CAGATCACTGCTTTGGAATCCAAGGAGACCGAGTTGCAAGCCCGACTCACTGAACTCACA

TCCACTCGTGAGGAACTAGCAGCTAAGCTAACAGAGCTGGAAGAAACTCAACAGAAGAAC

ACCCGGGAAACGGAAAAACTTCGCCAAGGCCATGCTGGTGAATTGGACTCCTTGCGACAA

TCTTCCGACGAACAACTCGCTGCTGCTGTCAAAGAGTTGGCCGACAAGATCGCATCTCTG

GAGGCCCACTTCAATGAGAAAGAGCAGCATTGGACCACAGAGCGCGCTGCCCTGGAGGAG

CAACTCTCGGAGAAAAGTGGCGAGCTTTCCAGTGCTGAGCGAGAGAAGGAAAGGTTGGAA

GGAGACAATATCCTCAAGGAGAAGCACCTTCAACGCGCGGTGGATGGGATGCGAATGACC

ATCGATAACTTGGGATCGGACTGTGACAGGCTGAGAAAGACACTTTCCAGCCTGGGAGAG

GCCACTGACCTCAGAAGCACCAAGGGCGATCAATTC

>CL109.Contig1_All 1079 3514 putative RNA-directed DNA polymerase from transposon X-element [Penicillium digitatum PHI26]

AAAGTACCAGTATATTGCTGCAAAGCCTGCTGGGAACACCTCAAAGACATAGTCCTCTCT

CTGTTTAACCGATGCCTCGCCCTCTGCCACATCCCCCTAGCCTGGAAGGTCGCGGAGGTG

GCTATGATACCCAAGGTCGGCAAGAAGGATAAGTCCTCCGTGCGCTCCTGGAGACCGATC

GCCCTCCTATCCTGTCTCTCTAAGGGACTAGAACGAATCGTCGCGAAAAGAATTGCCTGG

ACTGCCCTCACCCACGGCGTCCTCAGCCCCCAGCATGCGGGGGCTCTACCCAAACTTTCC

GCCACAGACCTAGTCGCCTCCTTCACCCACGATGCCGAAATGGCACTCTCACAGAACAAG

CAAGTCACCCTAGTCACGATGGACGTCCAGGGTGCATTCGATGCCCTCCTCCGGAGACGA

CTTCTCAAGCGCATGGGCGAGCAGGGCTGGCCACGAGAACTGCTACTTCTTGTGGACAGC

TTTCTCACCGGACGCAAAGCCCGCGTCCGGCTAGAAGGCTCAACCACCCCTGAGTACGAC

GTGGTCTGCGGAACCCCCCAAGGCTCCCCATTATCACCAGTACTATATATGCTCTACCTA

GCCGAACTCCTCAATATGGACCAAACGCTCCGTTTCGGGTATGCCGACGACCTAGCTCTA

TACAGAGCCTCCCACGATCTCACCCAGAATGTCAGACTCCTCGCTAAGGACGTTCAGAGC

ATTCTTGCCTGGGGAGAATATAATAAAGTGGCCTTCGCCCCCGAGAAACTAGAGATGATC

CATATCACTAGACATCGAGGGGATGAGTCCCCTTCAATTGTAGTCAACGACCGGCTCACC

ATCGACCCCGTTCAGGCCAAGAAACCAGGATACACACCCACTCTCCGCTGGCTGGGAGTC

TTTTTCGATAGAAAGCTCACATGGAGAAGCCACATTCTCGCCCGGGCGGGCAAGGCACGC

GCTGTCGCACAACATATCCGCAATCTGGCCCGCACGACCTGCGGCCCGCCCGCGAGCTCA

CTTCGCAAAGCAGTCATCACCTGTGTTATACCCTCCCTAACGTTCGGAACTGAGGCCTGG

TATGGCGGCCGGAATAGGCCCGCAAAACAGGCTAGCAAGGGCACCGTCAGCGCCCGTGTT

GGCTGGCATATCAATGTCATCGAGTCGACCCTGGCACTCGCCATCCGCGGCGTCCTCCCT

GTATGGCGCACCACACCAACTCCATCGCTCTTTAGGGACGCGGGAATCCCGTCTGGATAC

GCCACACTTGAAGAGGCGAAACTACGGTTCGCTCTAAGGCTTAACACCATCCACAAAGGT

CACACCCTTGTCCGTCGCATTCGACCCCCGATGATCACCCGAGGCCGCGGCACCGGCACC

CGCCAACCGGCAAAGACAATTATCCAGAGACTTGGGAGCATCCTCCCGGAAGTCCCAAGA

CCGACCCTCTCCCCCCCGCACTACTCACCGGGCTGCAGAATAGATCCTACAGGGGGTATA

GATAAGGCGACCGCGTCTAAAGCTTTCCAAGTCTGGCAGGAATCACTCCCACCCACAGAT

ATCTGCGTCTTCTCAGATGGCTCCGAGCAGTGGCAGGAGGGCATCAAGTACGTAGGCTAT

GGCTTCGTACTTATGGTAAACGGCACCCAAATCGACACTGGCGCTGGCGCTATCAACTCA

CGCTCTCATGTTTTCGACGCCGAAGCAATCGGAGCCTGGCGAGGGCTCGAACGGGCAATC

GCGGTAGCTCCGCCTAGGTCGAAAATCTGGCTCTGTATTGATAGCACATCCGTTATCTGG

TGTATTAGGGGGAATGCCTCTAACTCCTCGCAATGGGCATTTTTAGCCTGCCACCGGGCT

ATGGAACAGCACAATATCAGTCTCCGATGGGCCCCTGGGCACACTGGGATCGAAGGGAAC

GAAGCTGCTGACACCTTAGCCGGTGAAGGCGCGCTACGCGGTAGTGCTATAGGGATGGAA

GCCGAACCCACGATTAGCGGGATCCGATCCATCTTCCGGGAACTTCGGAACGAGGCTCGC

TTGCGCTGGTGGGACACGGTCTCTCAAAAACTCTCCCAGTGGTACCGACGCTGGTCAGAC

ACCTACGAGATTGATTCACTGCCGGAACTCGAACTCCGACGACCAGCGCTCCACCGCTGG

CTTGCCCTCCGCTCGTCGCATGGCGACTTCGACTGGTACCACCGCAAGTTCAACCACGAA

GACGCCAAACTCGACTGCTCATGCGGCCGCCGAAAGTCACCAGAGCACCTCGCTCTCTGC

CACAAAACCCAGAGGTCTTTCCGACACTGGCCAAAACGCCCCCCGACACCTCCAACCGAC

AGGACAGAGGCAGTCGCCTACCTTCGCAGCCTGGACCCCAAGCAGTTTGTTGAACTACTG

GAGCTCACAAGCTTCTACTCGCGGGTCTGCACGAGG

>CL109.Contig2_All 552 3089 minus strand putative RNA-directed DNA polymerase from transposon X-element [Penicillium digitatum PHI26]

ATGAGTGCGGTGGAAGTTTGACAAGCACCTTGGGTTGGCTATTTCCATATTCCAGAGGAA

TGTAGGTGTAATCAGTATCCTATCACCACAACCTCCAGCCTTCCATGGCTACAATCTGTC

ACAATGGAAGAAGTTGAGGCTAACACAATTGGCGTCTCCAGCACGTCGCCTGGCTCAGAT

AGGATAACTGTGCGCCTACTCAAAGCCTGCTGGGAACACCTCAAAGACATAGTCCTCTCT

CTGTTTAACCGATGCCTCGCCCTCTGCCACATCCCCCTAGCCTGGAAGGTCGCGGAGGTG

GCTATGATACCCAAGGTCGGCAAGAAGGATAAGTCCTCCGTGCGCTCCTGGAGACCGATC

GCCCTCCTATCCTGTCTCTCTAAGGGACTAGAACGAATCGTCGCGAAAAGAATTGCCTGG

ACTGCCCTCACCCACGGCGTCCTCAGCCCCCAGCATGCGGGGGCTCTACCCAAACTTTCC

GCCACAGACCTAGTCGCCTCCTTCACCCACGATGCCGAAATGGCACTCTCACAGAACAAG

CAAGTCACCCTAGTCACGATGGACGTCCAGGGTGCATTCGATGCCCTCCTCCGGAGACGA

CTTCTCAAGCGCATGGGCGAGCAGGGCTGGCCACGAGAACTGCTACTTCTTGTGGACAGC

TTTCTCACCGGACGCAAAGCCCGCGTCCGGCTAGAAGGCTCAACCACCCCTGAGTACGAC

GTGGTCTGCGGAACCCCCCAAGGCTCCCCATTATCACCAGTACTATATATGCTCTACCTA

GCCGAACTCCTCAATATGGACCAAACGCTCCGTTTCGGGTATGCCGACGACCTAGCTCTA

TACAGAGCCTCCCACGATCTCACCCAGAATGTCAGACTCCTCGCTAAGGACGTTCAGAGC

ATTCTTGCCTGGGGAGAATATAATAAAGTGGCCTTCGCCCCCGAGAAACTAGAGATGATC

CATATCACTAGACATCGAGGGGATGAGTCCCCTTCAATTGTAGTCAACGACCGGCTCACC

ATCGACCCCGTTCAGGCCAAGAAACCAGGATACACACCCACTCTCCGCTGGCTGGGAGTC

TTTTTCGATAGAAAGCTCACATGGAGAAGCCACATTCTCGCCCGGGCGGGCAAGGCACGC

GCTGTCGCACAACATATCCGCAATCTGGCCCGCACGACCTGCGGCCCGCCCGCGAGCTCA

CTTCGCAAAGCAGTCATCACCTGTGTTATACCCTCCCTAACGTTCGGAACTGAGGCCTGG

TATGGCGGCCGGAATAGGCCCGCAAAACAGGCTAGCAAGGGCACCGTCAGCGCCCGTGTT

GGCTGGCATATCAATGTCATCGAGTCGACCCTGGCACTCGCCATCCGCGGCGTCCTCCCT

GTATGGCGCACCACACCAACTCCATCGCTCTTTAGGGACGCGGGAATCCCGTCTGGATAC

GCCACACTTGAAGAGGCGAAACTACGGTTCGCTCTAAGGCTTAACACCATCCACAAAGGT

CACACCCTTGTCCGTCGCATTCGACCCCCGATGATCACCCGAGGCCGCGGCACCGGCACC

CGCCAACCGGCAAAGACAATTATCCAGAGACTTGGGAGCATCCTCCCGGAAGTCCCAAGA

CCGACCCTCTCCCCCCCGCACTACTCACCGGGCTGCAGAATAGATCCTACAGGGGGTATA

GATAAGGCGACCGCGTCTAAAGCTTTCCAAGTCTGGCAGGAATCACTCCCACCCACAGAT

ATCTGCGTCTTCTCAGATGGCTCCGAGCAGTGGCAGGAGGGCATCAAGTACGTAGGCTAT

GGCTTCGTACTTATGGTAAACGGCACCCAAATCGACACTGGCGCTGGCGCTATCAACTCA

CGCTCTCATGTTTTCGACGCCGAAGCAATCGGAGCCTGGCGAGGGCTCGAACGGGCAATC

GCGGTAGCTCCGCCTAGGTCGAAAATCTGGCTCTGTATTGATAGCACATCCGTTATCTGG

TGTATTAGGGGGAATGCCTCTAACTCCTCGCAATGGGCATTTTTAGCCTGCCACCGGGCT

ATGGAACAGCACAATATCAGTCTCCGATGGGCCCCTGGGCACACTGGGATCGAAGGGAAC

GAAGCTGCTGACACCTTAGCCGGTGAAGGCGCGCTACGCGGTAGTGGCTGGTGGGACACG

GTCTCTCAAAAACTCTCCCAGTGGTACCGACGCTGGTCAGACACCTACGAGATTGATTCA

CTGCCGGAACTCGAACTCCGACGACCAGCGCTCCACCGCTGGCTTGCCCTCCGCTCGTCG

CATGGCGACTTCGACTGGTACCACCGCAAGTTCAACCACGAAGACGCCAAACTCGACTGC

TCATGCGGCCGCCGAAAGTCACCAGAGCACCTCGCTCTCTGCCACAAAACCCAGAGGTCT

TTCCGACACTGGCCAAAACGCCCCCCGACACCTCCAACCGACAGGACAGAGGCAGTCGCC

TACCTTCGCAGCCTGGACCCCAAGCAGTTTGTTGAACTACTGGAGCTCACAAGCTTCTAC

TCGCGGGTCTGCACGAGG

>CL109.Contig3_All 1079 3436 putative RNA-directed DNA polymerase from transposon X-element [Penicillium digitatum PHI26]

AAAGTACCAGTATATTGCTGCAAAGCCTGCTGGGAACACCTCAAAGACATAGTCCTCTCT

CTGTTTAACCGATGCCTCGCCCTCTGCCACATCCCCCTAGCCTGGAAGGTCGCGGAGGTG

GCTATGATACCCAAGGTCGGCAAGAAGGATAAGTCCTCCGTGCGCTCCTGGAGACCGATC

GCCCTCCTATCCTGTCTCTCTAAGGGACTAGAACGAATCGTCGCGAAAAGAATTGCCTGG

ACTGCCCTCACCCACGGCGTCCTCAGCCCCCAGCATGCGGGGGCTCTACCCAAACTTTCC

GCCACAGACCTAGTCGCCTCCTTCACCCACGATGCCGAAATGGCACTCTCACAGAACAAG

CAAGTCACCCTAGTCACGATGGACGTCCAGGGTGCATTCGATGCCCTCCTCCGGAGACGA

CTTCTCAAGCGCATGGGCGAGCAGGGCTGGCCACGAGAACTGCTACTTCTTGTGGACAGC

TTTCTCACCGGACGCAAAGCCCGCGTCCGGCTAGAAGGCTCAACCACCCCTGAGTACGAC

GTGGTCTGCGGAACCCCCCAAGGCTCCCCATTATCACCAGTACTATATATGCTCTACCTA

GCCGAACTCCTCAATATGGACCAAACGCTCCGTTTCGGGTATGCCGACGACCTAGCTCTA

TACAGAGCCTCCCACGATCTCACCCAGAATGTCAGACTCCTCGCTAAGGACGTTCAGAGC

ATTCTTGCCTGGGGAGAATATAATAAAGTGGCCTTCGCCCCCGAGAAACTAGAGATGATC

CATATCACTAGACATCGAGGGGATGAGTCCCCTTCAATTGTAGTCAACGACCGGCTCACC

ATCGACCCCGTTCAGGCCAAGAAACCAGGATACACACCCACTCTCCGCTGGCTGGGAGTC

TTTTTCGATAGAAAGCTCACATGGAGAAGCCACATTCTCGCCCGGGCGGGCAAGGCACGC

GCTGTCGCACAACATATCCGCAATCTGGCCCGCACGACCTGCGGCCCGCCCGCGAGCTCA

CTTCGCAAAGCAGTCATCACCTGTGTTATACCCTCCCTAACGTTCGGAACTGAGGCCTGG

TATGGCGGCCGGAATAGGCCCGCAAAACAGGCTAGCAAGGGCACCGTCAGCGCCCGTGTT

GGCTGGCATATCAATGTCATCGAGTCGACCCTGGCACTCGCCATCCGCGGCGTCCTCCCT

GTATGGCGCACCACACCAACTCCATCGCTCTTTAGGGACGCGGGAATCCCGTCTGGATAC

GCCACACTTGAAGAGGCGAAACTACGGTTCGCTCTAAGGCTTAACACCATCCACAAAGGT

CACACCCTTGTCCGTCGCATTCGACCCCCGATGATCACCCGAGGCCGCGGCACCGGCACC

CGCCAACCGGCAAAGACAATTATCCAGAGACTTGGGAGCATCCTCCCGGAAGTCCCAAGA

CCGACCCTCTCCCCCCCGCACTACTCACCGGGCTGCAGAATAGATCCTACAGGGGGTATA

GATAAGGCGACCGCGTCTAAAGCTTTCCAAGTCTGGCAGGAATCACTCCCACCCACAGAT

ATCTGCGTCTTCTCAGATGGCTCCGAGCAGTGGCAGGAGGGCATCAAGTACGTAGGCTAT

GGCTTCGTACTTATGGTAAACGGCACCCAAATCGACACTGGCGCTGGCGCTATCAACTCA

CGCTCTCATGTTTTCGACGCCGAAGCAATCGGAGCCTGGCGAGGGCTCGAACGGGCAATC

GCGGTAGCTCCGCCTAGGTCGAAAATCTGGCTCTGTATTGATAGCACATCCGTTATCTGG

TGTATTAGGGGGAATGCCTCTAACTCCTCGCAATGGGCATTTTTAGCCTGCCACCGGGCT

ATGGAACAGCACAATATCAGTCTCCGATGGGCCCCTGGGCACACTGGGATCGAAGGGAAC

GAAGCTGCTGACACCTTAGCCGGTGAAGGCGCGCTACGCGGTAGTGGCTGGTGGGACACG

GTCTCTCAAAAACTCTCCCAGTGGTACCGACGCTGGTCAGACACCTACGAGATTGATTCA

CTGCCGGAACTCGAACTCCGACGACCAGCGCTCCACCGCTGGCTTGCCCTCCGCTCGTCG

CATGGCGACTTCGACTGGTACCACCGCAAGTTCAACCACGAAGACGCCAAACTCGACTGC

TCATGCGGCCGCCGAAAGTCACCAGAGCACCTCGCTCTCTGCCACAAAACCCAGAGGTCT

TTCCGACACTGGCCAAAACGCCCCCCGACACCTCCAACCGACAGGACAGAGGCAGTCGCC

TACCTTCGCAGCCTGGACCCCAAGCAGTTTGTTGAACTACTGGAGCTCACAAGCTTCTAC

TCGCGGGTCTGCACGAGG

>CL109.Contig4_All 1079 3514 putative RNA-directed DNA polymerase from transposon X-element [Penicillium digitatum PHI26]

AAAGTACCAGTATATTGCTGCAAAGCCTGCTGGGAACACCTCAAAGACATAGTCCTCTCT

CTGTTTAACCGATGCCTCGCCCTCTGCCACATCCCCCTAGCCTGGAAGGTCGCGGAGGTG

GCTATGATACCCAAGGTCGGCAAGAAGGATAAGTCCTCCGTGCGCTCCTGGAGACCGATC

GCCCTCCTATCCTGTCTCTCTAAGGGACTAGAACGAATCGTCGCGAAAAGAATTGCCTGG

ACTGCCCTCACCCACGGCGTCCTCAGCCCCCAGCATGCGGGGGCTCTACCCAAACTTTCC

GCCACAGACCTAGTCGCCTCCTTCACCCACGATGCCGAAATGGCACTCTCACAGAACAAG

CAAGTCACCCTAGTCACGATGGACGTCCAGGGTGCATTCGATGCCCTCCTCCGGAGACGA

CTTCTCAAGCGCATGGGCGAGCAGGGCTGGCCACGAGAACTGCTACTTCTTGTGGACAGC

TTTCTCACCGGACGCAAAGCCCGCGTCCGGCTAGAAGGCTCAACCACCCCTGAGTACGAC

GTGGTCTGCGGAACCCCCCAAGGCTCCCCATTATCACCAGTACTATATATGCTCTACCTA

GCCGAACTCCTCAATATGGACCAAACGCTCCGTTTCGGGTATGCCGACGACCTAGCTCTA

TACAGAGCCTCCCACGATCTCACCCAGAATGTCAGACTCCTCGCTAAGGACGTTCAGAGC

ATTCTTGCCTGGGGAGAATATAATAAAGTGGCCTTCGCCCCCGAGAAACTAGAGATGATC

CATATCACTAGACATCGAGGGGATGAGTCCCCTTCAATTGTAGTCAACGACCGGCTCACC

ATCGACCCCGTTCAGGCCAAGAAACCAGGATACACACCCACTCTCCGCTGGCTGGGAGTC

TTTTTCGATAGAAAGCTCACATGGAGAAGCCACATTCTCGCCCGGGCGGGCAAGGCACGC

GCTGTCGCACAACATATCCGCAATCTGGCCCGCACGACCTGCGGCCCGCCCGCGAGCTCA

CTTCGCAAAGCAGTCATCACCTGTGTTATACCCTCCCTAACGTTCGGAACTGAGGCCTGG

TATGGCGGCCGGAATAGGCCCGCAAAACAGGCTAGCAAGGGCACCGTCAGCGCCCGTGTT

GGCTGGCATATCAATGTCATCGAGTCGACCCTGGCACTCGCCATCCGCGGCGTCCTCCCT

GTATGGCGCACCACACCAACTCCATCGCTCTTTAGGGACGCGGGAATCCCGTCTGGATAC

GCCACACTTGAAGAGGCGAAACTACGGTTCGCTCTAAGGCTTAACACCATCCACAAAGGT

CACACCCTTGTCCGTCGCATTCGACCCCCGATGATCACCCGAGGCCGCGGCACCGGCACC

CGCCAACCGGCAAAGACAATTATCCAGAGACTTGGGAGCATCCTCCCGGAAGTCCCAAGA

CCGACCCTCTCCCCCCCGCACTACTCACCGGGCTGCAGAATAGATCCTACAGGGGGTATA

GATAAGGCGACCGCGTCTAAAGCTTTCCAAGTCTGGCAGGAATCACTCCCACCCACAGAT

ATCTGCGTCTTCTCAGATGGCTCCGAGCAGTGGCAGGAGGGCATCAAGTACGTAGGCTAT

GGCTTCGTACTTATGGTAAACGGCACCCAAATCGACACTGGCGCTGGCGCTATCAACTCA

CGCTCTCATGTTTTCGACGCCGAAGCAATCGGAGCCTGGCGAGGGCTCGAACGGGCAATC

GCGGTAGCTCCGCCTAGGTCGAAAATCTGGCTCTGTATTGATAGCACATCCGTTATCTGG

TGTATTAGGGGGAATGCCTCTAACTCCTCGCAATGGGCATTTTTAGCCTGCCACCGGGCT

ATGGAACAGCACAATATCAGTCTCCGATGGGCCCCTGGGCACACTGGGATCGAAGGGAAC

GAAGCTGCTGACACCTTAGCCGGTGAAGGCGCGCTACGCGGTAGTGCTATAGGGATGGAA

GCCGAACCCACGATTAGCGGGATCCGATCCATCTTCCGGGAACTTCGGAACGAGGCTCGC

TTGCGCTGGTGGGACACGGTCTCTCAAAAACTCTCCCAGTGGTACCGACGCTGGTCAGAC

ACCTACGAGATTGATTCACTGCCGGAACTCGAACTCCGACGACCAGCGCTCCACCGCTGG

CTTGCCCTCCGCTCGTCGCATGGCGACTTCGACTGGTACCACCGCAAGTTCAACCACGAA

GACGCCAAACTCGACTGCTCATGCGGCCGCCGAAAGTCACCAGAGCACCTCGCTCTCTGC

CACAAAACCCAGAGGTCTTTCCGACACTGGCCAAAACGCCCCCCGACACCTCCAACCGAC

AGGACAGAGGCAGTCGCCTACCTTCGCAGCCTGGACCCCAAGCAGTTTGTTGAACTACTG

GAGCTCACAAGCTTCTACTCGCGGGTCTGCACGAGG

>CL109.Contig5_All 1079 3514 putative RNA-directed DNA polymerase from transposon X-element [Penicillium digitatum PHI26]

AAAGTACCAGTATATTGCTGCAAAGCCTGCTGGGAACACCTCAAAGACATAGTCCTCTCT

CTGTTTAACCGATGCCTCGCCCTCTGCCACATCCCCCTAGCCTGGAAGGTCGCGGAGGTG

GCTATGATACCCAAGGTCGGCAAGAAGGATAAGTCCTCCGTGCGCTCCTGGAGACCGATC

GCCCTCCTATCCTGTCTCTCTAAGGGACTAGAACGAATCGTCGCGAAAAGAATTGCCTGG

ACTGCCCTCACCCACGGCGTCCTCAGCCCCCAGCATGCGGGGGCTCTACCCAAACTTTCC

GCCACAGACCTAGTCGCCTCCTTCACCCACGATGCCGAAATGGCACTCTCACAGAACAAG

CAAGTCACCCTAGTCACGATGGACGTCCAGGGTGCATTCGATGCCCTCCTCCGGAGACGA

CTTCTCAAGCGCATGGGCGAGCAGGGCTGGCCACGAGAACTGCTACTTCTTGTGGACAGC

TTTCTCACCGGACGCAAAGCCCGCGTCCGGCTAGAAGGCTCAACCACCCCTGAGTACGAC

GTGGTCTGCGGAACCCCCCAAGGCTCCCCATTATCACCAGTACTATATATGCTCTACCTA

GCCGAACTCCTCAATATGGACCAAACGCTCCGTTTCGGGTATGCCGACGACCTAGCTCTA

TACAGAGCCTCCCACGATCTCACCCAGAATGTCAGACTCCTCGCTAAGGACGTTCAGAGC

ATTCTTGCCTGGGGAGAATATAATAAAGTGGCCTTCGCCCCCGAGAAACTAGAGATGATC

CATATCACTAGACATCGAGGGGATGAGTCCCCTTCAATTGTAGTCAACGACCGGCTCACC

ATCGACCCCGTTCAGGCCAAGAAACCAGGATACACACCCACTCTCCGCTGGCTGGGAGTC

TTTTTCGATAGAAAGCTCACATGGAGAAGCCACATTCTCGCCCGGGCGGGCAAGGCACGC

GCTGTCGCACAACATATCCGCAATCTGGCCCGCACGACCTGCGGCCCGCCCGCGAGCTCA

CTTCGCAAAGCAGTCATCACCTGTGTTATACCCTCCCTAACGTTCGGAACTGAGGCCTGG

TATGGCGGCCGGAATAGGCCCGCAAAACAGGCTAGCAAGGGCACCGTCAGCGCCCGTGTT

GGCTGGCATATCAATGTCATCGAGTCGACCCTGGCACTCGCCATCCGCGGCGTCCTCCCT

GTATGGCGCACCACACCAACTCCATCGCTCTTTAGGGACGCGGGAATCCCGTCTGGATAC

GCCACACTTGAAGAGGCGAAACTACGGTTCGCTCTAAGGCTTAACACCATCCACAAAGGT

CACACCCTTGTCCGTCGCATTCGACCCCCGATGATCACCCGAGGCCGCGGCACCGGCACC

CGCCAACCGGCAAAGACAATTATCCAGAGACTTGGGAGCATCCTCCCGGAAGTCCCAAGA

CCGACCCTCTCCCCCCCGCACTACTCACCGGGCTGCAGAATAGATCCTACAGGGGGTATA

GATAAGGCGACCGCGTCTAAAGCTTTCCAAGTCTGGCAGGAATCACTCCCACCCACAGAT

ATCTGCGTCTTCTCAGATGGCTCCGAGCAGTGGCAGGAGGGCATCAAGTACGTAGGCTAT

GGCTTCGTACTTATGGTAAACGGCACCCAAATCGACACTGGCGCTGGCGCTATCAACTCA

CGCTCTCATGTTTTCGACGCCGAAGCAATCGGAGCCTGGCGAGGGCTCGAACGGGCAATC

GCGGTAGCTCCGCCTAGGTCGAAAATCTGGCTCTGTATTGATAGCACATCCGTTATCTGG

TGTATTAGGGGGAATGCCTCTAACTCCTCGCAATGGGCATTTTTAGCCTGCCACCGGGCT

ATGGAACAGCACAATATCAGTCTCCGATGGGCCCCTGGGCACACTGGGATCGAAGGGAAC

GAAGCTGCTGACACCTTAGCCGGTGAAGGCGCGCTACGCGGTAGTGCTATAGGGATGGAA

GCCGAACCCACGATTAGCGGGATCCGATCCATCTTCCGGGAACTTCGGAACGAGGCTCGC

TTGCGCTGGTGGGACACGGTCTCTCAAAAACTCTCCCAGTGGTACCGACGCTGGTCAGAC

ACCTACGAGATTGATTCACTGCCGGAACTCGAACTCCGACGACCAGCGCTCCACCGCTGG

CTTGCCCTCCGCTCGTCGCATGGCGACTTCGACTGGTACCACCGCAAGTTCAACCACGAA

GACGCCAAACTCGACTGCTCATGCGGCCGCCGAAAGTCACCAGAGCACCTCGCTCTCTGC

CACAAAACCCAGAGGTCTTTCCGACACTGGCCAAAACGCCCCCCGACACCTCCAACCGAC

AGGACAGAGGCAGTCGCCTACCTTCGCAGCCTGGACCCCAAGCAGTTTGTTGAACTACTG

GAGCTCACAAGCTTCTACTCGCGGGTCTGCACGAGG

>CL109.Contig6_All 1079 3436 putative RNA-directed DNA polymerase from transposon X-element [Penicillium digitatum PHI26]

AAAGTACCAGTATATTGCTGCAAAGCCTGCTGGGAACACCTCAAAGACATAGTCCTCTCT

CTGTTTAACCGATGCCTCGCCCTCTGCCACATCCCCCTAGCCTGGAAGGTCGCGGAGGTG

GCTATGATACCCAAGGTCGGCAAGAAGGATAAGTCCTCCGTGCGCTCCTGGAGACCGATC

GCCCTCCTATCCTGTCTCTCTAAGGGACTAGAACGAATCGTCGCGAAAAGAATTGCCTGG

ACTGCCCTCACCCACGGCGTCCTCAGCCCCCAGCATGCGGGGGCTCTACCCAAACTTTCC

GCCACAGACCTAGTCGCCTCCTTCACCCACGATGCCGAAATGGCACTCTCACAGAACAAG

CAAGTCACCCTAGTCACGATGGACGTCCAGGGTGCATTCGATGCCCTCCTCCGGAGACGA

CTTCTCAAGCGCATGGGCGAGCAGGGCTGGCCACGAGAACTGCTACTTCTTGTGGACAGC

TTTCTCACCGGACGCAAAGCCCGCGTCCGGCTAGAAGGCTCAACCACCCCTGAGTACGAC

GTGGTCTGCGGAACCCCCCAAGGCTCCCCATTATCACCAGTACTATATATGCTCTACCTA

GCCGAACTCCTCAATATGGACCAAACGCTCCGTTTCGGGTATGCCGACGACCTAGCTCTA

TACAGAGCCTCCCACGATCTCACCCAGAATGTCAGACTCCTCGCTAAGGACGTTCAGAGC

ATTCTTGCCTGGGGAGAATATAATAAAGTGGCCTTCGCCCCCGAGAAACTAGAGATGATC

CATATCACTAGACATCGAGGGGATGAGTCCCCTTCAATTGTAGTCAACGACCGGCTCACC

ATCGACCCCGTTCAGGCCAAGAAACCAGGATACACACCCACTCTCCGCTGGCTGGGAGTC

TTTTTCGATAGAAAGCTCACATGGAGAAGCCACATTCTCGCCCGGGCGGGCAAGGCACGC

GCTGTCGCACAACATATCCGCAATCTGGCCCGCACGACCTGCGGCCCGCCCGCGAGCTCA

CTTCGCAAAGCAGTCATCACCTGTGTTATACCCTCCCTAACGTTCGGAACTGAGGCCTGG

TATGGCGGCCGGAATAGGCCCGCAAAACAGGCTAGCAAGGGCACCGTCAGCGCCCGTGTT

GGCTGGCATATCAATGTCATCGAGTCGACCCTGGCACTCGCCATCCGCGGCGTCCTCCCT

GTATGGCGCACCACACCAACTCCATCGCTCTTTAGGGACGCGGGAATCCCGTCTGGATAC

GCCACACTTGAAGAGGCGAAACTACGGTTCGCTCTAAGGCTTAACACCATCCACAAAGGT

CACACCCTTGTCCGTCGCATTCGACCCCCGATGATCACCCGAGGCCGCGGCACCGGCACC

CGCCAACCGGCAAAGACAATTATCCAGAGACTTGGGAGCATCCTCCCGGAAGTCCCAAGA

CCGACCCTCTCCCCCCCGCACTACTCACCGGGCTGCAGAATAGATCCTACAGGGGGTATA

GATAAGGCGACCGCGTCTAAAGCTTTCCAAGTCTGGCAGGAATCACTCCCACCCACAGAT

ATCTGCGTCTTCTCAGATGGCTCCGAGCAGTGGCAGGAGGGCATCAAGTACGTAGGCTAT

GGCTTCGTACTTATGGTAAACGGCACCCAAATCGACACTGGCGCTGGCGCTATCAACTCA

CGCTCTCATGTTTTCGACGCCGAAGCAATCGGAGCCTGGCGAGGGCTCGAACGGGCAATC

GCGGTAGCTCCGCCTAGGTCGAAAATCTGGCTCTGTATTGATAGCACATCCGTTATCTGG

TGTATTAGGGGGAATGCCTCTAACTCCTCGCAATGGGCATTTTTAGCCTGCCACCGGGCT

ATGGAACAGCACAATATCAGTCTCCGATGGGCCCCTGGGCACACTGGGATCGAAGGGAAC

GAAGCTGCTGACACCTTAGCCGGTGAAGGCGCGCTACGCGGTAGTGGCTGGTGGGACACG

GTCTCTCAAAAACTCTCCCAGTGGTACCGACGCTGGTCAGACACCTACGAGATTGATTCA

CTGCCGGAACTCGAACTCCGACGACCAGCGCTCCACCGCTGGCTTGCCCTCCGCTCGTCG

CATGGCGACTTCGACTGGTACCACCGCAAGTTCAACCACGAAGACGCCAAACTCGACTGC

TCATGCGGCCGCCGAAAGTCACCAGAGCACCTCGCTCTCTGCCACAAAACCCAGAGGTCT

TTCCGACACTGGCCAAAACGCCCCCCGACACCTCCAACCGACAGGACAGAGGCAGTCGCC

TACCTTCGCAGCCTGGACCCCAAGCAGTTTGTTGAACTACTGGAGCTCACAAGCTTCTAC

TCGCGGGTCTGCACGAGG

>CL109.Contig7_All 552 3167 minus strand putative RNA-directed DNA polymerase from transposon X-element [Penicillium digitatum PHI26]

ATGAGTGCGGTGGAAGTTTGACAAGCACCTTGGGTTGGCTATTTCCATATTCCAGAGGAA

TGTAGGTGTAATCAGTATCCTATCACCACAACCTCCAGCCTTCCATGGCTACAATCTGTC

ACAATGGAAGAAGTTGAGGCTAACACAATTGGCGTCTCCAGCACGTCGCCTGGCTCAGAT

AGGATAACTGTGCGCCTACTCAAAGCCTGCTGGGAACACCTCAAAGACATAGTCCTCTCT

CTGTTTAACCGATGCCTCGCCCTCTGCCACATCCCCCTAGCCTGGAAGGTCGCGGAGGTG

GCTATGATACCCAAGGTCGGCAAGAAGGATAAGTCCTCCGTGCGCTCCTGGAGACCGATC

GCCCTCCTATCCTGTCTCTCTAAGGGACTAGAACGAATCGTCGCGAAAAGAATTGCCTGG

ACTGCCCTCACCCACGGCGTCCTCAGCCCCCAGCATGCGGGGGCTCTACCCAAACTTTCC

GCCACAGACCTAGTCGCCTCCTTCACCCACGATGCCGAAATGGCACTCTCACAGAACAAG

CAAGTCACCCTAGTCACGATGGACGTCCAGGGTGCATTCGATGCCCTCCTCCGGAGACGA

CTTCTCAAGCGCATGGGCGAGCAGGGCTGGCCACGAGAACTGCTACTTCTTGTGGACAGC

TTTCTCACCGGACGCAAAGCCCGCGTCCGGCTAGAAGGCTCAACCACCCCTGAGTACGAC

GTGGTCTGCGGAACCCCCCAAGGCTCCCCATTATCACCAGTACTATATATGCTCTACCTA

GCCGAACTCCTCAATATGGACCAAACGCTCCGTTTCGGGTATGCCGACGACCTAGCTCTA

TACAGAGCCTCCCACGATCTCACCCAGAATGTCAGACTCCTCGCTAAGGACGTTCAGAGC

ATTCTTGCCTGGGGAGAATATAATAAAGTGGCCTTCGCCCCCGAGAAACTAGAGATGATC

CATATCACTAGACATCGAGGGGATGAGTCCCCTTCAATTGTAGTCAACGACCGGCTCACC

ATCGACCCCGTTCAGGCCAAGAAACCAGGATACACACCCACTCTCCGCTGGCTGGGAGTC

TTTTTCGATAGAAAGCTCACATGGAGAAGCCACATTCTCGCCCGGGCGGGCAAGGCACGC

GCTGTCGCACAACATATCCGCAATCTGGCCCGCACGACCTGCGGCCCGCCCGCGAGCTCA

CTTCGCAAAGCAGTCATCACCTGTGTTATACCCTCCCTAACGTTCGGAACTGAGGCCTGG

TATGGCGGCCGGAATAGGCCCGCAAAACAGGCTAGCAAGGGCACCGTCAGCGCCCGTGTT

GGCTGGCATATCAATGTCATCGAGTCGACCCTGGCACTCGCCATCCGCGGCGTCCTCCCT

GTATGGCGCACCACACCAACTCCATCGCTCTTTAGGGACGCGGGAATCCCGTCTGGATAC

GCCACACTTGAAGAGGCGAAACTACGGTTCGCTCTAAGGCTTAACACCATCCACAAAGGT

CACACCCTTGTCCGTCGCATTCGACCCCCGATGATCACCCGAGGCCGCGGCACCGGCACC

CGCCAACCGGCAAAGACAATTATCCAGAGACTTGGGAGCATCCTCCCGGAAGTCCCAAGA

CCGACCCTCTCCCCCCCGCACTACTCACCGGGCTGCAGAATAGATCCTACAGGGGGTATA

GATAAGGCGACCGCGTCTAAAGCTTTCCAAGTCTGGCAGGAATCACTCCCACCCACAGAT

ATCTGCGTCTTCTCAGATGGCTCCGAGCAGTGGCAGGAGGGCATCAAGTACGTAGGCTAT

GGCTTCGTACTTATGGTAAACGGCACCCAAATCGACACTGGCGCTGGCGCTATCAACTCA

CGCTCTCATGTTTTCGACGCCGAAGCAATCGGAGCCTGGCGAGGGCTCGAACGGGCAATC

GCGGTAGCTCCGCCTAGGTCGAAAATCTGGCTCTGTATTGATAGCACATCCGTTATCTGG

TGTATTAGGGGGAATGCCTCTAACTCCTCGCAATGGGCATTTTTAGCCTGCCACCGGGCT

ATGGAACAGCACAATATCAGTCTCCGATGGGCCCCTGGGCACACTGGGATCGAAGGGAAC

GAAGCTGCTGACACCTTAGCCGGTGAAGGCGCGCTACGCGGTAGTGCTATAGGGATGGAA

GCCGAACCCACGATTAGCGGGATCCGATCCATCTTCCGGGAACTTCGGAACGAGGCTCGC

TTGCGCTGGTGGGACACGGTCTCTCAAAAACTCTCCCAGTGGTACCGACGCTGGTCAGAC

ACCTACGAGATTGATTCACTGCCGGAACTCGAACTCCGACGACCAGCGCTCCACCGCTGG

CTTGCCCTCCGCTCGTCGCATGGCGACTTCGACTGGTACCACCGCAAGTTCAACCACGAA

GACGCCAAACTCGACTGCTCATGCGGCCGCCGAAAGTCACCAGAGCACCTCGCTCTCTGC

CACAAAACCCAGAGGTCTTTCCGACACTGGCCAAAACGCCCCCCGACACCTCCAACCGAC

AGGACAGAGGCAGTCGCCTACCTTCGCAGCCTGGACCCCAAGCAGTTTGTTGAACTACTG

GAGCTCACAAGCTTCTACTCGCGGGTCTGCACGAGG

>CL109.Contig8_All 552 3167 minus strand putative RNA-directed DNA polymerase from transposon X-element [Penicillium digitatum PHI26]

ATGAGTGCGGTGGAAGTTTGACAAGCACCTTGGGTTGGCTATTTCCATATTCCAGAGGAA

TGTAGGTGTAATCAGTATCCTATCACCACAACCTCCAGCCTTCCATGGCTACAATCTGTC

ACAATGGAAGAAGTTGAGGCTAACACAATTGGCGTCTCCAGCACGTCGCCTGGCTCAGAT

AGGATAACTGTGCGCCTACTCAAAGCCTGCTGGGAACACCTCAAAGACATAGTCCTCTCT

CTGTTTAACCGATGCCTCGCCCTCTGCCACATCCCCCTAGCCTGGAAGGTCGCGGAGGTG

GCTATGATACCCAAGGTCGGCAAGAAGGATAAGTCCTCCGTGCGCTCCTGGAGACCGATC

GCCCTCCTATCCTGTCTCTCTAAGGGACTAGAACGAATCGTCGCGAAAAGAATTGCCTGG

ACTGCCCTCACCCACGGCGTCCTCAGCCCCCAGCATGCGGGGGCTCTACCCAAACTTTCC

GCCACAGACCTAGTCGCCTCCTTCACCCACGATGCCGAAATGGCACTCTCACAGAACAAG

CAAGTCACCCTAGTCACGATGGACGTCCAGGGTGCATTCGATGCCCTCCTCCGGAGACGA

CTTCTCAAGCGCATGGGCGAGCAGGGCTGGCCACGAGAACTGCTACTTCTTGTGGACAGC

TTTCTCACCGGACGCAAAGCCCGCGTCCGGCTAGAAGGCTCAACCACCCCTGAGTACGAC

GTGGTCTGCGGAACCCCCCAAGGCTCCCCATTATCACCAGTACTATATATGCTCTACCTA

GCCGAACTCCTCAATATGGACCAAACGCTCCGTTTCGGGTATGCCGACGACCTAGCTCTA

TACAGAGCCTCCCACGATCTCACCCAGAATGTCAGACTCCTCGCTAAGGACGTTCAGAGC

ATTCTTGCCTGGGGAGAATATAATAAAGTGGCCTTCGCCCCCGAGAAACTAGAGATGATC

CATATCACTAGACATCGAGGGGATGAGTCCCCTTCAATTGTAGTCAACGACCGGCTCACC

ATCGACCCCGTTCAGGCCAAGAAACCAGGATACACACCCACTCTCCGCTGGCTGGGAGTC

TTTTTCGATAGAAAGCTCACATGGAGAAGCCACATTCTCGCCCGGGCGGGCAAGGCACGC

GCTGTCGCACAACATATCCGCAATCTGGCCCGCACGACCTGCGGCCCGCCCGCGAGCTCA

CTTCGCAAAGCAGTCATCACCTGTGTTATACCCTCCCTAACGTTCGGAACTGAGGCCTGG

TATGGCGGCCGGAATAGGCCCGCAAAACAGGCTAGCAAGGGCACCGTCAGCGCCCGTGTT

GGCTGGCATATCAATGTCATCGAGTCGACCCTGGCACTCGCCATCCGCGGCGTCCTCCCT

GTATGGCGCACCACACCAACTCCATCGCTCTTTAGGGACGCGGGAATCCCGTCTGGATAC

GCCACACTTGAAGAGGCGAAACTACGGTTCGCTCTAAGGCTTAACACCATCCACAAAGGT

CACACCCTTGTCCGTCGCATTCGACCCCCGATGATCACCCGAGGCCGCGGCACCGGCACC

CGCCAACCGGCAAAGACAATTATCCAGAGACTTGGGAGCATCCTCCCGGAAGTCCCAAGA

CCGACCCTCTCCCCCCCGCACTACTCACCGGGCTGCAGAATAGATCCTACAGGGGGTATA

GATAAGGCGACCGCGTCTAAAGCTTTCCAAGTCTGGCAGGAATCACTCCCACCCACAGAT

ATCTGCGTCTTCTCAGATGGCTCCGAGCAGTGGCAGGAGGGCATCAAGTACGTAGGCTAT

GGCTTCGTACTTATGGTAAACGGCACCCAAATCGACACTGGCGCTGGCGCTATCAACTCA

CGCTCTCATGTTTTCGACGCCGAAGCAATCGGAGCCTGGCGAGGGCTCGAACGGGCAATC

GCGGTAGCTCCGCCTAGGTCGAAAATCTGGCTCTGTATTGATAGCACATCCGTTATCTGG

TGTATTAGGGGGAATGCCTCTAACTCCTCGCAATGGGCATTTTTAGCCTGCCACCGGGCT

ATGGAACAGCACAATATCAGTCTCCGATGGGCCCCTGGGCACACTGGGATCGAAGGGAAC

GAAGCTGCTGACACCTTAGCCGGTGAAGGCGCGCTACGCGGTAGTGCTATAGGGATGGAA

GCCGAACCCACGATTAGCGGGATCCGATCCATCTTCCGGGAACTTCGGAACGAGGCTCGC

TTGCGCTGGTGGGACACGGTCTCTCAAAAACTCTCCCAGTGGTACCGACGCTGGTCAGAC

ACCTACGAGATTGATTCACTGCCGGAACTCGAACTCCGACGACCAGCGCTCCACCGCTGG

CTTGCCCTCCGCTCGTCGCATGGCGACTTCGACTGGTACCACCGCAAGTTCAACCACGAA

GACGCCAAACTCGACTGCTCATGCGGCCGCCGAAAGTCACCAGAGCACCTCGCTCTCTGC

CACAAAACCCAGAGGTCTTTCCGACACTGGCCAAAACGCCCCCCGACACCTCCAACCGAC

AGGACAGAGGCAGTCGCCTACCTTCGCAGCCTGGACCCCAAGCAGTTTGTTGAACTACTG

GAGCTCACAAGCTTCTACTCGCGGGTCTGCACGAGG

>CL109.Contig9_All 552 3167 minus strand putative RNA-directed DNA polymerase from transposon X-element [Penicillium digitatum PHI26]

ATGAGTGCGGTGGAAGTTTGACAAGCACCTTGGGTTGGCTATTTCCATATTCCAGAGGAA

TGTAGGTGTAATCAGTATCCTATCACCACAACCTCCAGCCTTCCATGGCTACAATCTGTC

ACAATGGAAGAAGTTGAGGCTAACACAATTGGCGTCTCCAGCACGTCGCCTGGCTCAGAT

AGGATAACTGTGCGCCTACTCAAAGCCTGCTGGGAACACCTCAAAGACATAGTCCTCTCT

CTGTTTAACCGATGCCTCGCCCTCTGCCACATCCCCCTAGCCTGGAAGGTCGCGGAGGTG

GCTATGATACCCAAGGTCGGCAAGAAGGATAAGTCCTCCGTGCGCTCCTGGAGACCGATC

GCCCTCCTATCCTGTCTCTCTAAGGGACTAGAACGAATCGTCGCGAAAAGAATTGCCTGG

ACTGCCCTCACCCACGGCGTCCTCAGCCCCCAGCATGCGGGGGCTCTACCCAAACTTTCC

GCCACAGACCTAGTCGCCTCCTTCACCCACGATGCCGAAATGGCACTCTCACAGAACAAG

CAAGTCACCCTAGTCACGATGGACGTCCAGGGTGCATTCGATGCCCTCCTCCGGAGACGA

CTTCTCAAGCGCATGGGCGAGCAGGGCTGGCCACGAGAACTGCTACTTCTTGTGGACAGC

TTTCTCACCGGACGCAAAGCCCGCGTCCGGCTAGAAGGCTCAACCACCCCTGAGTACGAC

GTGGTCTGCGGAACCCCCCAAGGCTCCCCATTATCACCAGTACTATATATGCTCTACCTA

GCCGAACTCCTCAATATGGACCAAACGCTCCGTTTCGGGTATGCCGACGACCTAGCTCTA

TACAGAGCCTCCCACGATCTCACCCAGAATGTCAGACTCCTCGCTAAGGACGTTCAGAGC

ATTCTTGCCTGGGGAGAATATAATAAAGTGGCCTTCGCCCCCGAGAAACTAGAGATGATC

CATATCACTAGACATCGAGGGGATGAGTCCCCTTCAATTGTAGTCAACGACCGGCTCACC

ATCGACCCCGTTCAGGCCAAGAAACCAGGATACACACCCACTCTCCGCTGGCTGGGAGTC

TTTTTCGATAGAAAGCTCACATGGAGAAGCCACATTCTCGCCCGGGCGGGCAAGGCACGC

GCTGTCGCACAACATATCCGCAATCTGGCCCGCACGACCTGCGGCCCGCCCGCGAGCTCA

CTTCGCAAAGCAGTCATCACCTGTGTTATACCCTCCCTAACGTTCGGAACTGAGGCCTGG

TATGGCGGCCGGAATAGGCCCGCAAAACAGGCTAGCAAGGGCACCGTCAGCGCCCGTGTT

GGCTGGCATATCAATGTCATCGAGTCGACCCTGGCACTCGCCATCCGCGGCGTCCTCCCT

GTATGGCGCACCACACCAACTCCATCGCTCTTTAGGGACGCGGGAATCCCGTCTGGATAC

GCCACACTTGAAGAGGCGAAACTACGGTTCGCTCTAAGGCTTAACACCATCCACAAAGGT

CACACCCTTGTCCGTCGCATTCGACCCCCGATGATCACCCGAGGCCGCGGCACCGGCACC

CGCCAACCGGCAAAGACAATTATCCAGAGACTTGGGAGCATCCTCCCGGAAGTCCCAAGA

CCGACCCTCTCCCCCCCGCACTACTCACCGGGCTGCAGAATAGATCCTACAGGGGGTATA

GATAAGGCGACCGCGTCTAAAGCTTTCCAAGTCTGGCAGGAATCACTCCCACCCACAGAT

ATCTGCGTCTTCTCAGATGGCTCCGAGCAGTGGCAGGAGGGCATCAAGTACGTAGGCTAT

GGCTTCGTACTTATGGTAAACGGCACCCAAATCGACACTGGCGCTGGCGCTATCAACTCA

CGCTCTCATGTTTTCGACGCCGAAGCAATCGGAGCCTGGCGAGGGCTCGAACGGGCAATC

GCGGTAGCTCCGCCTAGGTCGAAAATCTGGCTCTGTATTGATAGCACATCCGTTATCTGG

TGTATTAGGGGGAATGCCTCTAACTCCTCGCAATGGGCATTTTTAGCCTGCCACCGGGCT

ATGGAACAGCACAATATCAGTCTCCGATGGGCCCCTGGGCACACTGGGATCGAAGGGAAC

GAAGCTGCTGACACCTTAGCCGGTGAAGGCGCGCTACGCGGTAGTGCTATAGGGATGGAA

GCCGAACCCACGATTAGCGGGATCCGATCCATCTTCCGGGAACTTCGGAACGAGGCTCGC

TTGCGCTGGTGGGACACGGTCTCTCAAAAACTCTCCCAGTGGTACCGACGCTGGTCAGAC

ACCTACGAGATTGATTCACTGCCGGAACTCGAACTCCGACGACCAGCGCTCCACCGCTGG

CTTGCCCTCCGCTCGTCGCATGGCGACTTCGACTGGTACCACCGCAAGTTCAACCACGAA

GACGCCAAACTCGACTGCTCATGCGGCCGCCGAAAGTCACCAGAGCACCTCGCTCTCTGC

CACAAAACCCAGAGGTCTTTCCGACACTGGCCAAAACGCCCCCCGACACCTCCAACCGAC

AGGACAGAGGCAGTCGCCTACCTTCGCAGCCTGGACCCCAAGCAGTTTGTTGAACTACTG

GAGCTCACAAGCTTCTACTCGCGGGTCTGCACGAGG

>CL109.Contig10_All 1083 3518 putative RNA-directed DNA polymerase from transposon X-element [Penicillium digitatum PHI26]

AAAGTACCAGTATATTGCTGCAAAGCCTGCTGGGAACACCTCAAAGACATAGTCCTCTCT

CTGTTTAACCGATGCCTCGCCCTCTGCCACATCCCCCTAGCCTGGAAGGTCGCGGAGGTG

GCTATGATACCCAAGGTCGGCAAGAAGGATAAGTCCTCCGTGCGCTCCTGGAGACCGATC

GCCCTCCTATCCTGTCTCTCTAAGGGACTAGAACGAATCGTCGCGAAAAGAATTGCCTGG

ACTGCCCTCACCCACGGCGTCCTCAGCCCCCAGCATGCGGGGGCTCTACCCAAACTTTCC

GCCACAGACCTAGTCGCCTCCTTCACCCACGATGCCGAAATGGCACTCTCACAGAACAAG

CAAGTCACCCTAGTCACGATGGACGTCCAGGGTGCATTCGATGCCCTCCTCCGGAGACGA

CTTCTCAAGCGCATGGGCGAGCAGGGCTGGCCACGAGAACTGCTACTTCTTGTGGACAGC

TTTCTCACCGGACGCAAAGCCCGCGTCCGGCTAGAAGGCTCAACCACCCCTGAGTACGAC

GTGGTCTGCGGAACCCCCCAAGGCTCCCCATTATCACCAGTACTATATATGCTCTACCTA

GCCGAACTCCTCAATATGGACCAAACGCTCCGTTTCGGGTATGCCGACGACCTAGCTCTA

TACAGAGCCTCCCACGATCTCACCCAGAATGTCAGACTCCTCGCTAAGGACGTTCAGAGC

ATTCTTGCCTGGGGAGAATATAATAAAGTGGCCTTCGCCCCCGAGAAACTAGAGATGATC

CATATCACTAGACATCGAGGGGATGAGTCCCCTTCAATTGTAGTCAACGACCGGCTCACC

ATCGACCCCGTTCAGGCCAAGAAACCAGGATACACACCCACTCTCCGCTGGCTGGGAGTC

TTTTTCGATAGAAAGCTCACATGGAGAAGCCACATTCTCGCCCGGGCGGGCAAGGCACGC

GCTGTCGCACAACATATCCGCAATCTGGCCCGCACGACCTGCGGCCCGCCCGCGAGCTCA

CTTCGCAAAGCAGTCATCACCTGTGTTATACCCTCCCTAACGTTCGGAACTGAGGCCTGG

TATGGCGGCCGGAATAGGCCCGCAAAACAGGCTAGCAAGGGCACCGTCAGCGCCCGTGTT

GGCTGGCATATCAATGTCATCGAGTCGACCCTGGCACTCGCCATCCGCGGCGTCCTCCCT

GTATGGCGCACCACACCAACTCCATCGCTCTTTAGGGACGCGGGAATCCCGTCTGGATAC

GCCACACTTGAAGAGGCGAAACTACGGTTCGCTCTAAGGCTTAACACCATCCACAAAGGT

CACACCCTTGTCCGTCGCATTCGACCCCCGATGATCACCCGAGGCCGCGGCACCGGCACC

CGCCAACCGGCAAAGACAATTATCCAGAGACTTGGGAGCATCCTCCCGGAAGTCCCAAGA

CCGACCCTCTCCCCCCCGCACTACTCACCGGGCTGCAGAATAGATCCTACAGGGGGTATA

GATAAGGCGACCGCGTCTAAAGCTTTCCAAGTCTGGCAGGAATCACTCCCACCCACAGAT

ATCTGCGTCTTCTCAGATGGCTCCGAGCAGTGGCAGGAGGGCATCAAGTACGTAGGCTAT

GGCTTCGTACTTATGGTAAACGGCACCCAAATCGACACTGGCGCTGGCGCTATCAACTCA

CGCTCTCATGTTTTCGACGCCGAAGCAATCGGAGCCTGGCGAGGGCTCGAACGGGCAATC

GCGGTAGCTCCGCCTAGGTCGAAAATCTGGCTCTGTATTGATAGCACATCCGTTATCTGG

TGTATTAGGGGGAATGCCTCTAACTCCTCGCAATGGGCATTTTTAGCCTGCCACCGGGCT

ATGGAACAGCACAATATCAGTCTCCGATGGGCCCCTGGGCACACTGGGATCGAAGGGAAC

GAAGCTGCTGACACCTTAGCCGGTGAAGGCGCGCTACGCGGTAGTGCTATAGGGATGGAA

GCCGAACCCACGATTAGCGGGATCCGATCCATCTTCCGGGAACTTCGGAACGAGGCTCGC

TTGCGCTGGTGGGACACGGTCTCTCAAAAACTCTCCCAGTGGTACCGACGCTGGTCAGAC

ACCTACGAGATTGATTCACTGCCGGAACTCGAACTCCGACGACCAGCGCTCCACCGCTGG

CTTGCCCTCCGCTCGTCGCATGGCGACTTCGACTGGTACCACCGCAAGTTCAACCACGAA

GACGCCAAACTCGACTGCTCATGCGGCCGCCGAAAGTCACCAGAGCACCTCGCTCTCTGC

CACAAAACCCAGAGGTCTTTCCGACACTGGCCAAAACGCCCCCCGACACCTCCAACCGAC

AGGACAGAGGCAGTCGCCTACCTTCGCAGCCTGGACCCCAAGCAGTTTGTTGAACTACTG

GAGCTCACAAGCTTCTACTCGCGGGTCTGCACGAGG

>CL109.Contig11_All 1079 3436 putative RNA-directed DNA polymerase from transposon X-element [Penicillium digitatum PHI26]

AAAGTACCAGTATATTGCTGCAAAGCCTGCTGGGAACACCTCAAAGACATAGTCCTCTCT

CTGTTTAACCGATGCCTCGCCCTCTGCCACATCCCCCTAGCCTGGAAGGTCGCGGAGGTG

GCTATGATACCCAAGGTCGGCAAGAAGGATAAGTCCTCCGTGCGCTCCTGGAGACCGATC

GCCCTCCTATCCTGTCTCTCTAAGGGACTAGAACGAATCGTCGCGAAAAGAATTGCCTGG

ACTGCCCTCACCCACGGCGTCCTCAGCCCCCAGCATGCGGGGGCTCTACCCAAACTTTCC

GCCACAGACCTAGTCGCCTCCTTCACCCACGATGCCGAAATGGCACTCTCACAGAACAAG

CAAGTCACCCTAGTCACGATGGACGTCCAGGGTGCATTCGATGCCCTCCTCCGGAGACGA

CTTCTCAAGCGCATGGGCGAGCAGGGCTGGCCACGAGAACTGCTACTTCTTGTGGACAGC

TTTCTCACCGGACGCAAAGCCCGCGTCCGGCTAGAAGGCTCAACCACCCCTGAGTACGAC

GTGGTCTGCGGAACCCCCCAAGGCTCCCCATTATCACCAGTACTATATATGCTCTACCTA

GCCGAACTCCTCAATATGGACCAAACGCTCCGTTTCGGGTATGCCGACGACCTAGCTCTA

TACAGAGCCTCCCACGATCTCACCCAGAATGTCAGACTCCTCGCTAAGGACGTTCAGAGC

ATTCTTGCCTGGGGAGAATATAATAAAGTGGCCTTCGCCCCCGAGAAACTAGAGATGATC

CATATCACTAGACATCGAGGGGATGAGTCCCCTTCAATTGTAGTCAACGACCGGCTCACC

ATCGACCCCGTTCAGGCCAAGAAACCAGGATACACACCCACTCTCCGCTGGCTGGGAGTC

TTTTTCGATAGAAAGCTCACATGGAGAAGCCACATTCTCGCCCGGGCGGGCAAGGCACGC

GCTGTCGCACAACATATCCGCAATCTGGCCCGCACGACCTGCGGCCCGCCCGCGAGCTCA

CTTCGCAAAGCAGTCATCACCTGTGTTATACCCTCCCTAACGTTCGGAACTGAGGCCTGG

TATGGCGGCCGGAATAGGCCCGCAAAACAGGCTAGCAAGGGCACCGTCAGCGCCCGTGTT

GGCTGGCATATCAATGTCATCGAGTCGACCCTGGCACTCGCCATCCGCGGCGTCCTCCCT

GTATGGCGCACCACACCAACTCCATCGCTCTTTAGGGACGCGGGAATCCCGTCTGGATAC

GCCACACTTGAAGAGGCGAAACTACGGTTCGCTCTAAGGCTTAACACCATCCACAAAGGT

CACACCCTTGTCCGTCGCATTCGACCCCCGATGATCACCCGAGGCCGCGGCACCGGCACC

CGCCAACCGGCAAAGACAATTATCCAGAGACTTGGGAGCATCCTCCCGGAAGTCCCAAGA

CCGACCCTCTCCCCCCCGCACTACTCACCGGGCTGCAGAATAGATCCTACAGGGGGTATA

GATAAGGCGACCGCGTCTAAAGCTTTCCAAGTCTGGCAGGAATCACTCCCACCCACAGAT

ATCTGCGTCTTCTCAGATGGCTCCGAGCAGTGGCAGGAGGGCATCAAGTACGTAGGCTAT

GGCTTCGTACTTATGGTAAACGGCACCCAAATCGACACTGGCGCTGGCGCTATCAACTCA

CGCTCTCATGTTTTCGACGCCGAAGCAATCGGAGCCTGGCGAGGGCTCGAACGGGCAATC

GCGGTAGCTCCGCCTAGGTCGAAAATCTGGCTCTGTATTGATAGCACATCCGTTATCTGG

TGTATTAGGGGGAATGCCTCTAACTCCTCGCAATGGGCATTTTTAGCCTGCCACCGGGCT

ATGGAACAGCACAATATCAGTCTCCGATGGGCCCCTGGGCACACTGGGATCGAAGGGAAC

GAAGCTGCTGACACCTTAGCCGGTGAAGGCGCGCTACGCGGTAGTGGCTGGTGGGACACG

GTCTCTCAAAAACTCTCCCAGTGGTACCGACGCTGGTCAGACACCTACGAGATTGATTCA

CTGCCGGAACTCGAACTCCGACGACCAGCGCTCCACCGCTGGCTTGCCCTCCGCTCGTCG

CATGGCGACTTCGACTGGTACCACCGCAAGTTCAACCACGAAGACGCCAAACTCGACTGC

TCATGCGGCCGCCGAAAGTCACCAGAGCACCTCGCTCTCTGCCACAAAACCCAGAGGTCT

TTCCGACACTGGCCAAAACGCCCCCCGACACCTCCAACCGACAGGACAGAGGCAGTCGCC

TACCTTCGCAGCCTGGACCCCAAGCAGTTTGTTGAACTACTGGAGCTCACAAGCTTCTAC

TCGCGGGTCTGCACGAGG

>CL109.Contig12_All 43 2499 minus strand putative RNA-directed DNA polymerase from transposon X-element [Penicillium digitatum PHI26]

CTCACCACAACCTCCAGCCTTCCATGGCTACAATCTGTCACAATGGAAGAAGTTGAGGCT

AACACAATTGGCGTCTCCAGCACGTCGCCTGGCTCAGATAGGATAACTGTGCGCCTACTC

AAAGCCTGCTGGGAACACCTCAAAGACATAGTCCTCTCTCTGTTTAACCGATGCCTCGCC

CTCTGCCACATCCCCCTGGCCTGGAAGGTCGCGGAGGTGGCTATGATACCCAAGGTCGGC

AAGAAGGATAAGTCCTCCGTGCGCTCCTGGAGACCGATCGCCCTCCTATCCTGTCTCTCT

AAGGGACTAGAACGAATCATCGCGAAAAGAATTGCCTGGACTGCCCTCACCCACGGCGTC

CTCAGCCCCCAGCATGCGGGGGCTCTACCCAAACTTTCCGCCACAGACCTAGTCGCCTCC

TTCACCCACGATGCCGAAATGGCACTCTCACAGAACAAGCAAGTCACCCTAGTCACGATG

GACGTCCAGGGTGCATTCGATGCCCTCCTCCGGAGACGACTTCTCAAGCGCATGGGCGAG

CAGGGCTGGCCACGAGAACTGCTACTTCTTGTGGACAGCTTTCTCACCGGACGCAAAGCC

CGCGTCCGGCTAGAAGGCTCAACCACCCCTGAGTACGACGTGGTCTGCGGAACCCCCCAA

GGCTCCCCATTATCACCAGTACTATATATGCTCTACCTAGCCGAACTCCTCAATATGGAC

CAAACGCTCCGTTTCGGGTATGCCGACGACCTAGCTCTATACCGAGCCTCCCACGATCTC

ACCCAGAATGTCAGACTCCTCGCTAAGGACGTTCAGAGCATTCTTGCCTGGGGAGAATAT

AATAAAGTGGCCTTCGCCCCCGAGAAACTAGAGATGATCCATATCACTAGACATCGAGGG

GATGAGTCCCCTTCAATTGTAGTCAACGACCGGCTCACCATCGACCCCGTTCAGGCCAAG

AAACCAGGATACACACCCACTCTCCGCTGGCTGGGAGTCTTTTTCGATAGAAAGCTCACA

TGGAGAAGCCACATTCTCGCCCGGGCGGGCAAGGCACGCGCTGTCGCACAACATATCCGC

AATCTGGCCCGCACGACCTGCGGCCCGCCCGCGAGCTCACTTCGCAAAGCAGTCATCACC

TGTGTTATACCCTCCCTAACGTTCGGAACTGAGGCCTGGTATGGCGGCCGGAATAGGCCC

GCAAAACAGGCTAGCAAGGGCACCGTCAGCGCCCGTGTTGGCTGGCATATCAATGTCATC

GAGTCGACCCTGGCACTCGCCATCCGCGGCGTCCTCCCTGTATGGCGCACCACACCAACT

CCCTCGCTCTTTAGGGATGCGGGAATCCCGTCTGGATACGCCACACTTGAAGAGGCGAAA

CTACGGTTCGCTCTAAGGCTTAACACCATCCACAAAGGTCACACCCTTGTCCGTCGCATT

CGACCCCCGATGATCACCCGAGGCCGCGGCACCGGCACCCGCCAACCGGCAAAGACAATT

ATCCAGAGACTTGGGAGCATCCTCCCGGAAGTCCCAAGACCGACCCTCTCCCCCCCGCAC

TACTCACCGGGCTGCAGAATAGATCCTACAGGGGGTATAGATAAGGCGACCGCGTCTAAA

GCTTTCCAAGTCTGGCAGGAATCACTCCCACCCACAGATATCTGCGTCTTCTCAGATGGC

TCCGAGCAGTGGCAGGAGGGCATCAAGTACGTAGGCTATGGCTTCGTACTTATGGTAAAC

GGCACCCAAATCGACACTGGCGCTGGCGCTATCAACTCACGCTCTCATGTTTTCGACGCC

GAAGCAATCGGAGCCTGGCGAGGGCTCGAACGGGCAATCGCGGTAGCTCCGCCTAGGTCG

AAAATCTGGCTCTGTATTGATAGCACATCCGTTATCTGGTGTATTAGGGGGAATGCCTCG

AACTCCTCGCAATGGGCATTTTTAGCCTGCCACCGGGCTATGGAACAGCACAATATCAGT

CTCCGATGGGCCCCTGGGCACACTGGGATCGAAGGGAACGAAGCTGCTGACACCTTAGCC

GGTGAAGGCGCGCTACGCGGTAGTGGCTGGTGGGACACGGTCTCTCAAAAACTCTCCCAG

TGGTACCGACGCTGGTCAGACACCTACGAGATTGATTCACTGCCGGAACTCGAACTCCGA

CGACCAGCGCTCCACCGCTGGCTTGCCCTCCGCTCGTCGCATGGCGACTTCGACTGGTAC

CACCGCAAGTTCAACCACGAAGACGCCAAACTCGACTGCTCATGCGGCCGCCGAAAGTCA

CCAGAGCACCTCGCTCTCTGCCACAAAACCCAGAGGTCTTTCCGACACTGGCCAAAACGC

CCCCCGACACCTCCAACCGACAGGACAGAGGCAGTCGCCTACCTTCGCAGCCTGGACCCC

AAGCAGTTTGTTGAACTACTGGAGCTCACAAGCTTCTACTCGCGGGTCTGCACGAGG

>CL109.Contig13_All 5 3247 minus strand putative RNA-directed DNA polymerase from transposon X-element [Penicillium digitatum PHI26]

AGGAACGAAGCCTTCGAACCCGGCGTCGCTAACGCTAACCGCGGAGGCGAAATTGCACAG

TGGTCAAGCGACAGCGGCCTTGATTTCATCGGAGAACCAGGAGTGCCCACTCACCAGGCG

GGACACGTCCTCGATCTCACTTTCTCCAACATACCCTACGCCTCGACAGTCGTCAGGGAA

GACCTTGCCACCGGGTCTGACCATGAGTCCCTCGTCACTCGCATCCCGGGTCGCGGCAGG

GTCCCCCTCGAACAATACAACTATAGAGTTCCCGAGTCTAAGCTACCAAAACTGTCTTCC

CTCATCGGGACTGGGATCCGCTCCCTACCGGACCCAAGCAGCATCGAGACCCATGATCAA

CTAGACCAGTTCGCGGCGACTCTCACAGCACTTTTCCAGGACGCTATAAAGACAGCCGGG

TCCCTGAACCGCACTCATACCTTCACCACCCCGTGGTGGACCTCCGAATGCCAAGCCAAG

CGCCAACAGTGGCTAGGCGTAAGACACACGGATCCAGATAAGGCTGACACCGCTAAAAGA

GCTTTTCTCTCCTGTGTACGCTCCTCGAAAAGGGCCTACTGGAGGGACCGCATTGACAAT

ATCAAAACGGACTCTGACCTATACAATATCATCAGCTGGCATAAATTGGGCACCGACCTT

AAGGCCCCTCCCCTTCTCGTCGATGGCCTCCCTGTGGAGGACACCATGGAAAAGGCGGAG

GCCCTACGCCGCGCAGTCCTTGGCCGTTTTAGCCCAGACGACGACCTACCAACGGACCCT

ATCCCTATCACCACAACCTCCAGCCTTCCATGGCTACAATCTGTCACAATGGAAGAAGTT

GAGGCTAACACAATTGGCGTCTCCAGCACGTCGCCTGGCTCAGATAGGATAACTGTGCGC

CTACTCAAAGCCTGCTGGGAACACCTCAAAGACATAGTCCTCTCTCTGTTTAACCGATGC

CTCGCCCTCTGCCACATCCCCCTGGCCTGGAAGGTCGCGGAGGTGGCTATGATACCCAAG

GTCGGCAAGAAGGATAAGTCCTCCGTGCGCTCCTGGAGACCGATCGCCCTCCTATCCTGT

CTCTCTAAGGGACTAGAACGAATCATCGCGAAAAGAATTGCCTGGACTGCCCTCACCCAC

GGCGTCCTCAGCCCCCAGCATGCGGGGGCTCTACCCAAACTTTCCGCCACAGACCTAGTC

GCCTCCTTCACCCACGATGCCGAAATGGCACTCTCACAGAACAAGCAAGTCACCCTAGTC

ACGATGGACGTCCAGGGTGCATTCGATGCCCTCCTCCGGAGACGACTTCTCAAGCGCATG

GGCGAGCAGGGCTGGCCACGAGAACTGCTACTTCTTGTGGACAGCTTTCTCACCGGACGC

AAAGCCCGCGTCCGGCTAGAAGGCTCAACCACCCCTGAGTACGACGTGGTCTGCGGAACC

CCCCAAGGCTCCCCATTATCACCAGTACTATATATGCTCTACCTAGCCGAACTCCTCAAT

ATGGACCAAACGCTCCGTTTCGGGTATGCCGACGACCTAGCTCTATACCGAGCCTCCCAC

GATCTCACCCAGAATGTCAGACTCCTCGCTAAGGACGTTCAGAGCATTCTTGCCTGGGGA

GAATATAATAAAGTGGCCTTCGCCCCCGAGAAACTAGAGATGATCCATATCACTAGACAT

CGAGGGGATGAGTCCCCTTCAATTGTAGTCAACGACCGGCTCACCATCGACCCCGTTCAG

GCCAAGAAACCAGGATACACACCCACTCTCCGCTGGCTGGGAGTCTTTTTCGATAGAAAG

CTCACATGGAGAAGCCACATTCTCGCCCGGGCGGGCAAGGCACGCGCTGTCGCACAACAT

ATCCGCAATCTGGCCCGCACGACCTGCGGCCCGCCCGCGAGCTCACTTCGCAAAGCAGTC

ATCACCTGTGTTATACCCTCCCTAACGTTCGGAACTGAGGCCTGGTATGGCGGCCGGAAT

AGGCCCGCAAAACAGGCTAGCAAGGGCACCGTCAGCGCCCGTGTTGGCTGGCATATCAAT

GTCATCGAGTCGACCCTGGCACTCGCCATCCGCGGCGTCCTCCCTGTATGGCGCACCACA

CCAACTCCCTCGCTCTTTAGGGATGCGGGAATCCCGTCTGGATACGCCACACTTGAAGAG

GCGAAACTACGGTTCGCTCTAAGGCTTAACACCATCCACAAAGGTCACACCCTTGTCCGT

CGCATTCGACCCCCGATGATCACCCGAGGCCGCGGCACCGGCACCCGCCAACCGGCAAAG

ACAATTATCCAGAGACTTGGGAGCATCCTCCCGGAAGTCCCAAGACCGACCCTCTCCCCC

CCGCACTACTCACCGGGCTGCAGAATAGATCCTACAGGGGGTATAGATAAGGCGACCGCG

TCTAAAGCTTTCCAAGTCTGGCAGGAATCACTCCCACCCACAGATATCTGCGTCTTCTCA

GATGGCTCCGAGCAGTGGCAGGAGGGCATCAAGTACGTAGGCTATGGCTTCGTACTTATG

GTAAACGGCACCCAAATCGACACTGGCGCTGGCGCTATCAACTCACGCTCTCATGTTTTC

GACGCCGAAGCAATCGGAGCCTGGCGAGGGCTCGAACGGGCAATCGCGGTAGCTCCGCCT

AGGTCGAAAATCTGGCTCTGTATTGATAGCACATCCGTTATCTGGTGTATTAGGGGGAAT

GCCTCGAACTCCTCGCAATGGGCATTTTTAGCCTGCCACCGGGCTATGGAACAGCACAAT

ATCAGTCTCCGATGGGCCCCTGGGCACACTGGGATCGAAGGGAACGAAGCTGCTGACACC

TTAGCCGGTGAAGGCGCGCTACGCGGTAGTGGCTGGTGGGACACGGTCTCTCAAAAACTC

TCCCAGTGGTACCGACGCTGGTCAGACACCTACGAGATTGATTCACTGCCGGAACTCGAA

CTCCGACGACCAGCGCTCCACCGCTGGCTTGCCCTCCGCTCGTCGCATGGCGACTTCGAC

TGGTACCACCGCAAGTTCAACCACGAAGACGCCAAACTCGACTGCTCATGCGGCCGCCGA

AAGTCACCAGAGCACCTCGCTCTCTGCCACAAAACCCAGAGGTCTTTCCGACACTGGCCA

AAACGCCCCCCGACACCTCCAACCGACAGGACAGAGGCAGTCGCCTACCTTCGCAGCCTG

GACCCCAAGCAGTTTGTTGAACTACTGGAGCTCACAAGCTTCTACTCGCGGGTCTGCACG

AGG

>CL109.Contig14_All 5 3247 minus strand putative RNA-directed DNA polymerase from transposon X-element [Penicillium digitatum PHI26]

AGGAACGAAGCCTTCGAACCCGGCGTCGCTAACGCTAACCGCGGAGGCGAAATTGCACAG

TGGTCAAGCGACAGCGGCCTTGATTTCATCGGAGAACCAGGAGTGCCCACTCACCAGGCG

GGACACGTCCTCGATCTCACTTTCTCCAACATACCCTACGCCTCGACAGTCGTCAGGGAA

GACCTTGCCACCGGGTCTGACCATGAGTCCCTCGTCACTCGCATCCCGGGTCGCGGCAGG

GTCCCCCTCGAACAATACAACTATAGAGTTCCCGAGTCTAAGCTACCAAAACTGTCTTCC

CTCATCGGGACTGGGATCCGCTCCCTACCGGACCCAAGCAGCATCGAGACCCATGATCAA

CTAGACCAGTTCGCGGCGACTCTCACAGCACTTTTCCAGGACGCTATAAAGACAGCCGGG

TCCCTGAACCGCACTCATACCTTCACCACCCCGTGGTGGACCTCCGAATGCCAAGCCAAG

CGCCAACAGTGGCTAGGCGTAAGACACACGGATCCAGATAAGGCTGACACCGCTAAAAGA

GCTTTTCTCTCCTGTGTACGCTCCTCGAAAAGGGCCTACTGGAGGGACCGCATTGACAAT

ATCAAAACGGACTCTGACCTATACAATATCATCAGCTGGCATAAATTGGGCACCGACCTT

AAGGCCCCTCCCCTTCTCGTCGATGGCCTCCCTGTGGAGGACACCATGGAAAAGGCGGAG

GCCCTACGCCGCGCAGTCCTTGGCCGTTTTAGCCCAGACGACGACCTACCAACGGACCCT

ATCCCTATCACCACAACCTCCAGCCTTCCATGGCTACAATCTGTCACAATGGAAGAAGTT

GAGGCTAACACAATTGGCGTCTCCAGCACGTCGCCTGGCTCAGATAGGATAACTGTGCGC

CTACTCAAAGCCTGCTGGGAACACCTCAAAGACATAGTCCTCTCTCTGTTTAACCGATGC

CTCGCCCTCTGCCACATCCCCCTGGCCTGGAAGGTCGCGGAGGTGGCTATGATACCCAAG

GTCGGCAAGAAGGATAAGTCCTCCGTGCGCTCCTGGAGACCGATCGCCCTCCTATCCTGT

CTCTCTAAGGGACTAGAACGAATCATCGCGAAAAGAATTGCCTGGACTGCCCTCACCCAC

GGCGTCCTCAGCCCCCAGCATGCGGGGGCTCTACCCAAACTTTCCGCCACAGACCTAGTC

GCCTCCTTCACCCACGATGCCGAAATGGCACTCTCACAGAACAAGCAAGTCACCCTAGTC

ACGATGGACGTCCAGGGTGCATTCGATGCCCTCCTCCGGAGACGACTTCTCAAGCGCATG

GGCGAGCAGGGCTGGCCACGAGAACTGCTACTTCTTGTGGACAGCTTTCTCACCGGACGC

AAAGCCCGCGTCCGGCTAGAAGGCTCAACCACCCCTGAGTACGACGTGGTCTGCGGAACC

CCCCAAGGCTCCCCATTATCACCAGTACTATATATGCTCTACCTAGCCGAACTCCTCAAT

ATGGACCAAACGCTCCGTTTCGGGTATGCCGACGACCTAGCTCTATACCGAGCCTCCCAC

GATCTCACCCAGAATGTCAGACTCCTCGCTAAGGACGTTCAGAGCATTCTTGCCTGGGGA

GAATATAATAAAGTGGCCTTCGCCCCCGAGAAACTAGAGATGATCCATATCACTAGACAT

CGAGGGGATGAGTCCCCTTCAATTGTAGTCAACGACCGGCTCACCATCGACCCCGTTCAG

GCCAAGAAACCAGGATACACACCCACTCTCCGCTGGCTGGGAGTCTTTTTCGATAGAAAG

CTCACATGGAGAAGCCACATTCTCGCCCGGGCGGGCAAGGCACGCGCTGTCGCACAACAT

ATCCGCAATCTGGCCCGCACGACCTGCGGCCCGCCCGCGAGCTCACTTCGCAAAGCAGTC

ATCACCTGTGTTATACCCTCCCTAACGTTCGGAACTGAGGCCTGGTATGGCGGCCGGAAT

AGGCCCGCAAAACAGGCTAGCAAGGGCACCGTCAGCGCCCGTGTTGGCTGGCATATCAAT

GTCATCGAGTCGACCCTGGCACTCGCCATCCGCGGCGTCCTCCCTGTATGGCGCACCACA

CCAACTCCCTCGCTCTTTAGGGATGCGGGAATCCCGTCTGGATACGCCACACTTGAAGAG

GCGAAACTACGGTTCGCTCTAAGGCTTAACACCATCCACAAAGGTCACACCCTTGTCCGT

CGCATTCGACCCCCGATGATCACCCGAGGCCGCGGCACCGGCACCCGCCAACCGGCAAAG

ACAATTATCCAGAGACTTGGGAGCATCCTCCCGGAAGTCCCAAGACCGACCCTCTCCCCC

CCGCACTACTCACCGGGCTGCAGAATAGATCCTACAGGGGGTATAGATAAGGCGACCGCG

TCTAAAGCTTTCCAAGTCTGGCAGGAATCACTCCCACCCACAGATATCTGCGTCTTCTCA

GATGGCTCCGAGCAGTGGCAGGAGGGCATCAAGTACGTAGGCTATGGCTTCGTACTTATG

GTAAACGGCACCCAAATCGACACTGGCGCTGGCGCTATCAACTCACGCTCTCATGTTTTC

GACGCCGAAGCAATCGGAGCCTGGCGAGGGCTCGAACGGGCAATCGCGGTAGCTCCGCCT

AGGTCGAAAATCTGGCTCTGTATTGATAGCACATCCGTTATCTGGTGTATTAGGGGGAAT

GCCTCGAACTCCTCGCAATGGGCATTTTTAGCCTGCCACCGGGCTATGGAACAGCACAAT

ATCAGTCTCCGATGGGCCCCTGGGCACACTGGGATCGAAGGGAACGAAGCTGCTGACACC

TTAGCCGGTGAAGGCGCGCTACGCGGTAGTGGCTGGTGGGACACGGTCTCTCAAAAACTC

TCCCAGTGGTACCGACGCTGGTCAGACACCTACGAGATTGATTCACTGCCGGAACTCGAA

CTCCGACGACCAGCGCTCCACCGCTGGCTTGCCCTCCGCTCGTCGCATGGCGACTTCGAC

TGGTACCACCGCAAGTTCAACCACGAAGACGCCAAACTCGACTGCTCATGCGGCCGCCGA

AAGTCACCAGAGCACCTCGCTCTCTGCCACAAAACCCAGAGGTCTTTCCGACACTGGCCA

AAACGCCCCCCGACACCTCCAACCGACAGGACAGAGGCAGTCGCCTACCTTCGCAGCCTG

GACCCCAAGCAGTTTGTTGAACTACTGGAGCTCACAAGCTTCTACTCGCGGGTCTGCACG

AGG

>CL109.Contig15_All 5 3325 minus strand putative RNA-directed DNA polymerase from transposon X-element [Penicillium digitatum PHI26]

AGGAACGAAGCCTTCGAACCCGGCGTCGCTAACGCTAACCGCGGAGGCGAAATTGCACAG

TGGTCAAGCGACAGCGGCCTTGATTTCATCGGAGAACCAGGAGTGCCCACTCACCAGGCG

GGACACGTCCTCGATCTCACTTTCTCCAACATACCCTACGCCTCGACAGTCGTCAGGGAA

GACCTTGCCACCGGGTCTGACCATGAGTCCCTCGTCACTCGCATCCCGGGTCGCGGCAGG

GTCCCCCTCGAACAATACAACTATAGAGTTCCCGAGTCTAAGCTACCAAAACTGTCTTCC

CTCATCGGGACTGGGATCCGCTCCCTACCGGACCCAAGCAGCATCGAGACCCATGATCAA

CTAGACCAGTTCGCGGCGACTCTCACAGCACTTTTCCAGGACGCTATAAAGACAGCCGGG

TCCCTGAACCGCACTCATACCTTCACCACCCCGTGGTGGACCTCCGAATGCCAAGCCAAG

CGCCAACAGTGGCTAGGCGTAAGACACACGGATCCAGATAAGGCTGACACCGCTAAAAGA

GCTTTTCTCTCCTGTGTACGCTCCTCGAAAAGGGCCTACTGGAGGGACCGCATTGACAAT

ATCAAAACGGACTCTGACCTATACAATATCATCAGCTGGCATAAATTGGGCACCGACCTT

AAGGCCCCTCCCCTTCTCGTCGATGGCCTCCCTGTGGAGGACACCATGGAAAAGGCGGAG

GCCCTACGCCGCGCAGTCCTTGGCCGTTTTAGCCCAGACGACGACCTACCAACGGACCCT

ATCCCTATCACCACAACCTCCAGCCTTCCATGGCTACAATCTGTCACAATGGAAGAAGTT

GAGGCTAACACAATTGGCGTCTCCAGCACGTCGCCTGGCTCAGATAGGATAACTGTGCGC

CTACTCAAAGCCTGCTGGGAACACCTCAAAGACATAGTCCTCTCTCTGTTTAACCGATGC

CTCGCCCTCTGCCACATCCCCCTGGCCTGGAAGGTCGCGGAGGTGGCTATGATACCCAAG

GTCGGCAAGAAGGATAAGTCCTCCGTGCGCTCCTGGAGACCGATCGCCCTCCTATCCTGT

CTCTCTAAGGGACTAGAACGAATCATCGCGAAAAGAATTGCCTGGACTGCCCTCACCCAC

GGCGTCCTCAGCCCCCAGCATGCGGGGGCTCTACCCAAACTTTCCGCCACAGACCTAGTC

GCCTCCTTCACCCACGATGCCGAAATGGCACTCTCACAGAACAAGCAAGTCACCCTAGTC

ACGATGGACGTCCAGGGTGCATTCGATGCCCTCCTCCGGAGACGACTTCTCAAGCGCATG

GGCGAGCAGGGCTGGCCACGAGAACTGCTACTTCTTGTGGACAGCTTTCTCACCGGACGC

AAAGCCCGCGTCCGGCTAGAAGGCTCAACCACCCCTGAGTACGACGTGGTCTGCGGAACC

CCCCAAGGCTCCCCATTATCACCAGTACTATATATGCTCTACCTAGCCGAACTCCTCAAT

ATGGACCAAACGCTCCGTTTCGGGTATGCCGACGACCTAGCTCTATACCGAGCCTCCCAC

GATCTCACCCAGAATGTCAGACTCCTCGCTAAGGACGTTCAGAGCATTCTTGCCTGGGGA

GAATATAATAAAGTGGCCTTCGCCCCCGAGAAACTAGAGATGATCCATATCACTAGACAT

CGAGGGGATGAGTCCCCTTCAATTGTAGTCAACGACCGGCTCACCATCGACCCCGTTCAG

GCCAAGAAACCAGGATACACACCCACTCTCCGCTGGCTGGGAGTCTTTTTCGATAGAAAG

CTCACATGGAGAAGCCACATTCTCGCCCGGGCGGGCAAGGCACGCGCTGTCGCACAACAT

ATCCGCAATCTGGCCCGCACGACCTGCGGCCCGCCCGCGAGCTCACTTCGCAAAGCAGTC

ATCACCTGTGTTATACCCTCCCTAACGTTCGGAACTGAGGCCTGGTATGGCGGCCGGAAT

AGGCCCGCAAAACAGGCTAGCAAGGGCACCGTCAGCGCCCGTGTTGGCTGGCATATCAAT

GTCATCGAGTCGACCCTGGCACTCGCCATCCGCGGCGTCCTCCCTGTATGGCGCACCACA

CCAACTCCCTCGCTCTTTAGGGATGCGGGAATCCCGTCTGGATACGCCACACTTGAAGAG

GCGAAACTACGGTTCGCTCTAAGGCTTAACACCATCCACAAAGGTCACACCCTTGTCCGT

CGCATTCGACCCCCGATGATCACCCGAGGCCGCGGCACCGGCACCCGCCAACCGGCAAAG

ACAATTATCCAGAGACTTGGGAGCATCCTCCCGGAAGTCCCAAGACCGACCCTCTCCCCC

CCGCACTACTCACCGGGCTGCAGAATAGATCCTACAGGGGGTATAGATAAGGCGACCGCG

TCTAAAGCTTTCCAAGTCTGGCAGGAATCACTCCCACCCACAGATATCTGCGTCTTCTCA

GATGGCTCCGAGCAGTGGCAGGAGGGCATCAAGTACGTAGGCTATGGCTTCGTACTTATG

GTAAACGGCACCCAAATCGACACTGGCGCTGGCGCTATCAACTCACGCTCTCATGTTTTC

GACGCCGAAGCAATCGGAGCCTGGCGAGGGCTCGAACGGGCAATCGCGGTAGCTCCGCCT

AGGTCGAAAATCTGGCTCTGTATTGATAGCACATCCGTTATCTGGTGTATTAGGGGGAAT

GCCTCGAACTCCTCGCAATGGGCATTTTTAGCCTGCCACCGGGCTATGGAACAGCACAAT

ATCAGTCTCCGATGGGCCCCTGGGCACACTGGGATCGAAGGGAACGAAGCTGCTGACACC

TTAGCCGGTGAAGGCGCGCTACGCGGTAGTGCTATAGGGATGGAAGCCGAACCCACGATT

AGCGGGATCCGATCCATCTTCCGGGAACTTCGGAACGAGGCTCGCTTGCGCTGGTGGGAC

ACGGTCTCTCAAAAACTCTCCCAGTGGTACCGACGCTGGTCAGACACCTACGAGATTGAT

TCACTGCCGGAACTCGAACTCCGACGACCAGCGCTCCACCGCTGGCTTGCCCTCCGCTCG

TCGCATGGCGACTTCGACTGGTACCACCGCAAGTTCAACCACGAAGACGCCAAACTCGAC

TGCTCATGCGGCCGCCGAAAGTCACCAGAGCACCTCGCTCTCTGCCACAAAACCCAGAGG

TCTTTCCGACACTGGCCAAAACGCCCCCCGACACCTCCAACCGACAGGACAGAGGCAGTC

GCCTACCTTCGCAGCCTGGACCCCAAGCAGTTTGTTGAACTACTGGAGCTCACAAGCTTC

TACTCGCGGGTCTGCACGAGG

>CL109.Contig16_All 5 3247 minus strand putative RNA-directed DNA polymerase from transposon X-element [Penicillium digitatum PHI26]

AGGAACGAAGCCTTCGAACCCGGCGTCGCTAACGCTAACCGCGGAGGCGAAATTGCACAG

TGGTCAAGCGACAGCGGCCTTGATTTCATCGGAGAACCAGGAGTGCCCACTCACCAGGCG

GGACACGTCCTCGATCTCACTTTCTCCAACATACCCTACGCCTCGACAGTCGTCAGGGAA

GACCTTGCCACCGGGTCTGACCATGAGTCCCTCGTCACTCGCATCCCGGGTCGCGGCAGG

GTCCCCCTCGAACAATACAACTATAGAGTTCCCGAGTCTAAGCTACCAAAACTGTCTTCC

CTCATCGGGACTGGGATCCGCTCCCTACCGGACCCAAGCAGCATCGAGACCCATGATCAA

CTAGACCAGTTCGCGGCGACTCTCACAGCACTTTTCCAGGACGCTATAAAGACAGCCGGG

TCCCTGAACCGCACTCATACCTTCACCACCCCGTGGTGGACCTCCGAATGCCAAGCCAAG

CGCCAACAGTGGCTAGGCGTAAGACACACGGATCCAGATAAGGCTGACACCGCTAAAAGA

GCTTTTCTCTCCTGTGTACGCTCCTCGAAAAGGGCCTACTGGAGGGACCGCATTGACAAT

ATCAAAACGGACTCTGACCTATACAATATCATCAGCTGGCATAAATTGGGCACCGACCTT

AAGGCCCCTCCCCTTCTCGTCGATGGCCTCCCTGTGGAGGACACCATGGAAAAGGCGGAG

GCCCTACGCCGCGCAGTCCTTGGCCGTTTTAGCCCAGACGACGACCTACCAACGGACCCT

ATCCCTATCACCACAACCTCCAGCCTTCCATGGCTACAATCTGTCACAATGGAAGAAGTT

GAGGCTAACACAATTGGCGTCTCCAGCACGTCGCCTGGCTCAGATAGGATAACTGTGCGC

CTACTCAAAGCCTGCTGGGAACACCTCAAAGACATAGTCCTCTCTCTGTTTAACCGATGC

CTCGCCCTCTGCCACATCCCCCTGGCCTGGAAGGTCGCGGAGGTGGCTATGATACCCAAG

GTCGGCAAGAAGGATAAGTCCTCCGTGCGCTCCTGGAGACCGATCGCCCTCCTATCCTGT

CTCTCTAAGGGACTAGAACGAATCATCGCGAAAAGAATTGCCTGGACTGCCCTCACCCAC

GGCGTCCTCAGCCCCCAGCATGCGGGGGCTCTACCCAAACTTTCCGCCACAGACCTAGTC

GCCTCCTTCACCCACGATGCCGAAATGGCACTCTCACAGAACAAGCAAGTCACCCTAGTC

ACGATGGACGTCCAGGGTGCATTCGATGCCCTCCTCCGGAGACGACTTCTCAAGCGCATG

GGCGAGCAGGGCTGGCCACGAGAACTGCTACTTCTTGTGGACAGCTTTCTCACCGGACGC

AAAGCCCGCGTCCGGCTAGAAGGCTCAACCACCCCTGAGTACGACGTGGTCTGCGGAACC

CCCCAAGGCTCCCCATTATCACCAGTACTATATATGCTCTACCTAGCCGAACTCCTCAAT

ATGGACCAAACGCTCCGTTTCGGGTATGCCGACGACCTAGCTCTATACCGAGCCTCCCAC

GATCTCACCCAGAATGTCAGACTCCTCGCTAAGGACGTTCAGAGCATTCTTGCCTGGGGA

GAATATAATAAAGTGGCCTTCGCCCCCGAGAAACTAGAGATGATCCATATCACTAGACAT

CGAGGGGATGAGTCCCCTTCAATTGTAGTCAACGACCGGCTCACCATCGACCCCGTTCAG

GCCAAGAAACCAGGATACACACCCACTCTCCGCTGGCTGGGAGTCTTTTTCGATAGAAAG

CTCACATGGAGAAGCCACATTCTCGCCCGGGCGGGCAAGGCACGCGCTGTCGCACAACAT

ATCCGCAATCTGGCCCGCACGACCTGCGGCCCGCCCGCGAGCTCACTTCGCAAAGCAGTC

ATCACCTGTGTTATACCCTCCCTAACGTTCGGAACTGAGGCCTGGTATGGCGGCCGGAAT

AGGCCCGCAAAACAGGCTAGCAAGGGCACCGTCAGCGCCCGTGTTGGCTGGCATATCAAT

GTCATCGAGTCGACCCTGGCACTCGCCATCCGCGGCGTCCTCCCTGTATGGCGCACCACA

CCAACTCCCTCGCTCTTTAGGGATGCGGGAATCCCGTCTGGATACGCCACACTTGAAGAG

GCGAAACTACGGTTCGCTCTAAGGCTTAACACCATCCACAAAGGTCACACCCTTGTCCGT

CGCATTCGACCCCCGATGATCACCCGAGGCCGCGGCACCGGCACCCGCCAACCGGCAAAG

ACAATTATCCAGAGACTTGGGAGCATCCTCCCGGAAGTCCCAAGACCGACCCTCTCCCCC

CCGCACTACTCACCGGGCTGCAGAATAGATCCTACAGGGGGTATAGATAAGGCGACCGCG

TCTAAAGCTTTCCAAGTCTGGCAGGAATCACTCCCACCCACAGATATCTGCGTCTTCTCA

GATGGCTCCGAGCAGTGGCAGGAGGGCATCAAGTACGTAGGCTATGGCTTCGTACTTATG

GTAAACGGCACCCAAATCGACACTGGCGCTGGCGCTATCAACTCACGCTCTCATGTTTTC

GACGCCGAAGCAATCGGAGCCTGGCGAGGGCTCGAACGGGCAATCGCGGTAGCTCCGCCT

AGGTCGAAAATCTGGCTCTGTATTGATAGCACATCCGTTATCTGGTGTATTAGGGGGAAT

GCCTCGAACTCCTCGCAATGGGCATTTTTAGCCTGCCACCGGGCTATGGAACAGCACAAT

ATCAGTCTCCGATGGGCCCCTGGGCACACTGGGATCGAAGGGAACGAAGCTGCTGACACC

TTAGCCGGTGAAGGCGCGCTACGCGGTAGTGGCTGGTGGGACACGGTCTCTCAAAAACTC

TCCCAGTGGTACCGACGCTGGTCAGACACCTACGAGATTGATTCACTGCCGGAACTCGAA

CTCCGACGACCAGCGCTCCACCGCTGGCTTGCCCTCCGCTCGTCGCATGGCGACTTCGAC

TGGTACCACCGCAAGTTCAACCACGAAGACGCCAAACTCGACTGCTCATGCGGCCGCCGA

AAGTCACCAGAGCACCTCGCTCTCTGCCACAAAACCCAGAGGTCTTTCCGACACTGGCCA

AAACGCCCCCCGACACCTCCAACCGACAGGACAGAGGCAGTCGCCTACCTTCGCAGCCTG

GACCCCAAGCAGTTTGTTGAACTACTGGAGCTCACAAGCTTCTACTCGCGGGTCTGCACG

AGG

>CL109.Contig17_All 43 2577 minus strand putative RNA-directed DNA polymerase from transposon X-element [Penicillium digitatum PHI26]

CTCACCACAACCTCCAGCCTTCCATGGCTACAATCTGTCACAATGGAAGAAGTTGAGGCT

AACACAATTGGCGTCTCCAGCACGTCGCCTGGCTCAGATAGGATAACTGTGCGCCTACTC

AAAGCCTGCTGGGAACACCTCAAAGACATAGTCCTCTCTCTGTTTAACCGATGCCTCGCC

CTCTGCCACATCCCCCTGGCCTGGAAGGTCGCGGAGGTGGCTATGATACCCAAGGTCGGC

AAGAAGGATAAGTCCTCCGTGCGCTCCTGGAGACCGATCGCCCTCCTATCCTGTCTCTCT

AAGGGACTAGAACGAATCATCGCGAAAAGAATTGCCTGGACTGCCCTCACCCACGGCGTC

CTCAGCCCCCAGCATGCGGGGGCTCTACCCAAACTTTCCGCCACAGACCTAGTCGCCTCC

TTCACCCACGATGCCGAAATGGCACTCTCACAGAACAAGCAAGTCACCCTAGTCACGATG

GACGTCCAGGGTGCATTCGATGCCCTCCTCCGGAGACGACTTCTCAAGCGCATGGGCGAG

CAGGGCTGGCCACGAGAACTGCTACTTCTTGTGGACAGCTTTCTCACCGGACGCAAAGCC

CGCGTCCGGCTAGAAGGCTCAACCACCCCTGAGTACGACGTGGTCTGCGGAACCCCCCAA

GGCTCCCCATTATCACCAGTACTATATATGCTCTACCTAGCCGAACTCCTCAATATGGAC

CAAACGCTCCGTTTCGGGTATGCCGACGACCTAGCTCTATACCGAGCCTCCCACGATCTC

ACCCAGAATGTCAGACTCCTCGCTAAGGACGTTCAGAGCATTCTTGCCTGGGGAGAATAT

AATAAAGTGGCCTTCGCCCCCGAGAAACTAGAGATGATCCATATCACTAGACATCGAGGG

GATGAGTCCCCTTCAATTGTAGTCAACGACCGGCTCACCATCGACCCCGTTCAGGCCAAG

AAACCAGGATACACACCCACTCTCCGCTGGCTGGGAGTCTTTTTCGATAGAAAGCTCACA

TGGAGAAGCCACATTCTCGCCCGGGCGGGCAAGGCACGCGCTGTCGCACAACATATCCGC

AATCTGGCCCGCACGACCTGCGGCCCGCCCGCGAGCTCACTTCGCAAAGCAGTCATCACC

TGTGTTATACCCTCCCTAACGTTCGGAACTGAGGCCTGGTATGGCGGCCGGAATAGGCCC

GCAAAACAGGCTAGCAAGGGCACCGTCAGCGCCCGTGTTGGCTGGCATATCAATGTCATC

GAGTCGACCCTGGCACTCGCCATCCGCGGCGTCCTCCCTGTATGGCGCACCACACCAACT

CCCTCGCTCTTTAGGGATGCGGGAATCCCGTCTGGATACGCCACACTTGAAGAGGCGAAA

CTACGGTTCGCTCTAAGGCTTAACACCATCCACAAAGGTCACACCCTTGTCCGTCGCATT

CGACCCCCGATGATCACCCGAGGCCGCGGCACCGGCACCCGCCAACCGGCAAAGACAATT

ATCCAGAGACTTGGGAGCATCCTCCCGGAAGTCCCAAGACCGACCCTCTCCCCCCCGCAC

TACTCACCGGGCTGCAGAATAGATCCTACAGGGGGTATAGATAAGGCGACCGCGTCTAAA

GCTTTCCAAGTCTGGCAGGAATCACTCCCACCCACAGATATCTGCGTCTTCTCAGATGGC

TCCGAGCAGTGGCAGGAGGGCATCAAGTACGTAGGCTATGGCTTCGTACTTATGGTAAAC

GGCACCCAAATCGACACTGGCGCTGGCGCTATCAACTCACGCTCTCATGTTTTCGACGCC

GAAGCAATCGGAGCCTGGCGAGGGCTCGAACGGGCAATCGCGGTAGCTCCGCCTAGGTCG

AAAATCTGGCTCTGTATTGATAGCACATCCGTTATCTGGTGTATTAGGGGGAATGCCTCG

AACTCCTCGCAATGGGCATTTTTAGCCTGCCACCGGGCTATGGAACAGCACAATATCAGT

CTCCGATGGGCCCCTGGGCACACTGGGATCGAAGGGAACGAAGCTGCTGACACCTTAGCC

GGTGAAGGCGCGCTACGCGGTAGTGCTATAGGGATGGAAGCCGAACCCACGATTAGCGGG

ATCCGATCCATCTTCCGGGAACTTCGGAACGAGGCTCGCTTGCGCTGGTGGGACACGGTC

TCTCAAAAACTCTCCCAGTGGTACCGACGCTGGTCAGACACCTACGAGATTGATTCACTG

CCGGAACTCGAACTCCGACGACCAGCGCTCCACCGCTGGCTTGCCCTCCGCTCGTCGCAT

GGCGACTTCGACTGGTACCACCGCAAGTTCAACCACGAAGACGCCAAACTCGACTGCTCA

TGCGGCCGCCGAAAGTCACCAGAGCACCTCGCTCTCTGCCACAAAACCCAGAGGTCTTTC

CGACACTGGCCAAAACGCCCCCCGACACCTCCAACCGACAGGACAGAGGCAGTCGCCTAC

CTTCGCAGCCTGGACCCCAAGCAGTTTGTTGAACTACTGGAGCTCACAAGCTTCTACTCG

CGGGTCTGCACGAGG

>CL109.Contig18_All 43 2499 minus strand putative RNA-directed DNA polymerase from transposon X-element [Penicillium digitatum PHI26]

CTCACCACAACCTCCAGCCTTCCATGGCTACAATCTGTCACAATGGAAGAAGTTGAGGCT

AACACAATTGGCGTCTCCAGCACGTCGCCTGGCTCAGATAGGATAACTGTGCGCCTACTC

AAAGCCTGCTGGGAACACCTCAAAGACATAGTCCTCTCTCTGTTTAACCGATGCCTCGCC

CTCTGCCACATCCCCCTGGCCTGGAAGGTCGCGGAGGTGGCTATGATACCCAAGGTCGGC

AAGAAGGATAAGTCCTCCGTGCGCTCCTGGAGACCGATCGCCCTCCTATCCTGTCTCTCT

AAGGGACTAGAACGAATCATCGCGAAAAGAATTGCCTGGACTGCCCTCACCCACGGCGTC

CTCAGCCCCCAGCATGCGGGGGCTCTACCCAAACTTTCCGCCACAGACCTAGTCGCCTCC

TTCACCCACGATGCCGAAATGGCACTCTCACAGAACAAGCAAGTCACCCTAGTCACGATG

GACGTCCAGGGTGCATTCGATGCCCTCCTCCGGAGACGACTTCTCAAGCGCATGGGCGAG

CAGGGCTGGCCACGAGAACTGCTACTTCTTGTGGACAGCTTTCTCACCGGACGCAAAGCC

CGCGTCCGGCTAGAAGGCTCAACCACCCCTGAGTACGACGTGGTCTGCGGAACCCCCCAA

GGCTCCCCATTATCACCAGTACTATATATGCTCTACCTAGCCGAACTCCTCAATATGGAC

CAAACGCTCCGTTTCGGGTATGCCGACGACCTAGCTCTATACCGAGCCTCCCACGATCTC

ACCCAGAATGTCAGACTCCTCGCTAAGGACGTTCAGAGCATTCTTGCCTGGGGAGAATAT

AATAAAGTGGCCTTCGCCCCCGAGAAACTAGAGATGATCCATATCACTAGACATCGAGGG

GATGAGTCCCCTTCAATTGTAGTCAACGACCGGCTCACCATCGACCCCGTTCAGGCCAAG

AAACCAGGATACACACCCACTCTCCGCTGGCTGGGAGTCTTTTTCGATAGAAAGCTCACA

TGGAGAAGCCACATTCTCGCCCGGGCGGGCAAGGCACGCGCTGTCGCACAACATATCCGC

AATCTGGCCCGCACGACCTGCGGCCCGCCCGCGAGCTCACTTCGCAAAGCAGTCATCACC

TGTGTTATACCCTCCCTAACGTTCGGAACTGAGGCCTGGTATGGCGGCCGGAATAGGCCC

GCAAAACAGGCTAGCAAGGGCACCGTCAGCGCCCGTGTTGGCTGGCATATCAATGTCATC

GAGTCGACCCTGGCACTCGCCATCCGCGGCGTCCTCCCTGTATGGCGCACCACACCAACT

CCCTCGCTCTTTAGGGATGCGGGAATCCCGTCTGGATACGCCACACTTGAAGAGGCGAAA

CTACGGTTCGCTCTAAGGCTTAACACCATCCACAAAGGTCACACCCTTGTCCGTCGCATT

CGACCCCCGATGATCACCCGAGGCCGCGGCACCGGCACCCGCCAACCGGCAAAGACAATT

ATCCAGAGACTTGGGAGCATCCTCCCGGAAGTCCCAAGACCGACCCTCTCCCCCCCGCAC

TACTCACCGGGCTGCAGAATAGATCCTACAGGGGGTATAGATAAGGCGACCGCGTCTAAA

GCTTTCCAAGTCTGGCAGGAATCACTCCCACCCACAGATATCTGCGTCTTCTCAGATGGC

TCCGAGCAGTGGCAGGAGGGCATCAAGTACGTAGGCTATGGCTTCGTACTTATGGTAAAC

GGCACCCAAATCGACACTGGCGCTGGCGCTATCAACTCACGCTCTCATGTTTTCGACGCC

GAAGCAATCGGAGCCTGGCGAGGGCTCGAACGGGCAATCGCGGTAGCTCCGCCTAGGTCG

AAAATCTGGCTCTGTATTGATAGCACATCCGTTATCTGGTGTATTAGGGGGAATGCCTCG

AACTCCTCGCAATGGGCATTTTTAGCCTGCCACCGGGCTATGGAACAGCACAATATCAGT

CTCCGATGGGCCCCTGGGCACACTGGGATCGAAGGGAACGAAGCTGCTGACACCTTAGCC

GGTGAAGGCGCGCTACGCGGTAGTGGCTGGTGGGACACGGTCTCTCAAAAACTCTCCCAG

TGGTACCGACGCTGGTCAGACACCTACGAGATTGATTCACTGCCGGAACTCGAACTCCGA

CGACCAGCGCTCCACCGCTGGCTTGCCCTCCGCTCGTCGCATGGCGACTTCGACTGGTAC

CACCGCAAGTTCAACCACGAAGACGCCAAACTCGACTGCTCATGCGGCCGCCGAAAGTCA

CCAGAGCACCTCGCTCTCTGCCACAAAACCCAGAGGTCTTTCCGACACTGGCCAAAACGC

CCCCCGACACCTCCAACCGACAGGACAGAGGCAGTCGCCTACCTTCGCAGCCTGGACCCC

AAGCAGTTTGTTGAACTACTGGAGCTCACAAGCTTCTACTCGCGGGTCTGCACGAGG

>CL109.Contig19_All 43 2577 minus strand putative RNA-directed DNA polymerase from transposon X-element [Penicillium digitatum PHI26]

CTCACCACAACCTCCAGCCTTCCATGGCTACAATCTGTCACAATGGAAGAAGTTGAGGCT

AACACAATTGGCGTCTCCAGCACGTCGCCTGGCTCAGATAGGATAACTGTGCGCCTACTC

AAAGCCTGCTGGGAACACCTCAAAGACATAGTCCTCTCTCTGTTTAACCGATGCCTCGCC

CTCTGCCACATCCCCCTGGCCTGGAAGGTCGCGGAGGTGGCTATGATACCCAAGGTCGGC

AAGAAGGATAAGTCCTCCGTGCGCTCCTGGAGACCGATCGCCCTCCTATCCTGTCTCTCT

AAGGGACTAGAACGAATCATCGCGAAAAGAATTGCCTGGACTGCCCTCACCCACGGCGTC

CTCAGCCCCCAGCATGCGGGGGCTCTACCCAAACTTTCCGCCACAGACCTAGTCGCCTCC

TTCACCCACGATGCCGAAATGGCACTCTCACAGAACAAGCAAGTCACCCTAGTCACGATG

GACGTCCAGGGTGCATTCGATGCCCTCCTCCGGAGACGACTTCTCAAGCGCATGGGCGAG

CAGGGCTGGCCACGAGAACTGCTACTTCTTGTGGACAGCTTTCTCACCGGACGCAAAGCC

CGCGTCCGGCTAGAAGGCTCAACCACCCCTGAGTACGACGTGGTCTGCGGAACCCCCCAA

GGCTCCCCATTATCACCAGTACTATATATGCTCTACCTAGCCGAACTCCTCAATATGGAC

CAAACGCTCCGTTTCGGGTATGCCGACGACCTAGCTCTATACCGAGCCTCCCACGATCTC

ACCCAGAATGTCAGACTCCTCGCTAAGGACGTTCAGAGCATTCTTGCCTGGGGAGAATAT

AATAAAGTGGCCTTCGCCCCCGAGAAACTAGAGATGATCCATATCACTAGACATCGAGGG

GATGAGTCCCCTTCAATTGTAGTCAACGACCGGCTCACCATCGACCCCGTTCAGGCCAAG

AAACCAGGATACACACCCACTCTCCGCTGGCTGGGAGTCTTTTTCGATAGAAAGCTCACA

TGGAGAAGCCACATTCTCGCCCGGGCGGGCAAGGCACGCGCTGTCGCACAACATATCCGC

AATCTGGCCCGCACGACCTGCGGCCCGCCCGCGAGCTCACTTCGCAAAGCAGTCATCACC

TGTGTTATACCCTCCCTAACGTTCGGAACTGAGGCCTGGTATGGCGGCCGGAATAGGCCC

GCAAAACAGGCTAGCAAGGGCACCGTCAGCGCCCGTGTTGGCTGGCATATCAATGTCATC

GAGTCGACCCTGGCACTCGCCATCCGCGGCGTCCTCCCTGTATGGCGCACCACACCAACT

CCCTCGCTCTTTAGGGATGCGGGAATCCCGTCTGGATACGCCACACTTGAAGAGGCGAAA

CTACGGTTCGCTCTAAGGCTTAACACCATCCACAAAGGTCACACCCTTGTCCGTCGCATT

CGACCCCCGATGATCACCCGAGGCCGCGGCACCGGCACCCGCCAACCGGCAAAGACAATT

ATCCAGAGACTTGGGAGCATCCTCCCGGAAGTCCCAAGACCGACCCTCTCCCCCCCGCAC

TACTCACCGGGCTGCAGAATAGATCCTACAGGGGGTATAGATAAGGCGACCGCGTCTAAA

GCTTTCCAAGTCTGGCAGGAATCACTCCCACCCACAGATATCTGCGTCTTCTCAGATGGC

TCCGAGCAGTGGCAGGAGGGCATCAAGTACGTAGGCTATGGCTTCGTACTTATGGTAAAC

GGCACCCAAATCGACACTGGCGCTGGCGCTATCAACTCACGCTCTCATGTTTTCGACGCC

GAAGCAATCGGAGCCTGGCGAGGGCTCGAACGGGCAATCGCGGTAGCTCCGCCTAGGTCG

AAAATCTGGCTCTGTATTGATAGCACATCCGTTATCTGGTGTATTAGGGGGAATGCCTCG

AACTCCTCGCAATGGGCATTTTTAGCCTGCCACCGGGCTATGGAACAGCACAATATCAGT

CTCCGATGGGCCCCTGGGCACACTGGGATCGAAGGGAACGAAGCTGCTGACACCTTAGCC

GGTGAAGGCGCGCTACGCGGTAGTGCTATAGGGATGGAAGCCGAACCCACGATTAGCGGG

ATCCGATCCATCTTCCGGGAACTTCGGAACGAGGCTCGCTTGCGCTGGTGGGACACGGTC

TCTCAAAAACTCTCCCAGTGGTACCGACGCTGGTCAGACACCTACGAGATTGATTCACTG

CCGGAACTCGAACTCCGACGACCAGCGCTCCACCGCTGGCTTGCCCTCCGCTCGTCGCAT

GGCGACTTCGACTGGTACCACCGCAAGTTCAACCACGAAGACGCCAAACTCGACTGCTCA

TGCGGCCGCCGAAAGTCACCAGAGCACCTCGCTCTCTGCCACAAAACCCAGAGGTCTTTC

CGACACTGGCCAAAACGCCCCCCGACACCTCCAACCGACAGGACAGAGGCAGTCGCCTAC

CTTCGCAGCCTGGACCCCAAGCAGTTTGTTGAACTACTGGAGCTCACAAGCTTCTACTCG

CGGGTCTGCACGAGG

>CL109.Contig20_All 5 3325 minus strand putative RNA-directed DNA polymerase from transposon X-element [Penicillium digitatum PHI26]

AGGAACGAAGCCTTCGAACCCGGCGTCGCTAACGCTAACCGCGGAGGCGAAATTGCACAG

TGGTCAAGCGACAGCGGCCTTGATTTCATCGGAGAACCAGGAGTGCCCACTCACCAGGCG

GGACACGTCCTCGATCTCACTTTCTCCAACATACCCTACGCCTCGACAGTCGTCAGGGAA

GACCTTGCCACCGGGTCTGACCATGAGTCCCTCGTCACTCGCATCCCGGGTCGCGGCAGG

GTCCCCCTCGAACAATACAACTATAGAGTTCCCGAGTCTAAGCTACCAAAACTGTCTTCC

CTCATCGGGACTGGGATCCGCTCCCTACCGGACCCAAGCAGCATCGAGACCCATGATCAA

CTAGACCAGTTCGCGGCGACTCTCACAGCACTTTTCCAGGACGCTATAAAGACAGCCGGG

TCCCTGAACCGCACTCATACCTTCACCACCCCGTGGTGGACCTCCGAATGCCAAGCCAAG

CGCCAACAGTGGCTAGGCGTAAGACACACGGATCCAGATAAGGCTGACACCGCTAAAAGA

GCTTTTCTCTCCTGTGTACGCTCCTCGAAAAGGGCCTACTGGAGGGACCGCATTGACAAT

ATCAAAACGGACTCTGACCTATACAATATCATCAGCTGGCATAAATTGGGCACCGACCTT

AAGGCCCCTCCCCTTCTCGTCGATGGCCTCCCTGTGGAGGACACCATGGAAAAGGCGGAG

GCCCTACGCCGCGCAGTCCTTGGCCGTTTTAGCCCAGACGACGACCTACCAACGGACCCT

ATCCCTATCACCACAACCTCCAGCCTTCCATGGCTACAATCTGTCACAATGGAAGAAGTT

GAGGCTAACACAATTGGCGTCTCCAGCACGTCGCCTGGCTCAGATAGGATAACTGTGCGC

CTACTCAAAGCCTGCTGGGAACACCTCAAAGACATAGTCCTCTCTCTGTTTAACCGATGC

CTCGCCCTCTGCCACATCCCCCTGGCCTGGAAGGTCGCGGAGGTGGCTATGATACCCAAG

GTCGGCAAGAAGGATAAGTCCTCCGTGCGCTCCTGGAGACCGATCGCCCTCCTATCCTGT

CTCTCTAAGGGACTAGAACGAATCATCGCGAAAAGAATTGCCTGGACTGCCCTCACCCAC

GGCGTCCTCAGCCCCCAGCATGCGGGGGCTCTACCCAAACTTTCCGCCACAGACCTAGTC

GCCTCCTTCACCCACGATGCCGAAATGGCACTCTCACAGAACAAGCAAGTCACCCTAGTC

ACGATGGACGTCCAGGGTGCATTCGATGCCCTCCTCCGGAGACGACTTCTCAAGCGCATG

GGCGAGCAGGGCTGGCCACGAGAACTGCTACTTCTTGTGGACAGCTTTCTCACCGGACGC

AAAGCCCGCGTCCGGCTAGAAGGCTCAACCACCCCTGAGTACGACGTGGTCTGCGGAACC

CCCCAAGGCTCCCCATTATCACCAGTACTATATATGCTCTACCTAGCCGAACTCCTCAAT

ATGGACCAAACGCTCCGTTTCGGGTATGCCGACGACCTAGCTCTATACCGAGCCTCCCAC

GATCTCACCCAGAATGTCAGACTCCTCGCTAAGGACGTTCAGAGCATTCTTGCCTGGGGA

GAATATAATAAAGTGGCCTTCGCCCCCGAGAAACTAGAGATGATCCATATCACTAGACAT

CGAGGGGATGAGTCCCCTTCAATTGTAGTCAACGACCGGCTCACCATCGACCCCGTTCAG

GCCAAGAAACCAGGATACACACCCACTCTCCGCTGGCTGGGAGTCTTTTTCGATAGAAAG

CTCACATGGAGAAGCCACATTCTCGCCCGGGCGGGCAAGGCACGCGCTGTCGCACAACAT

ATCCGCAATCTGGCCCGCACGACCTGCGGCCCGCCCGCGAGCTCACTTCGCAAAGCAGTC

ATCACCTGTGTTATACCCTCCCTAACGTTCGGAACTGAGGCCTGGTATGGCGGCCGGAAT

AGGCCCGCAAAACAGGCTAGCAAGGGCACCGTCAGCGCCCGTGTTGGCTGGCATATCAAT

GTCATCGAGTCGACCCTGGCACTCGCCATCCGCGGCGTCCTCCCTGTATGGCGCACCACA

CCAACTCCCTCGCTCTTTAGGGATGCGGGAATCCCGTCTGGATACGCCACACTTGAAGAG

GCGAAACTACGGTTCGCTCTAAGGCTTAACACCATCCACAAAGGTCACACCCTTGTCCGT

CGCATTCGACCCCCGATGATCACCCGAGGCCGCGGCACCGGCACCCGCCAACCGGCAAAG

ACAATTATCCAGAGACTTGGGAGCATCCTCCCGGAAGTCCCAAGACCGACCCTCTCCCCC

CCGCACTACTCACCGGGCTGCAGAATAGATCCTACAGGGGGTATAGATAAGGCGACCGCG

TCTAAAGCTTTCCAAGTCTGGCAGGAATCACTCCCACCCACAGATATCTGCGTCTTCTCA

GATGGCTCCGAGCAGTGGCAGGAGGGCATCAAGTACGTAGGCTATGGCTTCGTACTTATG

GTAAACGGCACCCAAATCGACACTGGCGCTGGCGCTATCAACTCACGCTCTCATGTTTTC

GACGCCGAAGCAATCGGAGCCTGGCGAGGGCTCGAACGGGCAATCGCGGTAGCTCCGCCT

AGGTCGAAAATCTGGCTCTGTATTGATAGCACATCCGTTATCTGGTGTATTAGGGGGAAT

GCCTCGAACTCCTCGCAATGGGCATTTTTAGCCTGCCACCGGGCTATGGAACAGCACAAT

ATCAGTCTCCGATGGGCCCCTGGGCACACTGGGATCGAAGGGAACGAAGCTGCTGACACC

TTAGCCGGTGAAGGCGCGCTACGCGGTAGTGCTATAGGGATGGAAGCCGAACCCACGATT

AGCGGGATCCGATCCATCTTCCGGGAACTTCGGAACGAGGCTCGCTTGCGCTGGTGGGAC

ACGGTCTCTCAAAAACTCTCCCAGTGGTACCGACGCTGGTCAGACACCTACGAGATTGAT

TCACTGCCGGAACTCGAACTCCGACGACCAGCGCTCCACCGCTGGCTTGCCCTCCGCTCG

TCGCATGGCGACTTCGACTGGTACCACCGCAAGTTCAACCACGAAGACGCCAAACTCGAC

TGCTCATGCGGCCGCCGAAAGTCACCAGAGCACCTCGCTCTCTGCCACAAAACCCAGAGG

TCTTTCCGACACTGGCCAAAACGCCCCCCGACACCTCCAACCGACAGGACAGAGGCAGTC

GCCTACCTTCGCAGCCTGGACCCCAAGCAGTTTGTTGAACTACTGGAGCTCACAAGCTTC

TACTCGCGGGTCTGCACGAGG

>CL109.Contig21_All 5 3325 minus strand putative RNA-directed DNA polymerase from transposon X-element [Penicillium digitatum PHI26]

AGGAACGAAGCCTTCGAACCCGGCGTCGCTAACGCTAACCGCGGAGGCGAAATTGCACAG

TGGTCAAGCGACAGCGGCCTTGATTTCATCGGAGAACCAGGAGTGCCCACTCACCAGGCG

GGACACGTCCTCGATCTCACTTTCTCCAACATACCCTACGCCTCGACAGTCGTCAGGGAA

GACCTTGCCACCGGGTCTGACCATGAGTCCCTCGTCACTCGCATCCCGGGTCGCGGCAGG

GTCCCCCTCGAACAATACAACTATAGAGTTCCCGAGTCTAAGCTACCAAAACTGTCTTCC

CTCATCGGGACTGGGATCCGCTCCCTACCGGACCCAAGCAGCATCGAGACCCATGATCAA

CTAGACCAGTTCGCGGCGACTCTCACAGCACTTTTCCAGGACGCTATAAAGACAGCCGGG

TCCCTGAACCGCACTCATACCTTCACCACCCCGTGGTGGACCTCCGAATGCCAAGCCAAG

CGCCAACAGTGGCTAGGCGTAAGACACACGGATCCAGATAAGGCTGACACCGCTAAAAGA

GCTTTTCTCTCCTGTGTACGCTCCTCGAAAAGGGCCTACTGGAGGGACCGCATTGACAAT

ATCAAAACGGACTCTGACCTATACAATATCATCAGCTGGCATAAATTGGGCACCGACCTT

AAGGCCCCTCCCCTTCTCGTCGATGGCCTCCCTGTGGAGGACACCATGGAAAAGGCGGAG

GCCCTACGCCGCGCAGTCCTTGGCCGTTTTAGCCCAGACGACGACCTACCAACGGACCCT

ATCCCTATCACCACAACCTCCAGCCTTCCATGGCTACAATCTGTCACAATGGAAGAAGTT

GAGGCTAACACAATTGGCGTCTCCAGCACGTCGCCTGGCTCAGATAGGATAACTGTGCGC

CTACTCAAAGCCTGCTGGGAACACCTCAAAGACATAGTCCTCTCTCTGTTTAACCGATGC

CTCGCCCTCTGCCACATCCCCCTGGCCTGGAAGGTCGCGGAGGTGGCTATGATACCCAAG

GTCGGCAAGAAGGATAAGTCCTCCGTGCGCTCCTGGAGACCGATCGCCCTCCTATCCTGT

CTCTCTAAGGGACTAGAACGAATCATCGCGAAAAGAATTGCCTGGACTGCCCTCACCCAC

GGCGTCCTCAGCCCCCAGCATGCGGGGGCTCTACCCAAACTTTCCGCCACAGACCTAGTC

GCCTCCTTCACCCACGATGCCGAAATGGCACTCTCACAGAACAAGCAAGTCACCCTAGTC

ACGATGGACGTCCAGGGTGCATTCGATGCCCTCCTCCGGAGACGACTTCTCAAGCGCATG

GGCGAGCAGGGCTGGCCACGAGAACTGCTACTTCTTGTGGACAGCTTTCTCACCGGACGC

AAAGCCCGCGTCCGGCTAGAAGGCTCAACCACCCCTGAGTACGACGTGGTCTGCGGAACC

CCCCAAGGCTCCCCATTATCACCAGTACTATATATGCTCTACCTAGCCGAACTCCTCAAT

ATGGACCAAACGCTCCGTTTCGGGTATGCCGACGACCTAGCTCTATACCGAGCCTCCCAC

GATCTCACCCAGAATGTCAGACTCCTCGCTAAGGACGTTCAGAGCATTCTTGCCTGGGGA

GAATATAATAAAGTGGCCTTCGCCCCCGAGAAACTAGAGATGATCCATATCACTAGACAT

CGAGGGGATGAGTCCCCTTCAATTGTAGTCAACGACCGGCTCACCATCGACCCCGTTCAG

GCCAAGAAACCAGGATACACACCCACTCTCCGCTGGCTGGGAGTCTTTTTCGATAGAAAG

CTCACATGGAGAAGCCACATTCTCGCCCGGGCGGGCAAGGCACGCGCTGTCGCACAACAT

ATCCGCAATCTGGCCCGCACGACCTGCGGCCCGCCCGCGAGCTCACTTCGCAAAGCAGTC

ATCACCTGTGTTATACCCTCCCTAACGTTCGGAACTGAGGCCTGGTATGGCGGCCGGAAT

AGGCCCGCAAAACAGGCTAGCAAGGGCACCGTCAGCGCCCGTGTTGGCTGGCATATCAAT

GTCATCGAGTCGACCCTGGCACTCGCCATCCGCGGCGTCCTCCCTGTATGGCGCACCACA

CCAACTCCCTCGCTCTTTAGGGATGCGGGAATCCCGTCTGGATACGCCACACTTGAAGAG

GCGAAACTACGGTTCGCTCTAAGGCTTAACACCATCCACAAAGGTCACACCCTTGTCCGT

CGCATTCGACCCCCGATGATCACCCGAGGCCGCGGCACCGGCACCCGCCAACCGGCAAAG

ACAATTATCCAGAGACTTGGGAGCATCCTCCCGGAAGTCCCAAGACCGACCCTCTCCCCC

CCGCACTACTCACCGGGCTGCAGAATAGATCCTACAGGGGGTATAGATAAGGCGACCGCG

TCTAAAGCTTTCCAAGTCTGGCAGGAATCACTCCCACCCACAGATATCTGCGTCTTCTCA

GATGGCTCCGAGCAGTGGCAGGAGGGCATCAAGTACGTAGGCTATGGCTTCGTACTTATG

GTAAACGGCACCCAAATCGACACTGGCGCTGGCGCTATCAACTCACGCTCTCATGTTTTC

GACGCCGAAGCAATCGGAGCCTGGCGAGGGCTCGAACGGGCAATCGCGGTAGCTCCGCCT

AGGTCGAAAATCTGGCTCTGTATTGATAGCACATCCGTTATCTGGTGTATTAGGGGGAAT

GCCTCGAACTCCTCGCAATGGGCATTTTTAGCCTGCCACCGGGCTATGGAACAGCACAAT

ATCAGTCTCCGATGGGCCCCTGGGCACACTGGGATCGAAGGGAACGAAGCTGCTGACACC

TTAGCCGGTGAAGGCGCGCTACGCGGTAGTGCTATAGGGATGGAAGCCGAACCCACGATT

AGCGGGATCCGATCCATCTTCCGGGAACTTCGGAACGAGGCTCGCTTGCGCTGGTGGGAC

ACGGTCTCTCAAAAACTCTCCCAGTGGTACCGACGCTGGTCAGACACCTACGAGATTGAT

TCACTGCCGGAACTCGAACTCCGACGACCAGCGCTCCACCGCTGGCTTGCCCTCCGCTCG

TCGCATGGCGACTTCGACTGGTACCACCGCAAGTTCAACCACGAAGACGCCAAACTCGAC

TGCTCATGCGGCCGCCGAAAGTCACCAGAGCACCTCGCTCTCTGCCACAAAACCCAGAGG

TCTTTCCGACACTGGCCAAAACGCCCCCCGACACCTCCAACCGACAGGACAGAGGCAGTC

GCCTACCTTCGCAGCCTGGACCCCAAGCAGTTTGTTGAACTACTGGAGCTCACAAGCTTC

TACTCGCGGGTCTGCACGAGG

>CL109.Contig22_All 43 2577 minus strand putative RNA-directed DNA polymerase from transposon X-element [Penicillium digitatum PHI26]

CTCACCACAACCTCCAGCCTTCCATGGCTACAATCTGTCACAATGGAAGAAGTTGAGGCT

AACACAATTGGCGTCTCCAGCACGTCGCCTGGCTCAGATAGGATAACTGTGCGCCTACTC

AAAGCCTGCTGGGAACACCTCAAAGACATAGTCCTCTCTCTGTTTAACCGATGCCTCGCC

CTCTGCCACATCCCCCTGGCCTGGAAGGTCGCGGAGGTGGCTATGATACCCAAGGTCGGC

AAGAAGGATAAGTCCTCCGTGCGCTCCTGGAGACCGATCGCCCTCCTATCCTGTCTCTCT

AAGGGACTAGAACGAATCATCGCGAAAAGAATTGCCTGGACTGCCCTCACCCACGGCGTC

CTCAGCCCCCAGCATGCGGGGGCTCTACCCAAACTTTCCGCCACAGACCTAGTCGCCTCC

TTCACCCACGATGCCGAAATGGCACTCTCACAGAACAAGCAAGTCACCCTAGTCACGATG

GACGTCCAGGGTGCATTCGATGCCCTCCTCCGGAGACGACTTCTCAAGCGCATGGGCGAG

CAGGGCTGGCCACGAGAACTGCTACTTCTTGTGGACAGCTTTCTCACCGGACGCAAAGCC

CGCGTCCGGCTAGAAGGCTCAACCACCCCTGAGTACGACGTGGTCTGCGGAACCCCCCAA

GGCTCCCCATTATCACCAGTACTATATATGCTCTACCTAGCCGAACTCCTCAATATGGAC

CAAACGCTCCGTTTCGGGTATGCCGACGACCTAGCTCTATACCGAGCCTCCCACGATCTC

ACCCAGAATGTCAGACTCCTCGCTAAGGACGTTCAGAGCATTCTTGCCTGGGGAGAATAT

AATAAAGTGGCCTTCGCCCCCGAGAAACTAGAGATGATCCATATCACTAGACATCGAGGG

GATGAGTCCCCTTCAATTGTAGTCAACGACCGGCTCACCATCGACCCCGTTCAGGCCAAG

AAACCAGGATACACACCCACTCTCCGCTGGCTGGGAGTCTTTTTCGATAGAAAGCTCACA

TGGAGAAGCCACATTCTCGCCCGGGCGGGCAAGGCACGCGCTGTCGCACAACATATCCGC

AATCTGGCCCGCACGACCTGCGGCCCGCCCGCGAGCTCACTTCGCAAAGCAGTCATCACC

TGTGTTATACCCTCCCTAACGTTCGGAACTGAGGCCTGGTATGGCGGCCGGAATAGGCCC

GCAAAACAGGCTAGCAAGGGCACCGTCAGCGCCCGTGTTGGCTGGCATATCAATGTCATC

GAGTCGACCCTGGCACTCGCCATCCGCGGCGTCCTCCCTGTATGGCGCACCACACCAACT

CCCTCGCTCTTTAGGGATGCGGGAATCCCGTCTGGATACGCCACACTTGAAGAGGCGAAA

CTACGGTTCGCTCTAAGGCTTAACACCATCCACAAAGGTCACACCCTTGTCCGTCGCATT

CGACCCCCGATGATCACCCGAGGCCGCGGCACCGGCACCCGCCAACCGGCAAAGACAATT

ATCCAGAGACTTGGGAGCATCCTCCCGGAAGTCCCAAGACCGACCCTCTCCCCCCCGCAC

TACTCACCGGGCTGCAGAATAGATCCTACAGGGGGTATAGATAAGGCGACCGCGTCTAAA

GCTTTCCAAGTCTGGCAGGAATCACTCCCACCCACAGATATCTGCGTCTTCTCAGATGGC

TCCGAGCAGTGGCAGGAGGGCATCAAGTACGTAGGCTATGGCTTCGTACTTATGGTAAAC

GGCACCCAAATCGACACTGGCGCTGGCGCTATCAACTCACGCTCTCATGTTTTCGACGCC

GAAGCAATCGGAGCCTGGCGAGGGCTCGAACGGGCAATCGCGGTAGCTCCGCCTAGGTCG

AAAATCTGGCTCTGTATTGATAGCACATCCGTTATCTGGTGTATTAGGGGGAATGCCTCG

AACTCCTCGCAATGGGCATTTTTAGCCTGCCACCGGGCTATGGAACAGCACAATATCAGT

CTCCGATGGGCCCCTGGGCACACTGGGATCGAAGGGAACGAAGCTGCTGACACCTTAGCC

GGTGAAGGCGCGCTACGCGGTAGTGCTATAGGGATGGAAGCCGAACCCACGATTAGCGGG

ATCCGATCCATCTTCCGGGAACTTCGGAACGAGGCTCGCTTGCGCTGGTGGGACACGGTC

TCTCAAAAACTCTCCCAGTGGTACCGACGCTGGTCAGACACCTACGAGATTGATTCACTG

CCGGAACTCGAACTCCGACGACCAGCGCTCCACCGCTGGCTTGCCCTCCGCTCGTCGCAT

GGCGACTTCGACTGGTACCACCGCAAGTTCAACCACGAAGACGCCAAACTCGACTGCTCA

TGCGGCCGCCGAAAGTCACCAGAGCACCTCGCTCTCTGCCACAAAACCCAGAGGTCTTTC

CGACACTGGCCAAAACGCCCCCCGACACCTCCAACCGACAGGACAGAGGCAGTCGCCTAC

CTTCGCAGCCTGGACCCCAAGCAGTTTGTTGAACTACTGGAGCTCACAAGCTTCTACTCG

CGGGTCTGCACGAGG

>CL109.Contig23_All 43 2499 minus strand putative RNA-directed DNA polymerase from transposon X-element [Penicillium digitatum PHI26]

CTCACCACAACCTCCAGCCTTCCATGGCTACAATCTGTCACAATGGAAGAAGTTGAGGCT

AACACAATTGGCGTCTCCAGCACGTCGCCTGGCTCAGATAGGATAACTGTGCGCCTACTC

AAAGCCTGCTGGGAACACCTCAAAGACATAGTCCTCTCTCTGTTTAACCGATGCCTCGCC

CTCTGCCACATCCCCCTGGCCTGGAAGGTCGCGGAGGTGGCTATGATACCCAAGGTCGGC

AAGAAGGATAAGTCCTCCGTGCGCTCCTGGAGACCGATCGCCCTCCTATCCTGTCTCTCT

AAGGGACTAGAACGAATCATCGCGAAAAGAATTGCCTGGACTGCCCTCACCCACGGCGTC

CTCAGCCCCCAGCATGCGGGGGCTCTACCCAAACTTTCCGCCACAGACCTAGTCGCCTCC

TTCACCCACGATGCCGAAATGGCACTCTCACAGAACAAGCAAGTCACCCTAGTCACGATG

GACGTCCAGGGTGCATTCGATGCCCTCCTCCGGAGACGACTTCTCAAGCGCATGGGCGAG

CAGGGCTGGCCACGAGAACTGCTACTTCTTGTGGACAGCTTTCTCACCGGACGCAAAGCC

CGCGTCCGGCTAGAAGGCTCAACCACCCCTGAGTACGACGTGGTCTGCGGAACCCCCCAA

GGCTCCCCATTATCACCAGTACTATATATGCTCTACCTAGCCGAACTCCTCAATATGGAC

CAAACGCTCCGTTTCGGGTATGCCGACGACCTAGCTCTATACCGAGCCTCCCACGATCTC

ACCCAGAATGTCAGACTCCTCGCTAAGGACGTTCAGAGCATTCTTGCCTGGGGAGAATAT

AATAAAGTGGCCTTCGCCCCCGAGAAACTAGAGATGATCCATATCACTAGACATCGAGGG

GATGAGTCCCCTTCAATTGTAGTCAACGACCGGCTCACCATCGACCCCGTTCAGGCCAAG

AAACCAGGATACACACCCACTCTCCGCTGGCTGGGAGTCTTTTTCGATAGAAAGCTCACA

TGGAGAAGCCACATTCTCGCCCGGGCGGGCAAGGCACGCGCTGTCGCACAACATATCCGC

AATCTGGCCCGCACGACCTGCGGCCCGCCCGCGAGCTCACTTCGCAAAGCAGTCATCACC

TGTGTTATACCCTCCCTAACGTTCGGAACTGAGGCCTGGTATGGCGGCCGGAATAGGCCC

GCAAAACAGGCTAGCAAGGGCACCGTCAGCGCCCGTGTTGGCTGGCATATCAATGTCATC

GAGTCGACCCTGGCACTCGCCATCCGCGGCGTCCTCCCTGTATGGCGCACCACACCAACT

CCCTCGCTCTTTAGGGATGCGGGAATCCCGTCTGGATACGCCACACTTGAAGAGGCGAAA

CTACGGTTCGCTCTAAGGCTTAACACCATCCACAAAGGTCACACCCTTGTCCGTCGCATT

CGACCCCCGATGATCACCCGAGGCCGCGGCACCGGCACCCGCCAACCGGCAAAGACAATT

ATCCAGAGACTTGGGAGCATCCTCCCGGAAGTCCCAAGACCGACCCTCTCCCCCCCGCAC

TACTCACCGGGCTGCAGAATAGATCCTACAGGGGGTATAGATAAGGCGACCGCGTCTAAA

GCTTTCCAAGTCTGGCAGGAATCACTCCCACCCACAGATATCTGCGTCTTCTCAGATGGC

TCCGAGCAGTGGCAGGAGGGCATCAAGTACGTAGGCTATGGCTTCGTACTTATGGTAAAC

GGCACCCAAATCGACACTGGCGCTGGCGCTATCAACTCACGCTCTCATGTTTTCGACGCC

GAAGCAATCGGAGCCTGGCGAGGGCTCGAACGGGCAATCGCGGTAGCTCCGCCTAGGTCG

AAAATCTGGCTCTGTATTGATAGCACATCCGTTATCTGGTGTATTAGGGGGAATGCCTCG

AACTCCTCGCAATGGGCATTTTTAGCCTGCCACCGGGCTATGGAACAGCACAATATCAGT

CTCCGATGGGCCCCTGGGCACACTGGGATCGAAGGGAACGAAGCTGCTGACACCTTAGCC

GGTGAAGGCGCGCTACGCGGTAGTGGCTGGTGGGACACGGTCTCTCAAAAACTCTCCCAG

TGGTACCGACGCTGGTCAGACACCTACGAGATTGATTCACTGCCGGAACTCGAACTCCGA

CGACCAGCGCTCCACCGCTGGCTTGCCCTCCGCTCGTCGCATGGCGACTTCGACTGGTAC

CACCGCAAGTTCAACCACGAAGACGCCAAACTCGACTGCTCATGCGGCCGCCGAAAGTCA

CCAGAGCACCTCGCTCTCTGCCACAAAACCCAGAGGTCTTTCCGACACTGGCCAAAACGC

CCCCCGACACCTCCAACCGACAGGACAGAGGCAGTCGCCTACCTTCGCAGCCTGGACCCC

AAGCAGTTTGTTGAACTACTGGAGCTCACAAGCTTCTACTCGCGGGTCTGCACGAGG

>CL109.Contig24_All 17 2554 putative RNA-directed DNA polymerase from transposon X-element [Penicillium digitatum PHI26]

ATGAGTGCGGTGGAAGTTTGACAAGCACCTTGGGTTGGCTATTTCCATATTCCAGAGGAA

TGTAGGTGTAATCAGTATCCTATCACCACAACCTCCAGCCTTCCATGGCTACAATCTGTC

ACAATGGAAGAAGTTGAGGCTAACACAATTGGCGTCTCCAGCACGTCGCCTGGCTCAGAT

AGGATAACTGTGCGCCTACTCAAAGCCTGCTGGGAACACCTCAAAGACATAGTCCTCTCT

CTGTTTAACCGATGCCTCGCCCTCTGCCACATCCCCCTAGCCTGGAAGGTCGCGGAGGTG

GCTATGATACCCAAGGTCGGCAAGAAGGATAAGTCCTCCGTGCGCTCCTGGAGACCGATC

GCCCTCCTATCCTGTCTCTCTAAGGGACTAGAACGAATCGTCGCGAAAAGAATTGCCTGG

ACTGCCCTCACCCACGGCGTCCTCAGCCCCCAGCATGCGGGGGCTCTACCCAAACTTTCC

GCCACAGACCTAGTCGCCTCCTTCACCCACGATGCCGAAATGGCACTCTCACAGAACAAG

CAAGTCACCCTAGTCACGATGGACGTCCAGGGTGCATTCGATGCCCTCCTCCGGAGACGA

CTTCTCAAGCGCATGGGCGAGCAGGGCTGGCCACGAGAACTGCTACTTCTTGTGGACAGC

TTTCTCACCGGACGCAAAGCCCGCGTCCGGCTAGAAGGCTCAACCACCCCTGAGTACGAC

GTGGTCTGCGGAACCCCCCAAGGCTCCCCATTATCACCAGTACTATATATGCTCTACCTA

GCCGAACTCCTCAATATGGACCAAACGCTCCGTTTCGGGTATGCCGACGACCTAGCTCTA

TACAGAGCCTCCCACGATCTCACCCAGAATGTCAGACTCCTCGCTAAGGACGTTCAGAGC

ATTCTTGCCTGGGGAGAATATAATAAAGTGGCCTTCGCCCCCGAGAAACTAGAGATGATC

CATATCACTAGACATCGAGGGGATGAGTCCCCTTCAATTGTAGTCAACGACCGGCTCACC

ATCGACCCCGTTCAGGCCAAGAAACCAGGATACACACCCACTCTCCGCTGGCTGGGAGTC

TTTTTCGATAGAAAGCTCACATGGAGAAGCCACATTCTCGCCCGGGCGGGCAAGGCACGC

GCTGTCGCACAACATATCCGCAATCTGGCCCGCACGACCTGCGGCCCGCCCGCGAGCTCA

CTTCGCAAAGCAGTCATCACCTGTGTTATACCCTCCCTAACGTTCGGAACTGAGGCCTGG

TATGGCGGCCGGAATAGGCCCGCAAAACAGGCTAGCAAGGGCACCGTCAGCGCCCGTGTT

GGCTGGCATATCAATGTCATCGAGTCGACCCTGGCACTCGCCATCCGCGGCGTCCTCCCT

GTATGGCGCACCACACCAACTCCATCGCTCTTTAGGGACGCGGGAATCCCGTCTGGATAC

GCCACACTTGAAGAGGCGAAACTACGGTTCGCTCTAAGGCTTAACACCATCCACAAAGGT

CACACCCTTGTCCGTCGCATTCGACCCCCGATGATCACCCGAGGCCGCGGCACCGGCACC

CGCCAACCGGCAAAGACAATTATCCAGAGACTTGGGAGCATCCTCCCGGAAGTCCCAAGA

CCGACCCTCTCCCCCCCGCACTACTCACCGGGCTGCAGAATAGATCCTACAGGGGGTATA

GATAAGGCGACCGCGTCTAAAGCTTTCCAAGTCTGGCAGGAATCACTCCCACCCACAGAT

ATCTGCGTCTTCTCAGATGGCTCCGAGCAGTGGCAGGAGGGCATCAAGTACGTAGGCTAT

GGCTTCGTACTTATGGTAAACGGCACCCAAATCGACACTGGCGCTGGCGCTATCAACTCA

CGCTCTCATGTTTTCGACGCCGAAGCAATCGGAGCCTGGCGAGGGCTCGAACGGGCAATC

GCGGTAGCTCCGCCTAGGTCGAAAATCTGGCTCTGTATTGATAGCACATCCGTTATCTGG

TGTATTAGGGGGAATGCCTCTAACTCCTCGCAATGGGCATTTTTAGCCTGCCACCGGGCT

ATGGAACAGCACAATATCAGTCTCCGATGGGCCCCTGGGCACACTGGGATCGAAGGGAAC

GAAGCTGCTGACACCTTAGCCGGTGAAGGCGCGCTACGCGGTAGTGGCTGGTGGGACACG

GTCTCTCAAAAACTCTCCCAGTGGTACCGACGCTGGTCAGACACCTACGAGATTGATTCA

CTGCCGGAACTCGAACTCCGACGACCAGCGCTCCACCGCTGGCTTGCCCTCCGCTCGTCG

CATGGCGACTTCGACTGGTACCACCGCAAGTTCAACCACGAAGACGCCAAACTCGACTGC

TCATGCGGCCGCCGAAAGTCACCAGAGCACCTCGCTCTCTGCCACAAAACCCAGAGGTCT

TTCCGACACTGGCCAAAACGCCCCCCGACACCTCCAACCGACAGGACAGAGGCAGTCGCC

TACCTTCGCAGCCTGGACCCCAAGCAGTTTGTTGAACTACTGGAGCTCACAAGCTTCTAC

TCGCGGGTCTGCACGAGG

>CL109.Contig25_All 17 2632 putative RNA-directed DNA polymerase from transposon X-element [Penicillium digitatum PHI26]

ATGAGTGCGGTGGAAGTTTGACAAGCACCTTGGGTTGGCTATTTCCATATTCCAGAGGAA

TGTAGGTGTAATCAGTATCCTATCACCACAACCTCCAGCCTTCCATGGCTACAATCTGTC

ACAATGGAAGAAGTTGAGGCTAACACAATTGGCGTCTCCAGCACGTCGCCTGGCTCAGAT

AGGATAACTGTGCGCCTACTCAAAGCCTGCTGGGAACACCTCAAAGACATAGTCCTCTCT

CTGTTTAACCGATGCCTCGCCCTCTGCCACATCCCCCTAGCCTGGAAGGTCGCGGAGGTG

GCTATGATACCCAAGGTCGGCAAGAAGGATAAGTCCTCCGTGCGCTCCTGGAGACCGATC

GCCCTCCTATCCTGTCTCTCTAAGGGACTAGAACGAATCGTCGCGAAAAGAATTGCCTGG

ACTGCCCTCACCCACGGCGTCCTCAGCCCCCAGCATGCGGGGGCTCTACCCAAACTTTCC

GCCACAGACCTAGTCGCCTCCTTCACCCACGATGCCGAAATGGCACTCTCACAGAACAAG

CAAGTCACCCTAGTCACGATGGACGTCCAGGGTGCATTCGATGCCCTCCTCCGGAGACGA

CTTCTCAAGCGCATGGGCGAGCAGGGCTGGCCACGAGAACTGCTACTTCTTGTGGACAGC

TTTCTCACCGGACGCAAAGCCCGCGTCCGGCTAGAAGGCTCAACCACCCCTGAGTACGAC

GTGGTCTGCGGAACCCCCCAAGGCTCCCCATTATCACCAGTACTATATATGCTCTACCTA

GCCGAACTCCTCAATATGGACCAAACGCTCCGTTTCGGGTATGCCGACGACCTAGCTCTA

TACAGAGCCTCCCACGATCTCACCCAGAATGTCAGACTCCTCGCTAAGGACGTTCAGAGC

ATTCTTGCCTGGGGAGAATATAATAAAGTGGCCTTCGCCCCCGAGAAACTAGAGATGATC

CATATCACTAGACATCGAGGGGATGAGTCCCCTTCAATTGTAGTCAACGACCGGCTCACC

ATCGACCCCGTTCAGGCCAAGAAACCAGGATACACACCCACTCTCCGCTGGCTGGGAGTC

TTTTTCGATAGAAAGCTCACATGGAGAAGCCACATTCTCGCCCGGGCGGGCAAGGCACGC

GCTGTCGCACAACATATCCGCAATCTGGCCCGCACGACCTGCGGCCCGCCCGCGAGCTCA

CTTCGCAAAGCAGTCATCACCTGTGTTATACCCTCCCTAACGTTCGGAACTGAGGCCTGG

TATGGCGGCCGGAATAGGCCCGCAAAACAGGCTAGCAAGGGCACCGTCAGCGCCCGTGTT

GGCTGGCATATCAATGTCATCGAGTCGACCCTGGCACTCGCCATCCGCGGCGTCCTCCCT

GTATGGCGCACCACACCAACTCCATCGCTCTTTAGGGACGCGGGAATCCCGTCTGGATAC

GCCACACTTGAAGAGGCGAAACTACGGTTCGCTCTAAGGCTTAACACCATCCACAAAGGT

CACACCCTTGTCCGTCGCATTCGACCCCCGATGATCACCCGAGGCCGCGGCACCGGCACC

CGCCAACCGGCAAAGACAATTATCCAGAGACTTGGGAGCATCCTCCCGGAAGTCCCAAGA

CCGACCCTCTCCCCCCCGCACTACTCACCGGGCTGCAGAATAGATCCTACAGGGGGTATA

GATAAGGCGACCGCGTCTAAAGCTTTCCAAGTCTGGCAGGAATCACTCCCACCCACAGAT

ATCTGCGTCTTCTCAGATGGCTCCGAGCAGTGGCAGGAGGGCATCAAGTACGTAGGCTAT

GGCTTCGTACTTATGGTAAACGGCACCCAAATCGACACTGGCGCTGGCGCTATCAACTCA

CGCTCTCATGTTTTCGACGCCGAAGCAATCGGAGCCTGGCGAGGGCTCGAACGGGCAATC

GCGGTAGCTCCGCCTAGGTCGAAAATCTGGCTCTGTATTGATAGCACATCCGTTATCTGG

TGTATTAGGGGGAATGCCTCTAACTCCTCGCAATGGGCATTTTTAGCCTGCCACCGGGCT

ATGGAACAGCACAATATCAGTCTCCGATGGGCCCCTGGGCACACTGGGATCGAAGGGAAC

GAAGCTGCTGACACCTTAGCCGGTGAAGGCGCGCTACGCGGTAGTGCTATAGGGATGGAA

GCCGAACCCACGATTAGCGGGATCCGATCCATCTTCCGGGAACTTCGGAACGAGGCTCGC

TTGCGCTGGTGGGACACGGTCTCTCAAAAACTCTCCCAGTGGTACCGACGCTGGTCAGAC

ACCTACGAGATTGATTCACTGCCGGAACTCGAACTCCGACGACCAGCGCTCCACCGCTGG

CTTGCCCTCCGCTCGTCGCATGGCGACTTCGACTGGTACCACCGCAAGTTCAACCACGAA

GACGCCAAACTCGACTGCTCATGCGGCCGCCGAAAGTCACCAGAGCACCTCGCTCTCTGC

CACAAAACCCAGAGGTCTTTCCGACACTGGCCAAAACGCCCCCCGACACCTCCAACCGAC

AGGACAGAGGCAGTCGCCTACCTTCGCAGCCTGGACCCCAAGCAGTTTGTTGAACTACTG

GAGCTCACAAGCTTCTACTCGCGGGTCTGCACGAGG

>CL110.Contig1_All 3 899 minus strand MFS alpha-glucoside transporter, putative [Penicillium digitatum Pd1] >gi|425776765|gb|EKV14971.1| MFS alpha-glucoside transporter, putative [Penicillium digitatum PHI26]

ACAGAGAAGGAGCATTCCATGACACTGATGCAGGGAATCCGGTTATATCCTAAGGCCGTA

GTTTGGAGTGTGCTCATCTCTACTTGCATTGCCATGGAGGGTTATGACATTAGTTTAGTC

AACAACTTCTATGCATTCCCGCAATTCAACCGCAAATATGGGGAGCGACTAGCCAATGGC

ACATATGAAGTATCCGCAGCGTGGCAAGCAGGTCTGAGCAACGGTGCTTACTGCGGCGAA

ATCATCGGTTTATTCATCAACGGTTGGGCTTCGGAACGCTTCGGTTACCGGTACACGATT

ATGGCTTGCCTCGTTCTTGTCAGCGCGTGGACGTCAATCTTCTTCACGGCCAAGAATGTG

CAATCCTTGCTTGTAGCGGAGATTCTCTGTGGTATTCCCTGGGGTGTTTTCCAGACTTTG

ACGATTACCTACGCTTCGGAGGTGTGTCCCGTGGCACTGCGAGGATATTTGACGACCTAT

GTCAATTTCTGCTGGGGCCTTGGTCAGCTGATTGGCATTGGGGTGATTAAGGGCATGTTG

AGCCGGACCGACGAATGGGCCTACCGGATTCCGTACGGGCTGCAATGGATGTGGCCGCTG

CCACTCTTCACTGGAATTTGGCTGGCTCCCGAATCTCCGTGGTGGTTGGTTCGGAAAGGC

CGCACGGAAGATGCGAAACGGTCCCTGATACGCTTGACCAGCAAAAGTCAAGGGGACTTC

GATCCCGATGAGACGATCTCGATGATGGTTCACACTACTGCTCTGGAGGAAAAGATTACC

AAAGGTGCGAGCTACCTGGATTGCTTCCGGGGTACCGATCGACGCCGCACAGAGATTGTC

TGTATGGTCTGGGCGATTCAGAATCTTAGTGGAAACTCCTTTTCCAATTACTCGACC

>CL110.Contig2_All 293 412 minus strand MFS alpha-glucoside transporter, putative [Penicillium digitatum Pd1] >gi|425776765|gb|EKV14971.1| MFS alpha-glucoside transporter, putative [Penicillium digitatum PHI26]

CAGTGGCAAGCAGGTCTGAGCAACGGTGCTTACTGCGGCGAAATCATCGGTTTATTCATC

AACGGTTGGGCTTCGGAACGCTTCGGTTACCGGTACACGATTATGGCTTTCCTCGTTCTT

>CL111.Contig1_All 228 1547 minus strand hypothetical protein PDIP_66590 [Penicillium digitatum Pd1] >gi|425771988|gb|EKV10416.1| hypothetical protein PDIG_57050 [Penicillium digitatum PHI26]

ATGACACACACCGTTCCCCCCGACCAGCCTATTGACCTCCTAACCGGCTGGCCCAACCCC

GCGCTCCTCCCCGCGCTCAATCTGCGCCACTCCGCCACAACCGTACTCTCCAATCCCTCC

CTCGCAAACCCTGCCCTTCTCTACGGTCCCGATGAAGGATACGAGCCGCTGCGCACACAC

ATCGCTGCATGGCTGAGCGATTTCTACCAGCAGCGCGAGCCCATCTCAACACAGCGCATT

TGTATCACCGGCGGGGCGAGCCAGAACCTAGCTTGCGTGCTGCAAACCTTCACAGATCCG

GTCTACACACGAAATGTATGGATGGTCGTACCGACATACCACCTGGCTGCGCGCATCATG

GACGACGCCGGCTTCGCCGGGCGTCTACGTGGAATCCCCGAAGATGACGAGGGTGTTGAC

ATAGCGGTGCTGGAGAACGGGTTGCGCGCCGCGGAAAAGATTGCTCTGCGCGATGGAAAT

ACTGAGCCGAAAATGAAACCGCCTAGGCCCTGGCGCAAGATCTACAAGCATATCATCTAT

GCTGTGCCGACTTTCGCAAATCCCTCAGGGAAGATCATGTCGCTGCATAGACGTGAAGCG

CTCGTTCGCCTCGCCCGCCAGTACGATGCACTGATCGTCACGGATGATGTGTACGATTTC

CTTTGGTGGTCGGCCACGCCAGGAGAGGAATTCTACGGTAACCGTGCGTGTAGTCCGCGC

CTTATCGACGTGGACCGCTACTTGGACGGTGGTCCACTGGATGAATGGGGACACACAATA

AGCAACGGTAGTTTCAGCAAGCTGATCGGACCTGGGGCCCGTACCGGCTGGGCCGAGGCG

TCGGAAAGAGTTGCGTATGGATTATCACAAACAGGCTCTTCCCGATCGGGCGGCGCTCCT

TCCCATCTAACCGCCGCTATCATTGACCAGATGTTCCCCACAGGCGTACCAAACCACATC

CGTAACGTCCTCCAACCGAAATACGCCGAGCGCTACCACACTTTGCTATCGGCAGTCCTC

GAGCATCTCGTCCCGCTGGGCGTCACAGTCCCAGCACCGGGCCCCGCCGCCGGAGGATAC

TTTATGTGGATCGGTCTACCGGCGCCGTTGATCGCCGCCGACGTAGTCCAGCTCGCGCAG

AGCGACGAAAAACTGCGTGTGTCACCTGGTCATGTGTTCCAGGTCCCCGGAGACCGGGTG

ATTGATGAGGGGTTTGCGGATCATCTACGCCTCTGTTTTGCGTGGGAAGAGCCACCTCAT

TTGACCGAGGGGATACGTAGGCTAGCGCGTGTTCTCAATCGTACGACTCGTACCCAGGTC

>CL111.Contig2_All 1193 1639 minus strand hypothetical protein PDIP_66590 [Penicillium digitatum Pd1] >gi|425771988|gb|EKV10416.1| hypothetical protein PDIG_57050 [Penicillium digitatum PHI26]

GGCTCTTCCCGATCGGGCGGCGCTCCTTCCCATCTAACCGCCGCTATCATTGACCAGATG

TTCCCCACAGGCGTACCAAACCACATCCGTAACGTCCTCCAACCGAAATACGCCGAGCGC

TACCACACTTTGCTATCGGCAGTCCTCGAGCATCTCGTCCCGCTGGGCGTCACAGTCCCA

GCACCGGGCCCCGCCGCCGGAGGATACTTTATGTGGATCGGTCTACCGGCGCCGTTGATC

GCCGCCGACGTAGTCCAGCTCGCGCAGAGCGACGAAAAACTGCGTGTGTCACCTGGTCAT

GTGTTCCAGGTCCCCGGAGACCGGGTGATTGATGAGGGGTTTGCGGATCATCTACGCCTC

TGTTTTGCGTGGGAAGAGCCACCTCATTTGACCGAGGGGATACGTAGGCTAGCGCGTGTT

CTCAATCGTACGACTCGTACCCAGGTC

>CL111.Contig3_All 259 678 minus strand ---

GACCAGCCTATTGACCTCCTAACCGGCTGGCCCAACCCCGCGCTCCTCCCCGCGCTCAAT

CTGCGCCACTCCGCCACAACCGTACTCTCCAATCCCTCCCTCGCAAACCCTGCCCTTCTC

TACGGTCCCGATGAAGGATACGAGCCGCTGCGCACACACATCGCTGCATGGCTGAGCGAT

TTCTACCAGCAGCGCGAGCCCATCTCAACACAGCGCATTTGTATCACCGGCGGGGCGAGC

CAGAACCTAGCTTGCGTGCTGCAAACCTTCACAGATCCGGTCTACACACGAAATGTATGG

ATGGTCGTACCGACATACCACCTGGCTGCGCGCATCATGGACGACGCCGGCTTCGCCGGG

CGTCTACGTGGAATCCCCGAAGATGACGAGGGTGTTGACATAGCGGTGCTGGAGAACGGG

>CL112.Contig1_All 2 433 minus strand C6 transcription factor, putative [Penicillium digitatum PHI26] >gi|425783401|gb|EKV21254.1| C6 transcription factor, putative [Penicillium digitatum Pd1]

CCCGAATCACTCCTATCACTCGTAGAAACGGTCTTAGTTGCCTTTGAAGAGCGCTCTCTT

TTGATTACAAAGGGGGCAAAAGACTACTCTTTCCTCTCAACGATCGTGGCACTAGTCAAA

TCCCGACTCTGGCCGGCACAGGCAACCATGTACAAGCAGCAGGTCGTCAAGCGGGTCCTG

TCGTTTGCGCAGATTCTCTTCGATCGGCACATGAACTGTGATCACCTCGGCGACCAGGGG

ATGGGCAGTTTCAAAAACAACCAGCTGGCCGCATTCATGGCGGCTCCAACAATGGTGCCT

GTGATGCAGCCCGAATTTGATCCCAATGGGATTCAACCGCCGCCGCAGCCTGGCGTGATA

TCGCCTGGCGAGTTCGATCCGTTCTTGGAGATCTTTGATTGGGAGGATCTGACAAGCATG

ACGTTTCCCTGT

>CL112.Contig2_All 13 2493 minus strand C6 transcription factor, putative [Penicillium digitatum PHI26] >gi|425783401|gb|EKV21254.1| C6 transcription factor, putative [Penicillium digitatum Pd1]

ATGGATGTTCAAGGGAGCGCACAGCCTGACCGCGTGCGCAAGCGCCGCCGCCGCACAATG

GCGTGCACGCAGTGTCGCACGCGTAAGTTGCGATGCGATCGCGAGTATCCTAGTTGCAGT

CGCTGTTTGAAGAGCAGAACCCCTAGCAAATGCACATATGAAGATGGCTTTTTATGGCAG

CAGCCTAGCACAGTCACAACCACTGCATTTGCTCCTGACCGAGGGACCGTGGTATCTATG

CCCCGAGATACCCCAGTCGACACACCTCGGGATTCGGGGATACCGACGGGGTCAACTCGG

ACAGAATCTTTCCCAACTAGTGAACCACCTCGCAGGGCTATGGCCGGCGGTCCAATGCAT

CACGAATATCATGGTCTTATGGGGCATGCTCTTGCGCCGCCCTACAGAGGTCAGCCACAT

GACAGAGAGCGAAAGGACTGTTTTTTGGAAACCGTTCTCGGTGCTCCTAAGGCTGCTGTT

AATCAGGAAACCTACGTAAACACGAGTATTTTACATCGTCCCAAGCGCCTAGCACCTGCA

TCGGAGCTGGGTATGCAAATACCGTCGCAGGTCGACGATGCGCACGTTGACGAAGAAGAA

GCCCCATTGTCGCCTACGGATCAGCTGGACCTCGCCCCTCGCATGATGATGAGGGGCCGT

GAAACTAAAACACGATTTAGTGGATCGGGCATCTTTGCTAGTCTAGTAGCACAGTTTCCG

GATATTAAGTCATTTGCAGAGGAGATCAGGCTTTCTGCCCCGATCTTCACCCAACTTCAG

CCGGATCTAAGAAGAGTCAAGGGCGGTCTATTCAAACTTATGGTGCTCAATCAATCCTTT

CCCGACCCTACTACCGCTTCACTAATAAACTTGCTACCCTCGCGTGCTGTGGTTGATGAG

CTGGTTCGGTTGTATATCACTCATATCGAGTTCACTCACCGCATTCTTCACGTTCCATCT

TTCTTACGAGAGCTTGACCAATTCTGGGCCATGTTAGACAGCCCGGCCTCAACATCTGCT

GCATTTGCAGTTCAACTATTATTGGTCCTTGCCTGTGCATGGAACCTTGCAGACCTTGCT

AGCCTGCAGGCAAAAAGTGCGGGCGAACTTAAGTGCCAGTCTGCGGTGGAGTGGACATTA

CACGCAGGAAAATGGATTGAGAATCTCCACACCAAGCGTCCTGAAATTAACTCACTGCGA

CTCTCTATTTTATTGATTTTGGCGCACAACTCCCACGGAATGAAACGCAGTCAGGCTTGG

CTCGCAACAAGCACATTGGTTAAGCAAGCCATGATGGCAGGCTACCACCGCGACCCAAGT

CGTTACACACGGATCTCTGTATTCAACAAGGAGATGCGACGGCGAATCTGGATAACCATC

GTGGAAATGGACCTGCAAATTGCTCTTGATCGGGGAATGCCGCCGTCAGTACAAACCTTT

GACTATGATGTAGCTCCGGGGCTGAACATTTTCGACGACGAGATCCACGAGAACAGCACT

GAAGCCCCTGAAAGCCGGCCACTGAGTGAAGTTACCGACTCATCGTTCCAGTCTATCCTG

TGTTGCTCGCTACCTCTTCGTCTTCAAGCTTGTTCCCTCATGCACTCCCCACGGATCAGC

TGTCGATATGAGGATATTCAGCGCCTGGACGGCGAGCTCAATCGGCATCTTTCTCGGATT

CCCGCGTGGACAACAGCCGAAACCAACGATATCGTCACCCAACATAAAGTAATATTGTGG

AAAGGGTTGATTGAGACCAAACTTTGCCAATCTCTCCTGTCTGTCCACACCCCTTTTGCC

ATTGAAGCACGGCGAGAATCACTTTTTGCACCCTCCGCGCGTACTCGCATGGATGCCGCA

ACCAGGATACTGTCAAGCCAGCGCCGGCTAAACGAGATCTCGCGAACTCTATCGTTATGT

ACGCTTGGTGAATGGACCATCCAAGCCTACATTTCCATCTGCCAATCGCTACACACAACC

GAATTCGACACCTCTTCCCCTTCCCATCCCCTCTCGTCAAGCTTTAATCTCCACAATATT

CCCGGTATCCCCGAATCACTCCTATCACTCGTAGAAACGGTCTTAGTTGCCTTTGAAGAG

CGCTCTCTTTTGATTACAAAGGGGGCAAAAGACTACTCTTTCCTCTCAACGATCGTGGCA

CTAGTCAAATCCCGACTCTGGCCGGCACAGGCAACCATGTACAAGCAGCAGGTCGTCAAG

CGGGTCCTGTCGTTTGCGCAGATTCTCTTCGATCGGCACATGAACTGTGATCACCTCGGC

GACCAGGGGATGGGCAGTTTCAAAAACAACCAGCTGGCCGCATTCATGGCGGCTCCAACA

ATGGTGCCTGTGATGCAGCCCGAATTTGATCCCAATGGGATTCAACCGCCGCCGCAGCCT

GGCGTGATATCGCCTGGCGAGTTCGATCCGTTCTTGGAGATCTTTGATTGGGAGGATCTG

ACAAGCATGACGTTTCCCTGT

>CL113.Contig1_All 183 314 minus strand hypothetical protein PDIG_43640 [Penicillium digitatum PHI26] >gi|425778576|gb|EKV16700.1| hypothetical protein PDIP_34870 [Penicillium digitatum Pd1]

ATGAACTCCAAGAGGGACCGCGTCTCCACCCAACGACTCTCTCGCTCCATTTCTCGCGGA

AATGGCCCTGTACCTCATGTGTCGGACTCCCGACGTGTCCTAGAAGATTCGTGGGTCGAA

AGTAGTTTTCAG

>CL113.Contig2_All 187 375 minus strand hypothetical protein PDIG_43640 [Penicillium digitatum PHI26] >gi|425778576|gb|EKV16700.1| hypothetical protein PDIP_34870 [Penicillium digitatum Pd1]

ATGAACTCCAAGAGGGACCGCGTCTCCACCCAACGACTCTCTCGCTCCATTTCTCGCGGA

AATGGCCCTGTACCTCATGTGTCGGACTCCCGACGTGTCCTAGAAGATTCGTGGGTCGAA

AGTAGTTTTCAGGTATTGCCGCATCAGAGAGACATCGATCCTCTAAACCGCTACGTCTTA

TTGAGGTTG

>CL113.Contig3_All 183 314 hypothetical protein PDIG_43640 [Penicillium digitatum PHI26] >gi|425778576|gb|EKV16700.1| hypothetical protein PDIP_34870 [Penicillium digitatum Pd1]

ATGAACTCCAAGAGGGACCGCGTCTCCACCCAACGACTCTCTCGCTCCATTTCTCGCGGA

AATGGCCCTGTACCTCATGTGTCGGACTCCCGACGTGTCCTAGAAGATTCGTGGGTCGAA

AGTAGTTTTCAG

>CL113.Contig4_All 187 318 hypothetical protein PDIG_43640 [Penicillium digitatum PHI26] >gi|425778576|gb|EKV16700.1| hypothetical protein PDIP_34870 [Penicillium digitatum Pd1]

ATGAACTCCAAGAGGGACCGCGTCTCCACCCAACGACTCTCTCGCTCCATTTCTCGCGGA

AATGGCCCTGTACCTCATGTGTCGGACTCCCGACGTGTCCTAGAAGATTCGTGGGTCGAA

AGTAGTTTTCAG

>CL113.Contig5_All 187 846 minus strand hypothetical protein PDIG_43640 [Penicillium digitatum PHI26] >gi|425778576|gb|EKV16700.1| hypothetical protein PDIP_34870 [Penicillium digitatum Pd1]

ATGAACTCCAAGAGGGACCGCGTCTCCACCCAACGACTCTCTCGCTCCATTTCTCGCGGA

AATGGCCCTGTACCTCATGTGTCGGACTCCCGACGTGTCCTAGAAGATTCGTGGGTCGAA

AGTAGTTTTCAGTCCGTTGACGCCACTCATTATGCTCTGCAAACCCTCAACAAGCACATC

GAACGAAGACTCGCCAAACTACAAGCTAATACAGTCCGCCTGAAGCGTGAACTATGGCTA

CTCCAGCGACACATCAAAGAATTTCGCCATCCTCTTTTCGAGAACTGGGAAGCCGACCTA

CTCACACGTCTTATTGAGGTTGCTCACGCCCATCAGCACAAGAAGCTCCCGGGCGGCGTC

GTGATTGGAGAGTCCTCCTTTGCCGAACGAGAATCTCTCAACCATGCATACAGCATCGCG

GCGAAGGGAATCCGCATGCCGACTCTTCGTAAGCTTGGTCTATCGGAGAAGTATCATCAG

GCTCTGCAGCGGTATTCAGAGGTCGCTCCTTATCGAAGCCCCAATCCCTTCCAAACCGAA

TTTGCATTCGCCAAATGGCTGGTCGAGGTGCGGGAAGATAGACCGGAGCTGTATTGTTTT

TGGTCCAAGCTGTTCCCCGTCTGCTACGACCGCACCGTCCAAGAGAGCGCCTCCATTTTC

>CL113.Contig6_All 187 375 minus strand hypothetical protein PDIG_43640 [Penicillium digitatum PHI26] >gi|425778576|gb|EKV16700.1| hypothetical protein PDIP_34870 [Penicillium digitatum Pd1]

ATGAACTCCAAGAGGGACCGCGTCTCCACCCAACGACTCTCTCGCTCCATTTCTCGCGGA

AATGGCCCTGTACCTCATGTGTCGGACTCCCGACGTGTCCTAGAAGATTCGTGGGTCGAA

AGTAGTTTTCAGGTATTGCCGCATCAGAGAGACATCGATCCTCTAAACCGCTACGTCTTA

TTGAGGTTG

>CL113.Contig7_All 187 318 minus strand hypothetical protein PDIG_43640 [Penicillium digitatum PHI26] >gi|425778576|gb|EKV16700.1| hypothetical protein PDIP_34870 [Penicillium digitatum Pd1]

ATGAACTCCAAGAGGGACCGCGTCTCCACCCAACGACTCTCTCGCTCCATTTCTCGCGGA

AATGGCCCTGTACCTCATGTGTCGGACTCCCGACGTGTCCTAGAAGATTCGTGGGTCGAA

AGTAGTTTTCAG

>CL113.Contig8_All 674 835 minus strand hypothetical protein PDIG_43640 [Penicillium digitatum PHI26] >gi|425778576|gb|EKV16700.1| hypothetical protein PDIP_34870 [Penicillium digitatum Pd1]

CAGGTCGCTCCTTATCGAAGCCCCAATCCCTTCCAAACCGAATTTGCATTCGCCAAATGG

CTGGTCGAGGTGCGGGAAGATAGACCGGAGCTGTATTGTTTTTGGTCCAAGCTGTTCCCC

GTCTGCTACGACCGCACCGTCCAAGAGAGCGCCTCCATTTTC

>CL113.Contig9_All 183 773 hypothetical protein PDIG_43640 [Penicillium digitatum PHI26] >gi|425778576|gb|EKV16700.1| hypothetical protein PDIP_34870 [Penicillium digitatum Pd1]

ATGAACTCCAAGAGGGACCGCGTCTCCACCCAACGACTCTCTCGCTCCATTTCTCGCGGA

AATGGCCCTGTACCTCATGTGTCGGACTCCCGACGTGTCCTAGAAGATTCGTGGGTCGAA

AGTAGTTTTCAGGTATTGCCGCATCAGAGAGACATCGATCCTCTAAACCGCTACGTCTTA

TTGAGGTTGCTCACGCCCATCAGCACAAGAAGCTCCCGGGCGGCGTCGTGATTGGAGAGT

CCTCCTTTGCCGAACGAGAATCTCTCAACCATGCATACAGCATCGCGGCGAAGGGAATCC

GCATGCCGACTCTTCGTAAGCTTGGTCTATCGGAGAAGTATCATCAGGCTCTGCAGCGGT

ATTCAGAGGTGAGTTCTCACCGTGGTCTTGATAGAACGTACCTAGGTCTAGTCACGTACT

TATGTGGATCAGGTCGCTCCTTATCGAAGCCCCAATCCCTTCCAAACCGAATTTGCATTC

GCCAAATGGCTGGTCGAGGTGCGGGAAGATAGACCGGAGCTGTATTGTTTTTGGTCCAAG

CTGTTCCCCGTCTGCTACGACCGCACCGTCCAAGAGAGCGCCTCCATTTTC

>CL113.Contig10_All 183 314 hypothetical protein PDIG_43640 [Penicillium digitatum PHI26] >gi|425778576|gb|EKV16700.1| hypothetical protein PDIP_34870 [Penicillium digitatum Pd1]

ATGAACTCCAAGAGGGACCGCGTCTCCACCCAACGACTCTCTCGCTCCATTTCTCGCGGA

AATGGCCCTGTACCTCATGTGTCGGACTCCCGACGTGTCCTAGAAGATTCGTGGGTCGAA

AGTAGTTTTCAG

>CL113.Contig11_All 183 314 hypothetical protein PDIG_43640 [Penicillium digitatum PHI26] >gi|425778576|gb|EKV16700.1| hypothetical protein PDIP_34870 [Penicillium digitatum Pd1]

ATGAACTCCAAGAGGGACCGCGTCTCCACCCAACGACTCTCTCGCTCCATTTCTCGCGGA

AATGGCCCTGTACCTCATGTGTCGGACTCCCGACGTGTCCTAGAAGATTCGTGGGTCGAA

AGTAGTTTTCAG

>CL113.Contig12_All 183 773 hypothetical protein PDIG_43640 [Penicillium digitatum PHI26] >gi|425778576|gb|EKV16700.1| hypothetical protein PDIP_34870 [Penicillium digitatum Pd1]

ATGAACTCCAAGAGGGACCGCGTCTCCACCCAACGACTCTCTCGCTCCATTTCTCGCGGA

AATGGCCCTGTACCTCATGTGTCGGACTCCCGACGTGTCCTAGAAGATTCGTGGGTCGAA

AGTAGTTTTCAGGTATTGCCGCATCAGAGAGACATCGATCCTCTAAACCGCTACGTCTTA

TTGAGGTTGCTCACGCCCATCAGCACAAGAAGCTCCCGGGCGGCGTCGTGATTGGAGAGT

CCTCCTTTGCCGAACGAGAATCTCTCAACCATGCATACAGCATCGCGGCGAAGGGAATCC

GCATGCCGACTCTTCGTAAGCTTGGTCTATCGGAGAAGTATCATCAGGCTCTGCAGCGGT

ATTCAGAGGTGAGTTCTCACCGTGGTCTTGATAGAACGTACCTAGGTCTAGTCACGTACT

TATGTGGATCAGGTCGCTCCTTATCGAAGCCCCAATCCCTTCCAAACCGAATTTGCATTC

GCCAAATGGCTGGTCGAGGTGCGGGAAGATAGACCGGAGCTGTATTGTTTTTGGTCCAAG

CTGTTCCCCGTCTGCTACGACCGCACCGTCCAAGAGAGCGCCTCCATTTTC

>CL114.Contig1_All 202 1191 hypothetical protein PDIP_62950 [Penicillium digitatum Pd1] >gi|425776973|gb|EKV15170.1| hypothetical protein PDIG_28510 [Penicillium digitatum PHI26]

ATGGATCGCATCTCTGCTCCGTCCGTCCAGTACCCGTCGGGGCCCAACTCGCTACGGCAA

CCCGACCATTTGGCCCCGGTGACAAACTATCAAGATGGTCGCTCCTGGTCATTACAAGTG

GTCCAGCAGCCCATTCGAGCTCGTATGTGTGGATTTGGTGATAAGGATCGGAGACCCATC

ACTCCCCCACCGTGTATTCGTCTGATTGTGCGCGATGCGCAGACGGAGAAGGAAATTGAT

ATCAATGAGATTGATACCTCATTCTACGTGCTCACCGTTGACCTGTGGAATGCCGACGGT

ACCAATGAAGTCAACCTCGTCAAGCATTCCGCGACCTCCCCTTCCATCTCTACTGCCATG

TCCTCCTCTTACCCTCCCCCGCCGCAAAGTGTTTCTCCCACCTACCAAGGCTATAACCAG

AACCAGTACCCTGTGGGCTACCCACCGCAGATGAACAACTACTACGGTGGTCAGCAGGTG

GCGTACCAAAACCAATACGGCCAGCCGGTGACGTACCCACAATATTACTCCGGTGGTCCA

ATGCCACCGTCCATGTCGCCCGCGGCGCAACCTGTTTCCGGTGGACCGGGTGGCATGTTC

ACTCGCAATCTGATCGGAAGTCTCAGCGCCAGTGCTTTCCGGTTGACAGATCCAGACAAC

AAAATCGGCGTGTGGTTTATCCTGCAAGATTTGAGTGTACGGACAGAAGGCACTTTCCGT

CTGAAAATGAGCTTCGTTGACGTGGGCACGTCGTCTGCCGAGACCTCCAACGGCGCTCCG

GTCATCAATCACGGAACCGCACCCGTTCTAGCATCGGTTTTCTCTGAACCCTTCCAAGTC

TTCTCCGCCAAGAAGTTCCCCGGTGTCATTGAGAGCACCCAGCTCAGCAAGTGCTTTGCG

CTACAGGGTATCAAGATCCCCATTCGCAAGGATGGAGTAAAGGGTCCCCGCCGAGGTGGA

GATGGCGATGACGATGATGAAGGCGACTAC

>CL114.Contig2_All 57 242 minus strand Pc22g22290 [Penicillium chrysogenum Wisconsin 54-1255] >gi|211593141|emb|CAP99517.1| Pc22g22290 [Penicillium chrysogenum Wisconsin 54-1255]

ATGAAGTTCACACTCACCTCCCTCGCCCTCCCCCTCCTCGCCGCCTCGGCGCCAGTTAGC

GAGCCAGCCCCAAACCCAGCCTTCAGCGTCATGGCCATCCGCTCCGCCTCGCCTATCCAC

TACCTACAGCTGAACGCCGCCGGCCAGAAGTTCTACCTCGGCGGAAACACCGCCTCCTAC

TGCCCA

>CL116.Contig1_All 77 1600 minus strand hypothetical protein PDIP_82300 [Penicillium digitatum Pd1] >gi|425780208|gb|EKV18225.1| hypothetical protein PDIG_10790 [Penicillium digitatum PHI26]

ATGGATAACGACGTGGTAGCTCAGTTCACTGAAATTACCGGCTCCACGCCGGAGCTGGCT

ATTCAGTACCTGCAGATCACCGAGTTCCAAATCGAGCAGGCCATGCAGCTTTACTTTGAA

AGTGGAGGCGCACCCTTGACTGCTGAACCTTCTCGGCCCTCTCATCTGTCTGGAATCCCA

CACGATTCCGAGGTGGTGAATATTGACTCGGATACTGATGACGATACCCCCCAACACATC

GCCCCGCCAACATTCGATGACGACGAGGCTATGGCTCGACGCCTCCAGGAGCAAATGTAT

GGTGGTACAGGTGGCATAGAAAATGAAGTTGAAGTGAGAGCGCCGTTAGCGCGGACAACG

GAGACTTTGGTGGGCCCCGGGGCGGACTATGACAGTGGTGAAGACATGCACGCCAACATC

CTCAGCCAACTACGAGCTCGTGGTAGATCAGGCCGAGCCGGAATCTTCAACCAGCAAGAG

TCATCTTCCATATGGACGGGCAGTGATGAATCTCGTCGCGAAGCGCTTTCTGCCGCTACT

GGGGGCGCATCGGAAGCATCTTCCAAATCAAATCTGCTCGCCGAAATGTACCGTCCTCCA

TTTGAAATCATGTCACGACTGCCTTGGGATGTGGCGCGCGAGGAGGGAAAGGACACCGAG

AAATGGCTGCTTGTCAATGTCCAGGACGCATCAATCTTTGACTGTCAGGTTCTGAACCGT

GATTTGTGGAAGGACCGTGGGGTGCAAGATACCGTCAAGGAGCATTTCATTTTCCTGCAG

TACTCGAAAGATGACCCCCGTGCCTCTTCCTACCTCCAATACTACTTCCAGGGAAGCGAT

GTCTCCGACAACTACCCCCACATTGCTATCGTCGACCCTCGGACTGGTGAACAAGTGAAG

GTCTGGTCTGGACCGCCTTTGGTCAAAGCCGCCGACTTTCTCATGCAACTTCACGAATTC

CTCGATCGTTACAGCTTGAACCGCAATGTGCGCAACCCTATTGCCAAGCGCAAATCCGAC

AAGAAAGACAAGAGCATTGATGCCATGACCGAAGAGGAGATGATGGAGATGGCAATGAGA

AATAGTCTTGGGGCCGCAGCAGAAGCTGGTCCAACGCTCGAAGACCCTGATGAATTGACG

CGCAGTACAGACAATGTTAAGGGCAAGGGCCGAGCTACCGACGAGGAAGATGTTATCATG

GATGAACCGGAGGCAGCTGCCGAAGCCTCCCCATTTTCATCTATCCCCGGCGACCAACCA

CACACTGAACCCGCTGCGGACCCGGCCACAACTACACGCATTCAGTTCCGACACCCGTCT

GGCAGAGTTATTCGGCGTTTCTTATTGGCAGATCCCGTTCAGAGAATCTATGAATGGCTC

AAGGCCGATCCTCCTTTGGAAGACAAGGCTGGTGTCGAGTTTGACCTGAACAGCATGGGG

CGCAATCTCATTGATCAACTTACTACGAGCATTGCAGATGCGGGACTGAAGAATGGTACG

GTGATGATTGGATATCTGGAAGAG

>CL116.Contig2_All 199 1722 minus strand hypothetical protein PDIP_82300 [Penicillium digitatum Pd1] >gi|425780208|gb|EKV18225.1| hypothetical protein PDIG_10790 [Penicillium digitatum PHI26]

ATGGATAACGACGTGGTAGCTCAGTTCACTGAAATTACCGGCTCCACGCCGGAGCTGGCT

ATTCAGTACCTGCAGATCACCGAGTTCCAAATCGAGCAGGCCATGCAGCTTTACTTTGAA

AGTGGAGGCGCACCCTTGACTGCTGAACCTTCTCGGCCCTCTCATCTGTCTGGAATCCCA

CACGATTCCGAGGTGGTGAATATTGACTCGGATACTGATGACGATACCCCCCAACACATC

GCCCCGCCAACATTCGATGACGACGAGGCTATGGCTCGACGCCTCCAGGAGCAAATGTAT

GGTGGTACAGGTGGCATAGAAAATGAAGTTGAAGTGAGAGCGCCGTTAGCGCGGACAACG

GAGACTTTGGTGGGCCCCGGGGCGGACTATGACAGTGGTGAAGACATGCACGCCAACATC

CTCAGCCAACTACGAGCTCGTGGTAGATCAGGCCGAGCCGGAATCTTCAACCAGCAAGAG

TCATCTTCCATATGGACGGGCAGTGATGAATCTCGTCGCGAAGCGCTTTCTGCCGCTACT

GGGGGCGCATCGGAAGCATCTTCCAAATCAAATCTGCTCGCCGAAATGTACCGTCCTCCA

TTTGAAATCATGTCACGACTGCCTTGGGATGTGGCGCGCGAGGAGGGAAAGGACACCGAG

AAATGGCTGCTTGTCAATGTCCAGGACGCATCAATCTTTGACTGTCAGGTTCTGAACCGT

GATTTGTGGAAGGACCGTGGGGTGCAAGATACCGTCAAGGAGCATTTCATTTTCCTGCAG

TACTCGAAAGATGACCCCCGTGCCTCTTCCTACCTCCAATACTACTTCCAGGGAAGCGAT

GTCTCCGACAACTACCCCCACATTGCTATCGTCGACCCTCGGACTGGTGAACAAGTGAAG

GTCTGGTCTGGACCGCCTTTGGTCAAAGCCGCCGACTTTCTCATGCAACTTCACGAATTC

CTCGATCGTTACAGCTTGAACCGCAATGTGCGCAACCCTATTGCCAAGCGCAAATCCGAC

AAGAAAGACAAGAGCATTGATGCCATGACCGAAGAGGAGATGATGGAGATGGCAATGAGA

AATAGTCTTGGGGCCGCAGCAGAAGCTGGTCCAACGCTCGAAGACCCTGATGAATTGACG

CGCAGTACAGACAATGTTAAGGGCAAGGGCCGAGCTACCGACGAGGAAGATGTTATCATG

GATGAACCGGAGGCAGCTGCCGAAGCCTCCCCATTTTCATCTATCCCCGGCGACCAACCA

CACACTGAACCCGCTGCGGACCCGGCCACAACTACACGCATTCAGTTCCGACACCCGTCT

GGCAGAGTTATTCGGCGTTTCTTATTGGCAGATCCCGTTCAGAGAATCTATGAATGGCTC

AAGGCCGATCCTCCTTTGGAAGACAAGGCTGGTGTCGAGTTTGACCTGAACAGCATGGGG

CGCAATCTCATTGATCAACTTACTACGAGCATTGCAGATGCGGGACTGAAGAATGGTACG

GTGATGATTGGATATCTGGAAGAG

>CL117.Contig1_All 254 1882 minus strand Nucleolar protein 12 [Penicillium digitatum Pd1] >gi|425777183|gb|EKV15367.1| Nucleolar protein 12 [Penicillium digitatum PHI26]

ATGGGCAAAAATTCAAATTCTTCATCCAAGACGGAGGAGAAGACCAGCAAGGCCGCTAGC

ACTCTTCCCTTTGGCGGTGTATCTCTGGACCCAACATTGTCCTCGCTTTTTGCGCAAAGT

GTATGTATGTCTTGATGTGCAATTAATGAACTTTGAACTGACAAAATCTAGGCGGGTCCC

GTGCAAGCCCCCGTGATCAAATATGTCGAGCCCATCCAACGGGCGAAAAAGGACGAAGAT

GATGCTTCTGAAGAGGAGGAATTGTCTGCATCAGGCGATGAAGTAATGGAGGATGCTGCT

GAAGAAAGCGATTCTGAGGCCGCAGAGGACGAAAAACCTACCGAGACACAGAACCGGAAA

CGCAAACGTGGCAATGCCGGCGAGGATGTCGAGGAGACATACATGCGTCGCATTGCTAAG

GAGGAGGAGAAGGATGAGGAAAAGAGGAAGACCGAGCAAGCTAAGCGCCAAAAGCAGACC

AAACAAGAAGGCAGCAAAGATGACAAAGATGAGGAAGGCGAGGACTCCGACAAAGTATCG

GCATCAGAGGACAGCGATGAAGAGGAGATTGCTCCTGCACCTGTTCACGAAAGTTTGACT

GGTTCCGCCAAGGCCGATGAAGTTGAGAAATCCAACCGCACCGTCTTTCTAGGCAACGTC

TCTACCGAGGCTATCAGGTCCAAGACCGCCAAGAAGACACTTCTCAGGCACCTGACCTCA

TTCTGCTCTTCGCTTCCGGAATCCACCGGCCCGCACAAGATCGAGTCTATCCGTTTCCGC

TCCGTTGCATTCGCCAGTGGGGGTGGTATTCCCAAGCGTGCCTCCTTTGCGAAACGTGAG

CTTCTCGACGAGACCACACCCAGCACCAACGCATACGCTGTCTACACCACTCTGCACGCT

GCACGCAAGGCGCCTGCCGCCTTGAACGGTACCATTGTTTTGGACCGTCACTTGCGAGTT

GATAGCCTTGCTCACCCCTCAGAAATTGATCACAAGCGCTGTGTGTTTGTCGGCAACTTG

TCTTTCATTGACAGTGAGACCCCCGAAGAGGATGAGAAGACGGGTAAGAAGAAGAAGGCT

CGCGCCCCCGCCGATGTCGAGGAGGGTCTCTGGCGTATTTTCAACGCTCACACTGGTGGC

AAGGACAAGAAGGCCATCAAGAAGAACGTCGAGTTCGTGCGCGTGATCCGTGACAGCACC

ACTCGTGTTGGTAAAGGATTTGCCTATGTCCAGTTCTACGATGGCAACGGTGTCGAGGAG

TCGCTTCCGCTCAATGGAAAGAACTTCCCGCCCATGCTTCCCCGTAAGCTGCGTGTCACT

CGGGCCCGCAAGATCGCTAAGAAGCGCGAACCCTCTGGCCCTGAAGCCAAGAAGTCTCGT

GTAGACGAGGCCCAGAAGACAATGCAAGGTCGTGCCAACAGGCTACTGGGCCGAGCGGGT

GCGGCTAAAGTGAAGGCCGATGCCAACAGTACCATTGCCGGCAACTCTTTCGTGTTTGAG

GGTCACCGCGCTACTGAGGGATCCTCGTCCATCAAGATGAAGCAAAAGAGCCGTGGCTCG

AAGGCCAAACGAGAAAGCAGAAGCAGTAAGCGGGCTGCCGCATACAAAGCTGCGGGTGGC

AGACGGGGC

>CL117.Contig2_All 254 1831 minus strand Nucleolar protein 12 [Penicillium digitatum Pd1] >gi|425777183|gb|EKV15367.1| Nucleolar protein 12 [Penicillium digitatum PHI26]

ATGGGCAAAAATTCAAATTCTTCATCCAAGACGGAGGAGAAGACCAGCAAGGCCGCTAGC

ACTCTTCCCTTTGGCGGTGTATCTCTGGACCCAACATTGTCCTCGCTTTTTGCGCAAAGT

GCGGGTCCCGTGCAAGCCCCCGTGATCAAATATGTCGAGCCCATCCAACGGGCGAAAAAG

GACGAAGATGATGCTTCTGAAGAGGAGGAATTGTCTGCATCAGGCGATGAAGTAATGGAG

GATGCTGCTGAAGAAAGCGATTCTGAGGCCGCAGAGGACGAAAAACCTACCGAGACACAG

AACCGGAAACGCAAACGTGGCAATGCCGGCGAGGATGTCGAGGAGACATACATGCGTCGC

ATTGCTAAGGAGGAGGAGAAGGATGAGGAAAAGAGGAAGACCGAGCAAGCTAAGCGCCAA

AAGCAGACCAAACAAGAAGGCAGCAAAGATGACAAAGATGAGGAAGGCGAGGACTCCGAC

AAAGTATCGGCATCAGAGGACAGCGATGAAGAGGAGATTGCTCCTGCACCTGTTCACGAA

AGTTTGACTGGTTCCGCCAAGGCCGATGAAGTTGAGAAATCCAACCGCACCGTCTTTCTA

GGCAACGTCTCTACCGAGGCTATCAGGTCCAAGACCGCCAAGAAGACACTTCTCAGGCAC

CTGACCTCATTCTGCTCTTCGCTTCCGGAATCCACCGGCCCGCACAAGATCGAGTCTATC

CGTTTCCGCTCCGTTGCATTCGCCAGTGGGGGTGGTATTCCCAAGCGTGCCTCCTTTGCG

AAACGTGAGCTTCTCGACGAGACCACACCCAGCACCAACGCATACGCTGTCTACACCACT

CTGCACGCTGCACGCAAGGCGCCTGCCGCCTTGAACGGTACCATTGTTTTGGACCGTCAC

TTGCGAGTTGATAGCCTTGCTCACCCCTCAGAAATTGATCACAAGCGCTGTGTGTTTGTC

GGCAACTTGTCTTTCATTGACAGTGAGACCCCCGAAGAGGATGAGAAGACGGGTAAGAAG

AAGAAGGCTCGCGCCCCCGCCGATGTCGAGGAGGGTCTCTGGCGTATTTTCAACGCTCAC

ACTGGTGGCAAGGACAAGAAGGCCATCAAGAAGAACGTCGAGTTCGTGCGCGTGATCCGT

GACAGCACCACTCGTGTTGGTAAAGGATTTGCCTATGTCCAGTTCTACGATGGCAACGGT

GTCGAGGAGTCGCTTCCGCTCAATGGAAAGAACTTCCCGCCCATGCTTCCCCGTAAGCTG

CGTGTCACTCGGGCCCGCAAGATCGCTAAGAAGCGCGAACCCTCTGGCCCTGAAGCCAAG

AAGTCTCGTGTAGACGAGGCCCAGAAGACAATGCAAGGTCGTGCCAACAGGCTACTGGGC

CGAGCGGGTGCGGCTAAAGTGAAGGCCGATGCCAACAGTACCATTGCCGGCAACTCTTTC

GTGTTTGAGGGTCACCGCGCTACTGAGGGATCCTCGTCCATCAAGATGAAGCAAAAGAGC

CGTGGCTCGAAGGCCAAACGAGAAAGCAGAAGCAGTAAGCGGGCTGCCGCATACAAAGCT

GCGGGTGGCAGACGGGGC

>CL118.Contig1_All 431 937 hypothetical protein PDIP_67600 [Penicillium digitatum Pd1] >gi|425771683|gb|EKV10120.1| hypothetical protein PDIG_58110 [Penicillium digitatum PHI26]

ATGGGAAACTTTGGAGCATGTAGCTGCGAACGCTACGTTGTTAGGTCCAAGCGAACAATA

TCAGTTGCTCTTCTCGCCATCTCAACGGTTCTCATCGCCTTATGCGCCGAATACTTTTCT

TCAAGCTTCTCAACGCTCAACCAACAAGGCGTCCTCGGTGAATCCTTCGTCGGTTTAATT

GTCATTCCCATCGCCGGAAACGTTGCTGAGAATGTCACCGCGGTTGTCGTGGCCTCGAAA

AACCAGATGGATCTTGCCATCAGTGTTGCTCTTGGATCAGCCATTCAAATCGGTCTTTTG

GTTGCACCTGCGATGGTGCTTATAGGTTGGGCTCTGGACAAGTCTATGACCTTACATTTT

GATGGGTTCGAACTGGTCACACTGATCGGGGCTGTTCTTCTGGTTGACTTTATCGTTCTC

AAAGGAAAGACCAATTATCTGGAGGGCGCTATCTTATGCGCCTGCTTTGCAGCTATTTCG

GTTGGCGCATACTTGTTGCCGCTCACT

>CL118.Contig2_All 562 1068 hypothetical protein PDIP_67600 [Penicillium digitatum Pd1] >gi|425771683|gb|EKV10120.1| hypothetical protein PDIG_58110 [Penicillium digitatum PHI26]

ATGGGAAACTTTGGAGCATGTAGCTGCGAACGCTACGTTGTTAGGTCCAAGCGAACAATA

TCAGTTGCTCTTCTCGCCATCTCAACGGTTCTCATCGCCTTATGCGCCGAATACTTTTCT

TCAAGCTTCTCAACGCTCAACCAACAAGGCGTCCTCGGTGAATCCTTCGTCGGTTTAATT

GTCATTCCCATCGCCGGAAACGTTGCTGAGAATGTCACCGCGGTTGTCGTGGCCTCGAAA

AACCAGATGGATCTTGCCATCAGTGTTGCTCTTGGATCAGCCATTCAAATCGGTCTTTTG

GTTGCACCTGCGATGGTGCTTATAGGTTGGGCTCTGGACAAGTCTATGACCTTACATTTT

GATGGGTTCGAACTGGTCACACTGATCGGGGCTGTTCTTCTGGTTGACTTTATCGTTCTC

AAAGGAAAGACCAATTATCTGGAGGGCGCTATCTTATGCGCCTGCTTTGCAGCTATTTCG

GTTGGCGCATACTTGTTGCCGCTCACT

>CL119.Contig1_All 686 1039 minus strand NAD-dependent D-isomer specific 2-hydroxyacid dehydrogenase, putative [Penicillium digitatum Pd1] >gi|425771851|gb|EKV10283.1| NAD-dependent D-isomer specific 2-hydroxyacid dehydrogenase, putative [Penicillium digitatum PHI26]

AGCAGTTCGGACGTGCTCAGCTTGAACTTGGCCTTGAACGCCTCTACGCGCCACATTATT

GGTGCGACCGAGTTCGCCAAGATGAAGGATGGTGTCGTGATTGTGAACACGGCTCGTGGT

GCGCTGATTGATGAGAAGGCGTTGGTTGCGGCGCTTGACTCTGGCAAGGTGCGTTCTGCT

GGTTTGGATGTGTATGAGTGTGAGCCGCAAATTGAGCCTGGGCTAATCAGCAACCCTAAT

GTGATGTTGCTGCCGCATATTGGTACTGGAACCTATGAGACTCAAAAGGAGATGGAGATT

CTGGTGCTGGATAACCTGCGGTCGGCTGTTGAAAAGGGTGAGCTTATTACACAG

>CL119.Contig2_All 93 1088 minus strand NAD-dependent D-isomer specific 2-hydroxyacid dehydrogenase, putative [Penicillium digitatum Pd1] >gi|425771851|gb|EKV10283.1| NAD-dependent D-isomer specific 2-hydroxyacid dehydrogenase, putative [Penicillium digitatum PHI26]

ATGGCACCACAATCAGCTCTTCTCATTGGAGAAATTACGCATGCGCGCAAGGAGTGGGAG

AGCATCTCATCTCTTTTGACATTGAAGGAATTTCCCAGCGGAACCAGAGAGGAGTTCATT

GCAAACTGCAAAGCTGGCAAGTACGACGATGTAGTCGCTCTCTACCGATCCAACAACTCC

ACCAAGTACACCGGTCCGTTCAATGCAGAGATGCTCGCAGTCCTGCCTGAATCTCTGAAG

TACATCTGCCATAACGGCGCGGGATACGATAACATTGATGTCGCTGCCTGCACAGAGAAG

AACATCGCTGTGTCCAGCACCCCAGTCGCTGTCAACAATGCGACAGCCGACGTGGGTATT

TTCCTCATGATCGGCGCTTTGCGCCAGGCCCATGTACCTATTACTGCTCTCCGGGAAGGA

AAGTGGCAAGGCCCAATCACCGCAGGAGGGAAGGAGTACAAGACCACCCTGGGCCACGAT

CCCAAGAACAAGGTCCTCGGAATTCTGGGTATGGGAGGTATCGGACGGGAAATGGCAATC

CGCGCCAAGGCCTTCGGAATGAAGATTCAGTACCACAACCGATCTCGTCTACCAGCTGAT

CTGGAAGTTAGCGCGACATACGTCTCGTTTGATGAGTTACTTGCCAGTTCGGACGTGCTC

AGCTTGAACTTGGCCTTGAACGCCTCTACGCGCCACATTATTGGTGCGACCGAGTTCGCC

AAGATGAAGGATGGTGTCGTGATTGTGAACACGGCTCGTGGTGCGCTGATTGATGAGAAG

GCGTTGGTTGCGGCGCTTGACTCTGGCAAGGTGCGTTCTGCTGGTTTGGATGTGTATGAG

TGTGAGCCGCAAATTGAGCCTGGGCTAATCAGCAACCCTAATGTGATGTTGCTGCCGCAT

ATTGGTACTGGAACCTATGAGACTCAAAAGGAGATGGAGATTCTGGTGCTGGATAACCTG

CGGTCGGCTGTTGAAAAGGGTGAGCTTATTACACAG

>CL119.Contig3_All 315 500 minus strand NAD-dependent D-isomer specific 2-hydroxyacid dehydrogenase, putative [Penicillium digitatum Pd1] >gi|425771851|gb|EKV10283.1| NAD-dependent D-isomer specific 2-hydroxyacid dehydrogenase, putative [Penicillium digitatum PHI26]

GTGCGTTCTGCTGGTTTGGATGTGTATGAGTGTGAGCCGCAAATTGAGCCTGGGCTAATC

AGCAACCCTAATGTGATGTTGCTGCCGCATATTGGTACTGGAACCTATGAGACTCAAAAG

GAGATGGAGATTCTGGTGCTGGATAACCTGCGGTCGGCTGTTGAAAAGGGTGAGCTTATT

ACACAG

>CL119.Contig4_All 93 527 minus strand NAD-dependent D-isomer specific 2-hydroxyacid dehydrogenase, putative [Penicillium digitatum Pd1] >gi|425771851|gb|EKV10283.1| NAD-dependent D-isomer specific 2-hydroxyacid dehydrogenase, putative [Penicillium digitatum PHI26]

ATGGCACCACAATCAGCTCTTCTCATTGGAGAAATTACGCATGCGCGCAAGGAGTGGGAG

AGCATCTCATCTCTTTTGACATTGAAGGAATTTCCCAGCGGAACCAGAGAGGAGTTCATT

GCAAACTGCAAAGCTGGCAAGTACGACGATGTAGTCGCTCTCTACCGATCCAACAACTCC

ACCAAGTACACCGGTCCGTTCAATGCAGAGATGCTCGCAGTCCTGCCTGAATCTCTGAAG

TACATCTGCCATAACGGCGCGGGATACGATAACATTGATGTCGCTGCCTGCACAGAGAAG

AACATCGCTGTGTCCAGCACCCCAGTCGCTGTCAACAATGCGACAGCCGACGTGGGTATT

TTCCTCATGATCGGCGCTTTGCGCCAGGCCCATGTACCTATTACTGCTCTCCGGGAAGGT

AAGTGGCTCTAACCT

>CL120.Contig1_All 61 231 Rho GTPase activator (Rgd1), putative [Penicillium digitatum PHI26] >gi|425784196|gb|EKV21987.1| Rho GTPase activator (Rgd1), putative [Penicillium digitatum Pd1]

ATGTCGGATCACACCCTTCGCGAATCCACGGCGATACCGACGCTCTCCGATGCGCCTTCG

TCGAGCGCCGGTCGCTCATCCCCTAGCCACGGGGCCGAGCAGACTCAGCCGACCCACGAG

GCCATCTCAGATGAGCTGAAAGCCCGCCTGGATAAAGTTCTCTACTCAGAT

>CL120.Contig2_All 133 2037 minus strand Rho GTPase activator (Rgd1), putative [Penicillium digitatum PHI26] >gi|425784196|gb|EKV21987.1| Rho GTPase activator (Rgd1), putative [Penicillium digitatum Pd1]

ATGTCGGATCACACCCTTCGCGAATCCACGGCGATACCGACGCTCTCCGATGCGCCTTCG

TCGAGCGCCGGTCGCTCATCCCCTAGCCACGGGGCCGAGCAGACTCAGCCGACCCACGAG

GCCATCTCAGATGAGCTGAAAGCCCGCCTGGATAAAGTTCTCTACTCAGATATTGGCATA

ACCACACTCCTGACACGGCTGAAGCAAAGCGTGGCCTCCGCTAAGGATTTTTCAACCTTC

CTGAAAAAACGTGGGTCCCTTGAAGAAGAGCACGCTCAGGGGCTAAGAAAACTGTCCCGC

GTAATCAGCGATGCGTCACAACGCTCCGAGAACCGACAAGGAACATACAGTTCTAGCTAC

AAGGACATCCACCGAATCCAGGAGCGAATGGTCGACCATGGCCTCCAATTCGCCGTCTCC

CTCCACCAGATGTCCGATGATCTGCATGAGCTCGCCGCCAACATTGAGCGCGGCCGCAAG

CAATGGAAACAGACCGGCTTAGCTGCGGAGAAGAAGGTCGTCGATGCAGAATCGCTGGCG

GAGAAGGCAAAGGCTAAGTACGAGTCACTGGCGGAGCAGTATGACCGTGTTCGCACGGGA

GACAAGCAGGGCGGAAAGTTCGGTCTTAAGGGCCACAAGTCTGCGGCGCAGCACGAGGAG

GAGTTGTTGCGCAAGGTCCAGAACGCGGATACCGACTATGGGTCTAAAGTTCAGGCTGCT

CAGGCGGCGCGCCAGGAACTGGTGTCTACCCACCGACCTCAGGCTGTACACAATCTTCAG

CAGTTAATAGCCGAGTGCGACTCGGGATTGACCCTGCAACAGCAGAAGTTTGCTACTTTC

AACGAGAAACTGCTACTAGGCCAAGGTCTCTGCGTCAACCCTCTGAAGACCGATGGGGCA

TCAGGACCTAAGAGCCTTGCCGATGTTATTCGCCAAGTTGATAATCAGAGAGATCTCCAC

GAGTTTATTTTAAGCCACGAAGGCAATCCTGGGGCCGTGGCGTCAGAACAGGTCAAATAT

GAGCGCCACCCTACACTAGGTGGTGCCGGGGCTGTTTCTTCTACTTCTGCTTCTGCGGCA

CCTTCGCATCCAAGCACCCAAAATAAGCCGCTTCAACCTCCGCAAAGCGGTGACCGATTA

CCGCAACCACCTTATCCCGCTGAGAACACCTTCACTCCGCCCCCAGCCAGCACACCTCTC

TACCTTGTCTCAGGACCGCCTGCTCCGGAACCCGCACCTAAGATCCCGCTCCCAATAGCA

GGACCTCCCCCAGTGGACAGGAATTTGCAATCAAATCTCCCTCCGCTGAGGCCAGTGTTC

GGTGTTTCGCTAAACGACCTGTACGCCCGGGATGGAACAGCAGTTCCTTTCATTGTGTAC

CAGTGCTTCCAAGCGGTTGAATTGTTCGGTCTGGATGTGGAGGGCATCTATCGACTCTCC

GGTAGTGCCAACCACATCAGTCACATGAAAGCATTGTTCGACAATGACTCATCGCAAGTC

GACTTCACAAACCCGGAGAGTTTTTACCATGATGTCAACAGCGTTGCAGGACTTGTGAAG

CAATTCTTCCGAGACCTACCTGATCCTCTGTTCACCACTCAGTTCTACCAACAGTTTGTT

GATGCTGCTCGCTTCGACGATGACATCCAGCGCCGAGACTCGATGCATGCCCTGATTAAT

AGTTTGCCTGATGCCCACTATGCCACTCTGCGGGCCATCATTTTGCATCTCAATAAGATC

CAAGAACACTATACCCAGAACCGCATGAACGCGGGAAATCTGGCCATTTGTTTCGGACCA

ACCCTCTTGGGCGCCAATTCCGGTGGCAGCATCGCCGACGCTGGGTGGCAAGTTCGTGTC

ATCGAGACGATCCTGAATAACACATTCCAGATCTTCGACGATGAC

>CL121.Contig1_All 81 182 minus strand GTP-binding protein [Penicillium digitatum PHI26] >gi|425780395|gb|EKV18402.1| GTP-binding protein [Penicillium digitatum Pd1]

ATGCCTCGAGATCCACTGATCGGACTGGTGGGCAAGCCGTCTAGTGGAAAATCCACGACA

TTGAACAGTTTGACCGATGCGACTTCGAAAGTTGGTAAGTAT

>CL121.Contig2_All 81 1322 minus strand GTP-binding protein [Penicillium digitatum PHI26] >gi|425780395|gb|EKV18402.1| GTP-binding protein [Penicillium digitatum Pd1]

ATGCCTCGAGATCCACTGATCGGACTGGTGGGCAAGCCGTCTAGTGGAAAATCCACGACA

TTGAACAGTTTGACCGATGCGACTTCGAAAGTTGGAAATTTTCCCTTCACAACAATTGAC

CCCCAGCGAGCCATTGGATACCTTCAGATCGACTGCCCATGTCAGCGACACAATGTTTCG

GACCGGTGCCAGCCCAATTACGGAAGCTGCCATGAAGGGCGACGTTCCGTGCCGATTGAG

TTGCTAGATGTCGCAGGTCTTGTGCCAGGTGCGCATCAAGGTCGTGGACTGGGAAACAAG

TTCTTGGACGATCTGCGCAACGCCGATGCTCTAATTCACGTCGTGGATGTGAGTGGGACC

ACGGATGCAGAAGGAAAGGCTACTCGAGGATATGATCCATCGGTAGACATTGAGTGGTTG

CGATCAGAGATCGTGCGATGGGTGTTGGGAAACCTAATGGAGAGGTGGGGCTCTATCAAG

AGGAGACACCAGGCATTGAAGGCAAACCCCATCGACACATTGCAGAAGCAGTTTGCTGGT

TATGGAAGTACTCATGTCACCGTTGCACGATTCATGGATAAACTTGCCTTAAAGGAGCCG

TTGGAAGATTGGTCAAATGAAACGGTAGAGCTTGTTGTGAATGCATTTATCGACGAGAAA

TTCCCCACAGTGTTCGCACTGAACAAGATCGACCACCCAGATGCAGACAAGAACATCAGC

AAGATTGCGAAAATGCAAGATCCCCAAAGCATCGTGCTTTGCTCTGCTATATCGGAAGTT

TTCCTTCGAAGGCTAGCAAAGCAGAAGTACATCAAGTATGTCGAGGGCAGCGAGTTTGTG

GACACACGAGAAGATTTGATCGAGATGGGTGACCCAGATGGGGGAGGCCTCAAAGAGATG

GACGAAAAATTGAAAACTCGGGTTGAGAATATGAAAGACATGGTGCTTTATCGATTTGGA

TCCACCGGAGTCGTCCAGTGTCTTTCCCGGGCTGCAGAACTTCTGGGTCTCGTCCCAGTG

TTTCCAGTCCGGAACGTGGCAACATTCAACTCTGGCACTGGAACTGCCGTATTTCGCGAT

TGTGTTTTGGTCAACAAAAACAGTACTGTCGGTGATGTTGCCCGGAAAGTAATGGGTGAT

GTACCTATCAACTATGTCGAAGGAGTGGGTGGAACTCGAGTCTCCGAGGATGACATTGTG

GCAGTTGGGAAAAACGATATCCTATCATTTAAGGTTGGTCGG

>CL122.Contig1_All 3 497 minus strand hypothetical protein PDIP_85010 [Penicillium digitatum Pd1] >gi|425775174|gb|EKV13456.1| hypothetical protein PDIG_38380 [Penicillium digitatum PHI26]

AAACTTTCCACGATCTCGCTGATCATTTGGCTTGCTGGTTTCTTTGCATTGTTCCAAGCT

CTTTTGAACGCTTTGGCCGAGGTCATGCGGTTTGGAGATCGCGAGTTCTACACTGACTGG

TGGAACAGCTCCAGTCTGGGTATGTATTGGCGATCTTGGAACCGACCTGTTTACCTGTTC

ATGAAGAGACATGTCTACTCGCCGCTGGTGGGCCGTGGATGGAGTCCTCTGGCTGCGAGT

GGAATGGTTTTCACGCTCTCCGCGATTCTTCATGAAATGCTTGTTGGCATTCCCACACAT

AACTTCATTGGTGTCGCGTTCTTTGGAATGATGTTCCAGCTGCCGTTAATTGGCCTCACC

GCACCATTGGATAAAATGCGTGGGCCTGAAGGCAGGGTCATCGGCAACTGCATCTTCTGG

GTCAGCTTTTGTCTAGTCGGACAACCGCTTGGAGCGCTCCTGTACTTCTTCGCTTGGCAG

GCCAAGTACGGGAGT

>CL122.Contig2_All 881 1486 minus strand Sterol O-acyltransferase 2 OS=Saccharomyces bayanus GN=ARE2 PE=3 SV=1

TACCCAAGAAACATTACCATCAATAACCTGGCCTACTTCTGGCTGGCGCCGACGCTGGTG

TATCAGCCTGTTTATCCGCGCACGGCATCCATCCGGTGGTCTTTTGTTGCAAAGCGCCTT

GCAGAGTTCGTGGGCCTGGCAATGTTCATTTGGCTTCTGTCTGCGCAGTATGCTGCGCCA

GTTCTGCGCAACTCGATCGATAAGATCGCAGTAATGGACCTTACATCCATTTTGGAGCGA

GTCATGAAACTTTCCACGATCTCGCTGATCATTTGGCTTGCTGGTTTCTTTGCATTGTTC

CAAGCTCTTTTGAACGCTTTGGCCGAGGTCATGCGGTTTGGAGATCGCGAGTTCTACACT

GACTGGTGGAACAGCTCCAGTCTGGGTATGTATTGGCGATCTTGGAACCGACCTGTTTAC

CTGTTCATGAAGAGACATGTCTACTCGCCGCTGGTGGGCCGTGGATGGAGTCCTCTGGCT

GCGAGTGGAATGGTTTTCACGCTCTCCGCGATTCTTCATGAAATGCTTGTTGGCATTCCC

ACACATAACTTCATTGGTGTCGCGTTCTTTGGAATGATGTTCCAGCTGCCGTTAATTGGC

CTCACC

>CL122.Contig3_All 158 1669 minus strand hypothetical protein PDIP_85010 [Penicillium digitatum Pd1] >gi|425775174|gb|EKV13456.1| hypothetical protein PDIG_38380 [Penicillium digitatum PHI26]

ATGACTGCTCGCATTGGTGCAGTTACGAGCGCTCGCGAAGAATCTCAGGTCAATTCAACG

AACGGGGCCACTCCGGATGTGACCAAGGTCTCTCCGCCATCAACTTCAACTTCCAAGAGA

CGCAGTAAATATCGCCATGTAGCTGCATACCACTCAGAGGCTCGTCATTCTAGCCTCAGT

CGAGAGGCTGAAGTGCTTCCCAACTTCCTGGGGCTTCGAAATCTCATGGTGTTGGTACTT

GTGGCGATGAACCTGCGATTGATTATTGAGAACTTCATGAAATATGGCGTGCTAATCTGT

ATTAAATGCCATGACTACCGCAGACAGGACGTTGTTTTGGGGTCTATTTTGTTTGCCTCG

GTTCCGTGCCATTTGCTCGTGGCATACATCATTGAACTTTCAGCCGCTACTGCAGCTAAG

CAGACAGTTGGTCGTCAGAAGAAGACAGAAAAGGAGAAAGAGCACGACCAAAAGGTCTTC

CAGTCGACGTGGCCGTACACTGCTTTCCTACACACGCTCAATGCCACGCTATGTCTCACA

GTAACCAGCTTCGTGGTGTATTTCTACATCCATCACCCTGGCATTGGGACATTATGCCAG

CTACACGCACTCATTGTATGGTTAAAGAACTGCTCTTATGCCTTCACAAACCGAGACCTT

CGCCTCGCTATGCTCAATCCTTCTGCAGACCCGGGCTTGCCCGAAATCTACTCCTCGTGT

CCTTACCCAAGAAACATTACCATCAATAACCTGGCCTACTTCTGGCTGGCGCCGACGCTG

GTGTATCAGCCTGTTTATCCGCGCACGGCATCCATCCGGTGGTCTTTTGTTGCAAAGCGC

CTTGCAGAGTTCGTGGGCCTGGCAATGTTCATTTGGCTTCTGTCTGCGCAGTATGCTGCG

CCAGTTCTGCGCAACTCGATCGATAAGATCGCAGTAATGGACCTTACATCCATTTTGGAG

CGAGTCATGAAACTTTCCACGATCTCGCTGATCATTTGGCTTGCTGGTTTCTTTGCATTG

TTCCAAGCTCTTTTGAACGCTTTGGCCGAGGTCATGCGGTTTGGAGATCGCGAGTTCTAC

ACTGACTGGTGGAACAGCTCCAGTCTGGGTATGTATTGGCGATCTTGGAACCGACCTGTT

TACCTGTTCATGAAGAGACATGTCTACTCGCCGCTGGTGGGCCGTGGATGGAGTCCTCTG

GCTGCGAGTGGAATGGTTTTCACGCTCTCCGCGATTCTTCATGAAATGCTTGTTGGCATT

CCCACACATAACTTCATTGGTATGTCTTTCACTCTCTGATCACATCTTTCATGCTAATTA

GTTCTAGGTGTCGCGTTCTTTGGAATGATGTTCCAGCTGCCGTTAATTGGCCTCACCGCA

CCATTGGATAAAATGCGTGGGCCTGAAGGCAGGGTCATCGGCAACTGCATCTTCTGGGTC

AGCTTTTGTCTAGTCGGACAACCGCTTGGAGCGCTCCTGTACTTCTTCGCTTGGCAGGCC

AAGTACGGGAGT

>CL122.Contig4_All 938 1543 minus strand Sterol O-acyltransferase 2 OS=Saccharomyces bayanus GN=ARE2 PE=3 SV=1

TACCCAAGAAACATTACCATCAATAACCTGGCCTACTTCTGGCTGGCGCCGACGCTGGTG

TATCAGCCTGTTTATCCGCGCACGGCATCCATCCGGTGGTCTTTTGTTGCAAAGCGCCTT

GCAGAGTTCGTGGGCCTGGCAATGTTCATTTGGCTTCTGTCTGCGCAGTATGCTGCGCCA

GTTCTGCGCAACTCGATCGATAAGATCGCAGTAATGGACCTTACATCCATTTTGGAGCGA

GTCATGAAACTTTCCACGATCTCGCTGATCATTTGGCTTGCTGGTTTCTTTGCATTGTTC

CAAGCTCTTTTGAACGCTTTGGCCGAGGTCATGCGGTTTGGAGATCGCGAGTTCTACACT

GACTGGTGGAACAGCTCCAGTCTGGGTATGTATTGGCGATCTTGGAACCGACCTGTTTAC

CTGTTCATGAAGAGACATGTCTACTCGCCGCTGGTGGGCCGTGGATGGAGTCCTCTGGCT

GCGAGTGGAATGGTTTTCACGCTCTCCGCGATTCTTCATGAAATGCTTGTTGGCATTCCC

ACACATAACTTCATTGGTGTCGCGTTCTTTGGAATGATGTTCCAGCTGCCGTTAATTGGC

CTCACC

>CL122.Contig5_All 158 1726 minus strand hypothetical protein PDIP_85010 [Penicillium digitatum Pd1] >gi|425775174|gb|EKV13456.1| hypothetical protein PDIG_38380 [Penicillium digitatum PHI26]

ATGACTGCTCGCATTGGTGCAGTTACGAGCGCTCGCGAAGAATCTCAGGTCAATTCAACG

AACGGGGCCACTCCGGATGTGACCAAGGTCTCTCCGCCATCAACTTCAACTTCCAAGAGA

CGCAGTAAATATCGCCATGTAGCTGCATACCACTCAGAGGCTCGTCATTCTAGCCTCAGT

CGAGAGGCTGAAGTGCTTCCCAACTTCCTGGGGCTTCGAAATCTCATGGTGTTGGTACTT

GTGGCGATGAACCTGCGATTGATTATTGAGAACTTCATGAAAGTGAGTAATTTAAAGGTT

CAACTACTGTTTCGAGGCTGCCAACTAACAATCATCCAGTATGGCGTGCTAATCTGTATT

AAATGCCATGACTACCGCAGACAGGACGTTGTTTTGGGGTCTATTTTGTTTGCCTCGGTT

CCGTGCCATTTGCTCGTGGCATACATCATTGAACTTTCAGCCGCTACTGCAGCTAAGCAG

ACAGTTGGTCGTCAGAAGAAGACAGAAAAGGAGAAAGAGCACGACCAAAAGGTCTTCCAG

TCGACGTGGCCGTACACTGCTTTCCTACACACGCTCAATGCCACGCTATGTCTCACAGTA

ACCAGCTTCGTGGTGTATTTCTACATCCATCACCCTGGCATTGGGACATTATGCCAGCTA

CACGCACTCATTGTATGGTTAAAGAACTGCTCTTATGCCTTCACAAACCGAGACCTTCGC

CTCGCTATGCTCAATCCTTCTGCAGACCCGGGCTTGCCCGAAATCTACTCCTCGTGTCCT

TACCCAAGAAACATTACCATCAATAACCTGGCCTACTTCTGGCTGGCGCCGACGCTGGTG

TATCAGCCTGTTTATCCGCGCACGGCATCCATCCGGTGGTCTTTTGTTGCAAAGCGCCTT

GCAGAGTTCGTGGGCCTGGCAATGTTCATTTGGCTTCTGTCTGCGCAGTATGCTGCGCCA

GTTCTGCGCAACTCGATCGATAAGATCGCAGTAATGGACCTTACATCCATTTTGGAGCGA

GTCATGAAACTTTCCACGATCTCGCTGATCATTTGGCTTGCTGGTTTCTTTGCATTGTTC

CAAGCTCTTTTGAACGCTTTGGCCGAGGTCATGCGGTTTGGAGATCGCGAGTTCTACACT

GACTGGTGGAACAGCTCCAGTCTGGGTATGTATTGGCGATCTTGGAACCGACCTGTTTAC

CTGTTCATGAAGAGACATGTCTACTCGCCGCTGGTGGGCCGTGGATGGAGTCCTCTGGCT

GCGAGTGGAATGGTTTTCACGCTCTCCGCGATTCTTCATGAAATGCTTGTTGGCATTCCC

ACACATAACTTCATTGGTATGTCTTTCACTCTCTGATCACATCTTTCATGCTAATTAGTT

CTAGGTGTCGCGTTCTTTGGAATGATGTTCCAGCTGCCGTTAATTGGCCTCACCGCACCA

TTGGATAAAATGCGTGGGCCTGAAGGCAGGGTCATCGGCAACTGCATCTTCTGGGTCAGC

TTTTGTCTAGTCGGACAACCGCTTGGAGCGCTCCTGTACTTCTTCGCTTGGCAGGCCAAG

TACGGGAGT

>CL122.Contig6_All 158 1678 minus strand hypothetical protein PDIP_85010 [Penicillium digitatum Pd1] >gi|425775174|gb|EKV13456.1| hypothetical protein PDIG_38380 [Penicillium digitatum PHI26]

ATGACTGCTCGCATTGGTGCAGTTACGAGCGCTCGCGAAGAATCTCAGGTCAATTCAACG

AACGGGGCCACTCCGGATGTGACCAAGGTCTCTCCGCCATCAACTTCAACTTCCAAGAGA

CGCAGTAAATATCGCCATGTAGCTGCATACCACTCAGAGGCTCGTCATTCTAGCCTCAGT

CGAGAGGCTGAAGTGCTTCCCAACTTCCTGGGGCTTCGAAATCTCATGGTGTTGGTACTT

GTGGCGATGAACCTGCGATTGATTATTGAGAACTTCATGAAAGTGAGTAATTTAAAGGTT

CAACTACTGTTTCGAGGCTGCCAACTAACAATCATCCAGTATGGCGTGCTAATCTGTATT

AAATGCCATGACTACCGCAGACAGGACGTTGTTTTGGGGTCTATTTTGTTTGCCTCGGTT

CCGTGCCATTTGCTCGTGGCATACATCATTGAACTTTCAGCCGCTACTGCAGCTAAGCAG

ACAGTTGGTCGTCAGAAGAAGACAGAAAAGGAGAAAGAGCACGACCAAAAGGTCTTCCAG

TCGACGTGGCCGTACACTGCTTTCCTACACACGCTCAATGCCACGCTATGTCTCACAGTA

ACCAGCTTCGTGGTGTATTTCTACATCCATCACCCTGGCATTGGGACATTATGCCAGCTA

CACGCACTCATTGTATGGTTAAAGAACTGCTCTTATGCCTTCACAAACCGAGACCTTCGC

CTCGCTATGCTCAATCCTTCTGCAGACCCGGGCTTGCCCGAAATCTACTCCTCGTGTCCT

TACCCAAGAAACATTACCATCAATAACCTGGCCTACTTCTGGCTGGCGCCGACGCTGGTG

TATCAGCCTGTTTATCCGCGCACGGCATCCATCCGGTGGTCTTTTGTTGCAAAGCGCCTT

GCAGAGTTCGTGGGCCTGGCAATGTTCATTTGGCTTCTGTCTGCGCAGTATGCTGCGCCA

GTTCTGCGCAACTCGATCGATAAGATCGCAGTAATGGACCTTACATCCATTTTGGAGCGA

GTCATGAAACTTTCCACGATCTCGCTGATCATTTGGCTTGCTGGTTTCTTTGCATTGTTC

CAAGCTCTTTTGAACGCTTTGGCCGAGGTCATGCGGTTTGGAGATCGCGAGTTCTACACT

GACTGGTGGAACAGCTCCAGTCTGGGTATGTATTGGCGATCTTGGAACCGACCTGTTTAC

CTGTTCATGAAGAGACATGTCTACTCGCCGCTGGTGGGCCGTGGATGGAGTCCTCTGGCT

GCGAGTGGAATGGTTTTCACGCTCTCCGCGATTCTTCATGAAATGCTTGTTGGCATTCCC

ACACATAACTTCATTGGTGTCGCGTTCTTTGGAATGATGTTCCAGCTGCCGTTAATTGGC

CTCACCGCACCATTGGATAAAATGCGTGGGCCTGAAGGCAGGGTCATCGGCAACTGCATC

TTCTGGGTCAGCTTTTGTCTAGTCGGACAACCGCTTGGAGCGCTCCTGTACTTCTTCGCT

TGGCAGGCCAAGTACGGGAGT

>CL122.Contig7_All 198 1661 minus strand hypothetical protein PDIP_85010 [Penicillium digitatum Pd1] >gi|425775174|gb|EKV13456.1| hypothetical protein PDIG_38380 [Penicillium digitatum PHI26]

ATGACTGCTCGCATTGGTGCAGTTACGAGCGCTCGCGAAGAATCTCAGGTCAATTCAACG

AACGGGGCCACTCCGGATGTGACCAAGGTCTCTCCGCCATCAACTTCAACTTCCAAGAGA

CGCAGTAAATATCGCCATGTAGCTGCATACCACTCAGAGGCTCGTCATTCTAGCCTCAGT

CGAGAGGCTGAAGTGCTTCCCAACTTCCTGGGGCTTCGAAATCTCATGGTGTTGGTACTT

GTGGCGATGAACCTGCGATTGATTATTGAGAACTTCATGAAATATGGCGTGCTAATCTGT

ATTAAATGCCATGACTACCGCAGACAGGACGTTGTTTTGGGGTCTATTTTGTTTGCCTCG

GTTCCGTGCCATTTGCTCGTGGCATACATCATTGAACTTTCAGCCGCTACTGCAGCTAAG

CAGACAGTTGGTCGTCAGAAGAAGACAGAAAAGGAGAAAGAGCACGACCAAAAGGTCTTC

CAGTCGACGTGGCCGTACACTGCTTTCCTACACACGCTCAATGCCACGCTATGTCTCACA

GTAACCAGCTTCGTGGTGTATTTCTACATCCATCACCCTGGCATTGGGACATTATGCCAG

CTACACGCACTCATTGTATGGTTAAAGAACTGCTCTTATGCCTTCACAAACCGAGACCTT

CGCCTCGCTATGCTCAATCCTTCTGCAGACCCGGGCTTGCCCGAAATCTACTCCTCGTGT

CCTTACCCAAGAAACATTACCATCAATAACCTGGCCTACTTCTGGCTGGCGCCGACGCTG

GTGTATCAGCCTGTTTATCCGCGCACGGCATCCATCCGGTGGTCTTTTGTTGCAAAGCGC

CTTGCAGAGTTCGTGGGCCTGGCAATGTTCATTTGGCTTCTGTCTGCGCAGTATGCTGCG

CCAGTTCTGCGCAACTCGATCGATAAGATCGCAGTAATGGACCTTACATCCATTTTGGAG

CGAGTCATGAAACTTTCCACGATCTCGCTGATCATTTGGCTTGCTGGTTTCTTTGCATTG

TTCCAAGCTCTTTTGAACGCTTTGGCCGAGGTCATGCGGTTTGGAGATCGCGAGTTCTAC

ACTGACTGGTGGAACAGCTCCAGTCTGGGTATGTATTGGCGATCTTGGAACCGACCTGTT

TACCTGTTCATGAAGAGACATGTCTACTCGCCGCTGGTGGGCCGTGGATGGAGTCCTCTG

GCTGCGAGTGGAATGGTTTTCACGCTCTCCGCGATTCTTCATGAAATGCTTGTTGGCATT

CCCACACATAACTTCATTGGTGTCGCGTTCTTTGGAATGATGTTCCAGCTGCCGTTAATT

GGCCTCACCGCACCATTGGATAAAATGCGTGGGCCTGAAGGCAGGGTCATCGGCAACTGC

ATCTTCTGGGTCAGCTTTTGTCTAGTCGGACAACCGCTTGGAGCGCTCCTGTACTTCTTC

GCTTGGCAGGCCAAGTACGGGAGT

>CL124.Contig1_All 212 3295 Ubiquitin-activating enzyme E1 [Penicillium digitatum Pd1] >gi|425769234|gb|EKV07734.1| Ubiquitin-activating enzyme E1 [Penicillium digitatum PHI26]

ATGCAGGTTGAGAATCCGCAAGAAACCATCGAAGCAATCAAGCATGGGGAGATCGACGAA

TCATTGTATAGTCGGCAGCTATATGTTCTTGGCCACGAGGCCATGAAGCGTATGGGATCT

TCCAATGTTCTGATTGTAGGCCTCAAGGGCCTGGGTGTGGAAATCGCCAAGAACATCGCC

CTCGCTGGCGTCAAGTCTCTGACCCTATACGATCCCGCACCAGTCGCTATCTCGGATCTG

TCTTCGCAGTTTTTCCTTCAGCCAGAGGACGTCGGCAAGCCCCGTGCTGAAGTTACCGCC

CCAAGGGTTGCCGAGTTGAACTCCTATGTCCCCGTTACAGTCCATGAGAGCAAGAGCCTC

GTGGGCGATCTCGAACAACTAAAGCGCTACCAGGCAGTGGTGCTCACACAGACTCCTTTG

AAGGAGCAACTGGTGATTGCCGACTTCTGCCACCAGAACAAAATTTACCTCACAATAACG

GATACCTTCGGTCTCTTTGGCTATATCTTCAATGACTTTGGAAAAAACTTCACTGTGGGT

GATCCAAACGGCGAAGAGCCAGCTGGTGGGATTGTAGCGGATATCAATGACGAAGGGTTG

GTTTCGGCGCTCGATGAGACAAGACATGGACTTGAGGATGGAGACTTTGTCACTTTCACT

GAAGTCAAGGGTATGGATGGCCTAAATAACAGTGATCCTCGCAAAGTCACTGTGAAAGGT

CCCTACACTTTCACTATTGGCGACGTCTCTAGTCTAGGATCCTATAAGGGCGGTGGTCTA

TTTACTCAGGTCAAGATGCCCAAGTTTATTGATTTCCAGCCACTAGAGGACCAACTTAAA

AAGCCTGAGCTTCTCATCTCTGACTCTGCCAAGTTTGACAGACCACAACAACTACACATT

GGTATCCAGGCTCTTCATAAGTTTGCGGAGACTCATGATGGCCAGTTGCCTCGTCCCCAC

AGTGATAGCGATGCCCAGGAGGTCCTAAAGATCGCTAACGATCTTGCTGCGGCCGGTGAG

GAAAAAATTGAACTGGATGAAAAGATTATCAAGGAGCTGAGCTACCAGGCCCGCGGTGAT

CTGAATCCGTTAGCAGCTTTCTTCGGTGGCATCGCCGCCCAGGAAGTTCTCAAGGCTGTC

TCCGGCAAGTTCAGTCCTGTGCATCAGTGGTTGTACTTTGACTCTTTGGAGTCCTTGCCT

ACATCGGTCACTCGATCCGAAGAAAGCTGTAAACCTCTTGGTATCCGCTACGATGGTCAA

ATTGCGGTATTTGGCAAGGAATACCAAGAAAAACTTGCTAATGTCACCCAATTTTTGGTC

GGTTCCGGTGCTATCGGTTGCGAGACGCTGAAGAATTGGGCCATGATGGGTCTGGGGACG

GGTCCTAAAGGAAAGCTTTACGTCACCGATATGGACCAGATTGAGAAGAGCAACCTCAAC

CGCCAGTTCCTCTTCCGTCCCAAAGACGTCGGTAGACTCAAGAGTGAATGCGCATCTGCA

GCCGCTCAAGCAATGAACCGAGAATTGAAAGATAAGATTGTCACTCTTCGTGATCGCGTT

GGTGCCGACACTGAACACGTCTTCAATGAGGATTTCTGGAACGGCCTGGATGGTGTGACA

AACGCTCTGGACAATTTGGATGCACGGACATATGTTGATCGCCGCTGTGTCTTCTTCCGC

AAGCCATTGCTAGAGAGTGGTACCCTGGGCACCAAGTGCAACACCCAGGTCGTGCTTCCT

TTTATCACGGAGTCCTACTCCAGCTCCCAGGATCCCCCAGAAAAGTCATTCCCTATGTGT

ACCCTGAAGAGTTTCCCGAACCGAATTGAGCACACCATTGCATGGGCACGGGATGTCTTC

CAAACCTACTTCGTTGGCCCACCCGAATCAGTCAACATGTATCTCTCGCAATCCGATTAC

ATTCAGCAGACCCTTAAGCAGGCGGGTAACGAAAAGCAAACCTTGGAGCACCTGCGTGAC

TTCTTGGTCACTGAAAAACCTCTGACTTTCGATGATTGTATTGTGTGGGCTCGCCAACAA

TTTGAAGCACAGTACAACAACGCGATCCAGCAGCTTCTCTACAACTTCCCCCGAGACTCC

AAAACTTCAACCGGTCAATTATTCTGGTCTGGGCCTAAGCGTGCCCCTACACCCTTAAAG

TTCGATAGCACTAATCCCACTCATCTCGGATTTGTTGTTGCTGGTGCTAACCTCCATGCG

TTCAACTACGGTATCAAGAACCCCGGTGCGGACAAGGACTACTACCGTAGGGTAGTTGAT

GACATGATTGTTCCCGAGTTCACGCCGAGCTCGAATGTCAAGATTCAGGCCAATGAAAAC

GATCCTGATCCGAATGCGCAGCCAGCTGGTTCGTCTACTGACGAGGAAGAAATCCAGAAA

CTGGTGGCGTCTCTTCCCTCGCCCAAGTCTCTTGCTGGATTCCGCTTGCAGCCAGTTGAG

TTTGAGAAGGACGATGACACCAACCACCACATCGACTTCATCACTGCGGCTAGCAACCTC

CGTGCAGATAACTACGAGATTCCTCAGGCTGACCGACACAAGACCAAATTTATCGCTGGA

AAGATCATCCCGGCAATTGCCACCACTACTGCCCTGGCCACCGGTCTTGTTGCCCTCGAA

ATGTACAAAATTGTTGACGGCAAGGACGACATCGAGCAATACAAAAACGGCTTCGTTAAT

TTGGCCCTTCCGCTATTTAGCTTCAGTGAGCCGATCGGTAGCGAAAAGGGCAAATATCAA

GGCAAGCAAGGCGAGGTCACAATTGACAAGCTTTGGGACCGATTTGAGGTGGAAGACCTT

CCCCTCCAGGACTTCCTGGACTTTTTCGCCGAAAAGGGGTTGGAAATCACGATGGTCAGC

TCTGGAGTCAGCCTGTTGTATGCCAGCTTCTATCCCCCATCGAAGGTCAAGGATCGCCTT

CCTCTTCCTATGAGCAAGCTTGTGGAGCACGTCAGCAAGAAGCCAGTCCCTGAACACCAG

AAAAACATCATCTTTGAGGTGACCGCCGAGGATCAGACAGAGGAGGATGTCGAGATTCCT

TACGTGATGGTCAAGCTCACACAT

>CL124.Contig2_All 340 3423 minus strand Ubiquitin-activating enzyme E1 [Penicillium digitatum Pd1] >gi|425769234|gb|EKV07734.1| Ubiquitin-activating enzyme E1 [Penicillium digitatum PHI26]

ATGCAGGTTGAGAATCCGCAAGAAACCATCGAAGCAATCAAGCATGGGGAGATCGACGAA

TCATTGTATAGTCGGCAGCTATATGTTCTTGGCCACGAGGCCATGAAGCGTATGGGATCT

TCCAATGTTCTGATTGTAGGCCTCAAGGGCCTGGGTGTGGAAATCGCCAAGAACATCGCC

CTCGCTGGCGTCAAGTCTCTGACCCTATACGATCCCGCACCAGTCGCTATCTCGGATCTG

TCTTCGCAGTTTTTCCTTCAGCCAGAGGACGTCGGCAAGCCCCGTGCTGAAGTTACCGCC

CCAAGGGTTGCCGAGTTGAACTCCTATGTCCCCGTTACAGTCCATGAGAGCAAGAGCCTC

GTGGGCGATCTCGAACAACTAAAGCGCTACCAGGCAGTGGTGCTCACACAGACTCCTTTG

AAGGAGCAACTGGTGATTGCCGACTTCTGCCACCAGAACAAAATTTACCTCACAATAACG

GATACCTTCGGTCTCTTTGGCTATATCTTCAATGACTTTGGAAAAAACTTCACTGTGGGT

GATCCAAACGGCGAAGAGCCAGCTGGTGGGATTGTAGCGGATATCAATGACGAAGGGTTG

GTTTCGGCGCTCGATGAGACAAGACATGGACTTGAGGATGGAGACTTTGTCACTTTCACT

GAAGTCAAGGGTATGGATGGCCTAAATAACAGTGATCCTCGCAAAGTCACTGTGAAAGGT

CCCTACACTTTCACTATTGGCGACGTCTCTAGTCTAGGATCCTATAAGGGCGGTGGTCTA

TTTACTCAGGTCAAGATGCCCAAGTTTATTGATTTCCAGCCACTAGAGGACCAACTTAAA

AAGCCTGAGCTTCTCATCTCTGACTCTGCCAAGTTTGACAGACCACAACAACTACACATT

GGTATCCAGGCTCTTCATAAGTTTGCGGAGACTCATGATGGCCAGTTGCCTCGTCCCCAC

AGTGATAGCGATGCCCAGGAGGTCCTAAAGATCGCTAACGATCTTGCTGCGGCCGGTGAG

GAAAAAATTGAACTGGATGAAAAGATTATCAAGGAGCTGAGCTACCAGGCCCGCGGTGAT

CTGAATCCGTTAGCAGCTTTCTTCGGTGGCATCGCCGCCCAGGAAGTTCTCAAGGCTGTC

TCCGGCAAGTTCAGTCCTGTGCATCAGTGGTTGTACTTTGACTCTTTGGAGTCCTTGCCT

ACATCGGTCACTCGATCCGAAGAAAGCTGTAAACCTCTTGGTATCCGCTACGATGGTCAA

ATTGCGGTATTTGGCAAGGAATACCAAGAAAAACTTGCTAATGTCACCCAATTTTTGGTC

GGTTCCGGTGCTATCGGTTGCGAGACGCTGAAGAATTGGGCCATGATGGGTCTGGGGACG

GGTCCTAAAGGAAAGCTTTACGTCACCGATATGGACCAGATTGAGAAGAGCAACCTCAAC

CGCCAGTTCCTCTTCCGTCCCAAAGACGTCGGTAGACTCAAGAGTGAATGCGCATCTGCA

GCCGCTCAAGCAATGAACCGAGAATTGAAAGATAAGATTGTCACTCTTCGTGATCGCGTT

GGTGCCGACACTGAACACGTCTTCAATGAGGATTTCTGGAACGGCCTGGATGGTGTGACA

AACGCTCTGGACAATTTGGATGCACGGACATATGTTGATCGCCGCTGTGTCTTCTTCCGC

AAGCCATTGCTAGAGAGTGGTACCCTGGGCACCAAGTGCAACACCCAGGTCGTGCTTCCT

TTTATCACGGAGTCCTACTCCAGCTCCCAGGATCCCCCAGAAAAGTCATTCCCTATGTGT

ACCCTGAAGAGTTTCCCGAACCGAATTGAGCACACCATTGCATGGGCACGGGATGTCTTC

CAAACCTACTTCGTTGGCCCACCCGAATCAGTCAACATGTATCTCTCGCAATCCGATTAC

ATTCAGCAGACCCTTAAGCAGGCGGGTAACGAAAAGCAAACCTTGGAGCACCTGCGTGAC

TTCTTGGTCACTGAAAAACCTCTGACTTTCGATGATTGTATTGTGTGGGCTCGCCAACAA

TTTGAAGCACAGTACAACAACGCGATCCAGCAGCTTCTCTACAACTTCCCCCGAGACTCC

AAAACTTCAACCGGTCAATTATTCTGGTCTGGGCCTAAGCGTGCCCCTACACCCTTAAAG

TTCGATAGCACTAATCCCACTCATCTCGGATTTGTTGTTGCTGGTGCTAACCTCCATGCG

TTCAACTACGGTATCAAGAACCCCGGTGCGGACAAGGACTACTACCGTAGGGTAGTTGAT

GACATGATTGTTCCCGAGTTCACGCCGAGCTCGAATGTCAAGATTCAGGCCAATGAAAAC

GATCCTGATCCGAATGCGCAGCCAGCTGGTTCGTCTACTGACGAGGAAGAAATCCAGAAA

CTGGTGGCGTCTCTTCCCTCGCCCAAGTCTCTTGCTGGATTCCGCTTGCAGCCAGTTGAG

TTTGAGAAGGACGATGACACCAACCACCACATCGACTTCATCACTGCGGCTAGCAACCTC

CGTGCAGATAACTACGAGATTCCTCAGGCTGACCGACACAAGACCAAATTTATCGCTGGA

AAGATCATCCCGGCAATTGCCACCACTACTGCCCTGGCCACCGGTCTTGTTGCCCTCGAA

ATGTACAAAATTGTTGACGGCAAGGACGACATCGAGCAATACAAAAACGGCTTCGTTAAT

TTGGCCCTTCCGCTATTTAGCTTCAGTGAGCCGATCGGTAGCGAAAAGGGCAAATATCAA

GGCAAGCAAGGCGAGGTCACAATTGACAAGCTTTGGGACCGATTTGAGGTGGAAGACCTT

CCCCTCCAGGACTTCCTGGACTTTTTCGCCGAAAAGGGGTTGGAAATCACGATGGTCAGC

TCTGGAGTCAGCCTGTTGTATGCCAGCTTCTATCCCCCATCGAAGGTCAAGGATCGCCTT

CCTCTTCCTATGAGCAAGCTTGTGGAGCACGTCAGCAAGAAGCCAGTCCCTGAACACCAG

AAAAACATCATCTTTGAGGTGACCGCCGAGGATCAGACAGAGGAGGATGTCGAGATTCCT

TACGTGATGGTCAAGCTCACACAT

>CL125.Contig1_All 141 1661 minus strand hypothetical protein PDIP_74570 [Penicillium digitatum Pd1] >gi|425770187|gb|EKV08660.1| hypothetical protein PDIG_65240 [Penicillium digitatum PHI26]

ATGACCCACCCCGACATTTCTGTTGACGTTCTCGTCATTGGTGCTGGCCCAACTGGTTTG

GGTGCCGCCAAACGTTTGAATCAGATTAATGGCCCCTCATGGATGATTATCGACTCAAAT

GAGATCCCCGGTGGTTTGGCTTCCACTGATGTTACCCCCGAAGGTTTCCTGTACGATGTC

GGTGGTCACGTTATCTTCTCCCACTACAAGTACTTCGACGACTGCATTAACGAGGCCCTC

CCCAAAGAAGAAGATTGGTACACACACGAGCGTATCTCCTACGTTCGCTGCCAGGAGCAA

TGGGTTCCCTACCCCTTCCAGAACAACATTTCTATGCTGCCCAAGGAAGAGCAGGTGAAG

TGCATTGACGGCATGATCGATGCTGCTCTTGAGGCCCGTGTCTCCAACACGAAGCCCAAG

GATTTTGATGAGTGGATTGTCCGCATGATGGGTACTGGTATTGCCGACCTCTTCATGAGA

CCCTACAACTACAAGGTCTGGGCTGTTCCCACCACCAAGATGCAATGCGCTTGGCTCGGT

GAACGCGTCGCTGCCCCCAACCTCAAGGCCGTGACTACCAACGTCATCCTTAACAAGACC

GCCGGTAACTGGGGTCCCAACGCTACCTTCCGTTTCCCCTCCCGCGATGGTACTGGTGGT

ATCTGGATTGCTGTTGCCAACACTCTCCCCAAGGAGAACACCCGCTATGGCCCCAACAAC

AAGGTCGAGAAGGTCAACGCTTACAACAAGACCGTCACTCTCGCCGATGGCACCACCATT

GGTTACGGAAAACTGGTCTCCACCATGGCTGTTGACTATCTCGCGGAGGCCATGAACGAC

ACCGAGCTCATTCCCCTCACCAAGCAGCTCTTCTACTCCTCCACCCACGTTATCGGTGTT

GGTATCCGTGGTGTCCGTCCCGATCGCATCGGCGACAAGTGCTGGCTGTACTTCCCCGAG

GATGATTGCCCATTCTACCGTGCTACCATCTTCTCCAACTACTCCCCCAACAACCAGCCC

GAAGCCTCCAAGAAGTTGCCCACCCTTCAGCTCGCTGACGGCTCCAAGCCTGAGAGCACC

GAGGCTCAGGAAGGTCCCTACTGGTCTGTCATGTTGGAGGTTTCCGAATCTTCCCTGAAG

CCCGTCAACCACGAGACCCTCCTTGCCGAATCCATCCAGGGCCTTGTCAACACCCAGATG

CTTCTGCCCGGTGATGAGATTGTCTCCACCTACCACCGCCGCTTCGACCACGGTTACCCC

ACCCCCAGCCTGGAGCGTGAGGGTGCCCTCACCCAGATTCTCCCCAAGCTCCAGGAGAAG

GGTATCTGGTCCCGTGGCCGCTTCGGTAGCTGGCGCTACGAGGTCGGCAACCAGGACCAC

TCCTTCATGCTCGGTGTCGAGGCCGTTGACAACATTGTCAACGGTGCCGTTGAGTTGACC

CTCAACTACCCCGACTTTGTCAACGGCCGCCAGAACAACGAGCGCCGCTTGGTCGACGGT

GCCCAGGCTTTCGCGAAGAAA

>CL127.Contig1_All 19 987 minus strand Pc22g10110 [Penicillium chrysogenum Wisconsin 54-1255] >gi|211592037|emb|CAP98299.1| Pc22g10110 [Penicillium chrysogenum Wisconsin 54-1255]

ATTTCATGTCATGCTAACAGGGCGATTACAGGTACTCCGCAAGAACAGATATCCACGTCT

TTTTCTAATATCGCCCATCTTCTGATCATTGTGTCCCACTATCTCTCCTTGAAACTTCCC

GCCGAAATTACGCTGCCCCACAAAAATTATCCCGTGCCCACAATCTATACCCCTTCAGCA

TCATATCGCTCGCGTGACGGTCATGATGGAGCTGACTTCCAGTCTTCGTCGAGTCCAGCT

GCATCAAGGACGGTTGACCCGCGCAACCATACACCACGTCCGCGACCTCTTTCCATTGAT

AAGCCTCTACCCCGGTTGGCGAAGGAGGATCCCGCAGCATACGCCTTCTTTCTTGAAGGA

GCAACCCTCCTGGCATGGAACGTTGCATGGTTATGTCGAACTCAAGGGATAAACCTCTCA

TCGGATTCTTGGGAGGAAGTCTGCGATATTGGGAAAAGCTTGTGGCAACTGCTAGTTGCT

CCGCCAGCCCATCCTTCAACTCTGATGCGAGCTTTTGCCGGCCGAGACACGCAGACACAA

ATTAAATCTGCCAAAGACACTCCTAGAACCACGATTCAGCGGACAACATCTTTTCCCATG

TTGGGTCATTATTCTCATGGCACAGCACATTCTTTCCTGGGAGCATCAGAAGGTGTTGAA

TTCATGCGAATGTGGAAACTTCCAACCCCAACCAAGATTGTGGATAAATTGAAGTCCAAT

CTTCTTGGGGAAATGGCCAGTGCAGAATGGGAATTGTTAGAAGAAATAGAGTGGGACGAT

GCGGCCATGAAGTCTCCTCGGCCATCTGCTGCGCAAAACCCCAAGGTCTTTATCCCCTCT

CAATCCGGAACGGTGGAAACAGACAGGAGTTTAAAGACACGTTCTAGGAGGACACCAGGC

CATGAGGATGGTTCTTCCCCGCGACTCGGAGGTACGAAAGGATGGACTAAGGTAGCAAGT

AGAGGACAC

>CL127.Contig2_All 2 1039 minus strand Pc22g10110 [Penicillium chrysogenum Wisconsin 54-1255] >gi|211592037|emb|CAP98299.1| Pc22g10110 [Penicillium chrysogenum Wisconsin 54-1255]

GCCAATCTCTATGGCCTGCGGAAGGCTACGAAGAAGGGCCAACGTCGGGAGACATATGTT

CTTGGGTGTGGTTTTATTGTTGACTTGAGGGATATGAATGGTACTCCGCAAGAACAGATA

TCCACGTCTTTTTCTAATATCGCCCATCTTCTGATCATTGTGTCCCACTATCTCTCCTTG

AAACTTCCCGCCGAAATTACGCTGCCCCACAAAAATTATCCCGTGCCCACAATCTATACC

CCTTCAGCATCATATCGCTCGCGTGACGGTCATGATGGAGCTGACTTCCAGTCTTCGTCG

AGTCCAGCTGCATCAAGGACGGTTGACCCGCGCAACCATACACCACGTCCGCGACCTCTT

TCCATTGATAAGCCTCTACCCCGGTTGGCGAAGGAGGATCCCGCAGCATACGCCTTCTTT

CTTGAAGGAGCAACCCTCCTGGCATGGAACGTTGCATGGTTATGTCGAACTCAAGGGATA

AACCTCTCATCGGATTCTTGGGAGGAAGTCTGCGATATTGGGAAAAGCTTGTGGCAACTG

CTAGTTGCTCCGCCAGCCCATCCTTCAACTCTGATGCGAGCTTTTGCCGGCCGAGACACG

CAGACACAAATTAAATCTGCCAAAGACACTCCTAGAACCACGATTCAGCGGACAACATCT

TTTCCCATGTTGGGTCATTATTCTCATGGCACAGCACATTCTTTCCTGGGAGCATCAGAA

GGTGTTGAATTCATGCGAATGTGGAAACTTCCAACCCCAACCAAGATTGTGGATAAATTG

AAGTCCAATCTTCTTGGGGAAATGGCCAGTGCAGAATGGGAATTGTTAGAAGAAATAGAG

TGGGACGATGCGGCCATGAAGTCTCCTCGGCCATCTGCTGCGCAAAACCCCAAGGTCTTT

ATCCCCTCTCAATCCGGAACGGTGGAAACAGACAGGAGTTTAAAGACACGTTCTAGGAGG

ACACCAGGCCATGAGGATGGTTCTTCCCCGCGACTCGGAGGTACGAAAGGATGGACTAAG

GTAGCAAGTAGAGGACAC

>CL128.Contig1_All 1 816 minus strand C6 transcription factor, putative [Penicillium digitatum Pd1] >gi|425780266|gb|EKV18281.1| C6 transcription factor, putative [Penicillium digitatum PHI26]

ATTCGAACTTTTTTCCGAATCTTGTCTGGAATGATCTTCATCTTGAAGACTTTCACTTTG

GGTGCTAGAGAAGACGATGTGCGAGTGTCTCTAGACCTGCAGGACCGTACGGTGGAGGCA

CTGCGCAATTATGTCGTGGATGATGTGCACCTGAGCAACACGGTAGCTCGGCTTCTCGAG

CTTCTCACTAGCAGCATTCGGACGCGTTTTCTGCGCTTTGCACCGCATGACCGGGGCGCA

GACGGAGAGGGACATGATCGTACCTCGGGGCCTCATTCGCCATCGCGTGACCATTCAACA

ACTCGTCGTGACGGCCCGAATGCACCTTGGTCCGCTAACCAGGGCCATGATACAACGTCG

GGCGGGTCGGGCCTAGGATATACGGACACTCCTAGCACTGGCCACCCGATAGTGTCAGGG

CATGATCCACTGGCGAACATCCCTGCCCAGCCGATCAACTCATCCAACCTCAATGTATCC

TTCATGCCGCCTCCGCCGTCCGTGTACCACAACTACTATGAGTCAAACTCGACATTTCCC

GCCAACGAGATGGACAGATCATCGCCAAATCAAGCCACGTCATCACAGACCATGGGCGAC

AGCCACCATTCTGCCTCCGGTGCGCTTCCGGATTGGTTTGCTCTGCCTCTTGATCAATTC

TTCAACAGCTCCACGGGGGTTGTTGACCAGGGTCTGGGTGGGACTGGACCCATGCTTGGT

GAGTTCGATATGCTCGAGGTACTCCTCAATGAGGGGTATGATGGGAACACTAACGGTGAG

GGTGAGACCGGGGCCGGACTATCGTCCCAGTATCTG

>CL128.Contig4_All 1422 2555 minus strand C6 transcription factor, putative [Penicillium digitatum Pd1] >gi|425780266|gb|EKV18281.1| C6 transcription factor, putative [Penicillium digitatum PHI26]

CTTTATATAAATTCCCTCGCTCTGCAAGCGGTCGTTGACCGATGGACTACCATGTCCAAT

GAGTCCGCTCAGAGTCAAAACCAGACACAAAATAGCCAACCAGTATCGGGGCCCTCTAAC

AGCAGCAACAGTTGGTTTCAAACGTTGAATGAGCTCTACCGTGTCAATGAGCATTATATT

CAGGAAGTCATCGATTCCTCGCGCAAAATTCTGCAGACTGTATTGGAAGGCCTCGTCCCC

GAAGGTCGTCTGAGACACGCACCTATTCGAACTTTTTTCCGAATCTTGTCTGGAATGATC

TTCATCTTGAAGGTATGTTCTTTGGCGGAACCCCCCAGGAAGCATAATTCTGACTCCCAA

TTATAGACTTTCACTTTGGGTGCTAGAGAAGACGATGTGCGAGTGTCTCTAGACCTGCAG

GACCGTACGGTGGAGGCACTGCGCAATTATGTCGTGGATGATGTGCACCTGAGCAACACG

GTAGCTCGGCTTCTCGAGCTTCTCACTAGCAGCATTCGGACGCGTTTTCTGCGCTTTGCA

CCGCATGACCGGGGCGCAGACGGAGAGGGACATGATCGTACCTCGGGGCCTCATTCGCCA

TCGCGTGACCATTCAACAACTCGTCGTGACGGCCCGAATGCACCTTGGTCCGCTAACCAG

GGCCATGATACAACGTCGGGCGGGTCGGGCCTAGGATATACGGACACTCCTAGCACTGGC

CACCCGATAGTGTCAGGGCATGATCCACTGGCGAACATCCCTGCCCAGCCGATCAACTCA

TCCAACCTCAATGTATCCTTCATGCCGCCTCCGCCGTCCGTGTACCACAACTACTATGAG

TCAAACTCGACATTTCCCGCCAACGAGATGGACAGATCATCGCCAAATCAAGCCACGTCA

TCACAGACCATGGGCGACAGCCACCATTCTGCCTCCGGTGCGCTTCCGGATTGGTTTGCT

CTGCCTCTTGATCAATTCTTCAACAGCTCCACGGGGGTTGTTGACCAGGGTCTGGGTGGG

ACTGGACCCATGCTTGGTGAGTTCGATATGCTCGAGGTACTCCTCAATGAGGGGTATGAT

GGGAACACTAACGGTGAGGGTGAGACCGGGGCCGGACTATCGTCCCAGTATCTG

>CL128.Contig5_All 119 229 minus strand C6 transcription factor, putative [Penicillium digitatum Pd1] >gi|425780266|gb|EKV18281.1| C6 transcription factor, putative [Penicillium digitatum PHI26]

ATGGGGGATAGTAGTGTGTACAGCAAGGCGGAGTCTGATGCACACCATCGCCGAGGATAT

CAGGCCTGTGATCCATGTCGCAAACGCAAGGTCAAATGTGACCTAGGAAGT

>CL130.Contig1_All 134 1009 hypothetical protein PDIP_46010 [Penicillium digitatum Pd1]

ATGGCCAAGAAAGGAGGAAAGAAGAACAAGAAGGGCAGCAAGCCCGCTGTTGCCGCTGTA

GACAACGTCAAGGACGTGGTTGAACCCGATAACGAAACTGTGGACGAGACCAACCACACT

GAAGGCCAGATTGAGACCGTCGAGCCGACAGAGACGCCCGAGATTGCAACACAACCTTCC

GAGACCGTTGCCGAGCCCACCCCCGCGACCGAGGCCTCCGGAACTGAGATCCCAGCTGTG

GTGGAGACCGAGGAAGTCAAGACCAAGGAGGCTGTCAAGGCTGAGGAAGCACCCGCGACA

GAGAACAAGGGTACGGTGGAGGAGGCCGTGGAGAAGGCGCAGGCCTCCAAGGCCGGTCAG

TTGGGCGAATTGGAAGGTGGCGCTGCCGCCGGCACTGCTGTCCTCCCGGAGACGACCACC

AAGGAGCCCGCTTCCTTGGCCGCGACCGGCATTCCCGAGACATTGGCCGAGCGCCCCACG

ACTGCGGCATCAACCGAAGTTCCTGCTGTTGTTGCGCCCGCGCCTATTGCTGCCCCCGTC

GAGACCGATGCTATCCACCCCAAGCGCCCGTATGAGAAGCCCATCTTCAACAACGAGGAG

AACTTGAAACCCCACAAGATGCCCAAGACCGACGAGGAGGCGCTCGCCCACAGCGTGGCG

GAGGACAAGAAGACTATTGAGGTTTTGGCCGGTGCTGGACCCGCTTCCACTGCTGCGTTC

ACAACCCCTGAGCCTGTGCCTGTTCAGGCCGAGGCACGCAAGCTTGCCGAGACCCCTCAG

ATCGCTGAGGAGAAGGTCGAAACTCCCGTGATTACTGAGGAGAAGGCCGAGGCACCCAAG

GCTGTTGAAGAGAAGGACGTCGAGACTCCTGTGGCC

>CL130.Contig2_All 3 224 minus strand Pc22g00940 [Penicillium chrysogenum Wisconsin 54-1255] >gi|211591156|emb|CAP97382.1| Pc22g00940 [Penicillium chrysogenum Wisconsin 54-1255]

AACAAGAAGGGCAGCAAGCCCGTCGCCGCTGCTGTAGAGACTGTCAAGGACGTGGTTGAA

CCCGGTCAAACCGAGGAGCAGATCAAGCAGACCGAAGGTCAGACTGAGACCGCCGAGCAA

ACCGTCACGCCCGAGACTGCGACACAGCCTACCGAGACCGTCGCCGAGCCCATCACCGAG

CCCGTCAAGGCGCCTGAGGCCACCCCCGAGACCGAAGCCCCC

>CL130.Contig3_All 182 1030 hypothetical protein PDIP_46010 [Penicillium digitatum Pd1]

ATGGCCAAGAAAGGAGGAAAGAAGAACAAGAAGGGCAGCAAGCCCGCTGTTGCCGCTGTA

GACAACGTCAAGGACGTGGTTGAACCCGATAACGAAACTGTGGACGAGACCAACCACACT

GAAGGCCAGATTGAGACCGTCGAGCCGACAGAGACGCCCGAGATTGCAACACAACCTTCC

GAGACCGTTGCCGAGCCCACCCCCGCGACCGAGGCCTCCGGAACTGAGATCCCAGCTGTG

GTGGAGACCGAGGAAGTCAAGACCAAGGAGGCTGTCAAGGCTGAGGAAGCACCCGCGACA

GAGAACAAGGGTACGGTGGAGGAGGCCGTGGAGAAGGCGCAGGCCTCCAAGGCCGGTCAG

TTGGGCGAATTGGAAGGTGGCGCTGCCGCCGGCACTGCTGTCCTCCCGGAGACGACCACC

AAGGAGCCCGCTTCCTTGGCCGCGACCGGCATTCCCGAGACATTGGCCGAGCGCCCCACG

ACTGCGGCATCAACCGAAGTTCCTGCTGTTGTTGCGCCCGCGCCTATTGCTGCCCCCGTC

GAGACCGATGCTATCCACCCCAAGCGCCCGTATGAGAAGCCCATCTTCAACAACGAGGAG

AACTTGAAACCCCACAAGATGCCCAAGACCGACGAGGAGGCGCTCGCCCACAGCGTGGCG

GAGGACAAGAAGACTATTGAGGTTTTGGCCGGTGCTGGACCCGCTTCCACTGCTGCGTTC

ACAACCCCTGAGCCTGTGCCTGTTCAGGCCGAGGCACGCAAGCTTGCCGAGACCCCTCAG

ATCGCTGAGGAGAAGGTCGAAACTCCCGTGATTACTGAGGAGAAGGCCGAGGCACCCAAG

GCTGTTGAA

>CL130.Contig4_All 182 298 hypothetical protein PDIP_46010 [Penicillium digitatum Pd1]

ATGGCCAAGAAAGGAGGAAAGAAGAACAAGAAGGGCAGCAAGCCCGCTGTTGCCGCTGTA

GACAACGTCAAGGACGTGGTGTGAGTTGAAAATTTCGAAATCATGCCATATCACCAT

>CL130.Contig5_All 134 250 hypothetical protein PDIP_46010 [Penicillium digitatum Pd1]

ATGGCCAAGAAAGGAGGAAAGAAGAACAAGAAGGGCAGCAAGCCCGCTGTTGCCGCTGTA

GACAACGTCAAGGACGTGGTGTGAGTTGAAAATTTCGAAATCATGCCATATCACCAT

>CL131.Contig1_All 3 107 major facilitator superfamily transporter 3 [Penicillium digitatum]

TCTATCAAGGCCGGGTGGTATAGCTCTCTTTCGCAGTATGCAGGATTTTTTCTCGTTCCC

TGCTTGGGCGTTTTCATTGATGTCCTCGGCAACCGAGCATCCATA

>CL131.Contig2_All 3 377 major facilitator superfamily transporter 3 [Penicillium digitatum]

TCTATCAAGGCCGGGTGGTATAGCTCTCTTTCGCAGTATGCAGGTATGATATACCAGGAA

TGCATAGTCAAAGTGGCTCGATAAAAGTTAACTTAAGCCAGGATTTTTTCTCGTTCCCTG

CTTGGGCGTTTTCATTGATGTCCTCGGCAACCGAGCATCCATATGTGAGTTTGACCTAAG

GGTTCTTGAACAAGCCGGATACGACTGATGCTGATCTCCTTAGTATTTGCCTGCGGTCTC

GGCATGCTGTTGAGCATGGTTCTCATTAACTTTGCAACATCAACGGCCGGCACAGGAGCT

GCATTTGGCATCTACGCCCTTGCTGTGTCCCTCGGACCCACCTCAGTCATCGACAGCGTT

CGCACCACACTCTGG

>CL133.Contig1_All 72 254 hypothetical protein PDIP_71230 [Penicillium digitatum Pd1] >gi|425771120|gb|EKV09574.1| hypothetical protein PDIG_61840 [Penicillium digitatum PHI26]

GGTACCAACCGGATCGATCGGTTCCAGCGTGTGCCAAGCGAACTGTACAAGTATCTAGAA

TACATGGCGCACATAAAGGCGGAGTATACATCCACGATGAGATTTGTGGTCAAGGAACGG

TTGGGCTGGGGGGATGTCAATTGGGAAGATCTCAAGCCAAGAGGGGGTCCTTTTGAATAT

AAG

>CL133.Contig2_All 72 575 hypothetical protein PDIP_71230 [Penicillium digitatum Pd1] >gi|425771120|gb|EKV09574.1| hypothetical protein PDIG_61840 [Penicillium digitatum PHI26]

GGTACCAACCGGATCGATCGGTTCCAGCGTGTGCCAAGCGAACTGTACAAGTATCTAGAA

TACATGGCGCACATAAAGGCGGAGTATACATCCACGATGAGATTTGTGGTCAAGGAACGG

TTGGGCTGGGGGGATGTCAATTGGGAAGATCTCAAGCCAAGAGGGGGTCCTTTTGAATAT

AAGGAGGACATCCGAATCATATACAACGACTGGCCATATGGTGTCGAAAAAGACATCATC

CATCTGGTCGTGTGGACCAAATTCGAGCTGGAAGATGACCCAGCCACAGATGATCTAACG

GCAAGTGCACGACGGACCATAGAGAGCTATGTGCAGGACACATTTTGCTCTCGAGTACCA

CCCAACCAGGTGGTCTGGTTCAAGAACTGGAAGTCACTCAAGTCAGTTCCCGGGATAGAA

CACTTCCACGTCATGCTCCATCGTCCCGATATGGCATTTGTTAGGGAGATCACTCTGGGA

GATGTACCTCTGATAGAGCGGCTA

>CL133.Contig3_All 103 870 hypothetical protein PDIP_71230 [Penicillium digitatum Pd1] >gi|425771120|gb|EKV09574.1| hypothetical protein PDIG_61840 [Penicillium digitatum PHI26]

ATGCTTAACTTTCAGCGGTTATCCATGACCCCAATCATCCTCGACGAGACATTGACTGCC

GATGCCGATGCGGCCCTGCACTTCCACGATCACGGCGCACCCTCTGAACCCCTCCCATAT

TGGCTCGTCAATTTACCTCGGTCGCAATGGACCGCAGAATGCCCTAGCTTTCTACGTGAC

CAATCTCCGAAGAATGTCCAATGCCTCTCCACCCCCAATCATCTTTATACTCGACAGAAC

TGGGAGCAGGTGAAGGAGATCATAGGTACCAACCGGATCGATCGGTTCCAGCGTGTGCCA

AGCGAACTGTACAAGTATCTAGAATACATGGCGCACATAAAGGCGGAGTATACATCCACG

ATGAGATTTGTGGTCAAGGAACGGTTGGGCTGGGGGGATGTCAATTGGGAAGATCTCAAG

CCAAGAGGGGGTCCTTTTGAATATAAGGAGGACATCCGAATCATATACAACGACTGGCCA

TATGGTGTCGAAAAAGACATCATCCATCTGGTCGTGTGGACCAAATTCGAGCTGGAAGAT

GACCCAGCCACAGATGATCTAACGGCAAGTGCACGACGGACCATAGAGAGCTATGTGCAG

GACACATTTTGCTCTCGAGTACCACCCAACCAGGTGGTCTGGTTCAAGAACTGGAAGTCA

CTCAAGTCAGTTCCCGGGATAGAACACTTCCACGTCATGCTCCATCGTCCCGATATGGCA

TTTGTTAGGGAGATCACTCTGGGAGATGTACCTCTGATAGAGCGGCTA

>CL133.Contig4_All 606 929 hypothetical protein PDIP_71230 [Penicillium digitatum Pd1] >gi|425771120|gb|EKV09574.1| hypothetical protein PDIG_61840 [Penicillium digitatum PHI26]

AAAGAGGACATCCGAATCATATACAACGACTGGCCATATGGTGTCGAAAAAGACATCATC

CATCTGGTCGTGTGGACCAAATTCGAGCTGGAAGATGACCCAGCCACAGATGATCTAACG

GCAAGTGCACGACGGACCATAGAGAGCTATGTGCAGGACACATTTTGCTCTCGAGTACCA

CCCAACCAGGTGGTCTGGTTCAAGAACTGGAAGTCACTCAAGTCAGTTCCCGGGATAGAA

CACTTCCACGTCATGCTCCATCGTCCCGATATGGCATTTGTTAGGGAGATCACTCTGGGA

GATGTACCTCTGATAGAGCGGCTA

>CL134.Contig1_All 1 297 Pc12g09020 [Penicillium chrysogenum Wisconsin 54-1255] >gi|211582349|emb|CAP80529.1| Pc12g09020 [Penicillium chrysogenum Wisconsin 54-1255]

CAGCCTGTTACAAACCTCAGGATCCTGAAACGGGTTGGGCAGCTGGGTGTTGATCGTGCT

GAATGGTCAAAGAATTTGATGGAGGATGGTTTTAGGGCTTATGAGGCTGTTGTGAAGGGT

TCGGCGGGGAAGTTTAGCGTTGGGGATTCCATTACCATTGCGGATTTGTGTTTGATTCCT

GCTGCTTGGGGAGCTCAGCGGTTTGGTGTTGACTTGGGTCAGTTCCCGGTTACGAATGGG

ATTGTGAAGAACTTGGAGATGGAGGAGGCTGTTAAGAAGGGGCATTGGAGGTCACAG

>CL134.Contig2_All 3 260 minus strand Maleylacetoacetate isomerase MaiA [Penicillium digitatum Pd1] >gi|425780509|gb|EKV18515.1| Maleylacetoacetate isomerase MaiA [Penicillium digitatum PHI26]

AACCTCAGGATCCTGAAGCGGGTTGGGCCGCTGGGTGTTGATCGTGCTGAGTGGTCAACG

GGTTTAGTAGAGGATGGGCTCAGGGCTTATGAGGCTGTTGTTAGAAAATCAGCTGGGAAG

TTTAGCGTTGGAGATTTCATCACAATAGCTGATTTGTGTTTAATTCCTGCTGTCTGGGGA

GCTCAGCGGCTTGGTGTTGACTTGGGTCAGTTTCCCATTACGAATGAGATTGTGAAGAAC

CTGGAGATGGAGGAGGCC

>CL135.Contig1_All 276 1511 minus strand SNARE complex subunit (Tlg2), putative [Penicillium digitatum PHI26] >gi|425780871|gb|EKV18866.1| SNARE complex subunit (Tlg2), putative [Penicillium digitatum Pd1]

TACCTCTCCTACCGCCAATCCCTAATTCACCACCCAGCCAAAAAGCCGCACTTCACACCC

ACAAATGGCTTCTCCGACACGCCCTCACACCCTGAAGAAAACAGGCGCCTAATATCCGAA

ACAGAAGAGGATGGCGACATGGTCATCGAGATGGATCTCCTCCCGCCGCGCTGGGTCGAC

GTGCAGGAAGAGGTCTCCGAATTGTTATCCGAGATCGCCCAGAAATCCGCCCAGCTCGAC

AAACTACACCAGAAACACCTGCTCCCAGGATTCGGCGATGAGGAGCTGCGCAAAAAGGAT

GAGGGCGTTATCGAGCGCTTGACGCAGGATGTCACGCGCTCCTTTCATGATTGCCAGCGC

TCCATCATGCGCATAGAGACTATGGTCGGCGAGTCCAAGGCGCATGGCGGTGTTACGAGT

GGGGAGGAGACTATGGCGAAGAACATTCAGATTTCACTTGCTGCGAGGGTGCAGGAGGCT

AGTGCTAGGTTTAGGAAGAAGCAGAGTACTTATTTGCGGAGTATGGGACCCTTGTTTCTT

TTTGTGTTCTTGTTCTTGTTCTTGTTCTCGGCTATTCGAGGCGGAAGCGGTTGGTTCAAA

TGTGCTAATGCGATGGTTTGCTTCTGTGCAGAATTGCGAGATCTAGAGGGTATTGCGACG

CCATTCGATGGCACGCCTACGCCTCTTACCCAAAATCCATACACGGACCCGTCGATGATG

GAGTCAGATGCCGATAGGTCCTTTTCGCAGACGATGTTGCAGGAGACCTCACAACGACAG

ACCGGTCAGAATGATGCTGCTATTGCGCAGCGGGAGCGCGAAATCAACGATATTGCAAAA

GGGATTATTGAGCTCTCGGATATTTTCCGTGAACTGCAGAGCATGATCATCGACCAAGGC

ACCATGCTGGATCGTATAGATTACAACGTTGAGCGGATGGGCACCGAGGTTAAGGCTGCG

GACAAGGAGCTGAAAGTGGCTACGGGTTATCAACAGCGAACGACCAAGCGCAAGATCATG

CTACTTCTCCTGCTGGTCGTGGTGGGAATGATCATTTTGCTGGTTGTGAAACCAAAGAGG

AGCCGTTCAGCGCCACCGCCACCGCCACCACCACCACCTCCTCCAGAGGATGAATCTTCG

AACAATATCCGCTCTGTGTTTGCTTACCGGGGGCGAAGACATCGGTTATCGAACCGCTTC

GCGCGAGATCGGTGGATGGATCCGGATATATTCCGA

>CL135.Contig2_All 106 1254 minus strand SNARE complex subunit (Tlg2), putative [Penicillium digitatum PHI26] >gi|425780871|gb|EKV18866.1| SNARE complex subunit (Tlg2), putative [Penicillium digitatum Pd1]

ATGTGGCGGGATCGTACCAACCTCTACCTCTCCTACCGCCAATCCCTAATTCACCACCCA

GCCAAAAAGCCGCACTTCACACCCACAAATGGCTTCTCCGACACGCCCTCACACCCTGAA

GAAAACAGGCGCCTAATATCCGAAACAGAAGAGGATGGCGACATGGTCATCGAGATGGAT

CTCCTCCCGCCGCGCTGGGTCGACGTGCAGGAAGAGGTCTCCGAATTGTTATCCGAGATC

GCCCAGAAATCCGCCCAGCTCGACAAACTACACCAGAAACACCTGCTCCCAGGATTCGGC

GATGAGGAGCTGCGCAAAAAGGATGAGGGCGTTATCGAGCGCTTGACGCAGGATGTCACG

CGCTCCTTTCATGATTGCCAGCGCTCCATCATGCGCATAGAGACTATGGTCGGCGAGTCC

AAGGCGCATGGCGGTGTTACGAGTGGGGAGGAGACTATGGCGAAGAACATTCAGATTTCA

CTTGCTGCGAGGGTGCAGGAGGCTAGTGCTAGGTTTAGGAAGAAGCAGAGTACTTATTTG

CGGAAATTGCGAGATCTAGAGGGTATTGCGACGCCATTCGATGGCACGCCTACGCCTCTT

ACCCAAAATCCATACACGGACCCGTCGATGATGGAGTCAGATGCCGATAGGTCCTTTTCG

CAGACGATGTTGCAGGAGACCTCACAACGACAGACCGGTCAGAATGATGCTGCTATTGCG

CAGCGGGAGCGCGAAATCAACGATATTGCAAAAGGGATTATTGAGCTCTCGGATATTTTC

CGTGAACTGCAGAGCATGATCATCGACCAAGGCACCATGCTGGATCGTATAGATTACAAC

GTTGAGCGGATGGGCACCGAGGTTAAGGCTGCGGACAAGGAGCTGAAAGTGGCTACGGGT

TATCAACAGCGAACGACCAAGCGCAAGATCATGCTACTTCTCCTGCTGGTCGTGGTGGGA

ATGATCATTTTGCTGGTTGTGAAACCAAAGAGGAGCCGTTCAGCGCCACCGCCACCGCCA

CCACCACCACCTCCTCCAGAGGATGAATCTTCGAACAATATCCGCTCTGTGTTTGCTTAC

CGGGGGCGAAGACATCGGTTATCGAACCGCTTCGCGCGAGATCGGTGGATGGATCCGGAT

ATATTCCGA

>CL135.Contig3_All 276 1400 minus strand SNARE complex subunit (Tlg2), putative [Penicillium digitatum PHI26] >gi|425780871|gb|EKV18866.1| SNARE complex subunit (Tlg2), putative [Penicillium digitatum Pd1]

TACCTCTCCTACCGCCAATCCCTAATTCACCACCCAGCCAAAAAGCCGCACTTCACACCC

ACAAATGGCTTCTCCGACACGCCCTCACACCCTGAAGAAAACAGGCGCCTAATATCCGAA

ACAGAAGAGGATGGCGACATGGTCATCGAGATGGATCTCCTCCCGCCGCGCTGGGTCGAC

GTGCAGGAAGAGGTCTCCGAATTGTTATCCGAGATCGCCCAGAAATCCGCCCAGCTCGAC

AAACTACACCAGAAACACCTGCTCCCAGGATTCGGCGATGAGGAGCTGCGCAAAAAGGAT

GAGGGCGTTATCGAGCGCTTGACGCAGGATGTCACGCGCTCCTTTCATGATTGCCAGCGC

TCCATCATGCGCATAGAGACTATGGTCGGCGAGTCCAAGGCGCATGGCGGTGTTACGAGT

GGGGAGGAGACTATGGCGAAGAACATTCAGATTTCACTTGCTGCGAGGGTGCAGGAGGCT

AGTGCTAGGTTTAGGAAGAAGCAGAGTACTTATTTGCGGAAATTGCGAGATCTAGAGGGT

ATTGCGACGCCATTCGATGGCACGCCTACGCCTCTTACCCAAAATCCATACACGGACCCG

TCGATGATGGAGTCAGATGCCGATAGGTCCTTTTCGCAGACGATGTTGCAGGAGACCTCA

CAACGACAGACCGGTCAGAATGATGCTGCTATTGCGCAGCGGGAGCGCGAAATCAACGAT

ATTGCAAAAGGGATTATTGAGCTCTCGGATATTTTCCGTGAACTGCAGAGCATGATCATC

GACCAAGGCACCATGCTGGATCGTATAGATTACAACGTTGAGCGGATGGGCACCGAGGTT

AAGGCTGCGGACAAGGAGCTGAAAGTGGCTACGGGTTATCAACAGCGAACGACCAAGCGC

AAGATCATGCTACTTCTCCTGCTGGTCGTGGTGGGAATGATCATTTTGCTGGTTGTGAAA

CCAAAGAGGAGCCGTTCAGCGCCACCGCCACCGCCACCACCACCACCTCCTCCAGAGGAT

GAATCTTCGAACAATATCCGCTCTGTGTTTGCTTACCGGGGGCGAAGACATCGGTTATCG

AACCGCTTCGCGCGAGATCGGTGGATGGATCCGGATATATTCCGA

>CL135.Contig4_All 106 1365 minus strand SNARE complex subunit (Tlg2), putative [Penicillium digitatum PHI26] >gi|425780871|gb|EKV18866.1| SNARE complex subunit (Tlg2), putative [Penicillium digitatum Pd1]

ATGTGGCGGGATCGTACCAACCTCTACCTCTCCTACCGCCAATCCCTAATTCACCACCCA

GCCAAAAAGCCGCACTTCACACCCACAAATGGCTTCTCCGACACGCCCTCACACCCTGAA

GAAAACAGGCGCCTAATATCCGAAACAGAAGAGGATGGCGACATGGTCATCGAGATGGAT

CTCCTCCCGCCGCGCTGGGTCGACGTGCAGGAAGAGGTCTCCGAATTGTTATCCGAGATC

GCCCAGAAATCCGCCCAGCTCGACAAACTACACCAGAAACACCTGCTCCCAGGATTCGGC

GATGAGGAGCTGCGCAAAAAGGATGAGGGCGTTATCGAGCGCTTGACGCAGGATGTCACG

CGCTCCTTTCATGATTGCCAGCGCTCCATCATGCGCATAGAGACTATGGTCGGCGAGTCC

AAGGCGCATGGCGGTGTTACGAGTGGGGAGGAGACTATGGCGAAGAACATTCAGATTTCA

CTTGCTGCGAGGGTGCAGGAGGCTAGTGCTAGGTTTAGGAAGAAGCAGAGTACTTATTTG

CGGAGTATGGGACCCTTGTTTCTTTTTGTGTTCTTGTTCTTGTTCTTGTTCTCGGCTATT

CGAGGCGGAAGCGGTTGGTTCAAATGTGCTAATGCGATGGTTTGCTTCTGTGCAGAATTG

CGAGATCTAGAGGGTATTGCGACGCCATTCGATGGCACGCCTACGCCTCTTACCCAAAAT

CCATACACGGACCCGTCGATGATGGAGTCAGATGCCGATAGGTCCTTTTCGCAGACGATG

TTGCAGGAGACCTCACAACGACAGACCGGTCAGAATGATGCTGCTATTGCGCAGCGGGAG

CGCGAAATCAACGATATTGCAAAAGGGATTATTGAGCTCTCGGATATTTTCCGTGAACTG

CAGAGCATGATCATCGACCAAGGCACCATGCTGGATCGTATAGATTACAACGTTGAGCGG

ATGGGCACCGAGGTTAAGGCTGCGGACAAGGAGCTGAAAGTGGCTACGGGTTATCAACAG

CGAACGACCAAGCGCAAGATCATGCTACTTCTCCTGCTGGTCGTGGTGGGAATGATCATT

TTGCTGGTTGTGAAACCAAAGAGGAGCCGTTCAGCGCCACCGCCACCGCCACCACCACCA

CCTCCTCCAGAGGATGAATCTTCGAACAATATCCGCTCTGTGTTTGCTTACCGGGGGCGA

AGACATCGGTTATCGAACCGCTTCGCGCGAGATCGGTGGATGGATCCGGATATATTCCGA

>CL136.Contig1_All 95 646 hypothetical protein PDIG_85560 [Penicillium digitatum PHI26] >gi|425775375|gb|EKV13649.1| hypothetical protein PDIP_47490 [Penicillium digitatum Pd1]

ATGTCTTACGCCGCCGCAGCTGCAAAGGGCCCCAAGCAGTCCCCCGAGGATGCGTGAGTT

TTGTGCGGTATTAAATATCCATTACACACAACTAACATGTACTCCAGCCGTGCTCCCCCG

GTGGACGGGATCTACCACGATGAATCCGAGAGCACGGCCTCATTGATCGACGTCGATGGC

CCCCATGTGCAGACTGTGGAGTCCGACTTCCTCAAGCAAGATGTCCAAACCACCACCCAG

GCGGAGCGGATAGAGCGCGAAGCTGAGGAGAAGGAGAAGCGCGAGGAGGAGGAAAAGAAG

AAGAAGAAGGCTAAGGCCCACAAGGTGAAGAGCAGTAGTATCCGTGGAAACACCAGCAAC

CCTGTCTTCCTTGCCAATGCCGCTATCGCAACCGTGATCGGCGCAGGCCTCAGCTTCGGT

GCCTACAAGCAACACACGAAGGGCAACCTCTCCTGGGAGCTGGTCGGACTCACTGCTGGT

GCTGTTGGAGTCTTCGGTACGGTGGACTACTTTGTCAGCAAATGGTTCCTGCAGAACAAG

TTCCCTCCCAAA

>CL136.Contig2_All 243 794 hypothetical protein PDIG_85560 [Penicillium digitatum PHI26] >gi|425775375|gb|EKV13649.1| hypothetical protein PDIP_47490 [Penicillium digitatum Pd1]

ATGTCTTACGCCGCCGCAGCTGCAAAGGGCCCCAAGCAGTCCCCCGAGGATGCGTGAGTT

TTGTGCGGTATTAAATATCCATTACACACAACTAACATGTACTCCAGCCGTGCTCCCCCG

GTGGACGGGATCTACCACGATGAATCCGAGAGCACGGCCTCATTGATCGACGTCGATGGC

CCCCATGTGCAGACTGTGGAGTCCGACTTCCTCAAGCAAGATGTCCAAACCACCACCCAG

GCGGAGCGGATAGAGCGCGAAGCTGAGGAGAAGGAGAAGCGCGAGGAGGAGGAAAAGAAG

AAGAAGAAGGCTAAGGCCCACAAGGTGAAGAGCAGTAGTATCCGTGGAAACACCAGCAAC

CCTGTCTTCCTTGCCAATGCCGCTATCGCAACCGTGATCGGCGCAGGCCTCAGCTTCGGT

GCCTACAAGCAACACACGAAGGGCAACCTCTCCTGGGAGCTGGTCGGACTCACTGCTGGT

GCTGTTGGAGTCTTCGGTACGGTGGACTACTTTGTCAGCAAATGGTTCCTGCAGAACAAG

TTCCCTCCCAAA

>CL136.Contig3_All 95 592 hypothetical protein PDIG_85560 [Penicillium digitatum PHI26] >gi|425775375|gb|EKV13649.1| hypothetical protein PDIP_47490 [Penicillium digitatum Pd1]

ATGTCTTACGCCGCCGCAGCTGCAAAGGGCCCCAAGCAGTCCCCCGAGGATGCCCGTGCT

CCCCCGGTGGACGGGATCTACCACGATGAATCCGAGAGCACGGCCTCATTGATCGACGTC

GATGGCCCCCATGTGCAGACTGTGGAGTCCGACTTCCTCAAGCAAGATGTCCAAACCACC

ACCCAGGCGGAGCGGATAGAGCGCGAAGCTGAGGAGAAGGAGAAGCGCGAGGAGGAGGAA

AAGAAGAAGAAGAAGGCTAAGGCCCACAAGGTGAAGAGCAGTAGTATCCGTGGAAACACC

AGCAACCCTGTCTTCCTTGCCAATGCCGCTATCGCAACCGTGATCGGCGCAGGCCTCAGC

TTCGGTGCCTACAAGCAACACACGAAGGGCAACCTCTCCTGGGAGCTGGTCGGACTCACT

GCTGGTGCTGTTGGAGTCTTCGGTACGGTGGACTACTTTGTCAGCAAATGGTTCCTGCAG

AACAAGTTCCCTCCCAAA

>CL136.Contig4_All 243 740 hypothetical protein PDIG_85560 [Penicillium digitatum PHI26] >gi|425775375|gb|EKV13649.1| hypothetical protein PDIP_47490 [Penicillium digitatum Pd1]

ATGTCTTACGCCGCCGCAGCTGCAAAGGGCCCCAAGCAGTCCCCCGAGGATGCCCGTGCT

CCCCCGGTGGACGGGATCTACCACGATGAATCCGAGAGCACGGCCTCATTGATCGACGTC

GATGGCCCCCATGTGCAGACTGTGGAGTCCGACTTCCTCAAGCAAGATGTCCAAACCACC

ACCCAGGCGGAGCGGATAGAGCGCGAAGCTGAGGAGAAGGAGAAGCGCGAGGAGGAGGAA

AAGAAGAAGAAGAAGGCTAAGGCCCACAAGGTGAAGAGCAGTAGTATCCGTGGAAACACC

AGCAACCCTGTCTTCCTTGCCAATGCCGCTATCGCAACCGTGATCGGCGCAGGCCTCAGC

TTCGGTGCCTACAAGCAACACACGAAGGGCAACCTCTCCTGGGAGCTGGTCGGACTCACT

GCTGGTGCTGTTGGAGTCTTCGGTACGGTGGACTACTTTGTCAGCAAATGGTTCCTGCAG

AACAAGTTCCCTCCCAAA

>CL137.Contig1_All 2 286 Pc16g10390 [Penicillium chrysogenum Wisconsin 54-1255] >gi|211585972|emb|CAP93709.1| Pc16g10390 [Penicillium chrysogenum Wisconsin 54-1255]

ATTGTCTTGCTGGGTCTTATGGGTGGTGGTAAGTTACCCGAGGGAGTGAATATTGCGCCG

TTGCTGTATAAGCGTGTGCGGATTGAGGGGAGCACGCTGCGCAGTCGGGATCTGGAGTAC

CAGCGGAAATTGCGCGATACGCTTGTTGAGCATGCTTTGCCGCAGTTCTGTGATGGGACG

TTTAAGGTTTTTGTGGAGAAGGTGTTTCCGTTTGAGAAGATTGAGGAGGCGCATAAGTTG

CTCGAGAGTAACACTACTAAGGGGAAGATTATTTGTGTTTTTGAG

>CL137.Contig2_All 78 1073 Quinone oxidoreductase, putative [Penicillium digitatum PHI26] >gi|425782187|gb|EKV20111.1| Quinone oxidoreductase, putative [Penicillium digitatum Pd1]

ATGCGAGCTATCGGCATTAAAGGCGGCAAAGGTCCCGCAGCAAATCTCTTCATCGACCAG

ATCGCCAAGCCCACCGCCGCAGATGGCGAAGCCATCGTCAAGATCCGGGCCTTCGGCCTG

AACCGCATGGATCTGCTTCAGCGTGAGGGCCTGTACCCTCTTCCAGCCCAAGCACCACCA

ATCATGGGCGTAGAGTTCTCCGGCGTGATCGAGAGCTTCGGGCCAGACGGACACGAAGAC

TTCAGGATCGGTGATGAAGTATTCGGTCTTGCATACGGCGGCGCCTATGCAGAGTACATC

GCCGTTTCAATACACATGCTAATGCACAAGCCGAAAGAGCTATCATGGGAAGAGGCCGCC

GGAGTTCCGGAGACTTGGATCACGGCTTCACAAGCGCTGTTCCTAATTGGCGAATTCCAA

TCGGGCCAAACCGTCCTCTGGCACGCTGGCGCATCCTCAGTCTCAATCTCGGGAATCCAA

CTGGCGAAAGACGCCGGCGCAAAAGCCATCTACGCAACAGCGGGCTCACAAGAGAAAATC

GACTTTCTGGAAAAAGAACTCGGTGTGACAAAGGCCTTTAACTACAAGACCCAAGACTGG

GCGGCCGAGATCCAGAGGGCGACGTCTGGCGCAGGTGTCGATCTAACAGTCGATTTCATC

GGCGCGACGTATTTTCAGGGTAATCTTGACGTAGCGGCTCGTGACAGCCGTATCGTCTTG

CTAGGTCTTATGGGTGGCGGCAAGTTACCCGAGGGAGTTAATATTGCGCCGTTACTGTTC

AAGCGTGTTCGAATTGAGGGGAGTACGCTACGCAGTCGGGATCTTGGATACCAGCGGAGA

TTGAGGGATACGCTTGTGGAGCATGCTTTGCCTCGGTTCTGTGATCGGACGTTTAAAGTT

TTTGTGGAGAAAATCTTTCCATTTGGGGAGATTGAGGCGGCCCATACGTTGCTTGAGAGC

AACACTACTAAAGGGAAGATCATTTGTGTTTTGGAG

>CL138.Contig1_All 463 573 minus strand hypothetical protein [Penicillium chrysogenum Wisconsin 54-1255] >gi|211588881|emb|CAP94996.1| hypothetical protein Pc21g00990 [Penicillium chrysogenum Wisconsin 54-1255]

AAGTCTTTGGTTTTCTGCCCTTCATATATCGTCGGTTTTGCTCCGGTAAGGTCACTGTCG

CATATGCTGTCTATTTCTGCTCTTATCAGGTTTCTCTGGATGCTTGCGAAA

>CL138.Contig2_All 519 629 minus strand hypothetical protein [Penicillium chrysogenum Wisconsin 54-1255] >gi|211588881|emb|CAP94996.1| hypothetical protein Pc21g00990 [Penicillium chrysogenum Wisconsin 54-1255]

AAGTCTTTGGTTTTCTGCCCTTCATATATCGTCGGTTTTGCTCCGGTAAGGTCACTGTCG

CATATGCTGTCTATTTCTGCTCTTATCAGGTTTCTCTGGATGCTTGCGAAA

>CL138.Contig3_All 442 552 minus strand hypothetical protein [Penicillium chrysogenum Wisconsin 54-1255] >gi|211588881|emb|CAP94996.1| hypothetical protein Pc21g00990 [Penicillium chrysogenum Wisconsin 54-1255]

AAGTCTTTGGTTTTCTGCCCTTCATATATCGTCGGTTTTGCTCCGGTAAGGTCACTGTCG

CATATGCTGTCTATTTCTGCTCTTATCAGGTTTCTCTGGATGCTTGCGAAA

>CL138.Contig4_All 610 720 minus strand hypothetical protein [Penicillium chrysogenum Wisconsin 54-1255] >gi|211588881|emb|CAP94996.1| hypothetical protein Pc21g00990 [Penicillium chrysogenum Wisconsin 54-1255]

AAGTCTTTGGTTTTCTGCCCTTCATATATCGTCGGTTTTGCTCCGGTAAGGTCACTGTCG

CATATGCTGTCTATTTCTGCTCTTATCAGGTTTCTCTGGATGCTTGCGAAA

>CL138.Contig5_All 540 650 minus strand hypothetical protein [Penicillium chrysogenum Wisconsin 54-1255] >gi|211588881|emb|CAP94996.1| hypothetical protein Pc21g00990 [Penicillium chrysogenum Wisconsin 54-1255]

AAGTCTTTGGTTTTCTGCCCTTCATATATCGTCGGTTTTGCTCCGGTAAGGTCACTGTCG

CATATGCTGTCTATTTCTGCTCTTATCAGGTTTCTCTGGATGCTTGCGAAA

>CL139.Contig1_All 226 801 Pc18g04060 [Penicillium chrysogenum Wisconsin 54-1255] >gi|211586975|emb|CAP94630.1| Pc18g04060 [Penicillium chrysogenum Wisconsin 54-1255]

TCCCCCCCGACATCTAAGCGTCTCAAGACGTCGGCAACTACCAGCGCCCCCCCGCATATG

CTTGCTCAACAACAGATTCATCCATTTCACCGCGTCCCAACCTTTGAAGGCATCCCGATT

CCGACTGCTCCTATACCTCAACAAAACCTCGGCACTTCGCGCAAACGTCCTCCTTCTCCT

ACGGGATCCTCAGCCATGATGGCAGGCCCAGGGAATCCTGCGGGTGGTGGGATTGACGAC

CCCGGTGCCATTCCCACGGTCGAATCTACACCCAAGAAAAAAGGGCGCACAAATACCCCA

TGGACCGCAGAGGAGGAGCAAAGGCTTAAGACAATGCGCGACGCCGGGCGCAGTTGGAGT

GAAATTGCAAAGACTTTCCCGACCCGAACCGAGGGCAGTGTCAAGAAGCATTGGTACAAG

GACATGCACTACGCGGAATTTGCCGAAGATGAGTCTGTGAAGCTTCGTGAGGCGATCAAG

GAGTACGAGGCGAACAAGTGGAAGGTGATCGGTCAGAAAGTGGGAAAGCCTGCTAAAGCC

TGCGAACAGTATGCCAAAGAGCATTTCAAGAACGTC

>CL139.Contig2_All 510 1073 Pc18g04060 [Penicillium chrysogenum Wisconsin 54-1255] >gi|211586975|emb|CAP94630.1| Pc18g04060 [Penicillium chrysogenum Wisconsin 54-1255]

ATGTCTTCCCCCCCGACATCTAAGCGTCTCAAGACGTCGGCAACTACCAGCGCCCCCCCG

CATATGCTTGCTCAACAACAGATTCATCCATTTCACCGCGTCCCAACCTTTGAAGGCATC

CCGATTCCGACTGCTCCTATACCTCAACAAAACCTCGGCACTTCGCGCAAACGTCCTCCT

TCTCCTACGGGATCCTCAGCCATGATGGCAGGCCCAGGGAATCCTGCGGGTGGTGGGATT

GACGACCCCGGTGCCATTCCCACGGTCGAATCTACACCCAAGAAAAAAGGGCGCACAAAT

ACCCCATGGACCGCAGAGGAGGAGCAAAGGCTTAAGACAATGCGCGACGCCGGGCGCAGT

TGGAGTGAAATTGCAAAGACTTTCCCGACCCGAACCGAGGGCAGTGTCAAGAAGCATTGG

TACAAGGACATGCACTACGCGGAATTTGCCGAAGATGAGTCTGTGAAGCTTCGTGAGGCG

ATCAAGGAGTACGAGGCGAACAAGTGGAAGGTGATCGGTCAGAAAGTGGGAAAGCCTGCT

AAAGCCTGCGAACAGTATGCCAAA

>CL141.Contig1_All 93 470 Pc16g09130 [Penicillium chrysogenum Wisconsin 54-1255] >gi|211585854|emb|CAP93583.1| Pc16g09130 [Penicillium chrysogenum Wisconsin 54-1255]

CCATAGAATTGCGATTTCTGCCACAAGCGTTTCTCTCGAGGGTAAATTCCGTTGAAGGTG

CATGAGAATTGTAACAATCTTCAAACCCACATGTTCGATTCCTAAATTTTGCGAGCATAC

AGCGATGTCTTGAGCCGACATGCCAAAAGCCATAATCATGCTGATGCGACAGCCGATTCA

TCAGAAGCGCCACATCGACAGTCATCATTAATTGCGGGTACATCTTCTATGGTCCCCGGA

TCCCGGAATGGGATCACCGGCGGAGAAGCACCGATTCTACCTCCATCTACCAAACTTCGC

GAGCGGCGTCTCTTCGCATCAACAAGGCTCTCTTCGTCCCTGGATTACCCAGCCGACATG

TCCGCCCACTATGGACGG

>CL141.Contig2_All 1 570 hypothetical protein PDIG_51500 [Penicillium digitatum PHI26] >gi|425782115|gb|EKV20044.1| hypothetical protein PDIP_20700 [Penicillium digitatum Pd1]

AACGGAGGAAGATGTGTCTACGACATCTACAAATTCTACATCAGCTATGCTGCGGTCAAT

GTGGCCACGGATGTGTCCATCTTGATGGTTCCAATGCCCATTGTGTGGAAGCTGCAAATG

CCAAGGACACAGAAAATCCTCGTATGCGGTATCCTTTTGATTGGAGGATTTGTCTGCGTT

ACCAGCTTCGTCCGGATTTATTACATTCACTTCCTCAGAGCCCACGATTACACCTGGGTT

GTCGGCAACGTCTTTCTCTGGTCCAGCATTGAGCCCTCCATCGGCATCTTGTGCGCCTGT

CTGCCAACATTGCATCCAATGATCCGCTCCGTTATCTCTCGCGTGTTTGGCATCAGCTCG

AACAGGTATGATTCAAAGAAACAGGAGACAACGAACAAAAGAACCATTGTTCGAAGACAG

AGGCCACTAGACTGGGACGAGACCTTGTTGACCACCCGGGACGTACAAGTCGAGATGAGC

GGAGTCAGGAGAGAATATGGCGAGGATGGGCAGATTACGGTGGACATGGACTTCCGAATT

GTCGAAGAGAGCAATCAATTAAATAAACGT

>CL141.Contig3_All 146 523 Pc16g09130 [Penicillium chrysogenum Wisconsin 54-1255] >gi|211585854|emb|CAP93583.1| Pc16g09130 [Penicillium chrysogenum Wisconsin 54-1255]

CCATAGAATTGCGATTTCTGCCACAAGCGTTTCTCTCGAGGGTAAATTCCGTTGAAGGTG

CATGAGAATTGTAACAATCTTCAAACCCACATGTTCGATTCCTAAATTTTGCGAGCATAC

AGCGATGTCTTGAGCCGACATGCCAAAAGCCATAATCATGCTGATGCGACAGCCGATTCA

TCAGAAGCGCCACATCGACAGTCATCATTAATTGCGGGTACATCTTCTATGGTCCCCGGA

TCCCGGAATGGGATCACCGGCGGAGAAGCACCGATTCTACCTCCATCTACCAAACTTCGC

GAGCGGCGTCTCTTCGCATCAACAAGGCTCTCTTCGTCCCTGGATTACCCAGCCGACATG

TCCGCCCACTATGGACGG

>CL141.Contig4_All 88 660 hypothetical protein PDIG_51500 [Penicillium digitatum PHI26] >gi|425782115|gb|EKV20044.1| hypothetical protein PDIP_20700 [Penicillium digitatum Pd1]

CCCAACGGAGGAAGATGTGTCTACGACATCTACAAATTCTACATCAGCTATGCTGCGGTC

AATGTGGCCACGGATGTGTCCATCTTGATGGTTCCAATGCCCATTGTGTGGAAGCTGCAA

ATGCCAAGGACACAGAAAATCCTCGTATGCGGTATCCTTTTGATTGGAGGATTTGTCTGC

GTTACCAGCTTCGTCCGGATTTATTACATTCACTTCCTCAGAGCCCACGATTACACCTGG

GTTGTCGGCAACGTCTTTCTCTGGTCCAGCATTGAGCCCTCCATCGGCATCTTGTGCGCC

TGTCTGCCAACATTGCATCCAATGATCCGCTCCGTTATCTCTCGCGTGTTTGGCATCAGC

TCGAACAGGTATGATTCAAAGAAACAGGAGACAACGAACAAAAGAACCATTGTTCGAAGA

CAGAGGCCACTAGACTGGGACGAGACCTTGTTGACCACCCGGGACGTACAAGTCGAGATG

AGCGGAGTCAGGAGAGAATATGGCGAGGATGGGCAGATTACGGTGGACATGGACTTCCGA

ATTGTCGAAGAGAGCAATCAATTAAATAAACGT

>CL142.Contig1_All 2 253 minus strand Pc06g00890 [Penicillium chrysogenum Wisconsin 54-1255] >gi|211581311|emb|CAP79082.1| Pc06g00890 [Penicillium chrysogenum Wisconsin 54-1255]

AAGCGTGACGAAGCCGAACGTTCGGGCGACACCCAGACAGCTGCTGACCTCGAATACTAC

GCCATCCCAGAGACCAAGGCCCTCATCGAGCGTCTAGAGGCTGACCGGGCCAGGGCCGAT

GCTGAACGTCGTGCTCAACAAGGCGACGCTGGCGAAGCACTCCTTGCCGACGCAGTTGGT

CCTGATCAGATTAATGAAATCGTTGGCCGGTGGACCGGTATCCCTGTCACGAGACTCAAG

ACCACCGAAAAG

>CL142.Contig2_All 2 352 minus strand Heat shock protein Hsp98/Hsp104/ClpA, putative [Penicillium digitatum PHI26] >gi|425783711|gb|EKV21541.1| Heat shock protein Hsp98/Hsp104/ClpA, putative [Penicillium digitatum Pd1]

ATGGGTGCTGGATCTAGCGGCGAGGGCGGTATGGACGCTGCCAACCTTCTCAAGCCAATG

CTGGCTCGCGGCCAGCTGCACTGCATTGGTGCCACGACTCTCTCTGAATACCGCAAATAC

ATTGAGAAAGATCAGGCCTTCGAGCGACGATTCCAACAGGTCCTTGTCAAGGAACCTACC

GTCCCAGAGACTATCTCTATCCTTCGTGGTCTGAAGGAAAAGTACGAAGCTCACCACGGT

GTCAACATTCTGGATGGCGCGATCGTCACCGCTGCCACTCTCGCGTCTCGCTACCTGACG

GCACGTCGACTACCTGATTCTGCGGTCGATTTGATTGATGAGGCTGCAGCT

>CL142.Contig3_All 152 2947 Heat shock protein Hsp98/Hsp104/ClpA, putative [Penicillium digitatum PHI26] >gi|425783711|gb|EKV21541.1| Heat shock protein Hsp98/Hsp104/ClpA, putative [Penicillium digitatum Pd1]
[truncated: 13,771,519 more chars]
